# Immunogenicity of the Foldon trimerization domain and efforts to shield it

**DOI:** 10.1371/journal.pone.0362571

**Published:** 2026-11-13

**Authors:** John V. Dzimianski, Spencer R. Pierce, Sara M. O’Rourke, Ian Kunkel, Kevin Delgado-Cunningham, Aaron D. Gingerich, Jarrod J. Mousa, Ted M. Ross, Rebecca M. DuBois

**Affiliations:** 1 Department of Biomolecular Engineering, University of California Sant Cruz, Santa Cruz, California, United States of America; 2 Center for Vaccines and Immunology, College of Veterinary Medicine, University of Georgia, Athens, Georgia, United States of America; 3 Department of Infectious Diseases, College of Veterinary Medicine, University of Georgia, Athens, Georgia, United States of America; 4 Florida Research and Innovation Center, Cleveland Clinic, Cleveland, Ohio, United States of America; 5 Department of Biochemistry and Molecular Biology, Franklin College of Arts and Sciences, University of Georgia, Athens, Georgia, United States of America; 6 Department of Biomedical Sciences, College of Medicine, Florida State University, Tallahassee, Florida, United States of America; University of the Pacific, UNITED STATES OF AMERICA

## Abstract

Protein subunit and mRNA vaccines encoding viral glycoprotein antigens fused to the bacteriophage T4 fibritintrimerization domain are increasingly being tested in human clinical trials. AREXVY and ABRYSVO^®^, subunit vaccines composed of respiratory syncytial virus (RSV) prefusion F glycoprotein(s) fused to Foldon, are now FDA-approved and regularly administered. However, administration of vaccine antigens containing the Foldon domain has the potential to elicit off-target antibody responses. Here, we demonstrate that protein subunit vaccine antigens RSV F Foldon and influenza hemagglutinin (HA) Foldon elicit antibodies against the Foldon domain. To dampen Foldon-induced antibody responses, we engineered influenza computationally optimized broadly reactive antigen (COBRA) HA proteins with N-glycosylation motif mutations at three different sites in Foldon. Mass spectrometry analyses confirmed partial occupancy of N-glycosylation modifications at two of the three intended sites. Two of the three COBRA HA Foldon mutant antigens demonstrated reduced binding by anti-Foldon monoclonal and polyclonal antibodies compared to the wild-type COBRA HA Foldon. However, mice immunized with the COBRA HA Foldon mutant antigens did not have a significant reduction in the elicitation of anti-Foldon antibodies. Further analysis suggested that some of the observed differences in antibody binding to mutant Foldon antigens can be attributed to differences in protein sequence alone. Altogether, these data suggest that while the N-glycosylation modifications may partially shield the Foldon from reactive antibodies, this shielding does not significantly reduce its immunogenicity in a naïve setting. Importantly, these data highlight that the Foldon trimerization domain is immunogenic, which should be considered during clinical trials of vaccines that contain Foldon.

## Introduction

The carboxy-terminal region of the bacteriophage T4 fibritin forms a tight β-hairpin domain that stabilizes the oligomeric state of the protein [1]. Since the discovery of this domain via a seminal X-ray crystallographic structure, it has been extensively studied and characterized to elucidate its role within the fibritin protein. These efforts revealed specific properties of the domain that make it a fine-tuned region primed for efficient trimerization, serving to initiate and stabilize the folding of T4 fibritin [1–8]. Consequently, due to its ability to nucleate the folding of homotrimeric proteins, this domain came to be known as a “Foldon” and was quickly adapted for the expression of stabilized recombinant proteins containing trimeric coiled-coil motifs [9].

One of the most ubiquitous uses of the Foldon domain for protein engineering is in the recombinant expression of soluble viral surface proteins for immunological studies and vaccine design. This was first demonstrated with the human immunodeficiency virus type 1 (HIV-1) envelope (Env) protein, where the use of the Foldon yielded Env glycoprotein trimers with enhanced stability relative to Env fused to a GCN4 trimerization domain [10,11]. Since then, Foldon has been used to stabilize the ectodomains of glycoprotein trimers from a wide range of viruses, including influenza hemagglutinin (HA) [12–14], coronavirus spike (S) [15–17], pneumovirus fusion (F) [18–23], flavivirus E [24], henipavirus F [25], and the Lassa virus glycoprotein complex (GPC) [26]. The inclusion of an exogenous trimerization domain can be critical to improving expression yields, retaining immunogenicity to relevant epitopes, and enhancing stability in various storage conditions [13,15,18,22,27]. Compared to other trimerization domains, the Foldon specifically can be preferable in the context of epitopes that are sensitive to conformational disruption [28]. Multiple Foldon-containing viral antigens have been advanced to clinical trials [29–36], with two, DSCav1 [22] and RSVpreF [37], obtaining FDA approval as the vaccine products AREXVY and ABRYSVO^®^.

A concern with the use of exogenous trimerization domains, such as Foldon and the GCN4 trimer, for vaccine design is the potential for off-target immune responses. When trimer-stabilized Env antigen was administered in a rabbit model, both the Foldon and GCN4 domains elicited antibody responses that were detectable by enzyme-linked immunosorbent assay (ELISA) [38]. Similarly, current RSV F subunit vaccine designs elicit anti-Foldon antibodies in mice, non-human primates, and humans [39]. These concerns motivated an attempt to enzymatically remove the Foldon domain from an RSV F antigen post-expression, resulting in the dissociation of RSV F into a monomeric form [40]. Recently, a study demonstrated the engineering of the prefusion RSV F in its trimeric form without fusion to a trimerization domain [39]. Given the potential benefits for antigen stability presented by trimerization domains and the added technical complexities associated with removing them, another strategy to abrogate potential off-target effects is to reduce the inherent immunogenicity of the trimerization domains. This was successfully performed in a rabbit model with Env-GCN4 by strategically introducing glycosylation sites to shield the trimerization domain from antibodies [38]. Whether a similar approach can be applied to the more compact Foldon domain has remained an open question.

We sought to assess the feasibility of immunoshielding the Foldon domain through engineered glycosylation. Using structural analysis, we selected three solvent-exposed sites to incorporate N-linked glycosylation motifs into the Foldon sequence. These were incorporated into an HA-Foldon antigen and expressed in a mammalian expression system. Antigens containing a single glycosylation site in the Foldon expressed and purified as trimers similar to a wildtype HA-Foldon, while an antigen combining two of the sites yielded monomer. The recombinantly expressed and purified trimeric proteins were subjected to glycan analysis, confirming partial occupancy of N-linked glycosylation at two out of the three sites. Testing for cross-reactivity with sera from mice vaccinated with DSCav1 revealed that Foldon-targeting antibodies were elicited in these mice, and that two of the three Foldon mutants had lower reactivity. Testing for binding with a Foldon specific monoclonal antibody, 3E11, failed to delineate between the impact of amino acid substitutions versus the presence of glycan on recognition by this antibody. Sera from mice vaccinated with trimeric HA-Foldon, wildtype and mutants, all showed reactivity with Foldon, with two of the Foldon mutants exhibiting lower levels that were not statistically significant. These data provide important insights into the inherent limitations and challenges to glycoengineering the Foldon domain, presenting key considerations for an expanding landscape of recombinant subunit vaccines stabilized with exogenous trimerization domains.

## Results

### Glycosylation motifs can be incorporated into trimeric Foldon

To evaluate the potential for glycosylation sites to immunoshield the Foldon domain in a vaccine context, we incorporated strategic mutations into plasmids containing a computationally optimized broadly reactive antigen (COBRA) HA, termed Y2, that was appended at the C-terminus by Foldon, an Avi-tag, and a hexahistidine tag (Fig 1A). Based on an analysis of the fibritin crystal structure [1], we selected three candidate sites (“S1”, “S2”, and “S3”) to target for the incorporation of N-linked glycosylation motifs (Fig 1A, 1B). In the context of the expressed protein, these sites are predicted to be solvent exposed and permit the glycans to orient outward without disrupting the tertiary fold (Fig 1C, S1 Fig in S1 File.). Each protein expressed well in CHO cells and could be purified to homogeneity. All the proteins with a single engineered glycosylation site eluted at a similar retention volume to the unmodified “wildtype” protein, indicating no substantial disruption to normal trimer formation in solution (Fig 1D). To see if this could be extended to proteins containing multiple engineered sites in the Foldon, we cloned, expressed, and purified a construct combining the Site 1 and Site 3 engineered glycosylation. While the protein expressed well, size-exclusion chromatography revealed that it eluted at a later retention time compared to a batch of wildtype protein expressed alongside it (S2 Fig in S1 File, S8 Data in S1 File). This indicates probable dissociation of the HA protomers, suggesting that there is a limit to the number of amino acid substitutions that can be made before the trimeric structure of Foldon is impacted.

**Fig 1:**
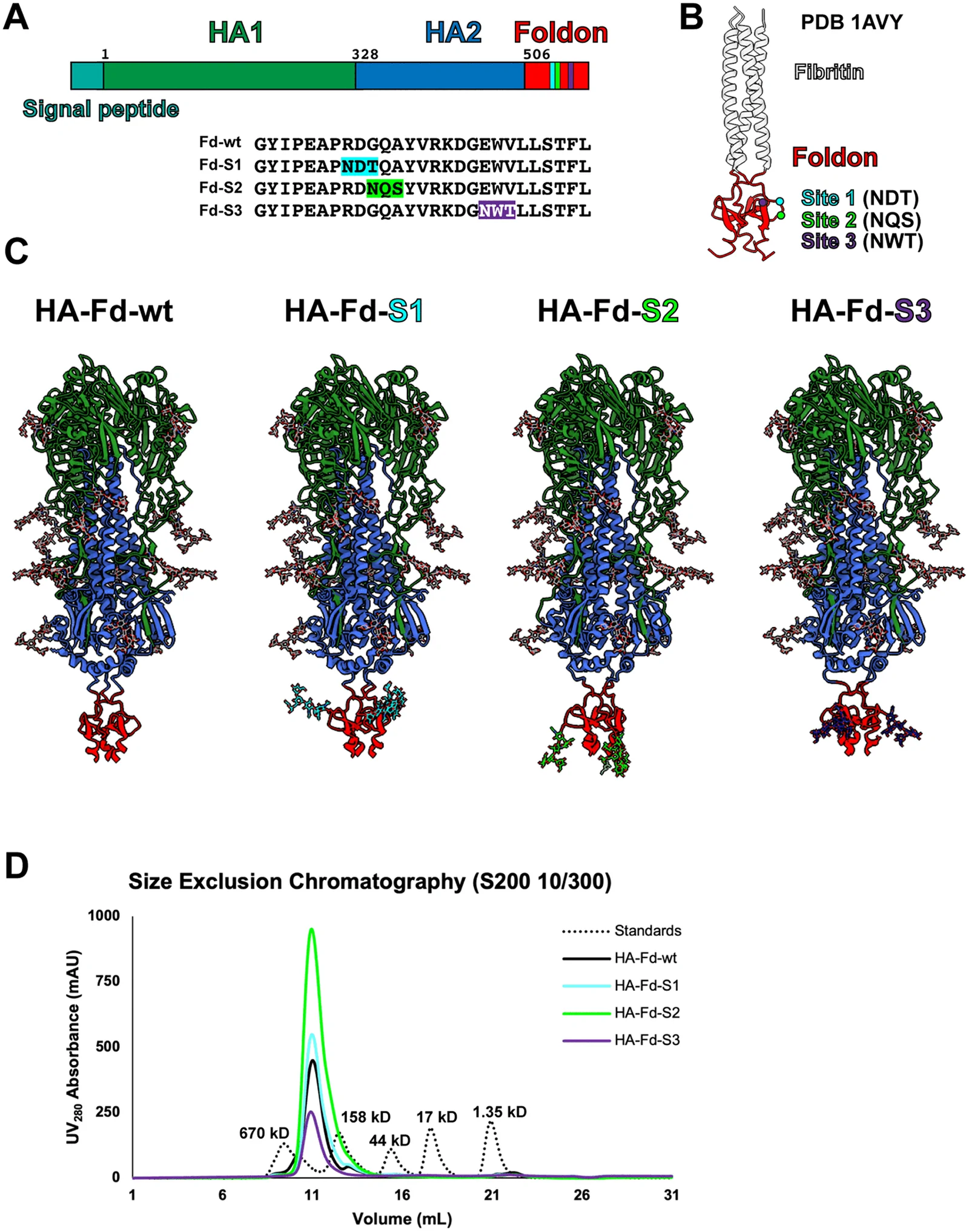
Design, expression, and purification of hemagglutinin Foldon mutants. (A) Construct design of the HA-Foldon mutants. The relative positions of sites 1, 2 and 3 in the protein sequence are marked as cyan, bright green, and purple bars, respectively. The sequences of the different Foldon versions are shown. The C-terminal AviTag and hexahistidine tag are removed for simplicity. (B) Crystal structure (PDB 1AVY) of the fibritin protein used for structure-based protein design. The approximate locations of the mutation sites within the structure are denoted by dots, colored as in (A). (C) Predicted models of the wildtype and mutant protein constructs from AlphaFold3 containing mammalian glycosylation trees, with complex branching excluded for simplicity. Protein regions are colored as in (A). Glycan residues linked to the HA are shown in gray, while those on the Foldon are color-coded based on the mutation site. The residues comprising the AviTags and hexahistidine tags have been hidden for simplicity. (D) SEC traces of each purified protein, expressed from CHO cells, overlayed with protein size standards.

### Engineered glycosylation motifs can lower reactivity to Foldon-reactive sera

In the context of previous exposure to Foldon-containing antigens, it is possible that Foldon-specific antibody responses could be recalled and amplified by subsequent administration of another antigen containing Foldon. Proteins with glycoengineered Foldon have the potential to reduce recognition by these antibodies, thus diminishing the amplification of these off-target responses. To assess whether this might be the case for our HA-Foldon antigens containing single glycosylation sites in the Foldon, we performed ELISAs to test the reactivity with sera from mice vaccinated with the Foldon-containing RSV F antigen PR-DM [41] (Fig 2, S9 Data in S1 File). While HA-Foldon proteins all showed measurable reactivity, the proteins containing the S1 and S3 glycosylation motifs exhibited a statistically significant reduction in binding. This demonstrates that Foldon-specific antibodies are elicited in a mouse model of RSV F vaccination and that Foldon epitopes can be disrupted by the introduction of specific glycosylation motifs.

**Fig 2:**
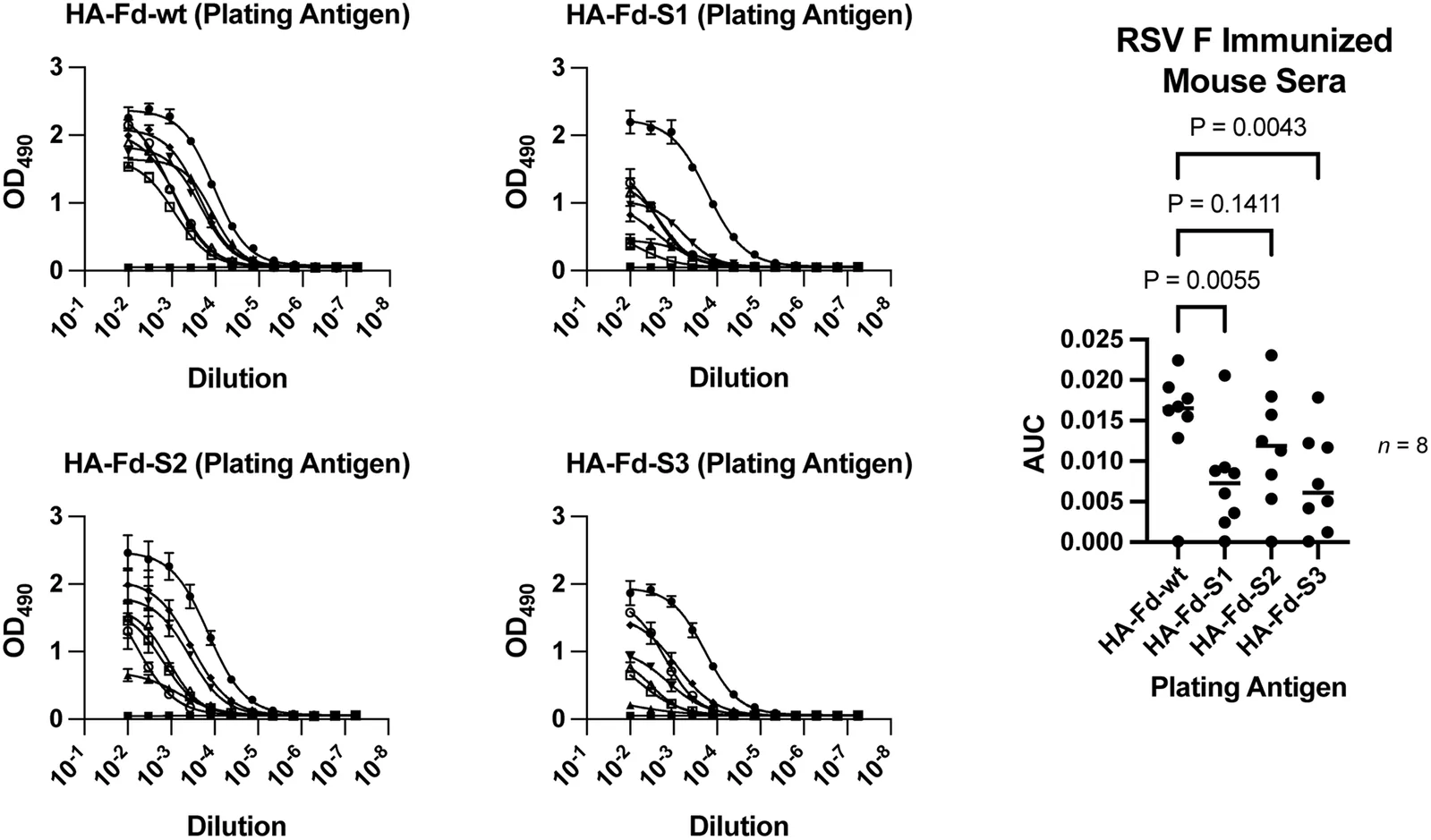
ELISA data with HA-Foldon antigens and RSV immunized mouse sera. Plates were coated with the indicated antigen and tested across the same sera samples. Technical duplicates of eight biological replicates were analyzed in Prism by fitting the data to a nonlinear 4 parameter model, followed by calculation of the area under the curve (AUC). Each data point represents the mean of the technical duplicates, with error bars indicating the standard deviation. A repeated measures one-way ANOVA with Dunnett’s multiple comparisons test was performed to track the changes in each biological replicate from the plate coated with wildtype antigen compared to each plate coated with one of the engineered antigens. One biological replicate showed no response in all of the assays.

### Single glycosylation motifs in Foldon are insufficient to prevent the induction of Foldon-targeting antibodies

While proteins with modified Foldon have reduced antigenicity, the question remained as to whether they would yield lower Foldon-specific responses upon vaccination in naïve subjects. To test this, we vaccinated mice with one of the four HA-Foldon antigens. Following the completion of a prime-boost regimen, serum samples were collected and tested by ELISA for reactivity against recombinant Foldon alone in order to evaluate antibody responses only to the Foldon (Fig 3, S3–S4 Figs in S1 File, S8–S9 Data). None of the groups vaccinated with antigen containing glycoengineered Foldon showed a significant reduction in Foldon-specific antibodies compared to the group vaccinated with antigen containing wildtype Foldon. This suggests that the presence of a single glycosylation site in Foldon is insufficient to shield it from immune recognition.

**Fig 3:**
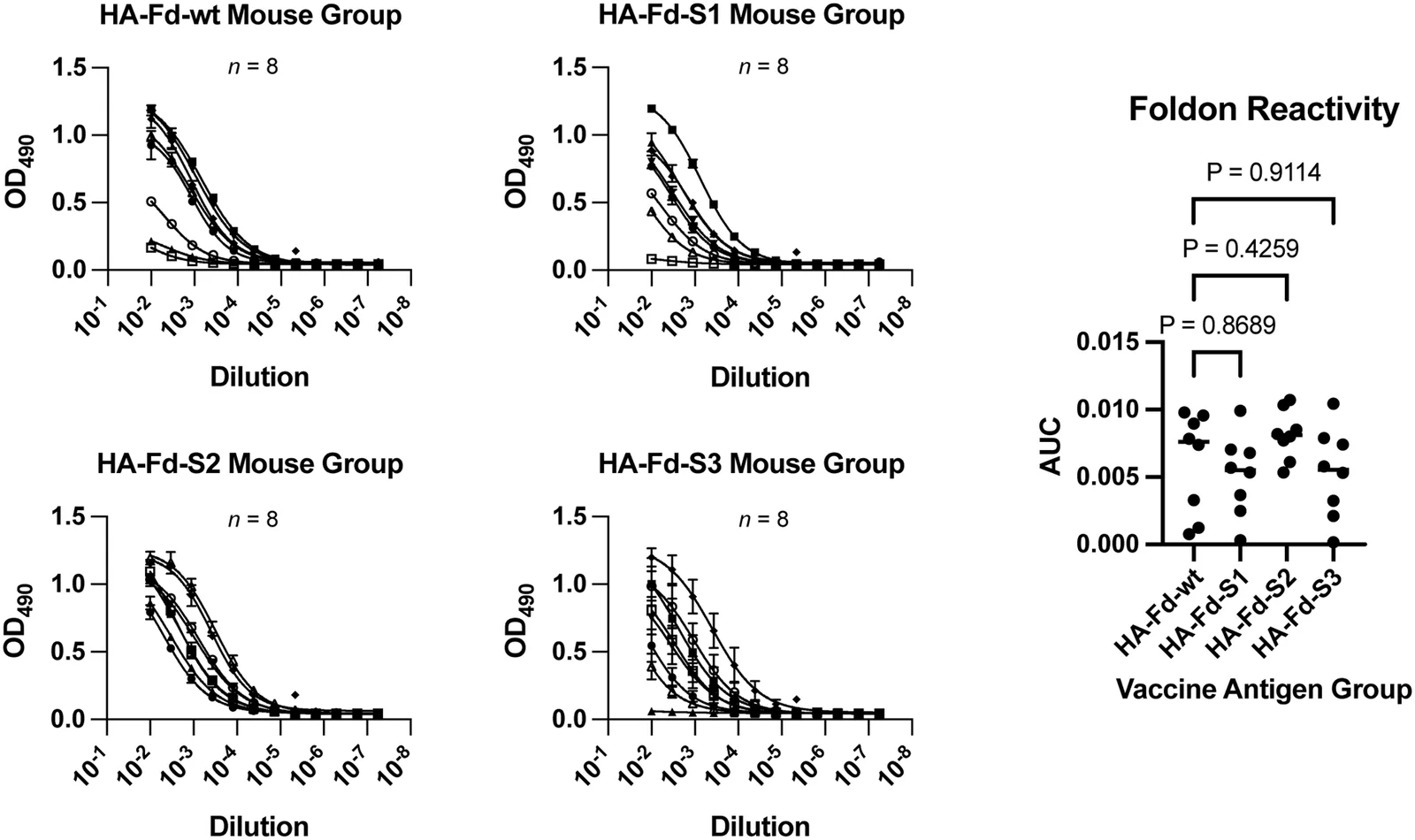
ELISA data with recombinant Foldon and HA-Foldon immunized mouse groups. The plates were coated with Foldon and tested with sera obtained from mouse groups immunized with four different HA-Foldon antigens. The assay with each group was run with technical duplicates with eight biological replicates. Each data point represents the mean of technical duplicates with error bars for the standard deviation. The data were analyzed in Prism by fitting the data to a nonlinear 4 parameter model, followed by calculation of the area under the curve (AUC). An ordinary one-way ANOVA with Dunnett’s multiple comparisons test was performed to assess the differences of each Foldon mutant vaccine group relative to the control group vaccinated with wildtype antigen.

### Diminished binding of anti-Foldon antibodies can be partly accounted for by protein sequence alone

While the ELISA data with the RSV sera showed lower levels of reactivity for S1 and S3 Foldons that were statistically significant, it could not conclude that this was strictly due to glycosylation on the Foldon. An alternative explanation is that the amino acids mutated in the S1 and S3 Foldons form major components of epitopes, and that the difference in amino acids alone is sufficient to cause diminished binding. To delineate between the impact of glycosylation versus protein amino acid changes, we performed PNGase F digestions under native conditions to remove N-linked glycosylations from the four HA-Foldon antigens (Fig 4A). These proteins were then compared to mock digested samples of the same proteins for binding to a known anti-Foldon antibody, 3E11, by biolayer interferometry (BLI) (Fig 4B, S5 Fig in S1 File, S10 Data). The HA stem anchor antibody P1-05 was used as a control to ensure each protein was antigenically intact. Initial analysis of the data revealed that the 3E11 monoclonal antibody recapitulates the general trends of polyclonal sera. Both the S1 and S3 containing proteins show diminished binding by 3E11 relative to the wildtype Foldon (Fig 4B). This is especially pronounced when using the 3E11 in the Fab form, where avidity effects no longer play a role, in contrast to the bivalent mAb. However, comparison of the native and digested proteins revealed no substantial difference in binding between the two. One explanation for this observation is that PNGase F digestion results in the glycosylated asparagine becoming an unglycosylated aspartate, which could also reduce antibody 3E11 binding via its charge [42–45]. Another explanation could be that there is low glycan occupancy in the Foldon sites in general. To investigate this possibility, we utilized mass spectrometry to determine the state of glycosylation at the sites of interest (Fig 4C, S6 Fig in S1 File, S11 Data). This revealed less than 50% occupancy of the glycosylation at the S1 and S2 sites. The S3 sample was more difficult to assess due to partial degradation of the protein, as seen in Fig 4A, but the data was of sufficient quality to identify the protein sequence yet unable to positively identify glycosylation at this site. As a further assessment of the S3 Foldon, we used ELISA to calculate the EC_50_ of 3E11 binding to the wildtype and S3 Foldon proteins expressed in *Escherichia coli* that completely lack glycosylation (Fig 4D, S9 Data). This showed a more than 3-fold reduction in 3E11 binding for the Foldon-S3 protein compared to the wildtype, suggesting that the impact of the S3 mutation on antibody binding may be due to the amino acid changes alone in the absence of glycosylation. Overall, these data suggest that partial glycan occupancy and/or amino acid mutations lower reactivity to anti-Foldon antibodies.

**Fig 4:**
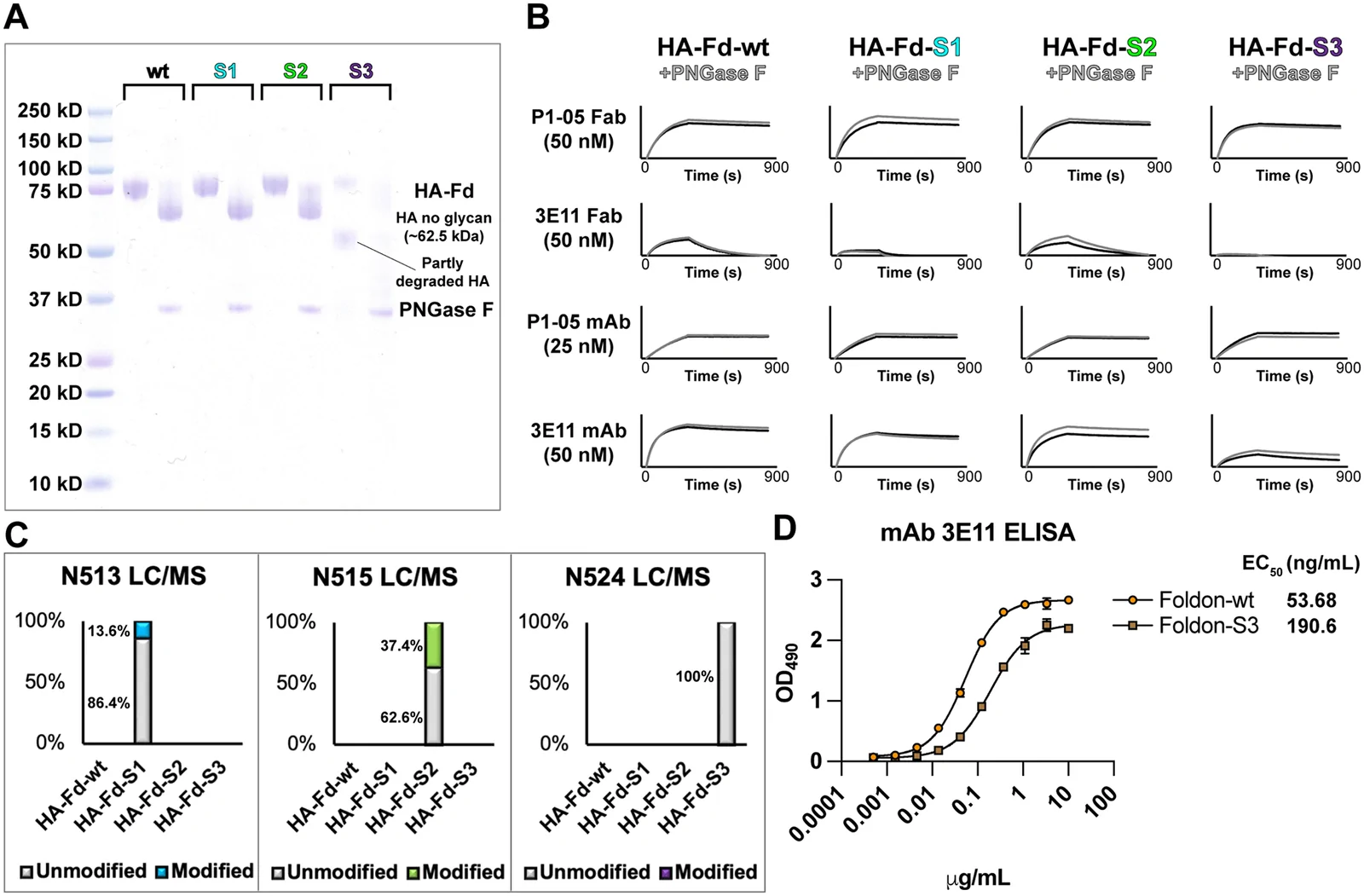
Probing the glycosylation state of Foldon mutants and the impact on antibody binding. (A) SDS-PAGE under reducing conditions of each HA-Foldon protein before and after digestion by PNGase F. (B) Represented graphs of BLI experiments measuring the binding of a stem anchor antibody, P1-05, or Foldon antibody, 3E11, for each HA-Foldon protein with or without PNGase F digestion. (C) Summary graphs of mass spectrometry data of each HA-Foldon protein. Relative percentages of unmodified/modified residues at each residue position of interest are shown as stacked graphs. (D) ELISA probing the binding of the 3E11 monoclonal antibody for the wildtype and Site 3 mutant in bacterially expressed Foldon. Each data point represents the mean of technical duplicates with error bars depicting the standard deviation.

To complement the biochemical analysis, we used AlphaFold3 to generate predicted models of a 3E11 Fab bound to the different versions of Foldon without glycosylation. All produced predictions with similar interfaces with high confidence (Fig 5, S7 Fig in S1 File.). In the prediction with the wildtype Foldon, 3E11 light chain residues R59 and D92 along with heavy chain residue Y102 form major parts of the interface. R59 forms a predicted salt bridge with Foldon residue E524 (numbering in fusion with the HA), D92 a salt bridge with R513, and Y102 a hydrophobic surface that accommodates V526. In the S2 Foldon, all of these residues remain intact, with the mutated residues of the Foldon positioned away from the main interface. Given the position of the residues relative to the antibody, it is possible that binding would remain unhindered even in the presence of glycosylation. In S1, one of the predicted salt bridges is lost with the R513N mutation. With S3, there is loss of the other predicted salt bridge with E524N and decreased hydrophobicity from V526T. While the proximity of the involved residues suggests that glycosylation of S1 and S3 could potentially reduce antibody binding, the predictions without glycosylation also comport well with the experimental data and provide a plausible molecular mechanism that accounts for the impact of amino acid differences with or without glycosylation.

**Fig 5:**
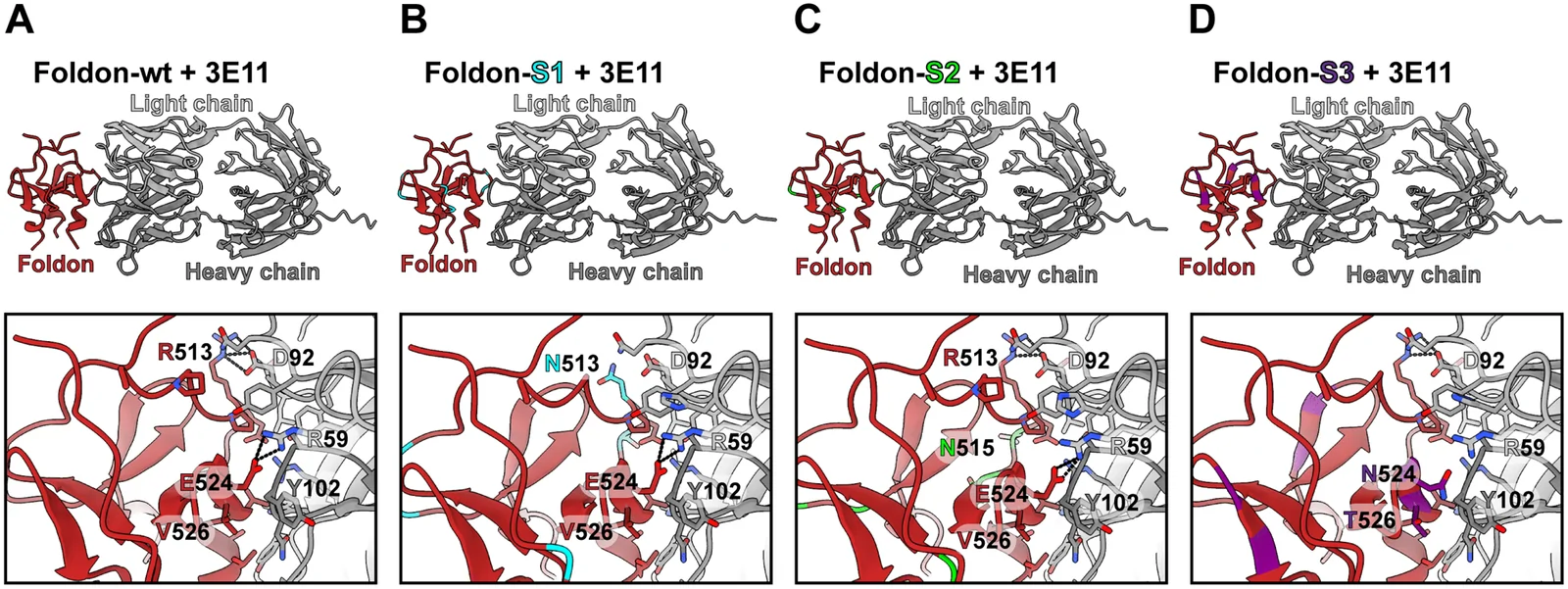
Predicted models of unglycosylated Foldon proteins bound by Fab 3E11. AlphaFold3 predictions of Fab 3E11 bound to (A) wildtype Foldon, (B) Site 1 modified Foldon, (C) Site 2 modified Foldon, and (D) Site 3 modified Foldon. Residues of specific interest are indicated within the closeup regions. Predicted salt bridges with distances less than 4 Å are depicted by black dashes.

## Discussion

Recombinant subunit vaccines necessitate the ability to express, purify, and store proteins in stable formulations. For viral proteins, this often presents a challenge due to the inherent metastable nature of proteins that are the natural target of neutralizing antibody responses. While in some cases this can be overcome by extracting membrane-bound proteins, as in the case of the FluBlok and Novavax vaccines for influenza and SARS-CoV-2, respectively, this may not be a feasible solution for other proteins. This has made the use of exogenous trimerization domains, including GCN4, Foldon, and the more recent “molecular clamp” [46,47], attractive due to their generic ability to stabilize soluble versions of natively trimeric viral proteins. This has culminated in the advancement of several trimerization domain-stabilized recombinant subunit vaccine candidates to Clinical Trials, including the FDA approved AREXVY and ABRYSVO^®^ RSV vaccines.

Despite their utility, however, concerns have lingered over whether these trimerization domains could be the target of undesired immune responses. These concerns have grown as data from clinical trials have demonstrated antibody responses in human cohorts. The first-generation “molecular clamp” was not advanced beyond Phase I studies due to the presence of HIV gp41 sequence motifs that elicited antibody responses that interfered with HIV testing [47]. More recently, RSV vaccine formulations containing Foldon under use in a clinical trial showed measurable anti-Foldon responses that were amplified after boost [39]. These specific cases highlight the risk that as more subunit vaccines advance to human patients, unintended immune responses towards trimerization domains could be elicited.

As a precautionary measure, different approaches have been explored to reduce the impact of trimerization domains on immune responses induced by recombinant subunit vaccines. This includes designing stabilized antigens that dispense with the need for a trimerization domain altogether, such as what has been attempted for RSV F and HIV gp120 [39,48–52]. While ideal from the standpoint of eliminating the potential for off-target responses, a potential drawback is increased developmental lead time with the need to extensively customize designs on a case-by-case basis. An alternative approach that seeks to retain the general utility of trimerization domains while limiting the immunological impact is to modify them in a way that diminishes their immunogenicity. Such an approach demonstrated a measure of success with the second generation “molecular clamp” [53]. While not being able to eliminate the immunogenicity entirely, a combined approach of sequence modification with the introduction of glycosylation was able to lessen the immunogenicity of the trimerization domain and abrogate the cross-reactivity with HIV tests. Similarly, the GCN4 trimerization domain was shielded by strategically introducing glycosylation sites [38]. In a similar manner, we sought to assess whether glycosylation-mediated immunoshielding of the Foldon domain was feasible.

Based on the structure of Foldon, three sites were chosen that had the potential to display glycosylation towards the solvent. When fused to HA gene constructs, all three successfully yielded trimeric protein. However, attempts to combine mutations in a single construct resulted in the dissociation of HA protomers. This indicates a probable limitation to the mutability of the Foldon domain before it loses structural integrity. For the single site mutants, mass spectrometry was able to confirm partial occupancy of the Site 1 and Site 2 Foldon mutants. No glycosylation could be confirmed for Site 3, though the analysis was complicated by partial degradation of the protein. This propensity towards degradation may indicate a loss in stability of the Site 3 Foldon. Although it retains a trimeric structure, the mutation of valine to threonine near the C-terminus may increase structural flexibility and make it more susceptible to proteolysis.

When assessing the impact of these glycosylation sites on antibody recognition, we were unable to positively establish that the presence of glycosylation mediated a decrease in immune recognition. This could partly stem from incomplete occupancy of the glycosylation sites, which would allow some epitopes to remain exposed. Alternatively, a single glycan may be insufficient to block all Foldon epitopes, the number of which is unknown. Where there is a measurable reduction in anti-Foldon antibody binding for the Site 1 and Site 3 mutants, some of the effect can be directly attributed to changes in amino acids that form part of the epitope of some antibodies like 3E11. Taken together, while glycans and/or the underlying amino acid changes can shift the antigenicity, they were unable to significantly reduce the immunogenicity of the Foldon in a naïve model. Although not statistically significant, we note that there were trends in reducing immunogenicity of the Foldon by the Site 1 and 3 mutants and repeating this study with a larger number of mice may show significant differences. In addition, considering the statistically-significant reduction in antigenicity to Foldon by the Site 1 and Site 3 mutants, future studies could evaluate whether these mutants reduce anti-Foldon recall responses in a pre-immune model.

In conclusion, while glycosylation motifs can be introduced into the Foldon domain, there are limits to the number of point mutations that can be introduced before it loses its structural integrity. Single site mutations are also insufficient to shield the Foldon from immune recognition. Due to these constraints, alternative strategies that preserve the structural core of the Foldon are probably necessary to successfully immunoshield the Foldon.

## Materials and methods

### Ethics statement

Mouse studies were performed in accordance with the recommendation in the Guide for the Care and Use of Laboratory Animals of the NIH. The protocol for the mouse vaccine studies with RSV F PR-DM was approved by the University of Georgia IACUC under protocol number A2022 04–023-Y2-A1. The protocol for the mouse vaccine studies with HA-Fd was approved by the University of Georgia IACUC under protocol number A2024 06–001-Y1-A1.

### HA plasmid cloning and preparation

COBRA Y2 in pcDNA3.1 was synthesized [54] and cloned as previously described [55]. The Foldon mutants were generated by Phusion site-directed mutagenesis (Site 1) or by inserting a Gene Block (IDT) by Gibson Assembly (Sites 2 and 3) (Fig 1). The HA-Fd constructs were subsequently transferred into a plasmid derivative of pcDNA3.1 optimized for high protein expression [56] by restriction enzyme cloning. Following Sanger sequence verification, the plasmids were transformed into *Escherichia coli* DH5α cells. Large scale plasmid preps were performed using the Plasmid Maxi Kit (QIAGEN) or PureLink™ Expi Endotoxin-Free Maxi Plasmid Purification Kit (Invitrogen) according to the manufacturer’s instructions.

### Recombinant protein expression in Chinese Hamster Ovary cells

All recombinant proteins were produced in suspension-adapted Chinese Hamster ovary cells (CHO-S) obtained from Thermo Fisher (Thermo Fisher, Life Technologies, Carlsbad, CA, US) were propagated at 37 °C, 8% CO_2_, 85% humidity, agitating at 135 rpm using a Kuhner ISF1-X shaker incubator (Kuhner, Birsfelden, Switzerland). Standard growth media comprised CD-CHO medium supplemented with 8 mM GlutaMax, 10 mM hypoxanthine and 1.6 mM thymidine. All media and additives were purchased from Thermo Fisher (Carlsbad, CA) unless stated otherwise. For protein expression, cells were transiently transfected by electroporation using the MaxCyte STX (MaxCyte, Gaithersburg, MD, US) according to the manufacturer’s instructions. Briefly, cells were resuspended in electroporation buffer (MaxCyte, Gaithersburg, MD, US) at a density of 2 × 10^8^ cells/mL with 300 µg/mL of expression plasmid, using a 1:1 (w/w) light to heavy chain ratio for antibody expression, and electroporated in OC-400 processing assemblies (MaxCyte, Gaithersburg, MD, US). For preparative-scale transient transfection experiments, 3.2 × 10^8^ CHO cells were transfected and seeded at 5 × 10^6^ /mL in OptiCHO medium supplemented with 0.1% pluronic acid, 2 mM GlutaMax, 10 mM and 1.6 mM thymidine, maintained at 37 °C, 8% CO_2_, 85% humidity with 135 rpm agitation. 24 h after transfection, the cultures were supplemented with 1 mM Sodium Butyrate, and the incubation temperature lowered to 32 °C. Daily feeding with 0.5% (v/v) Yeastolate (BD, Franklin Lakes, NJ, US), 2.5% (v/v) CHO-CD Efficient Feed A, 0.25 mM GlutaMax and 2 g/L Glucose (Sigma-Aldrich St. Louis, MO) is required to maintain the cells at ~1 × 10^7^ cells/mL for protein expression. Cultures were harvested when the viable cell density dropped below 50%, usually 8–10 days after transfection. Conditioned media was then harvested by pelleting the cells in the presence of cOmplete inhibitor cocktail (Sigma-Aldrich St Louis MO) at 250xg for 30 min, followed by prefiltration through Nalgene Glass Prefilters (Thermo Scientific, Waltham, MA, US) and filtration through 0.2-μm PES filters (Nalgene, Thermo Scientific, Waltham, MA, US). Conditioned media was immediately processed for purification or stored briefly (< 2 days) at 4 °C.

### Purification of recombinantly expressed HA

Harvested supernatants of the Y2.CHO.wt, Y2.CHO.S1, Y2.CHO.S2, and Y2.CHO.S3 were diluted with an equal volume of Buffer A (500 mM NaCl, 20 mM sodium phosphate, 20 mM imidazole [pH 7.4]) and subsequently filtered through a 0.2 µm PES membrane. The buffer adjusted samples were then loaded onto Ni-NTA columns (Cytiva) equilibrated with Buffer A. The loaded columns were washed with 5 column volumes (CV) of Buffer A, followed by a 20 CV gradient elution to Buffer B (500 mM NaCl, 20 mM sodium phosphate, 500 mM imidazole [pH 7.4]). Fractions were pooled based on the chromatograms and SDS-PAGE analysis, concentrated, and run on a Superdex 200 10/300 GL column (Cytiva) equilibrated with Phosphate Buffered Saline (PBS; Sigma Aldrich). Sample purity was assessed by SDS-PAGE and the purest fractions pooled and concentrated prior to storage at 4 °C or flash freezing and storage at −80 °C. Samples of the wildtype and three single-site Foldon glycan mutants were submitted to the Mass Spectrometry Laboratory for Protein Sequencing at the Lerner Research Institute (Cleveland Clinic Foundation) for assessment of the glycosylation state in the Foldon region.

Harvested supernatants of Y2.CHO.wt and Y2.CHO.S1 + S3 were filtered through 0.2 µm PES membrane and loaded directly onto HisTrap excel columns (Cytiva) equilibrated in Buffer C (500 mM NaCl, 20 mM sodium phosphate [pH 7.5]). The columns were subsequently washed/eluted with a 20 CV gradient to Buffer B. The fractions were pooled based on the chromatogram and SDS-PAGE analysis and further purified using a Superdex 200 10/300 GL column equilibrated with PBS. The major peak was assessed by SDS-PAGE and the purest fractions pooled prior to sample concentration and storage.

### Expression and purification of recombinant mAb 3E11

The variable regions of antibody 3E11 [57] were synthesized and cloned into human IgG1 and kappa framework plasmids for the heavy and light chain genes, respectively (Twist Biosciences). Recombinant mAb 3E11 was purified from serum-free CHO-S culture supernatant ten days after transfection by electroporation with light and heavy chain expression plasmids using a HiTrap Protein G column (Cytiva, Marlborough, MA). 60 mL of 0.2 µm filtered supernatant was diluted 1:1 with 20 mM sodium phosphate pH 7.2 binding buffer and applied to a pre-equilibrated column at a flow rate of 2 mL per minute. The column was washed with 10 CV of binding buffer, and antibody eluted with 5 CV 0.1 M glycine-HCl, pH 2.7 elution buffer, collecting 1 mL fractions into 200 µL 1 M Tris-HCl, pH 9.0 neutralizing buffer. Fractions were pooled based on 280 nm absorbance and SDS-PAGE analysis and dialyzed into PBS pH 7.2, before aliquoting and snap freezing in liquid nitrogen for long term storage at −80 °C.

### Expression and purification of Foldon from insect cells

COBRA HA proteins Y2 and X6 fused to a proteolytically removable Foldon with a hexahistidine tag were expressed and purified from Sf9 cells using the flashBAC baculovirus system as previously described [58–60] The proteins were digested by thrombin, dialyzed overnight in PBS, and separated by size-exclusion chromatography on a Superdex 100 10/300 GL column (Cytiva). Fractions from multiple SEC runs containing the His-tagged Foldon were pooled, concentrated, and snap frozen using liquid nitrogen, and stored at −80 °C until further use.

### Expression and purification of Foldon from bacterial cells

The Foldon domains for the wildtype and three single-site glycan mutants were cloned by Gibson Assembly into vector pET52b in frame with an N-terminal StrepTag II and HRV 3C Protease Cleavage Site. Sequence confirmed plasmids were transformed into T7 Express Escherichia coli cells (NEB) by heat shock. A single colony from each was used to inoculate Luria-Bertani (LB) medium supplemented with 100 µg/mL of ampicillin and incubated with shaking at 37 °C overnight. The following day, the starter cultures were added to fresh LB supplemented with ampicillin at a ratio of 10–15 mL per L of medium. The cultures were incubated with shaking until the optical density at 600 nm (OD600) reached 0.6–0.8, then Isopropyl ß-D-1-thiogalactopyranoside (IPTG) was added at a final concentration of 0.8 or 0.4 mM. The culture was continued either for 4–5 h at 37 °C (0.8 mM IPTG) or overnight at 18 °C (0.4 mM IPTG). The cells were pelleted by centrifugation at 5000xg for 10 min. The recovered pellets were processed immediately for protein purification or frozen and stored at −80 °C.

The pellets were resuspended in Buffer D (150 mM NaCl, 100 mM Tris [pH 7.5], 1 mM EDTA) at a ratio of approximately 30 mL of buffer per L of culture prior to cell pelleting. Cell lysis was performed by sonication on ice with a 0.5” probe at 50% amplitude, 5 sec on, 5 sec off, with a total cycle time of 6 min. The lysates were clarified by centrifugation at 40,000xg for 30 min, followed by filtration through 0.2 µm Acrodisc syringe filters (PALL). The samples were poured over Streptactin (IBA Life Sciences) or Streptactin XT superflow resin (BioRad) equilibrated with Buffer D. The resin was washed with 10 CV of Buffer D, then protein was eluted with 5 or 10 CV of Buffer E (150 mM NaCl, 100 mM Tris [pH 7.5], 1 mM EDTA, 5 mM d-desthiobiotin). The wildtype, S1, and S3 Foldon constructs all yielded detectable protein, while the S2 Foldon failed to express solubly. The elutions for the wildtype, S1, and S3 were subsequently concentrated and further purified by size exclusion chromatography using a Superdex 75 10/300 column equilibrated in PBS. Fractions containing Foldon based on SDS-PAGE analysis were pooled, concentrated, then stored at 4 °C until use.

### Mouse vaccine studies with recombinant RSV F PR-DM

Eight BALB/c mice (6–8 weeks old; The Jackson Laboratory) were immunized in a prime-boost regimen with 25 μL purified RSV A2 PR-DM F (trimer), (5 μg protein/mouse) + 25 μL AddaS03™ adjuvant *via* the subcutaneous route into the loose skin over the neck, while mice in control groups were immunized with PBS + AddaS03™ adjuvant (8 animals per group). BALB/c mice were used as the standard model for RSV studies [61]. AddaS03™ is a preclinical adjuvant compositionally similar to AS03® and improves humoral responses in BALB/c mice [62]. Three weeks after prime, the mice were boosted with the same amount of the antigen + adjuvant. Three weeks after the boost, mice were bled. All procedures were done on non-anesthetized mice. At the end of the study, mice were euthanized via 2,2,2-tribromoethanol (Avertin), followed by a secondary method of cervical dislocation. No animals experienced any adverse events due to the vaccination or bleeding procedures.

### Mouse vaccine studies with recombinant HA-Fd

DBA2/J mice (6–8 weeks old; The Jackson Laboratory) were vaccinated with 1 of 4 vaccine formulations consisting of the Y2 COBRA antigen or one of the glycosylation mutants, with 8 mice per group. DBA/2J mice were used as a standard strain showing high susceptibility to influenza infection [63]. The vaccine formulations consisted of CHO produced soluble proteins were adjuvanted with AddaVax™, a squalene-based oil-in-water nano-emulsion commonly used in preclinical studies that is similar to MF59® in some licensed influenza vaccines [64,65]. A Mock group was also included in the study consisting of mice vaccinated with PBS + AddaVax™ as a naïve comparator. Mice were vaccinated in a prime-boost regimen at days 0 and 28 with blood collections at days 14 and 42 to assess the vaccine-mediated serologic response. Euthanasia was performed via carbon dioxide and secondarily cervical dislocation when humane endpoints are reached or serious complications experienced outside of the usual progression of the disease. Mice were anesthetized via inhaled isoflurane at a concentration of 2–5%. Depth of anesthesia was monitored by toe pinch and movement of mice. After procedure, mice were monitored until alert and upright. Animals received supportive care when it did not interfere with the experiment being performed or euthanized when humane endpoints were reached or serious complications were experienced outside of the usual progression of the disease to prevent suffering.

### Enzyme-linked immunosorbent assays

Costar High Binding polystyrene plates were coated at 4 °C overnight with 50 µL of antigen diluted antigens (HA-Foldon or Foldon alone) diluted to 1 µg/mL in PBS. The plates were washed one time with PBS supplemented with 0.05% Tween 20 (PBS-T) with a Tecan 96W plate washer. Blocking was performed at room temperature for at least 1 h with Blocking Buffer (PBS-T supplemented with 3% milk), 200 µl per well. The Blocking Buffer was aspirated and the experimental samples added. Samples were prepared in Dilution Buffer (PBS-T supplemented in 1% milk). For sera, 3-fold dilution series were utilized beginning at 1:100. For monoclonal antibody 3E11, a 3-fold dilution series was utilized beginning at a concentration of 10 µg/mL of antibody. The samples were added, 100 µL per well, and incubated at room temperature for 2 h. The plates were washed three times with PBS-T, then 100 µL of the secondary antibody, diluted 1:5000 in Dilution Buffer, was added to each well and incubated at room temperature for 1 h. For mouse sera, a goat anti-mouse IgG antibody conjugated to horse-radish peroxidase was used (Jackson ImmunoResearch, catalog number 115-035-071). For 3E11, a goat anti-human IgG antibody conjugated to horse-radish peroxidase was used (Thermo Fisher, catalog number A18817). The plates were washed three times with PBS-T and tapped dry. Substrate solutions were prepared by dissolving 5 mg OPD tablets (o-phenylenediamine dihydrochloride; Thermo Scientific) in 12.5 mL of phosphate citrate buffer (Sigma Aldrich) and 6.25 µL of 30% hydrogen peroxide per ELISA plate. The substrate solution was added, 100 µL per well, and incubated at room temperature. After 10 min, the reaction was quenched by adding 50 µL of 3 M HCl per well. The absorbance at 490 nm was measured with a SpectraMax® Plus384 plate reader and SoftMax® Pro software v4.8. All assays were performed in duplicate.

Statistical analyses were performed in Prism v10.3.0 (GraphPad). The data was fit with a nonlinear 4 parameter model as a function of sample dilution. For sera, the area under the curve (AUC) was calculated for each sample dilution series. For mice vaccinated with different antigens, an ordinary one-way ANOVA with Dunnett’s multiple comparisons test was performed to assess the differences of each Foldon mutant vaccine group relative to the control group vaccinated with wildtype antigen. For the sera obtained from mice vaccinated with RSV F, a repeated measures one-way ANOVA with Dunnett’s multiple comparisons test was performed to assess the differences in reactivity observed for each serum sample on each Foldon mutant antigen plate relative to the wildtype antigen plate. Differences were considered statistically significant if P < 0.05. For assays with 3E11, IC50 values were calculated based on the curve fit. All data and analyses are included in the supporting information (S9 data).

### Removal of N-linked glycans by PNGase F digestion

N-linked glycan removal from recombinant HA under native conditions was performed with PNGase F based the manufacturer’s protocol (New England Biolabs, product P0704). Briefly, approximately 20 µg of protein was diluted in PBS to a volume of 18 µL, followed by the addition of 2 µL of 10X GlycoBuffer 2 (500 mM sodium phosphate, pH 7.5). Subsequently, 5 µL of PBS (mock digestion) or PNGase F enzyme were added and the solution gently mixed. Digestion was performed at 37°C for 24 h. The mock and PNGase F digested samples were assessed by SDS-PAGE on a NuPAGE™ 4–12% gel (Invitrogen).

### Biolayer interferometry (BLI) with Fabs P1-05 and 3E11

Recombinantly expressed P1-05 and the Fab derivative were repurposed from a previous study [66]. Fab 3E11 was generated from recombinantly expressed mAb using the Pierce Fab Preparation kit (Thermo) according to the manufacturer’s instructions. All BLI assays were performed on an Octet Red384 system with solutions prepared in Kinetics Buffer (PBS, 0.1% Bovine Serum Albumin, 0.02% Tween 20). Experiments were conducted with black costar 96-well plates with shaking at 1000 rpm and a constant temperature of 30°C. HIS2 biosensors were hydrated for at least 10 min in Kinetics Buffer prior to running the assay. After an initial 120 s baseline, recombinant HA at a concentration of ~2 µg/mL from the mock or PNGase F digested samples were loaded until the signal reached a wavelength shift of ~1 nm. Following another baseline step for 120 s, the loaded biosensors were dipped into P1-05 Fab (50 nM), 3E11 Fab (50 nM), P1-05 mAb (25 nM), or 3E11 mAb (50 nM) for 300 s. The biosensors were then returned to the wells used for the second baseline to measure dissociation for 600 s. Data processing was performed in the Octet Data Analysis HT software v7 (Sartorius) to remove high frequency noise by Savitzky-Golay filtering, normalize the data, and account for interstep discrepancies. The curves were analyzed visually to qualitatively compare the relative binding of each antigen to the different antibodies. Each experiment was performed twice. The raw and processed data are included in the supporting information (S10 Data).

### Generation of computational models

Predicted models were generated with AlphaFold3 using a different random seed for each prediction (https://alphafoldserver.com/) [67]. The HA models were predicted with the glycan string NAG(FUC)(NAG(BMA(MAN)(MAN))) linked to each predicted N-linked asparagine residue. All output models were assessed based on the pTM and iPTM scores and the best scoring models (or model “0” when scores were equivalent) were chosen for depiction in the main text. The models and associated predicted alignment error (PAE) plots were visualized using ChimeraX [68–70].

## Supporting information

S1 File Supporting figures S1-S7. S1 Fig. Related to Fig 1C. Expanded details on HA-Foldon predicted models generated by AlphaFold3. S2 Fig. Combining Foldon mutants destabilizes the trimeric assembly of hemagglutinin. S3 Fig. Purification of Foldon proteins used in the ELISA experiments. S4 Fig. Related to Fig 3. Expanded ELISA data with recombinant Foldon and HA-Fd immunized mouse groups. S5 Fig. Related to Fig 4. Expanded data for assessment of Foldon glycosylation and the impact on antibody binding. S6 Fig. Related to Fig 4. Summary data from mass spectrometry. S7 Fig. Related to Fig 5. Expanded details on AlphaFold3 predicted models of unglycosylated Foldon proteins bound to Fab 3E11.
(PDF)

S8 Data Size Exclusion Chromatography source data. (ZIP)

S9 Data Enzyme-linked immunosorbent assay source data. (ZIP)

S10 Data Biolayer interferometry source data. (ZIP)

S11 Data Mass spectrometry reports and source data. (ZIP)

S12 Data Raw gel images. (PDF)
