# Supplementary material for: Immunogenicity of the Foldon trimerization domain and efforts to shield it
Source: PLoS One. 2026 Nov 13;21(11):e0362571. doi: 10.1371/journal.pone.0362571 (PMC13766278; doi:10.1371/journal.pone.0362571)
Supplement: Supplementary file 1 [file pone.0362571.s001.pdf]

Immunogenicity of the Foldon trimerization domain and efforts to shield it

John V. Dzimianski[1], Spencer R. Pierce[2,3,4], Sara M. O'Rourke[1], Ian Kunkel[1], Kevin Delgado-Cunningham[1], Aaron D. Gingerich[2], Jarrod J. Mousa[2,3,5,6], Ted M. Ross[2,3,4], Rebecca M. DuBois[1]*

[1]Department of Biomolecular Engineering, University of California Sant Cruz

[2]Center for Vaccines and Immunology, College of Veterinary Medicine, University of Georgia

[3]Department of Infectious Diseases, College of Veterinary Medicine, University of Georgia

[4]Florida Research and Innovation Center, Cleveland Clinic

[5]Department of Biochemistry and Molecular Biology, Franklin College of Arts and Sciences, University of Georgia

[6]Department of Biomedical Sciences, College of Medicine, Florida State University

* Corresponding author

E-mail: rmdubois@ucsc.edu

Supplemental Figures

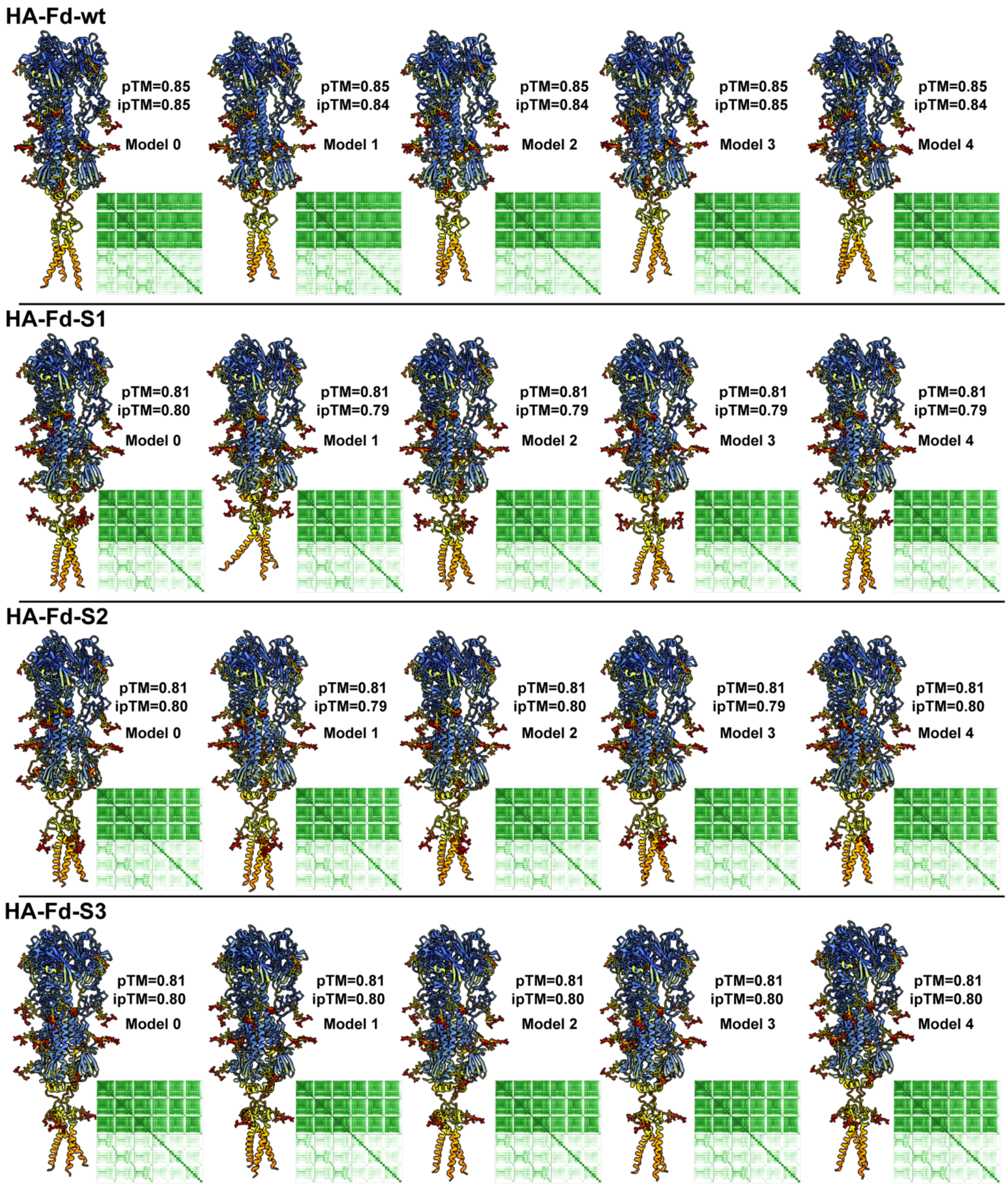

S1 Fig. Related to Fig 1C. Expanded details on HA-Foldon predicted models generated by AlphaFold3. The five predicted models are shown for each protein construct with residues colored based on the predicted Local Distance Difference Test (pLDDT) values. Predicted alignment error (PAE) plots are depicted as green matrices. The predicted template modeling (pTM) and interface predicted template modeling scores for each model are shown.

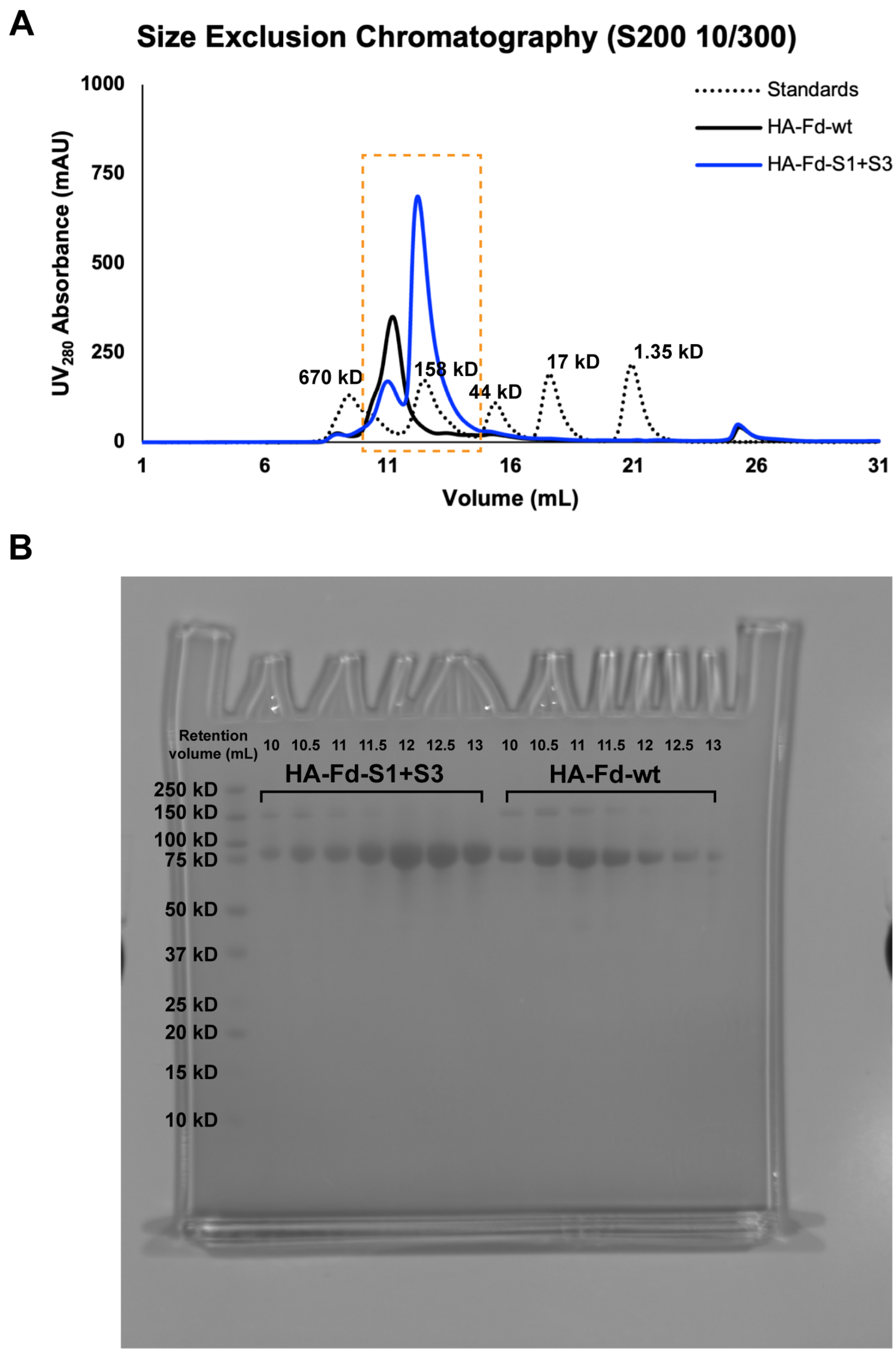

S2 Fig. Combining Foldon mutants destabilizes the trimeric assembly of hemagglutinin. (A) SEC traces of the wildtype and double site mutant HA-Foldon proteins overlayed with protein size standards. The dashed orange box indicates the range of fractions sampled for SDS-PAGE analysis. (B) SDS-PAGE under reducing conditions of the fractions sampled in (A).

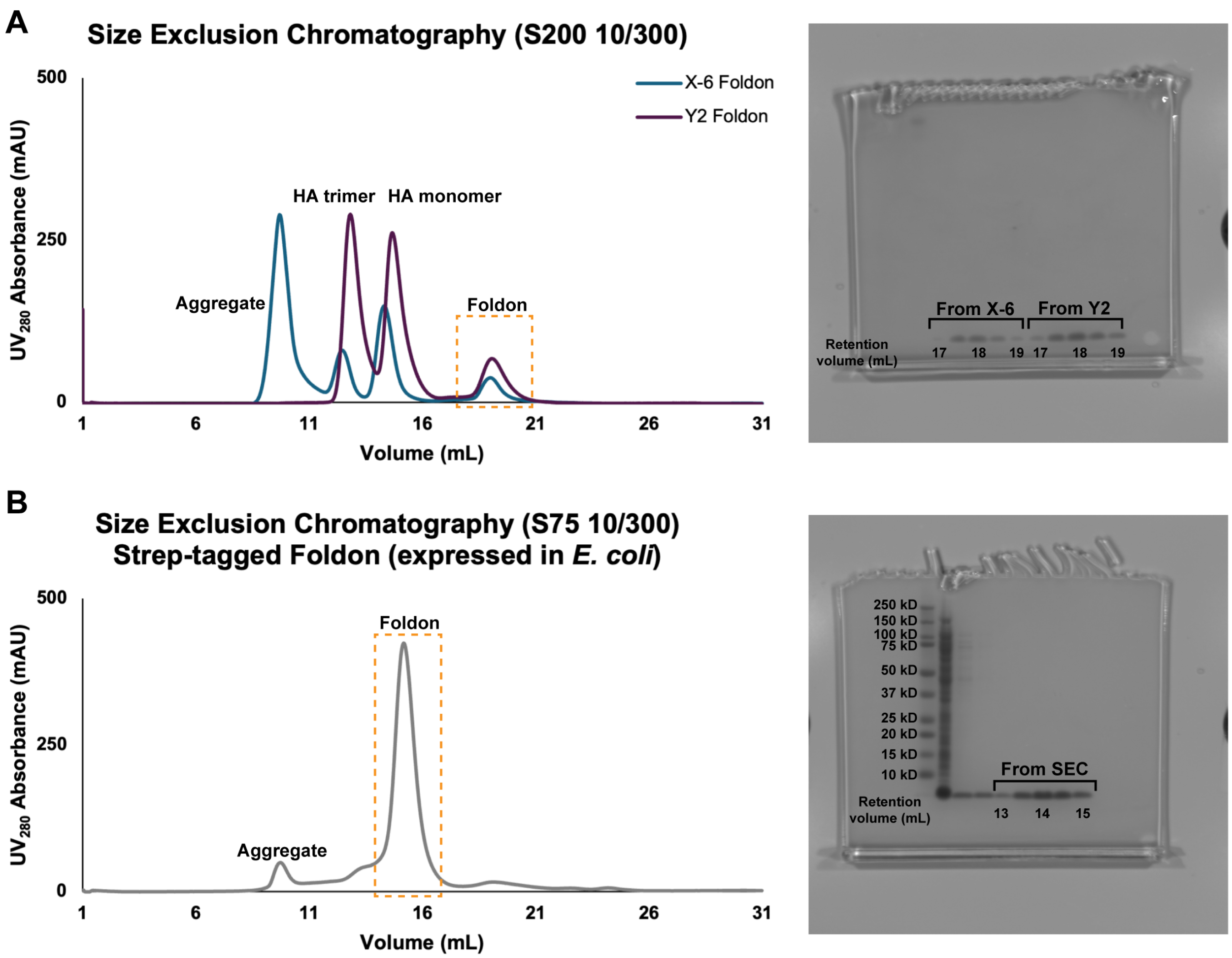

S3 Fig. Purification of Foldon proteins used in the ELISA experiments. (A) SEC traces and SDS-PAGE under reducing conditions of HA proteins digested by thrombin to liberate the Foldon domain (containing both hexahistidine and StrepTag II affinity tags). (B) SEC trace and SDS-PAGE under reducing conditions of Strep-tagged Foldon expressed in *Escherichia coli*.

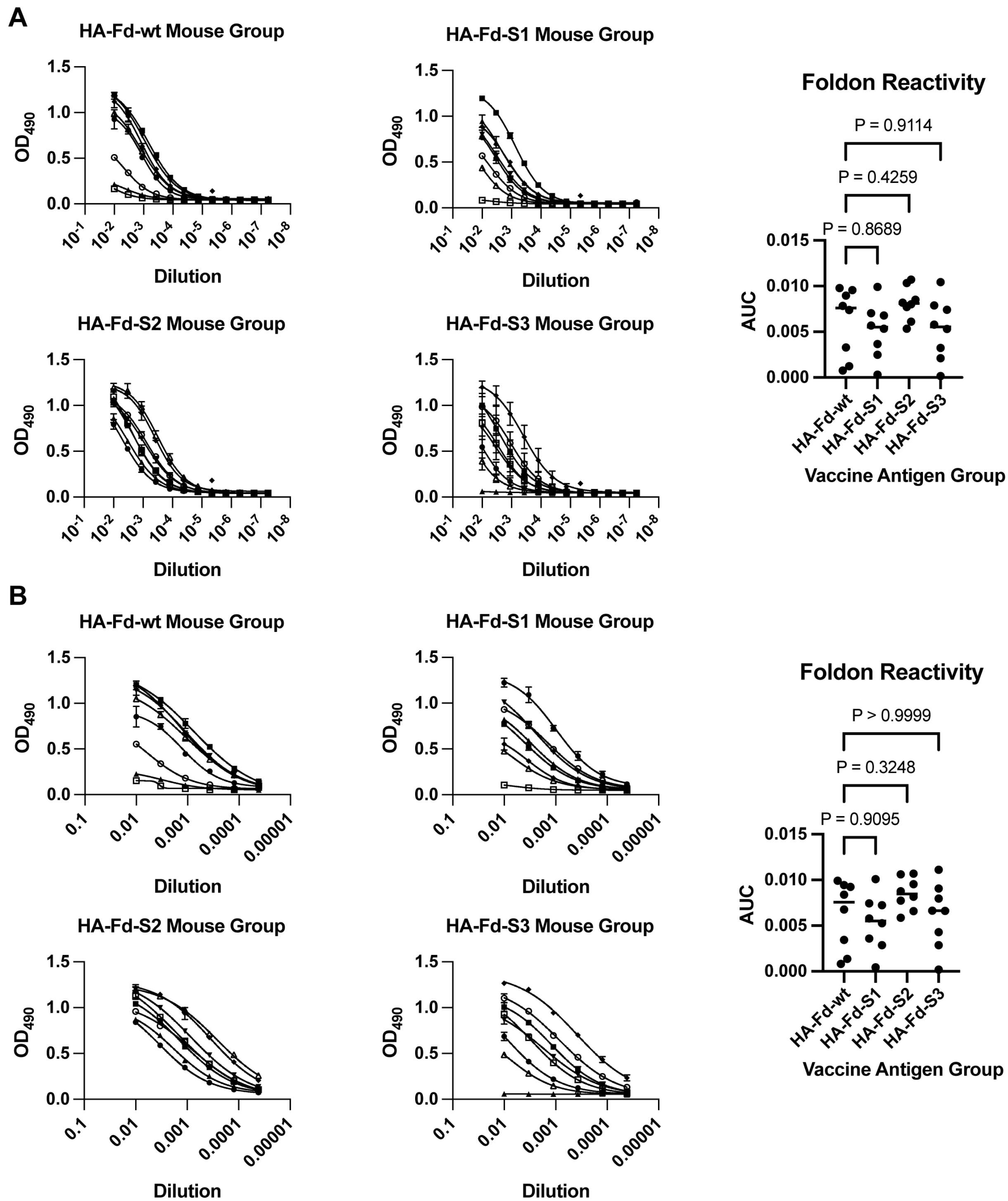

S4 Fig. Related to Fig 3. Expanded ELISA data with recombinant Foldon and HA-Fd immunized mouse groups. The plates were coated with (A) His-tagged Foldon from insect cell expressions or (B) Strep-tagged Foldon derived from bacterial expressions. Each data point represents the mean of two technical replicates with error bars depicting the standard deviation. Eight biological replicates were used in each experimental group.

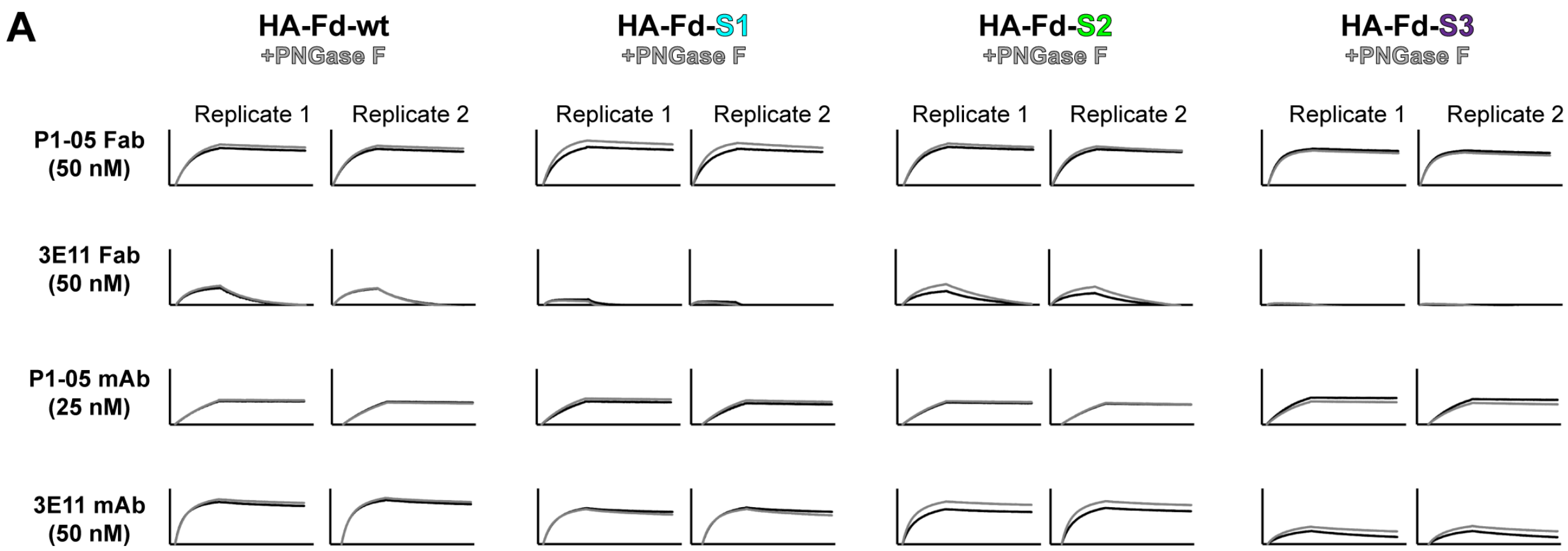

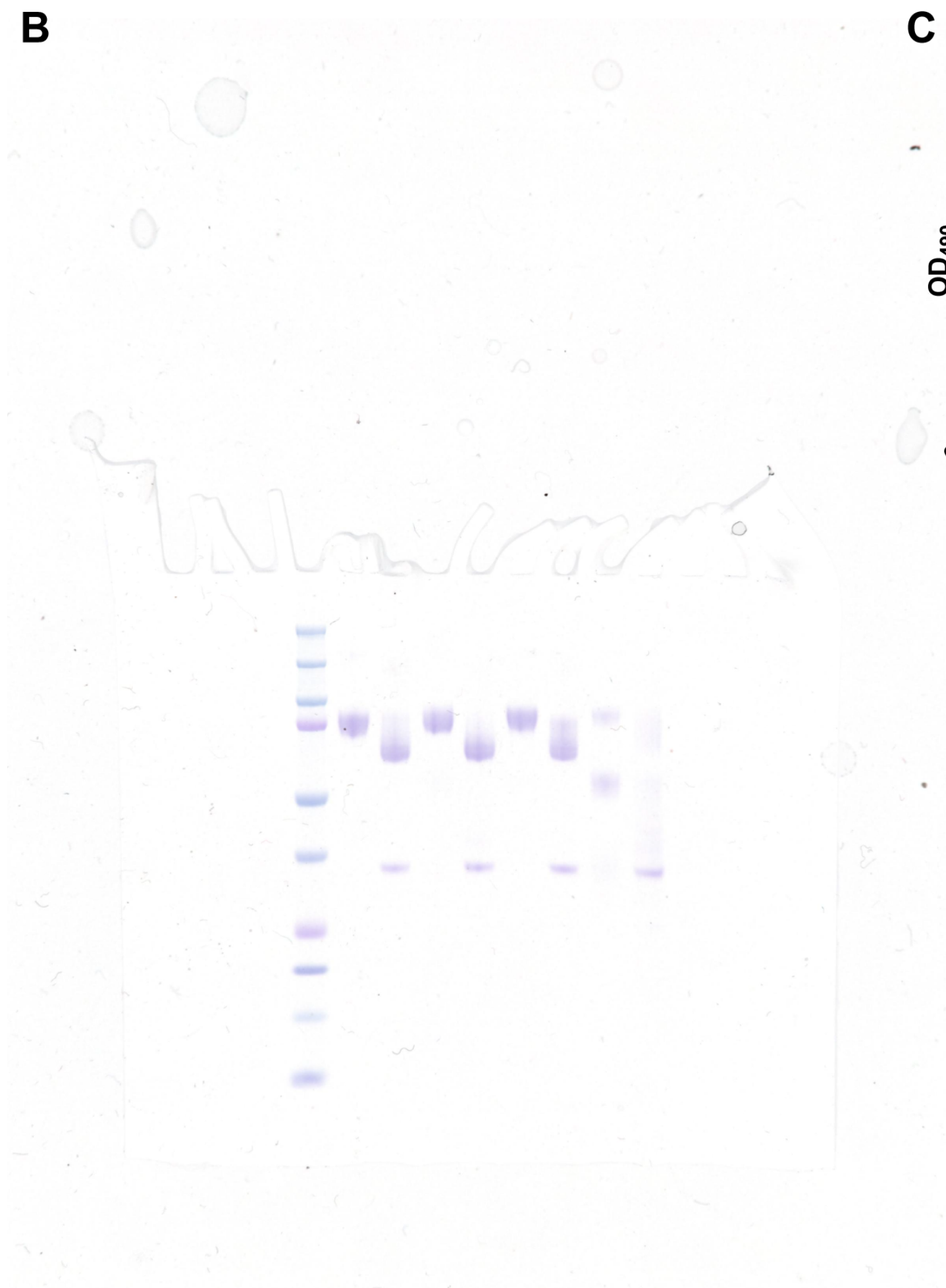

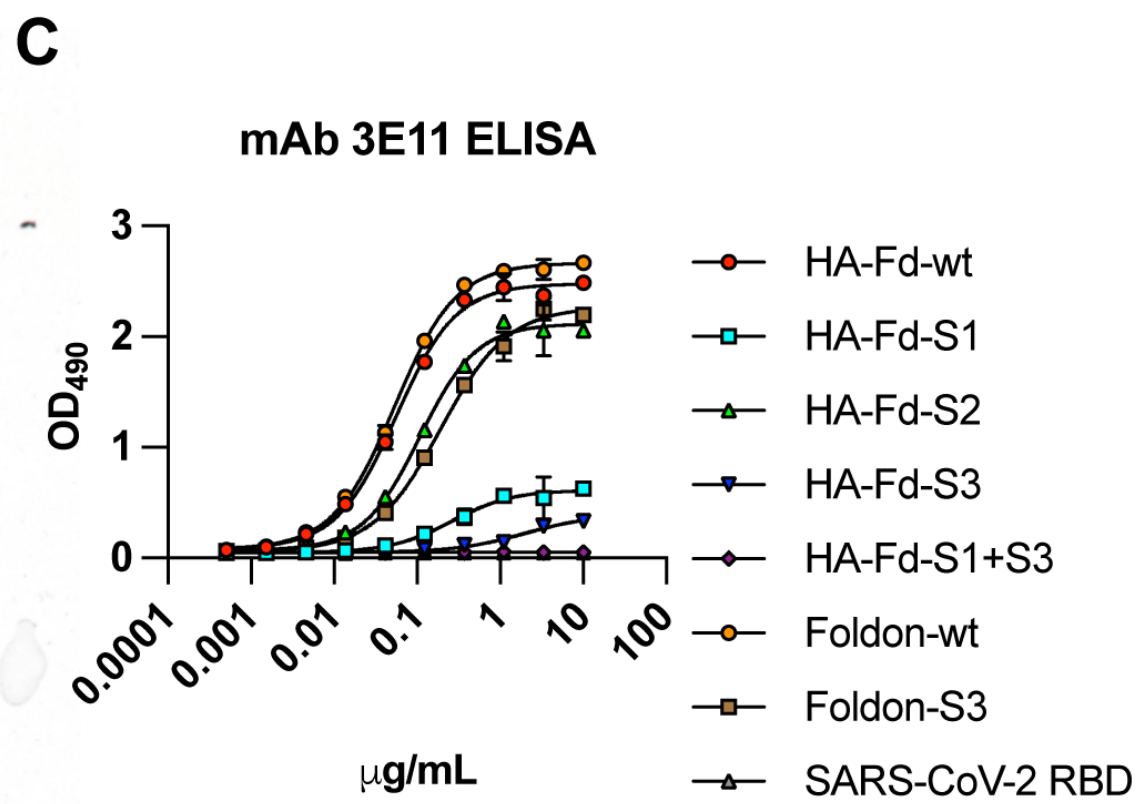

Plating Antigen	EC_{50} (ng/mL)
HA-Fd-wt	55.90
HA-Fd-S1	262.0
HA-Fd-S2	107.8
HA-Fd-S3	1952
HA-Fd-S1+S3	N/A
Foldon-wt	53.68
Foldon-S3	190.6
SARS-CoV-2 RDB	N/A

S5 Fig. Related to Fig 4. Expanded data for assessment of Foldon glycosylation and the impact on antibody binding. (A) Complete replicate data from the BLI experiments. (B) Uncropped image from the SDS-PAGE analysis. (C) Additional ELISA data of mAb 3E11 binding with different proteins. Each data point and its associated error bar represents the mean ± standard deviation of two technical replicates.

A

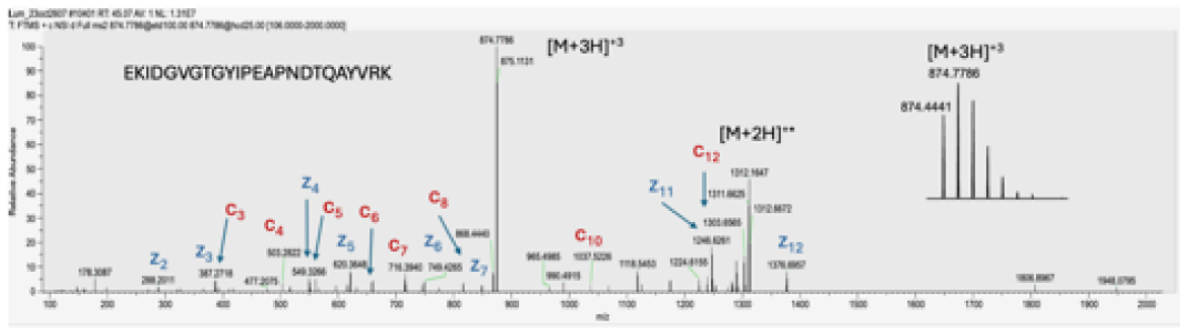

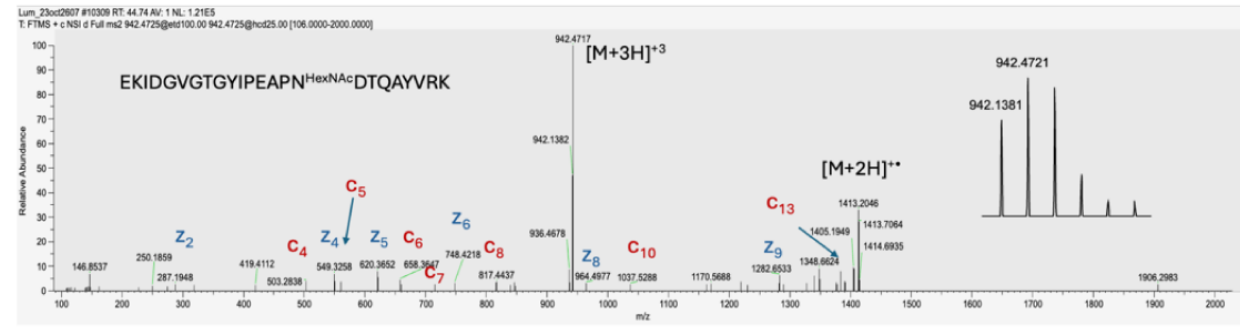

Summary of the N513 glycosylated peptides identified in LC-MS/MS analysis in HA-Foldon-S1.

[M+H]	m/z	z	Δm	Retention Time		Modification	Sequence	Score	Peak Areas			
			Da	Sec	Min	Mass			WT	S1	S2	S3
2621.3	874.4441	3	-0.0004	2970.0	49.50	0	EKIDGVGTGYIPEAPNDTQAYVRK	5.51[a]	0	1219613705	0	0
2824.4	942.1388	3	0.0026	2866.2	47.77	203.07937	EKIDGVGTGYIPEAPN^{HexNAc}DTQAYVRK	4.75[a]	0	5615925	0	0
3861.8	966.4489	4	0.0022	2637.0	43.95	1241.4545	EKIDGVGTGYIPEAPN$^{HexNAc(3)Hex(3)Fuc(1)}$DTQAYVRK	33.89	0	20388314	0	0
4267.9	1067.989	4	0.0017	2623.4	43.72	1647.6132	EKIDGVGTGYIPEAPN$^{HexNAc(5)Hex(3)Fuc(1)}$DTQAYVRK	45.261	0	37964353	0	0
4430.0	1108.503	4	-0.0004	2613.9	43.56	1809.6661	EKIDGVGTGYIPEAPN$^{HexNAc(5)Hex(4)Fuc(1)}$DTQAYVRK	16.325	0	6543846	0	0
4471.0	1118.761	4	0.0013	2615.4	43.59	1850.6927	EKIDGVGTGYIPEAPN$^{HexNAc(6)Hex(3)Fuc(1)}$DTQAYVRK	32.658	0	36430036	0	0
4633.1	1159.272	4	0.0003	2608.9	43.48	2012.7455	EKIDGVGTGYIPEAPN$^{HexNAc(6)Hex(4)Fuc(1)}$DTQAYVRK	17.153	0	9743381	0	0
4680.0	1171.018	4	-0.0004	2834.4	47.24	2059.735	EKIDGVGTGYIPEAPN$^{HexNAc(4)Hex(5)Fuc(1)NeuAc(1)}$DTQAYVRK	22.638	0	10317148	0	0
4721.1	1181.275	4	-0.0006	2836.7	47.28	2100.7615	EKIDGVGTGYIPEAPN$^{HexNAc(5)Hex(4)Fuc(1)NeuAc(1)}$DTQAYVRK	20.493	0	6299961	0	0
4883.1	1221.79	4	-1.0191	2829.4	47.16	2263.8347	EKIDGVGTGYIPEAPN$^{HexNAc(5)Hex(5)Fuc(3)}$DTQAYVRK	14.451	0	7854511	0	0
4971.1	1243.79	4	0.0351	3085.6	51.43	2350.7927	EKIDGVGTGYIPEAPN$^{HexNAc(2)Hex(12)}$DTQAYVRK	13.961	0	8237527	0	0
5045.2	1262.303	4	-0.0026	2819.7	46.99	2424.8671	EKIDGVGTGYIPEAPN$^{HexNAc(5)Hex(6)Fuc(1)NeuAc(1)}$DTQAYVRK	19.808	0	6799533	0	0
5086.2	1272.559	4	-0.0069	2820.5	47.01	2465.8938	EKIDGVGTGYIPEAPN$^{HexNAc(6)Hex(5)Fuc(1)NeuAc(1)}$DTQAYVRK	18.641	0	5487429	0	0
5248.3	1313.072	4	5.959	2815.3	46.92	2621.9836	EKIDGVGTGYIPEAPN$^{HexNAc(6)Hex(6)Fuc(1)NeuAc(1)}$DTQAYVRK	12.833	0	1709331	0	0
5336.3	1335.076	4	-0.0044	3067.2	51.12	2715.9626	EKIDGVGTGYIPEAPN$^{HexNAc(5)Hex(6)Fuc(1)NeuAc(2)}$DTQAYVRK	28.073	0	13633886	0	0
5539.4	1385.845	4	-0.0013	3060.8	51.01	2919.042	EKIDGVGTGYIPEAPN$^{HexNAc(6)Hex(6)Fuc(1)NeuAc(2)}$DTQAYVRK	19.166	0	2604447	0	0
5627.4	1407.848	4	-0.0056	3347.1	55.78	3007.058	EKIDGVGTGYIPEAPN$^{HexNAc(5)Hex(6)Fuc(1)NeuAc(3)}$DTQAYVRK	16.248	0	4712801	0	0
5701.4	1426.351	4	-0.0048	3045.5	50.76	3081.0948	EKIDGVGTGYIPEAPN$^{HexNAc(6)Hex(7)Fuc(1)NeuAc(2)}$DTQAYVRK	17.725	0	7199398	0	0

B

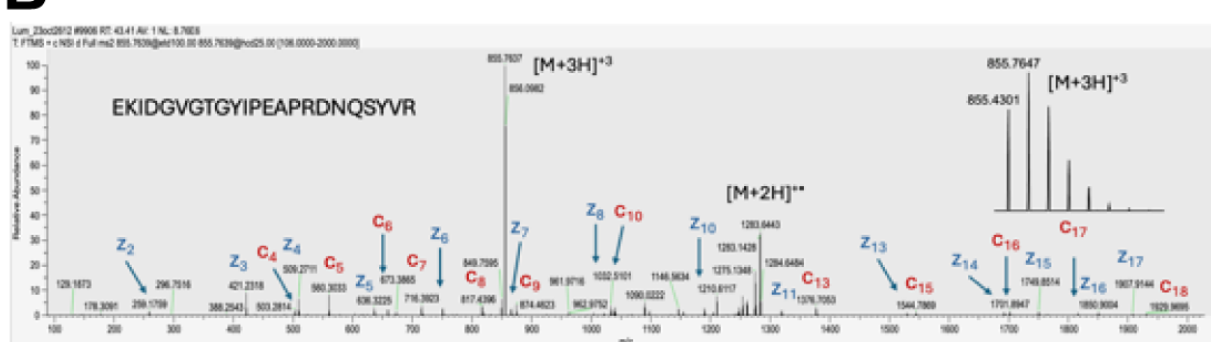

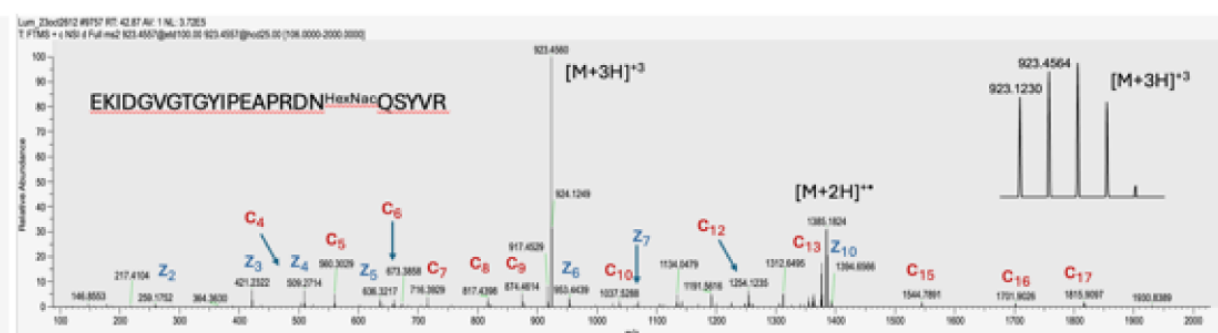

Summary of the N515 glycosylated peptides identified in LC-MS/MS analysis in HA-Foldon-S2.

[M+H]	m/z	z	Δm	Retention Time		Modification	Sequence	Score	Peak Areas			
			Da	Sec	Min	Mass			WT	S1	S2	S3
2307.1	1154.071	2	-0.00043	2990.4	49.84	-	IDGVGTGYIPEAPRDNQSYVR	2.25[a]	0	0	40579477	0
2564.3	855.4301	3	-0.00012	2867.4	47.79	-	EKIDGVGTGYIPEAPRDNQSYVR	2.91[a]	0	0	712386900	0
2638.3	660.3337	4	0.00054	2561.4	42.69	203.0794	IDGVGTGYIPEAPRDN^{HexNac}QSYVRK	3.71[a]	0	0	13006803	0
2767.3	923.123	3	-0.0021	2742.6	45.71	203.0794	EKIDGVGTGYIPEAPRDN^{HexNac}QSYVR	2.61[a]	0	0	17442764	0
3401.5	1134.8496	3	-0.0258	2711.5	45.19	1095.3966	IDGVGTGYIPEAPRDN$^{HexNAc(3)Hex(3)}$QSYVR	19.775	0	0	10384795	0
3522.6	1175.192	3	-0.0006	2697.0	44.95	1216.4229	IDGVGTGYIPEAPRDN$^{HexNAc(2)Hex(5)}$QSYVR	14.203	0	0	21270347	0
3779.7	1260.904	3	-0.0035	2485.2	41.42	1216.4229	EKIDGVGTGYIPEAPRDN$^{HexNAc(2)Hex(5)}$QSYVR	27.079	0	0	11676201	0
3547.6	1183.5372	3	0.0008	2703.9	45.06	1241.4545	IDGVGTGYIPEAPRDN$^{HexNAc(3)Hex(3)Fuc(1)}$QSYVR	17.199	0	0	13053301	0
3804.7	1269.2477	3	-0.0019	2500.7	41.68	1241.4545	EKIDGVGTGYIPEAPRDN$^{HexNAc(3)Hex(3)Fuc(1)}$QSYVR	14.937	0	0	5076465	0
3604.6	1202.5431	3	0.0015	2699.4	44.99	1298.4760	IDGVGTGYIPEAPRDN$^{HexNAc(4)Hex(3)}$QSYVR	16.192	0	0	11290211	0
3750.7	1251.2282	3	-0.0027	2696.4	44.94	1444.5339	IDGVGTGYIPEAPRDN$^{HexNAc(4)Hex(3)Fuc(1)}$QSYVR	32.106	0	0	36424968	0
4007.8	1336.9406	3	-0.0027	2495.0	41.58	1444.5339	EKIDGVGTGYIPEAPRDN$^{HexNAc(4)Hex(3)Fuc(1)}$QSYVR	31.952	0	0	2829726	0
4169.9	1390.9588	3	-0.0018	2484.3	41.41	1606.5867	EKIDGVGTGYIPEAPRDN$^{HexNAc(4)Hex(4)Fuc(1)}$QSYVR	25.259	0	0	13058319	0
3953.7	1318.9206	3	-0.0034	2682.1	44.70	1647.6132	IDGVGTGYIPEAPRDN$^{HexNAc(5)Hex(3)Fuc(1)}$QSYVR	25.365	0	0	24593245	0
4075.8	1359.2626	3	-0.0009	2665.2	44.42	1768.6396	IDGVGTGYIPEAPRDN$^{HexNAc(4)Hex(5)Fuc(1)}$QSYVR	39.263	0	0	134786815	0
4278.9	1426.9565	3	-0.0001	2661.0	44.35	1971.7189	IDGVGTGYIPEAPRDN$^{HexNAc(5)Hex(5)Fuc(1)}$QSYVR	23.939	0	0	11763642	0
4697.0	1566.6873	3	-0.0037	2456.6	40.94	2133.7717	EKIDGVGTGYIPEAPRDN$^{HexNAc(5)Hex(6)Fuc(1)}$QSYVR	14.353	0	0	12980581	0
4731.0	1578.0071	3	-0.0029	2908.5	48.48	2424.8671	IDGVGTGYIPEAPRDN$^{HexNAc(5)Hex(6)Fuc(1)NeuAc(1)}$QSYVR	16.518	0	0	28901653	0
4806.0	1602.6863	3	-0.0013	2626.2	43.77	2498.9040	IDGVGTGYIPEAPRDN$^{HexNAc(6)Hex(7)Fuc(1)}$ QSYVR	44.015	0	0	19376553	0
5428.3	1357.8322	4	0.0008	2418.0	40.30	2864.0362	EKIDGVGTGYIPEAPRDN$^{HexNAc(7)Hex(8)Fuc(1)}$ QSYVR	14.875	0	0	29583633	0
5645.4	1412.0983	4	-0.0007	2911.8	48.53	3081.0948	EKIDGVGTGYIPEAPRDN$^{HexNAc(6)Hex(7)Fuc(1)NeuAc(2)}$QSYVR	19.25	0	0	32641264	0

C

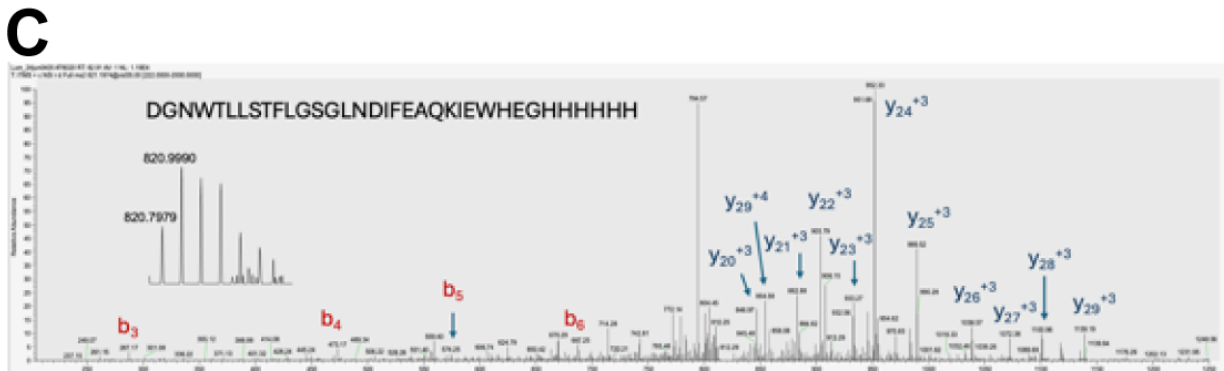

Summary of the N524 peptide identified in LC-MS/MS analysis in HA-Foldon-S3.

[M+H]	m/z	z	Δm	Retention Time		Modification	Sequence	Score	Peak Areas			
			Da	Sec	Min	Mass			WT	S1	S2	S3
4099.9	820.7979	5	0.0054	4974.6	82.91	-	DGNWTLLSTFLGSGLNDIFEAQKIEWHEGHHHHHH	-	0	0	0	2438109

S6 Fig. Related to Fig 4. Summary data from mass spectrometry. (A) Representative spectra and summary table of fragments containing N513, modified or unmodified, in the mass spectrometry analysis. (B) Representative spectra and summary table of fragments containing N515, modified or unmodified, in the mass spectrometry analysis. (C) Representative spectrum and summary table of the fragment containing N524, unmodified, in the mass spectrometry analysis.

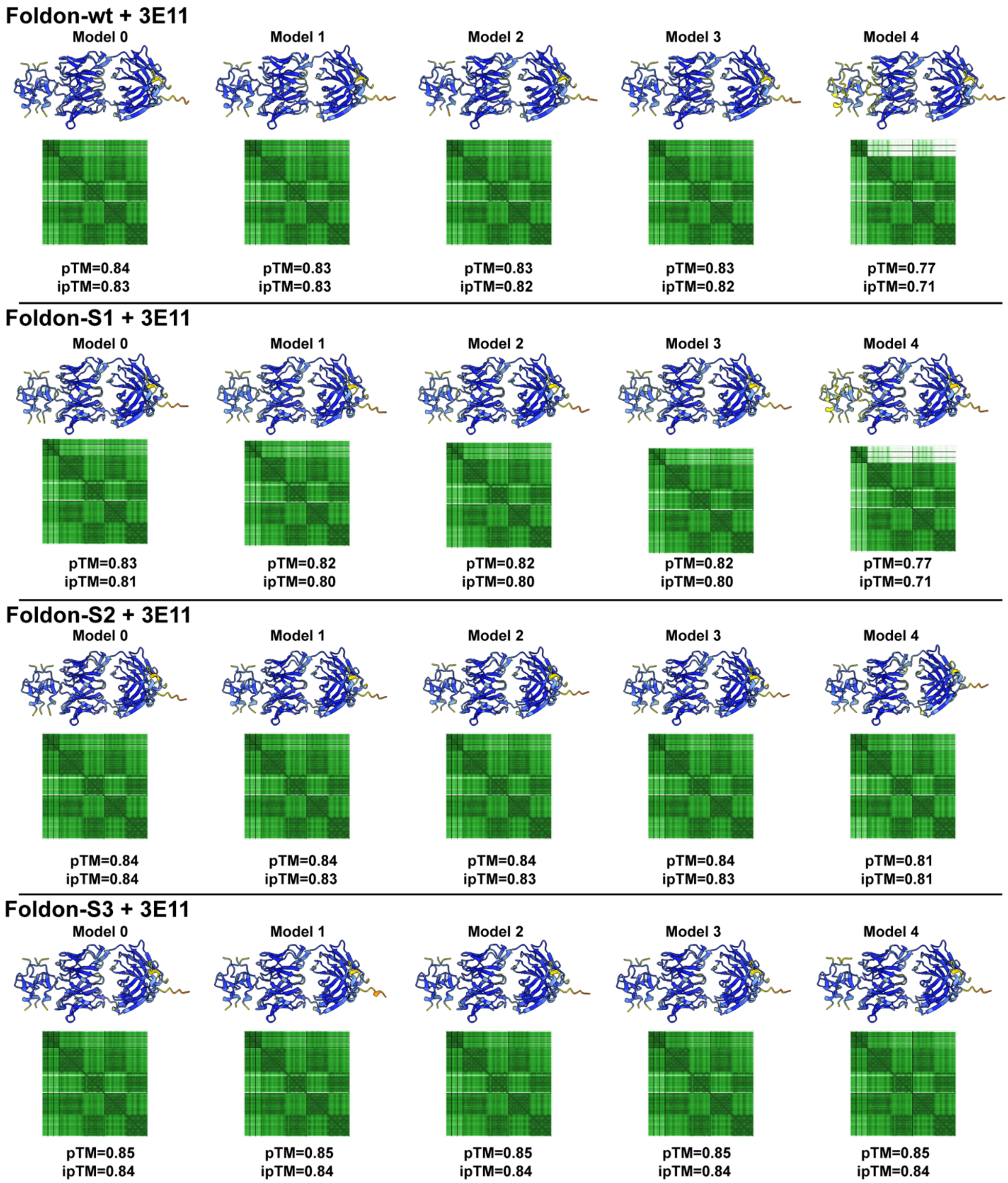

S7 Fig. Related to Fig 5. Expanded details on AlphaFold3 predicted models of unglycosylated Foldon proteins bound to Fab 3E11. The five predicted models are shown for each protein construct with residues colored based on the predicted Local Distance Difference Test (pLDDT) values. Predicted alignment error (PAE) plots are depicted as green matrices. The predicted template modeling (pTM) and interface predicted template modeling scores for each model are shown.
