# Supplementary material for: Immunogenicity of the Foldon trimerization domain and efforts to shield it
Source: PLoS One. 2026 Nov 13;21(11):e0362571. doi: 10.1371/journal.pone.0362571 (PMC13766278; doi:10.1371/journal.pone.0362571)

Fractions of HA-Fd-S1 and HA-Fd-wt from size exclusion chromatoagraphy (S2 Fig panel B)

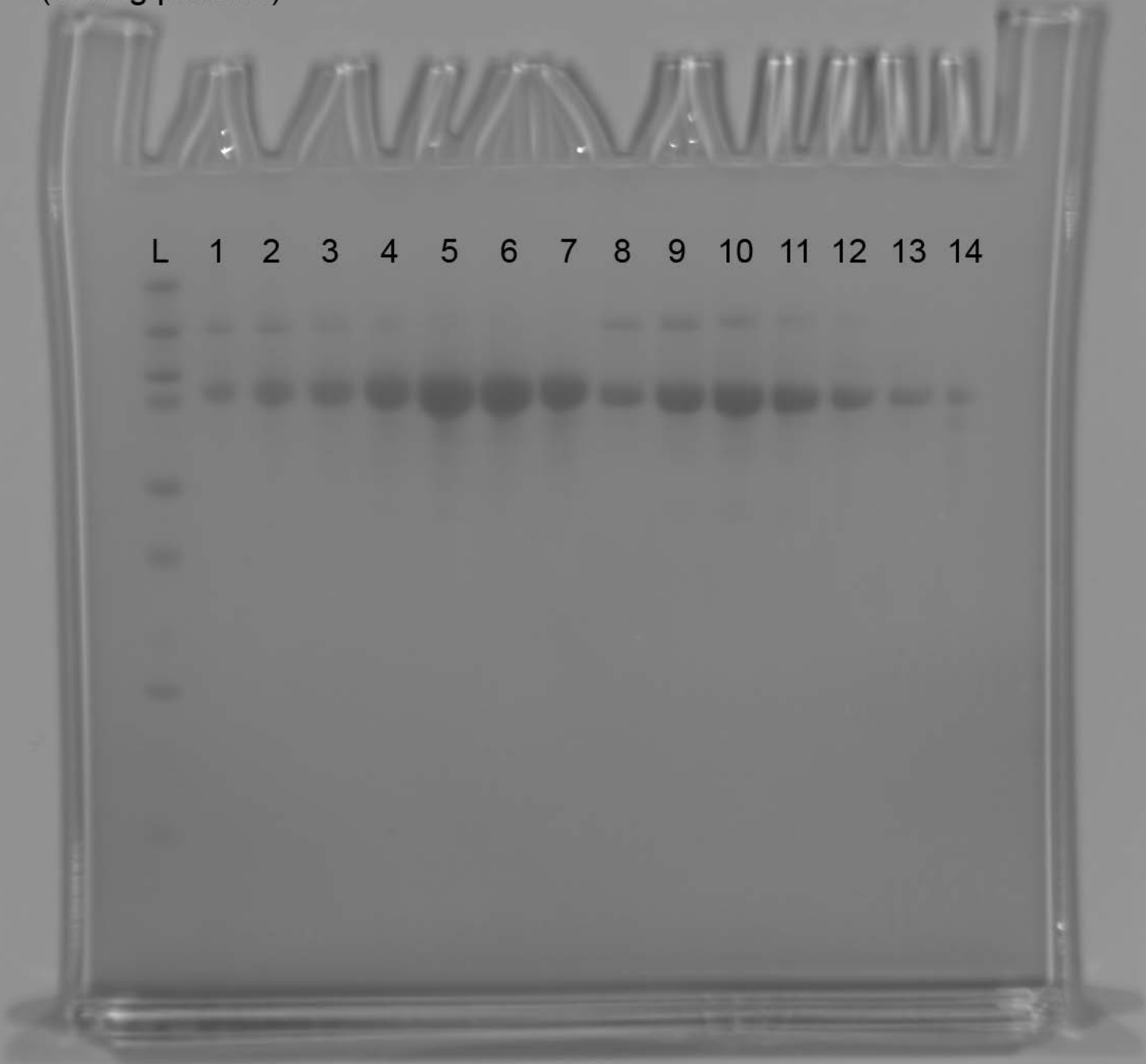

L = Ladder

1-7 = HA-Fd-S1+S3 from size exclusion chromatography

8-14 = HA-Fd-wt from size exclusion chromatography

Foldon from thrombin-digested recombinant HA proteins purified by size exclusion chromatography expressed in Sf9 cells (S3 Fig panel A)

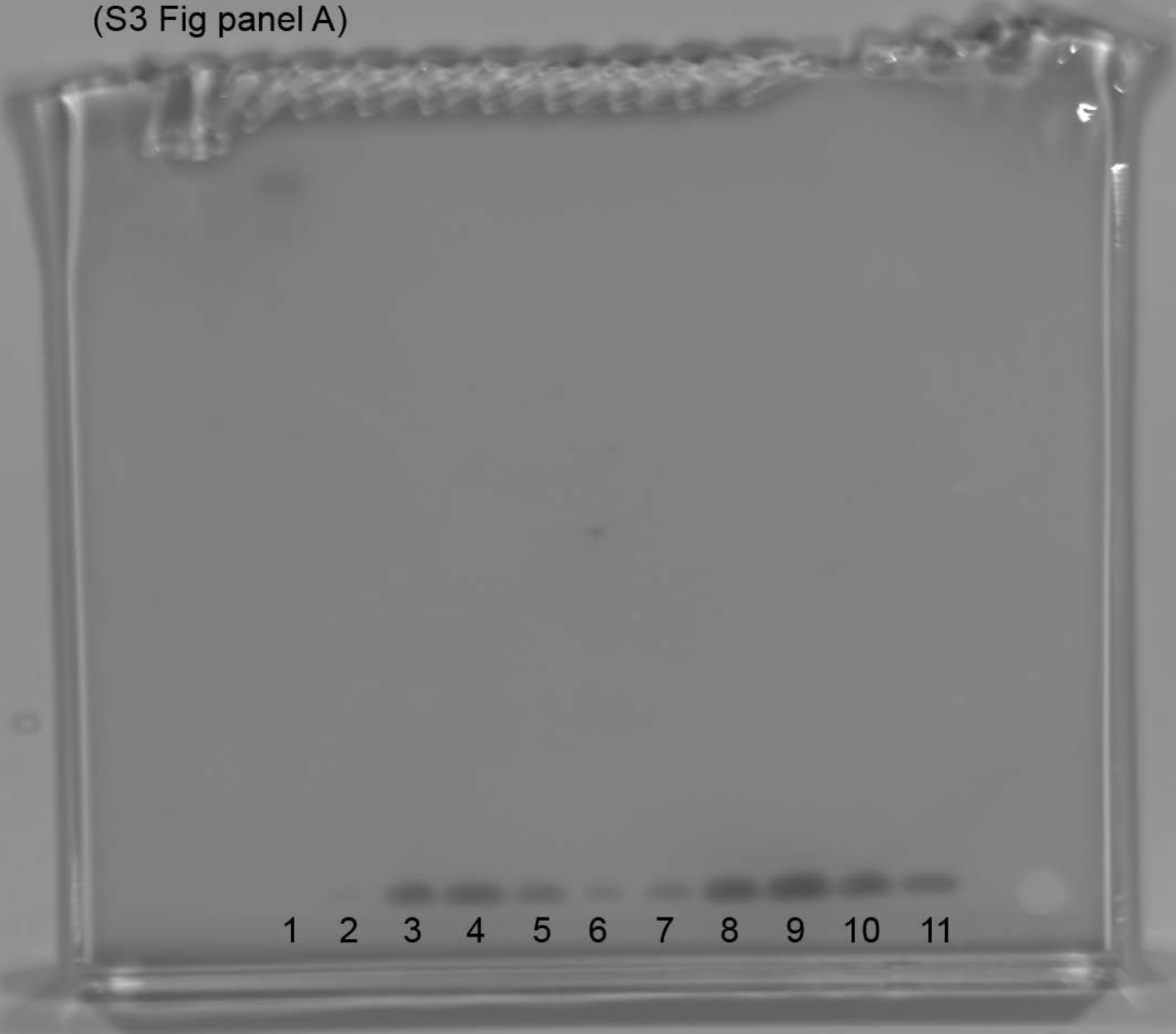

1 = Unrelated protein sample
2-6 = Foldon derived from COBRA HA X-6
7-11 = Foldon derived from COBRA HA Y2

Purification of Strep-tagged Foldon expressed in *Escherichia coli*
(S3 Fig panel B)

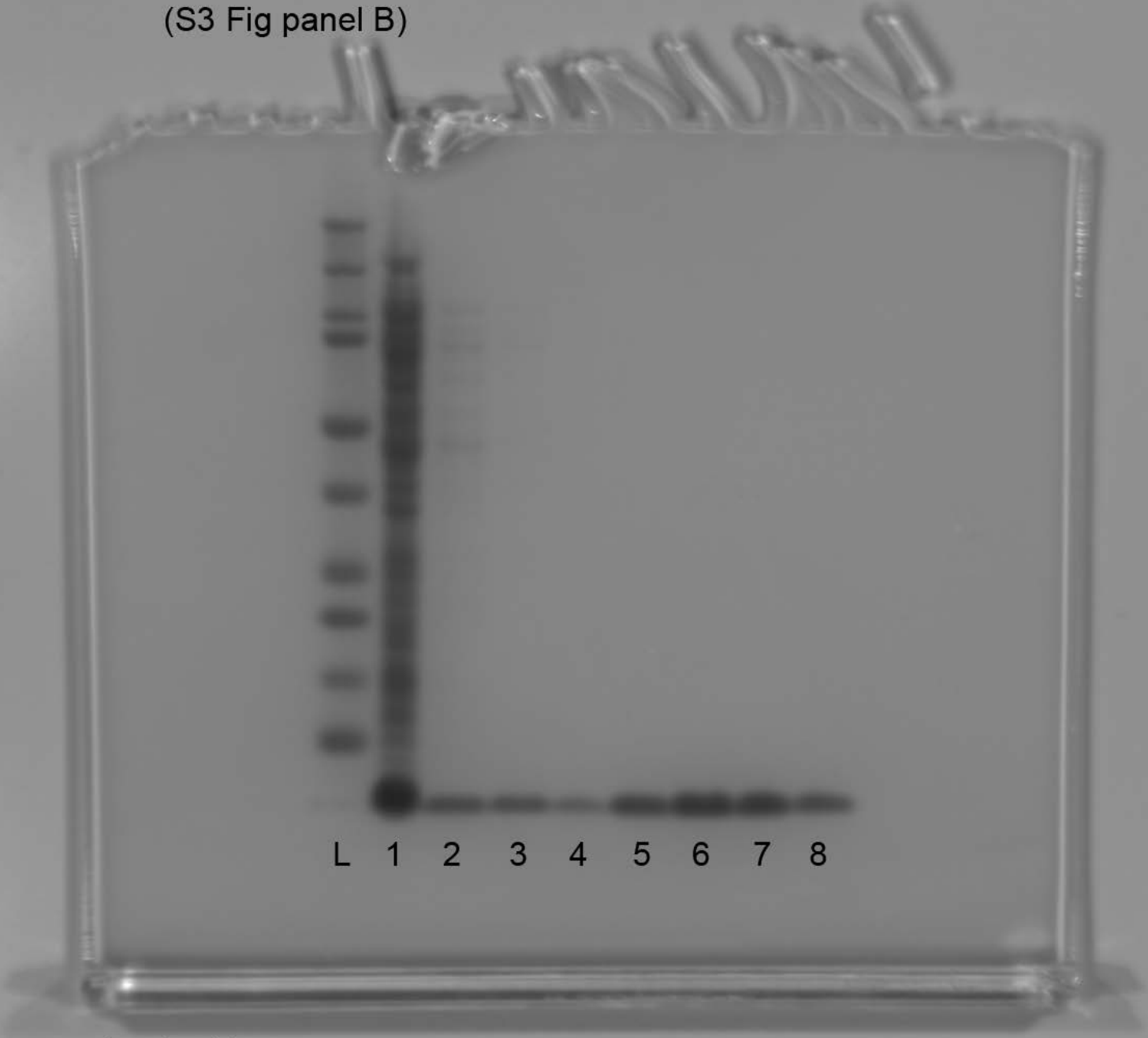

L = Ladder
1 = Pre-column
2 = Streptactic column wash
3 = Streptactin column elution
4-8 = Fractions from size exclusion chromatography

PNGase F digestion of HA-Foldon samples (Fig 4 panel A and S5 Fig panel B)

L = Ladder
1 = HA-Fd-wt PNGase F (-)
2 = HA-Fd-wt PNGase F (+)
3 = HA-Fd-S1 PNGase F (-)
4 = HA-Fd-S1 PNGase F (+)
5 = HA-Fd-S2 PNGase F (-)
6 = HA-Fd-S2 PNGase F (+)
7 = HA-Fd-S3 PNGase F (-)
8 = HA-Fd-S3 PNGase F (+)

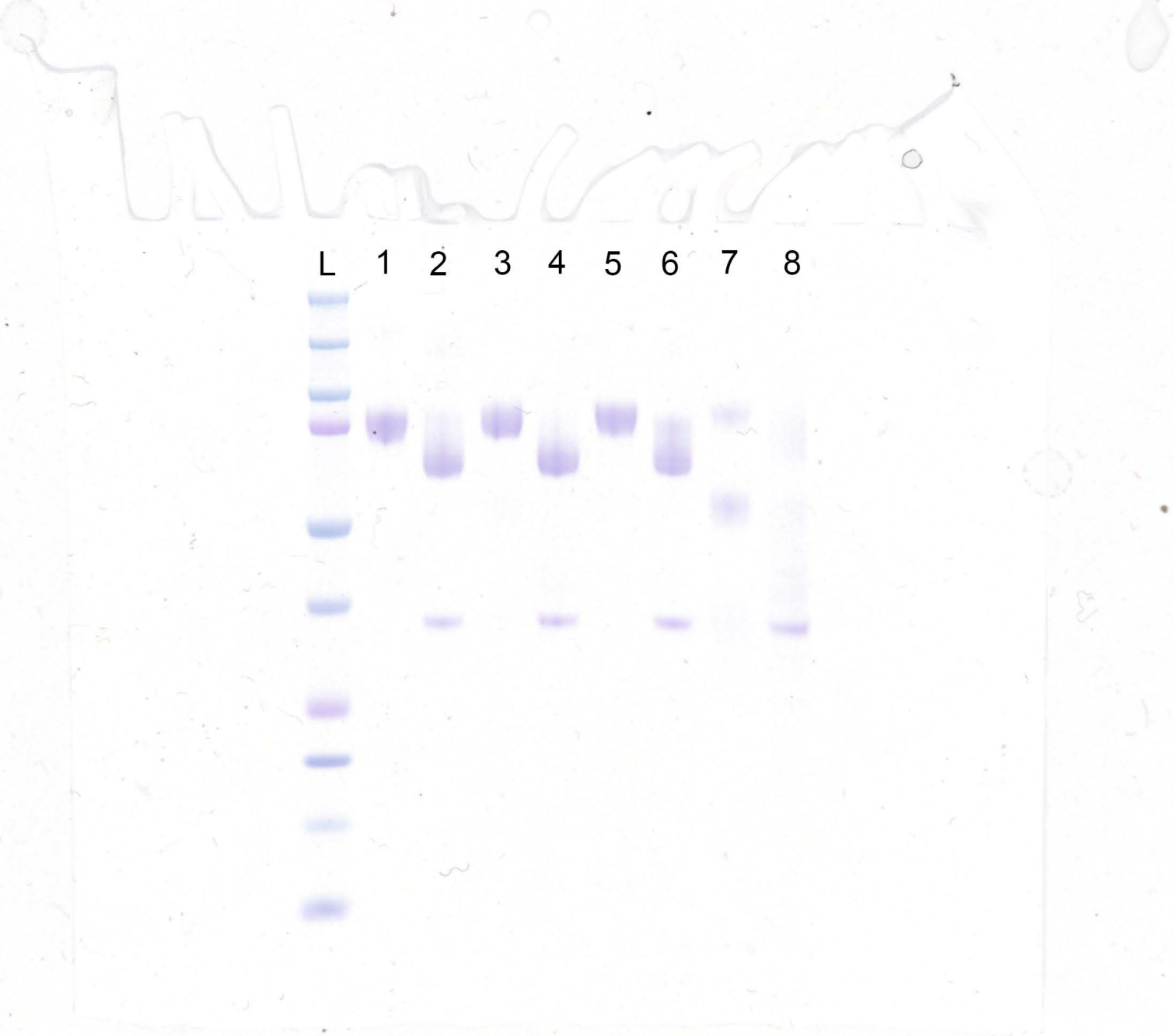

Supplement: Supplementary file 2 [file pone.0362571.s006.pdf]
