# Supplementary material for: Immunogenicity of the Foldon trimerization domain and efforts to shield it
Source: PLoS One. 2026 Nov 13;21(11):e0362571. doi: 10.1371/journal.pone.0362571 (PMC13766278; doi:10.1371/journal.pone.0362571)
Supplement: Supplementary file 6 [file pone.0362571.s005.zip › S11_Data/Report number 5037 - Ross Spenser.pdf]

Lerner Research Institute
Mass Spectrometry Laboratory for Protein Sequencing
The Cleveland Clinic Foundation
Belinda Willard, Ph.D., Director
216-444-7170, willarb@ccf.org

Report number: 5037
Title: Sequence of analysis of four protein samples: Hemagglutinin
Date: 11-16-2023

Principal Investigator: Dr. Ted Ross, Global Director for Vaccine development
For: Spencer Pierce

Samples prepared by: Ling Li
Reported by: Belinda Willard
Approved by: Belinda Willard
Charges: 4 Protein PTM @ $ 240 = $960
iLab Service ID: PMC-SP-1669

Brief description of samples and goal of the experiment.

The samples were a four expressed protein samples. Table 1 contains a summary of the samples analyzed in this set. The goal of these studies is to identify sites of glycosylation.

Table 1. Samples received.[a]

	Investigator sample name	Digest sample number	MS file number	Method
			Orbi	
1	Ross-Pierce 9.27.23 in-soln sample Y2-CHO-WT trypsin	SB20-89-33	Lum_23oct2602<br>Lum_23oct2603	CID<br>ETD
2	Ross-Pierce 9.27.23 in-soln sample Y2-CHO-WT GluC	SB20-89-34	Lum_23oct2604<br>Lum_23oct2605	CID<br>ETD
3	Ross-Pierce 9.27.23 in-soln sample Y2-CHO-S1 trypsin	SB20-89-35	Lum_23oct2606<br>Lum_23oct2607	CID<br>ETD
4	Ross-Pierce 9.27.23 in-soln sample Y2-CHO-S2 GluC	SB20-89-36	Lum_23oct2608<br>Lum_23oct2609	CID<br>ETD
5	Ross-Pierce 9.27.23 in-soln sample Y2-CHO-S2 trypsin	SB20-89-37	Lum_23oct2611<br>Lum_23oct2612	CID<br>ETD
6	Ross-Pierce 9.27.23 in-soln sample Y2-CHO-S2 GluC	SB20-89-38	Lum_23oct2613<br>Lum_23oct2614	CID<br>ETD
7	Ross-Pierce 9.27.23 in-soln sample Y2-CHO-S3 trypsin	SB20-89-39	Lum_23oct2615<br>Lum_23oct2616	CID<br>ETD
8	Ross-Pierce 9.27.23 in-soln sample Y2-CHO-S3 GluC	SB20-89-40	Lum_23oct2617<br>Lum_23oct2618	CID<br>ETD

Methods:

The four protein samples submitted for in-solution protein digestion each contain 10 ug of protein with volumes ranged from 7 ul to 24 ul. The volumes for these samples were adjusted to 24 ul using 0.1M TrisHCl pH 8 buffer. A 30 ul aliquot of 8M urea, 0.1M TrisHCl pH 8 buffer was added to each sample. The samples were reduced by adding 2 ul 0.2M dithiothreitol and incubated

at room temperature for 30 minutes. Reduction was followed by alkylation using a 9.6 ul aliquot of 0.2M iodoacetamide and incubating at room temperature in the dark for 30 minutes. The alkylation reaction was quenched by adding 8 ul 0.2M dithiothreitol and incubating at room temperature for 30 minutes. The pH of the samples were adjusted to 8 with 1M triethyl ammonium bicarbonate. The sample volume was adjused to 240 ul using deionized water. The samples were then split into 2 aliquots of 120 ul each. These samples were digested by adding 0.1 ug sequencing grade trypsin or Glu_C and incubating the samples overnight at 37°C. After digestion, the samples were desalted using C18 Ziptips, the eluted samples were dried on a SpeedVac, then reconstituted in 30 ul 0.1% formic acid for LC-MS analysis.

The LC-MS system was a Finnigan LTQ-Obitrap Fusion Lumos hybrid mass spectrometer system. The HPLC column was a Dionex 15 cm x 75 µm id Acclaim Pepmap C18, 2µm, 100 Å reversed-phase capillary chromatography column. Five µL volumes of the extract were injected and the peptides eluted from the column by an acetonitrile/0.1% formic acid gradient at a flow rate of 0.25 µL/min were introduced into the source of the mass spectrometer on-line. The microelectrospray ion source is operated at 2.5 kV. The digest was analyzed using the data dependent multitask capability of the instrument acquiring full scan mass spectra to determine peptide molecular weights and product ion spectra to determine amino acid sequence in successive instrument scans. The data were analyzed by using all CID spectra collected in the experiment to search the human reference sequence database with the search program Mascot. All data was filtered based on a Mascot ion score > 40. In addition, the data was searched specifically against the sequence of mouse and human Akt1 using the program Sequest and considering phosphorylation, methylation, ubiquitination, acetylation, and glycosylation.

Results:

Four protein samples were submitted on 9.27.23. These samples were digested with trypsin and GluC. The digests were analyzed by capillary column LC-tandem MS and the CID spectra searched against the sequence of Hemagglutinin using the program Sequest. These samples were analyzed utilizing an LC gradient from 2 to 70% acetonitrile in 110 minutes. The results are summarized in Table 2, with the specific peptides that were sequenced shown in the attached figures.

SB20-89-33: 9.27.23 in-soln sample Y2-CHO-WT trypsin

The data from this digest was searched specifically against the expressed protein sequences. The WT form of Hemagglutinin was identified by 82 peptides covering 81% of the protein sequence.

SB20-89-34: 9.27.23 in-soln sample Y2-CHO-WT GluC

The data from this digest was searched specifically against the expressed protein sequences. The WT form of Hemagglutinin was identified by 153 peptides covering 93% of the protein sequence.

SB20-89-35: 9.27.23 in-soln sample Y2-CHO-S1 trypsin

The data from this digest was searched specifically against the expressed protein sequences. The S1 form of Hemagglutinin was identified by 64 peptides covering 62% of the protein sequence.

SB20-89-36: 9.27.23 in-soln sample Y2-CHO-S1 GluC

The data from this digest was searched specifically against the expressed protein sequences. The S1 form of Hemagglutinin was identified by 123 peptides covering 83% of the protein sequence.

SB20-89-37: 9.27.23 in-soln sample Y2-CHO-S2 trypsin

The data from this digest was searched specifically against the expressed protein sequences. The S2 form of Hemagglutinin was identified by 86 peptides covering 82% of the protein sequence.

SB20-89-38: 9.27.23 in-soln sample Y2-CHO-S2 GluC

The data from this digest was searched specifically against the expressed protein sequences. The S2 form of Hemagglutinin was identified by 197 peptides covering 97% of the protein sequence.

SB20-89-39: 9.27.23 in-soln sample Y2-CHO-S3 trypsin

The data from this digest was searched specifically against the expressed protein sequences. Hemagglutinin was identified by in this sample by 42 peptides covering 53% of the protein sequence. No peptides were identified that can differentiate the S3 and WT forms of Hemagglutinin.

SB20-89-40: 9.27.23 in-soln sample Y2-CHO-S3 GluC

The data from this digest was searched specifically against the expressed protein sequences. Hemagglutinin was identified by in this sample by 328 peptides covering 93% of the protein sequence. No peptides were identified that can differentiate the S3 and WT forms of Hemagglutinin.

Chromatographic analysis

The chromatograms for all four tryptic digests were compared and three of these samples; WT, S1, and S2 appear very similar. The S3 sample contains several ions that are not identified or are lower abundant in the WT, S1, and S2 samples. Close examination of the MS/MS spectra for some of the abundant S3 peptides were manually interrogated and these peptides were mapped to hemagglutinin but were determined to be semi-tryptic peptides (formed by one tryptic specific cleavage event and one non-specific cleavage event). To confirm the presence of these semi-tryptic peptides in the S3 sample, the data from all four tryptic digests was researched considering both tryptic and semi-tryptic protein digestions. The number of tryptic and semi-tryptic peptides identified in these samples is summarized below. The S3 sample has the largest number of semi-tryptic peptides suggesting that this sample may have undergone a significant amount of proteolysis prior tryptic digestion.

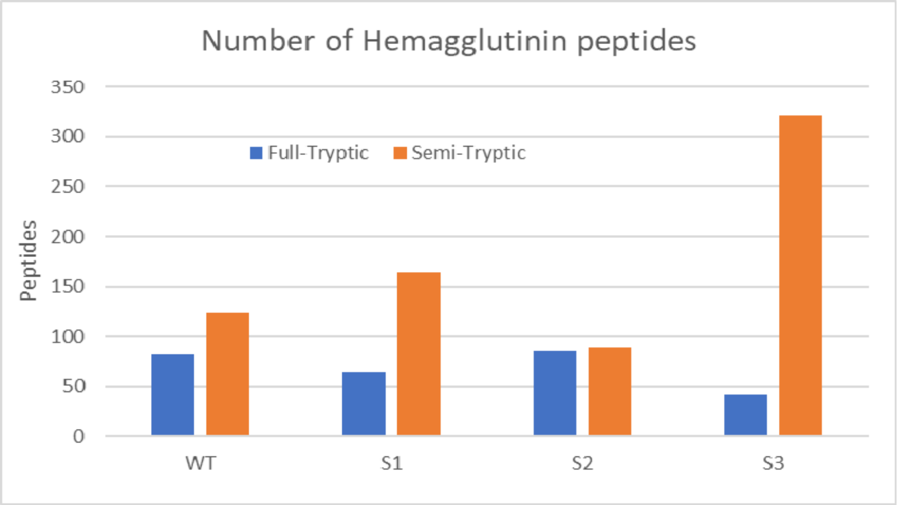

Post Translational modification analysis

The goal of these experiments is to quantify sites of hemagglutinin glycosylation. The CID data was searched specifically against the sequences of Hemagglutinin looking for HexNac modification using the program Sequest. A total of 47 HexNac modified peptides were identified and are given in Table 3. The more complex sugar modifications cannot be identified with CID fragmentation. Due to this, the samples were analyzed using EThcD fragmentation, a method more commonly used for glycosylation analysis. The EThcD data was searched using the program MSFragger specifically against the sequences of Hemagglutinin considering N-linked glycosylation as a variable modification. Several glycosylated peptides were identified, and the results are summarized in Tables 4 to 12 and below.

N481: CDNTCMESVKNGTYDYPK and CDNTCMESVKNGTYDYPKYSEEAK

A total of 42 unique N481 containing tryptic peptides were identified in the LC-MS/MS analysis. These peptides are summarized in Table 4 and 28 different glycosylation forms were identified. Single ion monitoring (SIM) chromatograms were plotted for each N481 peptide, these chromatograms were integrated, and the resulting peak areas which were used to estimate the distribution of these peptides across these samples. The number of N481 glycosylation events is much lower in S3.

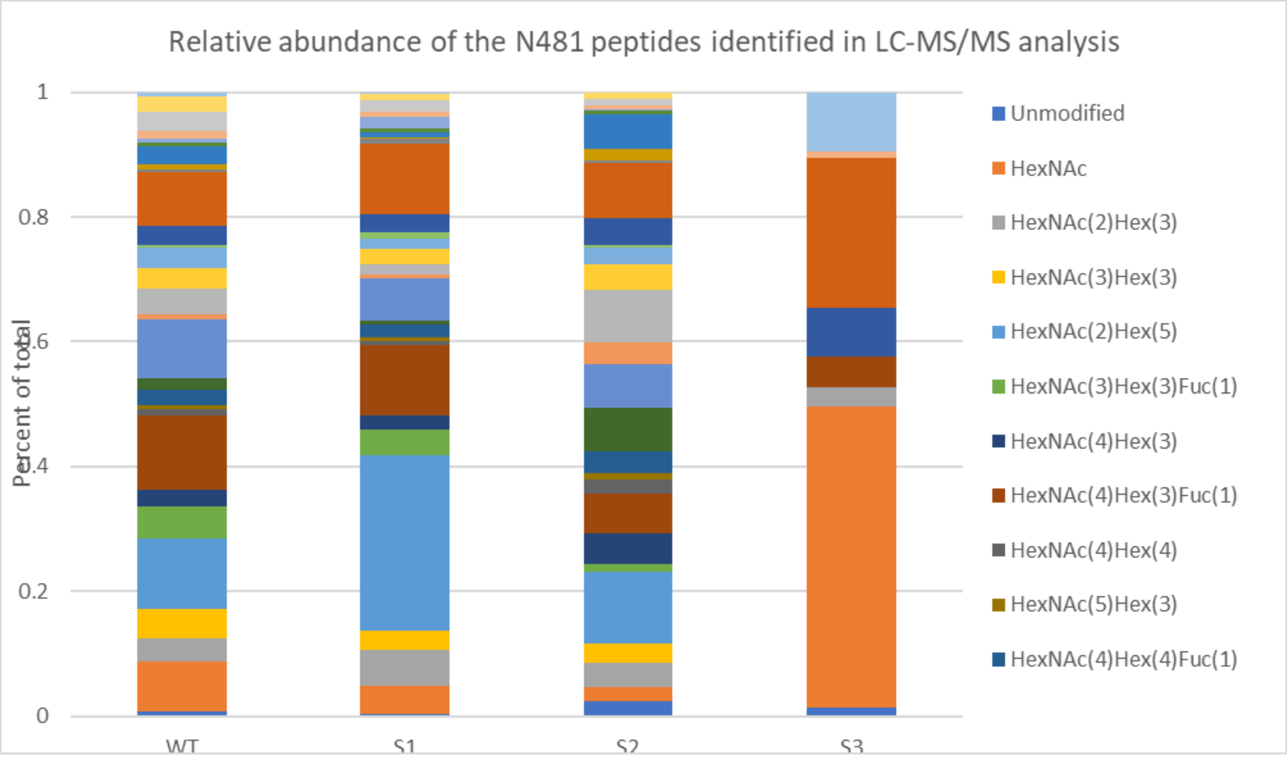

N513: EKIDGVGTGYIPEAPNDTQAYVRK

A total of 18 unique N513 containing tryptic peptides were identified in the LC-MS/MS analysis. These peptides are summarized in Table 5 and 17 different glycosylation forms were identified. Single ion monitoring (SIM) chromatograms were plotted for each N513 peptide, these chromatograms were integrated, and the resulting peak areas which were used to estimate the distribution of these peptides across these samples. This peptide was only identified in the S1 sample.

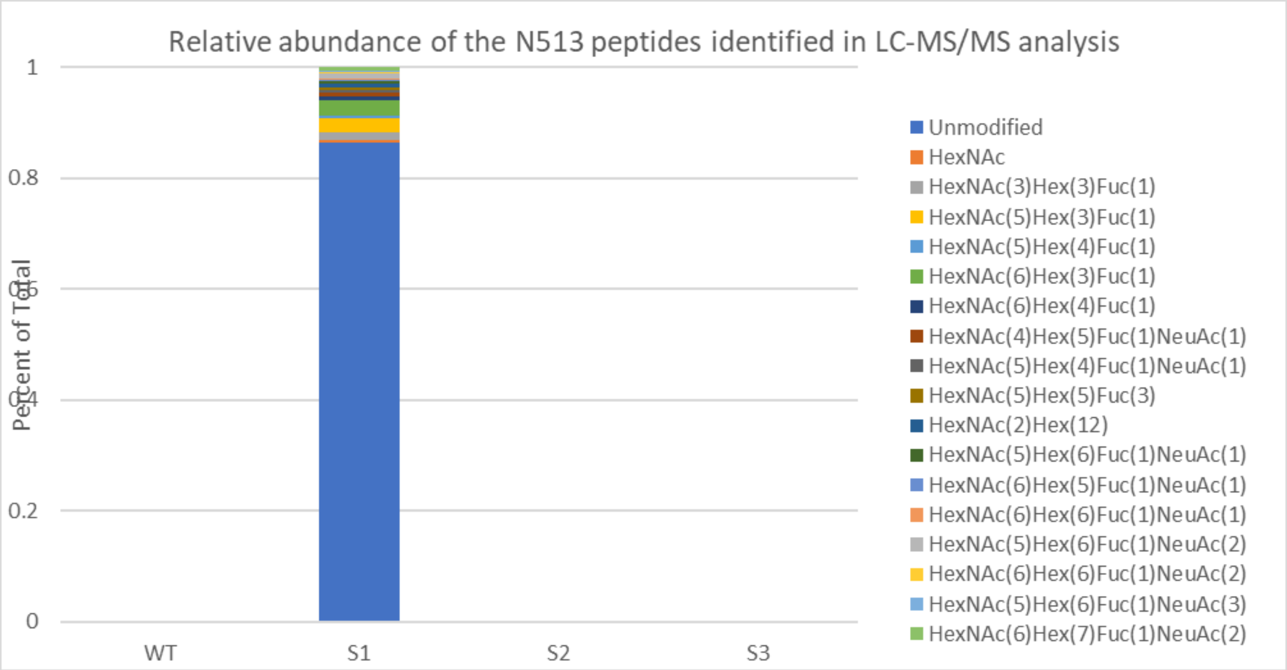

N515: IDGVGTGYIPEAPRDNQSYVR and EKIDGVGTGYIPEAPRDNQSYVR

A total of 21 unique N515 containing tryptic peptides were identified in the LC-MS/MS analysis. These peptides are summarized in Table 6 and 15 different glycosylation forms were identified. Single ion monitoring (SIM) chromatograms were plotted for each N515 peptide, these chromatograms were integrated, and the resulting peak areas which were used to estimate the distribution of these peptides across these samples. This peptide was only identified in the S2 sample.

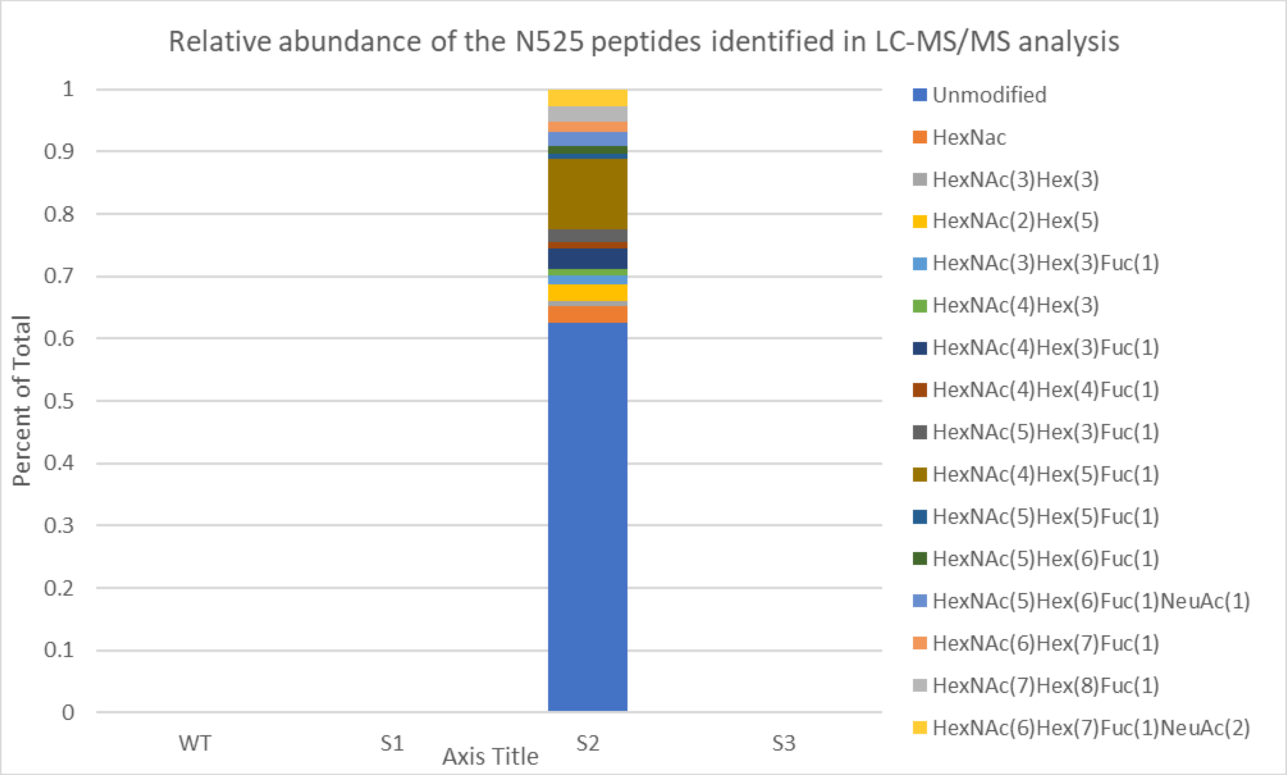

N276-N287: NAGSGIIISDTPVHDCNTTCQTPEGAINTSLPFQNVHPITIGK

A total of 10 unique N276/N287 containing tryptic peptides were identified in the LC-MS/MS analysis. These peptides are summarized in Table 7 and 9 different glycosylation forms were identified. The relative abundance of these peptides was not determined due to inability to identify the modifications on N276 and N287.

N11: DTLCIGYHANNSTDTVDTVLE

A total of 16 unique N11 containing GluC peptides were identified in the LC-MS/MS analysis. These peptides are summarized in Table 8 and 15 different glycosylation forms were identified. Single ion monitoring (SIM) chromatograms were plotted for each N11 peptide, these chromatograms were integrated, and the resulting peak areas were used to estimate the distribution of these peptides across these samples. No N11 containing peptides were identified in the S1 sample and only two were identified in the S3 sample.

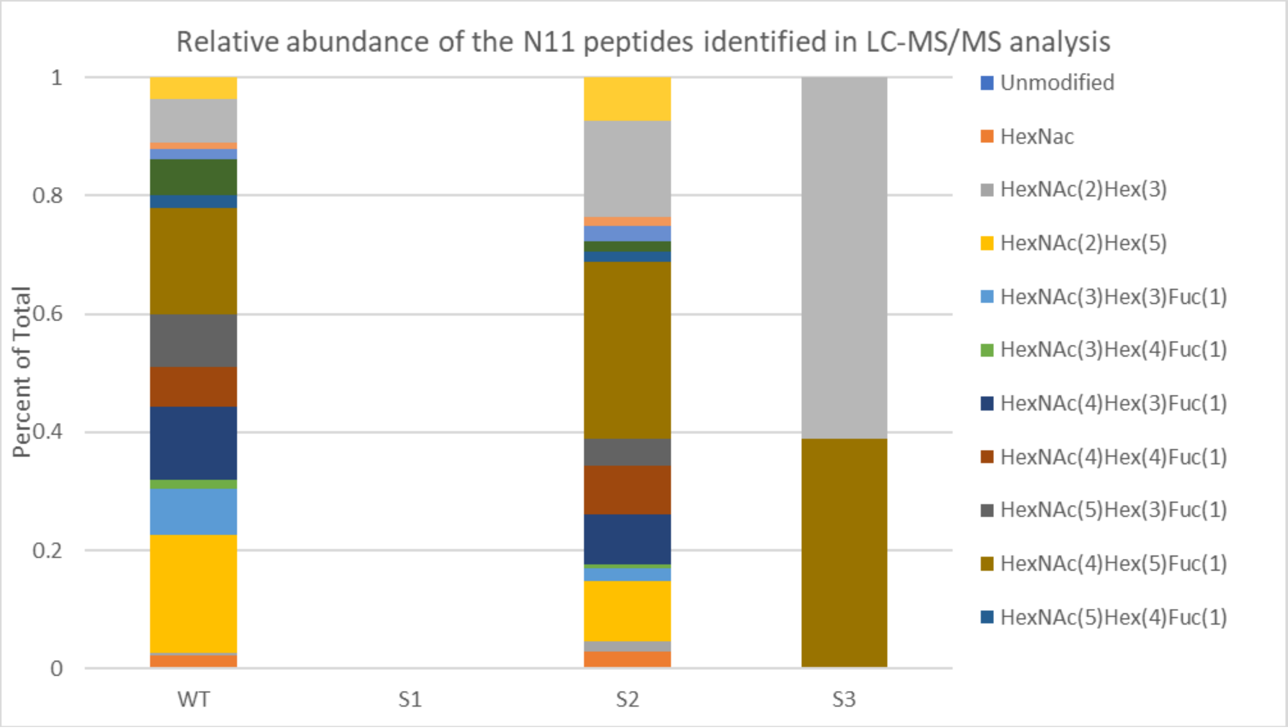

N23: KNVTVTHSVNLLE

A total of 50 unique N23 containing GluC peptides were identified in the LC-MS/MS analysis. These peptides are summarized in Table 9 and 49 different glycosylation forms were identified. Single ion monitoring (SIM) chromatograms were plotted for each N23 peptide, these chromatograms were integrated, and the resulting peak areas were used to estimate the distribution of these peptides across these samples.

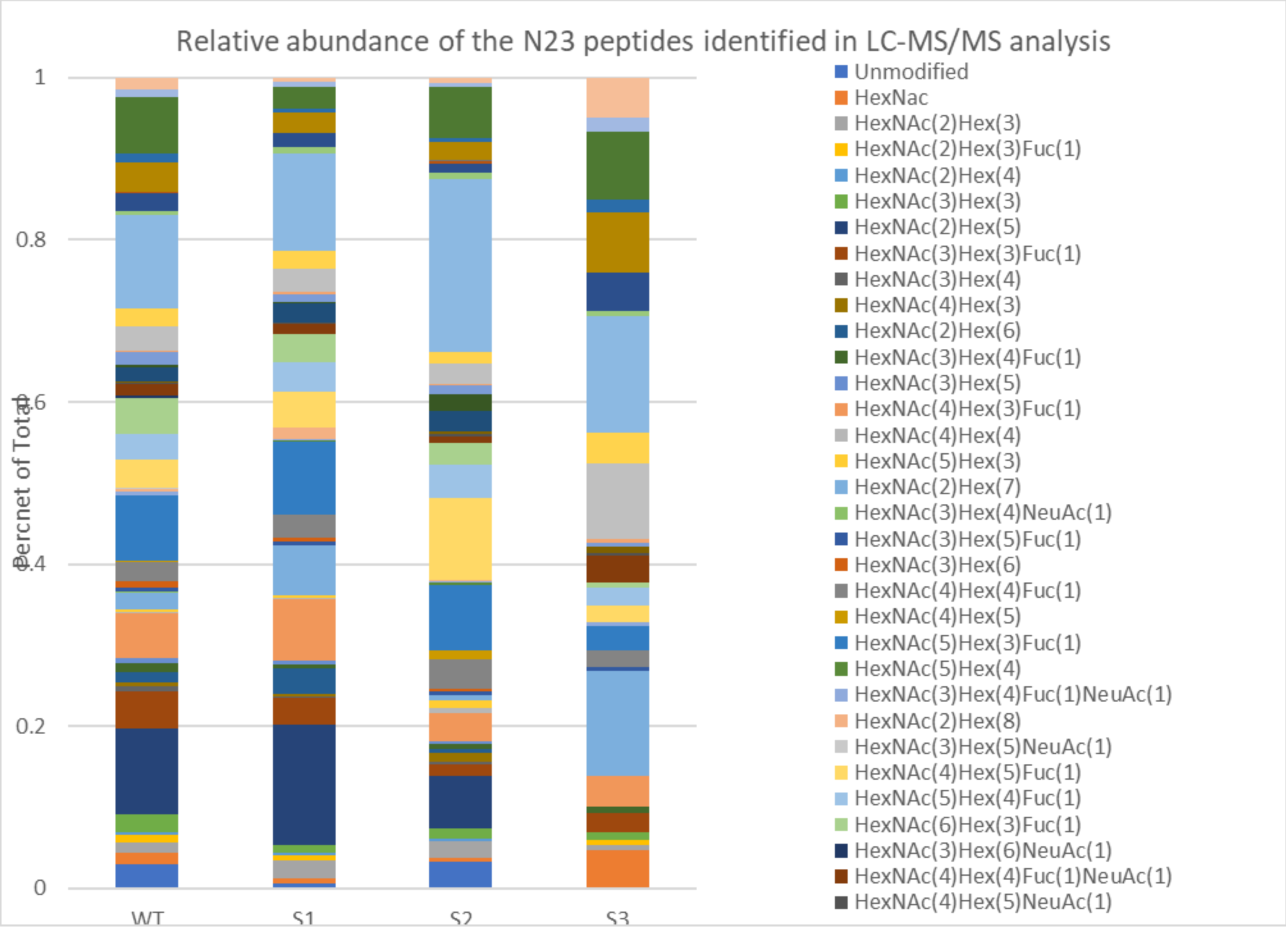

N276: RNAGSGIIISDTPVHDCNTTCQTPE

A total of 41 unique N276 containing GluC peptides were identified in the LC-MS/MS analysis. These peptides are summarized in Table 10 and 40 different glycosylation forms were identified. Single ion monitoring (SIM) chromatograms were plotted for each N276 peptide, these chromatograms were integrated, and the resulting peak areas were used to estimate the distribution of these peptides across these samples.

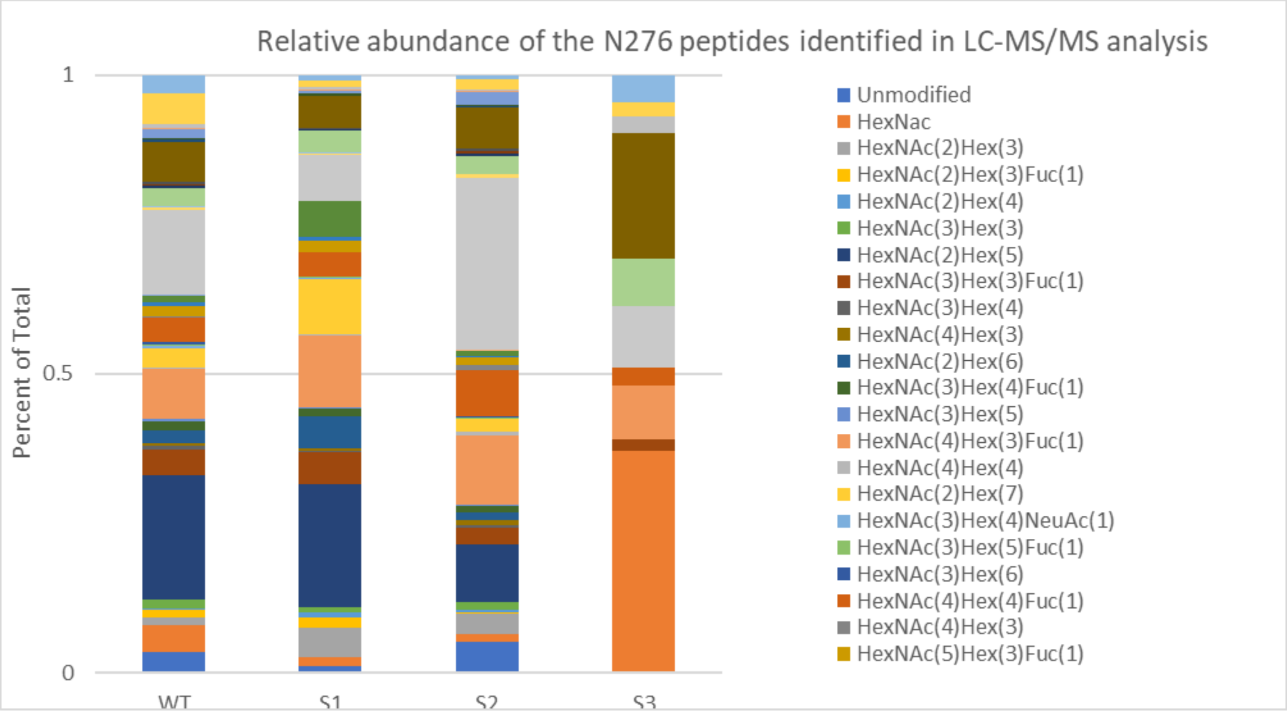

N87: TSNSDNGTCYPGDFINYEE

A total of 18 unique N87 containing GluC peptides were identified in the LC-MS/MS analysis. These peptides are summarized in Table 11 and 17 different glycosylation forms were identified. Single ion monitoring (SIM) chromatograms were plotted for each N87 peptide, these chromatograms were integrated, and the resulting peak areas were used to estimate the distribution of these peptides across these samples.

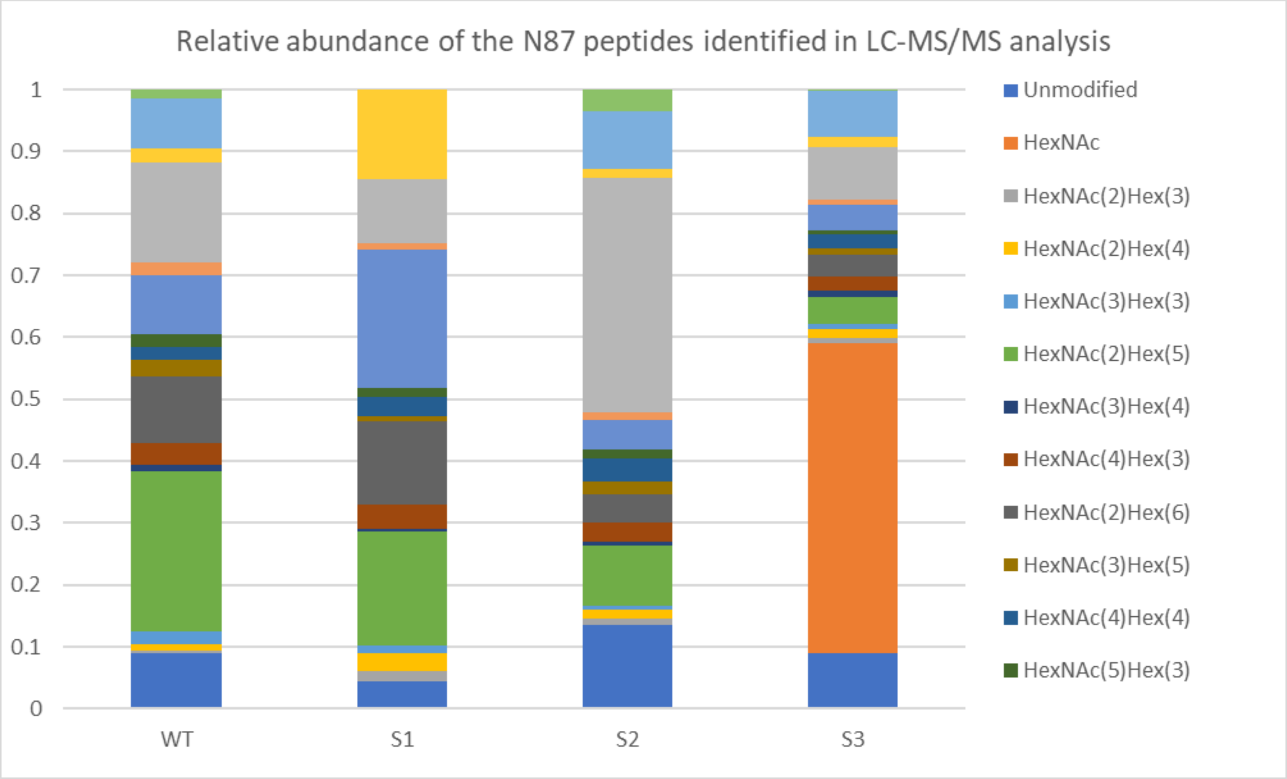

N287: TSNSDNGTCYPGDFINYEE

A total of 2 unique N287 containing GluC peptides were identified in the LC-MS/MS analysis. These peptides are summarized in Table 12 and only 1 glycosylated peptide was identified. Single ion monitoring (SIM) chromatograms were plotted for each N287 peptide, these chromatograms were integrated, and the resulting peak areas were used to estimate the distribution of these peptides across these samples. Please note that the inability to identify additional glycosylation events may reflect the modification status of this site or it may be due to the poor performance of the N287 GluC peptide with EThcD fragmentation. An alternative protease may need to be considered to effectively analyze modifications at this site.

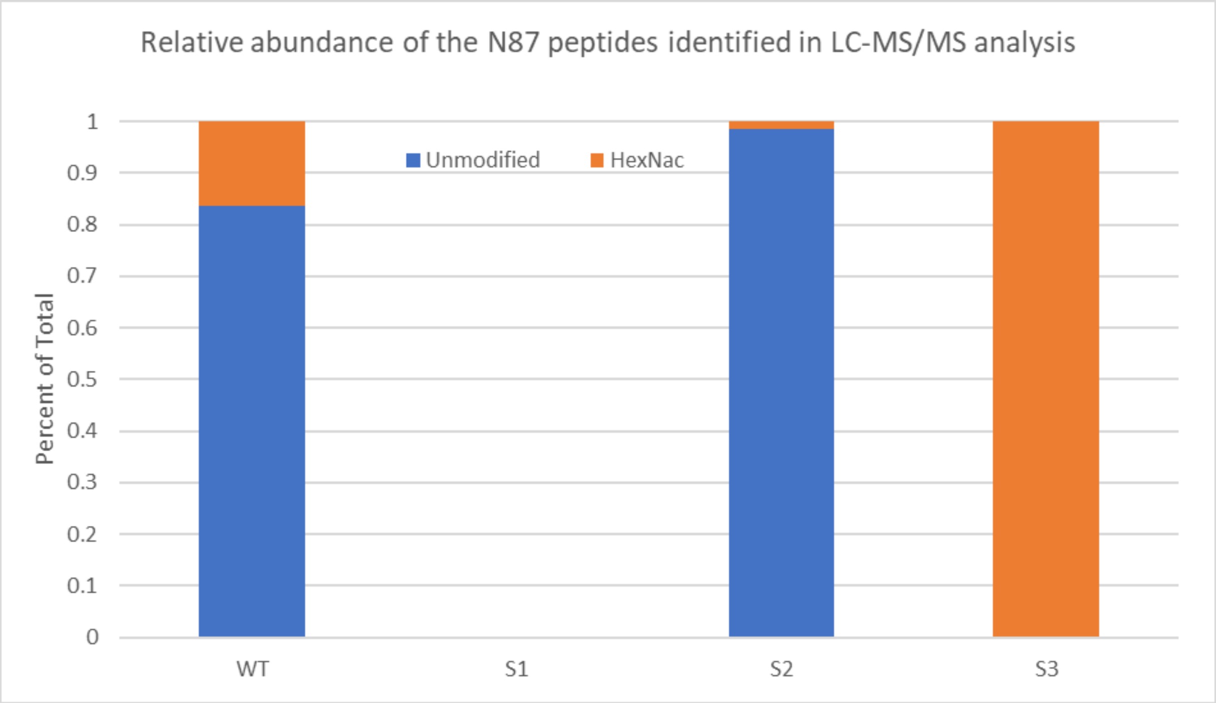

Conclusion

Four protein samples were submitted for in-solution digestion and LC-MS/MS analysis. These samples were digested with both trypsin and GluC and the LC-MS/MS data from these digests was searched against the sequences of Hemagglutinin submitted by the investigator. Hemagglutinin was identified in the tryptic digests by 42 to 86 peptides covering 53 to 82% of the protein sequences and by 123 to 328 GluC peptides covering 83 to 97% of the protein sequence. Chromatograms were plotted for three of the most abundant hemagglutinin peptides and the relative abundance of these peptides across these samples is given below.

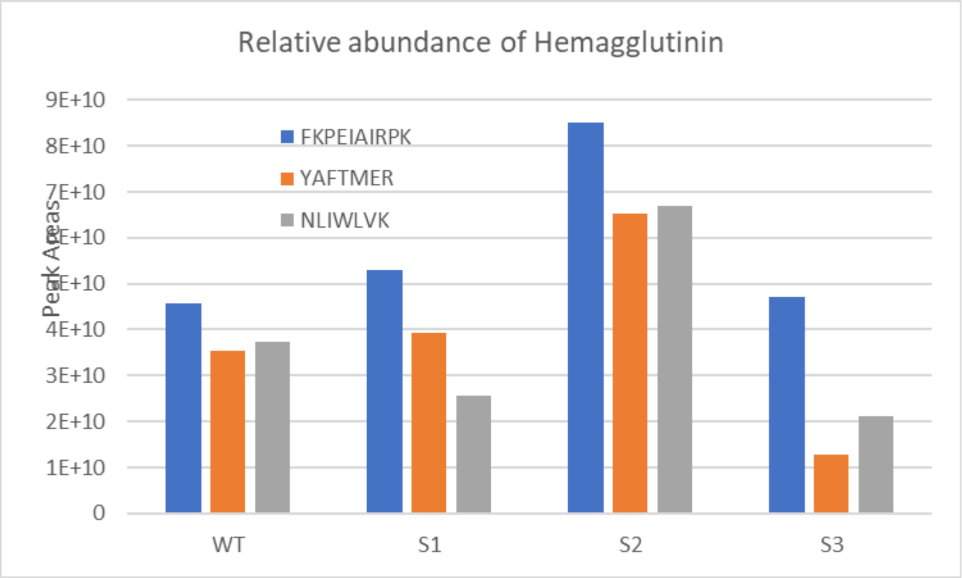

The chromatograms for all four tryptic digests were compared and three of these samples; WT, S1, and S2 appear very similar. The S3 sample contains several ions that are not identified or are lower abundant in the WT, S1, and S2 samples. Close examination of the MS/MS spectra for some of the abundant S3 peptides were manually interrogated and these peptides were mapped to hemagglutinin but were determined to be semi-tryptic peptides (formed by one tryptic specific cleavage event and one non-specific cleavage event). The data from all four tryptic digests were analyzed looking for semi-tryptic peptides and the S3 sample has the largest number of semi-tryptic peptides suggesting that this sample may have undergone a significant amount of proteolysis prior tryptic digestion.

The forms of Hemagglutinin analyzed in these samples differ in the Foldon region of the protein sequence. The coverage of this region in the four samples is summarzed below where regions identified by trypsin are given in red font, regions identified by GluC in purple font, and regions identified in both the trypsin and GluC digestions given in green font (**trypsin, GluC, trypsin and GluC**). The entire foldon sequence was identified in the WT sample while the R513N and G515N mutations were identified in S1 and S2 respectively. The E524N mutation in S3 was not identified in these searches. It is unclear whether this is due to the apparent protealysis of the protein prior to digestion, the lack of this mutation, or due to the poor performance of this region of the protein with trypsin and GluC digestion.

WT: GYIPEAPRDGQAYVRKDGEWVLLSTFL
S1: GYIPEAPNDTQAYVRKDGEWVLLSTFL
S2: GYIPEAPRDNQSYVRKDGEWVLLSTFL
S3: GYIPEAPRDGQAYVRKDGNWTLLSTFL

The goal of these experiments is to determine the glycosylation status for several sites including N11, N23, N87, N276, N287, N481, N513, and N515. The data from these samples was searched looking for glycosylation. Several glycosylated peptides were identified. For each of these peptides, single ion monitoring (SIM) chromatograms were plotted, these chromatograms were integrated, and the resulting peak areas were used to estimate the distribution of glycosylation across these samples. These results are summarized in Tables 4 to 12 in the attached spreadsheet.

Summary of total glycosylation sites identified in the LC-MS/MS analysis is given below.

	WT	S1	S2	S3
N11	15	0	15	2
N23	49	47	48	30
N87	16	14	16	17
N276	40	40	40	10
N287	1	0	1	1
N481	28	28	27	8
N513	0	17	0	0
N515	0	0	15	0

Table 2. Summary of results

Sample Name	Sample number	Protein	Accession	Gene	Mass kDa	Pep	Seq. Cov	Sequest Score
9.27.23 Y2-CHO WT Trypsin	SB20-89-33	Hemagglutinin Y2.CHO.wt - Full tryptic search	C3W5S1	HA	62.5	82	81	2432.6
		Hemagglutinin Y2.CHO.wt - Semi tryptic search				206	90	3783.9
9.27.23 Y2-CHO WT GluC	SB20-89-34	Hemagglutinin Y2.CHO.wt	C3W5S1	HA	62.5	153	93	2956.7
9.27.23 Y2-CHO S1 Trypsin	SB20-89-35	Hemagglutinin Y2.CHO.wt	C3W5S1	HA	62.5	64	62	2094.9
		Hemagglutinin Y2.CHO.wt - Semi tryptic search				228	90	4034.5
9.27.23 Y2-CHO S1 GluC	SB20-89-36	Hemagglutinin Y2.CHO.wt	C3W5S1	HA	62.5	123	8	2115.9
9.27.23 Y2-CHO S2 Trypsin	SB20-89-37	Hemagglutinin Y2.CHO.wt	C3W5S1	HA	62.5	86	82	2422.5
		Hemagglutinin Y2.CHO.wt - Semi tryptic search				175	86	3804
9.27.23 Y2-CHO S2 GluC	SB20-89-38	Hemagglutinin Y2.CHO.wt	C3W5S1	HA	62.5	197	97	3349
9.27.23 Y2-CHO S3 Trypsin	SB20-89-39	Hemagglutinin Y2.CHO.wt	C3W5S1	HA	62.5	42	53	768.6
		Hemagglutinin Y2.CHO.wt - Semi tryptic search				363	95	2128.6
9.27.23 Y2-CHO S3 GluC	SB20-89-40	Hemagglutinin Y2.CHO.wt	C3W5S1	HA	62.5	328	93	4303.9

Proteomics Sample Drop Off
Belinda Willard Ph.D.
willarb@ccf.org
216-339-9022 (cell)

Date: 8/28/23

Name: Spencer Pierce

Lab: Dr. Ted Ross

Number of Samples: 4

Sample type: In gel

On bead

In-solution

Pre-digested

Experiment: Protein ID

Quantitative Proteomics: Label free, SILAC, TMT

Modification Analysis:

Modifications of interest: Glycosylation at added point (see attached sequence)

Global or protein specific

IP experiment

Cross-linking MS

LC-MS/MS

Species: Influenza A Hemagglutinin

Special Instructions: Needed glycosylation confirmation

For Lab Use: vaccine antigen

Sample storage location: sending to Ted Ross Lab at Cleveland Clinic

```
Native N-linked glycosylation sites
Engineered N-linked glycosylation sites
Foldon

>Y2.CHO.wt
DTLCIGYHANNSTDTVDTVLEKNVTVTHSVNLLEDKHNGKLCKLRGVAPLHLGKCNIAGWILGN
PECESLSTASSWSYIVETSNSDNGTCYPGDFINYEELREQLSSVSSFERFEIFPKTSSWPNHDS
NKGVTAACPHAGAKSFYKNLIWLVKKGNSYPKLSQSYINDKGKEVLVLWGIHHPSTTADQQSLY
QNADAYVFVGTSRYSKKFKPEIAIRPKVRDQEGRMNYYWTLVEPGDKITFEATGNLVVPRYAFT
MERNAGSGIIISDTPVHDCNTTCQTPEGAINTSLPFQNVHPITIGKCPKYVKSTKLRLATGLRN
VPSIQSRGLFGAIAGFIEGGWTGMVDGWYGYHHQNEQGSGYAADLKSTQNAIDKITNKVNSVIE
KMNTQFTAVGKEFNHLEKRIENLNKKVDDGFLDIWTYNAELLVLLENERTLDYHDSNVKNLYEK
VRNQLKNNAKEIGNGCFEFYHKCDNTCMESVKNGTYDYPKYSEEAKLNREKIDGVGTGYIPEAP
RDGQAYVRKDGEWVLLSTFLGSGLNDIFEAQKIEWHEGHHHHHH*

>Y2.CHO.S1
DTLCIGYHANNSTDTVDTVLEKNVTVTHSVNLLEDKHNGKLCKLRGVAPLHLGKCNIAGWILGN
PECESLSTASSWSYIVETSNSDNGTCYPGDFINYEELREQLSSVSSFERFEIFPKTSSWPNHDS
NKGVTAACPHAGAKSFYKNLIWLVKKGNSYPKLSQSYINDKGKEVLVLWGIHHPSTTADQQSLY
QNADAYVFVGTSRYSKKFKPEIAIRPKVRDQEGRMNYYWTLVEPGDKITFEATGNLVVPRYAFT
MERNAGSGIIISDTPVHDCNTTCQTPEGAINTSLPFQNVHPITIGKCPKYVKSTKLRLATGLRN
VPSIQSRGLFGAIAGFIEGGWTGMVDGWYGYHHQNEQGSGYAADLKSTQNAIDKITNKVNSVIE
KMNTQFTAVGKEFNHLEKRIENLNKKVDDGFLDIWTYNAELLVLLENERTLDYHDSNVKNLYEK
VRNQLKNNAKEIGNGCFEFYHKCDNTCMESVKNGTYDYPKYSEEAKLNREKIDGVGTGYIPEAP
NDTQAYVRKDGEWVLLSTFLGSGLNDIFEAQKIEWHEGHHHHHH*

>Y2.CHO.S2
DTLCIGYHANNSTDTVDTVLEKNVTVTHSVNLLEDKHNGKLCKLRGVAPLHLGKCNIAGWILGN
PECESLSTASSWSYIVETSNSDNGTCYPGDFINYEELREQLSSVSSFERFEIFPKTSSWPNHDS
NKGVTAACPHAGAKSFYKNLIWLVKKGNSYPKLSQSYINDKGKEVLVLWGIHHPSTTADQQSLY
QNADAYVFVGTSRYSKKFKPEIAIRPKVRDQEGRMNYYWTLVEPGDKITFEATGNLVVPRYAFT
MERNAGSGIIISDTPVHDCNTTCQTPEGAINTSLPFQNVHPITIGKCPKYVKSTKLRLATGLRN
VPSIQSRGLFGAIAGFIEGGWTGMVDGWYGYHHQNEQGSGYAADLKSTQNAIDKITNKVNSVIE
KMNTQFTAVGKEFNHLEKRIENLNKKVDDGFLDIWTYNAELLVLLENERTLDYHDSNVKNLYEK
VRNQLKNNAKEIGNGCFEFYHKCDNTCMESVKNGTYDYPKYSEEAKLNREKIDGVGTGYIPEAP
RDNQSYVRKDGEWVLLSTFLGSGLNDIFEAQKIEWHEGHHHHHH*

>Y2.CHO.S3
DTLCIGYHANNSTDTVDTVLEKNVTVTHSVNLLEDKHNGKLCKLRGVAPLHLGKCNIAGWILGN
PECESLSTASSWSYIVETSNSDNGTCYPGDFINYEELREQLSSVSSFERFEIFPKTSSWPNHDS
NKGVTAACPHAGAKSFYKNLIWLVKKGNSYPKLSQSYINDKGKEVLVLWGIHHPSTTADQQSLY
QNADAYVFVGTSRYSKKFKPEIAIRPKVRDQEGRMNYYWTLVEPGDKITFEATGNLVVPRYAFT
MERNAGSGIIISDTPVHDCNTTCQTPEGAINTSLPFQNVHPITIGKCPKYVKSTKLRLATGLRN
VPSIQSRGLFGAIAGFIEGGWTGMVDGWYGYHHQNEQGSGYAADLKSTQNAIDKITNKVNSVIE
KMNTQFTAVGKEFNHLEKRIENLNKKVDDGFLDIWTYNAELLVLLENERTLDYHDSNVKNLYEK
VRNQLKNNAKEIGNGCFEFYHKCDNTCMESVKNGTYDYPKYSEEAKLNREKIDGVGTGYIPEAP
RDGQAYVRKDGNWTLLSTFLGSGLNDIFEAQKIEWHEGHHHHHH*
```

SB20-89-33: 9.27.23 in-soln sample Y2-CHO-WT trypsin

Protein MW: **62533**
Amino Acid Composition: **A29 C12 D28 E38 F19 G45 H22 I31 K42 L41 M5 N43 P21 Q16 R18 S38 T37 V34 W11 Y26**

```
1   DTLCIGYHAN NSTDTVDTVL EKNVTVTHSV NLLEDKHNGK LCKLRGVAPL HLGKCNIAGW ILGNPECESL STASSWSYIV
81  ETSNSDNGTC YPGDFINYEE LREQLSSVSS FERFEIFPKT SSWPNHDSNK GVTAACPHAG AKSFYKNLIW LVKKGNSYPK
161 LSQSYINDKG KEVLVLWGIH HPSTTADQQS LYQNADAYVF VGTSRYSKKF KPEIAIRPKV RDQEGRMNYY WTLVEPGDKI
241 TFEATGNLVV PRYAFTMERN AGSGIIISDT PVHDCNTTCQ TPEGAINTSL PFQNVHPITI GKCPKYVKST KLRLATGLRN
321 VPSIQSRGLF GAIAGFIEGG WTGMVDGWYG YHHQNEQGSG YAADLKSTQN AIDKITNKVN SVIEKMNTQF TAVGKEFNHL
401 EKRIENLNKK VDDGFLDIWT YNAELLVLLE NERTLDYHDS NVKNLYEKVR NQLKNNAKEI GNGCFEFYHK CDNTCMESVK
481 NGTYDYPKYS EEAKLNREKI DGVGTGYIPE APRDGQAYVR KDGEWVLLST FLGSGLNDIF EAQKIEWHEG HHHHHH
```

SB20-89-34: 9.27.23 in-soln sample Y2-CHO-WT GluC

Protein MW: **62533**
Amino Acid Composition: **A29 C12 D28 E38 F19 G45 H22 I31 K42 L41 M5 N43 P21 Q16 R18 S38 T37 V34 W11 Y26**

```
1   DTLCIGYHAN NSTDTVDTVL EKNVTVTHSV NLLEDKHNGK LCKLRGVAPL HLGKCNIAGW ILGNPECESL STASSWSYIV
81  ETSNSDNGTC YPGDFINYEE LREQLSSVSS FERFEIFPKT SSWPNHDSNK GVTAACPHAG AKSFYKNLIW LVKKGNSYPK
161 LSQSYINDKG KEVLVLWGIH HPSTTADQQS LYQNADAYVF VGTSRYSKKF KPEIAIRPKV RDQEGRMNYY WTLVEPGDKI
241 TFEATGNLVV PRYAFTMERN AGSGIIISDT PVHDCNTTCQ TPEGAINTSL PFQNVHPITI GKCPKYVKST KLRLATGLRN
321 VPSIQSRGLF GAIAGFIEGG WTGMVDGWYG YHHQNEQGSG YAADLKSTQN AIDKITNKVN SVIEKMNTQF TAVGKEFNHL
401 EKRIENLNKK VDDGFLDIWT YNAELLVLLE NERTLDYHDS NVKNLYEKVR NQLKNNAKEI GNGCFEFYHK CDNTCMESVK
481 NGTYDYPKYS EEAKLNREKI DGVGTGYIPE APRDGQAYVR KDGEWVLLST FLGSGLNDIF EAQKIEWHEG HHHHHH
```

SB20-89-35: 9.27.23 in-soln sample Y2-CHO-S1 trypsin

Protein MW: **62535**
Amino Acid Composition: **A29 C12 D28 E38 F19 G44 H22 I31 K42 L41 M5 N44 P21 Q16 R17 S38 T38 V34 W11 Y26**

```
1   DTLCIGYHAN NSTDTVDTVL EKNVTVTHSV NLLEDKHNGK LCKLRGVAPL HLGKCNIAGW ILGNPECESL STASSWSYIV
81  ETSNSDNGTC YPGDFINYEE LREQLSSVSS FERFEIFPKT SSWPNHDSNK GVTAACPHAG AKSFYKNLIW LVKKGNSYPK
161 LSQSYINDKG KEVLVLWGIH HPSTTADQQS LYQNADAYVF VGTSRYSKKF KPEIAIRPKV RDQEGRMNYY WTLVEPGDKI
241 TFEATGNLVV PRYAFTMERN AGSGIIISDT PVHDCNTTCQ TPEGAINTSL PFQNVHPITI GKCPKYVKST KLRLATGLRN
321 VPSIQSRGLF GAIAGFIEGG WTGMVDGWYG YHHQNEQGSG YAADLKSTQN AIDKITNKVN SVIEKMNTQF TAVGKEFNHL
401 EKRIENLNKK VDDGFLDIWT YNAELLVLLE NERTLDYHDS NVKNLYEKVR NQLKNNAKEI GNGCFEFYHK CDNTCMESVK
481 NGTYDYPKYS EEAKLNREKI DGVGTGYIPE APNDTQAYVR KDGEWVLLST FLGSGLNDIF EAQKIEWHEG HHHHHH
```

SB20-89-36: 9.27.23 in-soln sample Y2-CHO-S1 GluC

Protein MW: **62535**
Amino Acid Composition: **A29 C12 D28 E38 F19 G44 H22 I31 K42 L41 M5 N44 P21 Q16 R17 S38 T38 V34 W11 Y26**

```
1   DTLCIGYHAN NSTDTVDTVL EKNVTVTHSV NLLEDKHNGK LCKLRGVAPL HLGKCNIAGW ILGNPECESL STASSWSYIV
81  ETSNSDNGTC YPGDFINYEE LREQLSSVSS FERFEIFPKT SSWPNHDSNK GVTAACPHAG AKSFYKNLIW LVKKGNSYPK
161 LSQSYINDKG KEVLVLWGIH HPSTTADQQS LYQNADAYVF VGTSRYSKKF KPEIAIRPKV RDQEGRMNYY WTLVEPGDKI
241 TFEATGNLVV PRYAFTMERN AGSGIIISDT PVHDCNTTCQ TPEGAINTSL PFQNVHPITI GKCPKYVKST KLRLATGLRN
321 VPSIQSRGLF GAIAGFIEGG WTGMVDGWYG YHHQNEQGSG YAADLKSTQN AIDKITNKVN SVIEKMNTQF TAVGKEFNHL
401 EKRIENLNKK VDDGFLDIWT YNAELLVLLE NERTLDYHDS NVKNLYEKVR NQLKNNAKEI GNGCFEFYHK CDNTCMESVK
481 NGTYDYPKYS EEAKLNREKI DGVGTGYIPE APNDTQAYVR KDGEWVLLST FLGSGLNDIF EAQKIEWHEG HHHHHH
```

SB20-89-37: 9.27.23 in-soln sample Y2-CHO-S2 trypsin

Protein MW: **62606**
Amino Acid Composition: **A28 C12 D28 E38 F19 G44 H22 I31 K42 L41 M5 N44 P21 Q16 R18 S39 T37 V34 W11 Y26**

```
1   DTLCIGYHAN NSTDTVDTVL EKNVTVTHSV NLLEDKHNGK LCKLRGVAPL HLGKCNIAGW ILGNPECESL STASSWSYIV
81  ETSNSDNGTC YPGDFINYEE LREQLSSVSS FERFEIFPKT SSWPNHDSNK GVTAACPHAG AKSFYKNLIW LVKKGNSYPK
161 LSQSYINDKG KEVLVLWGIH HPSTTADQQS LYQNADAYVF VGTSRYSKKF KPEIAIRPKV RDQEGRMNYY WTLVEPGDKI
241 TFEATGNLVV PRYAFTMERN AGSGIIISDT PVHDCNTTCQ TPEGAINTSL PFQNVHPITI GKCPKYVKST KLRLATGLRN
321 VPSIQSRGLF GAIAGFIEGG WTGMVDGWYG YHHQNEQGSG YAADLKSTQN AIDKITNKVN SVIEKMNTQF TAVGKEFNHL
401 EKRIENLNKK VDDGFLDIWT YNAELLVLLE NERTLDYHDS NVKNLYEKVR NQLKNNAKEI GNGCFEFYHK CDNTCMESVK
481 NGTYDYPKYS EEAKLNREKI DGVGTGYIPE APRDNQSYVR KDGEWVLLST FLGSGLNDIF EAQKIEWHEG HHHHHH
```

SB20-89-38: 9.27.23 in-soln sample Y2-CHO-S2 GluC

Protein MW: **62606**
Amino Acid Composition: **A28 C12 D28 E38 F19 G44 H22 I31 K42 L41 M5 N44 P21 Q16 R18 S39 T37 V34 W11 Y26**

```
1   DTLCIGYHAN NSTDTVDTVL EKNVTVTHSV NLLEDKHNGK LCKLRGVAPL HLGKCNIAGW ILGNPECESL STASSWSYIV
81  ETSNSDNGTC YPGDFINYEE LREQLSSVSS FERFEIFPKT SSWPNHDSNK GVTAACPHAG AKSFYKNLIW LVKKGNSYPK
161 LSQSYINDKG KEVLVLWGIH HPSTTADQQS LYQNADAYVF VGTSRYSKKF KPEIAIRPKV RDQEGRMNYY WTLVEPGDKI
241 TFEATGNLVV PRYAFTMERN AGSGIIISDT PVHDCNTTCQ TPEGAINTSL PFQNVHPITI GKCPKYVKST KLRLATGLRN
321 VPSIQSRGLF GAIAGFIEGG WTGMVDGWYG YHHQNEQGSG YAADLKSTQN AIDKITNKVN SVIEKMNTQF TAVGKEFNHL
401 EKRIENLNKK VDDGFLDIWT YNAELLVLLE NERTLDYHDS NVKNLYEKVR NQLKNNAKEI GNGCFEFYHK CDNTCMESVK
481 NGTYDYPKYS EEAKLNREKI DGVGTGYIPE APRDNQSYVR KDGEWVLLST FLGSGLNDIF EAQKIEWHEG HHHHHH
```

SB20-89-39: 9.27.23 in-soln sample Y2-CHO-S3 trypsin

Protein MW: **62520**
Amino Acid Composition: **A29 C12 D28 E37 F19 G45 H22 I31 K42 L41 M5 N44 P21 Q16 R18 S38 T38 V33 W11 Y26**

```
1   DTLCIGYHAN NSTDTVDTVL EKNVTVTHSV NLLEDKHNGK LCKLRGVAPL HLGKCNIAGW ILGNPECESL STASSWSYIV
81  ETSNSDNGTC YPGDFINYEE LREQLSSVSS FERFEIFPKT SSWPNHDSNK GVTAACPHAG AKSFYKNLIW LVKKGNSYPK
161 LSQSYINDKG KEVLVLWGIH HPSTTADQQS LYQNADAYVF VGTSRYSKKF KPEIAIRPKV RDQEGRMNYY WTLVEPGDKI
241 TFEATGNLVV PRYAFTMERN AGSGIIISDT PVHDCNTTCQ TPEGAINTSL PFQNVHPITI GKCPKYVKST KLRLATGLRN
321 VPSIQSRGLF GAIAGFIEGG WTGMVDGWYG YHHQNEQGSG YAADLKSTQN AIDKITNKVN SVIEKMNTQF TAVGKEFNHL
401 EKRIENLNKK VDDGFLDIWT YNAELLVLLE NERTLDYHDS NVKNLYEKVR NQLKNNAKEI GNGCFEFYHK CDNTCMESVK
481 NGTYDYPKYS EEAKLNREKI DGVGTGYIPE APRDGQAYVR KDGNWTLLST FLGSGLNDIF EAQKIEWHEG HHHHHH
```

SB20-89-40: 9.27.23 in-soln sample Y2-CHO-S3 GluC

Protein MW: **62520**
Amino Acid Composition: **A29 C12 D28 E37 F19 G45 H22 I31 K42 L41 M5 N44 P21 Q16 R18 S38 T38 V33 W11 Y26**

```
1   DTLCIGYHAN NSTDTVDTVL EKNVTVTHSV NLLEDKHNGK LCKLRGVAPL HLGKCNIAGW ILGNPECESL STASSWSYIV
81  ETSNSDNGTC YPGDFINYEE LREQLSSVSS FERFEIFPKT SSWPNHDSNK GVTAACPHAG AKSFYKNLIW LVKKGNSYPK
161 LSQSYINDKG KEVLVLWGIH HPSTTADQQS LYQNADAYVF VGTSRYSKKF KPEIAIRPKV RDQEGRMNYY WTLVEPGDKI
241 TFEATGNLVV PRYAFTMERN AGSGIIISDT PVHDCNTTCQ TPEGAINTSL PFQNVHPITI GKCPKYVKST KLRLATGLRN
321 VPSIQSRGLF GAIAGFIEGG WTGMVDGWYG YHHQNEQGSG YAADLKSTQN AIDKITNKVN SVIEKMNTQF TAVGKEFNHL
401 EKRIENLNKK VDDGFLDIWT YNAELLVLLE NERTLDYHDS NVKNLYEKVR NQLKNNAKEI GNGCFEFYHK CDNTCMESVK
481 NGTYDYPKYS EEAKLNREKI DGVGTGYIPE APRDGQAYVR KDGNWTLLST FLGSGLNDIF EAQKIEWHEG HHHHHH
```

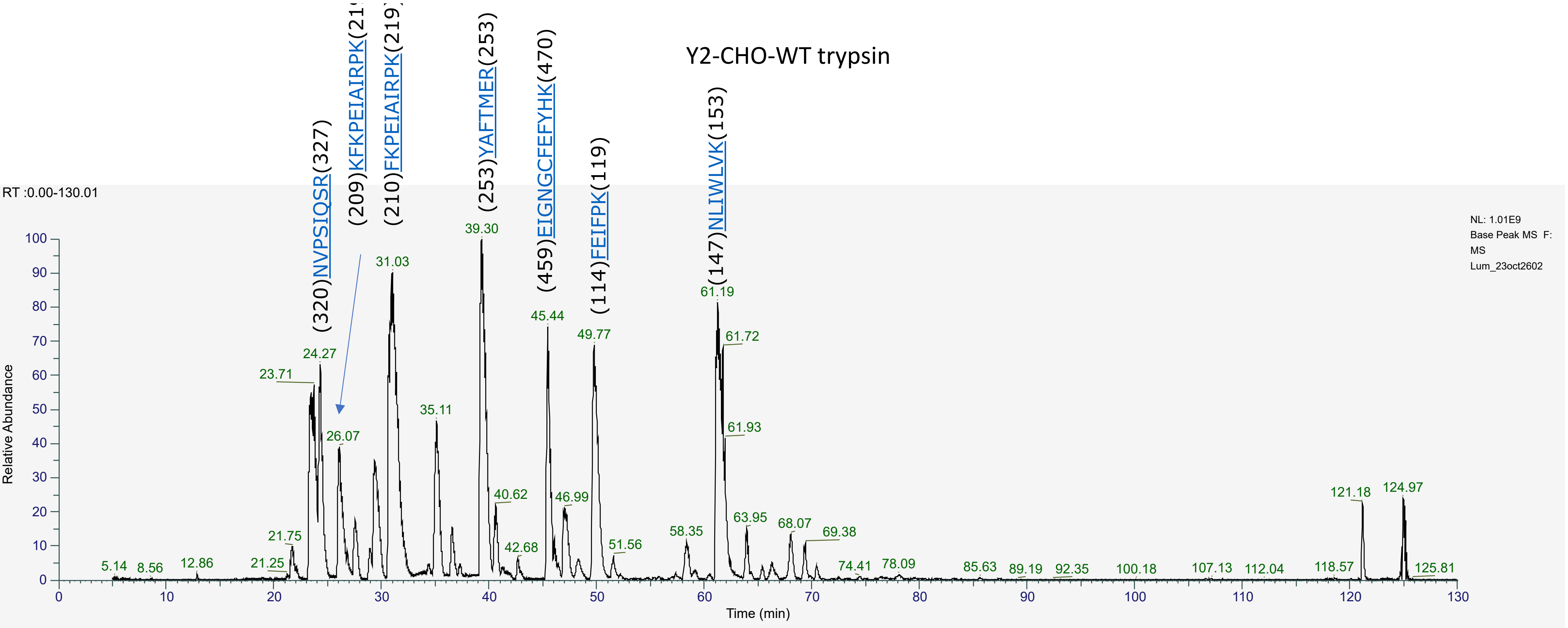

Y2-CHO-WT trypsin
RT :0.00-130.01
NL: 1.01E9
Base Peak MS F: MS
Lum_23oct2602
Relative Abundance
Time (min)
(320)NVPSIQSR(327)
(209)KFKPEIAIRPK(21
(210)FKPEIAIRPK(219
(253)YAFTMER(253)
(459)EIGNGCFEFYHK(470)
(114)FEIFPK(119)
(147)NLIWLVK(153)
5.14
8.56
12.86
21.25
21.75
23.71
24.27
26.07
31.03
35.11
39.30
40.62
42.68
45.44
46.99
49.77
51.56
58.35
61.19
61.72
61.93
63.95
68.07
69.38
74.41
78.09
85.63
89.19
92.35
100.18
107.13
112.04
118.57
121.18
124.97
125.81

Y2-CHO-S1 trypsin

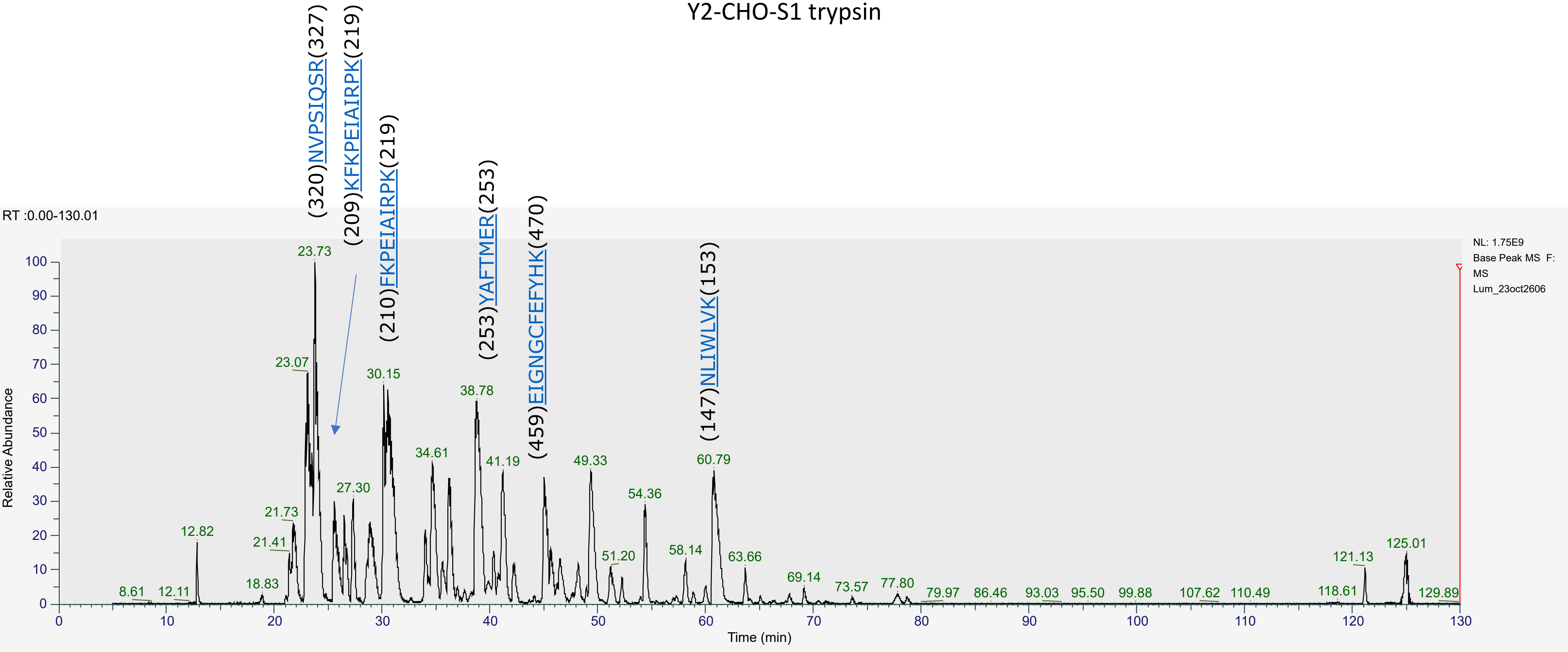

Y2-CHO-S2 trypsin

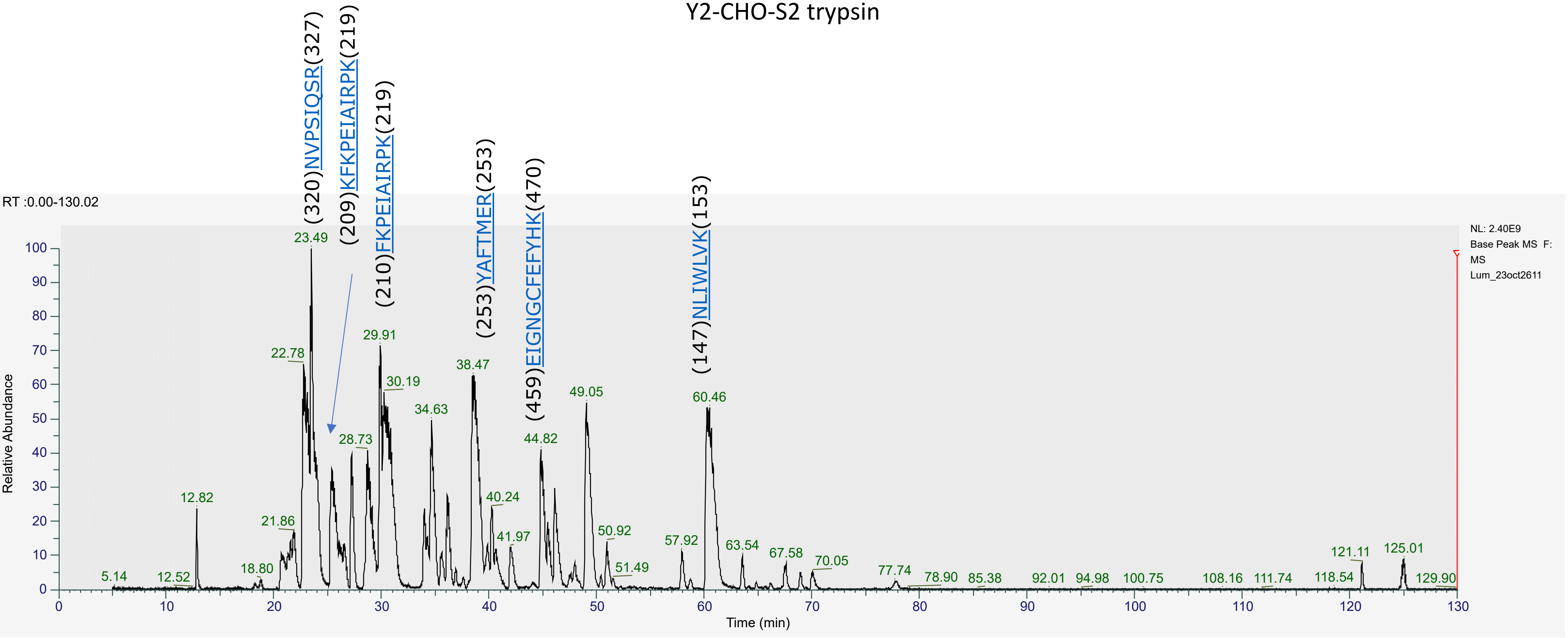

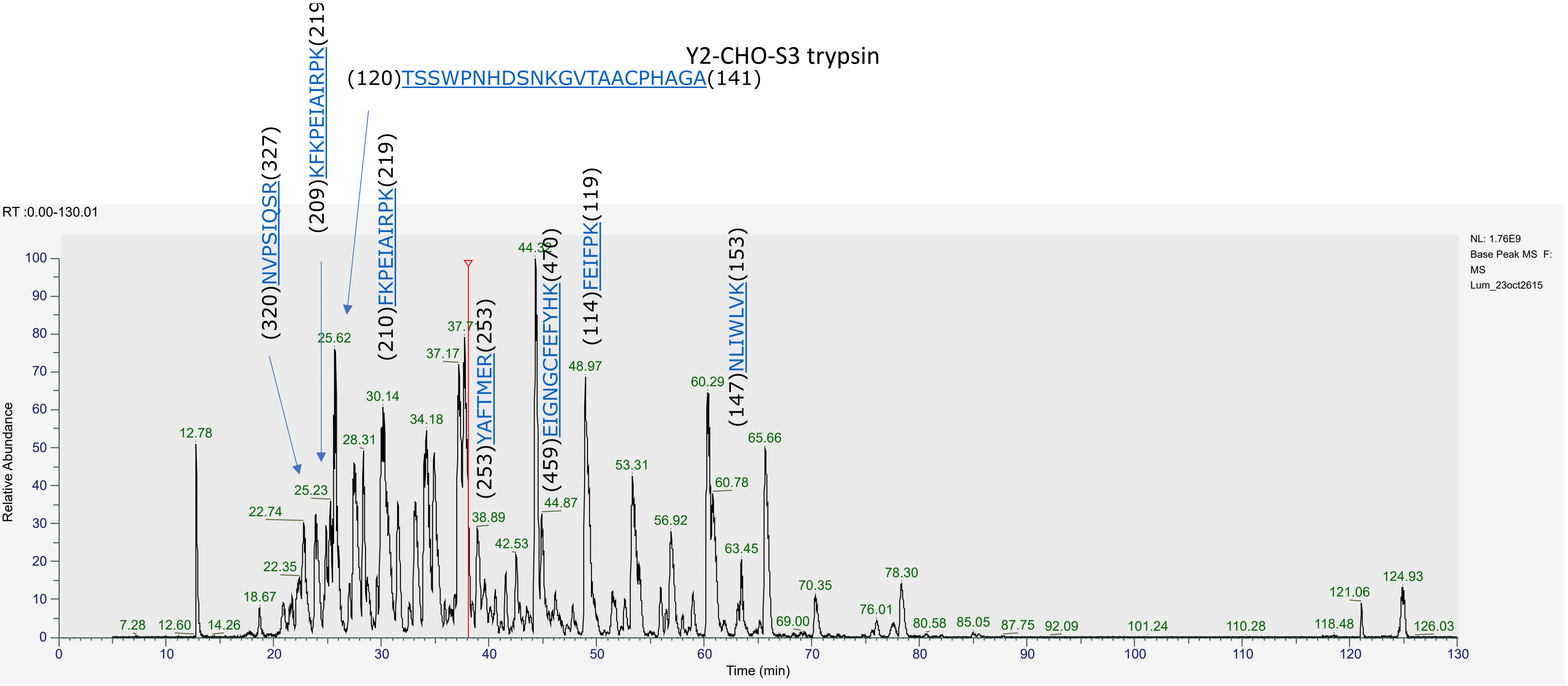

Y2-CHO-S3 trypsin
(120)TSSWPNHDSNKGVTAACPHAGA(141)
(320)NVPSIQSR(327)
(209)KFKPEIAIRPK(219
(210)FKPEIAIRPK(219)
(253)YAFTMER(253)
(459)EIGNGCFEFYHK(470)
(114)FEIFPK(119)
(147)NLIWLVK(153)
RT :0.00-130.01
NL: 1.76E9
Base Peak MS F:
MS
Lum_23oct2615
Relative Abundance
Time (min)
12.78
25.62
30.14
37.71
44.32
48.97
60.29
65.66

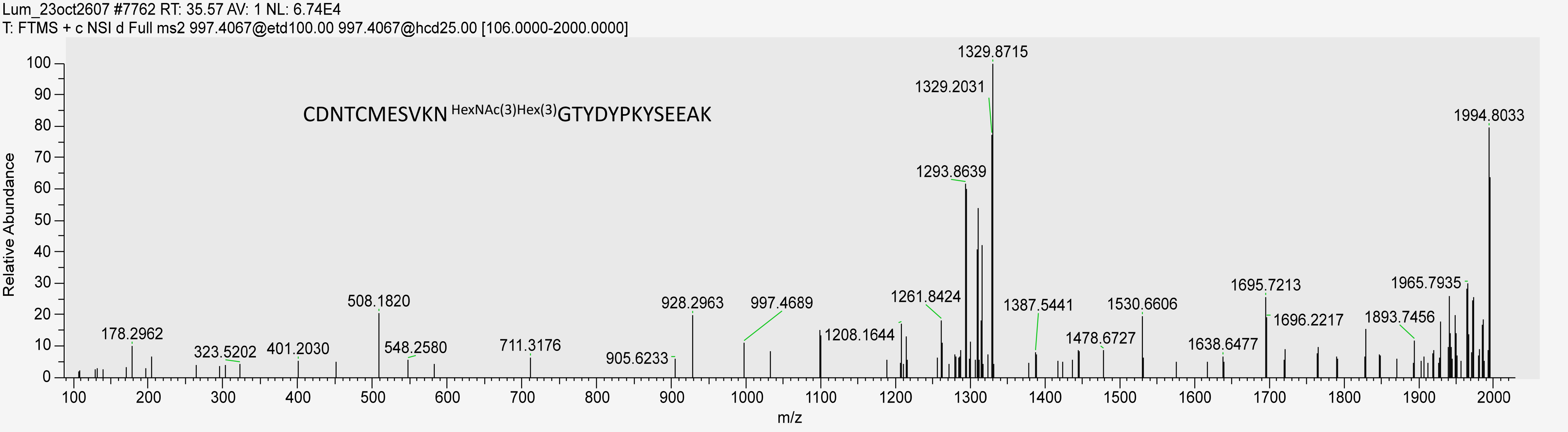
Lum_23oct2607 #7762 RT: 35.57 AV: 1 NL: 6.74E4
T: FTMS + c NSI d Full ms2 997.4067@etd100.00 997.4067@hcd25.00 [106.0000-2000.0000]
CDNTCMESVKN HexNAc(3)Hex(3)GTYDYPKYSEEAK
Relative Abundance
m/z
1329.8715
1329.2031
1994.8033
1293.8639
1261.8424
1387.5441
1530.6606
1695.7213
1965.7935
1696.2217
1893.7456
178.2962
323.5202
401.2030
508.1820
548.2580
711.3176
905.6233
928.2963
997.4689
1208.1644
1478.6727
1638.6477

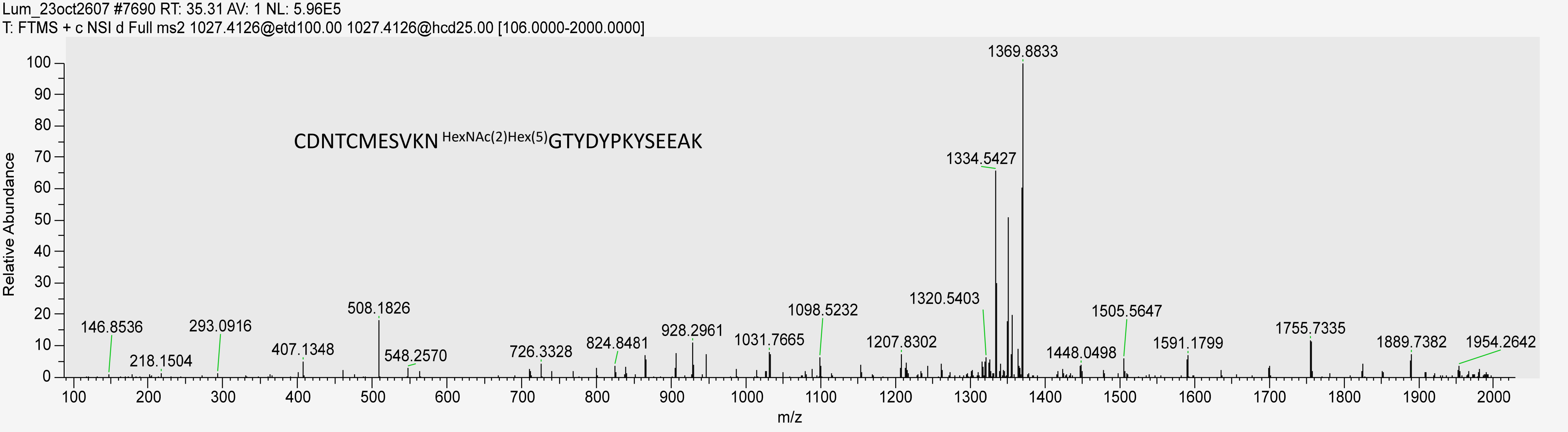
Lum_23oct2607 #7690 RT: 35.31 AV: 1 NL: 5.96E5
T: FTMS + c NSI d Full ms2 1027.4126@etd100.00 1027.4126@hcd25.00 [106.0000-2000.0000]
CDNTCMESVKN HexNAc(2)Hex(5)GTYDYPKYSEEAK
Relative Abundance
m/z
1369.8833
1334.5427
1320.5403
1505.5647
1755.7335
1098.5232
146.8536
293.0916
508.1826
218.1504
407.1348
548.2570
726.3328
824.8481
928.2961
1031.7665
1207.8302
1448.0498
1591.1799
1889.7382
1954.2642

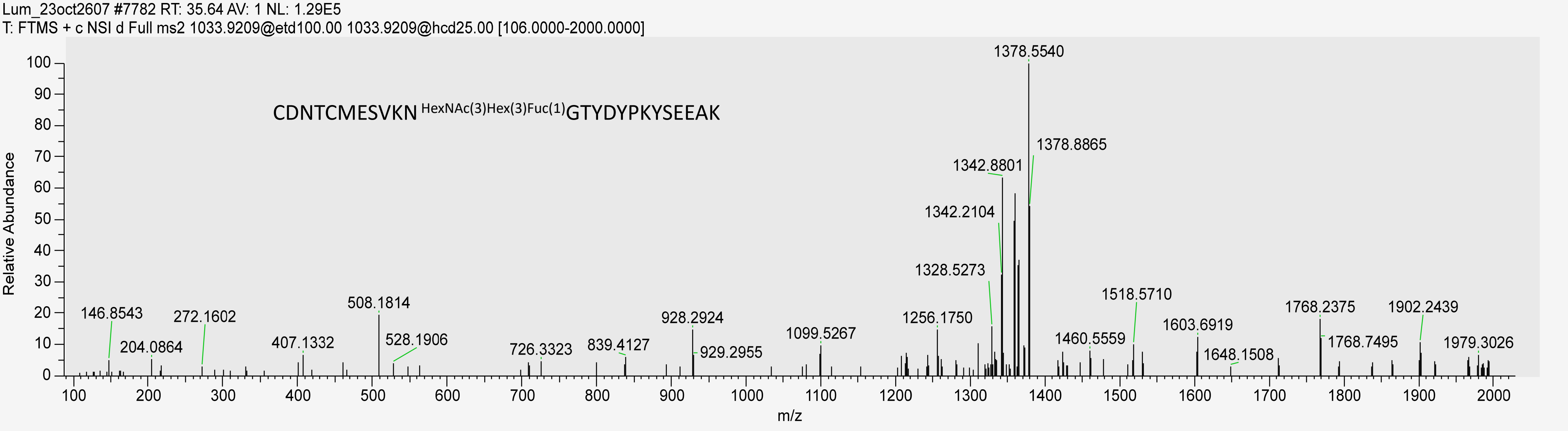
Lum_23oct2607 #7782 RT: 35.64 AV: 1 NL: 1.29E5
T: FTMS + c NSI d Full ms2 1033.9209@etd100.00 1033.9209@hcd25.00 [106.0000-2000.0000]
CDNTCMESVKN HexNAc(3)Hex(3)Fuc(1)GTYDYPKYSEEAK
Relative Abundance
m/z
1378.5540
1378.8865
1342.8801
1342.2104
1328.5273
1518.5710
1256.1750
1768.2375
1902.2439
1603.6919
146.8543
272.1602
508.1814
928.2924
204.0864
407.1332
528.1906
726.3323
839.4127
929.2955
1099.5267
1460.5559
1648.1508
1768.7495
1979.3026

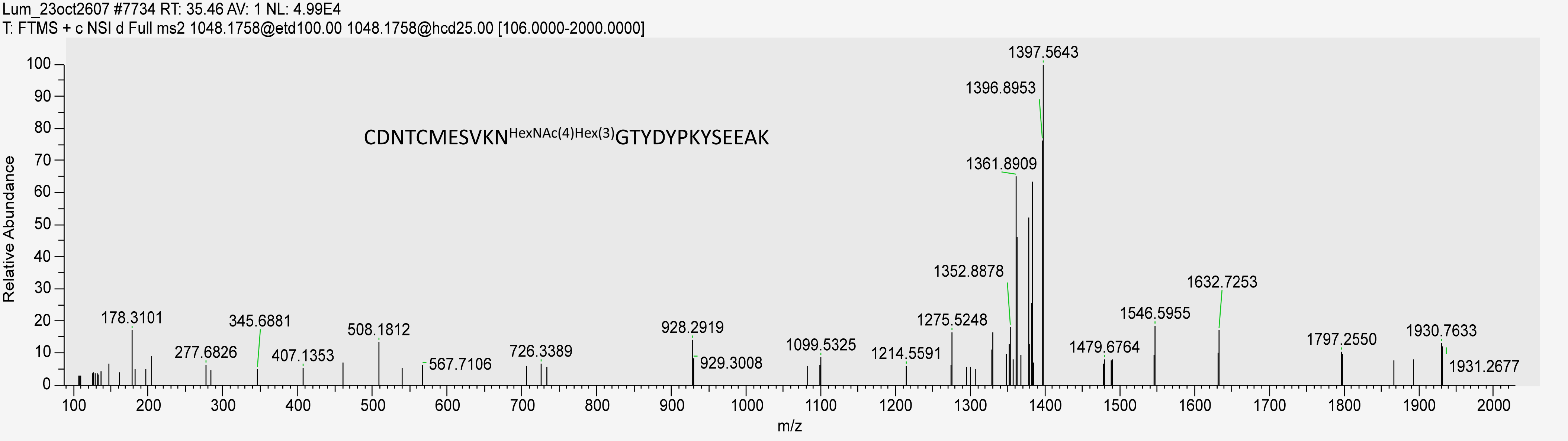
Lum_23oct2607 #7734 RT: 35.46 AV: 1 NL: 4.99E4
T: FTMS + c NSI d Full ms2 1048.1758@etd100.00 1048.1758@hcd25.00 [106.0000-2000.0000]
CDNTCMESVKNHexNAc(4)Hex(3)GTYDYPKYSEEAK
Relative Abundance
m/z
1397.5643
1396.8953
1361.8909
1352.8878
1632.7253
1546.5955
1275.5248
178.3101
345.6881
508.1812
928.2919
1930.7633
1797.2550
277.6826
407.1353
726.3389
1099.5325
1214.5591
1479.6764
567.7106
929.3008
1931.2677

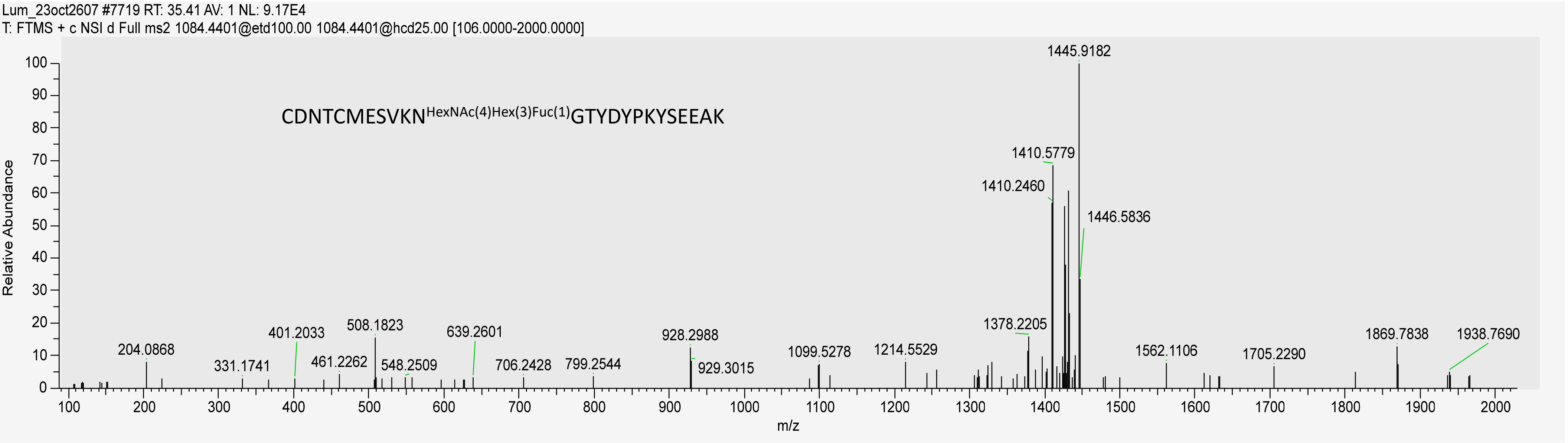
Lum_23oct2607 #7719 RT: 35.41 AV: 1 NL: 9.17E4
T: FTMS + c NSI d Full ms2 1084.4401@etd100.00 1084.4401@hcd25.00 [106.0000-2000.0000]
CDNTCMESVKN HexNAc(4)Hex(3)Fuc(1) GTYDYPKYSEEAK
Relative Abundance
m/z
1445.9182
1410.5779
1410.2460
1446.5836
1378.2205
204.0868
331.1741
401.2033
461.2262
508.1823
548.2509
639.2601
706.2428
799.2544
928.2988
929.3015
1099.5278
1214.5529
1562.1106
1705.2290
1869.7838
1938.7690

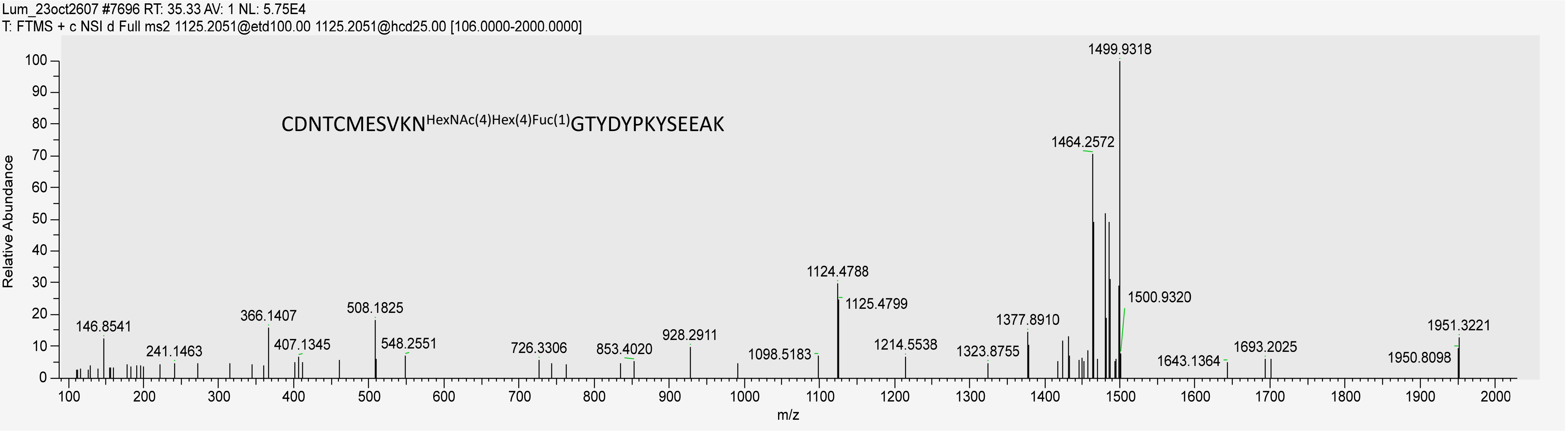
Lum_23oct2607 #7696 RT: 35.33 AV: 1 NL: 5.75E4
T: FTMS + c NSI d Full ms2 1125.2051@etd100.00 1125.2051@hcd25.00 [106.0000-2000.0000]
CDNTCMESVKN HexNAc(4)Hex(4)Fuc(1) GTYDYPKYSEEAK
Relative Abundance
m/z
1499.9318
1464.2572
1124.4788
1125.4799
1500.9320
146.8541
241.1463
366.1407
407.1345
508.1825
548.2551
726.3306
853.4020
928.2911
1098.5183
1214.5538
1323.8755
1377.8910
1643.1364
1693.2025
1950.8098
1951.3221

Lum_23oct2607 #7699 RT: 35.34 AV: 1 NL: 1.08E5
T: FTMS + c NSI d Full ms2 1135.7109@etd100.00 1135.7109@hcd25.00 [106.0000-2000.0000]

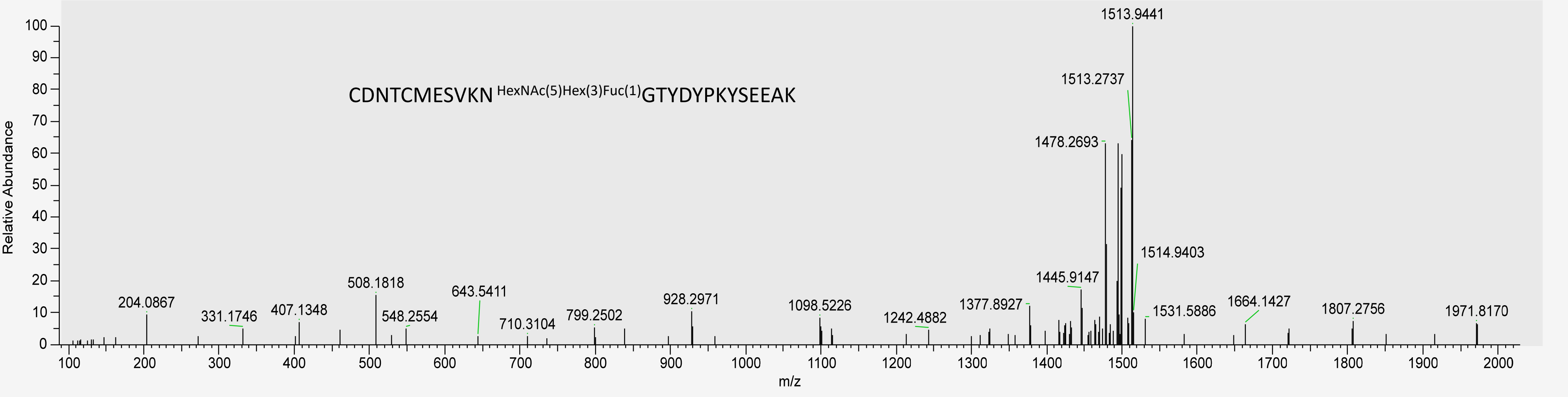

Lum_23oct2607 #7652 RT: 35.17 AV: 1 NL: 4.49E4
T: FTMS + c NSI d Full ms2 1165.7167@etd100.00 1165.7167@hcd25.00 [106.0000-2000.0000]

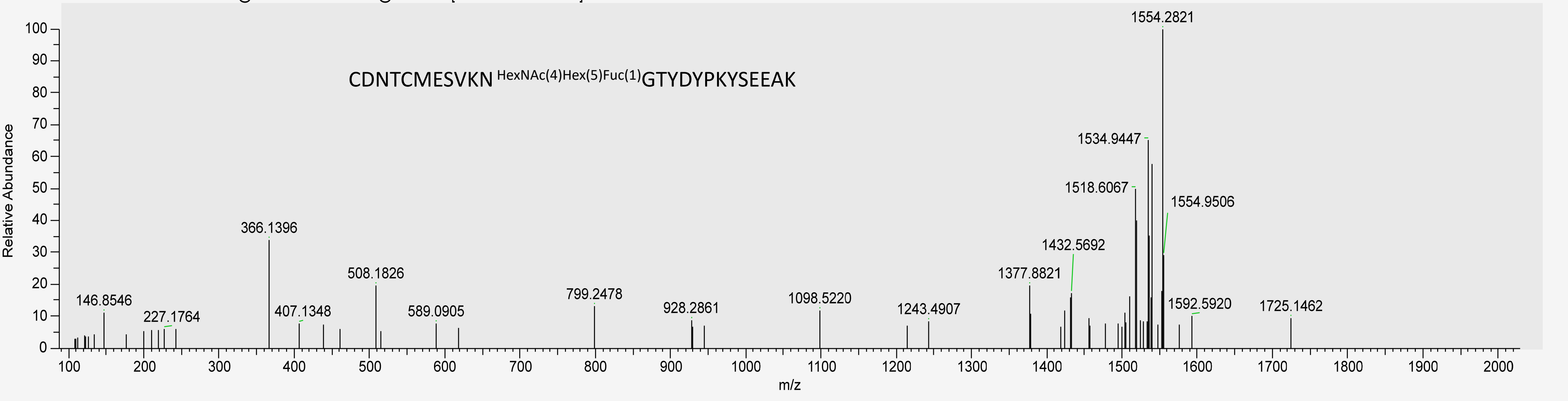

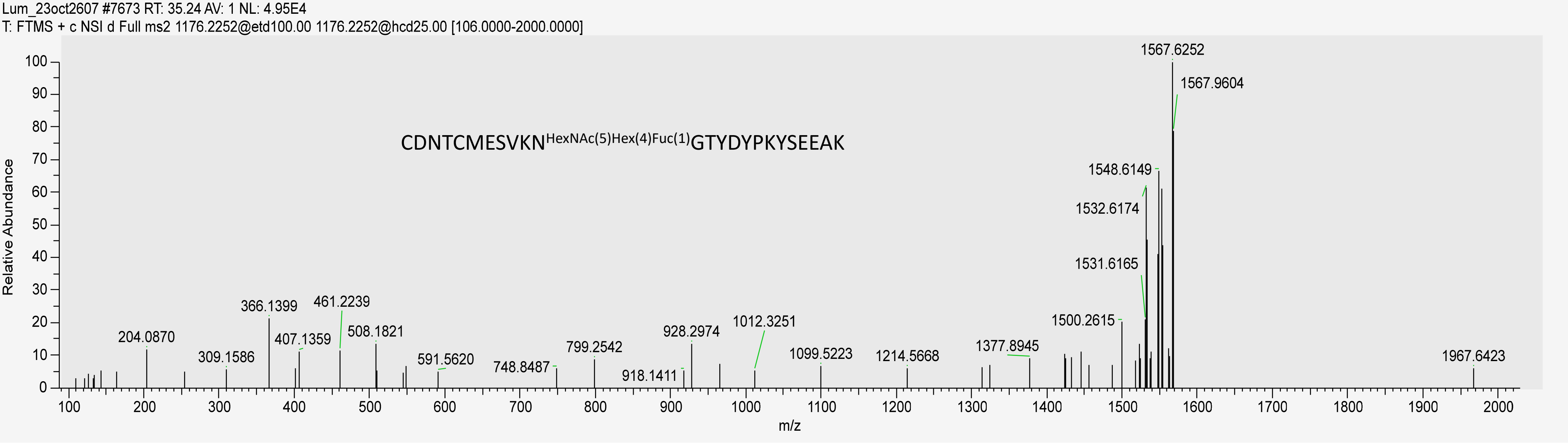
Lum_23oct2607 #7673 RT: 35.24 AV: 1 NL: 4.95E4
T: FTMS + c NSI d Full ms2 1176.2252@etd100.00 1176.2252@hcd25.00 [106.0000-2000.0000]
CDNTCMESVKN HexNAc(5)Hex(4)Fuc(1) GTYDYPKYSEEAK
Relative Abundance
m/z
1567.6252
1567.9604
1548.6149
1532.6174
1531.6165
1500.2615
1377.8945
1214.5668
1099.5223
1012.3251
928.2974
918.1411
799.2542
748.8487
591.5620
508.1821
461.2239
407.1359
366.1399
309.1586
204.0870
1967.6423

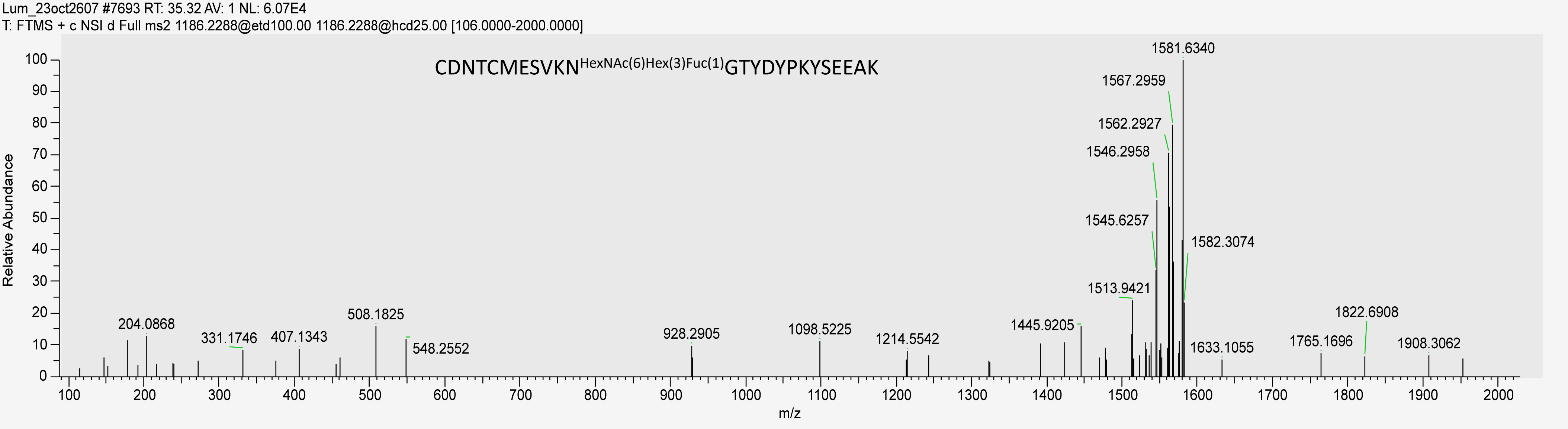
Lum_23oct2607 #7693 RT: 35.32 AV: 1 NL: 6.07E4
T: FTMS + c NSI d Full ms2 1186.2288@etd100.00 1186.2288@hcd25.00 [106.0000-2000.0000]
CDNTCMESVKN HexNAc(6)Hex(3)Fuc(1) GTYDYPKYSEEAK
Relative Abundance
m/z
1581.6340
1567.2959
1562.2927
1546.2958
1545.6257
1582.3074
1513.9421
1445.9205
1214.5542
1098.5225
928.2905
548.2552
508.1825
407.1343
331.1746
204.0868
1633.1055
1765.1696
1822.6908
1908.3062

Lum_23oct2607 #8650 RT: 38.76 AV: 1 NL: 4.39E4
T: FTMS + c NSI d Full ms2 1198.2288@etd100.00 1198.2288@hcd25.00 [106.0000-2000.0000]

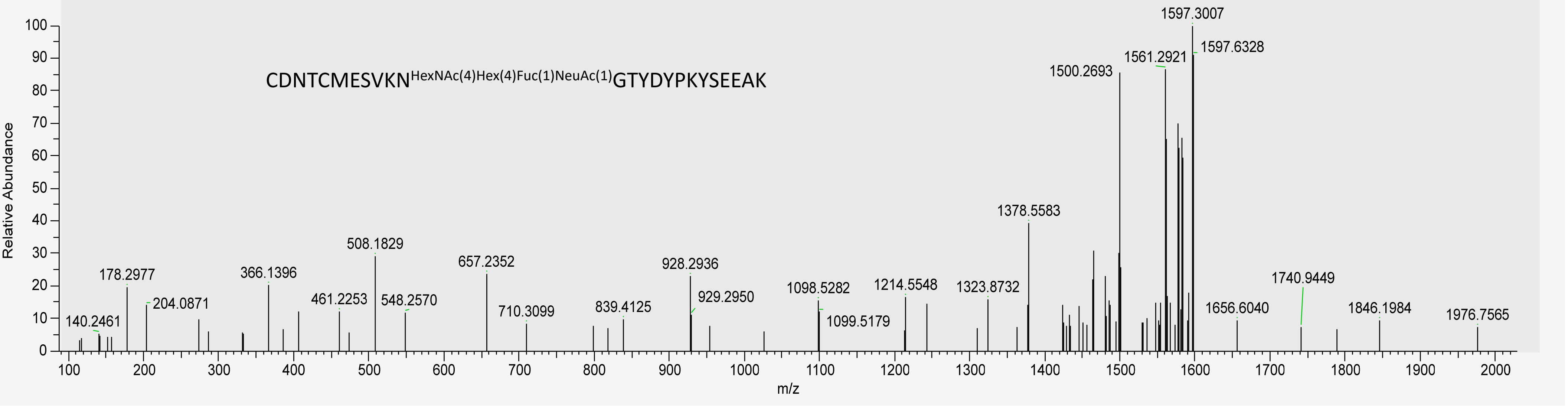

Lum_23oct2607 #8605 RT: 38.60 AV: 1 NL: 7.22E4
T: FTMS + c NSI d Full ms2 1202.2280@etd100.00 1202.2280@hcd25.00 [106.0000-2000.0000]

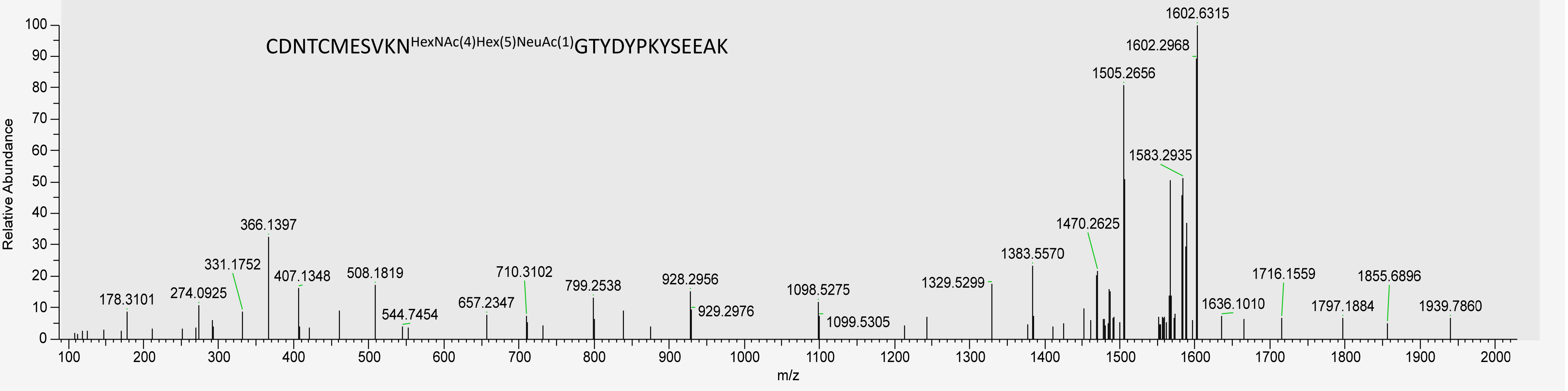

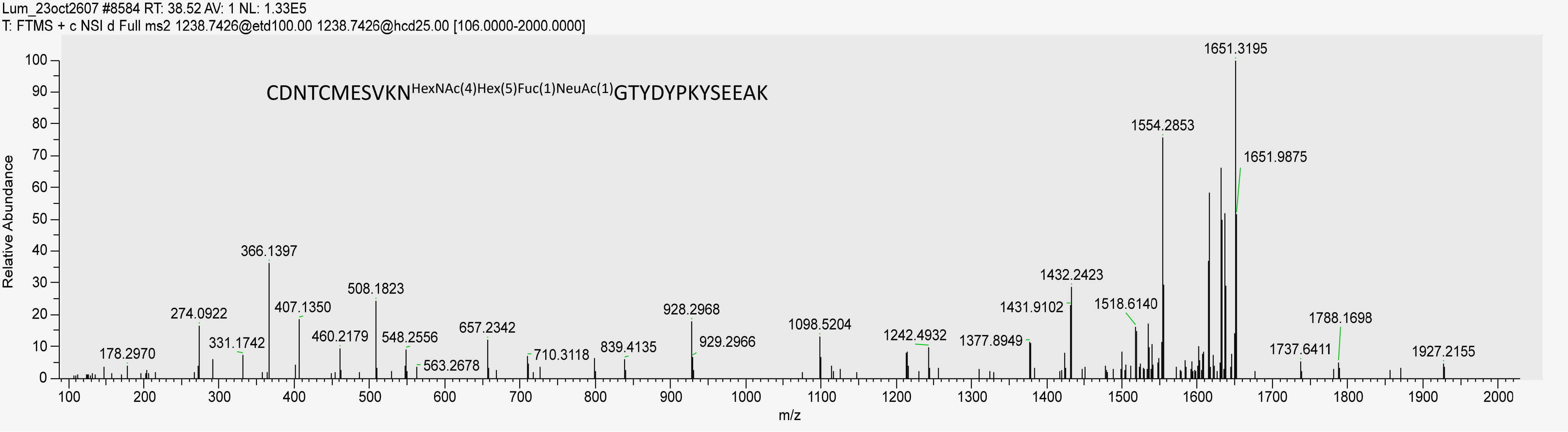
Lum_23oct2607 #8584 RT: 38.52 AV: 1 NL: 1.33E5
T: FTMS + c NSI d Full ms2 1238.7426@etd100.00 1238.7426@hcd25.00 [106.0000-2000.0000]
CDNTCMESVKN HexNAc(4)Hex(5)Fuc(1)NeuAc(1) GTYDYPKYSEEAK
Relative Abundance
m/z
1651.3195
1554.2853
1651.9875
366.1397
1432.2423
508.1823
1431.9102
1518.6140
407.1350
928.2968
274.0922
1788.1698
1098.5204
460.2179
548.2556
657.2342
1242.4932
1377.8949
331.1742
929.2966
839.4135
1737.6411
1927.2155
178.2970
710.3118
563.2678

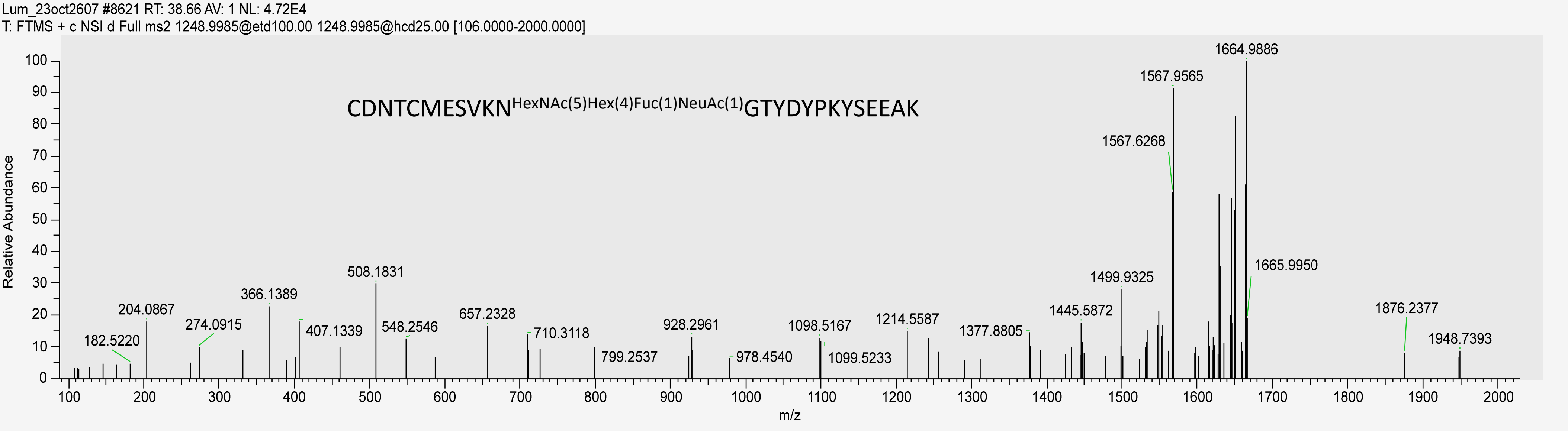
Lum_23oct2607 #8621 RT: 38.66 AV: 1 NL: 4.72E4
T: FTMS + c NSI d Full ms2 1248.9985@etd100.00 1248.9985@hcd25.00 [106.0000-2000.0000]
CDNTCMESVKN HexNAc(5)Hex(4)Fuc(1)NeuAc(1) GTYDYPKYSEEAK
Relative Abundance
m/z
1664.9886
1567.9565
1567.6268
1665.9950
1499.9325
508.1831
366.1389
204.0867
1876.2377
274.0915
1445.5872
1214.5587
657.2328
548.2546
928.2961
1098.5167
407.1339
1377.8805
710.3118
182.5220
1948.7393
799.2537
978.4540
1099.5233

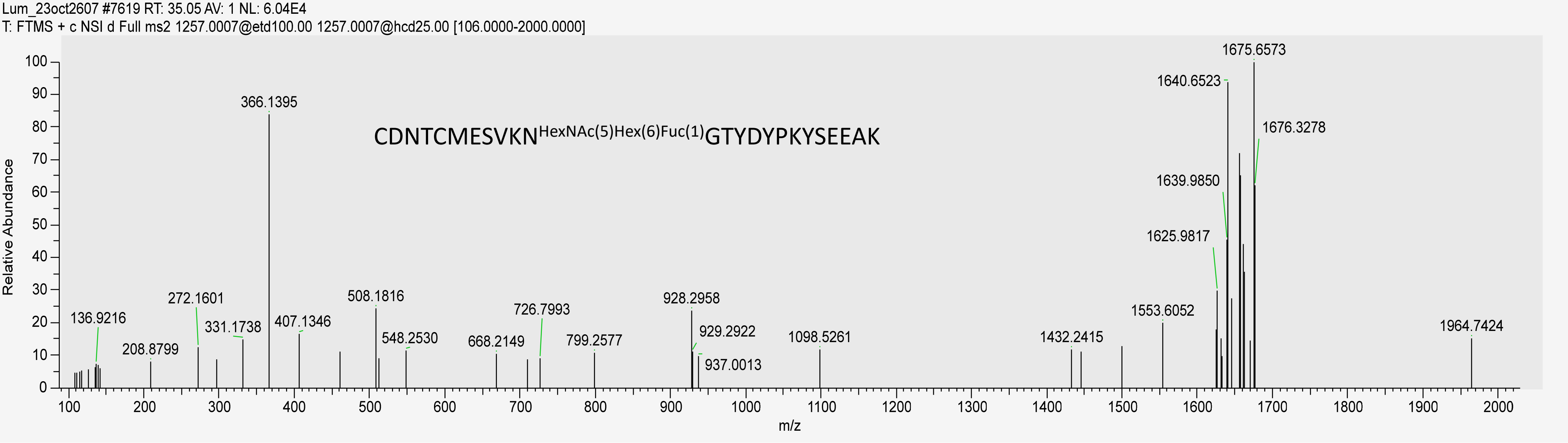
Lum_23oct2607 #7619 RT: 35.05 AV: 1 NL: 6.04E4
T: FTMS + c NSI d Full ms2 1257.0007@etd100.00 1257.0007@hcd25.00 [106.0000-2000.0000]
CDNTCMESVKN HexNAc(5)Hex(6)Fuc(1) GTYDYPKYSEEAK
Relative Abundance
m/z
1675.6573
1640.6523
1676.3278
1639.9850
1625.9817
366.1395
136.9216
208.8799
272.1601
331.1738
407.1346
508.1816
548.2530
668.2149
726.7993
799.2577
928.2958
929.2922
937.0013
1098.5261
1432.2415
1553.6052
1964.7424

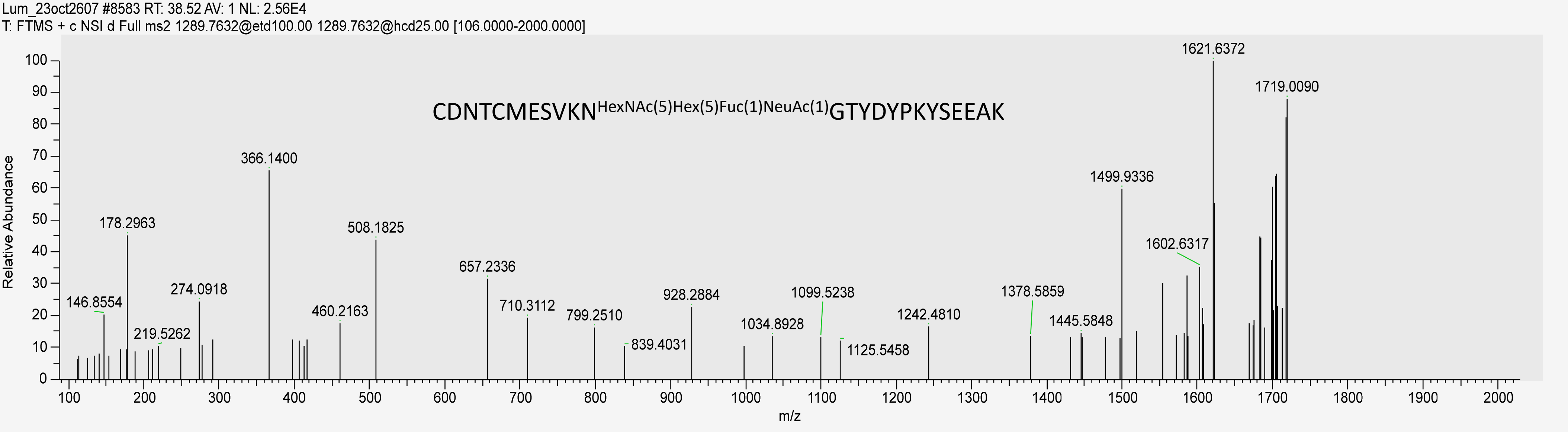
Lum_23oct2607 #8583 RT: 38.52 AV: 1 NL: 2.56E4
T: FTMS + c NSI d Full ms2 1289.7632@etd100.00 1289.7632@hcd25.00 [106.0000-2000.0000]
CDNTCMESVKN HexNAc(5)Hex(5)Fuc(1)NeuAc(1) GTYDYPKYSEEAK
Relative Abundance
m/z
1621.6372
1719.0090
1499.9336
1602.6317
366.1400
178.2963
508.1825
657.2336
274.0918
146.8554
460.2163
710.3112
799.2510
928.2884
1099.5238
1242.4810
1378.5859
1445.5848
1034.8928
219.5262
839.4031
1125.5458

Lum_23oct2607 #9649 RT: 42.37 AV: 1 NL: 3.78E4
T: FTMS + c NSI d Full ms2 1311.5143@etd100.00 1311.5143@hcd25.00 [106.0000-2000.0000]

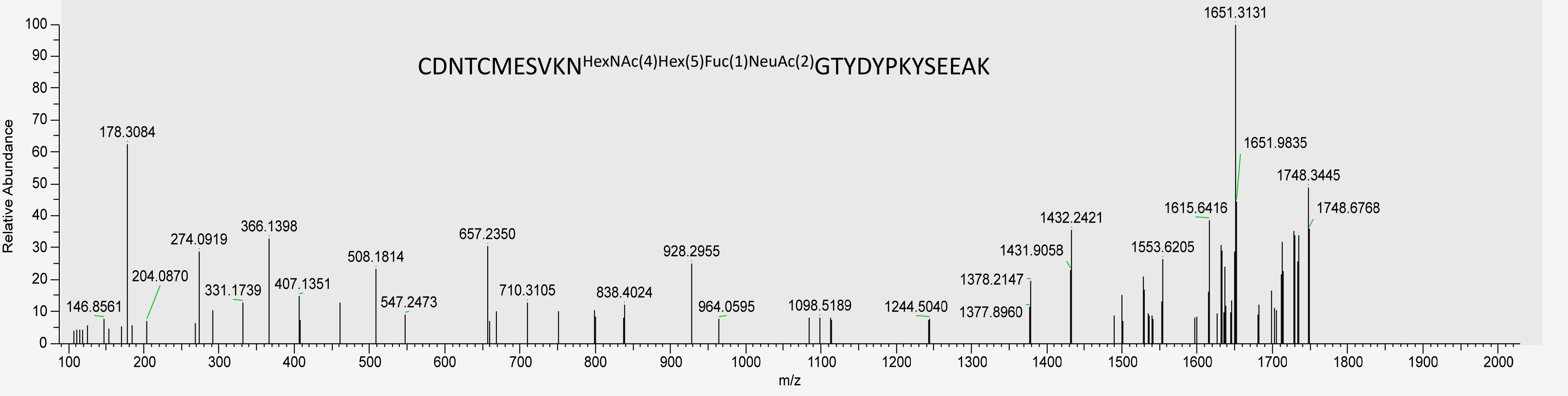

Lum_23oct2607 #9550 RT: 42.01 AV: 1 NL: 4.55E4
T: FTMS + c NSI d Full ms2 1402.7994@etd100.00 1402.7994@hcd25.00 [106.0000-2000.0000]

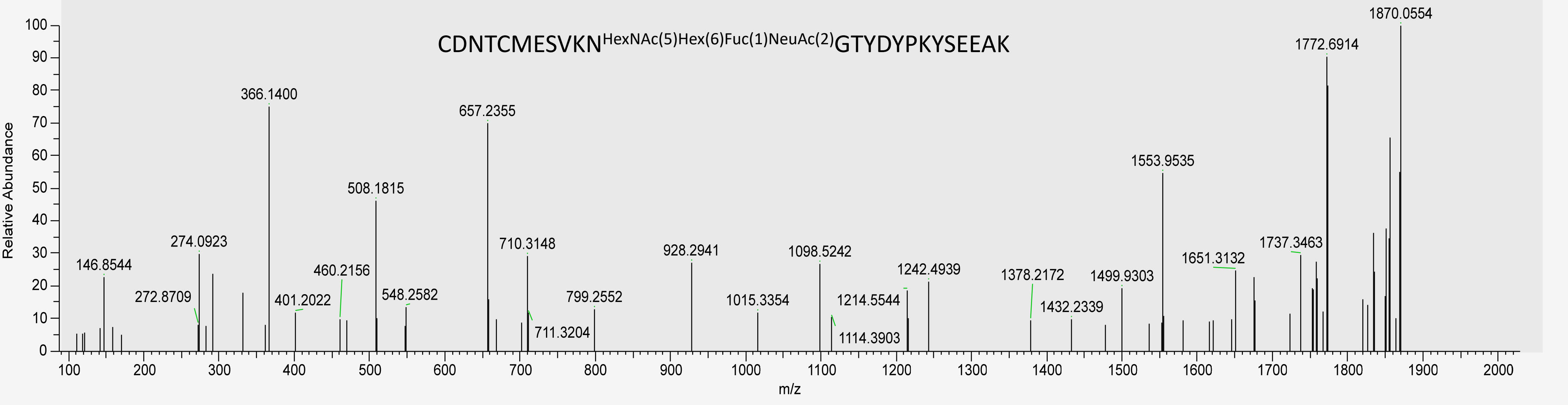

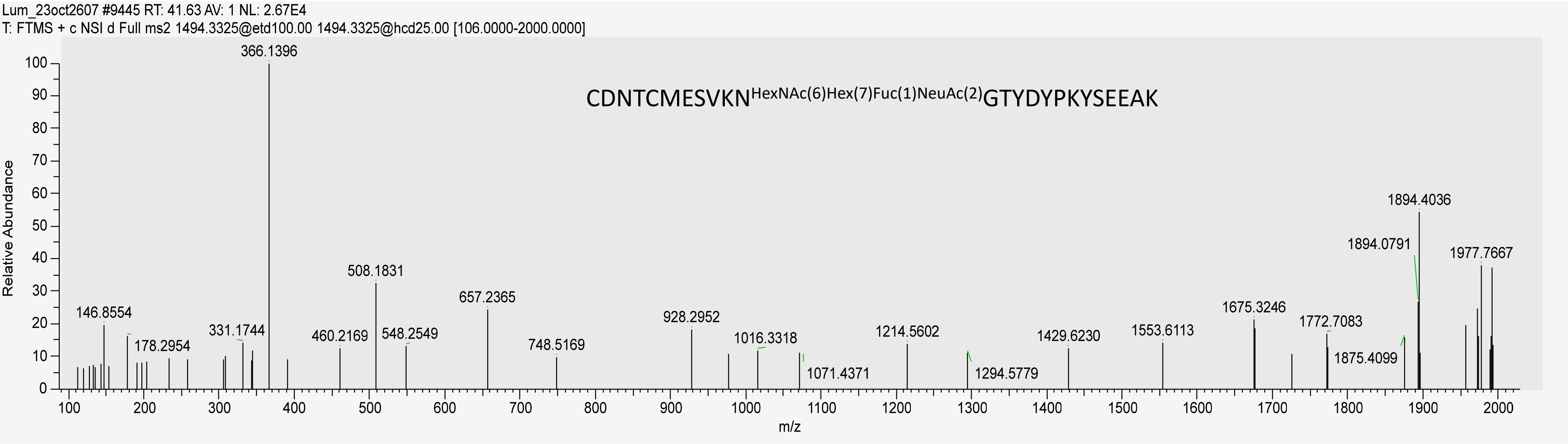

Lum_23oct2607 #9445 RT: 41.63 AV: 1 NL: 2.67E4
T: FTMS + c NSI d Full ms2 1494.3325@etd100.00 1494.3325@hcd25.00 [106.0000-2000.0000]
CDNTCMESVKN HexNAc(6)Hex(7)Fuc(1)NeuAc(2) GTYDYPKYSEEAK
Relative Abundance
m/z
366.1396
146.8554
178.2954
331.1744
460.2169
508.1831
548.2549
657.2365
748.5169
928.2952
1016.3318
1071.4371
1214.5602
1294.5779
1429.6230
1553.6113
1675.3246
1772.7083
1875.4099
1894.0791
1894.4036
1977.7667

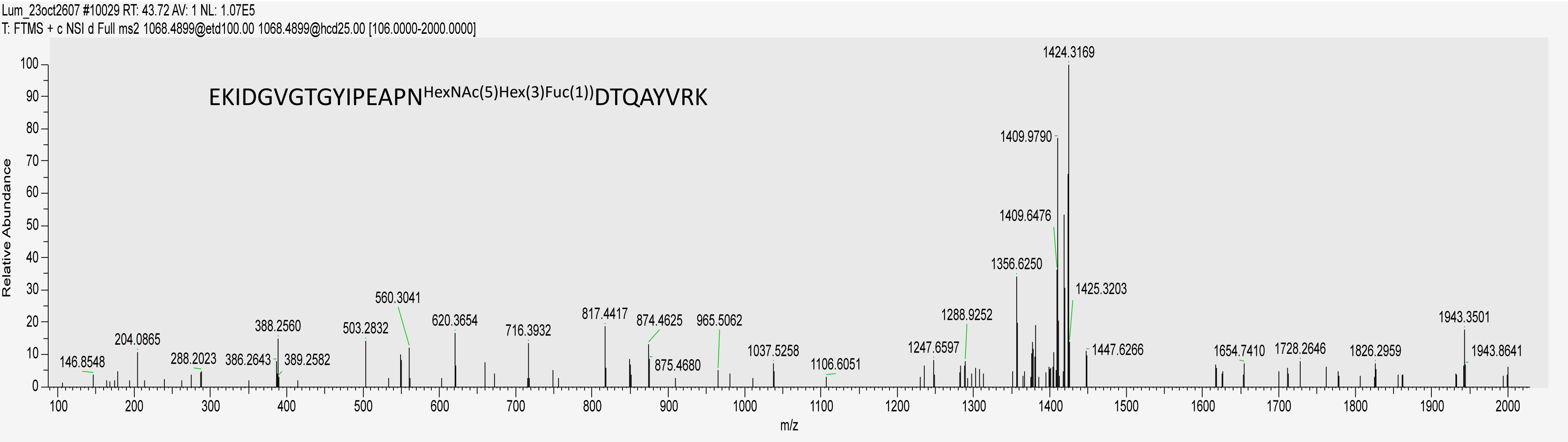

Lum_23oct2607 #10029 RT: 43.72 AV: 1 NL: 1.07E5
T: FTMS + c NSI d Full ms2 1068.4899@etd100.00 1068.4899@hcd25.00 [106.0000-2000.0000]
EKIDGVGTGYIPEAPN HexNAc(5)Hex(3)Fuc(1)) DTQAYVRK
Relative Abundance
m/z
1424.3169
1409.9790
1409.6476
1356.6250
1425.3203
1288.9252
1943.3501
1247.6597
1447.6266
1654.7410
1728.2646
1826.2959
1943.8641
560.3041
620.3654
817.4417
874.4625
965.5062
716.3932
503.2832
388.2560
204.0865
1037.5258
1106.6051
875.4680
146.8548
288.2023
386.2643
389.2582

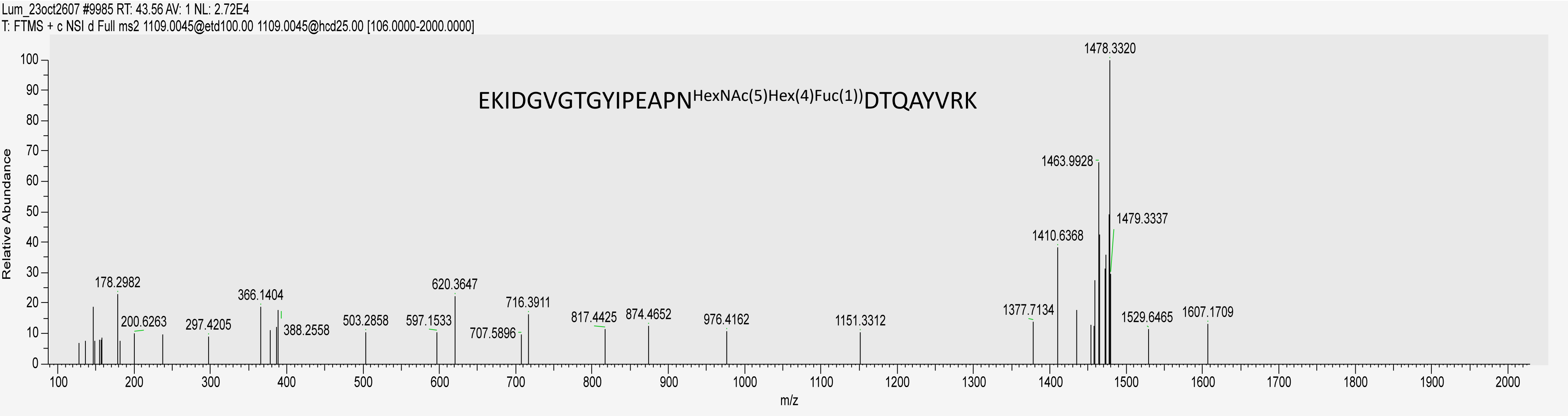

Lum_23oct2607 #9985 RT: 43.56 AV: 1 NL: 2.72E4
T: FTMS + c NSI d Full ms2 1109.0045@etd100.00 1109.0045@hcd25.00 [106.0000-2000.0000]
EKIDGVGTGYIPEAPN HexNAc(5)Hex(4)Fuc(1)) DTQAYVRK
Relative Abundance
m/z
1478.3320
1463.9928
1479.3337
1410.6368
1377.7134
1529.6465
1607.1709
178.2982
366.1404
620.3647
716.3911
200.6263
297.4205
388.2558
503.2858
597.1533
707.5896
817.4425
874.4652
976.4162
1151.3312

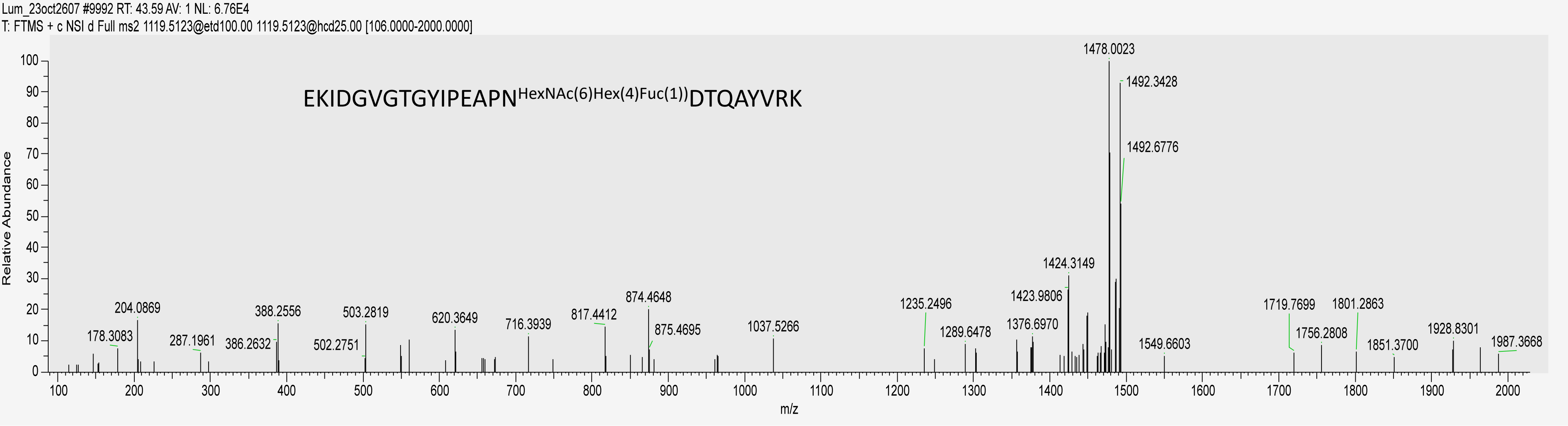
Lum_23oct2607 #9992 RT: 43.59 AV: 1 NL: 6.76E4
T: FTMS + c NSI d Full ms2 1119.5123@etd100.00 1119.5123@hcd25.00 [106.0000-2000.0000]
EKIDGVGTGYIPEAPN HexNAc(6)Hex(4)Fuc(1)) DTQAYVRK
Relative Abundance
m/z
1478.0023
1492.3428
1492.6776
1424.3149
1423.9806
1376.6970
1289.6478
1235.2496
1037.5266
874.4648
875.4695
817.4412
716.3939
620.3649
503.2819
502.2751
388.2556
386.2632
287.1961
204.0869
178.3083
1549.6603
1719.7699
1756.2808
1801.2863
1851.3700
1928.8301
1987.3668

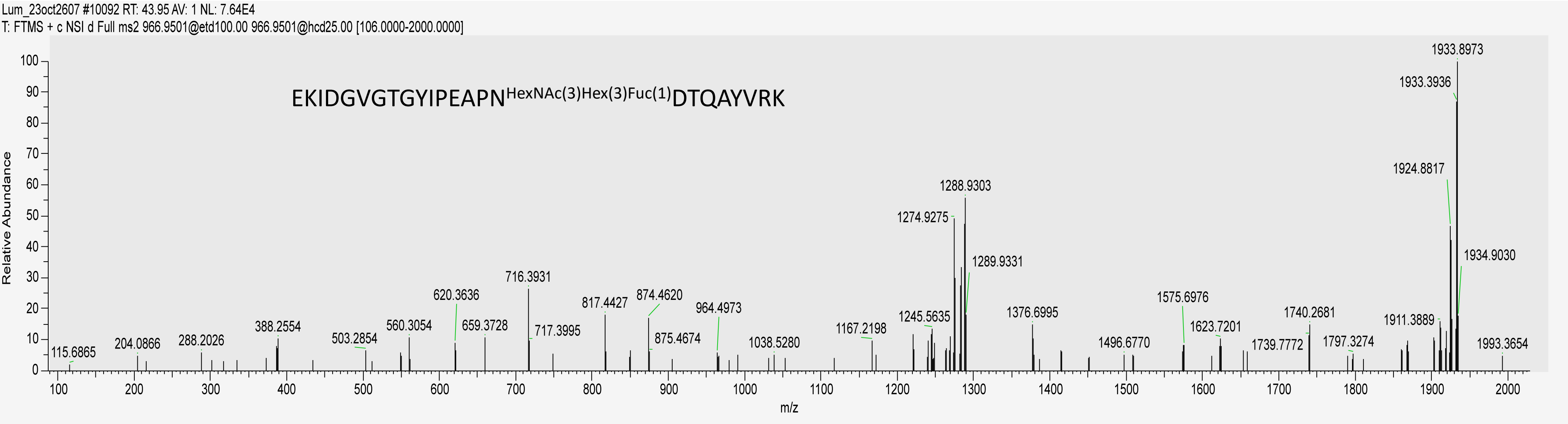
Lum_23oct2607 #10092 RT: 43.95 AV: 1 NL: 7.64E4
T: FTMS + c NSI d Full ms2 966.9501@etd100.00 966.9501@hcd25.00 [106.0000-2000.0000]
EKIDGVGTGYIPEAPN HexNAc(3)Hex(3)Fuc(1) DTQAYVRK
Relative Abundance
m/z
1933.8973
1933.3936
1924.8817
1934.9030
1911.3889
1993.3654
1797.3274
1740.2681
1739.7772
1623.7201
1575.6976
1496.6770
1376.6995
1288.9303
1274.9275
1289.9331
1245.5635
1167.2198
1038.5280
964.4973
874.4620
875.4674
817.4427
716.3931
717.3995
659.3728
620.3636
560.3054
503.2854
388.2554
288.2026
204.0866
115.6865

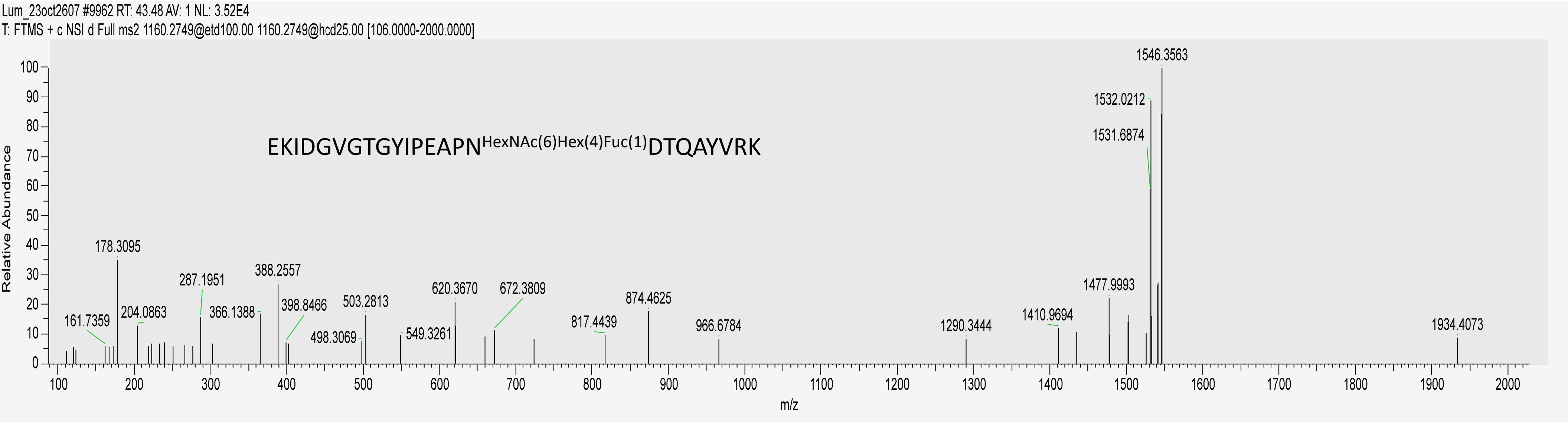
Lum_23oct2607 #9962 RT: 43.48 AV: 1 NL: 3.52E4
T: FTMS + c NSI d Full ms2 1160.2749@etd100.00 1160.2749@hcd25.00 [106.0000-2000.0000]
EKIDGVGTGYIPEAPN HexNAc(6)Hex(4)Fuc(1) DTQAYVRK
Relative Abundance
m/z
1546.3563
1532.0212
1531.6874
178.3095
287.1951
388.2557
366.1388
398.8466
161.7359
204.0863
503.2813
498.3069
549.3261
620.3670
672.3809
817.4439
874.4625
966.6784
1290.3444
1410.9694
1477.9993
1934.4073

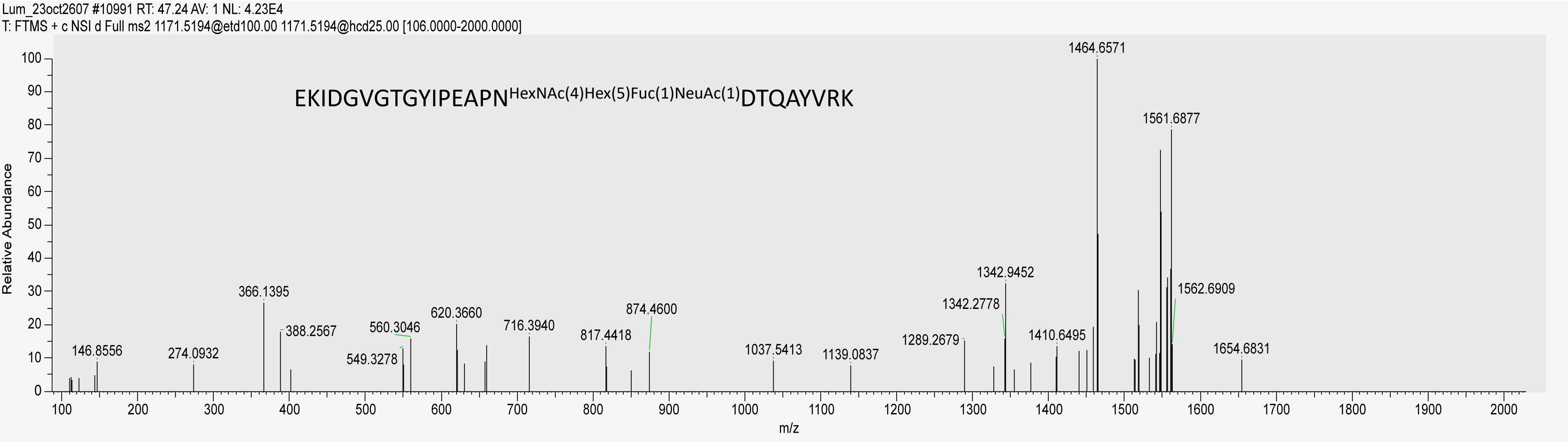
Lum_23oct2607 #10991 RT: 47.24 AV: 1 NL: 4.23E4
T: FTMS + c NSI d Full ms2 1171.5194@etd100.00 1171.5194@hcd25.00 [106.0000-2000.0000]
EKIDGVGTGYIPEAPN HexNAc(4)Hex(5)Fuc(1)NeuAc(1) DTQAYVRK
Relative Abundance
m/z
1464.6571
1561.6877
1342.9452
1562.6909
1342.2778
366.1395
874.4600
620.3660
388.2567
560.3046
716.3940
817.4418
1289.2679
1410.6495
146.8556
274.0932
549.3278
1037.5413
1139.0837
1654.6831

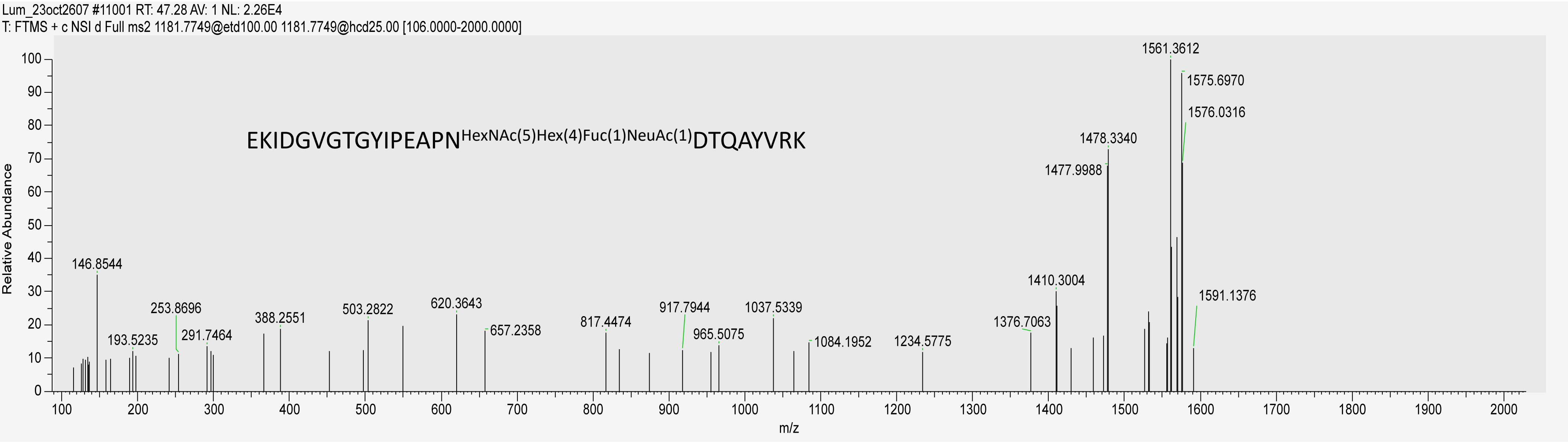
Lum_23oct2607 #11001 RT: 47.28 AV: 1 NL: 2.26E4
T: FTMS + c NSI d Full ms2 1181.7749@etd100.00 1181.7749@hcd25.00 [106.0000-2000.0000]
EKIDGVGTGYIPEAPN HexNAc(5)Hex(4)Fuc(1)NeuAc(1) DTQAYVRK
Relative Abundance
m/z
1561.3612
1575.6970
1576.0316
1478.3340
1477.9988
146.8544
1410.3004
1591.1376
253.8696
620.3643
503.2822
917.7944
1037.5339
388.2551
1376.7063
817.4474
291.7464
193.5235
657.2358
965.5075
1084.1952
1234.5775

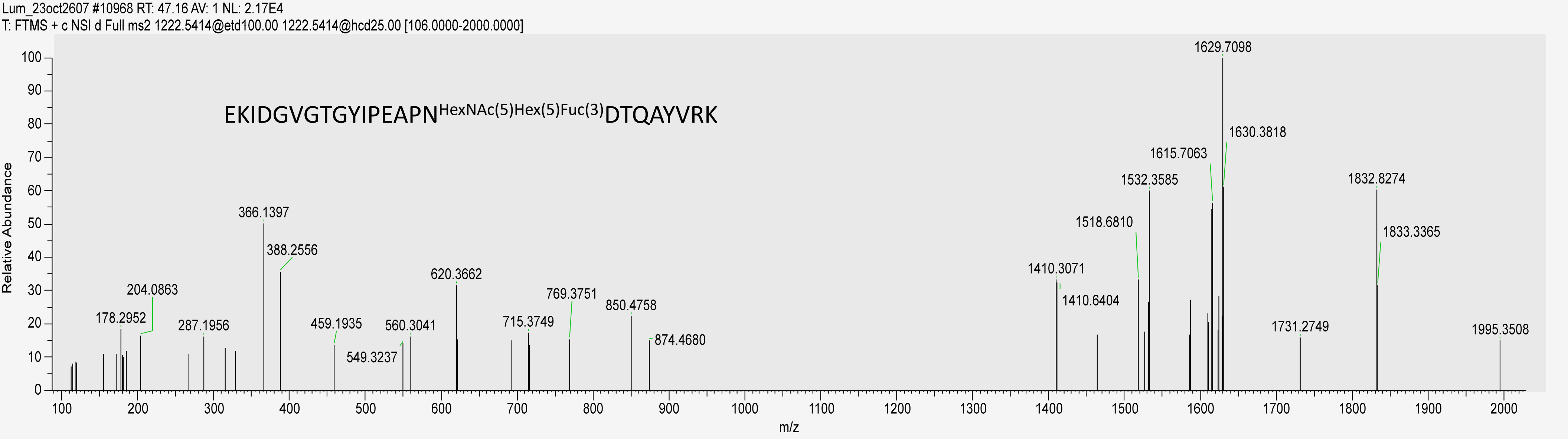
Lum_23oct2607 #10968 RT: 47.16 AV: 1 NL: 2.17E4
T: FTMS + c NSI d Full ms2 1222.5414@etd100.00 1222.5414@hcd25.00 [106.0000-2000.0000]
EKIDGVGTGYIPEAPN HexNAc(5)Hex(5)Fuc(3) DTQAYVRK
Relative Abundance
m/z
1629.7098
1630.3818
1615.7063
1532.3585
1832.8274
1518.6810
1833.3365
366.1397
388.2556
1410.3071
204.0863
620.3662
1410.6404
769.3751
178.2952
850.4758
287.1956
459.1935
560.3041
715.3749
1731.2749
1995.3508
874.4680
549.3237

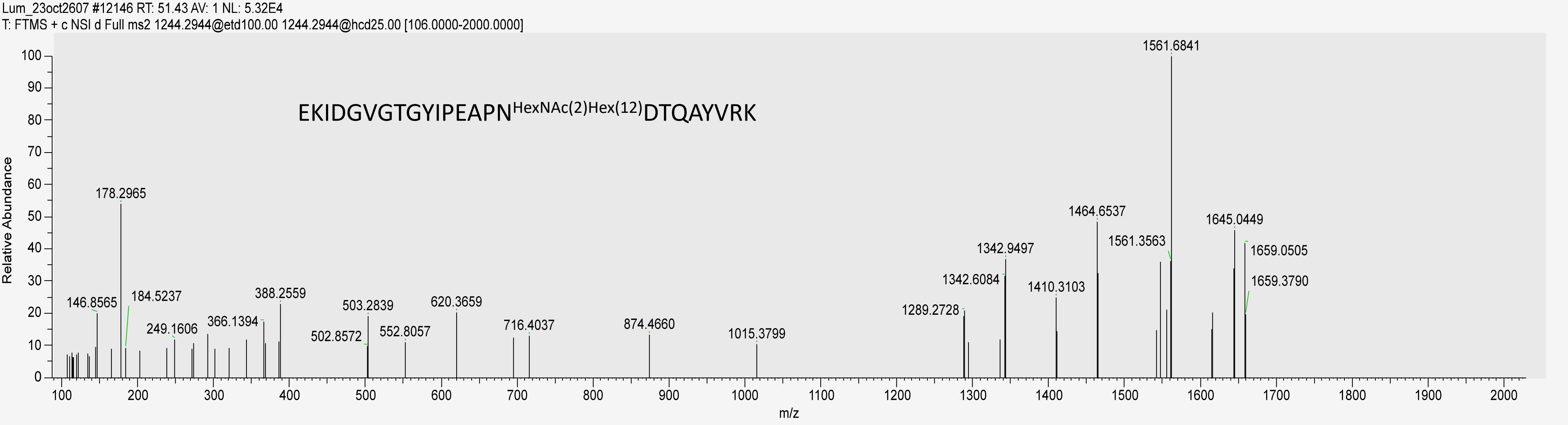
Lum_23oct2607 #12146 RT: 51.43 AV: 1 NL: 5.32E4
T: FTMS + c NSI d Full ms2 1244.2944@etd100.00 1244.2944@hcd25.00 [106.0000-2000.0000]
EKIDGVGTGYIPEAPN HexNAc(2)Hex(12) DTQAYVRK
Relative Abundance
m/z
1561.6841
178.2965
1464.6537
1645.0449
1561.3563
1659.0505
1342.9497
1342.6084
1410.3103
1659.3790
146.8565
184.5237
388.2559
503.2839
620.3659
1289.2728
249.1606
366.1394
502.8572
552.8057
716.4037
874.4660
1015.3799

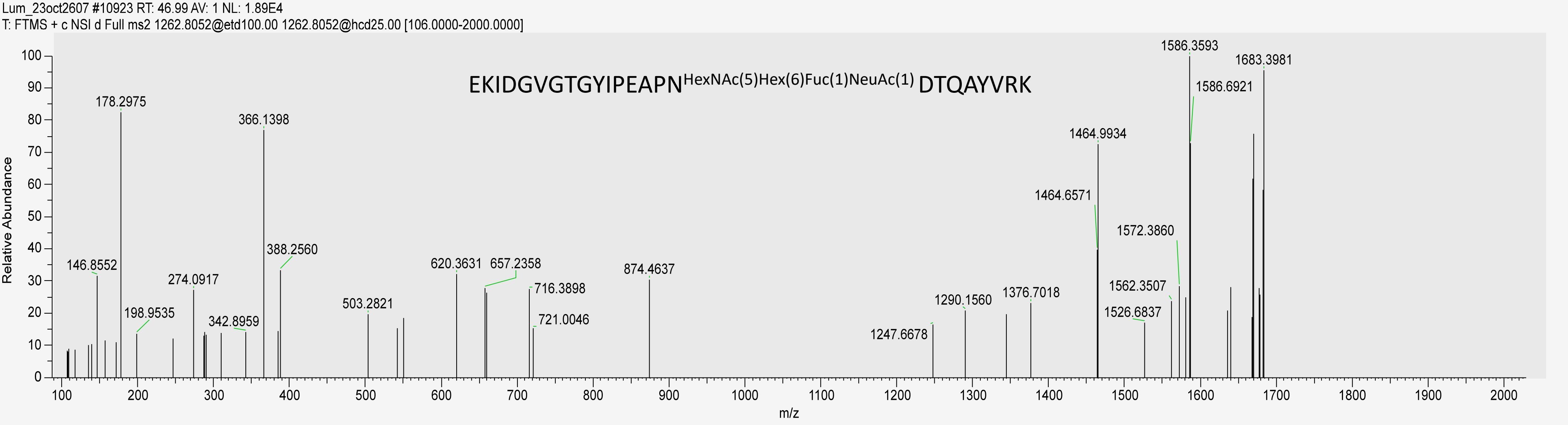
Lum_23oct2607 #10923 RT: 46.99 AV: 1 NL: 1.89E4
T: FTMS + c NSI d Full ms2 1262.8052@etd100.00 1262.8052@hcd25.00 [106.0000-2000.0000]
EKIDGVGTGYIPEAPN HexNAc(5)Hex(6)Fuc(1)NeuAc(1) DTQAYVRK
Relative Abundance
m/z
1586.3593
1683.3981
1586.6921
178.2975
366.1398
1464.9934
1464.6571
1572.3860
388.2560
146.8552
274.0917
620.3631
657.2358
874.4637
716.3898
1562.3507
1290.1560
1376.7018
198.9535
342.8959
503.2821
721.0046
1526.6837
1247.6678

Lum_23oct2607 #10927 RT: 47.01 AV: 1 NL: 2.20E4
T: FTMS + c NSI d Full ms2 1273.3113@etd100.00 1273.3113@hcd25.00 [106.0000-2000.0000]

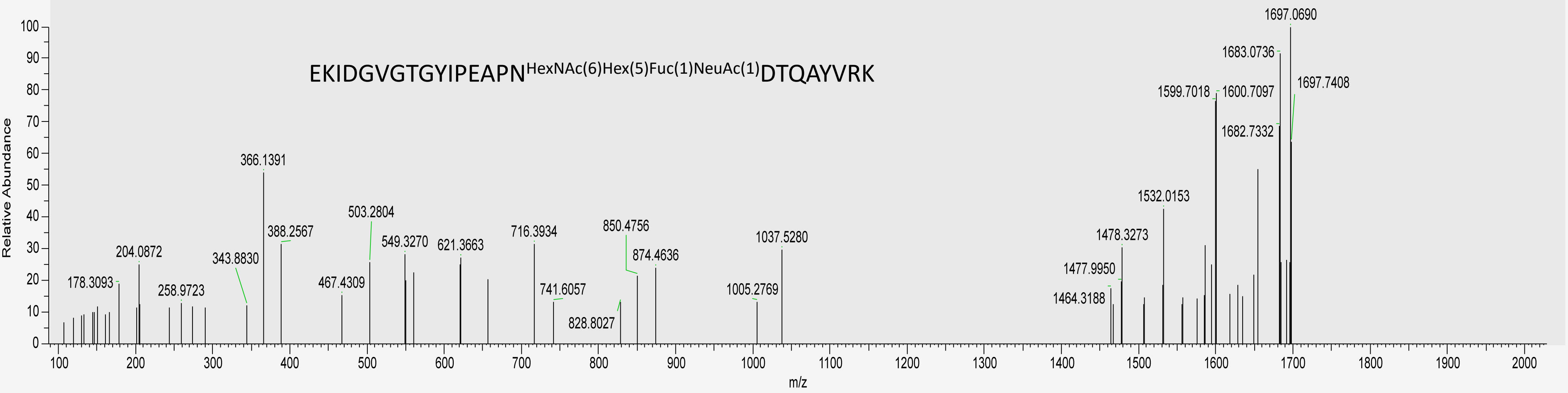

Lum_23oct2607 #10903 RT: 46.92 AV: 1 NL: 1.20E4
T: FTMS + c NSI d Full ms2 1313.8235@etd100.00 1313.8235@hcd25.00 [106.0000-2000.0000]

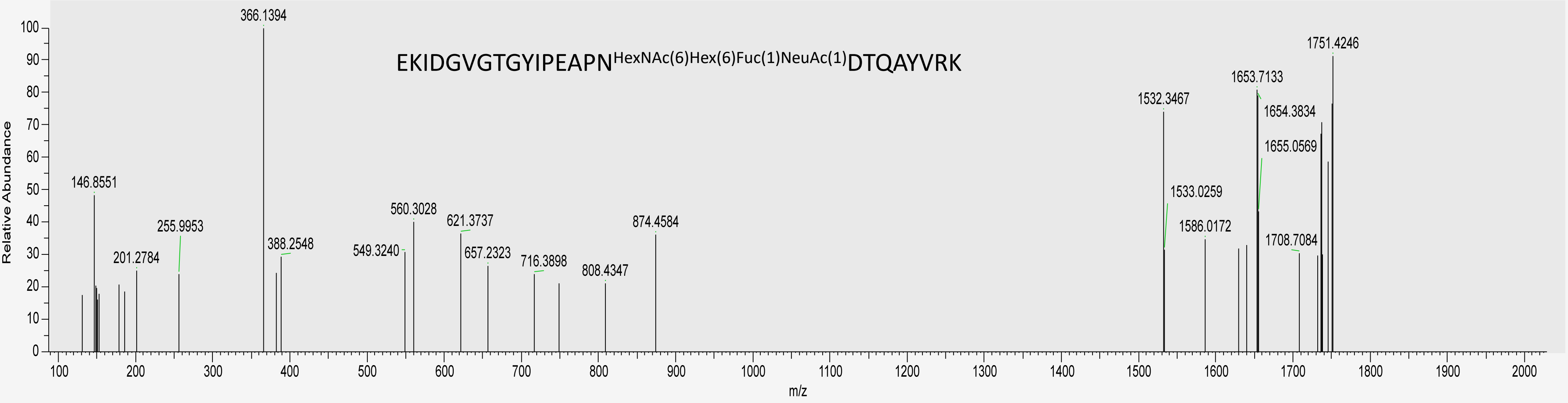

Lum_23oct2607 #12061 RT: 51.12 AV: 1 NL: 3.58E4
T: FTMS + c NSI d Full ms2 1335.5768@etd100.00 1335.5768@hcd25.00 [106.0000-2000.0000]

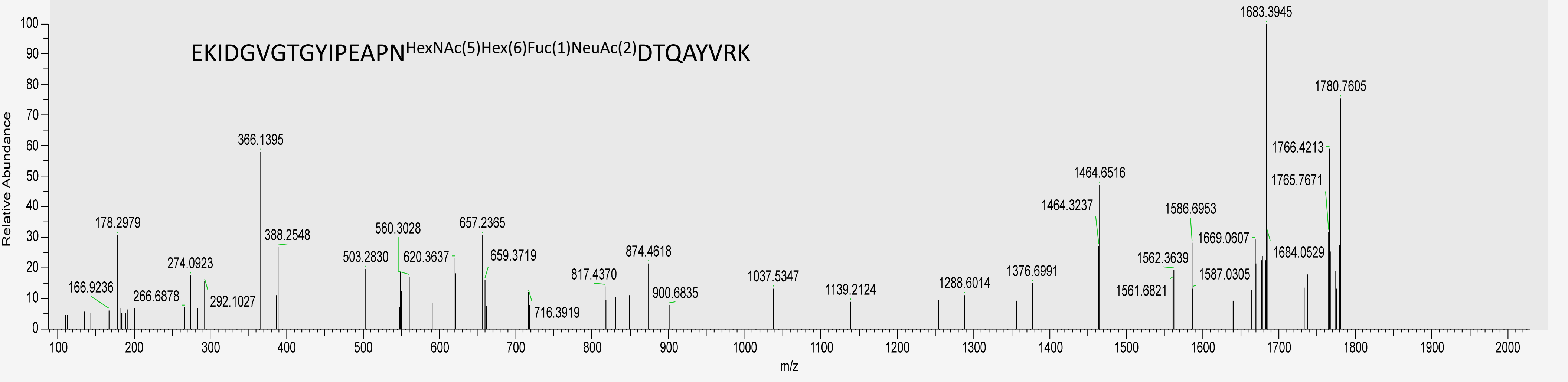

Lum_23oct2607 #12032 RT: 51.01 AV: 1 NL: 1.52E4
T: FTMS + c NSI d Full ms2 1386.3462@etd100.00 1386.3462@hcd25.00 [106.0000-2000.0000]

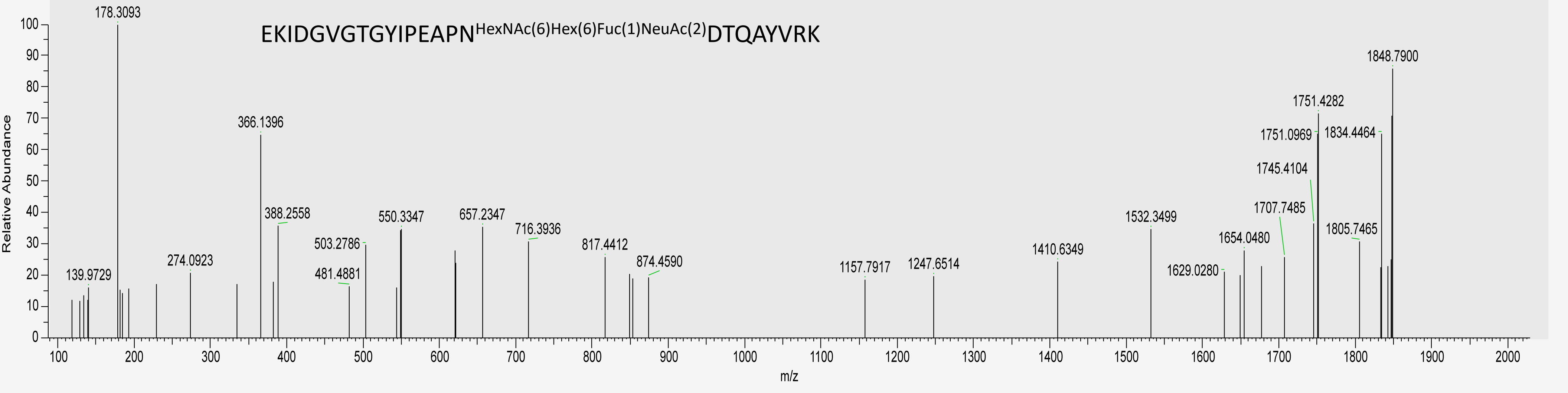

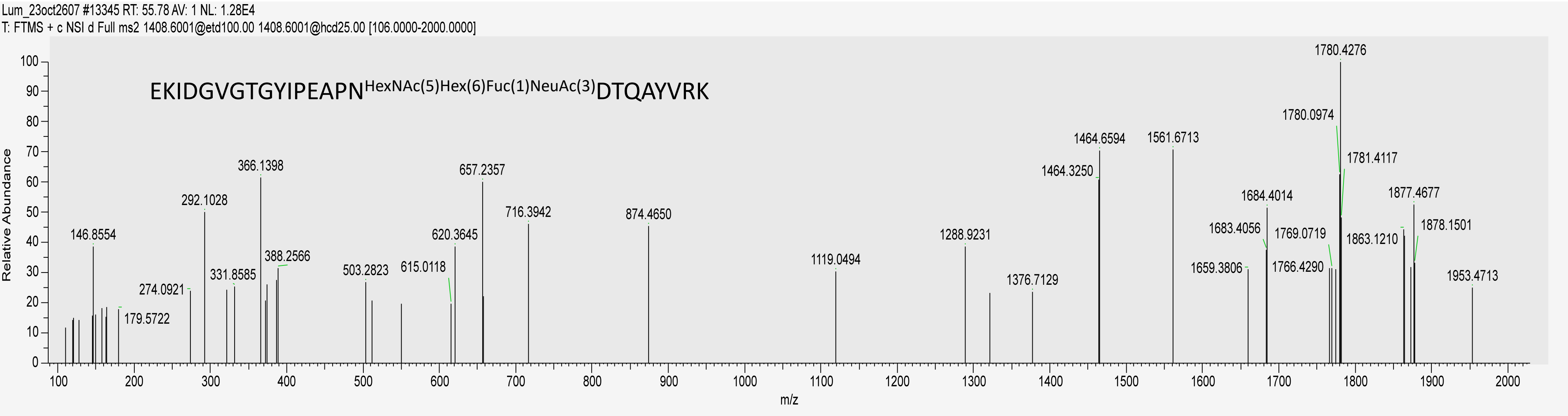
Lum_23oct2607 #13345 RT: 55.78 AV: 1 NL: 1.28E4
T: FTMS + c NSI d Full ms2 1408.6001@etd100.00 1408.6001@hcd25.00 [106.0000-2000.0000]
EKIDGVGTGYIPEAPN HexNAc(5)Hex(6)Fuc(1)NeuAc(3) DTQAYVRK
Relative Abundance
m/z
146.8554
179.5722
274.0921
292.1028
331.8585
366.1398
388.2566
503.2823
615.0118
620.3645
657.2357
716.3942
874.4650
1119.0494
1288.9231
1376.7129
1464.3250
1464.6594
1561.6713
1659.3806
1683.4056
1684.4014
1766.4290
1769.0719
1780.0974
1780.4276
1781.4117
1863.1210
1877.4677
1878.1501
1953.4713

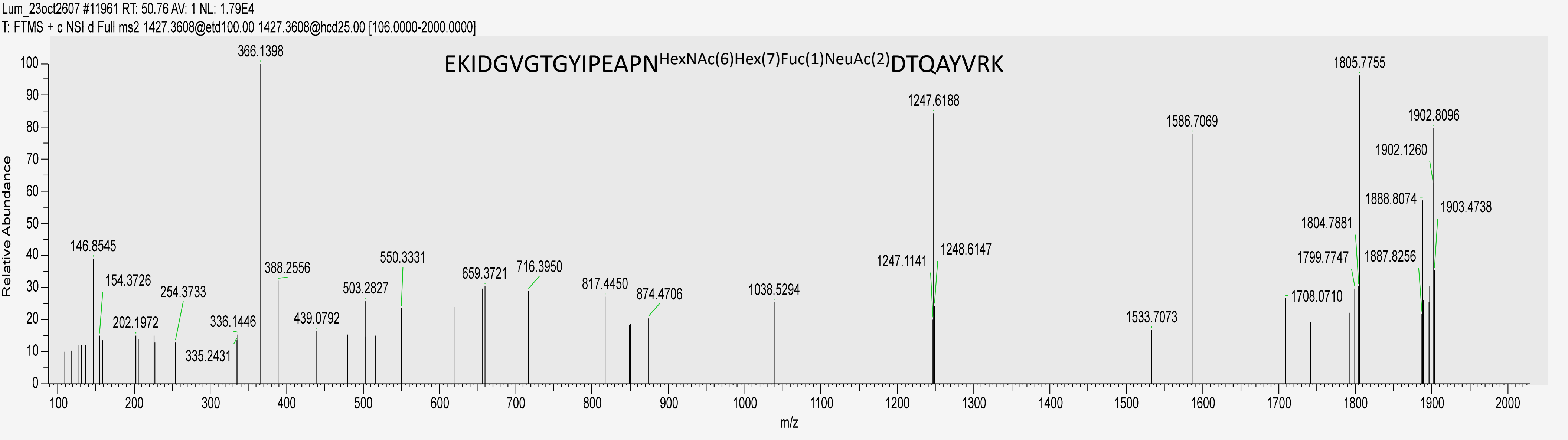
Lum_23oct2607 #11961 RT: 50.76 AV: 1 NL: 1.79E4
T: FTMS + c NSI d Full ms2 1427.3608@etd100.00 1427.3608@hcd25.00 [106.0000-2000.0000]
EKIDGVGTGYIPEAPN HexNAc(6)Hex(7)Fuc(1)NeuAc(2) DTQAYVRK
Relative Abundance
m/z
146.8545
154.3726
202.1972
254.3733
335.2431
336.1446
366.1398
388.2556
439.0792
503.2827
550.3331
659.3721
716.3950
817.4450
874.4706
1038.5294
1247.1141
1247.6188
1248.6147
1533.7073
1586.7069
1708.0710
1799.7747
1804.7881
1805.7755
1887.8256
1888.8074
1902.1260
1902.8096
1903.4738

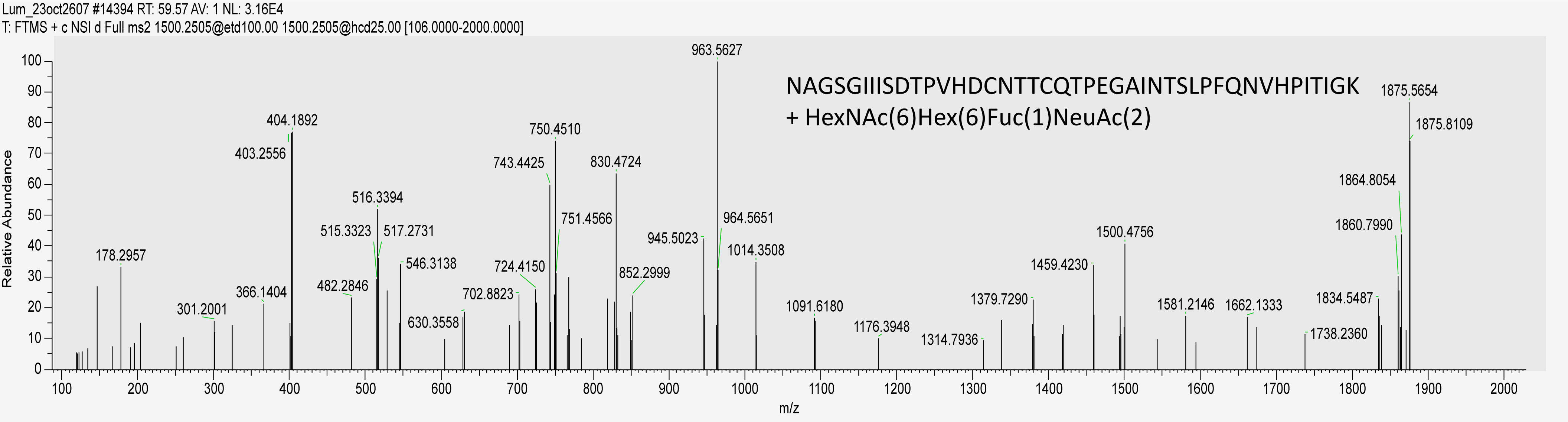
Lum_23oct2607 #14394 RT: 59.57 AV: 1 NL: 3.16E4
T: FTMS + c NSI d Full ms2 1500.2505@etd100.00 1500.2505@hcd25.00 [106.0000-2000.0000]
NAGSGIIISDTPVHDCNTTCQTPEGAINTSLPFQNVHPITIGK
+ HexNAc(6)Hex(6)Fuc(1)NeuAc(2)
Relative Abundance
m/z
963.5627
1875.5654
1875.8109
404.1892
403.2556
750.4510
743.4425
830.4724
1864.8054
516.3394
751.4566
964.5651
515.3323
517.2731
1500.4756
945.5023
1860.7990
178.2957
1014.3508
546.3138
1459.4230
724.4150
852.2999
366.1404
482.2846
702.8823
1379.7290
1834.5487
301.2001
1091.6180
1581.2146
1662.1333
630.3558
1176.3948
1314.7936
1738.2360

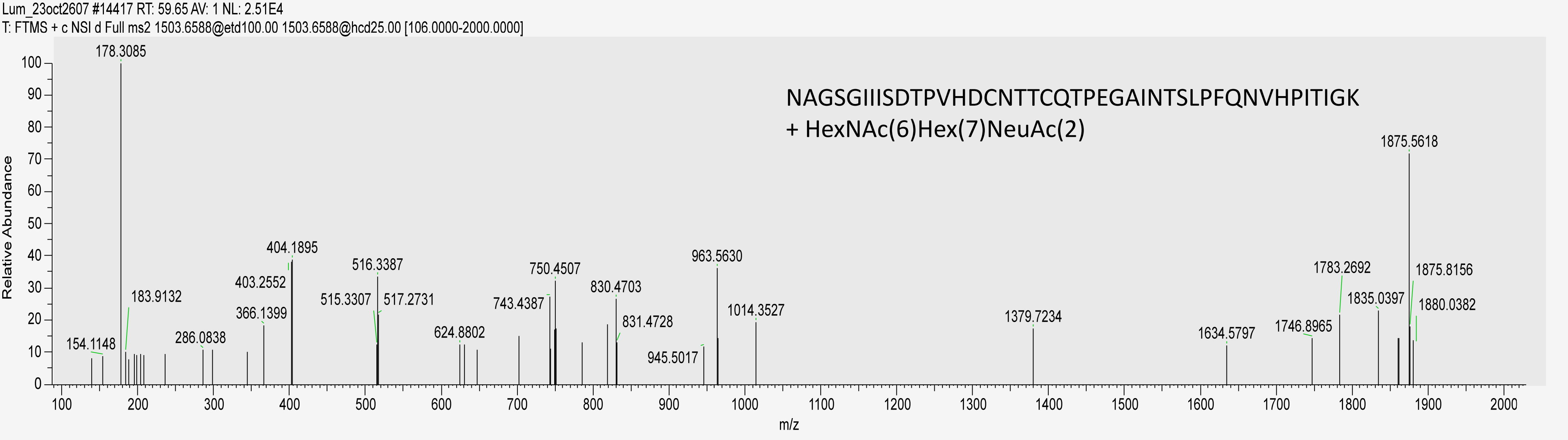
Lum_23oct2607 #14417 RT: 59.65 AV: 1 NL: 2.51E4
T: FTMS + c NSI d Full ms2 1503.6588@etd100.00 1503.6588@hcd25.00 [106.0000-2000.0000]
NAGSGIIISDTPVHDCNTTCQTPEGAINTSLPFQNVHPITIGK
+ HexNAc(6)Hex(7)NeuAc(2)
Relative Abundance
m/z
178.3085
1875.5618
404.1895
516.3387
750.4507
963.5630
403.2552
830.4703
1783.2692
1875.8156
183.9132
515.3307
517.2731
743.4387
1014.3527
1835.0397
366.1399
831.4728
1379.7234
1880.0382
1746.8965
154.1148
286.0838
624.8802
1634.5797
945.5017

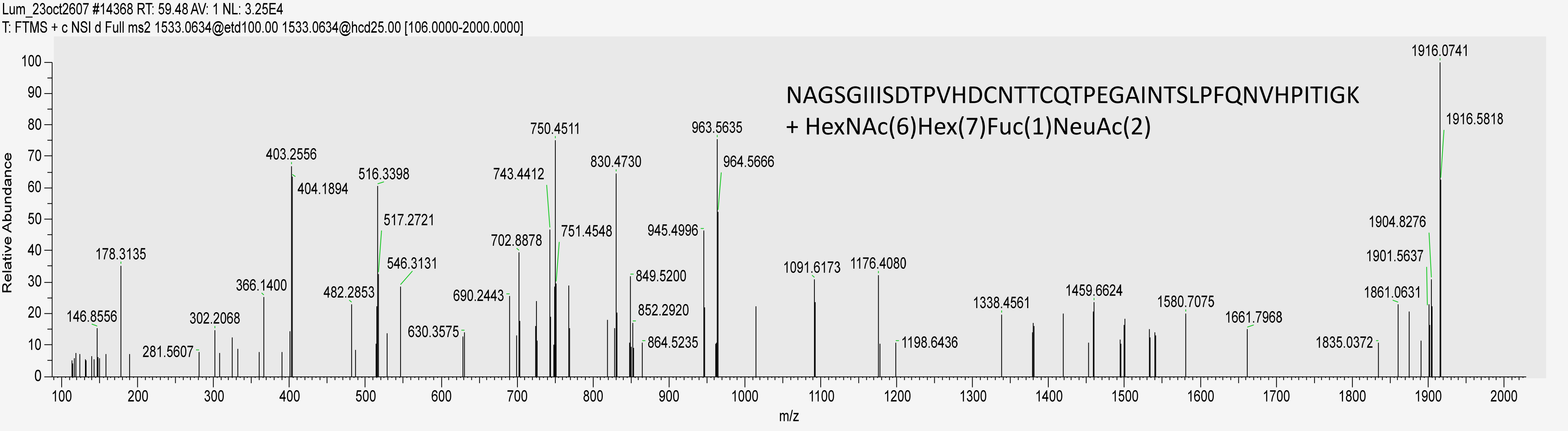
Lum_23oct2607 #14368 RT: 59.48 AV: 1 NL: 3.25E4
T: FTMS + c NSI d Full ms2 1533.0634@etd100.00 1533.0634@hcd25.00 [106.0000-2000.0000]
NAGSGIIISDTPVHDCNTTCQTPEGAINTSLPFQNVHPITIGK
+ HexNAc(6)Hex(7)Fuc(1)NeuAc(2)
Relative Abundance
m/z
1916.0741
1916.5818
963.5635
964.5666
750.4511
743.4412
751.4548
830.4730
403.2556
404.1894
516.3398
517.2721
546.3131
482.2853
366.1400
302.2068
281.5607
178.3135
146.8556
702.8878
690.2443
630.3575
945.4996
849.5200
852.2920
864.5235
1091.6173
1176.4080
1198.6436
1338.4561
1459.6624
1580.7075
1661.7968
1835.0372
1861.0631
1901.5637
1904.8276

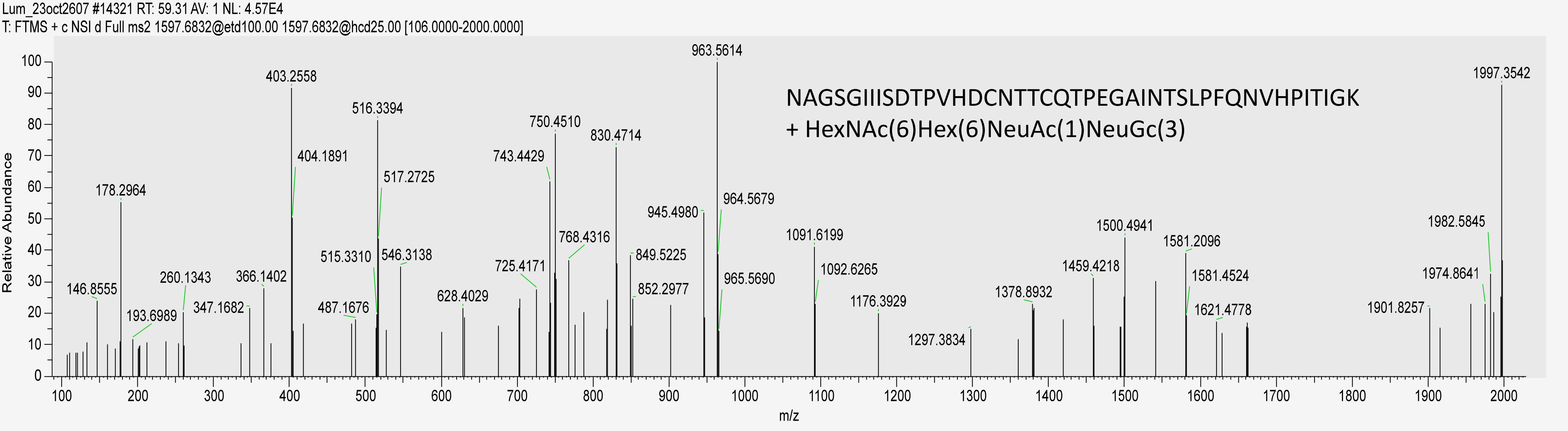
Lum_23oct2607 #14321 RT: 59.31 AV: 1 NL: 4.57E4
T: FTMS + c NSI d Full ms2 1597.6832@etd100.00 1597.6832@hcd25.00 [106.0000-2000.0000]
NAGSGIIISDTPVHDCNTTCQTPEGAINTSLPFQNVHPITIGK
+ HexNAc(6)Hex(6)NeuAc(1)NeuGc(3)
Relative Abundance
m/z
963.5614
1997.3542
403.2558
404.1891
516.3394
517.2725
515.3310
546.3138
487.1676
366.1402
347.1682
260.1343
193.6989
178.2964
146.8555
628.4029
725.4171
743.4429
750.4510
768.4316
830.4714
849.5225
852.2977
945.4980
964.5679
965.5690
1091.6199
1092.6265
1176.3929
1297.3834
1378.8932
1459.4218
1500.4941
1581.2096
1581.4524
1621.4778
1901.8257
1974.8641
1982.5845

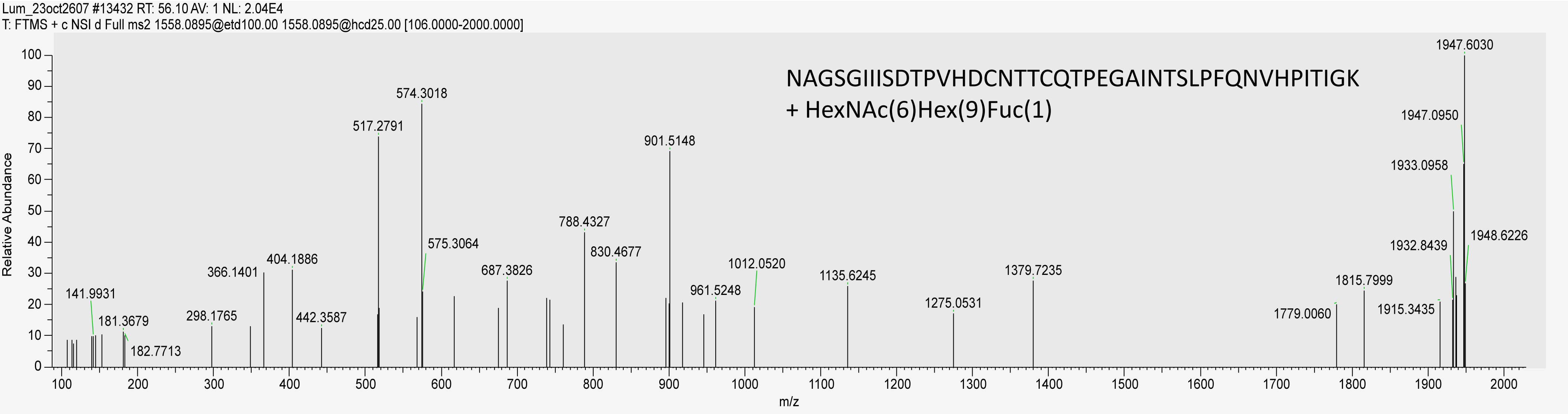
Lum_23oct2607 #13432 RT: 56.10 AV: 1 NL: 2.04E4
T: FTMS + c NSI d Full ms2 1558.0895@etd100.00 1558.0895@hcd25.00 [106.0000-2000.0000]
NAGSGIIISDTPVHDCNTTCQTPEGAINTSLPFQNVHPITIGK
+ HexNAc(6)Hex(9)Fuc(1)
Relative Abundance
m/z
1947.6030
574.3018
517.2791
1947.0950
901.5148
1933.0958
788.4327
575.3064
1948.6226
1932.8439
404.1886
830.4677
366.1401
687.3826
1012.0520
1135.6245
1379.7235
1815.7999
961.5248
141.9931
1275.0531
1915.3435
298.1765
1779.0060
181.3679
442.3587
182.7713

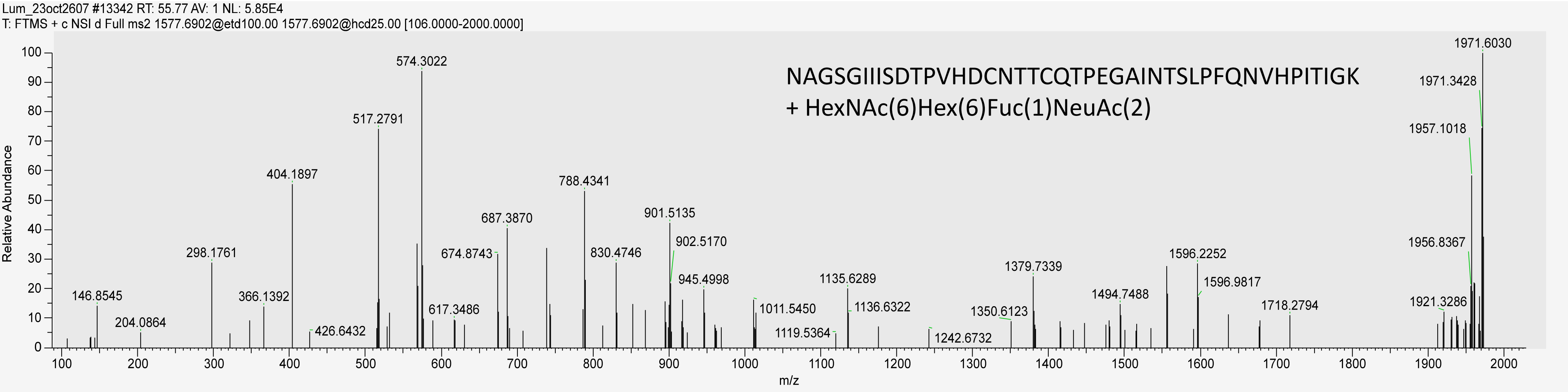
Lum_23oct2607 #13342 RT: 55.77 AV: 1 NL: 5.85E4
T: FTMS + c NSI d Full ms2 1577.6902@etd100.00 1577.6902@hcd25.00 [106.0000-2000.0000]
NAGSGIIISDTPVHDCNTTCQTPEGAINTSLPFQNVHPITIGK
+ HexNAc(6)Hex(6)Fuc(1)NeuAc(2)
Relative Abundance
m/z
1971.6030
574.3022
1971.3428
517.2791
1957.1018
404.1897
788.4341
687.3870
901.5135
902.5170
298.1761
674.8743
830.4746
1596.2252
1956.8367
1379.7339
945.4998
1135.6289
1596.9817
146.8545
366.1392
1494.7488
1718.2794
1921.3286
617.3486
1011.5450
1136.6322
1350.6123
204.0864
426.6432
1119.5364
1242.6732

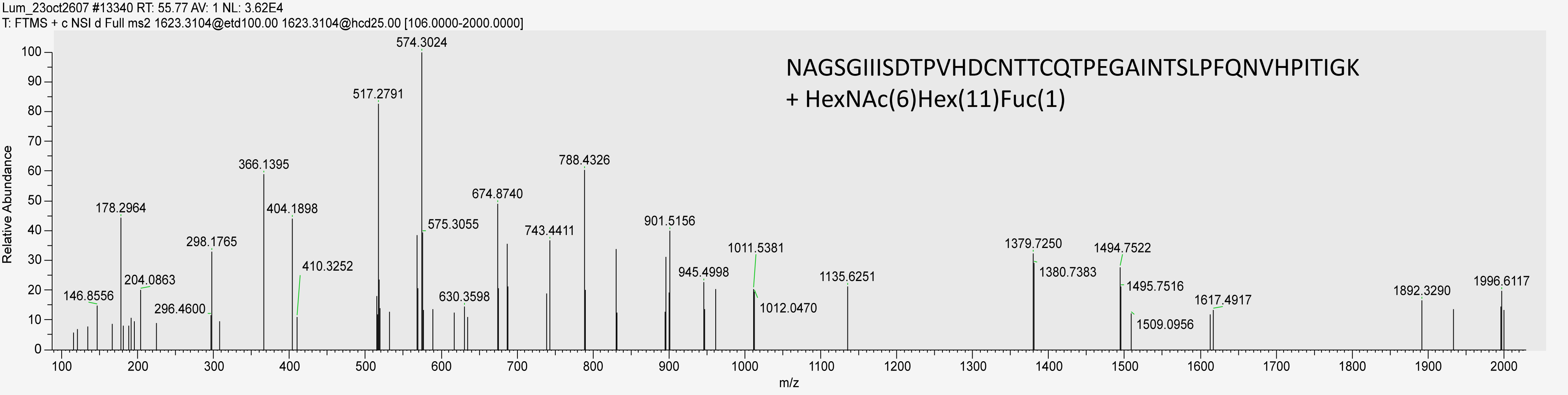
Lum_23oct2607 #13340 RT: 55.77 AV: 1 NL: 3.62E4
T: FTMS + c NSI d Full ms2 1623.3104@etd100.00 1623.3104@hcd25.00 [106.0000-2000.0000]
NAGSGIIISDTPVHDCNTTCQTPEGAINTSLPFQNVHPITIGK
+ HexNAc(6)Hex(11)Fuc(1)
Relative Abundance
m/z

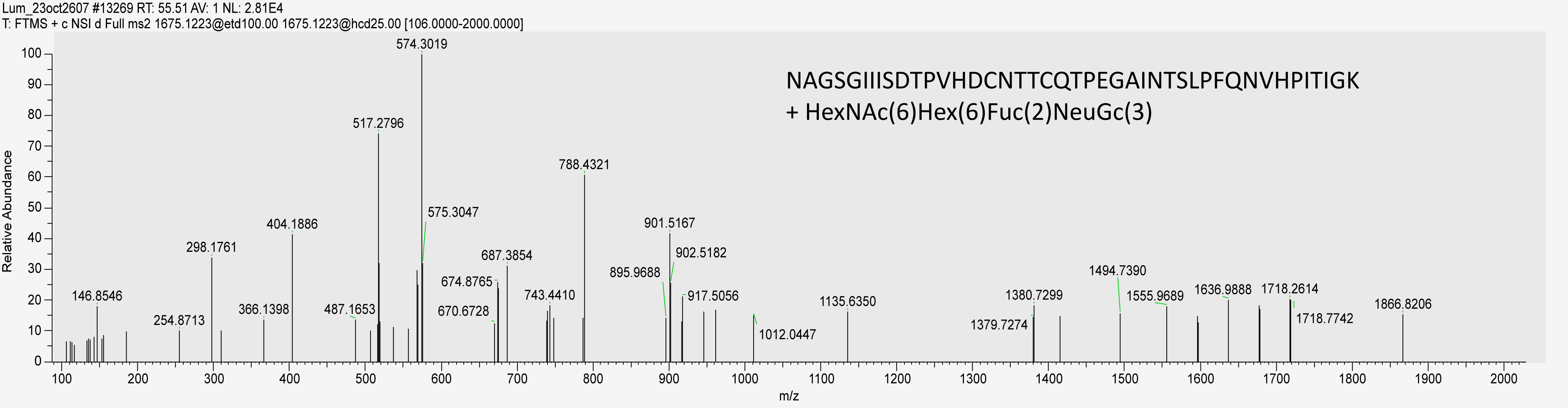
Lum_23oct2607 #13269 RT: 55.51 AV: 1 NL: 2.81E4
T: FTMS + c NSI d Full ms2 1675.1223@etd100.00 1675.1223@hcd25.00 [106.0000-2000.0000]
NAGSGIIISDTPVHDCNTTCQTPEGAINTSLPFQNVHPITIGK
+ HexNAc(6)Hex(6)Fuc(2)NeuGc(3)
Relative Abundance
m/z

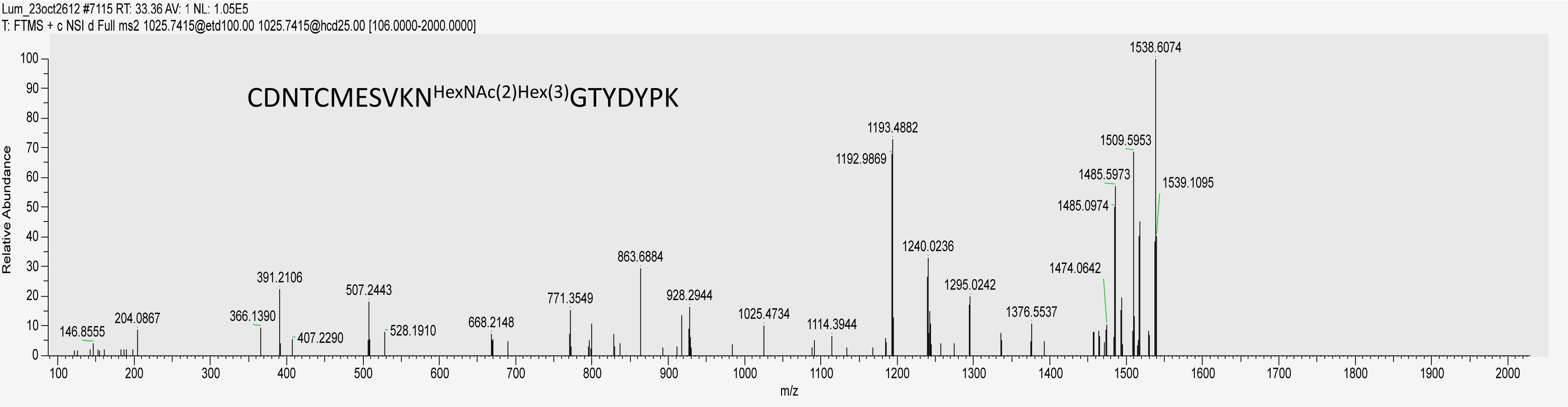
Lum_23oct2612 #7115 RT: 33.36 AV: 1 NL: 1.05E5
T: FTMS + c NSI d Full ms2 1025.7415@etd100.00 1025.7415@hcd25.00 [106.0000-2000.0000]
CDNTCMESVKN HexNAc(2)Hex(3) GTYDYPK
Relative Abundance
m/z
1538.6074
1193.4882
1192.9869
1509.5953
1485.5973
1539.1095
1485.0974
1240.0236
1474.0642
863.6884
1295.0242
391.2106
507.2443
771.3549
928.2944
1376.5537
1025.4734
366.1390
204.0867
668.2148
1114.3944
146.8555
528.1910
407.2290

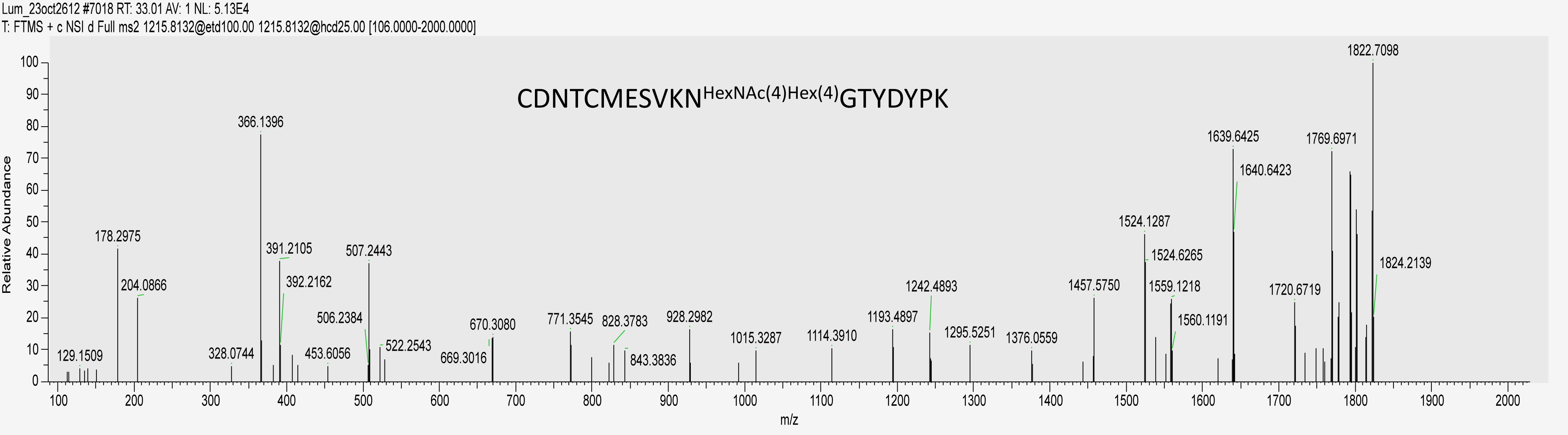
Lum_23oct2612 #7018 RT: 33.01 AV: 1 NL: 5.13E4
T: FTMS + c NSI d Full ms2 1215.8132@etd100.00 1215.8132@hcd25.00 [106.0000-2000.0000]
CDNTCMESVKN HexNAc(4)Hex(4) GTYDYPK
Relative Abundance
m/z
1822.7098
366.1396
1639.6425
1769.6971
1640.6423
1524.1287
178.2975
391.2105
507.2443
1524.6265
1824.2139
204.0866
392.2162
1242.4893
1457.5750
1559.1218
1720.6719
506.2384
1193.4897
1560.1191
670.3080
771.3545
828.3783
928.2982
1015.3287
1114.3910
1295.5251
1376.0559
522.2543
129.1509
328.0744
453.6056
669.3016
843.3836

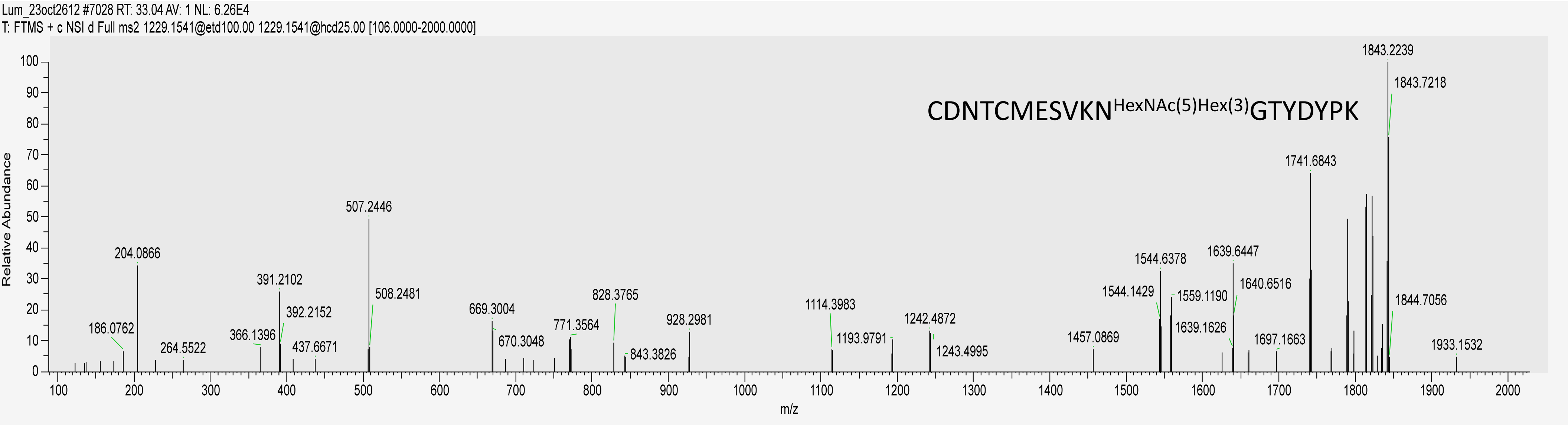
Lum_23oct2612 #7028 RT: 33.04 AV: 1 NL: 6.26E4
T: FTMS + c NSI d Full ms2 1229.1541@etd100.00 1229.1541@hcd25.00 [106.0000-2000.0000]
CDNTCMESVKN HexNAc(5)Hex(3) GTYDYPK
Relative Abundance
m/z
1843.2239
1843.7218
1741.6843
507.2446
204.0866
1544.6378
1639.6447
391.2102
1544.1429
1559.1190
1640.6516
508.2481
828.3765
392.2152
669.3004
1114.3983
1844.7056
186.0762
771.3564
928.2981
1242.4872
670.3048
1457.0869
1639.1626
264.5522
366.1396
437.6671
1193.9791
1697.1663
1933.1532
843.3826
1243.4995

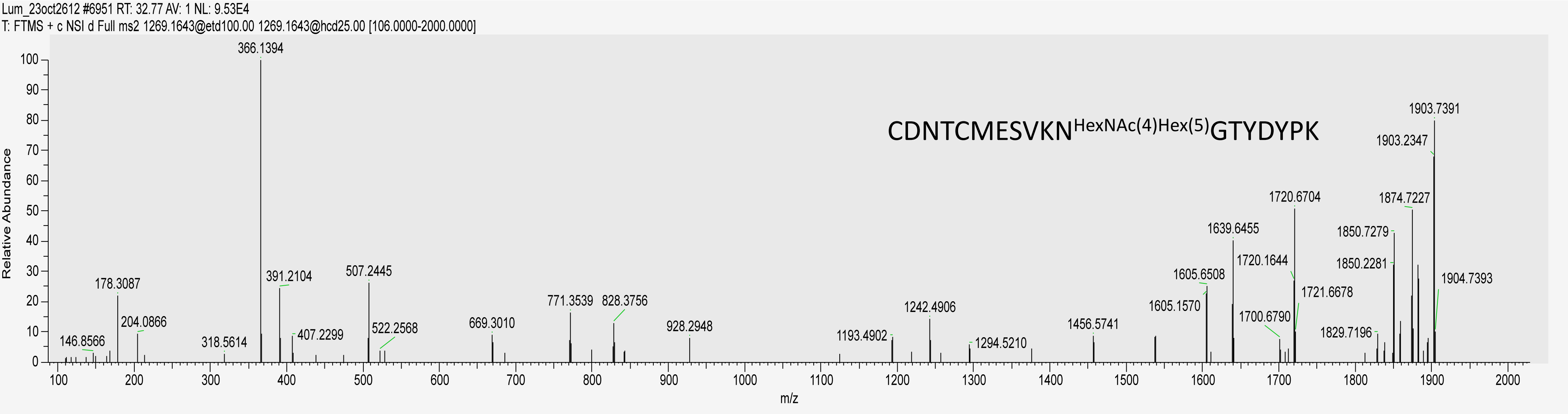
Lum_23oct2612 #6951 RT: 32.77 AV: 1 NL: 9.53E4
T: FTMS + c NSI d Full ms2 1269.1643@etd100.00 1269.1643@hcd25.00 [106.0000-2000.0000]
366.1394
CDNTCMESVKN HexNAc(4)Hex(5) GTYDYPK
Relative Abundance
m/z
1903.7391
1903.2347
1720.6704
1874.7227
1639.6455
1850.7279
1720.1644
1850.2281
1605.6508
391.2104
507.2445
178.3087
1904.7393
1721.6678
1605.1570
771.3539
828.3756
1242.4906
1700.6790
669.3010
1456.5741
204.0866
522.2568
928.2948
407.2299
1829.7196
1193.4902
146.8566
318.5614
1294.5210

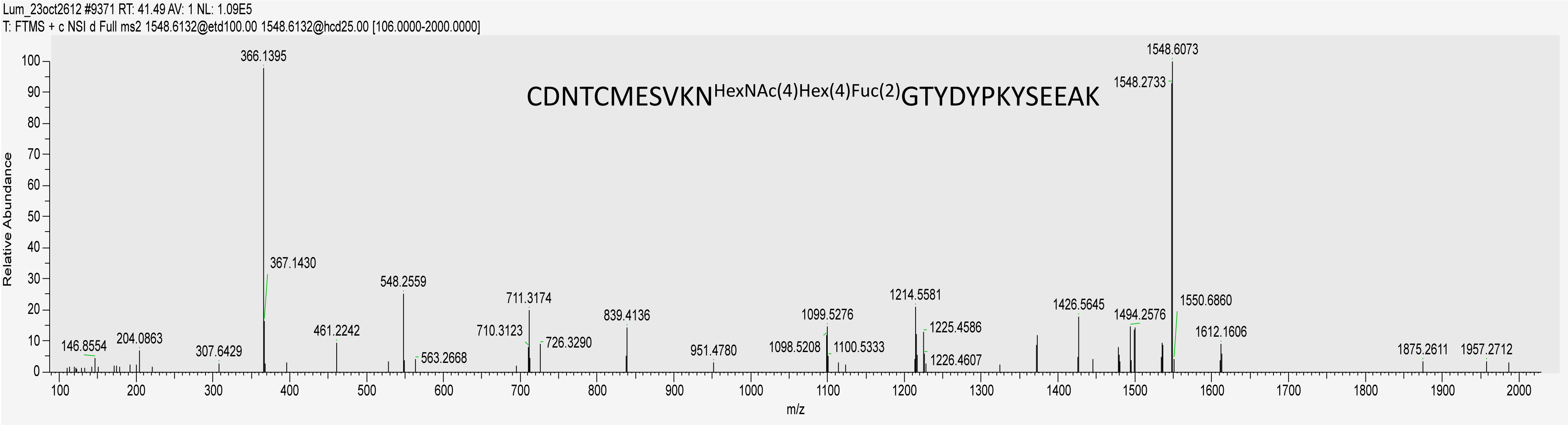
Lum_23oct2612 #9371 RT: 41.49 AV: 1 NL: 1.09E5
T: FTMS + c NSI d Full ms2 1548.6132@etd100.00 1548.6132@hcd25.00 [106.0000-2000.0000]
CDNTCMESVKN$^{HexNAc(4)Hex(4)Fuc(2)}$GTYDYPKYSEEAK
Relative Abundance
m/z
366.1395
367.1430
548.2559
711.3174
710.3123
726.3290
839.4136
146.8554
204.0863
307.6429
461.2242
563.2668
951.4780
1099.5276
1098.5208
1100.5333
1214.5581
1225.4586
1226.4607
1426.5645
1494.2576
1548.6073
1548.2733
1550.6860
1612.1606
1875.2611
1957.2712

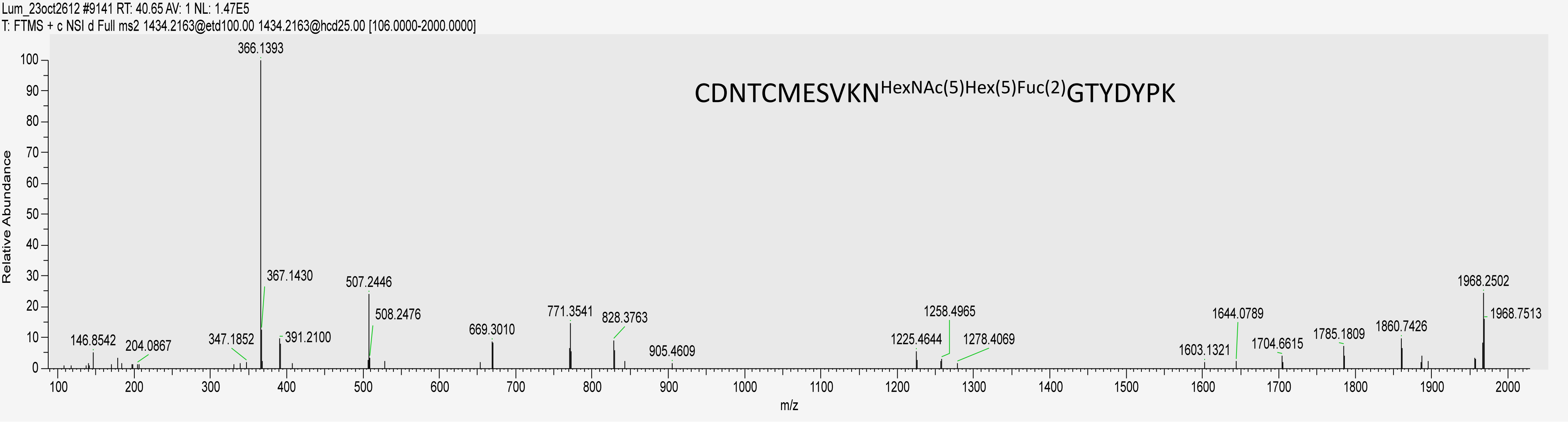
Lum_23oct2612 #9141 RT: 40.65 AV: 1 NL: 1.47E5
T: FTMS + c NSI d Full ms2 1434.2163@etd100.00 1434.2163@hcd25.00 [106.0000-2000.0000]
CDNTCMESVKN$^{HexNAc(5)Hex(5)Fuc(2)}$GTYDYPK
Relative Abundance
m/z
366.1393
367.1430
146.8542
204.0867
347.1852
391.2100
507.2446
508.2476
669.3010
771.3541
828.3763
905.4609
1225.4644
1258.4965
1278.4069
1603.1321
1644.0789
1704.6615
1785.1809
1860.7426
1968.2502
1968.7513

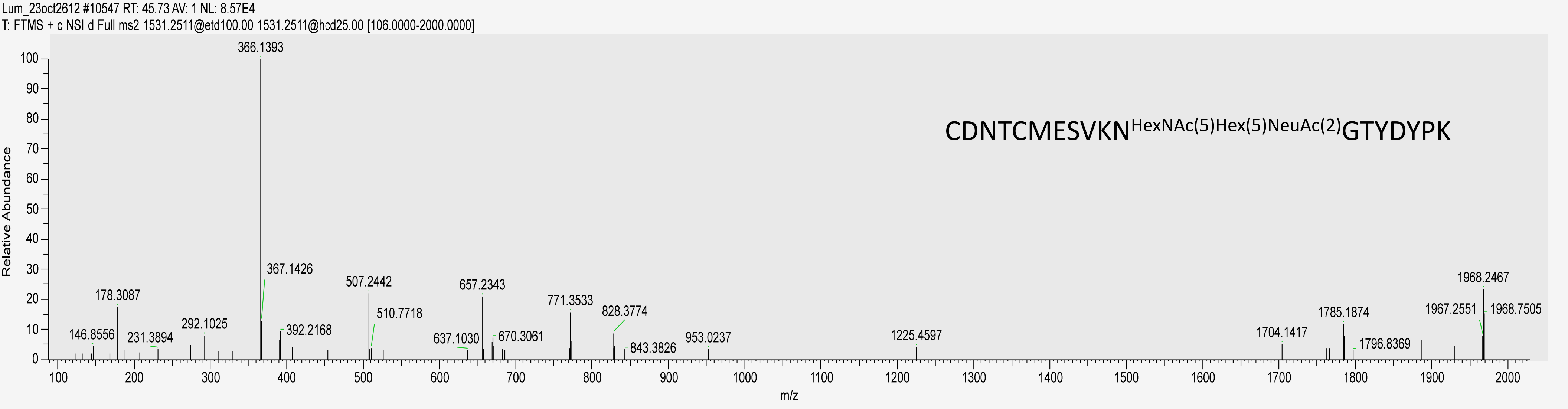
Lum_23oct2612 #10547 RT: 45.73 AV: 1 NL: 8.57E4
T: FTMS + c NSI d Full ms2 1531.2511@etd100.00 1531.2511@hcd25.00 [106.0000-2000.0000]
CDNTCMESVKN HexNAc(5)Hex(5)NeuAc(2) GTYDYPK
366.1393
367.1426
178.3087
146.8556
231.3894
292.1025
392.2168
507.2442
510.7718
637.1030
657.2343
670.3061
771.3533
828.3774
843.3826
953.0237
1225.4597
1704.1417
1785.1874
1796.8369
1967.2551
1968.2467
1968.7505
Relative Abundance
m/z

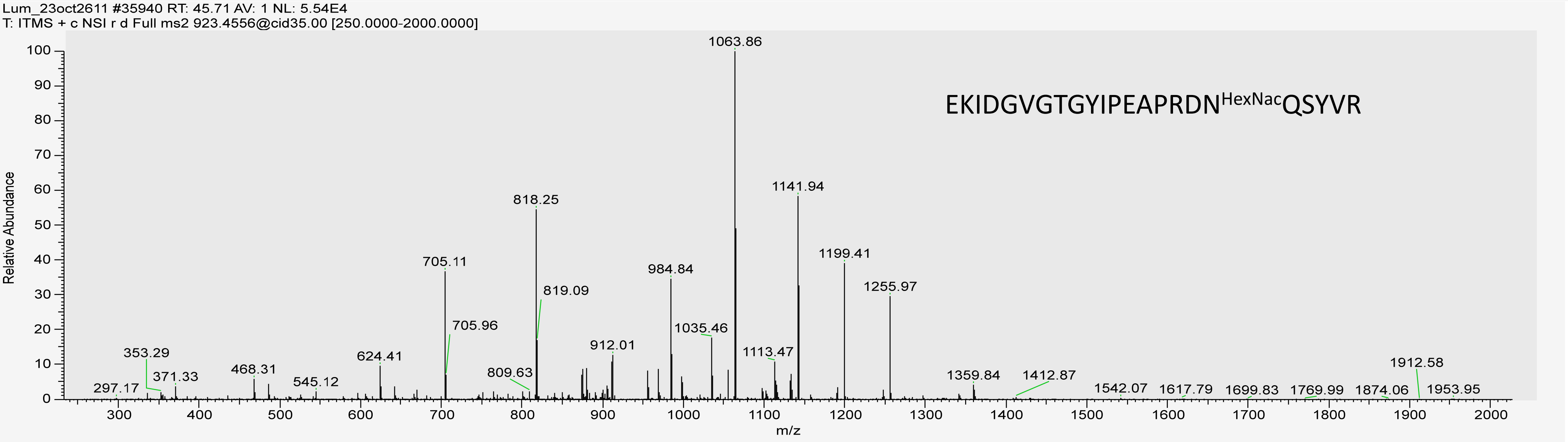
Lum_23oct2611 #35940 RT: 45.71 AV: 1 NL: 5.54E4
T: ITMS + c NSI r d Full ms2 923.4556@cid35.00 [250.0000-2000.0000]
EKIDGVGTGYIPEAPRDN HexNac QSYVR
1063.86
1141.94
818.25
1199.41
1255.97
705.11
984.84
819.09
705.96
1035.46
912.01
1113.47
353.29
624.41
468.31
371.33
545.12
297.17
809.63
1359.84
1412.87
1542.07
1617.79
1699.83
1769.99
1874.06
1912.58
1953.95
Relative Abundance
m/z

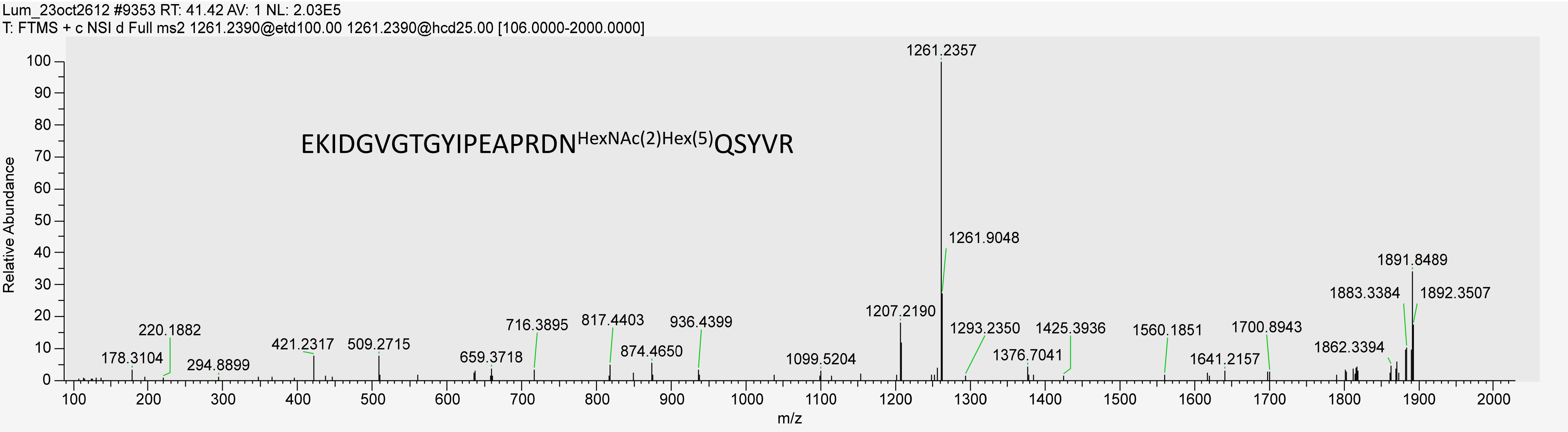
Lum_23oct2612 #9353 RT: 41.42 AV: 1 NL: 2.03E5
T: FTMS + c NSI d Full ms2 1261.2390@etd100.00 1261.2390@hcd25.00 [106.0000-2000.0000]
EKIDGVGTGYIPEAPRDN^HexNAc(2)Hex(5)QSYVR
Relative Abundance
m/z
1261.2357
1261.9048
1891.8489
1883.3384
1892.3507
1207.2190
1293.2350
1425.3936
1560.1851
1700.8943
1862.3394
1376.7041
1641.2157
1099.5204
936.4399
874.4650
817.4403
716.3895
659.3718
509.2715
421.2317
294.8899
220.1882
178.3104

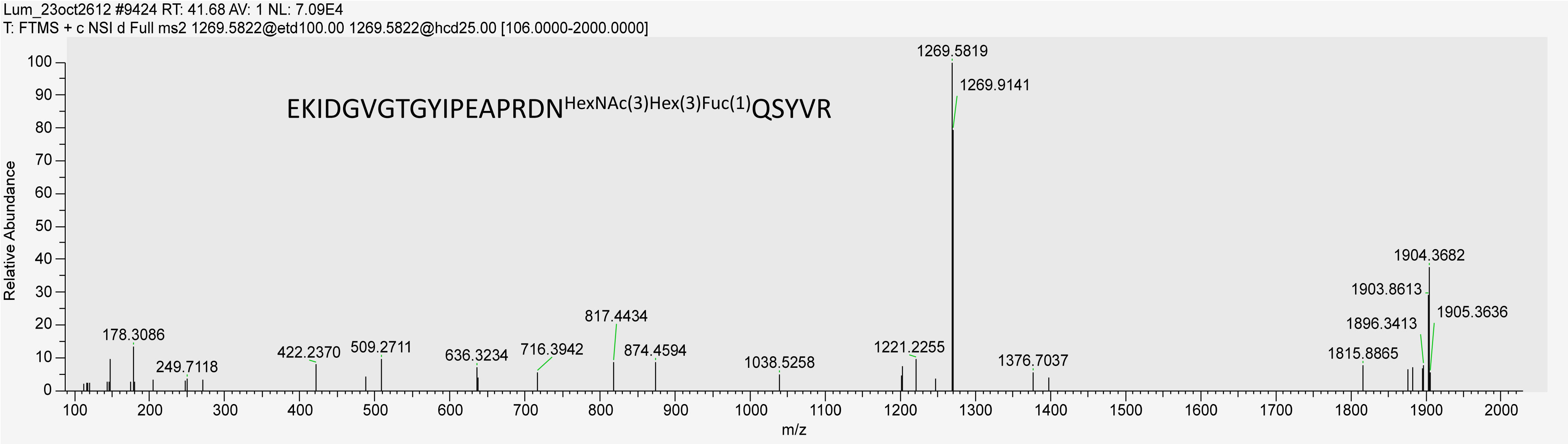
Lum_23oct2612 #9424 RT: 41.68 AV: 1 NL: 7.09E4
T: FTMS + c NSI d Full ms2 1269.5822@etd100.00 1269.5822@hcd25.00 [106.0000-2000.0000]
EKIDGVGTGYIPEAPRDN^HexNAc(3)Hex(3)Fuc(1)QSYVR
Relative Abundance
m/z
1269.5819
1269.9141
1904.3682
1903.8613
1905.3636
1896.3413
1815.8865
1376.7037
1221.2255
1038.5258
874.4594
817.4434
716.3942
636.3234
509.2711
422.2370
249.7118
178.3086

Lum_23oct2612 #9398 RT: 41.58 AV: 1 NL: 1.16E5
T: FTMS + c NSI d Full ms2 1337.6085@etd100.00 1337.6085@hcd25.00 [106.0000-2000.0000]

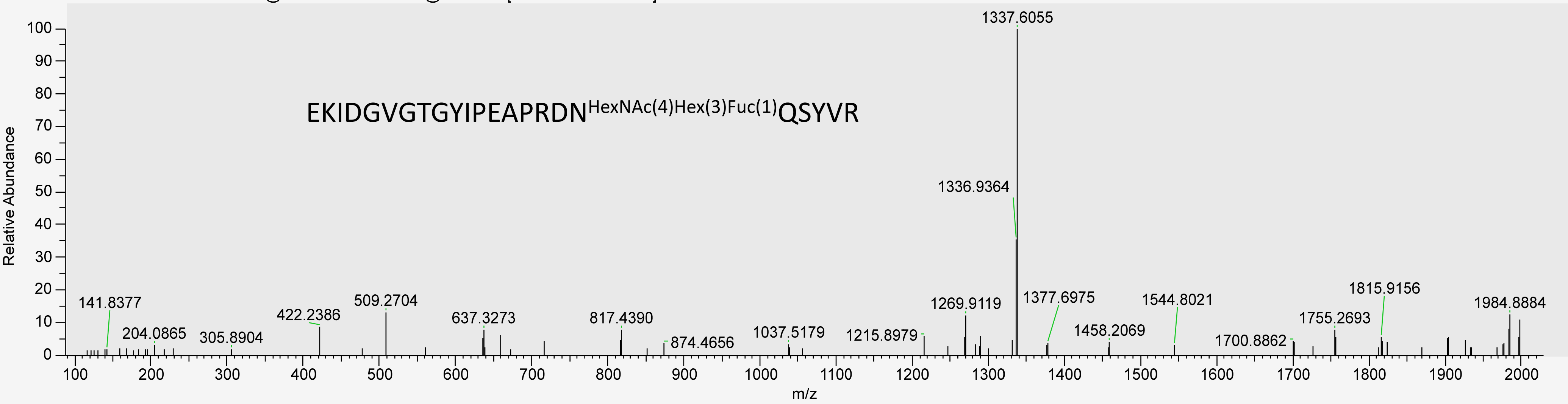

Lum_23oct2612 #9349 RT: 41.41 AV: 1 NL: 1.30E5
T: FTMS + c NSI d Full ms2 1391.2935@etd100.00 1391.2935@hcd25.00 [106.0000-2000.0000]

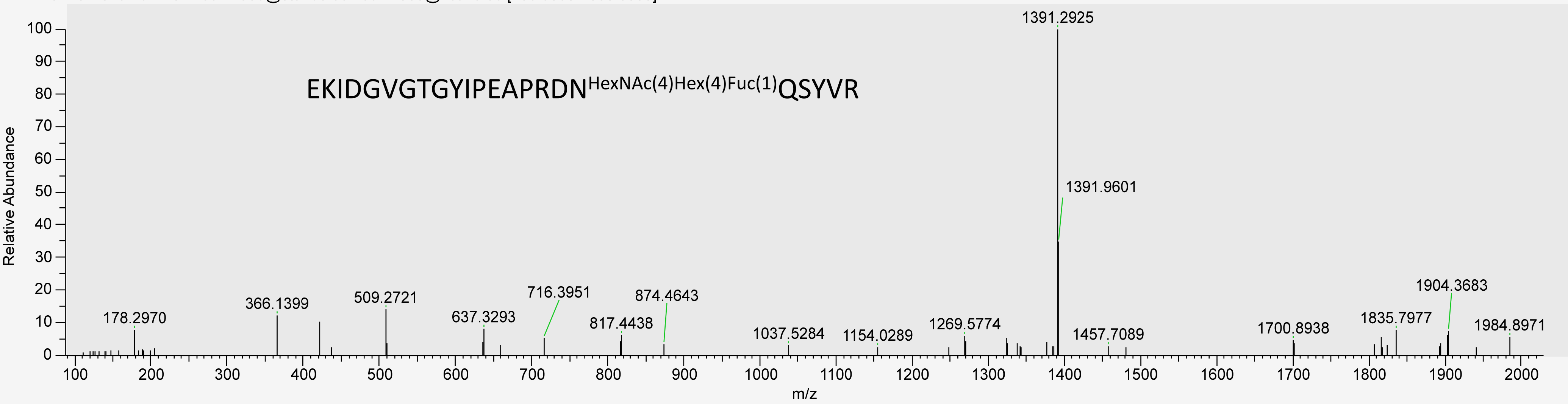

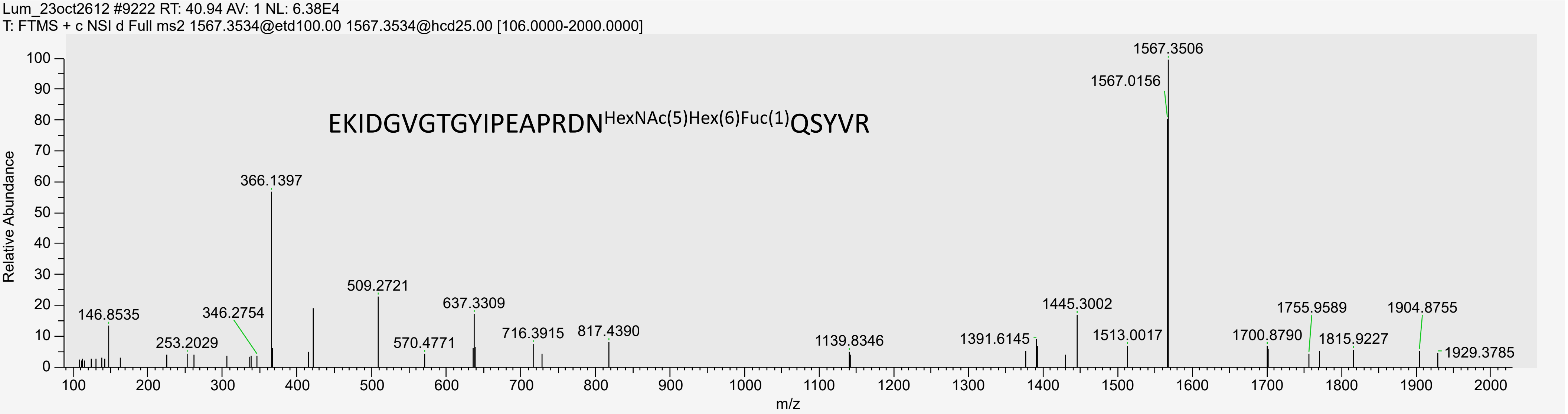
Lum_23oct2612 #9222 RT: 40.94 AV: 1 NL: 6.38E4
T: FTMS + c NSI d Full ms2 1567.3534@etd100.00 1567.3534@hcd25.00 [106.0000-2000.0000]
EKIDGVGTGYIPEAPRDNHexNAc(5)Hex(6)Fuc(1)QSYVR
Relative Abundance
m/z
1567.3506
1567.0156
366.1397
509.2721
637.3309
1445.3002
146.8535
346.2754
253.2029
570.4771
716.3915
817.4390
1139.8346
1391.6145
1513.0017
1755.9589
1904.8755
1700.8790
1815.9227
1929.3785

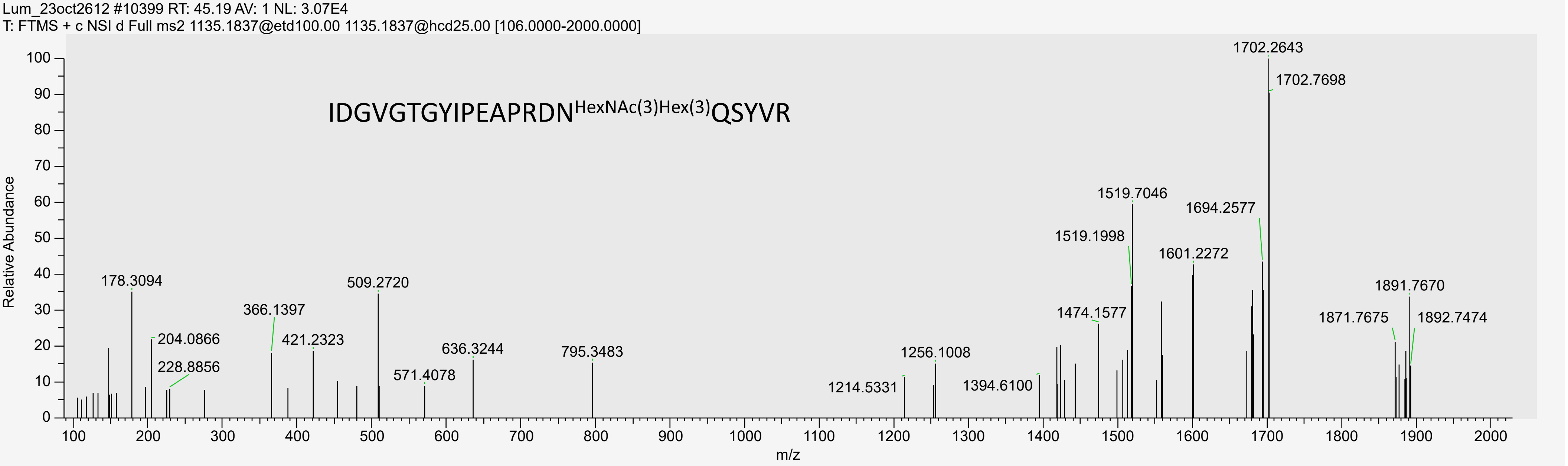
Lum_23oct2612 #10399 RT: 45.19 AV: 1 NL: 3.07E4
T: FTMS + c NSI d Full ms2 1135.1837@etd100.00 1135.1837@hcd25.00 [106.0000-2000.0000]
IDGVGTGYIPEAPRDNHexNAc(3)Hex(3)QSYVR
Relative Abundance
m/z
1702.2643
1702.7698
1519.7046
1694.2577
1519.1998
1601.2272
178.3094
509.2720
1891.7670
366.1397
1474.1577
1871.7675
1892.7474
204.0866
421.2323
636.3244
795.3483
1256.1008
228.8856
571.4078
1214.5331
1394.6100

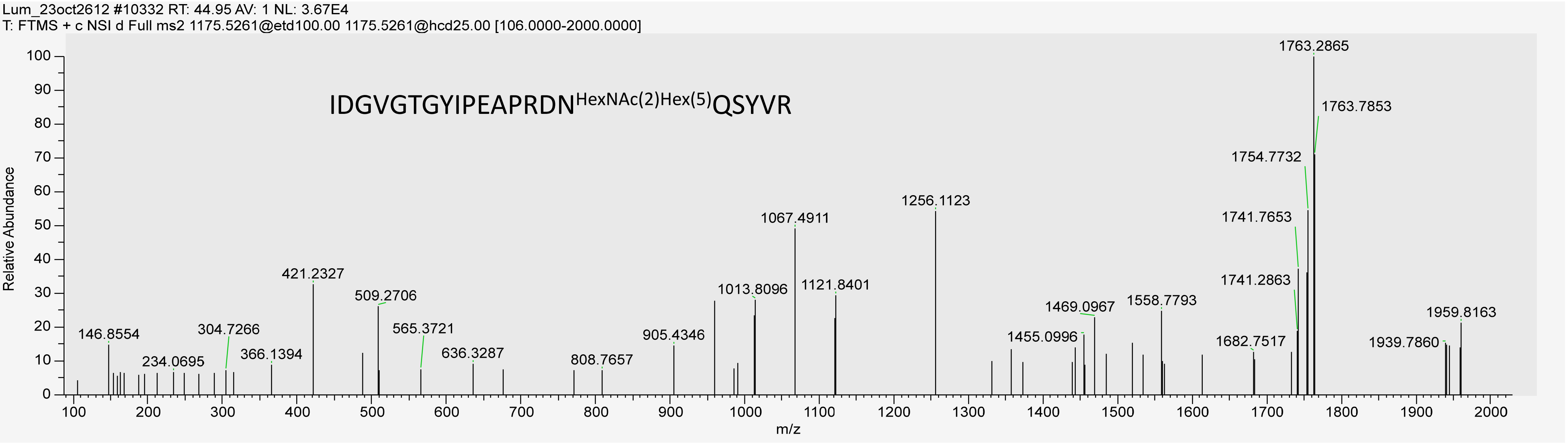
Lum_23oct2612 #10332 RT: 44.95 AV: 1 NL: 3.67E4
T: FTMS + c NSI d Full ms2 1175.5261@etd100.00 1175.5261@hcd25.00 [106.0000-2000.0000]
IDGVGTGYIPEAPRDN HexNAc(2)Hex(5) QSYVR
Relative Abundance
m/z
1763.2865
1763.7853
1754.7732
1741.7653
1741.2863
1256.1123
1067.4911
1121.8401
1013.8096
421.2327
509.2706
565.3721
304.7266
146.8554
366.1394
234.0695
636.3287
808.7657
905.4346
1469.0967
1455.0996
1558.7793
1682.7517
1959.8163
1939.7860

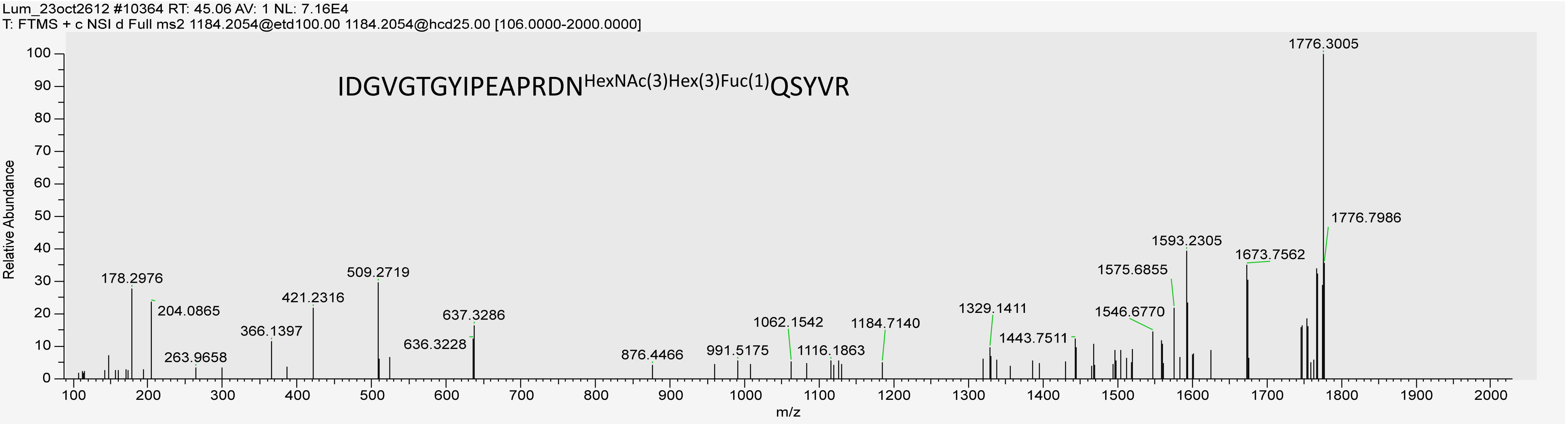
Lum_23oct2612 #10364 RT: 45.06 AV: 1 NL: 7.16E4
T: FTMS + c NSI d Full ms2 1184.2054@etd100.00 1184.2054@hcd25.00 [106.0000-2000.0000]
IDGVGTGYIPEAPRDN HexNAc(3)Hex(3)Fuc(1) QSYVR
Relative Abundance
m/z
1776.3005
1776.7986
1593.2305
1673.7562
1575.6855
1546.6770
1329.1411
1443.7511
1184.7140
1062.1542
1116.1863
991.5175
876.4466
637.3286
636.3228
509.2719
421.2316
366.1397
178.2976
204.0865
263.9658

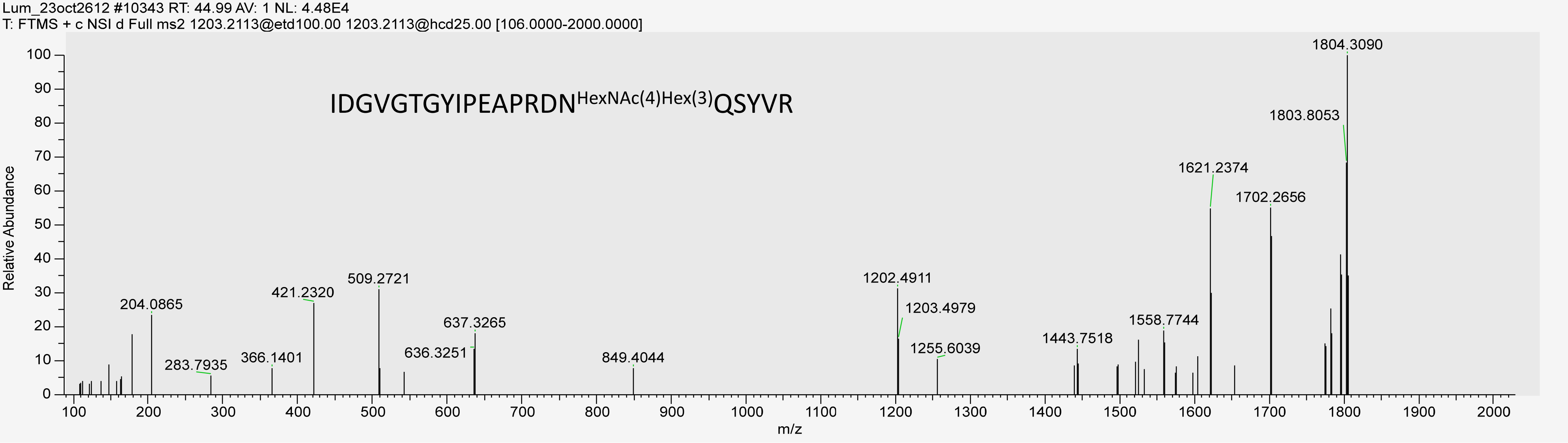
Lum_23oct2612 #10343 RT: 44.99 AV: 1 NL: 4.48E4
T: FTMS + c NSI d Full ms2 1203.2113@etd100.00 1203.2113@hcd25.00 [106.0000-2000.0000]
IDGVGTGYIPEAPRDNHexNAc(4)Hex(3)QSYVR
Relative Abundance
m/z
1804.3090
1803.8053
1621.2374
1702.2656
1202.4911
1203.4979
1255.6039
1443.7518
1558.7744
509.2721
421.2320
204.0865
637.3265
636.3251
366.1401
283.7935
849.4044

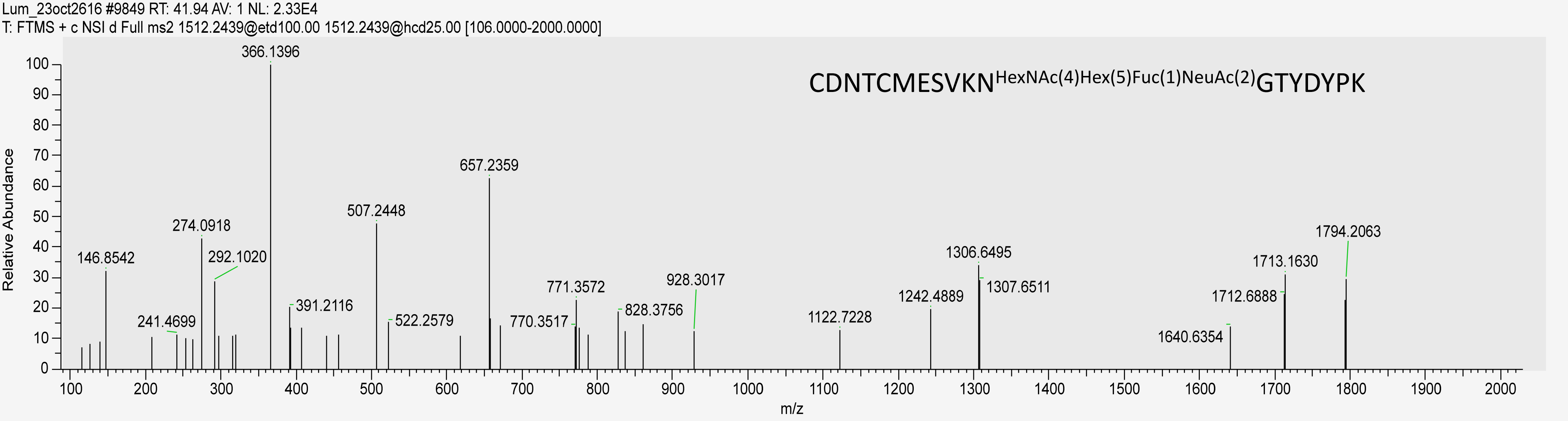
Lum_23oct2616 #9849 RT: 41.94 AV: 1 NL: 2.33E4
T: FTMS + c NSI d Full ms2 1512.2439@etd100.00 1512.2439@hcd25.00 [106.0000-2000.0000]
CDNTCMESVKNHexNAc(4)Hex(5)Fuc(1)NeuAc(2)GTYDYPK
Relative Abundance
m/z
366.1396
657.2359
507.2448
274.0918
146.8542
292.1020
241.4699
391.2116
522.2579
771.3572
770.3517
828.3756
928.3017
1122.7228
1242.4889
1306.6495
1307.6511
1640.6354
1712.6888
1713.1630
1794.2063

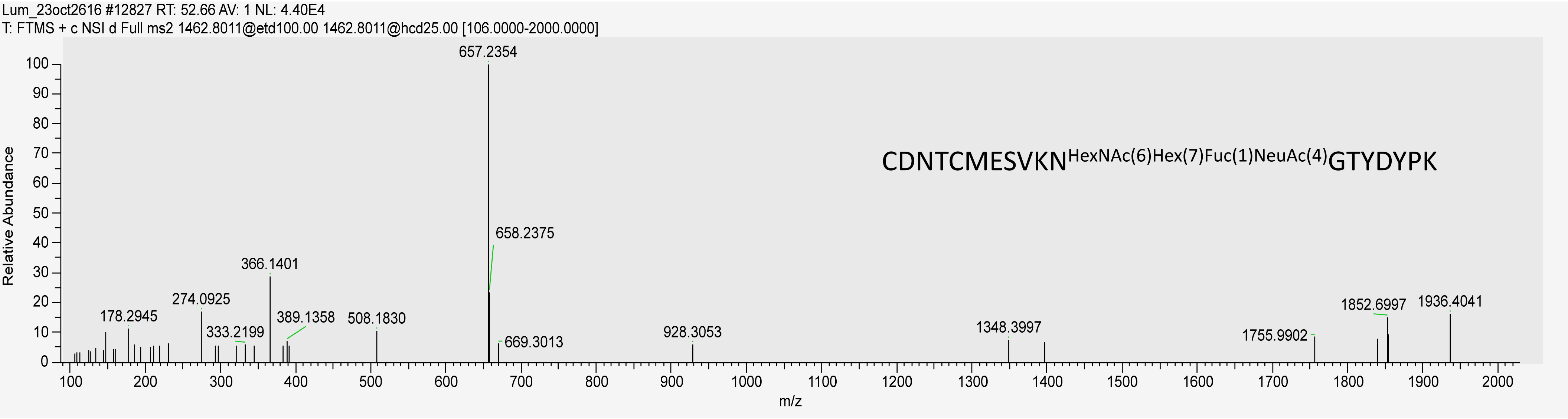
Lum_23oct2616 #12827 RT: 52.66 AV: 1 NL: 4.40E4
T: FTMS + c NSI d Full ms2 1462.8011@etd100.00 1462.8011@hcd25.00 [106.0000-2000.0000]
CDNTCMESVKN HexNAc(6)Hex(7)Fuc(1)NeuAc(4) GTYDYPK
Relative Abundance
m/z
657.2354
658.2375
366.1401
274.0925
178.2945
333.2199
389.1358
508.1830
669.3013
928.3053
1348.3997
1755.9902
1852.6997
1936.4041

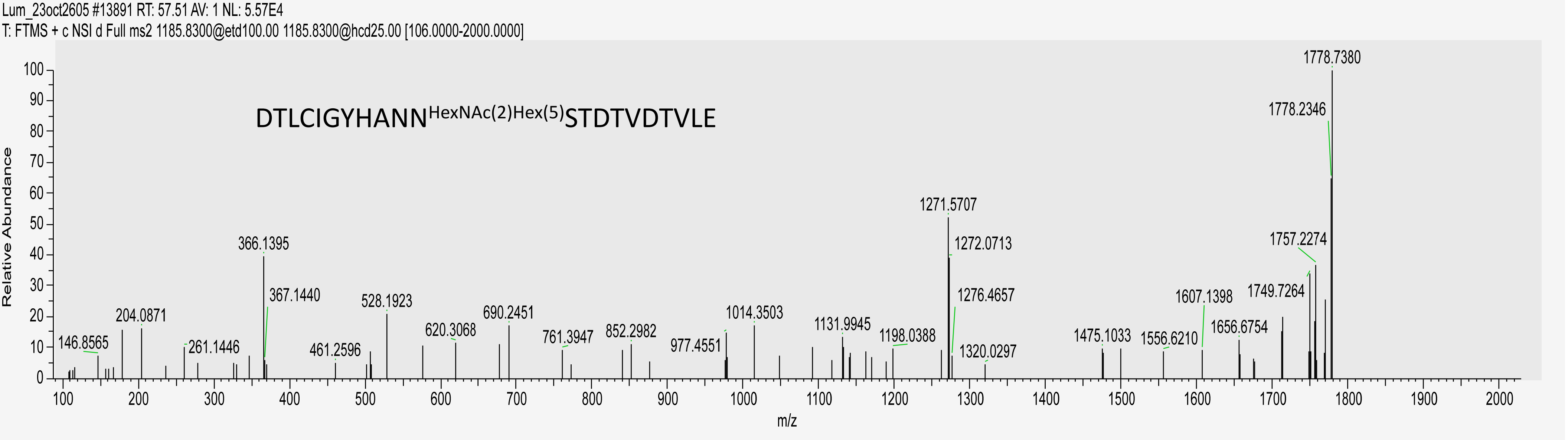
Lum_23oct2605 #13891 RT: 57.51 AV: 1 NL: 5.57E4
T: FTMS + c NSI d Full ms2 1185.8300@etd100.00 1185.8300@hcd25.00 [106.0000-2000.0000]
DTLCIGYHANN HexNAc(2)Hex(5) STDTVDTVLE
Relative Abundance
m/z
1778.7380
1778.2346
1271.5707
1272.0713
1757.2274
366.1395
367.1440
1276.4657
1749.7264
1607.1398
204.0871
528.1923
690.2451
1014.3503
1131.9945
1656.6754
146.8565
261.1446
461.2596
620.3068
761.3947
852.2982
977.4551
1198.0388
1320.0297
1475.1033
1556.6210

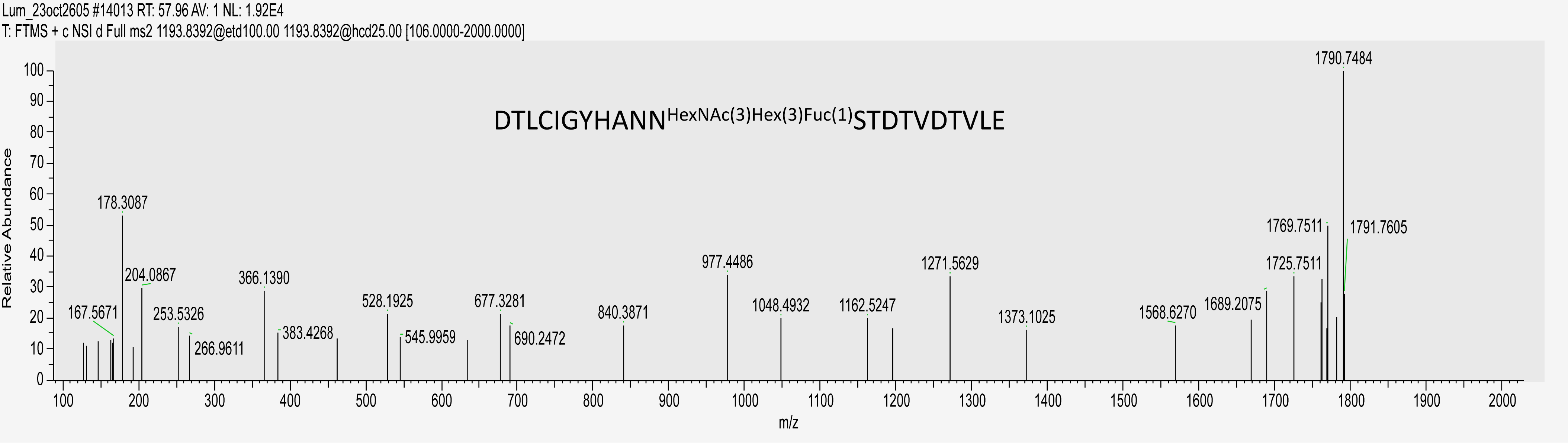
Lum_23oct2605 #14013 RT: 57.96 AV: 1 NL: 1.92E4
T: FTMS + c NSI d Full ms2 1193.8392@etd100.00 1193.8392@hcd25.00 [106.0000-2000.0000]
DTLCIGYHANN HexNAc(3)Hex(3)Fuc(1) STDTVDTVLE
Relative Abundance
m/z
1790.7484
178.3087
1769.7511
1791.7605
1725.7511
977.4486
1271.5629
204.0867
366.1390
167.5671
253.5326
266.9611
383.4268
528.1925
545.9959
677.3281
690.2472
840.3871
1048.4932
1162.5247
1373.1025
1568.6270
1689.2075

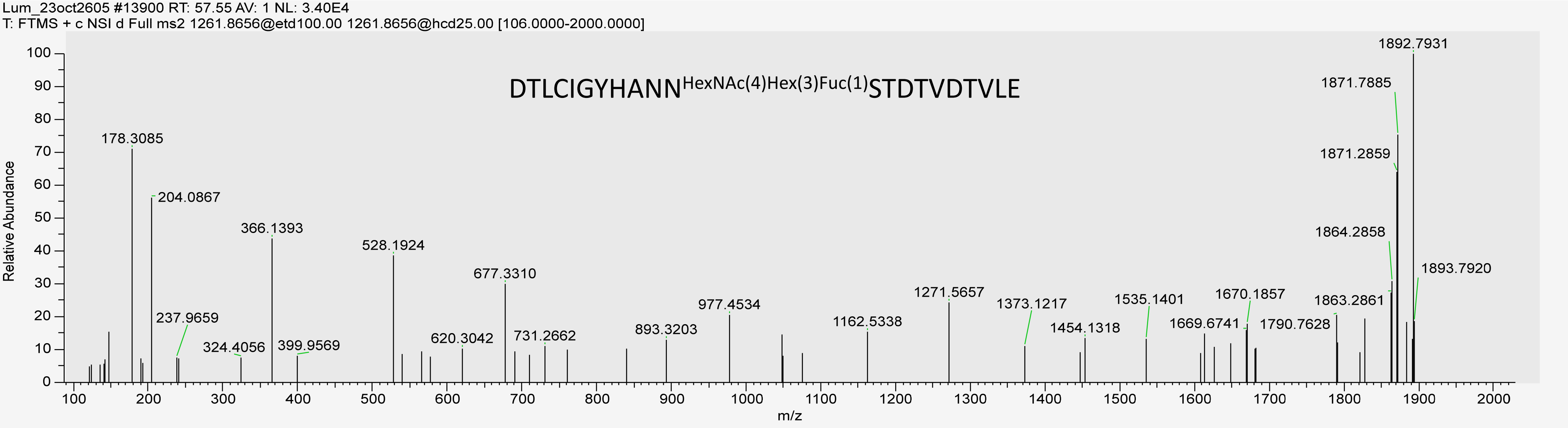
Lum_23oct2605 #13900 RT: 57.55 AV: 1 NL: 3.40E4
T: FTMS + c NSI d Full ms2 1261.8656@etd100.00 1261.8656@hcd25.00 [106.0000-2000.0000]
DTLCIGYHANN HexNAc(4)Hex(3)Fuc(1) STDTVDTVLE
Relative Abundance
m/z
1892.7931
1871.7885
1871.2859
178.3085
204.0867
366.1393
528.1924
1864.2858
1893.7920
677.3310
1271.5657
977.4534
1373.1217
1535.1401
1670.1857
1863.2861
237.9659
1162.5338
1454.1318
1669.6741
1790.7628
620.3042
731.2662
893.3203
324.4056
399.9569

Lum_23oct2605 #13855 RT: 57.38 AV: 1 NL: 2.67E4
T: FTMS + c NSI d Full ms2 1329.8933@etd100.00 1329.8933@hcd25.00 [106.0000-2000.0000]

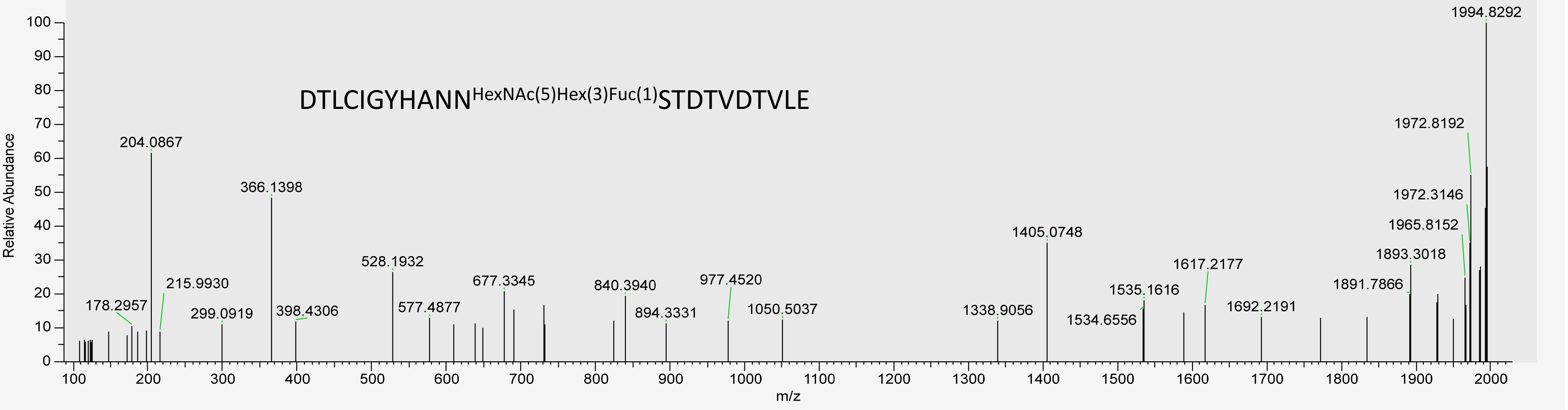

Lum_23oct2605 #7408 RT: 34.14 AV: 1 NL: 2.00E5
T: FTMS + c NSI d Full ms2 783.0444@etd100.00 783.0444@hcd25.00 [106.0000-2000.0000]

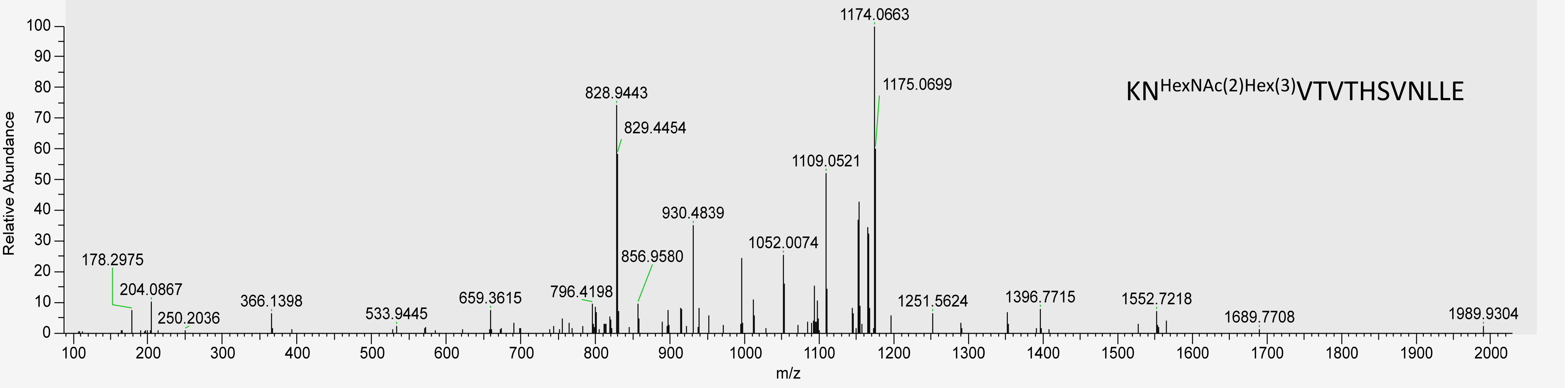

Lum_23oct2605 #7493 RT: 34.44 AV: 1 NL: 1.40E5
T: FTMS + c NSI d Full ms2 832.0634@etd100.00 832.0634@hcd25.00 [106.0000-2000.0000]

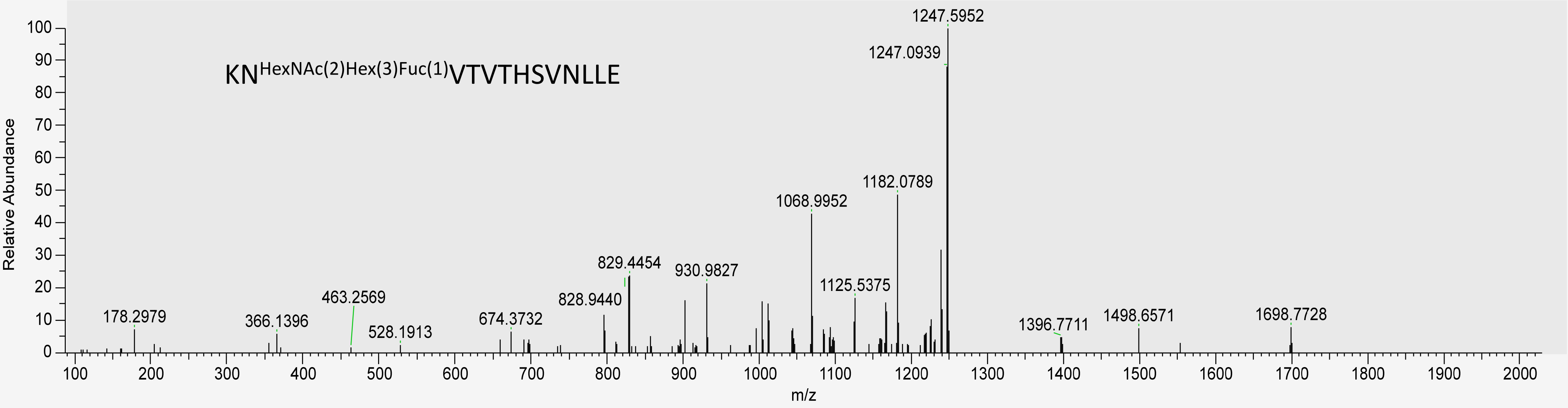

Lum_23oct2605 #7222 RT: 33.48 AV: 1 NL: 5.41E5
T: FTMS + c NSI d Full ms2 837.3983@etd100.00 837.3983@hcd25.00 [106.0000-2000.0000]

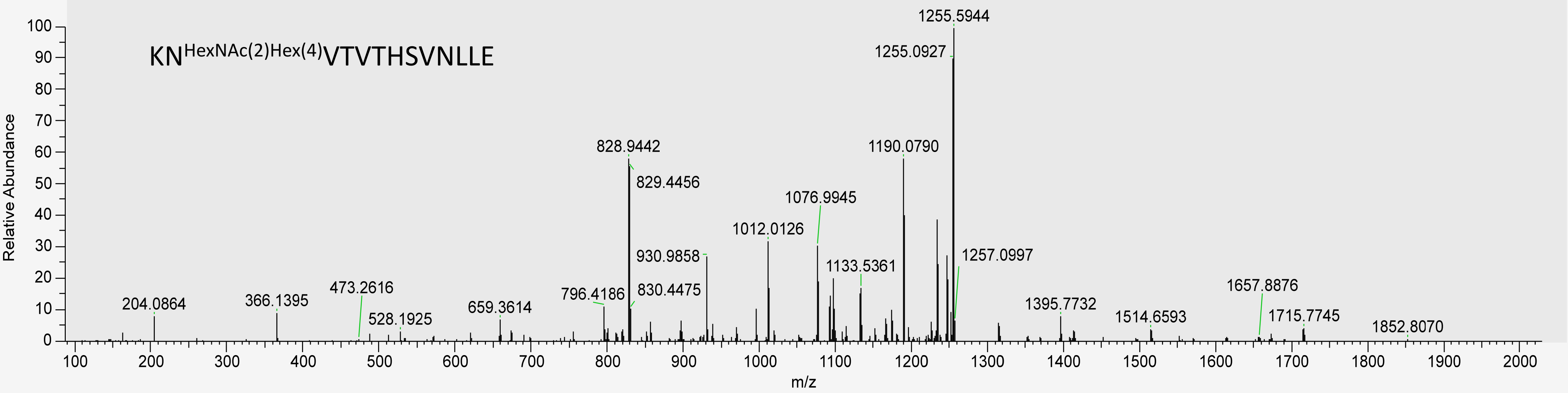

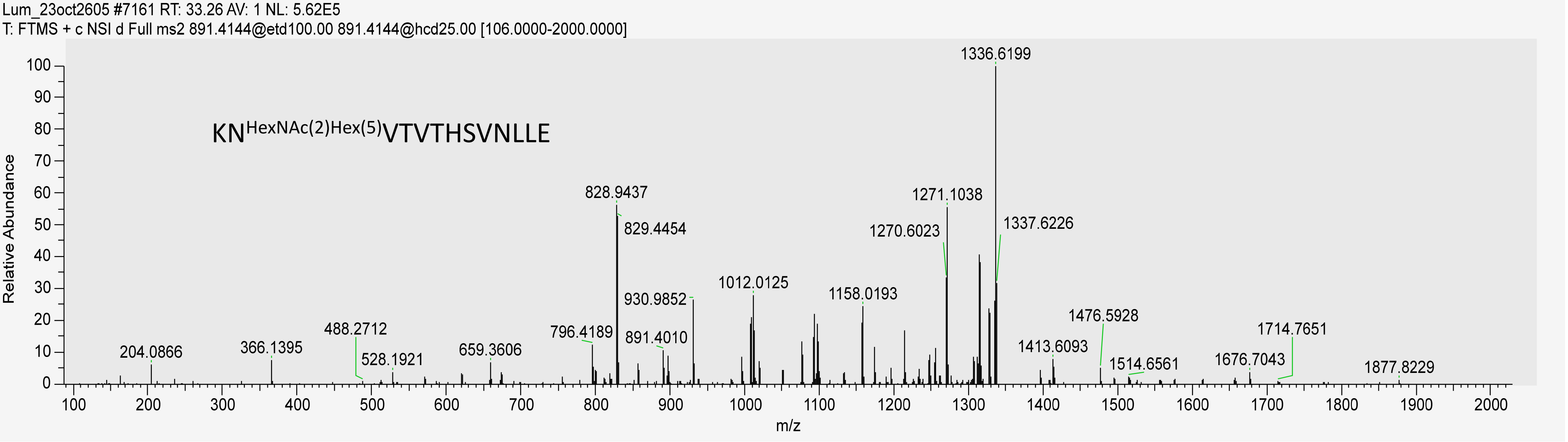
Lum_23oct2605 #7161 RT: 33.26 AV: 1 NL: 5.62E5
T: FTMS + c NSI d Full ms2 891.4144@etd100.00 891.4144@hcd25.00 [106.0000-2000.0000]
KN^HexNAc(2)Hex(5)VTVTHSVNLLE
Relative Abundance
m/z
1336.6199
828.9437
829.4454
1271.1038
1270.6023
1337.6226
1012.0125
1158.0193
930.9852
1476.5928
488.2712
1714.7651
796.4189
891.4010
1413.6093
204.0866
366.1395
659.3606
528.1921
1514.6561
1676.7043
1877.8229

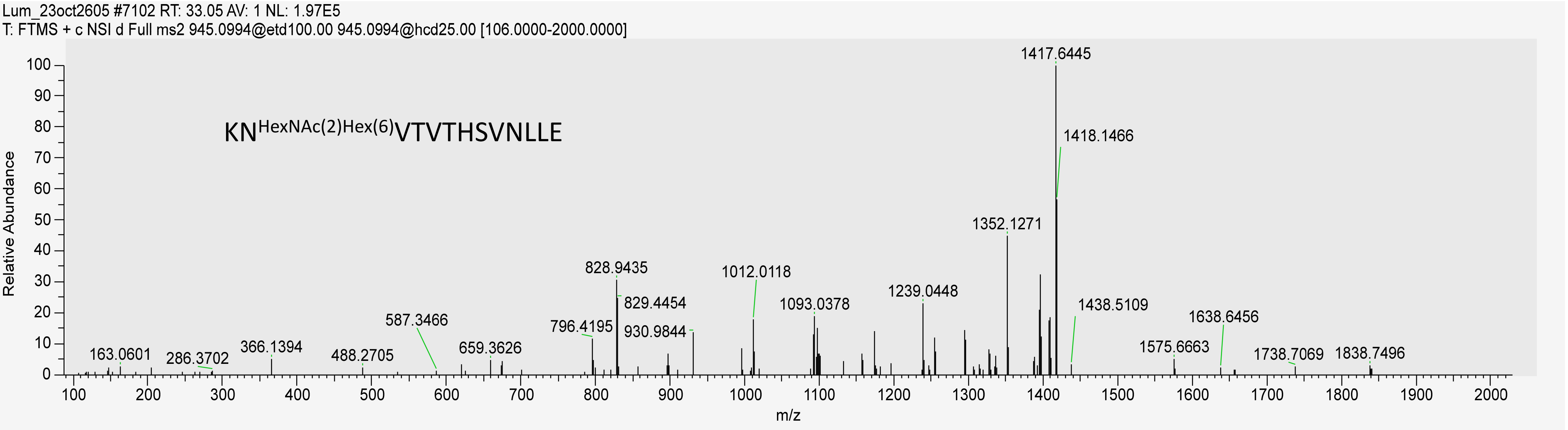
Lum_23oct2605 #7102 RT: 33.05 AV: 1 NL: 1.97E5
T: FTMS + c NSI d Full ms2 945.0994@etd100.00 945.0994@hcd25.00 [106.0000-2000.0000]
KN^HexNAc(2)Hex(6)VTVTHSVNLLE
Relative Abundance
m/z
1417.6445
1418.1466
1352.1271
828.9435
1012.0118
1239.0448
829.4454
1093.0378
1438.5109
587.3466
930.9844
1638.6456
796.4195
366.1394
659.3626
1575.6663
163.0601
286.3702
488.2705
1738.7069
1838.7496

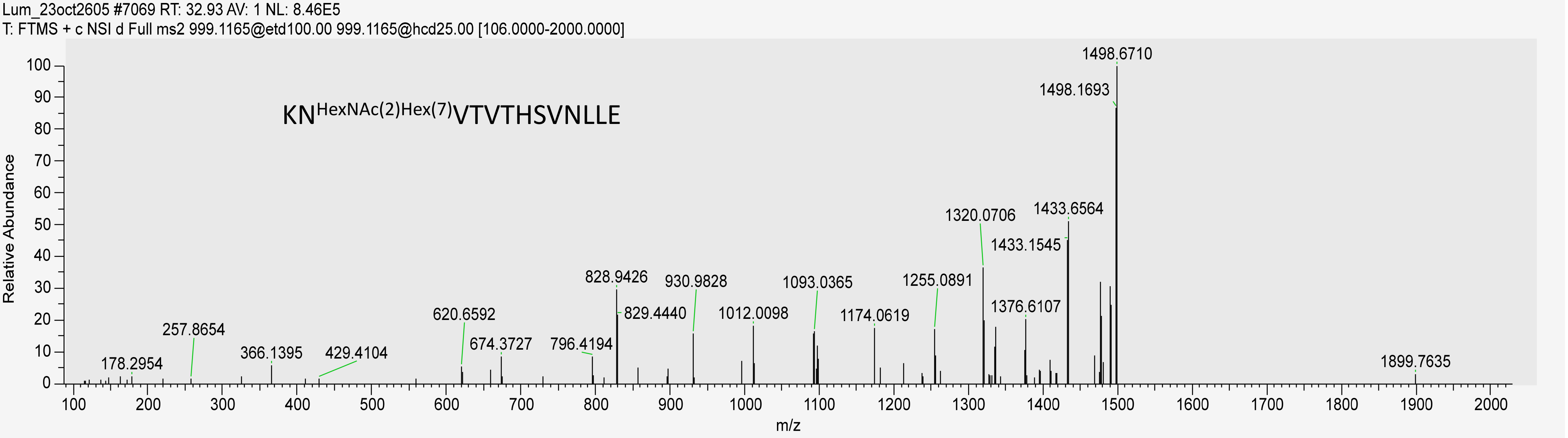
Lum_23oct2605 #7069 RT: 32.93 AV: 1 NL: 8.46E5
T: FTMS + c NSI d Full ms2 999.1165@etd100.00 999.1165@hcd25.00 [106.0000-2000.0000]
KN^HexNAc(2)Hex(7)VTVTHSVNLLE
Relative Abundance
m/z
1498.6710
1498.1693
1433.6564
1433.1545
1320.0706
1376.6107
1255.0891
1174.0619
1093.0365
1012.0098
930.9828
828.9426
829.4440
796.4194
674.3727
620.6592
429.4104
366.1395
257.8654
178.2954
1899.7635

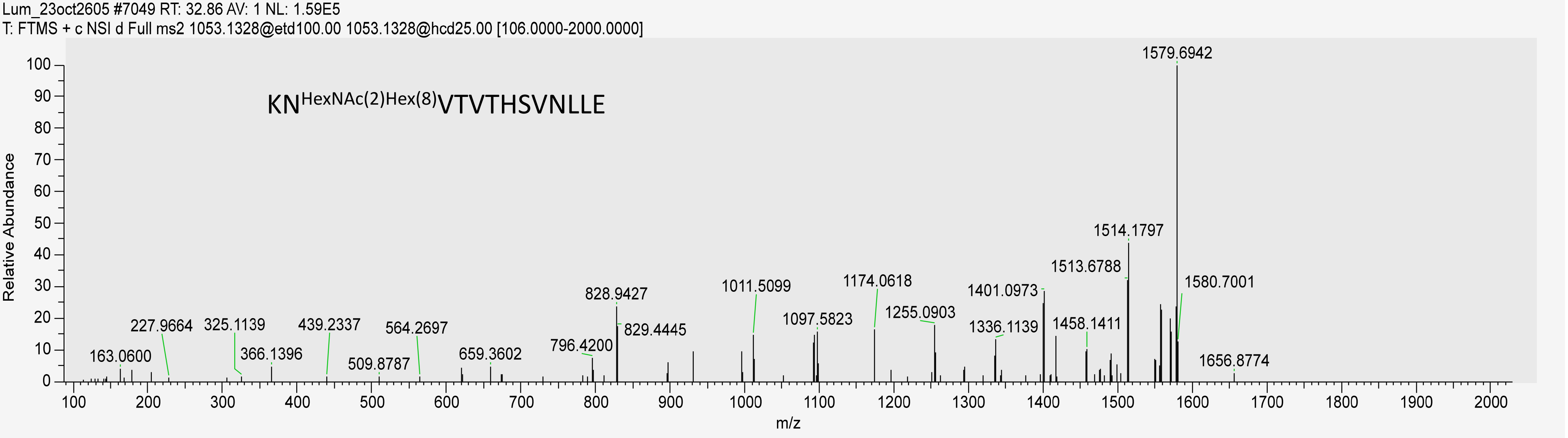
Lum_23oct2605 #7049 RT: 32.86 AV: 1 NL: 1.59E5
T: FTMS + c NSI d Full ms2 1053.1328@etd100.00 1053.1328@hcd25.00 [106.0000-2000.0000]
KN^HexNAc(2)Hex(8)VTVTHSVNLLE
Relative Abundance
m/z
1579.6942
1514.1797
1513.6788
1580.7001
1401.0973
1174.0618
1011.5099
828.9427
829.4445
1255.0903
1097.5823
1336.1139
1458.1411
1656.8774
796.4200
659.3602
564.2697
509.8787
439.2337
366.1396
325.1139
227.9664
163.0600

Lum_23oct2605 #7347 RT: 33.92 AV: 1 NL: 2.71E5
T: FTMS + c NSI d Full ms2 850.7389@etd100.00 850.7389@hcd25.00 [106.0000-2000.0000]

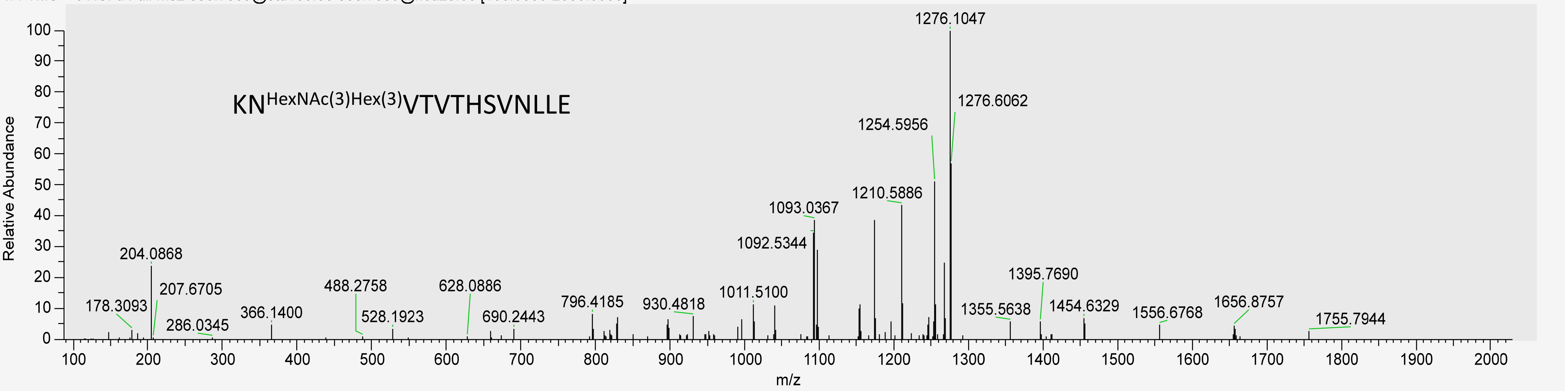

Lum_23oct2605 #7414 RT: 34.16 AV: 1 NL: 3.03E5
T: FTMS + c NSI d Full ms2 899.4233@etd100.00 899.4233@hcd25.00 [106.0000-2000.0000]

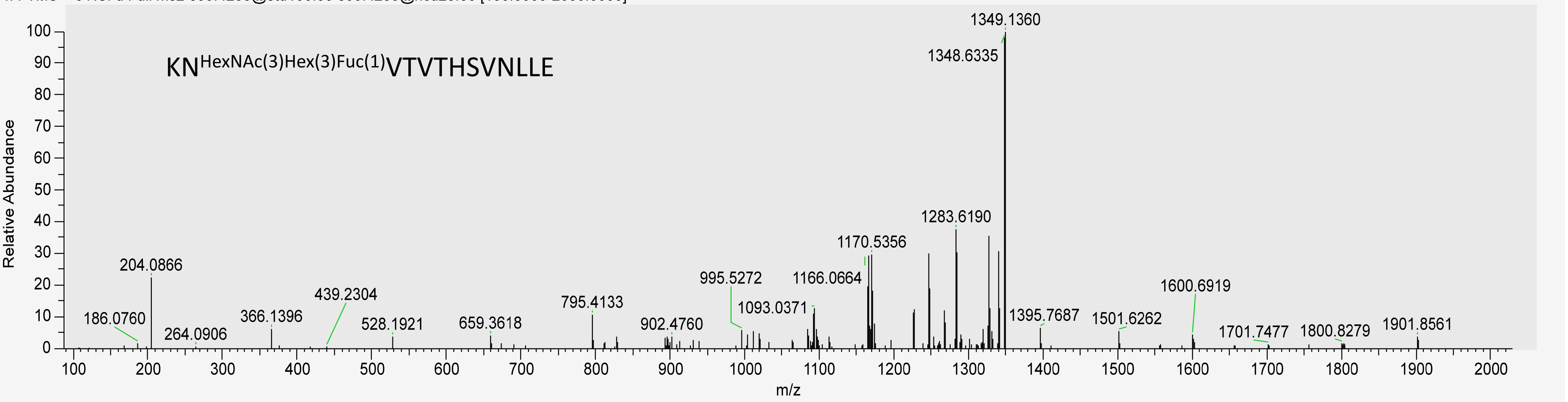

Lum_23oct2605 #7272 RT: 33.66 AV: 1 NL: 1.02E5
T: FTMS + c NSI d Full ms2 904.7573@etd100.00 904.7573@hcd25.00 [106.0000-2000.0000]

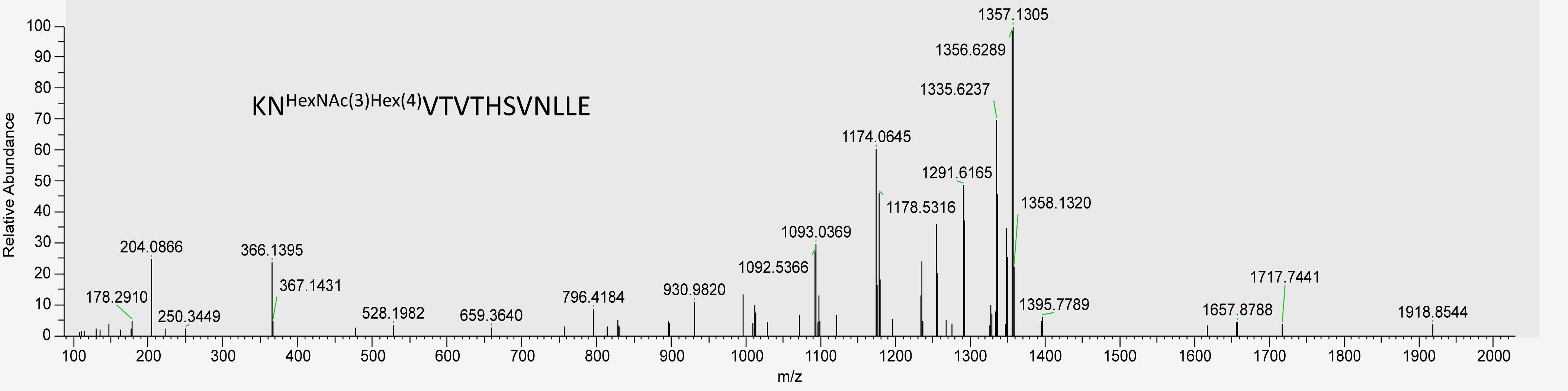

Lum_23oct2605 #7334 RT: 33.88 AV: 1 NL: 7.10E4
T: FTMS + c NSI d Full ms2 953.4410@etd100.00 953.4410@hcd25.00 [106.0000-2000.0000]

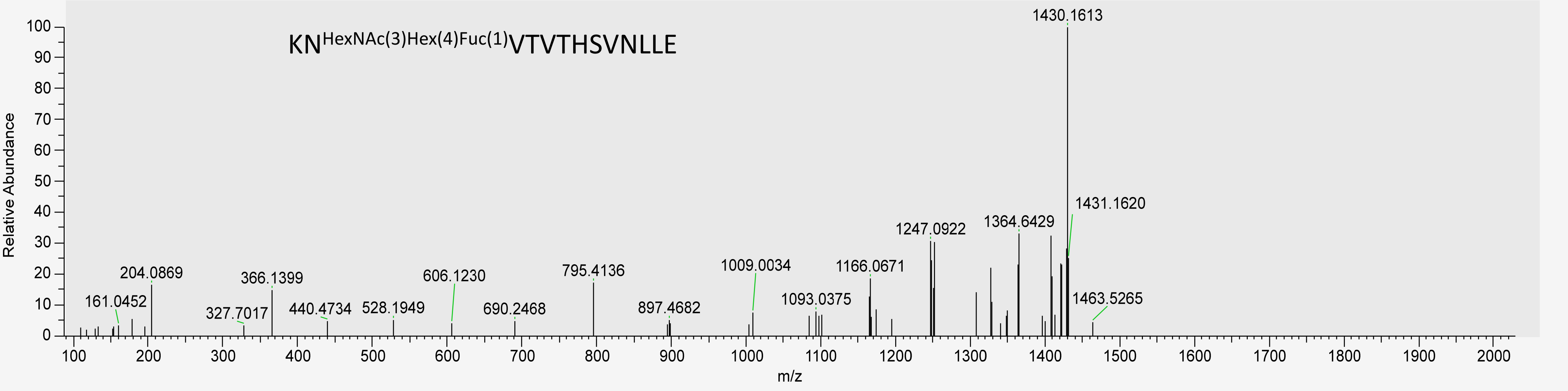

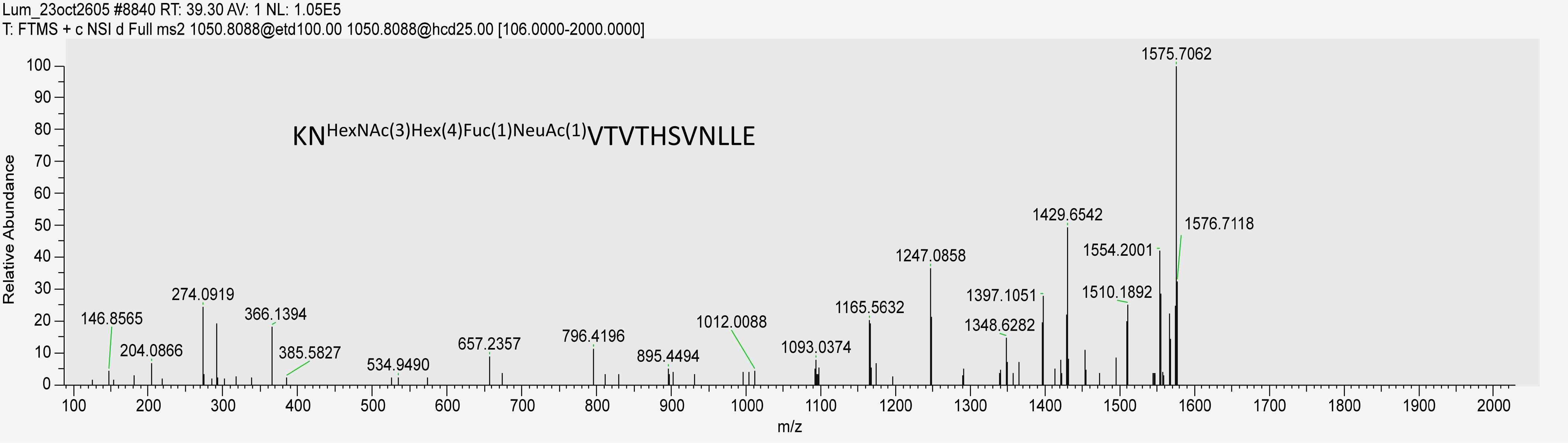

Lum_23oct2605 #8840 RT: 39.30 AV: 1 NL: 1.05E5
T: FTMS + c NSI d Full ms2 1050.8088@etd100.00 1050.8088@hcd25.00 [106.0000-2000.0000]
KN^HexNAc(3)Hex(4)Fuc(1)NeuAc(1)VTVTHSVNLLE
Relative Abundance
m/z
1575.7062
1576.7118
1429.6542
1554.2001
1247.0858
1397.1051
1510.1892
1165.5632
1348.6282
274.0919
366.1394
146.8565
1012.0088
204.0866
385.5827
657.2357
796.4196
895.4494
1093.0374
534.9490

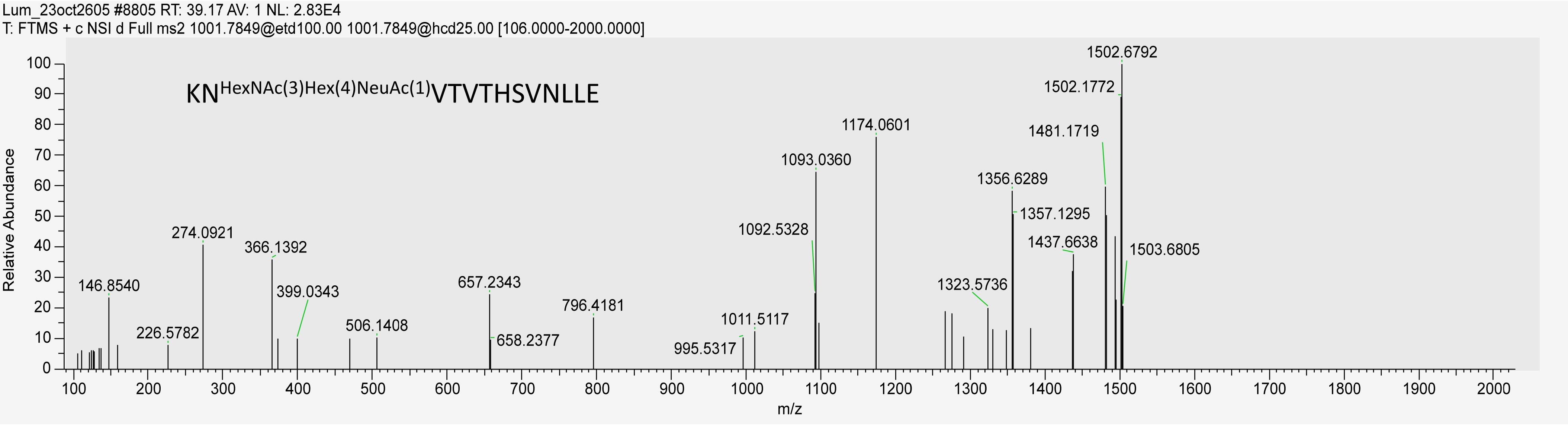

Lum_23oct2605 #8805 RT: 39.17 AV: 1 NL: 2.83E4
T: FTMS + c NSI d Full ms2 1001.7849@etd100.00 1001.7849@hcd25.00 [106.0000-2000.0000]
KN^HexNAc(3)Hex(4)NeuAc(1)VTVTHSVNLLE
Relative Abundance
m/z
1502.6792
1502.1772
1174.0601
1481.1719
1093.0360
1356.6289
1357.1295
1092.5328
1437.6638
1503.6805
274.0921
366.1392
1323.5736
146.8540
399.0343
657.2343
796.4181
1011.5117
506.1408
226.5782
658.2377
995.5317

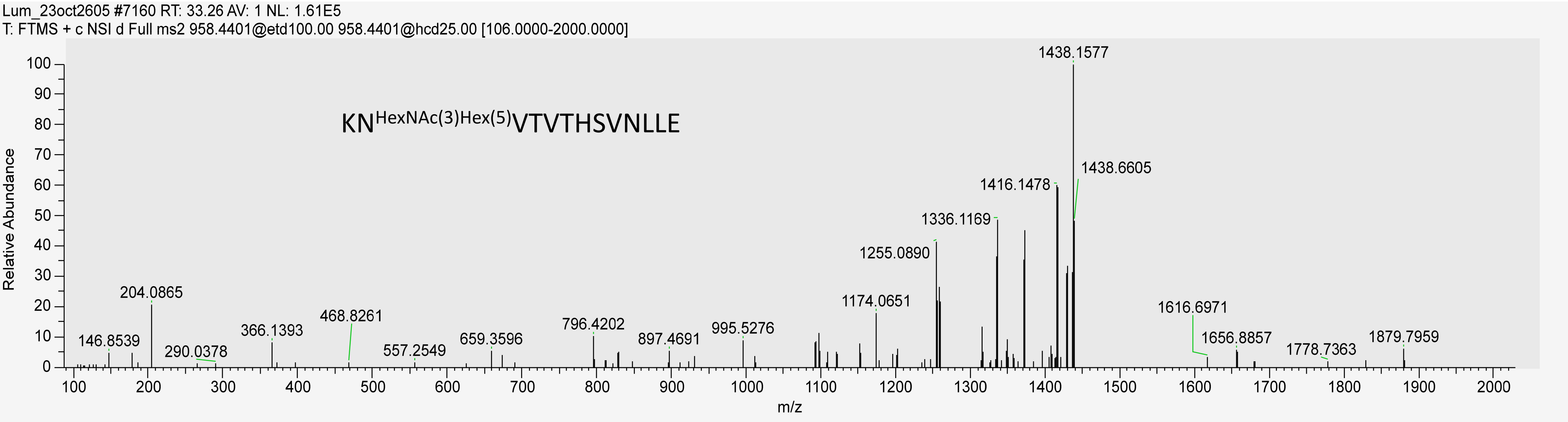
Lum_23oct2605 #7160 RT: 33.26 AV: 1 NL: 1.61E5
T: FTMS + c NSI d Full ms2 958.4401@etd100.00 958.4401@hcd25.00 [106.0000-2000.0000]
KN^HexNAc(3)Hex(5)VTVTHSVNLLE
Relative Abundance
m/z
1438.1577
1438.6605
1416.1478
1336.1169
1255.0890
1174.0651
1616.6971
1656.8857
1778.7363
1879.7959
995.5276
897.4691
796.4202
659.3596
557.2549
468.8261
366.1393
290.0378
204.0865
146.8539

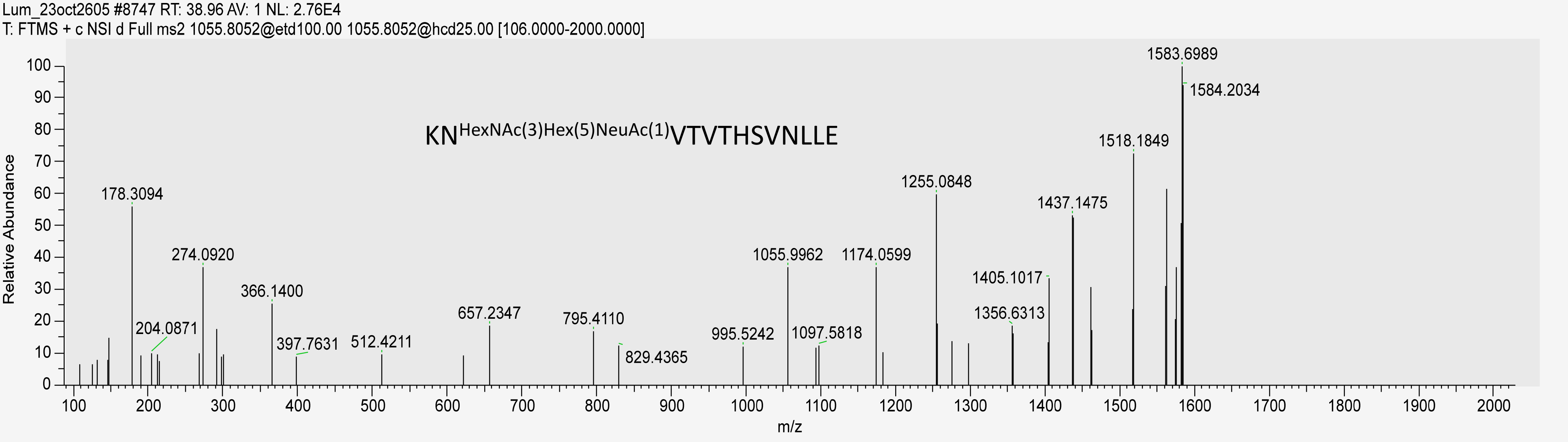
Lum_23oct2605 #8747 RT: 38.96 AV: 1 NL: 2.76E4
T: FTMS + c NSI d Full ms2 1055.8052@etd100.00 1055.8052@hcd25.00 [106.0000-2000.0000]
KN^HexNAc(3)Hex(5)NeuAc(1)VTVTHSVNLLE
Relative Abundance
m/z
1583.6989
1584.2034
1518.1849
1255.0848
1437.1475
1405.1017
1356.6313
1174.0599
1055.9962
1097.5818
995.5242
829.4365
795.4110
657.2347
512.4211
397.7631
366.1400
274.0920
204.0871
178.3094

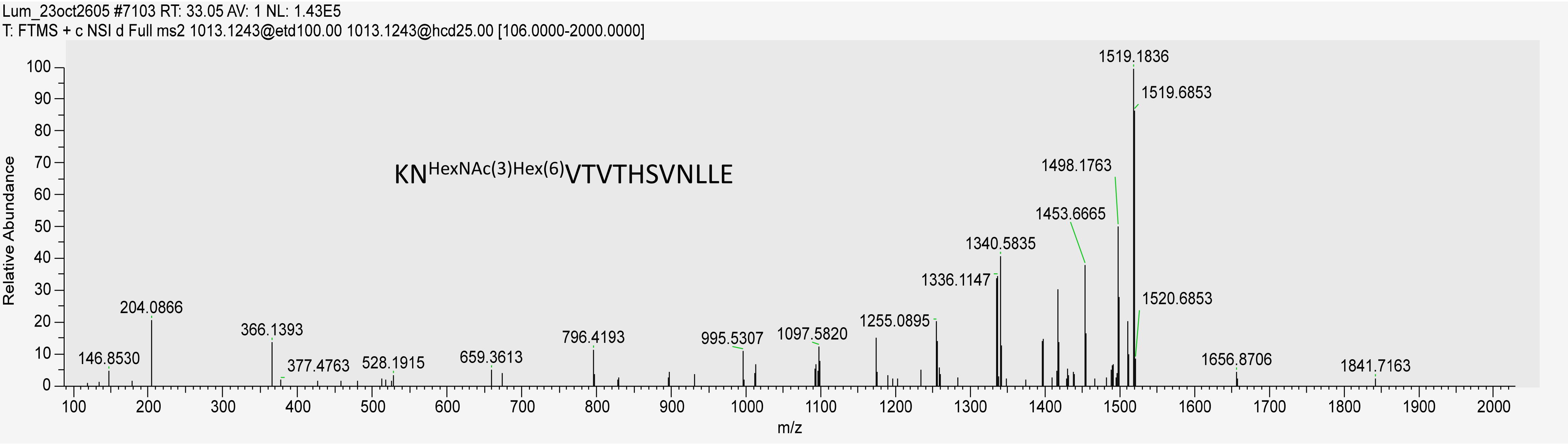
Lum_23oct2605 #7103 RT: 33.05 AV: 1 NL: 1.43E5
T: FTMS + c NSI d Full ms2 1013.1243@etd100.00 1013.1243@hcd25.00 [106.0000-2000.0000]
KN HexNAc(3)Hex(6) VTVTHSVNLLE
Relative Abundance
m/z
1519.1836
1519.6853
1498.1763
1453.6665
1340.5835
1336.1147
1520.6853
1255.0895
204.0866
366.1393
146.8530
377.4763
528.1915
659.3613
796.4193
995.5307
1097.5820
1656.8706
1841.7163

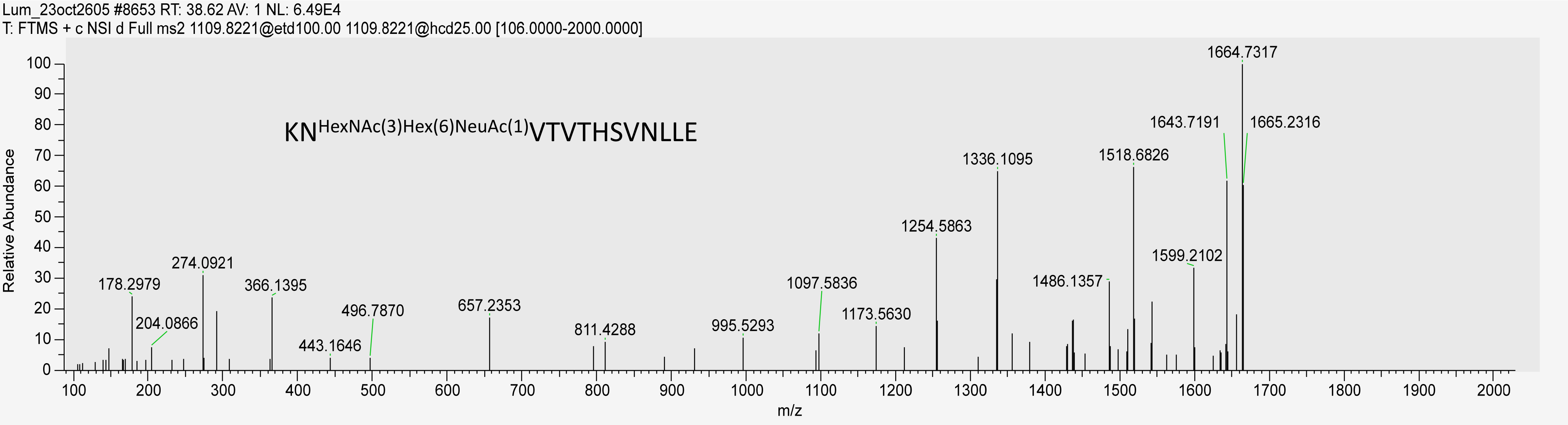
Lum_23oct2605 #8653 RT: 38.62 AV: 1 NL: 6.49E4
T: FTMS + c NSI d Full ms2 1109.8221@etd100.00 1109.8221@hcd25.00 [106.0000-2000.0000]
KN HexNAc(3)Hex(6)NeuAc(1) VTVTHSVNLLE
Relative Abundance
m/z
1664.7317
1643.7191
1665.2316
1518.6826
1336.1095
1254.5863
1599.2102
1486.1357
1097.5836
1173.5630
274.0921
178.2979
366.1395
204.0866
496.7870
443.1646
657.2353
811.4288
995.5293

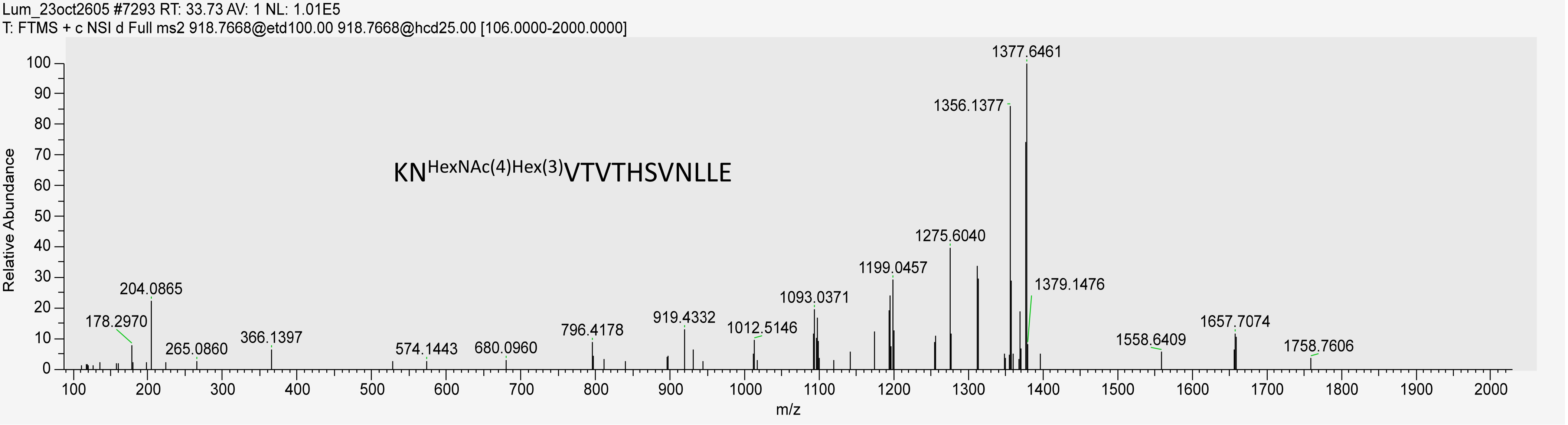
Lum_23oct2605 #7293 RT: 33.73 AV: 1 NL: 1.01E5
T: FTMS + c NSI d Full ms2 918.7668@etd100.00 918.7668@hcd25.00 [106.0000-2000.0000]
KN HexNAc(4)Hex(3) VTVTHSVNLLE
Relative Abundance
m/z
1377.6461
1356.1377
1275.6040
1199.0457
1379.1476
1093.0371
204.0865
178.2970
919.4332
796.4178
1012.5146
1657.7074
1558.6409
1758.7606
366.1397
265.0860
574.1443
680.0960

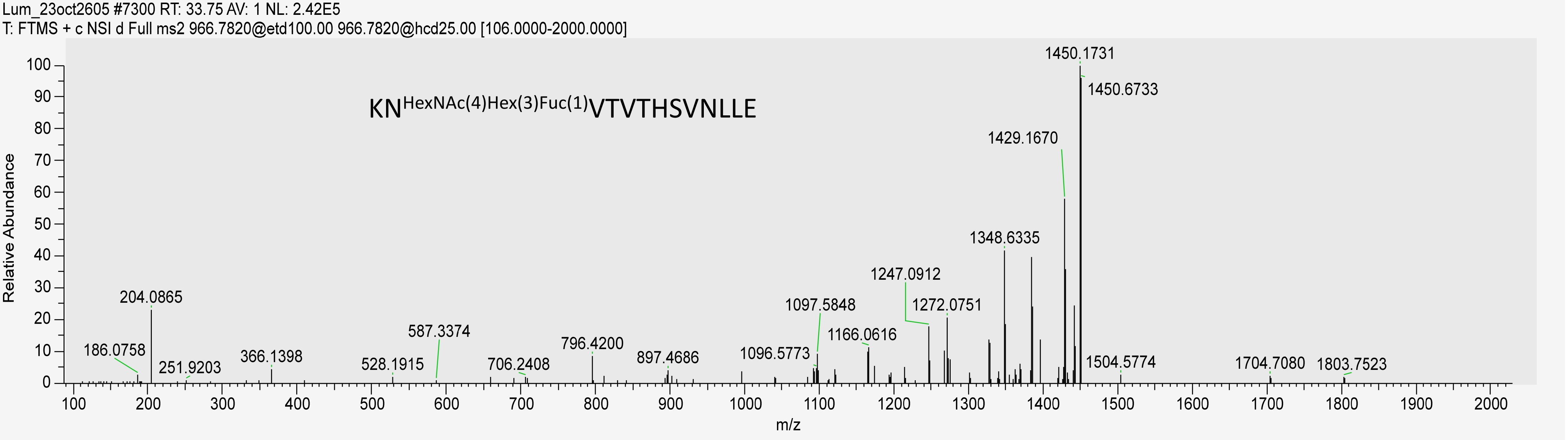
Lum_23oct2605 #7300 RT: 33.75 AV: 1 NL: 2.42E5
T: FTMS + c NSI d Full ms2 966.7820@etd100.00 966.7820@hcd25.00 [106.0000-2000.0000]
KN HexNAc(4)Hex(3)Fuc(1) VTVTHSVNLLE
Relative Abundance
m/z
1450.1731
1450.6733
1429.1670
1348.6335
1247.0912
1272.0751
1097.5848
1166.0616
204.0865
587.3374
186.0758
796.4200
366.1398
1096.5773
897.4686
251.9203
528.1915
706.2408
1504.5774
1704.7080
1803.7523

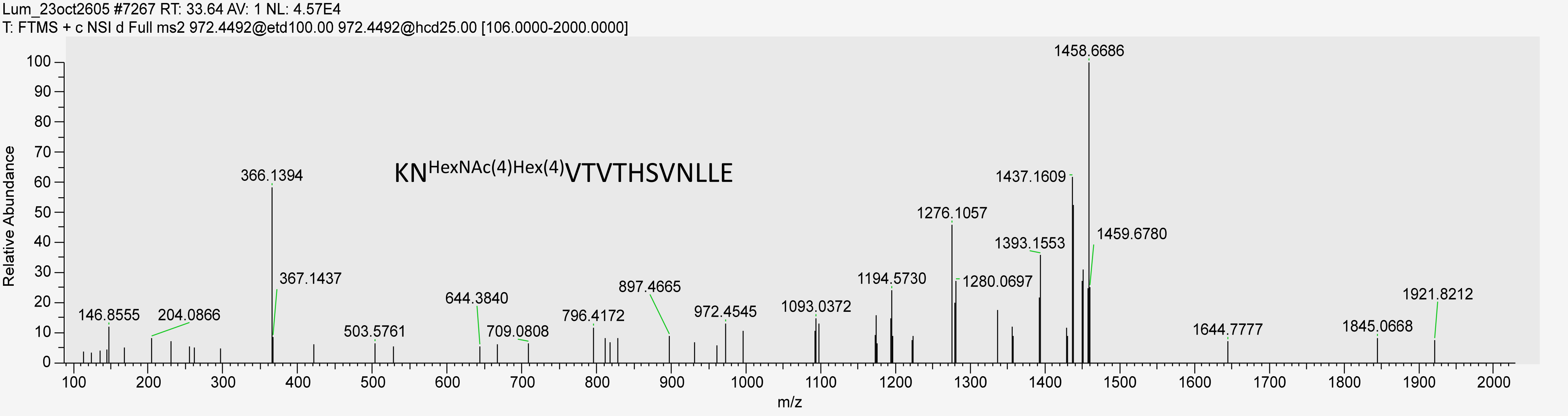
Lum_23oct2605 #7267 RT: 33.64 AV: 1 NL: 4.57E4
T: FTMS + c NSI d Full ms2 972.4492@etd100.00 972.4492@hcd25.00 [106.0000-2000.0000]
KN HexNAc(4)Hex(4) VTVTHSVNLLE
Relative Abundance
m/z
1458.6686
1437.1609
366.1394
1276.1057
1459.6780
1393.1553
367.1437
1194.5730
1280.0697
1921.8212
644.3840
897.4665
146.8555
204.0866
972.4545
1093.0372
796.4172
503.5761
709.0808
1644.7777
1845.0668

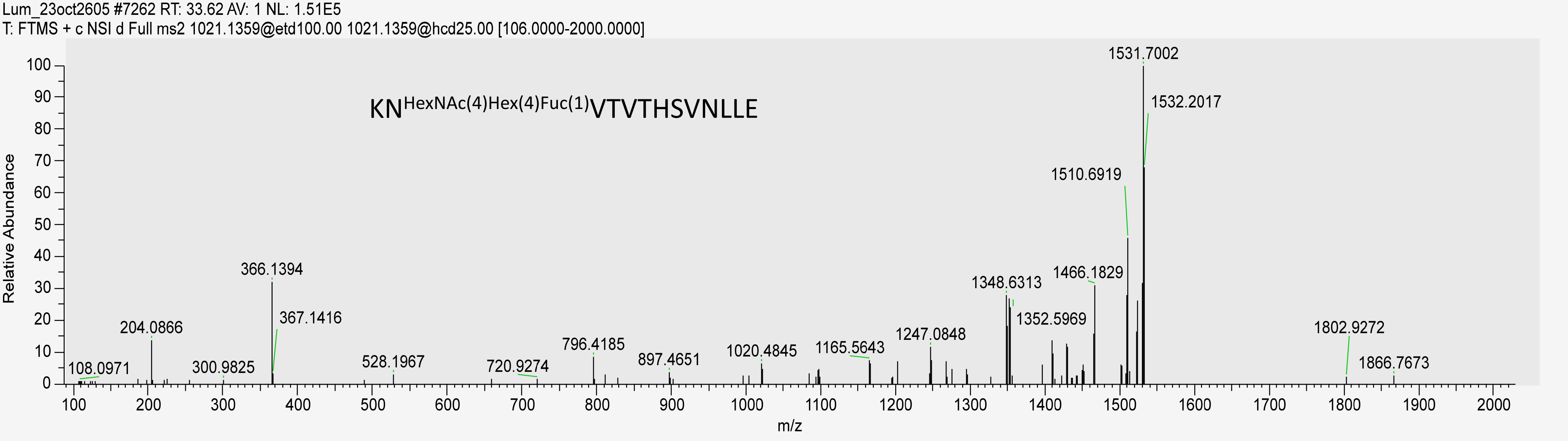
Lum_23oct2605 #7262 RT: 33.62 AV: 1 NL: 1.51E5
T: FTMS + c NSI d Full ms2 1021.1359@etd100.00 1021.1359@hcd25.00 [106.0000-2000.0000]
KN HexNAc(4)Hex(4)Fuc(1) VTVTHSVNLLE
Relative Abundance
m/z
1531.7002
1532.2017
1510.6919
366.1394
1466.1829
1348.6313
367.1416
1352.5969
204.0866
1247.0848
1802.9272
796.4185
1165.5643
1020.4845
897.4651
528.1967
1866.7673
108.0971
300.9825
720.9274

Lum_23oct2605 #8753 RT: 38.99 AV: 1 NL: 1.18E5
T: FTMS + c NSI d Full ms2 1118.1655@etd100.00 1118.1655@hcd25.00 [106.0000-2000.0000]

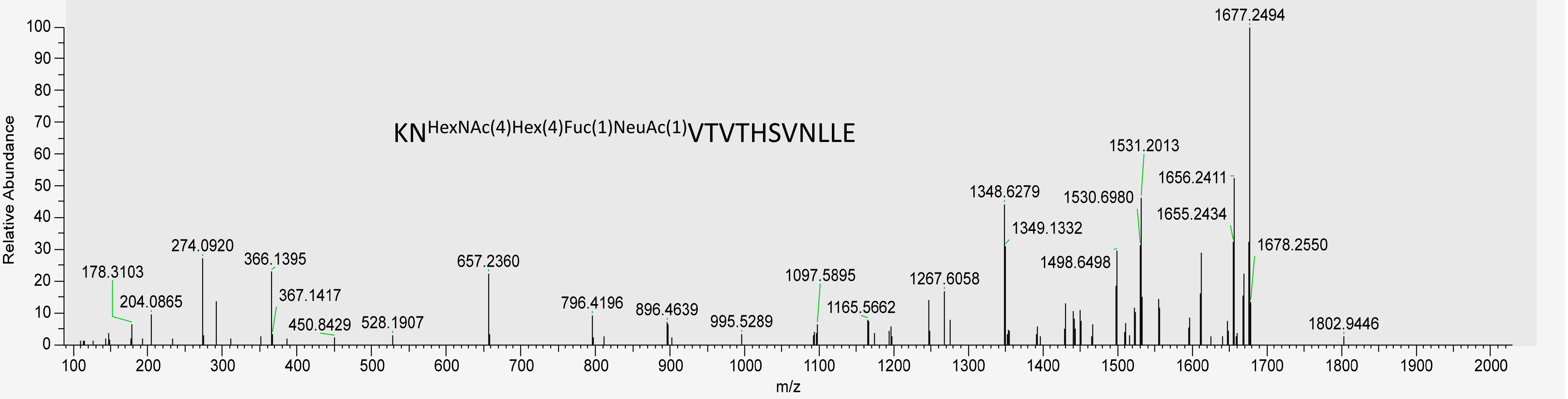

Lum_23oct2605 #7170 RT: 33.29 AV: 1 NL: 1.18E5
T: FTMS + c NSI d Full ms2 1026.7987@etd100.00 1026.7987@hcd25.00 [106.0000-2000.0000]

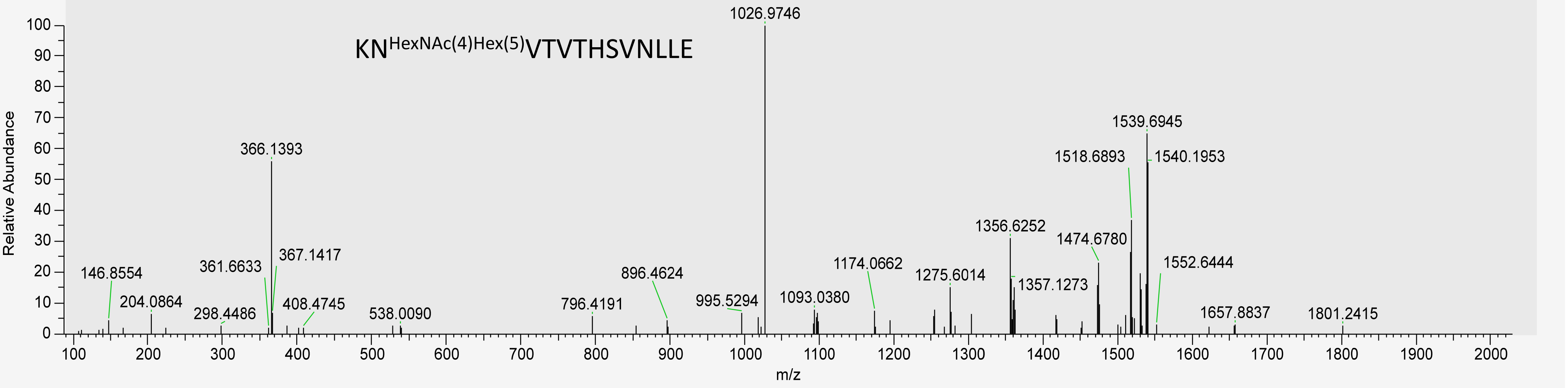

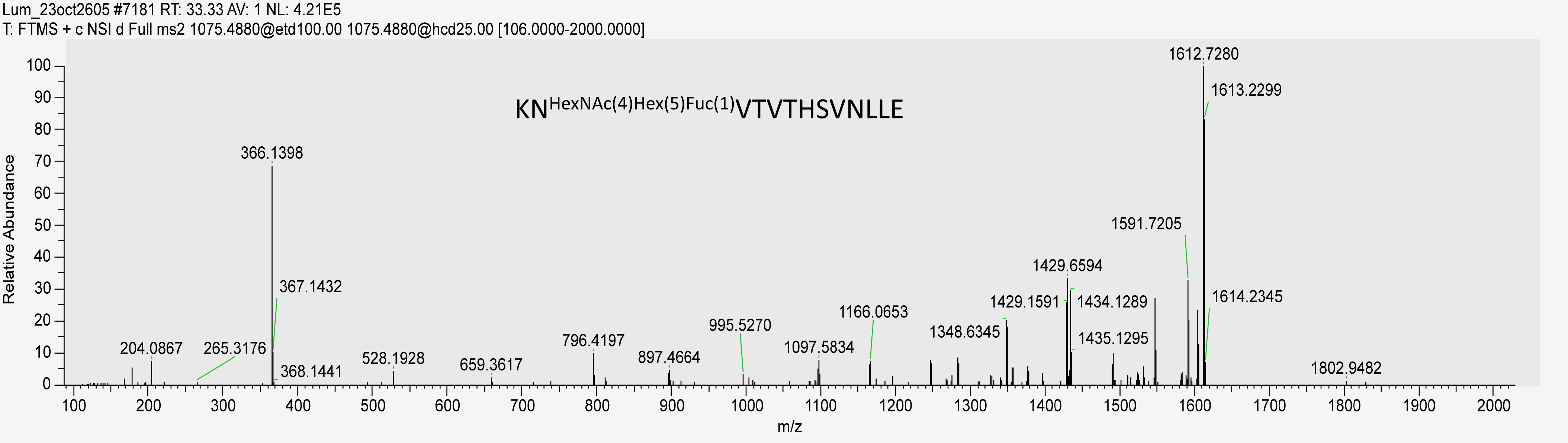

Lum_23oct2605 #7181 RT: 33.33 AV: 1 NL: 4.21E5
T: FTMS + c NSI d Full ms2 1075.4880@etd100.00 1075.4880@hcd25.00 [106.0000-2000.0000]
KN$^{HexNAc(4)Hex(5)Fuc(1)}$VTVTHSVNLLE
Relative Abundance
m/z
1612.7280
1613.2299
366.1398
367.1432
368.1441
204.0867
265.3176
528.1928
659.3617
796.4197
897.4664
995.5270
1097.5834
1166.0653
1348.6345
1429.1591
1429.6594
1434.1289
1435.1295
1591.7205
1614.2345
1802.9482

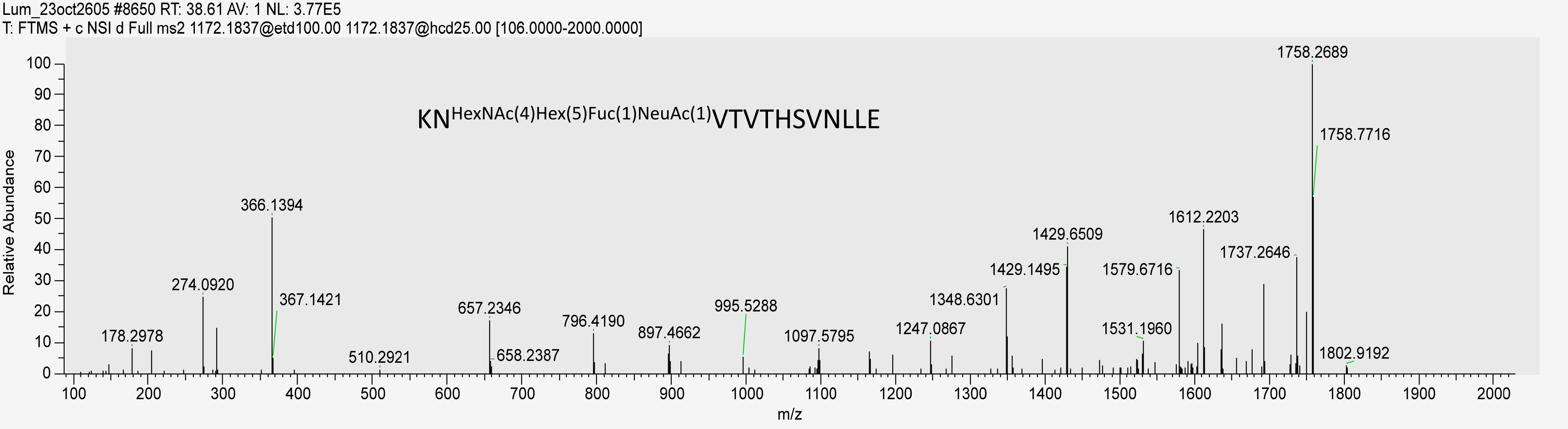

Lum_23oct2605 #8650 RT: 38.61 AV: 1 NL: 3.77E5
T: FTMS + c NSI d Full ms2 1172.1837@etd100.00 1172.1837@hcd25.00 [106.0000-2000.0000]
KN$^{HexNAc(4)Hex(5)Fuc(1)NeuAc(1)}$VTVTHSVNLLE
Relative Abundance
m/z
1758.2689
1758.7716
366.1394
367.1421
274.0920
178.2978
510.2921
657.2346
658.2387
796.4190
897.4662
995.5288
1097.5795
1247.0867
1348.6301
1429.1495
1429.6509
1531.1960
1579.6716
1612.2203
1737.2646
1802.9192

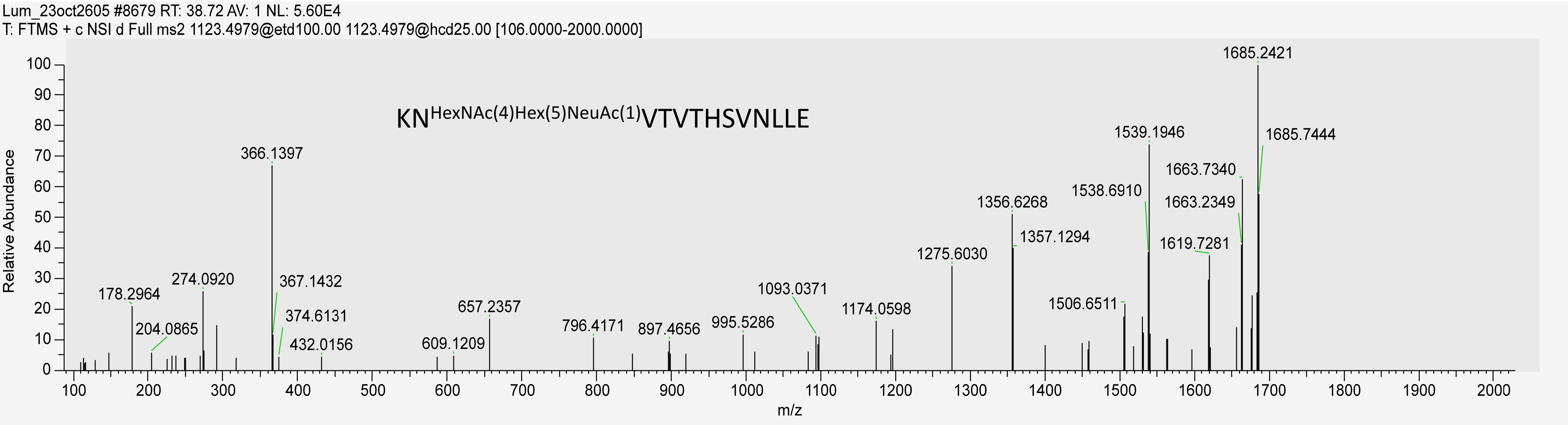
Lum_23oct2605 #8679 RT: 38.72 AV: 1 NL: 5.60E4
T: FTMS + c NSI d Full ms2 1123.4979@etd100.00 1123.4979@hcd25.00 [106.0000-2000.0000]
KN HexNAc(4)Hex(5)NeuAc(1) VTVTHSVNLLE
Relative Abundance
m/z
1685.2421
1685.7444
1539.1946
1663.7340
1538.6910
1663.2349
1356.6268
1357.1294
1619.7281
1275.6030
1506.6511
1093.0371
1174.0598
995.5286
897.4656
796.4171
657.2357
609.1209
366.1397
367.1432
374.6131
432.0156
274.0920
178.2964
204.0865

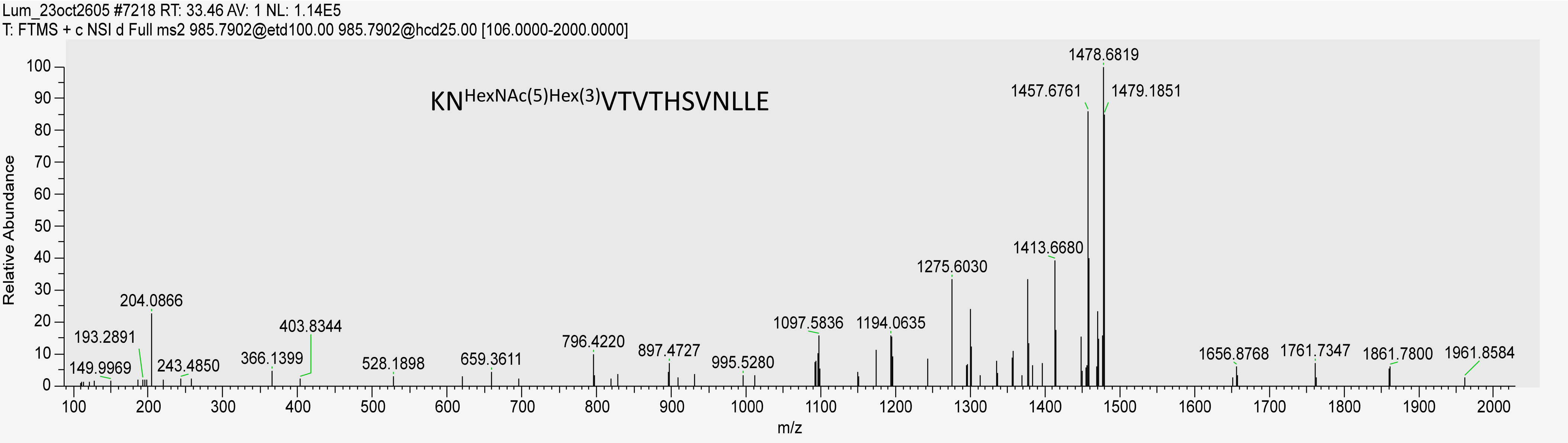
Lum_23oct2605 #7218 RT: 33.46 AV: 1 NL: 1.14E5
T: FTMS + c NSI d Full ms2 985.7902@etd100.00 985.7902@hcd25.00 [106.0000-2000.0000]
KN HexNAc(5)Hex(3) VTVTHSVNLLE
Relative Abundance
m/z
1478.6819
1457.6761
1479.1851
1413.6680
1275.6030
1097.5836
1194.0635
796.4220
897.4727
995.5280
659.3611
528.1898
403.8344
366.1399
243.4850
204.0866
193.2891
149.9969
1656.8768
1761.7347
1861.7800
1961.8584

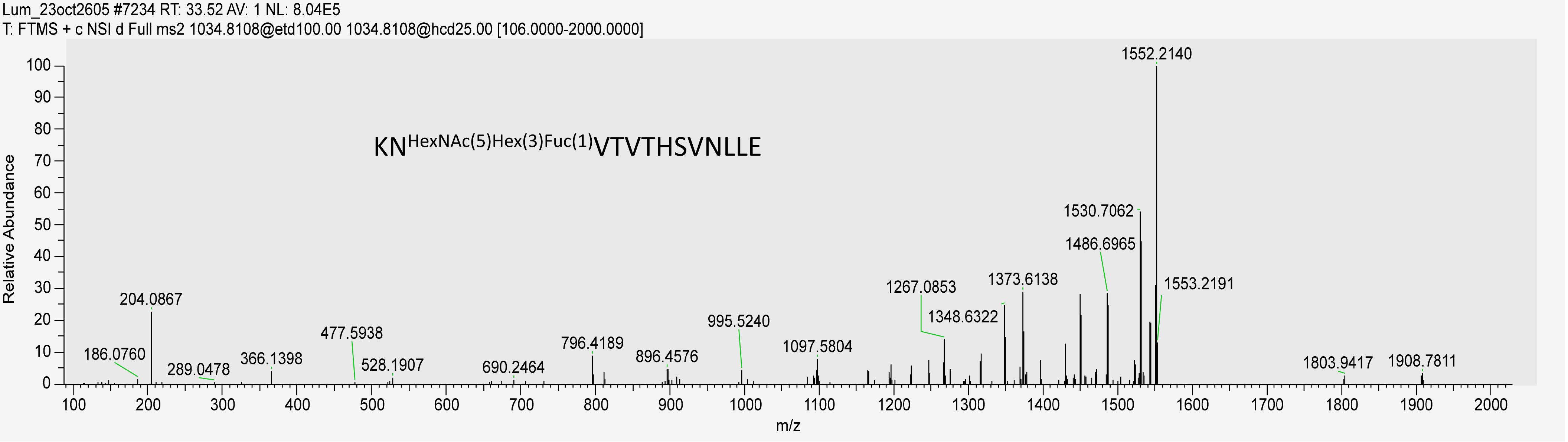
Lum_23oct2605 #7234 RT: 33.52 AV: 1 NL: 8.04E5
T: FTMS + c NSI d Full ms2 1034.8108@etd100.00 1034.8108@hcd25.00 [106.0000-2000.0000]
KN HexNAc(5)Hex(3)Fuc(1) VTVTHSVNLLE
Relative Abundance
m/z
1552.2140
1530.7062
1486.6965
1373.6138
1553.2191
1267.0853
1348.6322
995.5240
204.0867
477.5938
796.4189
1097.5804
896.4576
186.0760
366.1398
528.1907
690.2464
289.0478
1803.9417
1908.7811

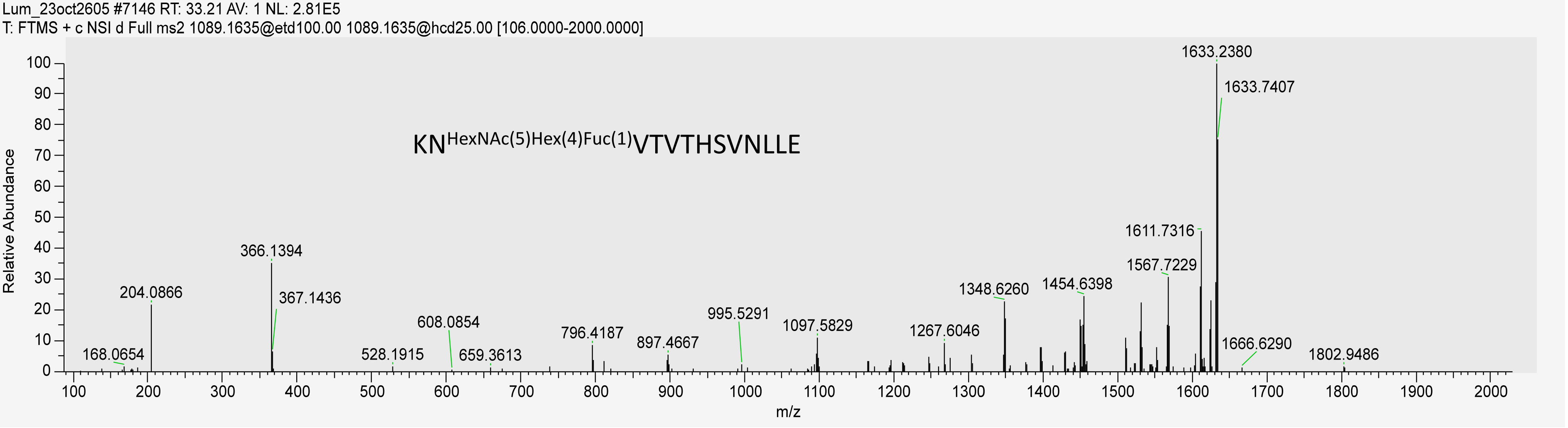
Lum_23oct2605 #7146 RT: 33.21 AV: 1 NL: 2.81E5
T: FTMS + c NSI d Full ms2 1089.1635@etd100.00 1089.1635@hcd25.00 [106.0000-2000.0000]
KN HexNAc(5)Hex(4)Fuc(1) VTVTHSVNLLE
Relative Abundance
m/z
1633.2380
1633.7407
1611.7316
1567.7229
366.1394
367.1436
204.0866
1348.6260
1454.6398
608.0854
995.5291
1097.5829
1267.6046
796.4187
897.4667
168.0654
528.1915
659.3613
1666.6290
1802.9486

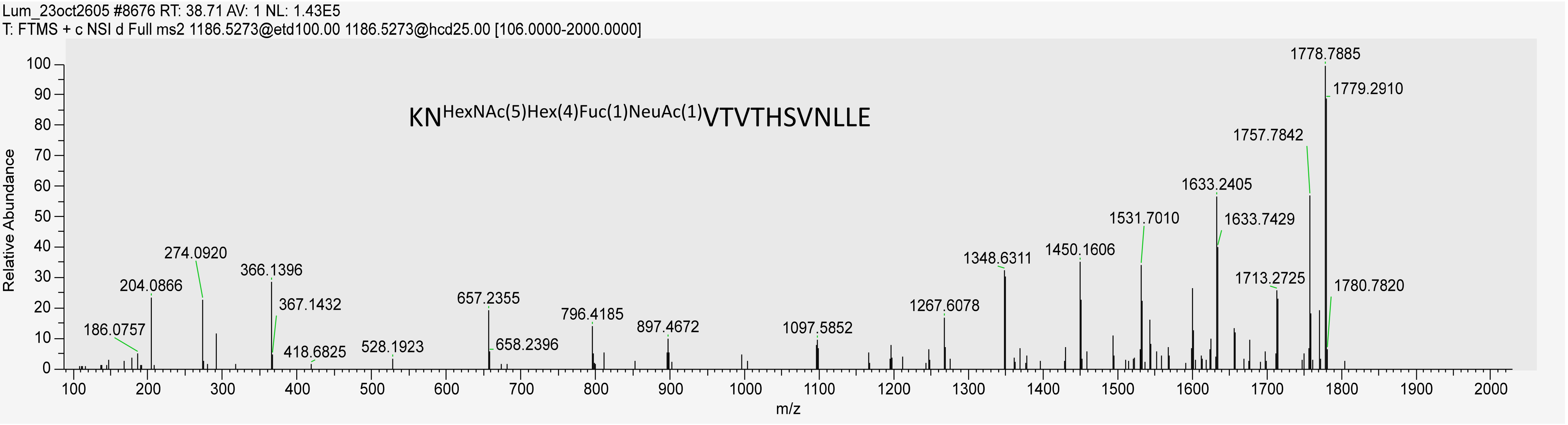
Lum_23oct2605 #8676 RT: 38.71 AV: 1 NL: 1.43E5
T: FTMS + c NSI d Full ms2 1186.5273@etd100.00 1186.5273@hcd25.00 [106.0000-2000.0000]
KN^HexNAc(5)Hex(4)Fuc(1)NeuAc(1)VTVTHSVNLLE
Relative Abundance
m/z
1778.7885
1779.2910
1757.7842
1633.2405
1531.7010
1633.7429
1450.1606
1348.6311
1713.2725
1780.7820
1267.6078
274.0920
366.1396
204.0866
367.1432
657.2355
796.4185
897.4672
1097.5852
186.0757
658.2396
418.6825
528.1923

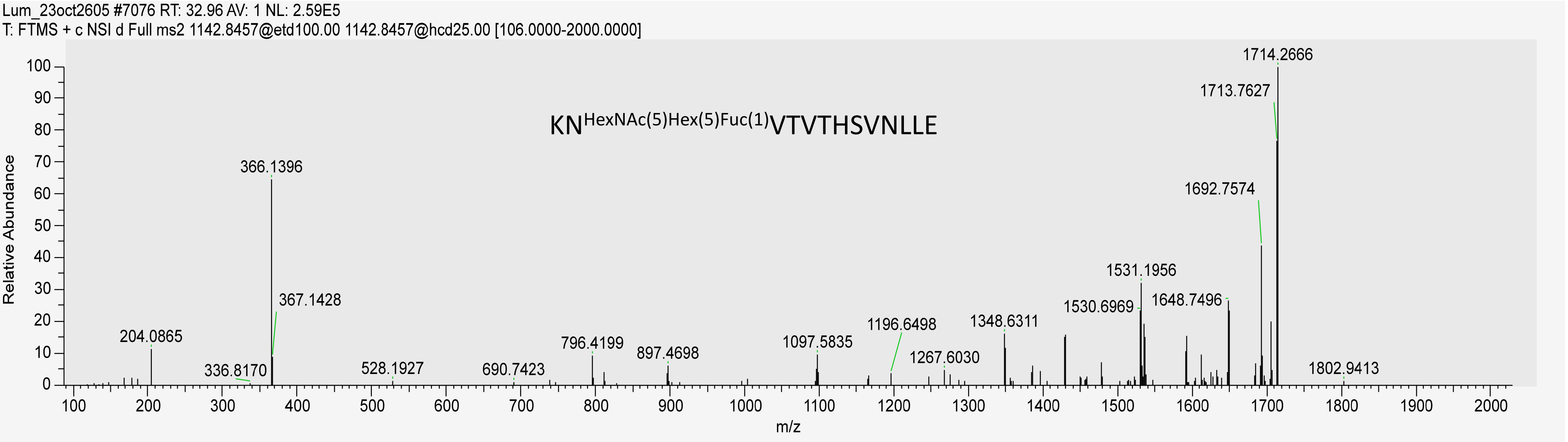
Lum_23oct2605 #7076 RT: 32.96 AV: 1 NL: 2.59E5
T: FTMS + c NSI d Full ms2 1142.8457@etd100.00 1142.8457@hcd25.00 [106.0000-2000.0000]
KN^HexNAc(5)Hex(5)Fuc(1)VTVTHSVNLLE
Relative Abundance
m/z
1714.2666
1713.7627
366.1396
1692.7574
1531.1956
367.1428
1530.6969
1648.7496
1196.6498
1348.6311
204.0865
796.4199
897.4698
1097.5835
1267.6030
336.8170
528.1927
690.7423
1802.9413

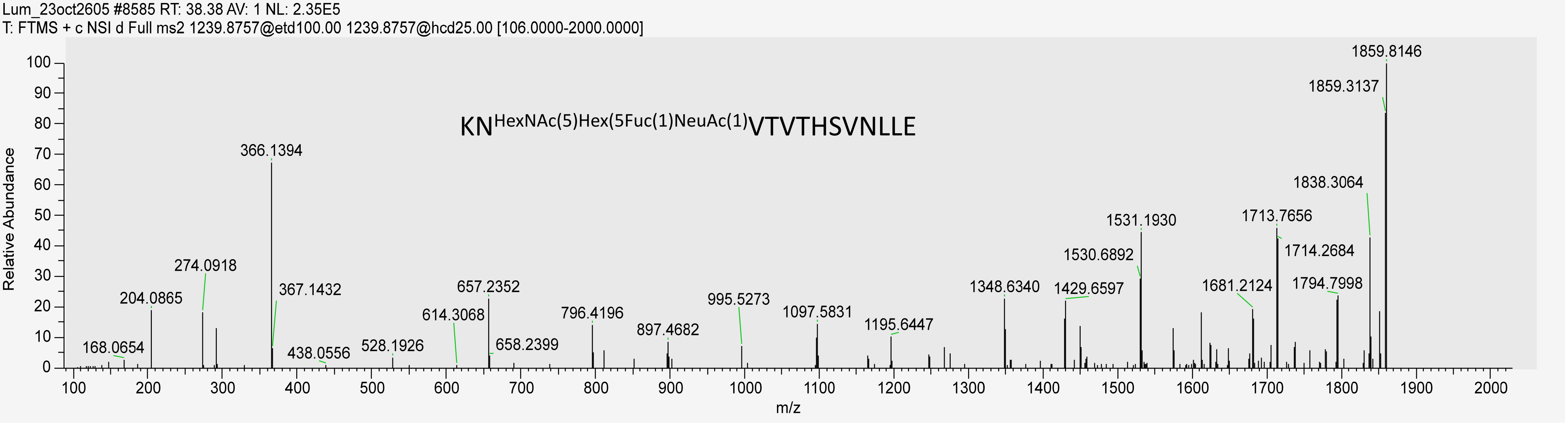
Lum_23oct2605 #8585 RT: 38.38 AV: 1 NL: 2.35E5
T: FTMS + c NSI d Full ms2 1239.8757@etd100.00 1239.8757@hcd25.00 [106.0000-2000.0000]
KNHexNAc(5)Hex(5Fuc(1)NeuAc(1)VTVTHSVNLLE
Relative Abundance
m/z
1859.8146
1859.3137
1838.3064
1713.7656
1714.2684
1531.1930
1530.6892
1794.7998
1681.2124
1348.6340
1429.6597
366.1394
274.0918
367.1432
204.0865
657.2352
614.3068
796.4196
995.5273
1097.5831
897.4682
1195.6447
658.2399
528.1926
168.0654
438.0556

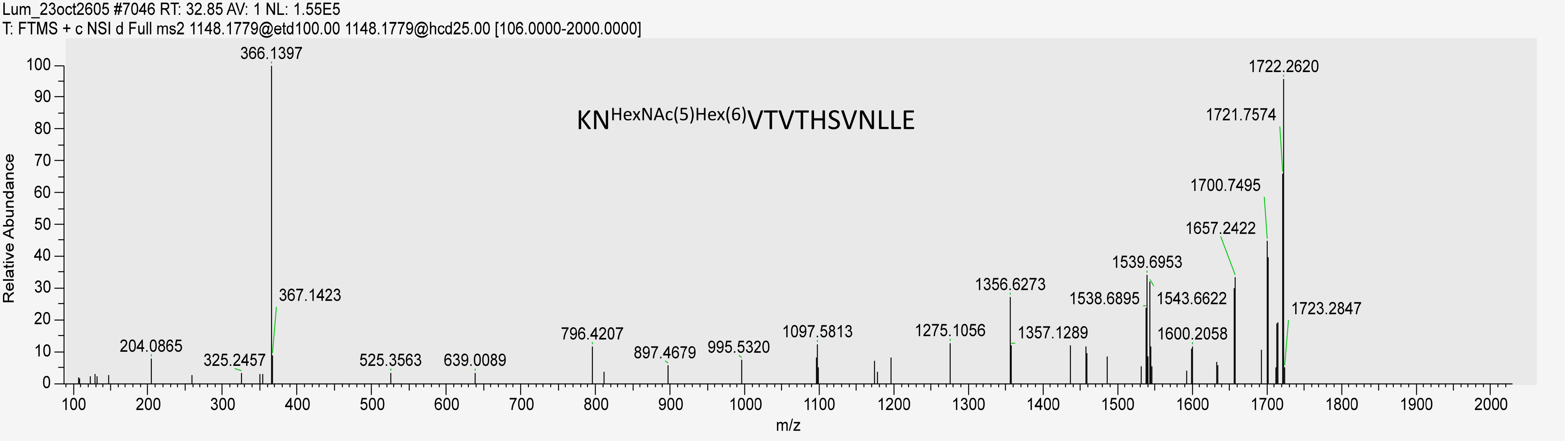
Lum_23oct2605 #7046 RT: 32.85 AV: 1 NL: 1.55E5
T: FTMS + c NSI d Full ms2 1148.1779@etd100.00 1148.1779@hcd25.00 [106.0000-2000.0000]
KNHexNAc(5)Hex(6)VTVTHSVNLLE
Relative Abundance
m/z
366.1397
1722.2620
1721.7574
1700.7495
1657.2422
1539.6953
1356.6273
1538.6895
1543.6622
1723.2847
367.1423
1357.1289
1275.1056
1600.2058
1097.5813
796.4207
204.0865
995.5320
897.4679
325.2457
525.3563
639.0089

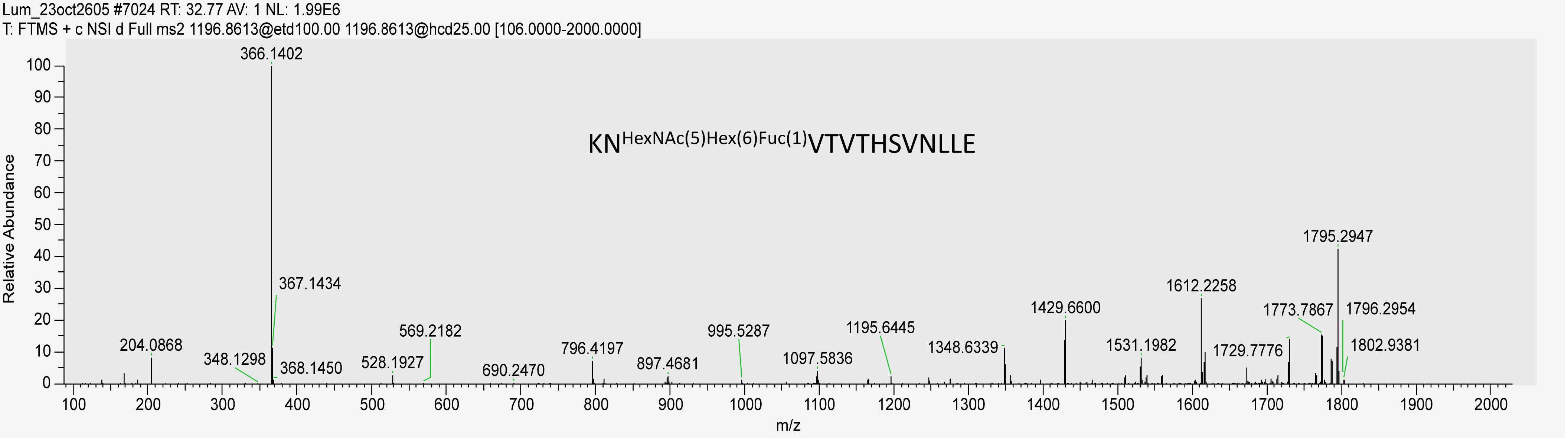

Lum_23oct2605 #7024 RT: 32.77 AV: 1 NL: 1.99E6
T: FTMS + c NSI d Full ms2 1196.8613@etd100.00 1196.8613@hcd25.00 [106.0000-2000.0000]
KN$^{HexNAc(5)Hex(6)Fuc(1)}$VTVTHSVNLLE
366.1402
367.1434
204.0868
348.1298
368.1450
528.1927
569.2182
690.2470
796.4197
897.4681
995.5287
1097.5836
1195.6445
1348.6339
1429.6600
1531.1982
1612.2258
1729.7776
1773.7867
1795.2947
1796.2954
1802.9381
Relative Abundance
m/z

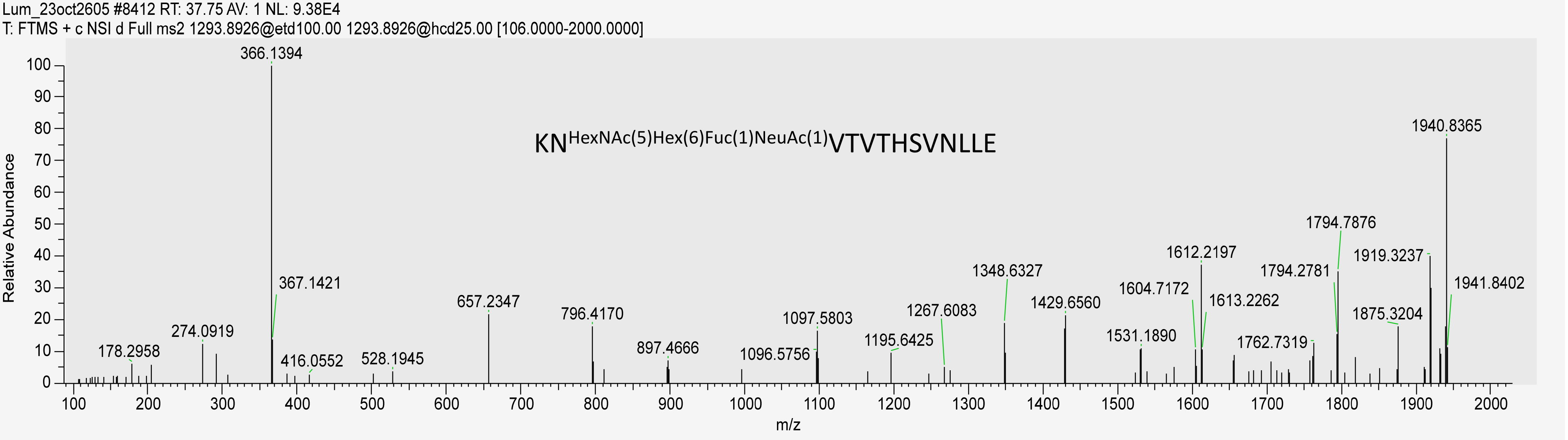

Lum_23oct2605 #8412 RT: 37.75 AV: 1 NL: 9.38E4
T: FTMS + c NSI d Full ms2 1293.8926@etd100.00 1293.8926@hcd25.00 [106.0000-2000.0000]
KN$^{HexNAc(5)Hex(6)Fuc(1)NeuAc(1)}$VTVTHSVNLLE
366.1394
367.1421
178.2958
274.0919
416.0552
528.1945
657.2347
796.4170
897.4666
1096.5756
1097.5803
1195.6425
1267.6083
1348.6327
1429.6560
1531.1890
1604.7172
1612.2197
1613.2262
1762.7319
1794.2781
1794.7876
1875.3204
1919.3237
1940.8365
1941.8402
Relative Abundance
m/z

Lum_23oct2605 #7198 RT: 33.39 AV: 1 NL: 2.89E5
T: FTMS + c NSI d Full ms2 1102.5040@etd100.00 1102.5040@hcd25.00 [106.0000-2000.0000]

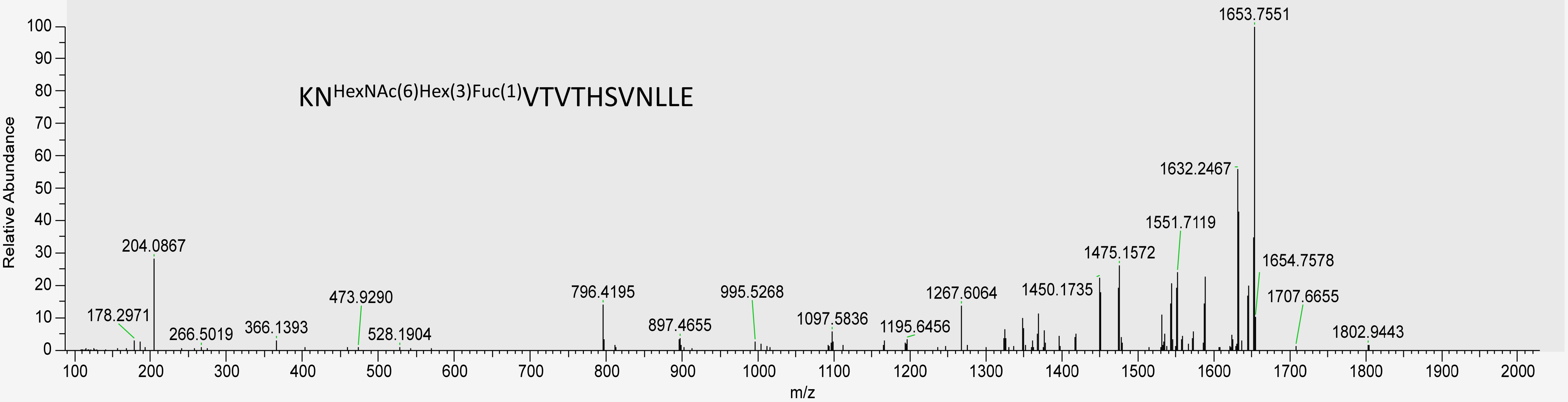

Lum_23oct2605 #7127 RT: 33.14 AV: 1 NL: 8.85E4
T: FTMS + c NSI d Full ms2 1156.8571@etd100.00 1156.8571@hcd25.00 [106.0000-2000.0000]

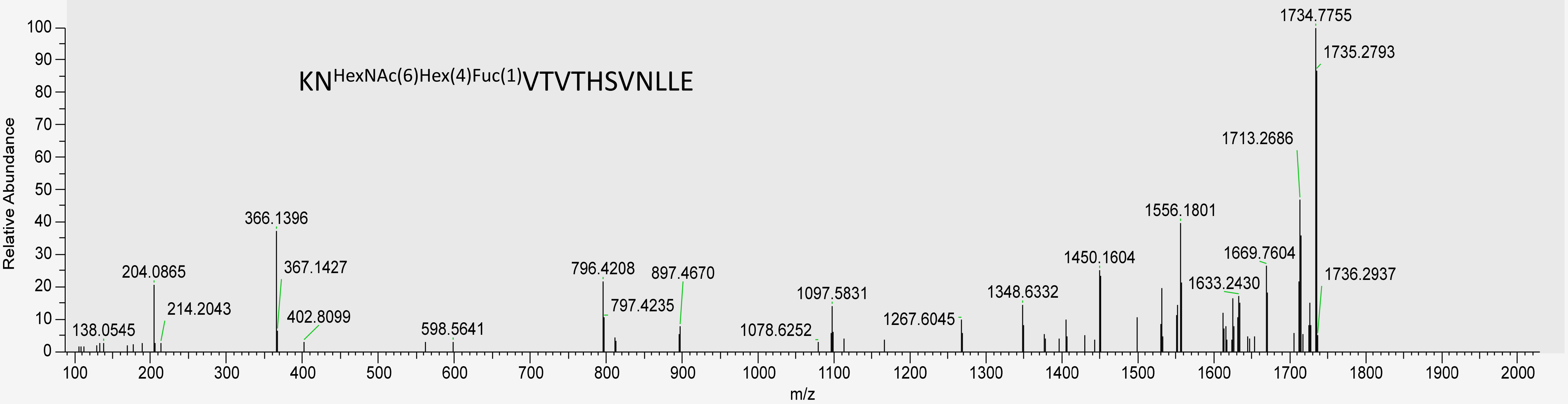

Lum_23oct2605 #8584 RT: 38.37 AV: 1 NL: 1.66E5
T: FTMS + c NSI d Full ms2 1307.5665@etd100.00 1307.5665@hcd25.00 [106.0000-2000.0000]

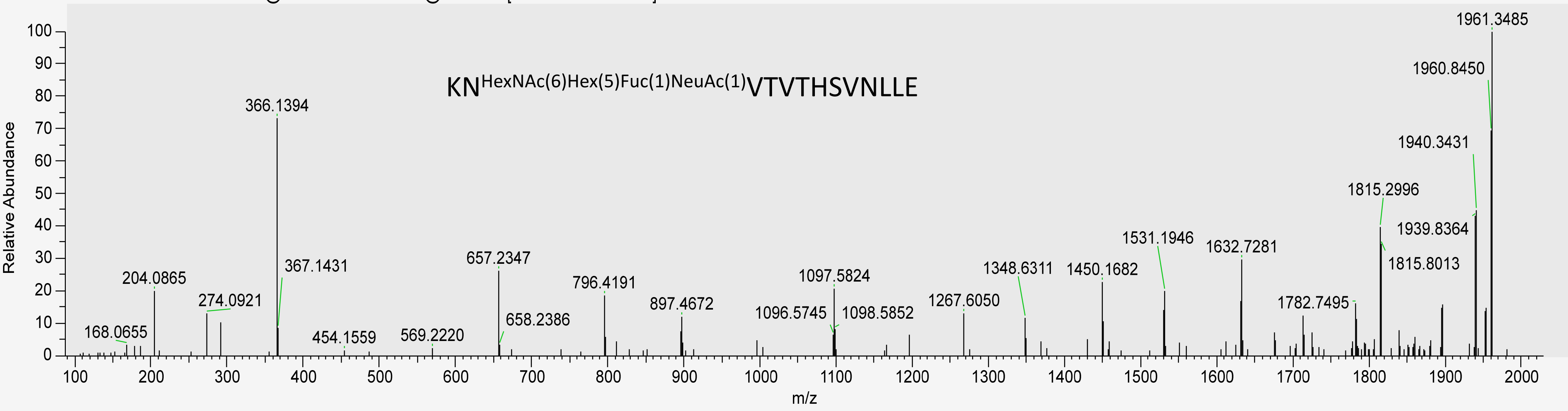

Lum_23oct2605 #6961 RT: 32.54 AV: 1 NL: 1.43E5
T: FTMS + c NSI d Full ms2 1318.9054@etd100.00 1318.9054@hcd25.00 [106.0000-2000.0000]

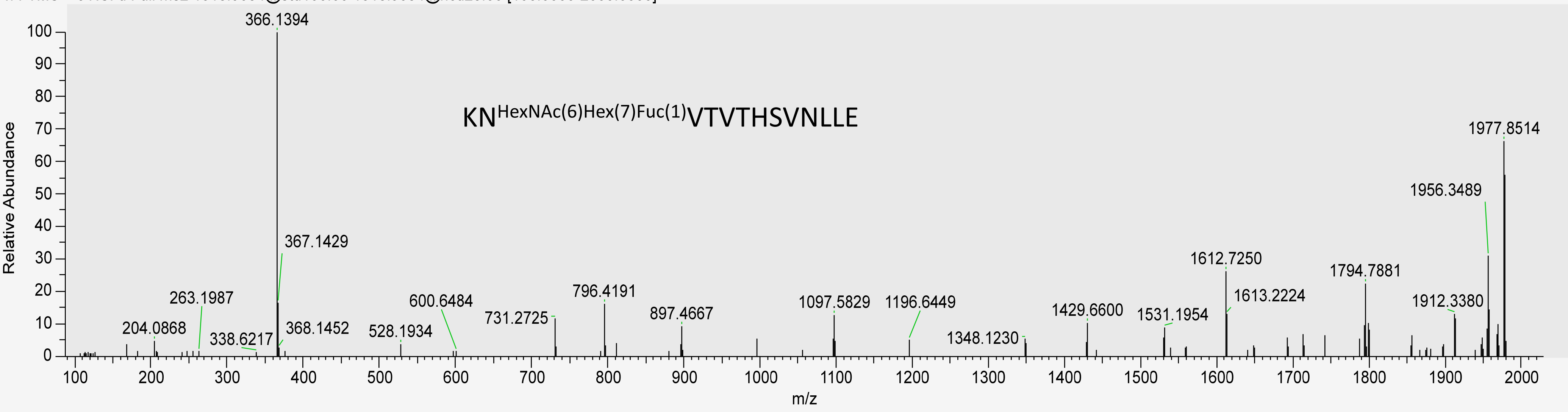

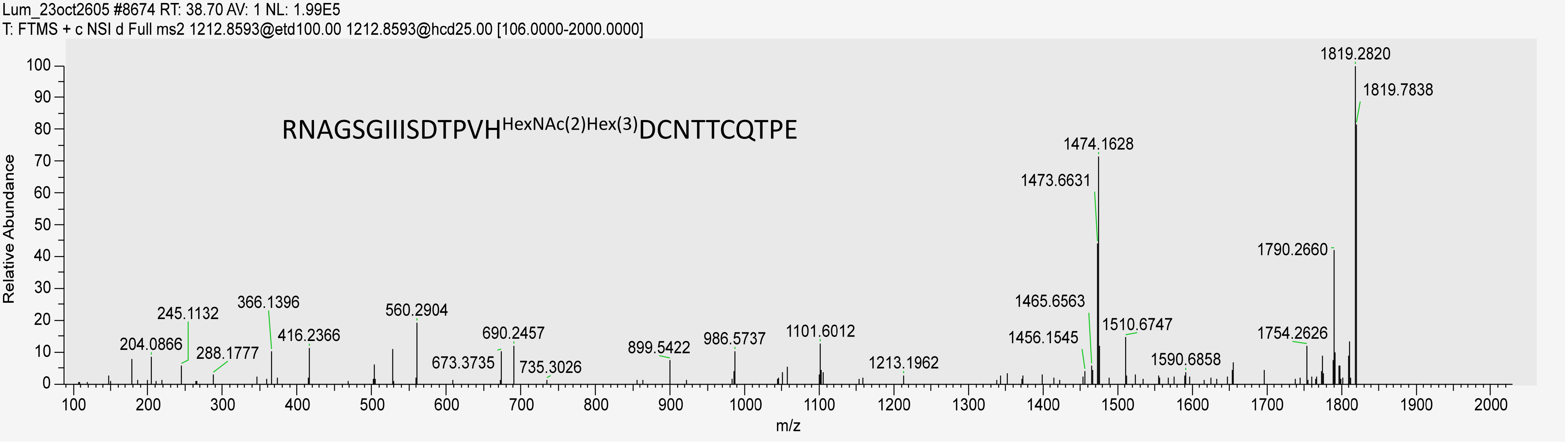
Lum_23oct2605 #8674 RT: 38.70 AV: 1 NL: 1.99E5
T: FTMS + c NSI d Full ms2 1212.8593@etd100.00 1212.8593@hcd25.00 [106.0000-2000.0000]
RNAGSGIIISDTPVH HexNAc(2)Hex(3) DCNTTCQTPE
Relative Abundance
m/z
1819.2820
1819.7838
1474.1628
1473.6631
1790.2660
1465.6563
1510.6747
1456.1545
1754.2626
1590.6858
1213.1962
1101.6012
986.5737
899.5422
735.3026
690.2457
673.3735
560.2904
416.2366
366.1396
288.1777
245.1132
204.0866

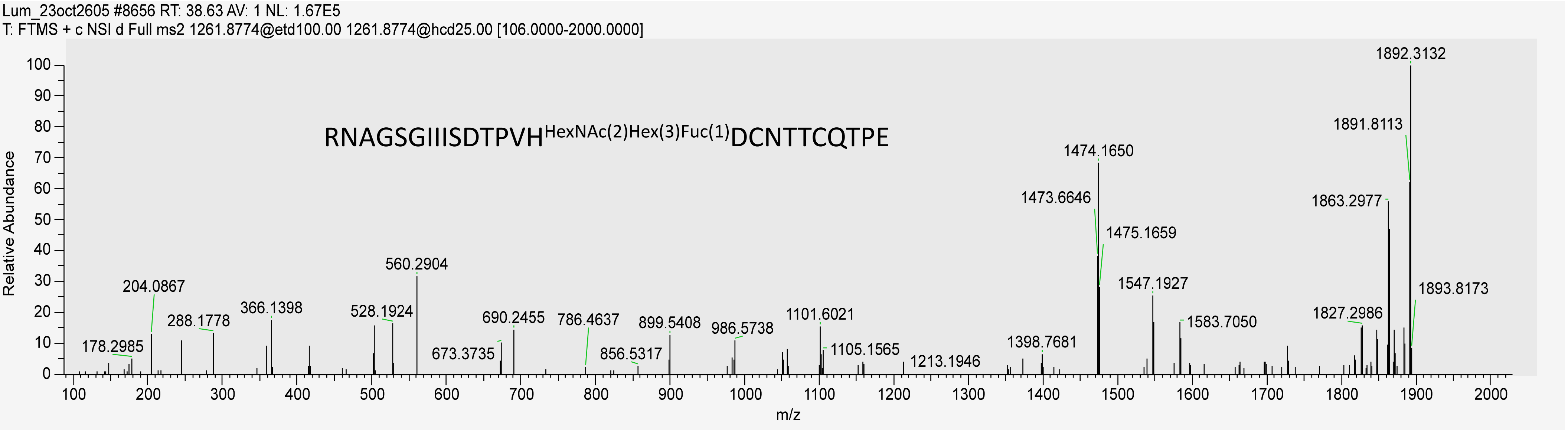
Lum_23oct2605 #8656 RT: 38.63 AV: 1 NL: 1.67E5
T: FTMS + c NSI d Full ms2 1261.8774@etd100.00 1261.8774@hcd25.00 [106.0000-2000.0000]
RNAGSGIIISDTPVH HexNAc(2)Hex(3)Fuc(1) DCNTTCQTPE
Relative Abundance
m/z
1892.3132
1891.8113
1863.2977
1893.8173
1827.2986
1474.1650
1473.6646
1475.1659
1547.1927
1583.7050
1398.7681
1213.1946
1105.1565
1101.6021
986.5738
899.5408
856.5317
786.4637
690.2455
673.3735
560.2904
528.1924
366.1398
288.1778
204.0867
178.2985

Lum_23oct2605 #8658 RT: 38.64 AV: 1 NL: 5.00E4
T: FTMS + c NSI d Full ms2 1266.8760@etd100.00 1266.8760@hcd25.00 [106.0000-2000.0000]

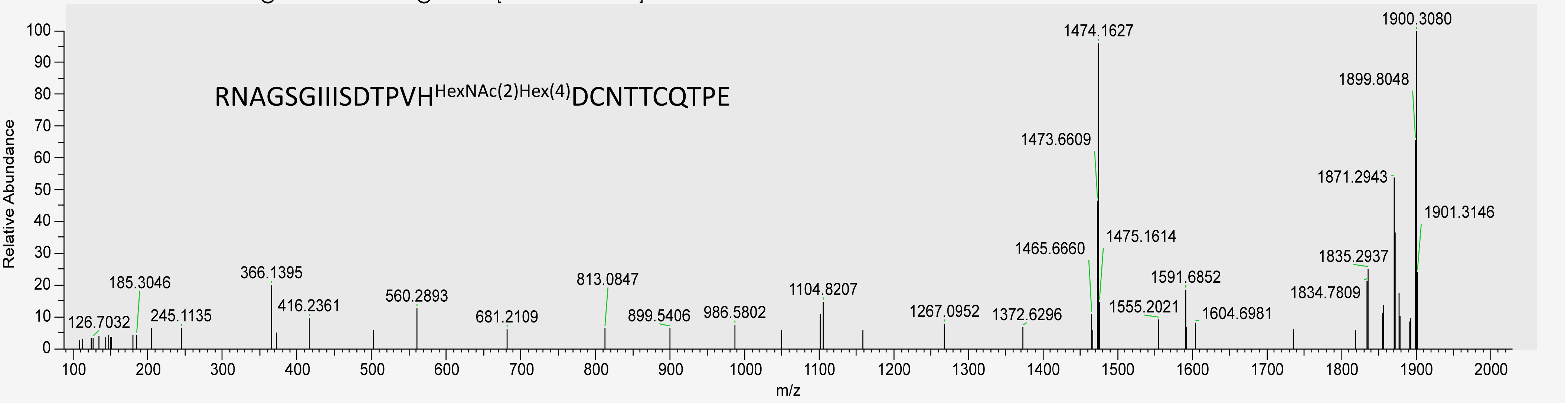

Lum_23oct2605 #8512 RT: 38.11 AV: 1 NL: 1.11E6
T: FTMS + c NSI d Full ms2 1321.2277@etd100.00 1321.2277@hcd25.00 [106.0000-2000.0000]

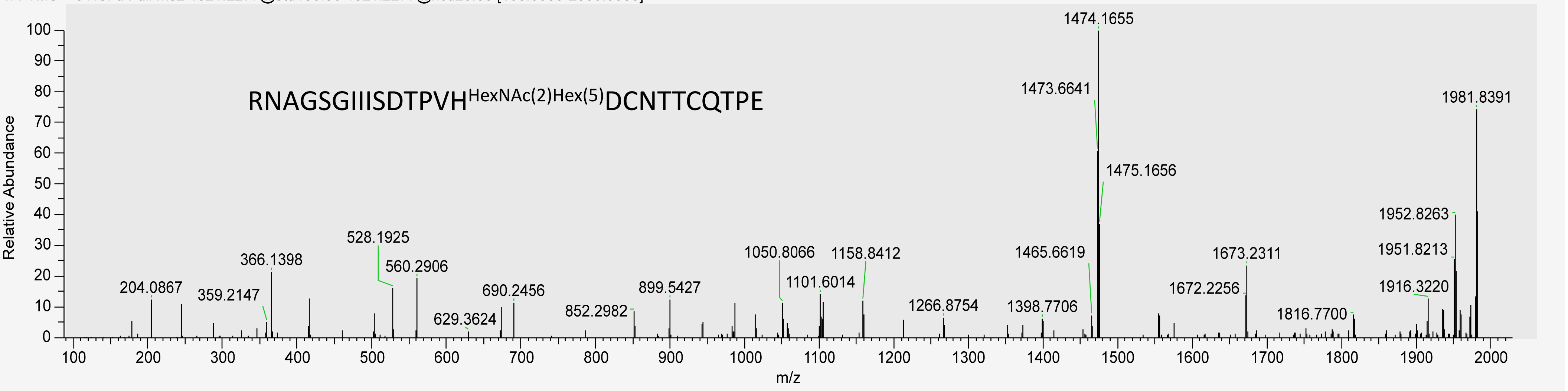

Lum_23oct2605 #8470 RT: 37.96 AV: 1 NL: 3.50E5
T: FTMS + c NSI d Full ms2 1375.2428@etd100.00 1375.2428@hcd25.00 [106.0000-2000.0000]

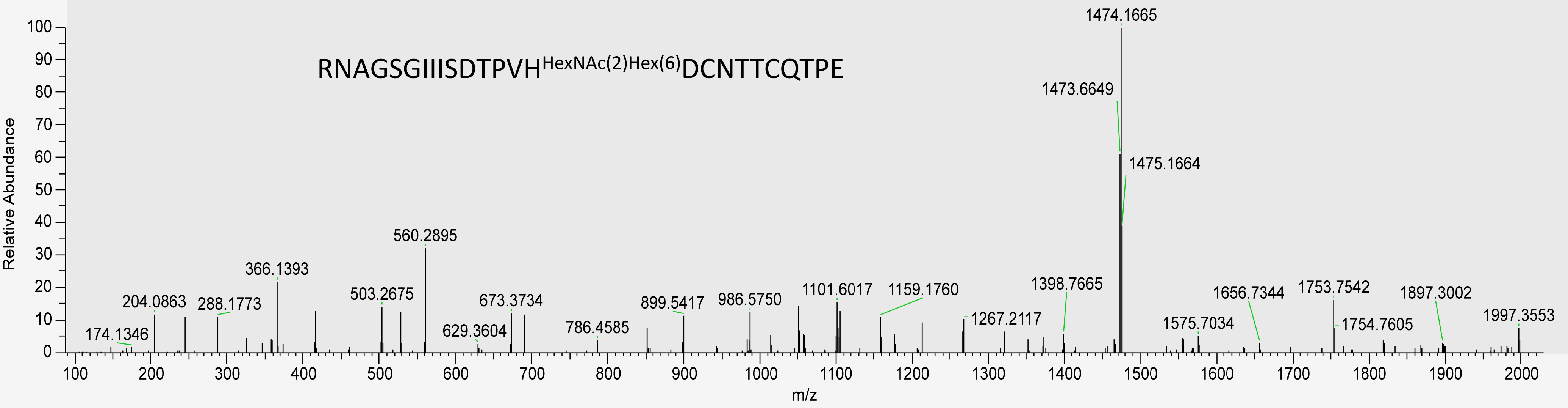

Lum_23oct2605 #8442 RT: 37.86 AV: 1 NL: 6.65E5
T: FTMS + c NSI d Full ms2 1429.2627@etd100.00 1429.2627@hcd25.00 [106.0000-2000.0000]

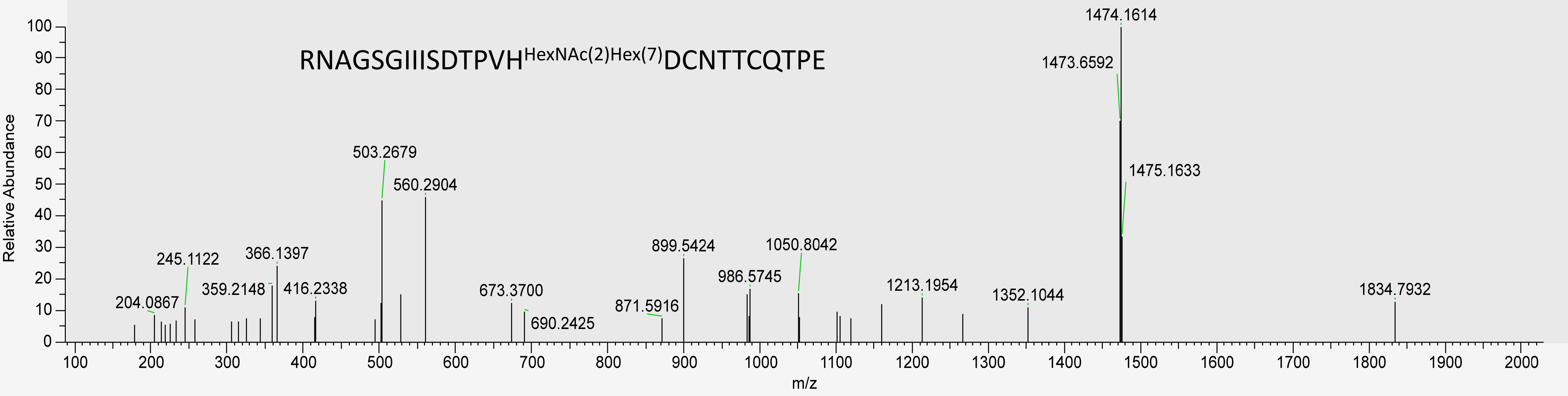

Lum_23oct2605 #8402 RT: 37.72 AV: 1 NL: 3.02E5
T: FTMS + c NSI d Full ms2 1483.2808@etd100.00 1483.2808@hcd25.00 [106.0000-2000.0000]

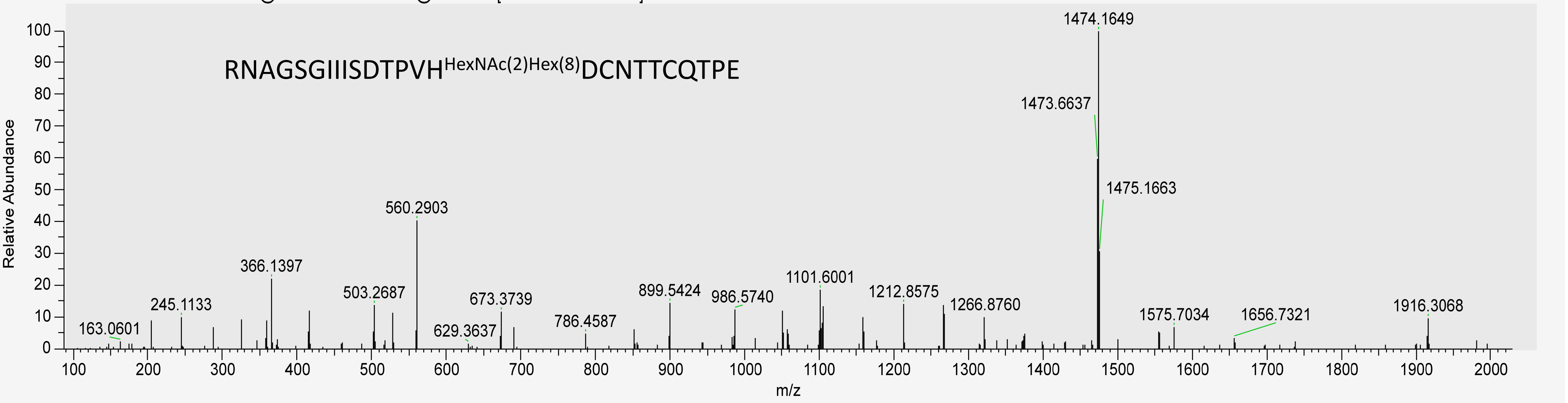

Lum_23oct2605 #8628 RT: 38.53 AV: 1 NL: 1.94E5
T: FTMS + c NSI d Full ms2 1280.5500@etd100.00 1280.5500@hcd25.00 [106.0000-2000.0000]

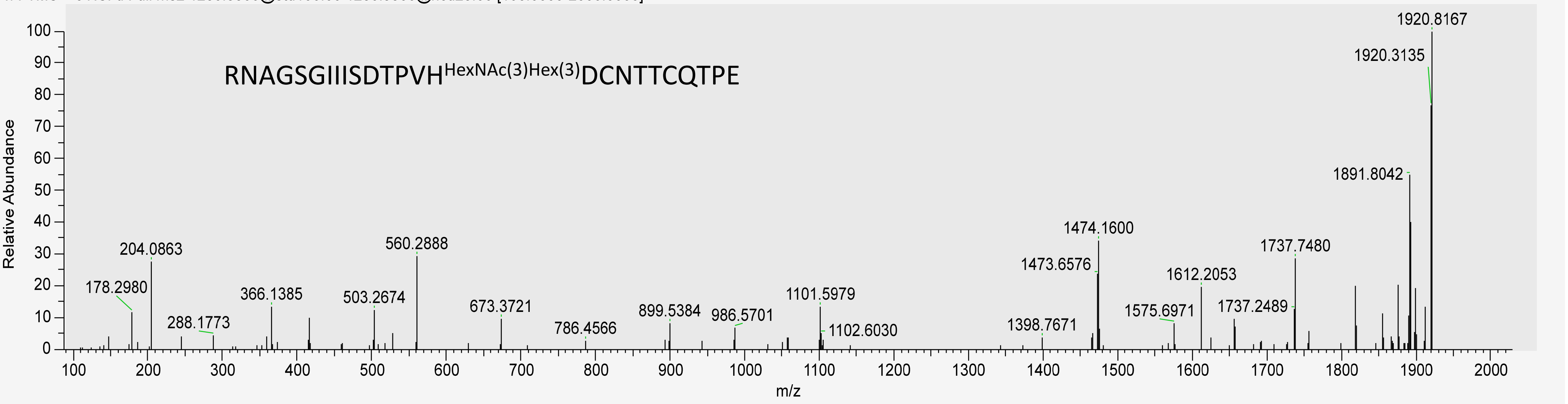

Lum_23oct2605 #8607 RT: 38.46 AV: 1 NL: 3.76E5
T: FTMS + c NSI d Full ms2 1329.5707@etd100.00 1329.5707@hcd25.00 [106.0000-2000.0000]

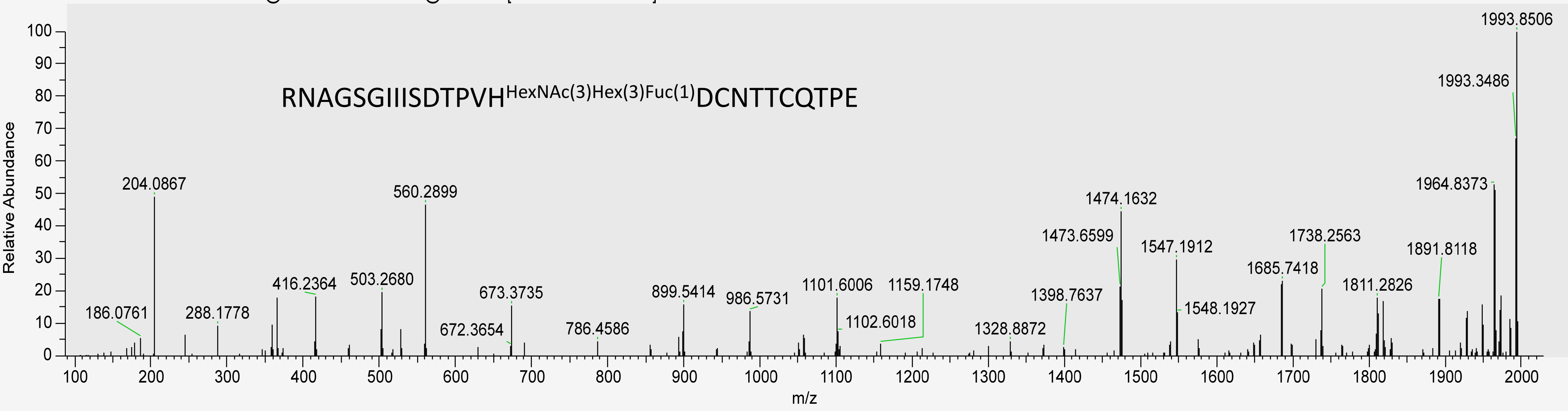

Lum_23oct2605 #8583 RT: 38.37 AV: 1 NL: 8.24E4
T: FTMS + c NSI d Full ms2 1334.8997@etd100.00 1334.8997@hcd25.00 [106.0000-2000.0000]

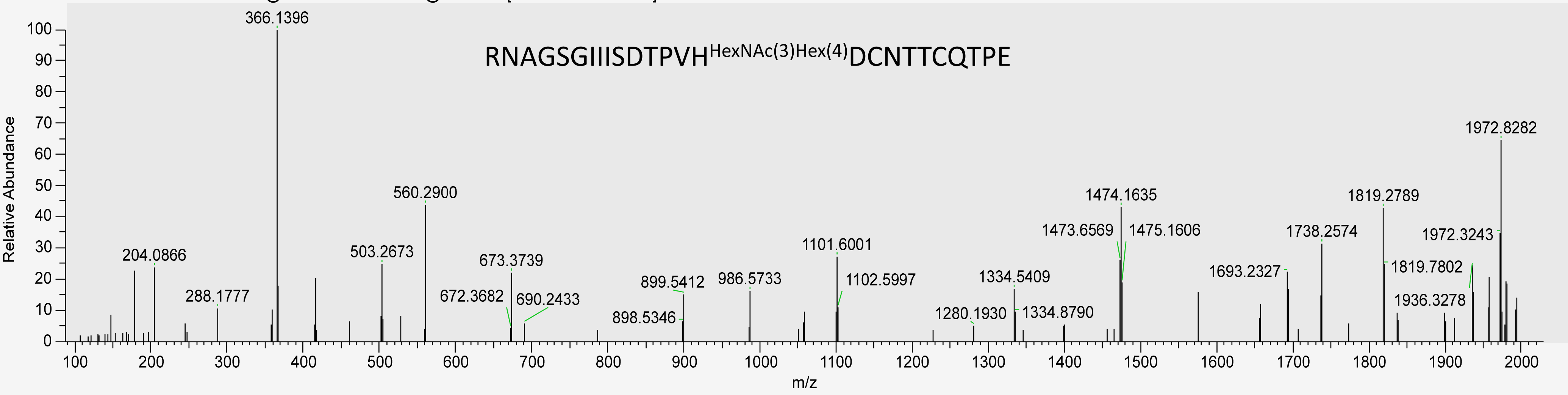

Lum_23oct2605 #8533 RT: 38.19 AV: 1 NL: 8.09E4
T: FTMS + c NSI d Full ms2 1383.5881@etd100.00 1383.5881@hcd25.00 [106.0000-2000.0000]

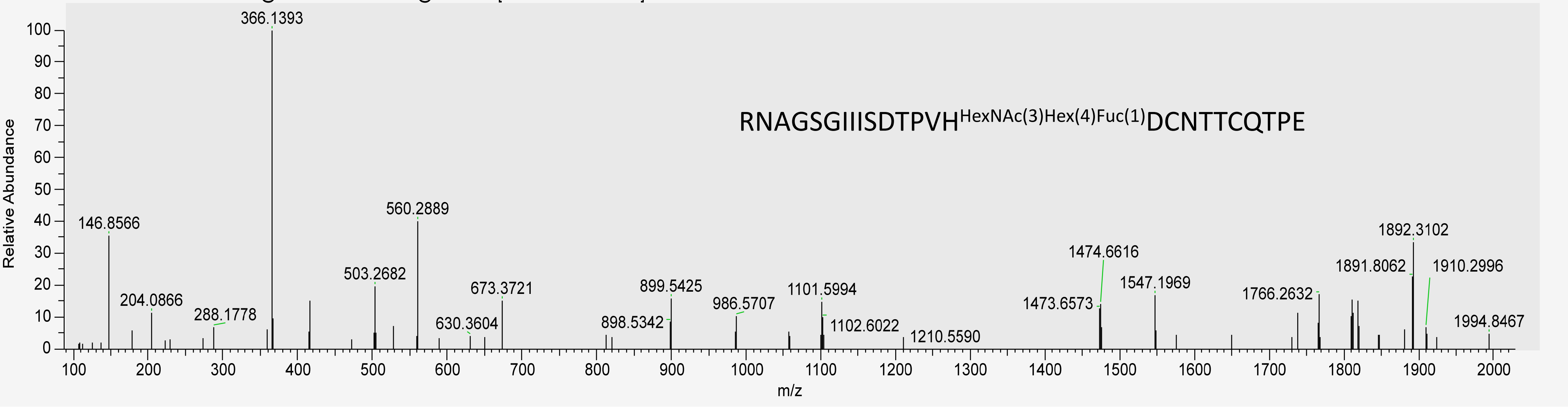

Lum_23oct2605 #9738 RT: 42.54 AV: 1 NL: 5.25E4
T: FTMS + c NSI d Full ms2 1480.6227@etd100.00 1480.6227@hcd25.00 [106.0000-2000.0000]

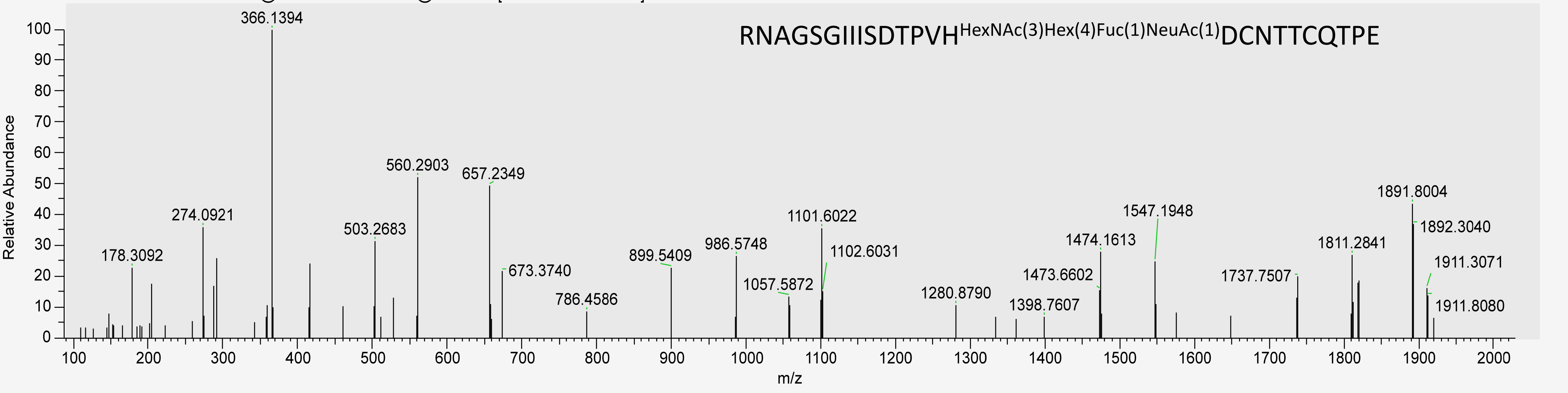

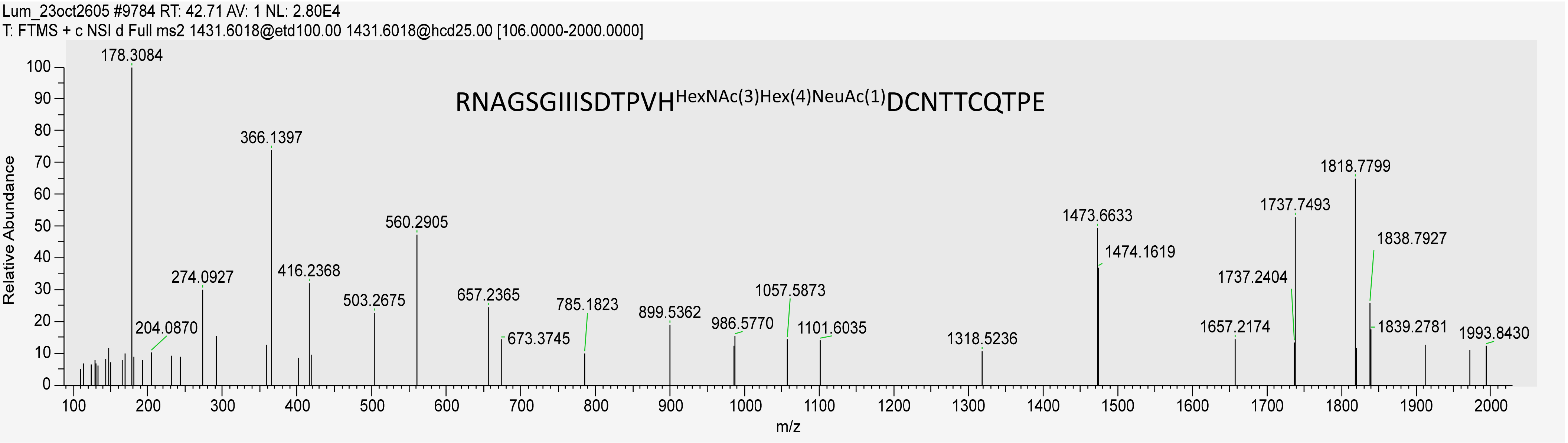

Lum_23oct2605 #9784 RT: 42.71 AV: 1 NL: 2.80E4
T: FTMS + c NSI d Full ms2 1431.6018@etd100.00 1431.6018@hcd25.00 [106.0000-2000.0000]
RNAGSGIIISDTPVH HexNAc(3)Hex(4)NeuAc(1) DCNTTCQTPE
Relative Abundance
m/z
178.3084
366.1397
560.2905
274.0927
416.2368
503.2675
204.0870
657.2365
673.3745
785.1823
899.5362
986.5770
1057.5873
1101.6035
1318.5236
1473.6633
1474.1619
1657.2174
1737.2404
1737.7493
1818.7799
1838.7927
1839.2781
1993.8430

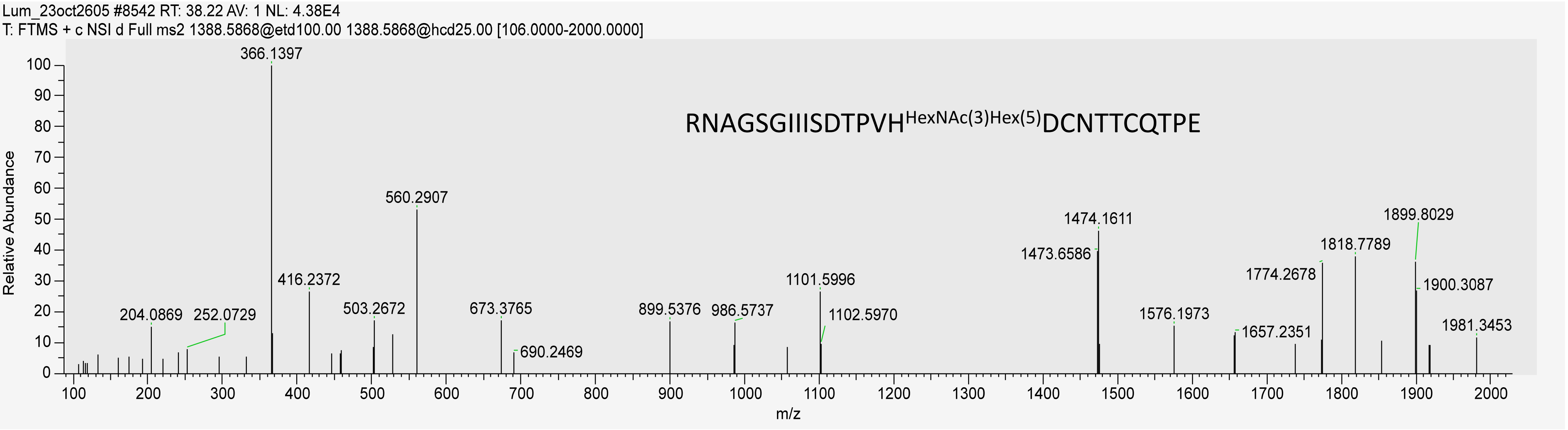

Lum_23oct2605 #8542 RT: 38.22 AV: 1 NL: 4.38E4
T: FTMS + c NSI d Full ms2 1388.5868@etd100.00 1388.5868@hcd25.00 [106.0000-2000.0000]
RNAGSGIIISDTPVH HexNAc(3)Hex(5) DCNTTCQTPE
Relative Abundance
m/z
366.1397
560.2907
416.2372
204.0869
252.0729
503.2672
673.3765
690.2469
899.5376
986.5737
1101.5996
1102.5970
1473.6586
1474.1611
1576.1973
1657.2351
1774.2678
1818.7789
1899.8029
1900.3087
1981.3453

Lum_23oct2605 #8530 RT: 38.18 AV: 1 NL: 3.10E4
T: FTMS + c NSI d Full ms2 1437.2744@etd100.00 1437.2744@hcd25.00 [106.0000-2000.0000]

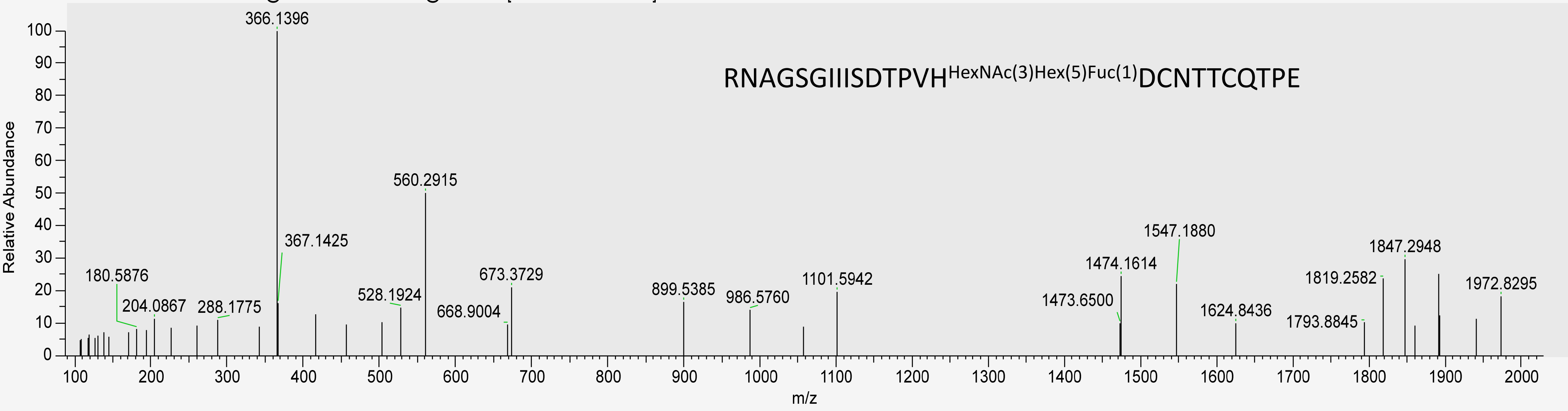

Lum_23oct2605 #9742 RT: 42.56 AV: 1 NL: 1.88E4
T: FTMS + c NSI d Full ms2 1485.9550@etd100.00 1485.9550@hcd25.00 [106.0000-2000.0000]

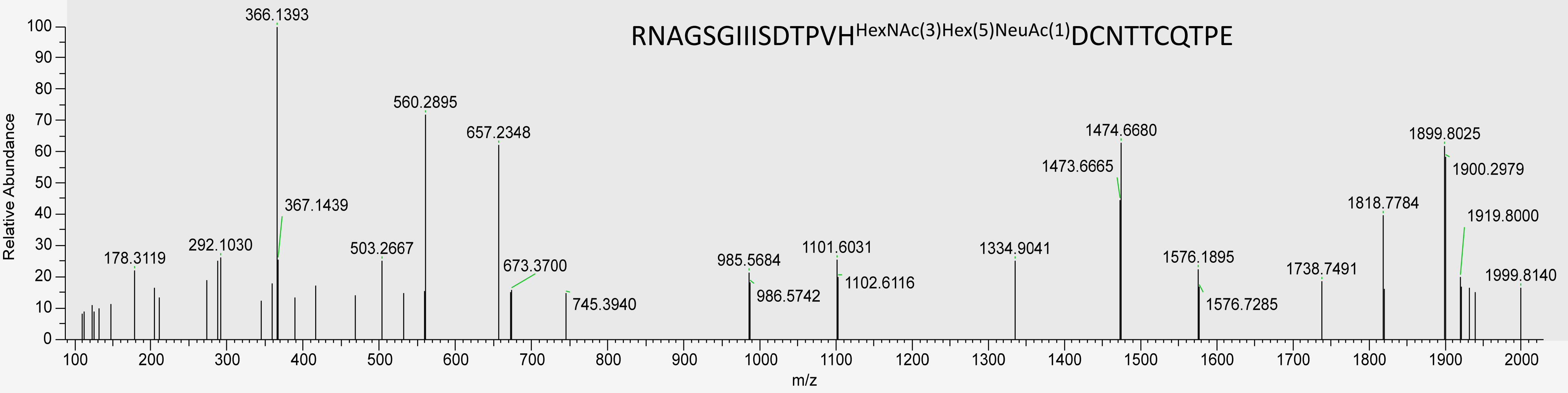

Lum_23oct2605 #8486 RT: 38.02 AV: 1 NL: 7.31E4
T: FTMS + c NSI d Full ms2 1443.2725@etd100.00 1443.2725@hcd25.00 [106.0000-2000.0000]

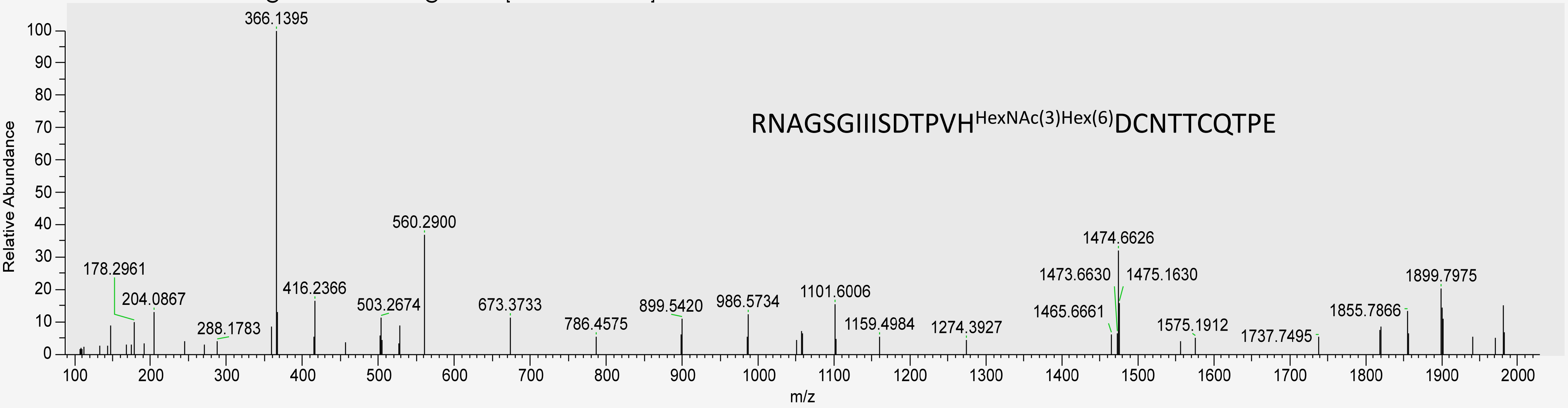

Lum_23oct2605 #9694 RT: 42.38 AV: 1 NL: 3.00E4
T: FTMS + c NSI d Full ms2 1539.9705@etd100.00 1539.9705@hcd25.00 [106.0000-2000.0000]

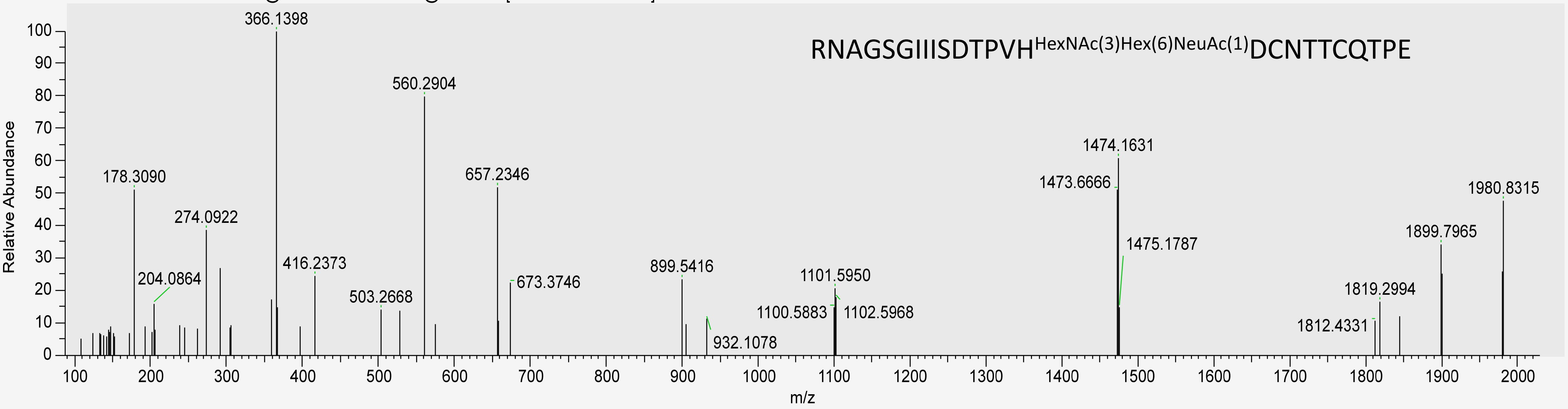

Lum_23oct2605 #8546 RT: 38.24 AV: 1 NL: 2.09E5
T: FTMS + c NSI d Full ms2 1396.9301@etd100.00 1396.9301@hcd25.00 [106.0000-2000.0000]

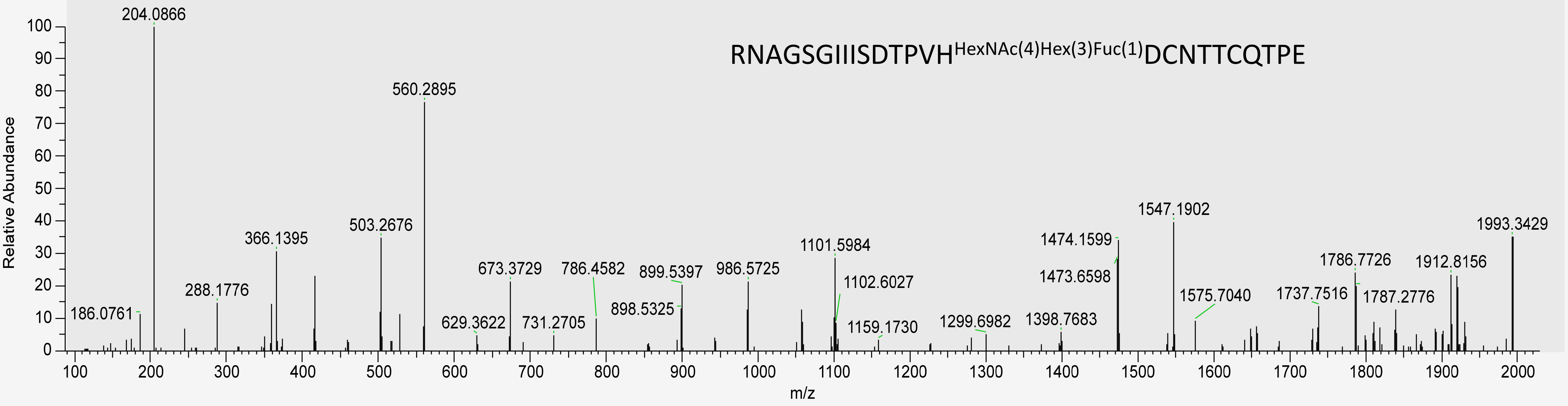

Lum_23oct2605 #8501 RT: 38.07 AV: 1 NL: 3.57E5
T: FTMS + c NSI d Full ms2 1451.2811@etd100.00 1451.2811@hcd25.00 [106.0000-2000.0000]

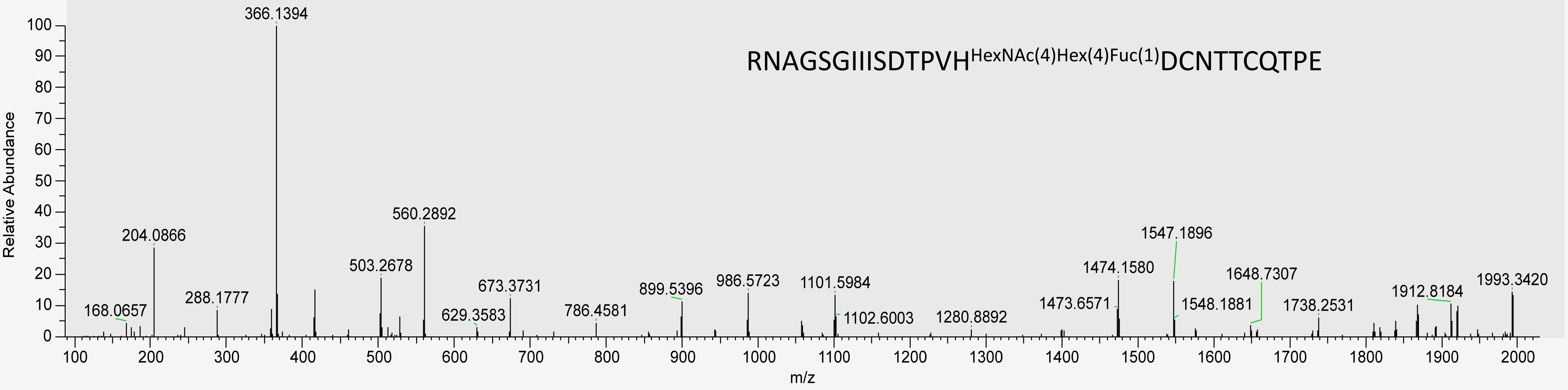

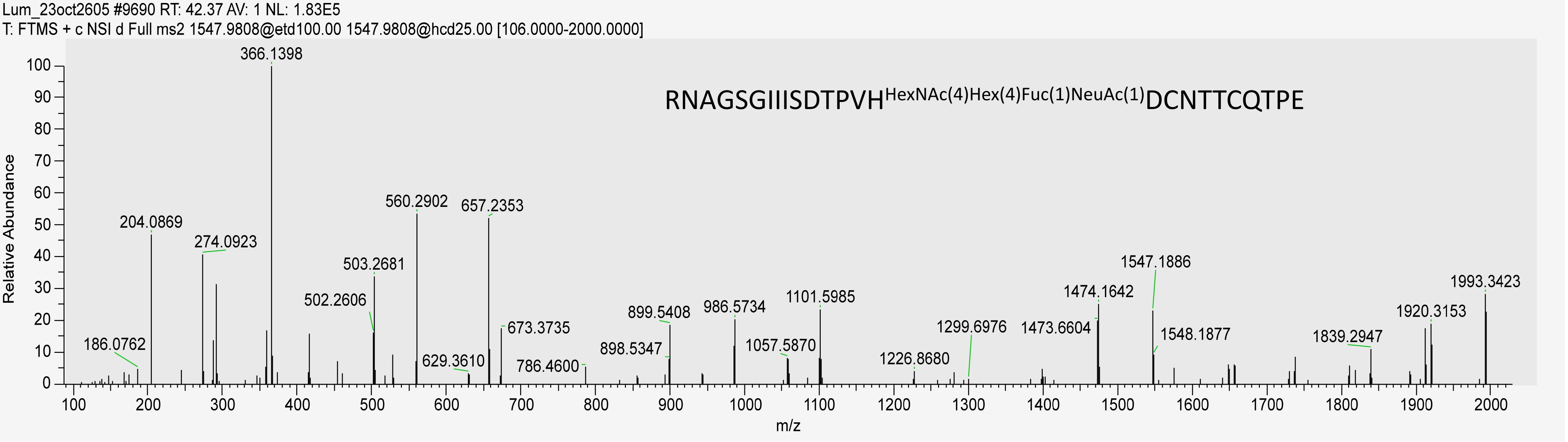
Lum_23oct2605 #9690 RT: 42.37 AV: 1 NL: 1.83E5
T: FTMS + c NSI d Full ms2 1547.9808@etd100.00 1547.9808@hcd25.00 [106.0000-2000.0000]
RNAGSGIIISDTPVH HexNAc(4)Hex(4)Fuc(1)NeuAc(1) DCNTTCQTPE
Relative Abundance
m/z
366.1398
204.0869
274.0923
560.2902
657.2353
503.2681
502.2606
186.0762
629.3610
673.3735
786.4600
898.5347
899.5408
986.5734
1057.5870
1101.5985
1226.8680
1299.6976
1473.6604
1474.1642
1547.1886
1548.1877
1839.2947
1920.3153
1993.3423

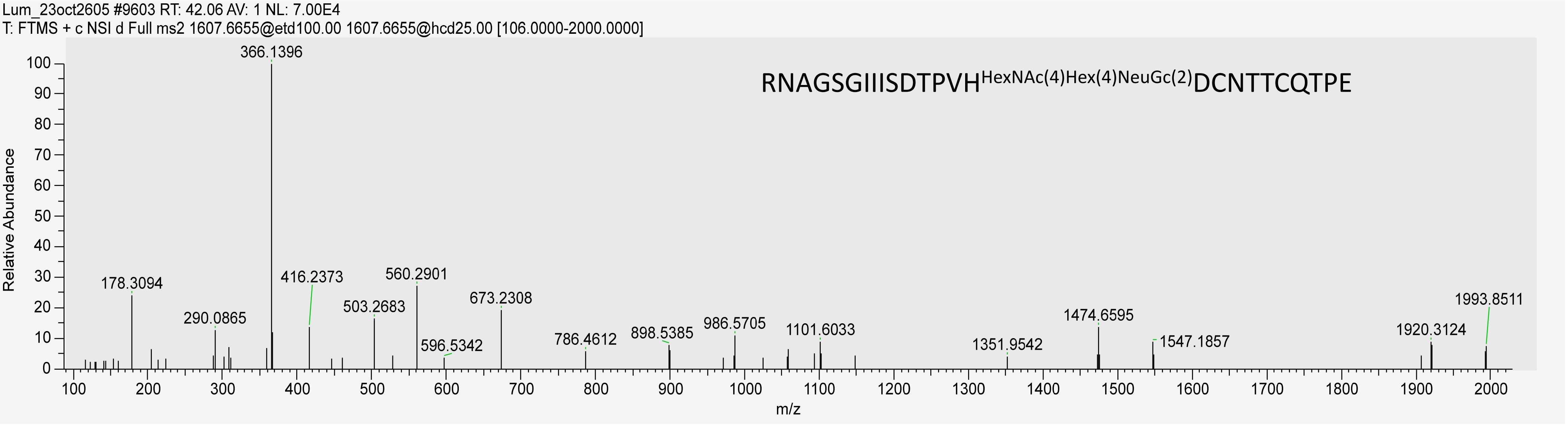
Lum_23oct2605 #9603 RT: 42.06 AV: 1 NL: 7.00E4
T: FTMS + c NSI d Full ms2 1607.6655@etd100.00 1607.6655@hcd25.00 [106.0000-2000.0000]
RNAGSGIIISDTPVH HexNAc(4)Hex(4)NeuGc(2) DCNTTCQTPE
Relative Abundance
m/z
366.1396
178.3094
290.0865
416.2373
503.2683
560.2901
596.5342
673.2308
786.4612
898.5385
986.5705
1101.6033
1351.9542
1474.6595
1547.1857
1920.3124
1993.8511

Lum_23oct2605 #8502 RT: 38.08 AV: 1 NL: 6.39E4
T: FTMS + c NSI d Full ms2 1456.6115@etd100.00 1456.6115@hcd25.00 [106.0000-2000.0000]

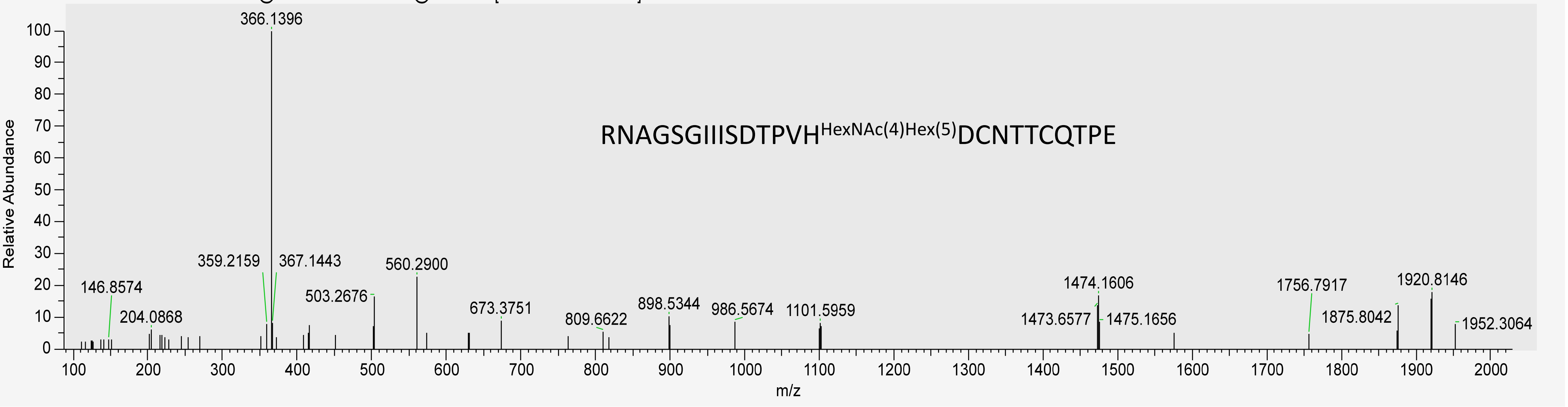

Lum_23oct2605 #8430 RT: 37.82 AV: 1 NL: 2.11E6
T: FTMS + c NSI d Full ms2 1505.6382@etd100.00 1505.6382@hcd25.00 [106.0000-2000.0000]

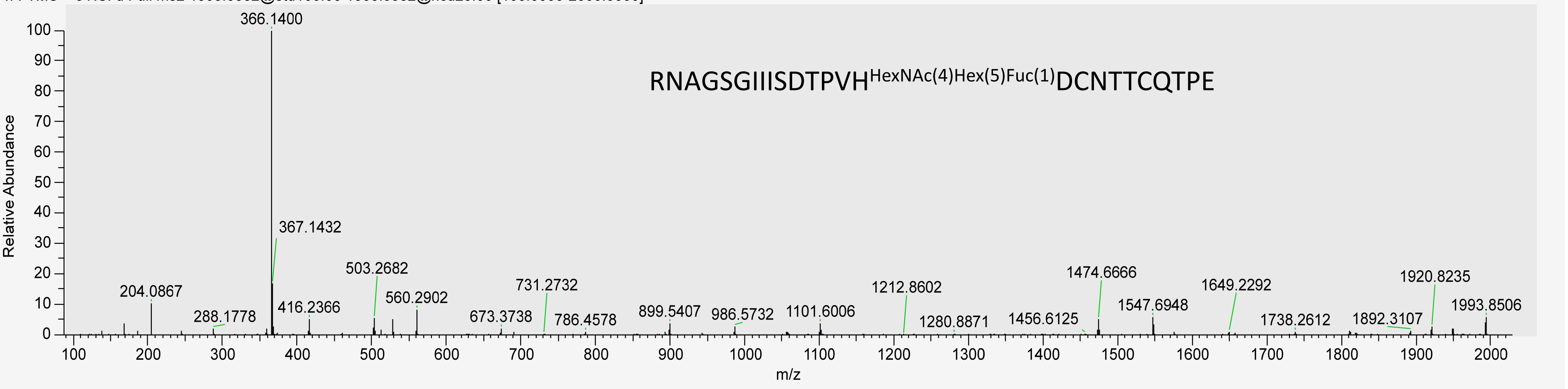

Lum_23oct2605 #9601 RT: 42.05 AV: 1 NL: 1.14E6
T: FTMS + c NSI d Full ms2 1602.0009@etd100.00 1602.0009@hcd25.00 [106.0000-2000.0000]

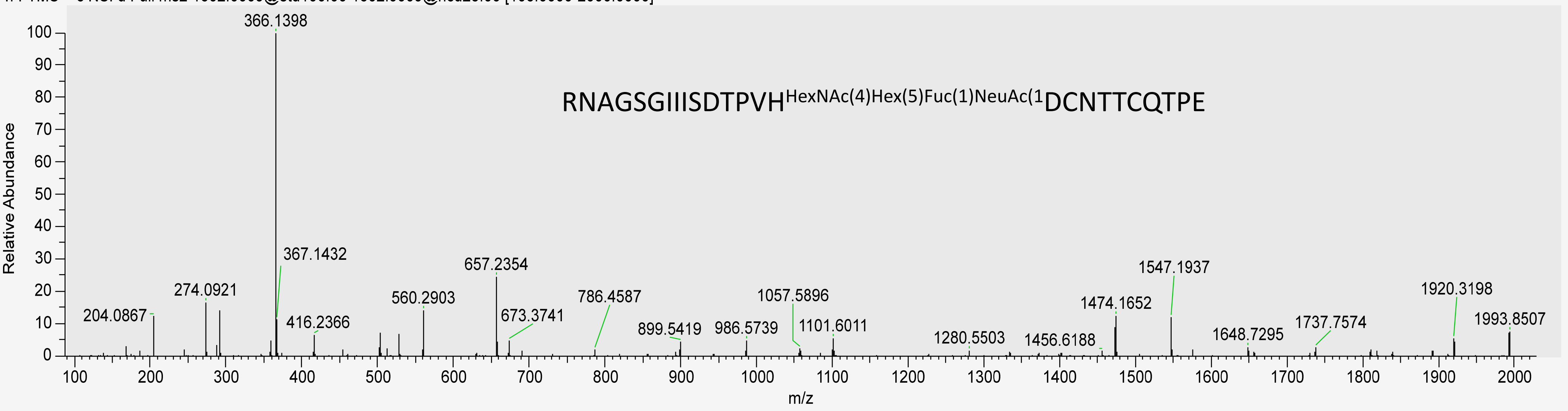

Lum_23oct2605 #11018 RT: 47.17 AV: 1 NL: 1.26E5
T: FTMS + c NSI d Full ms2 1699.3647@etd100.00 1699.3647@hcd25.00 [106.0000-2000.0000]

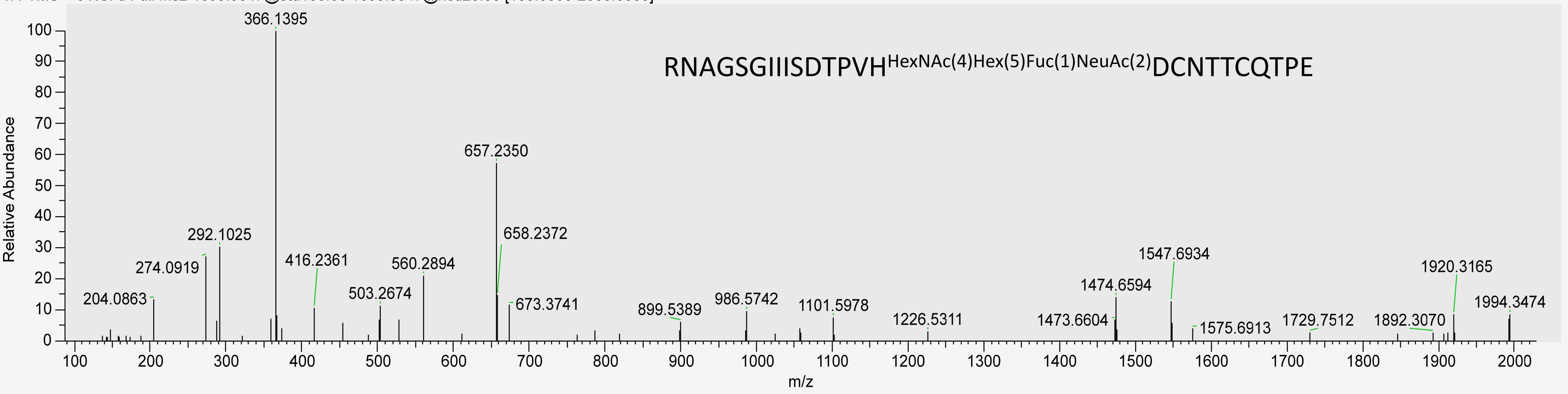

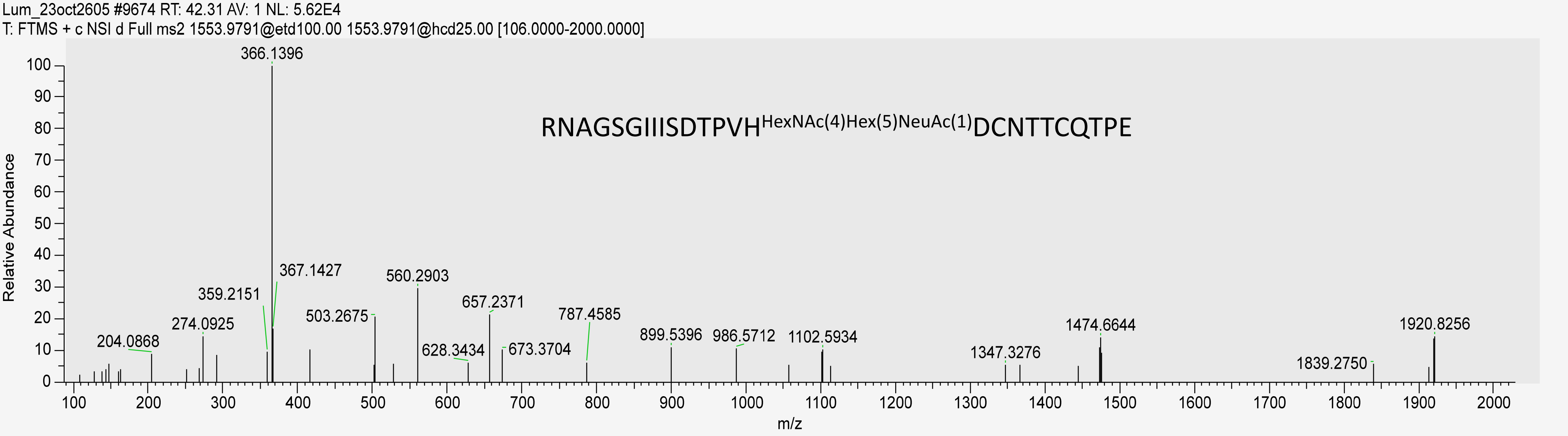
Lum_23oct2605 #9674 RT: 42.31 AV: 1 NL: 5.62E4
T: FTMS + c NSI d Full ms2 1553.9791@etd100.00 1553.9791@hcd25.00 [106.0000-2000.0000]
RNAGSGIIISDTPVH HexNAc(4)Hex(5)NeuAc(1) DCNTTCQTPE
Relative Abundance
m/z
366.1396
367.1427
359.2151
560.2903
657.2371
503.2675
274.0925
204.0868
787.4585
628.3434
673.3704
899.5396
986.5712
1102.5934
1347.3276
1474.6644
1839.2750
1920.8256

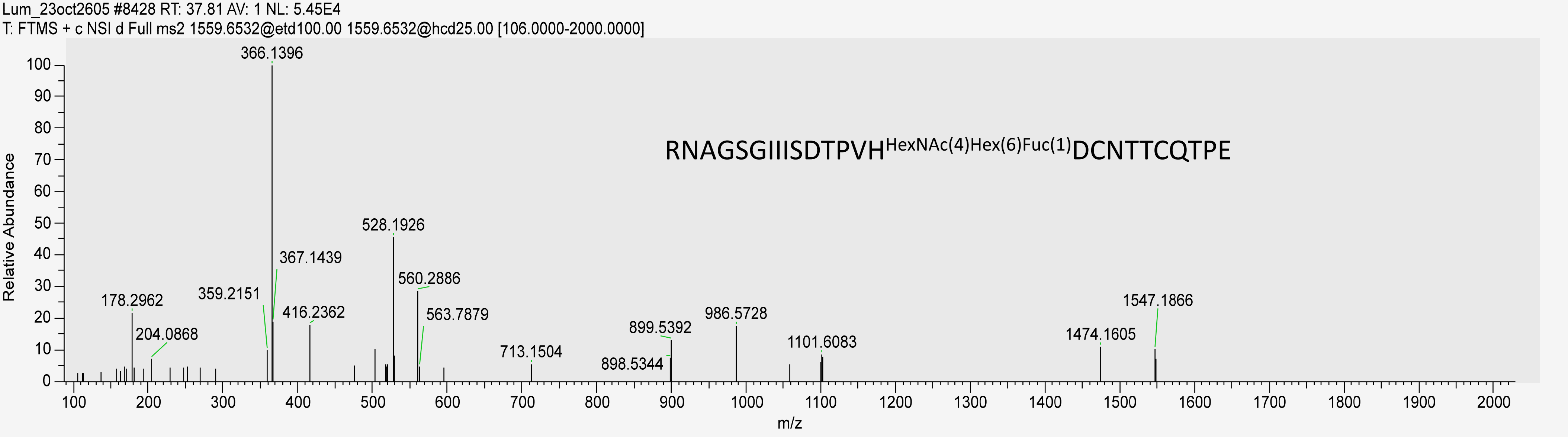
Lum_23oct2605 #8428 RT: 37.81 AV: 1 NL: 5.45E4
T: FTMS + c NSI d Full ms2 1559.6532@etd100.00 1559.6532@hcd25.00 [106.0000-2000.0000]
RNAGSGIIISDTPVH HexNAc(4)Hex(6)Fuc(1) DCNTTCQTPE
Relative Abundance
m/z
366.1396
367.1439
528.1926
560.2886
359.2151
178.2962
416.2362
563.7879
204.0868
899.5392
986.5728
713.1504
898.5344
1101.6083
1474.1605
1547.1866

Lum_23oct2605 #8517 RT: 38.13 AV: 1 NL: 1.07E5
T: FTMS + c NSI d Full ms2 1465.2926@etd100.00 1465.2926@hcd25.00 [106.0000-2000.0000]

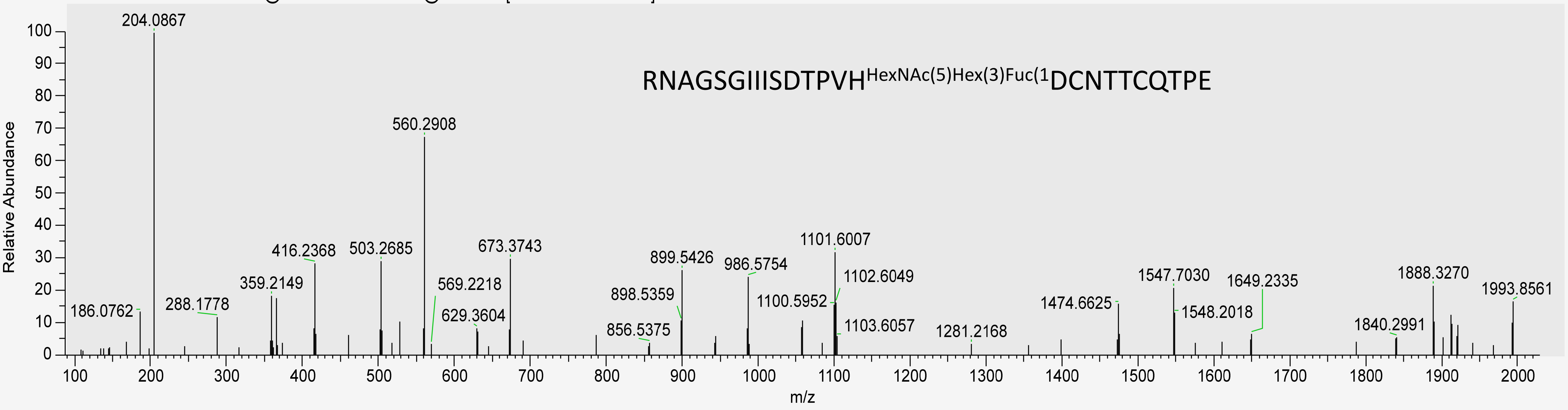

Lum_23oct2605 #8479 RT: 37.99 AV: 1 NL: 7.74E4
T: FTMS + c NSI d Full ms2 1518.9711@etd100.00 1518.9711@hcd25.00 [106.0000-2000.0000]

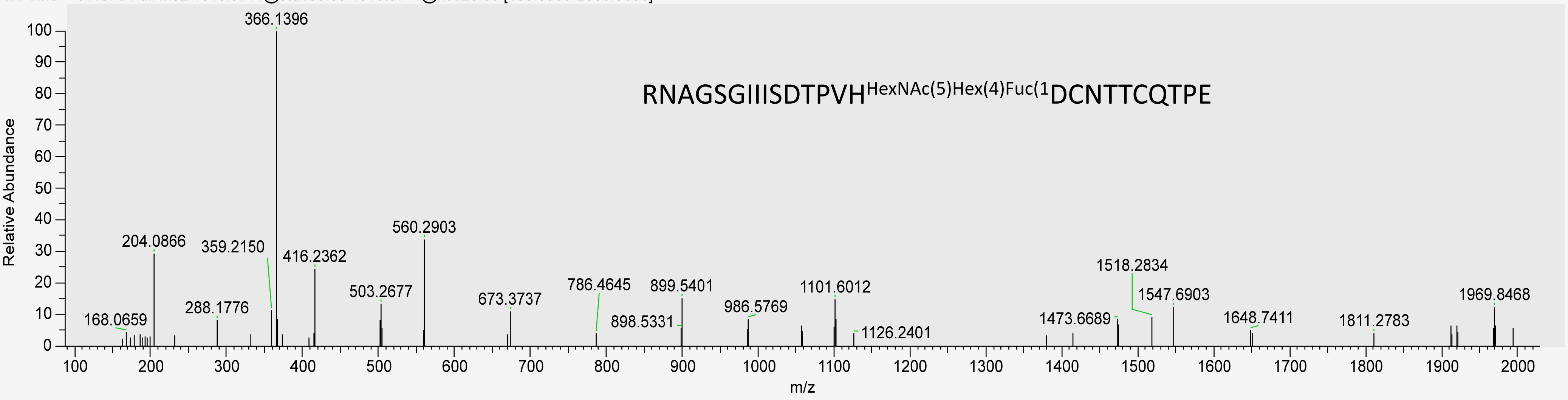

Lum_23oct2605 #9689 RT: 42.37 AV: 1 NL: 4.10E4
T: FTMS + c NSI d Full ms2 1616.0061@etd100.00 1616.0061@hcd25.00 [106.0000-2000.0000]

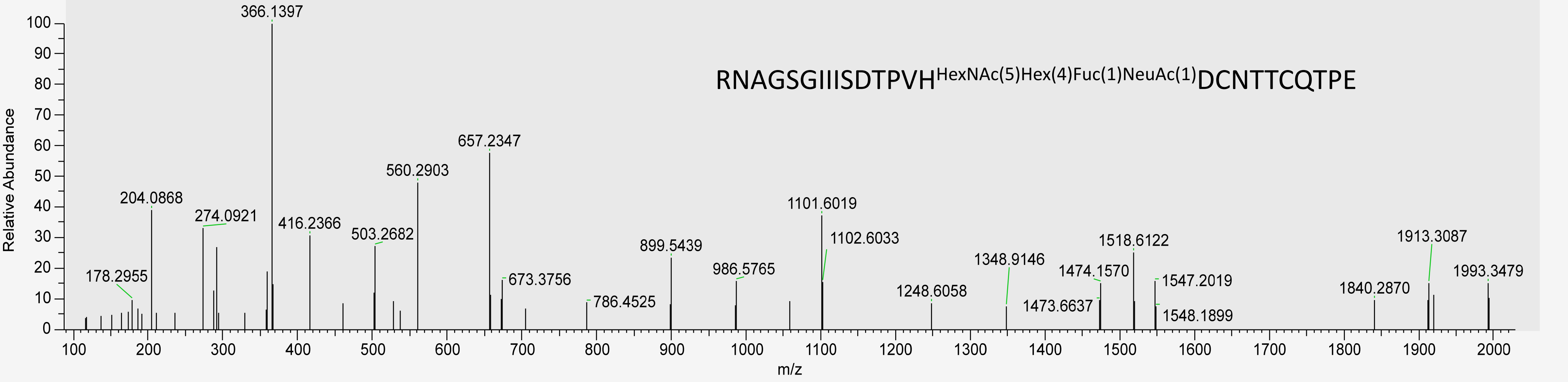

Lum_23oct2605 #8434 RT: 37.83 AV: 1 NL: 8.64E4
T: FTMS + c NSI d Full ms2 1572.6589@etd100.00 1572.6589@hcd25.00 [106.0000-2000.0000]

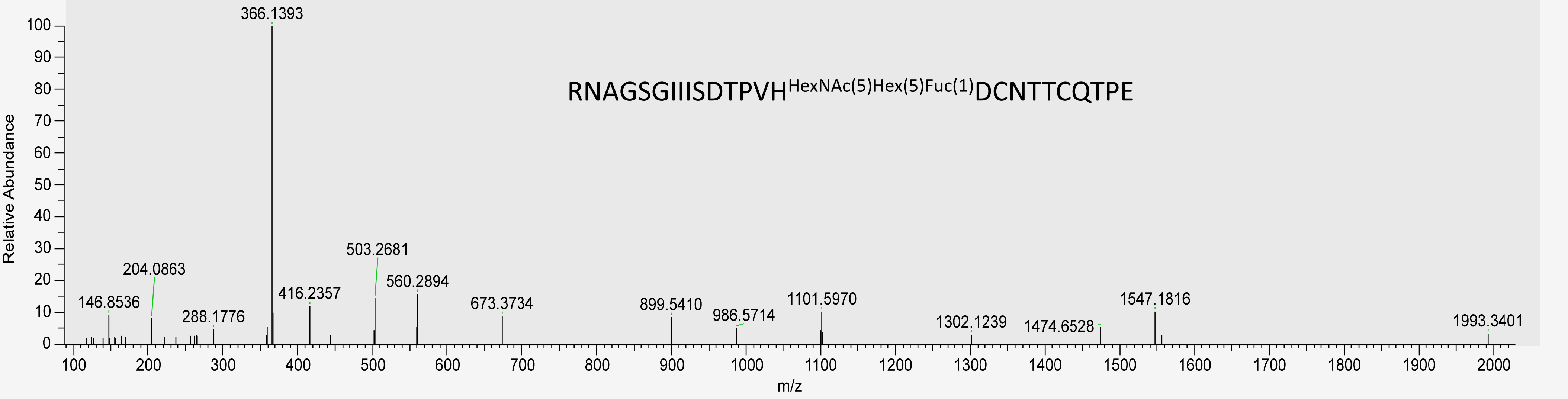

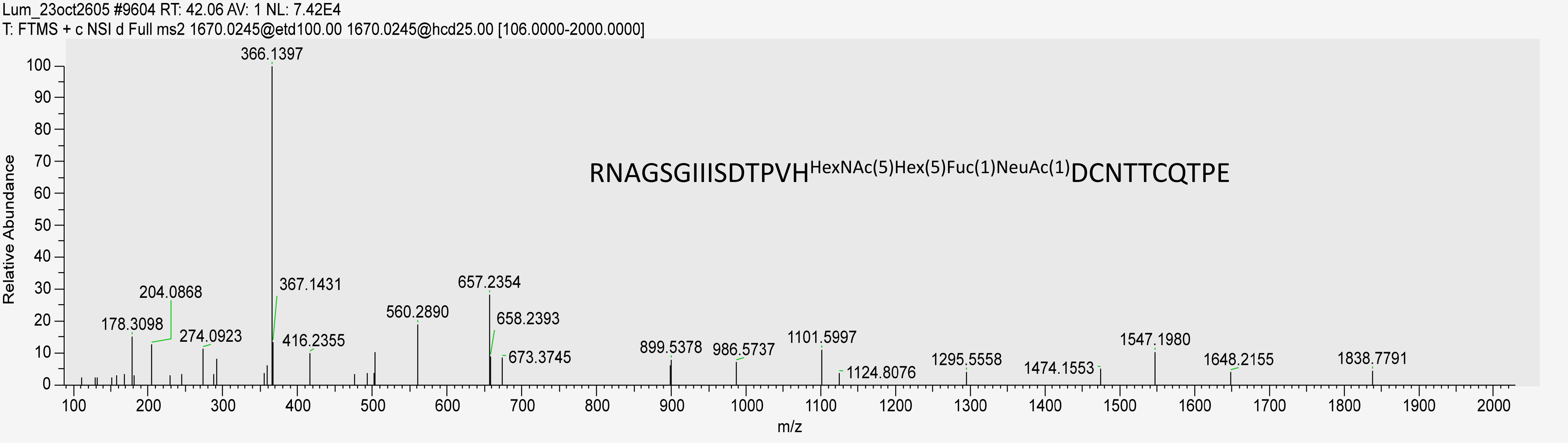
Lum_23oct2605 #9604 RT: 42.06 AV: 1 NL: 7.42E4
T: FTMS + c NSI d Full ms2 1670.0245@etd100.00 1670.0245@hcd25.00 [106.0000-2000.0000]
RNAGSGIIISDTPVH HexNAc(5)Hex(5)Fuc(1)NeuAc(1) DCNTTCQTPE
366.1397
367.1431
204.0868
178.3098
274.0923
416.2355
560.2890
657.2354
658.2393
673.3745
899.5378
986.5737
1101.5997
1124.8076
1295.5558
1474.1553
1547.1980
1648.2155
1838.7791
Relative Abundance
m/z

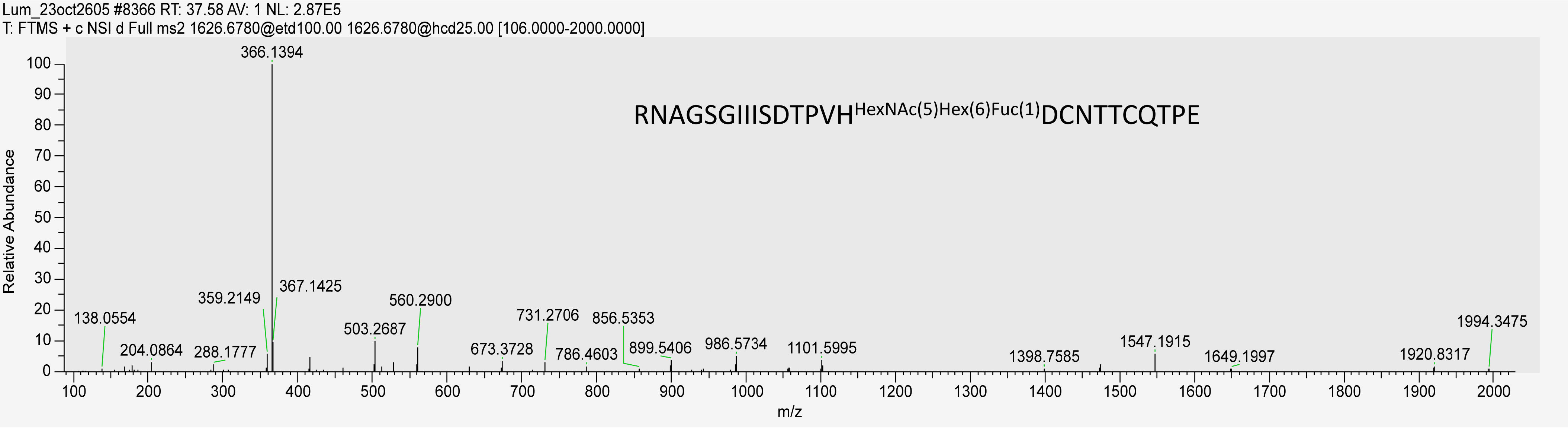
Lum_23oct2605 #8366 RT: 37.58 AV: 1 NL: 2.87E5
T: FTMS + c NSI d Full ms2 1626.6780@etd100.00 1626.6780@hcd25.00 [106.0000-2000.0000]
RNAGSGIIISDTPVH HexNAc(5)Hex(6)Fuc(1) DCNTTCQTPE
366.1394
367.1425
359.2149
138.0554
204.0864
288.1777
503.2687
560.2900
673.3728
731.2706
786.4603
856.5353
899.5406
986.5734
1101.5995
1398.7585
1547.1915
1649.1997
1920.8317
1994.3475
Relative Abundance
m/z

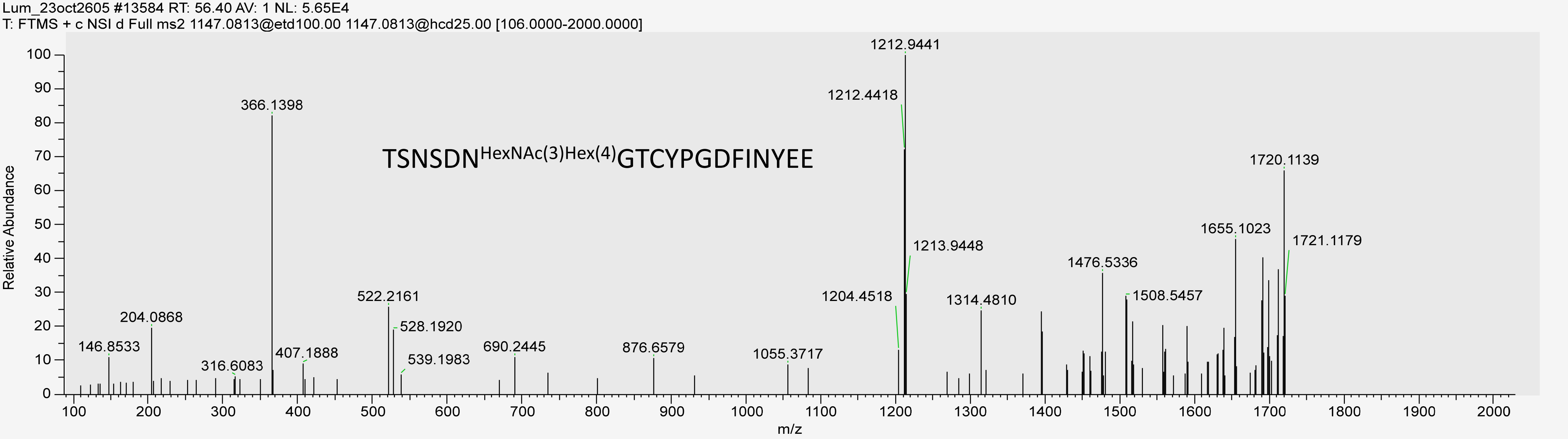
Lum_23oct2605 #13584 RT: 56.40 AV: 1 NL: 5.65E4
T: FTMS + c NSI d Full ms2 1147.0813@etd100.00 1147.0813@hcd25.00 [106.0000-2000.0000]
TSNSDN HexNAc(3)Hex(4) GTCYPGDFINYEE
Relative Abundance
m/z
1212.9441
1212.4418
366.1398
1720.1139
1655.1023
1721.1179
1213.9448
1476.5336
1508.5457
1204.4518
1314.4810
522.2161
204.0868
528.1920
146.8533
690.2445
876.6579
407.1888
539.1983
1055.3717
316.6083

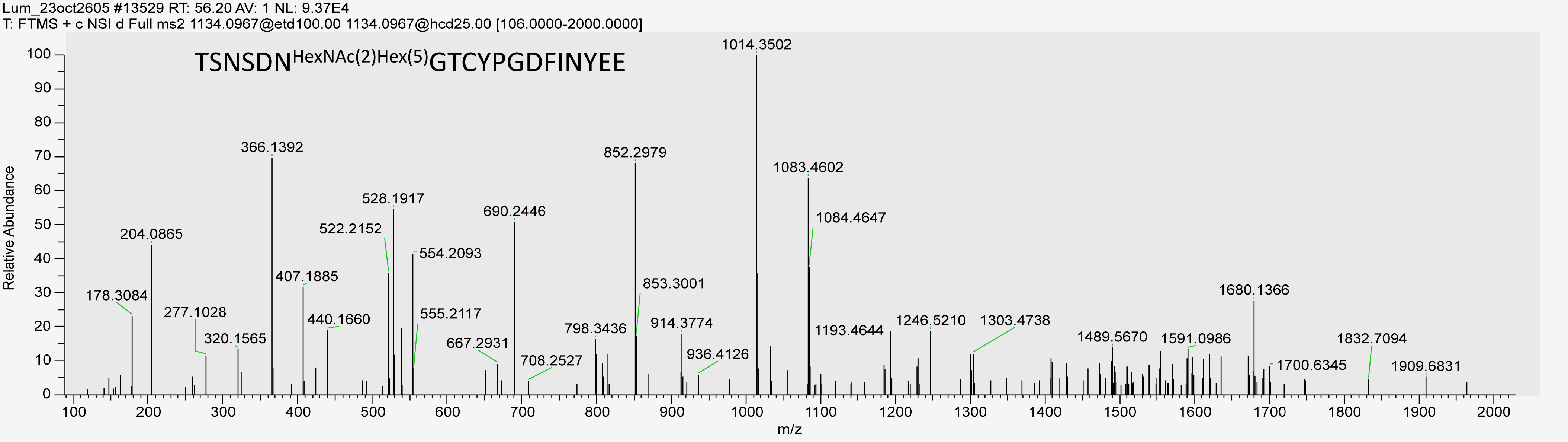
Lum_23oct2605 #13529 RT: 56.20 AV: 1 NL: 9.37E4
T: FTMS + c NSI d Full ms2 1134.0967@etd100.00 1134.0967@hcd25.00 [106.0000-2000.0000]
TSNSDN HexNAc(2)Hex(5) GTCYPGDFINYEE
Relative Abundance
m/z
1014.3502
366.1392
852.2979
1083.4602
528.1917
690.2446
522.2152
1084.4647
204.0865
554.2093
407.1885
853.3001
1680.1366
178.3084
277.1028
440.1660
555.2117
914.3774
798.3436
1246.5210
1303.4738
1193.4644
320.1565
667.2931
1489.5670
1591.0986
1832.7094
936.4126
708.2527
1700.6345
1909.6831

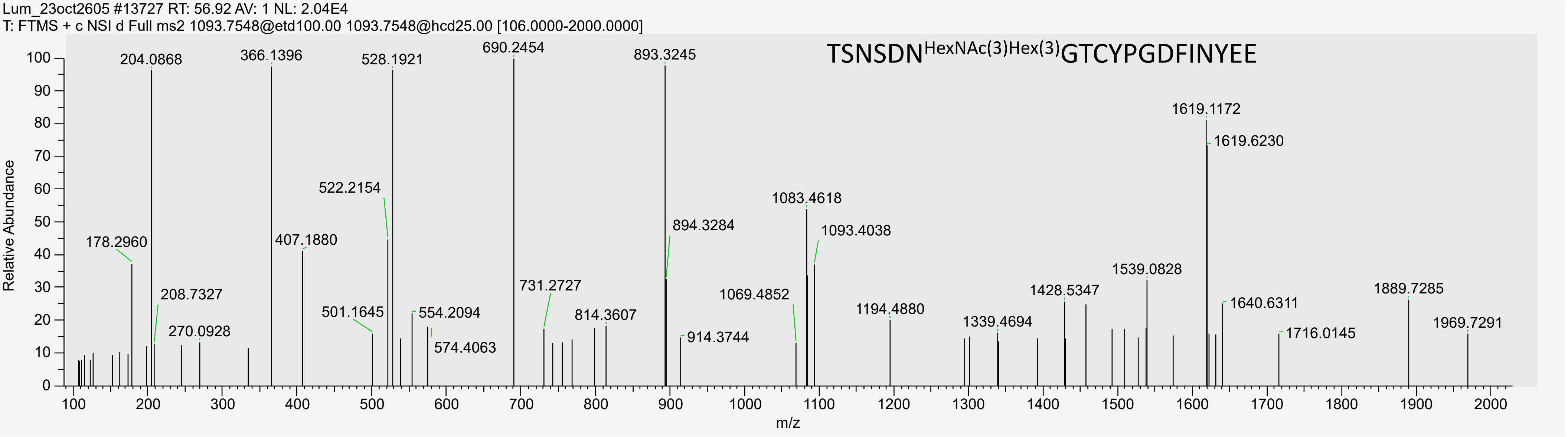
Lum_23oct2605 #13727 RT: 56.92 AV: 1 NL: 2.04E4
T: FTMS + c NSI d Full ms2 1093.7548@etd100.00 1093.7548@hcd25.00 [106.0000-2000.0000]
TSNSDN HexNAc(3)Hex(3) GTCYPGDFINYEE
Relative Abundance
m/z
204.0868
366.1396
528.1921
690.2454
893.3245
1619.1172
1619.6230
522.2154
1083.4618
894.3284
1093.4038
178.2960
407.1880
1539.0828
208.7327
731.2727
1069.4852
1428.5347
1889.7285
501.1645
554.2094
814.3607
1194.4880
1640.6311
270.0928
1339.4694
1969.7291
914.3744
574.4063
1716.0145

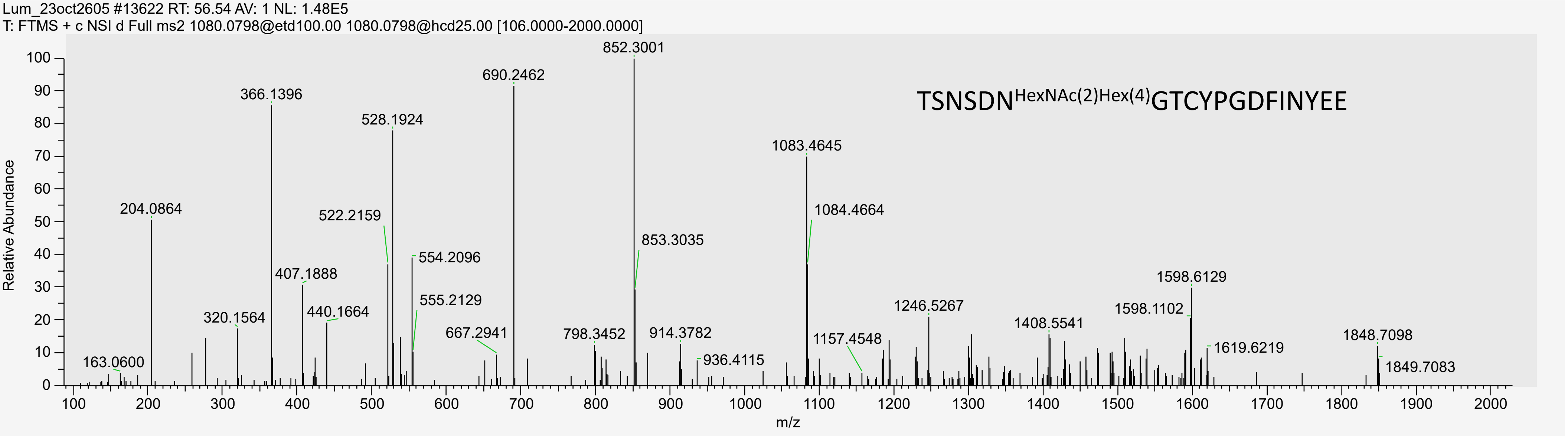
Lum_23oct2605 #13622 RT: 56.54 AV: 1 NL: 1.48E5
T: FTMS + c NSI d Full ms2 1080.0798@etd100.00 1080.0798@hcd25.00 [106.0000-2000.0000]
TSNSDN HexNAc(2)Hex(4) GTCYPGDFINYEE
Relative Abundance
m/z
852.3001
690.2462
366.1396
528.1924
1083.4645
204.0864
522.2159
1084.4664
554.2096
853.3035
407.1888
1598.6129
555.2129
1246.5267
320.1564
440.1664
1598.1102
1408.5541
667.2941
798.3452
914.3782
1848.7098
1157.4548
1619.6219
163.0600
936.4115
1849.7083

Lum_23oct2605 #13642 RT: 56.61 AV: 1 NL: 5.40E4
T: FTMS + c NSI d Full ms2 1161.4478@etd100.00 1161.4478@hcd25.00 [106.0000-2000.0000]

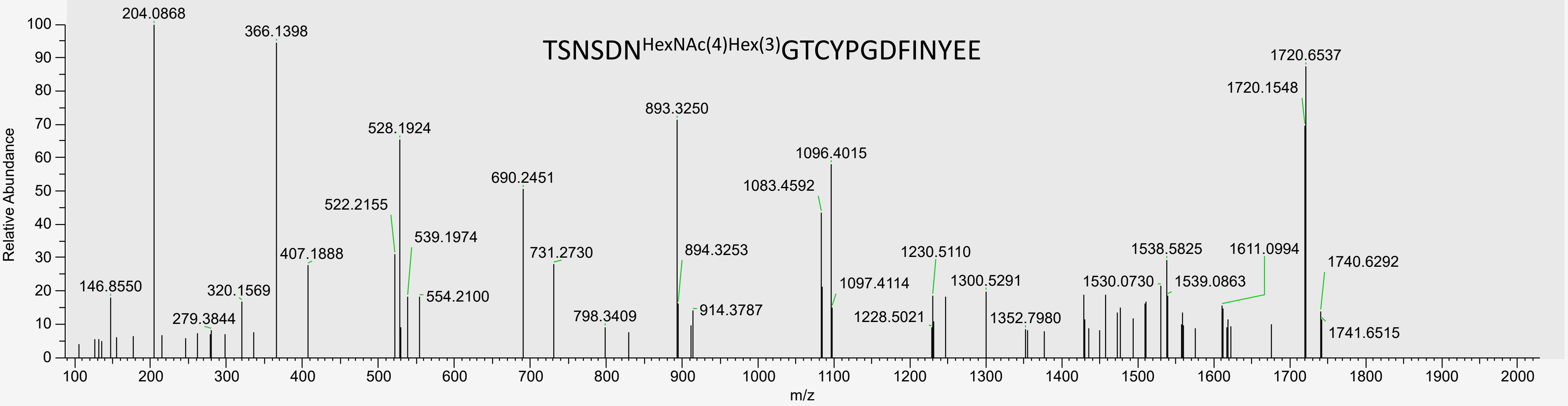

Lum_23oct2605 #13436 RT: 55.86 AV: 1 NL: 4.66E4
T: FTMS + c NSI d Full ms2 1188.4492@etd100.00 1188.4492@hcd25.00 [106.0000-2000.0000]

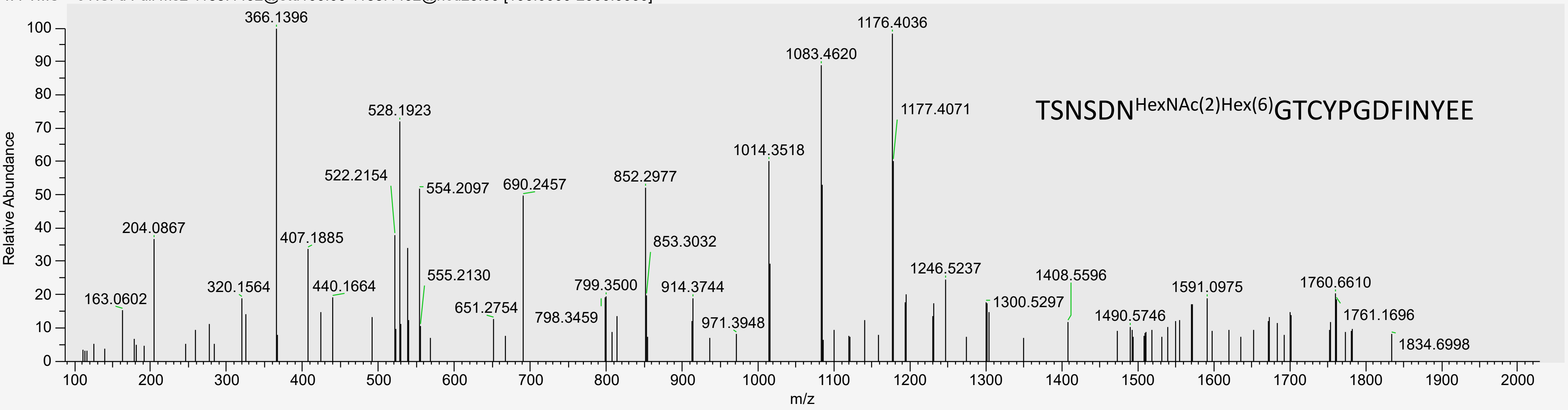

Lum_23oct2605 #13506 RT: 56.12 AV: 1 NL: 2.11E5
T: FTMS + c NSI d Full ms2 1202.1226@etd100.00 1202.1226@hcd25.00 [106.0000-2000.0000]

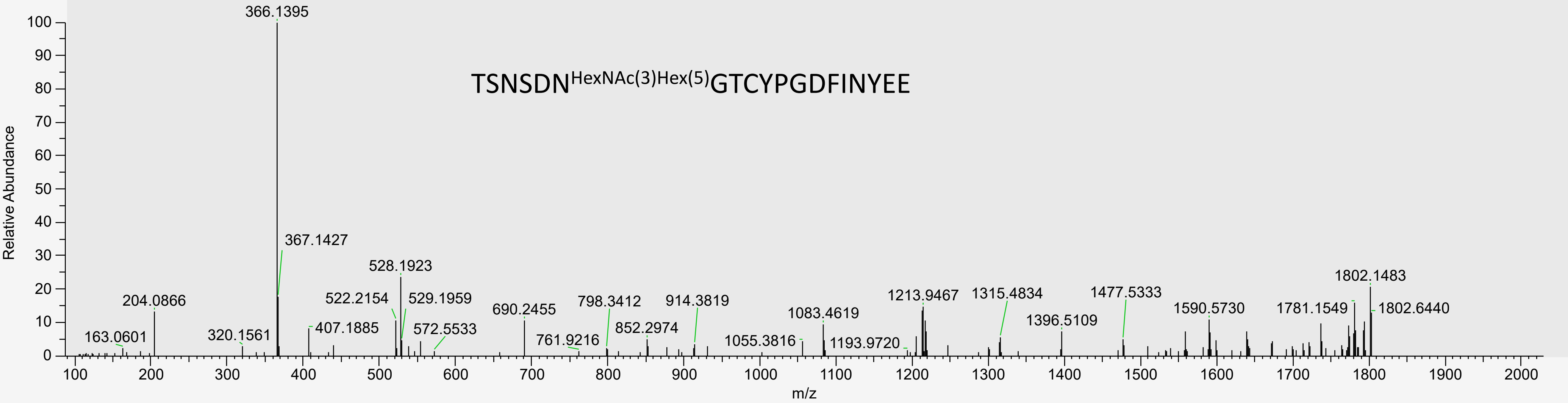

Lum_23oct2605 #13514 RT: 56.15 AV: 1 NL: 1.52E5
T: FTMS + c NSI d Full ms2 1216.1322@etd100.00 1216.1322@hcd25.00 [106.0000-2000.0000]

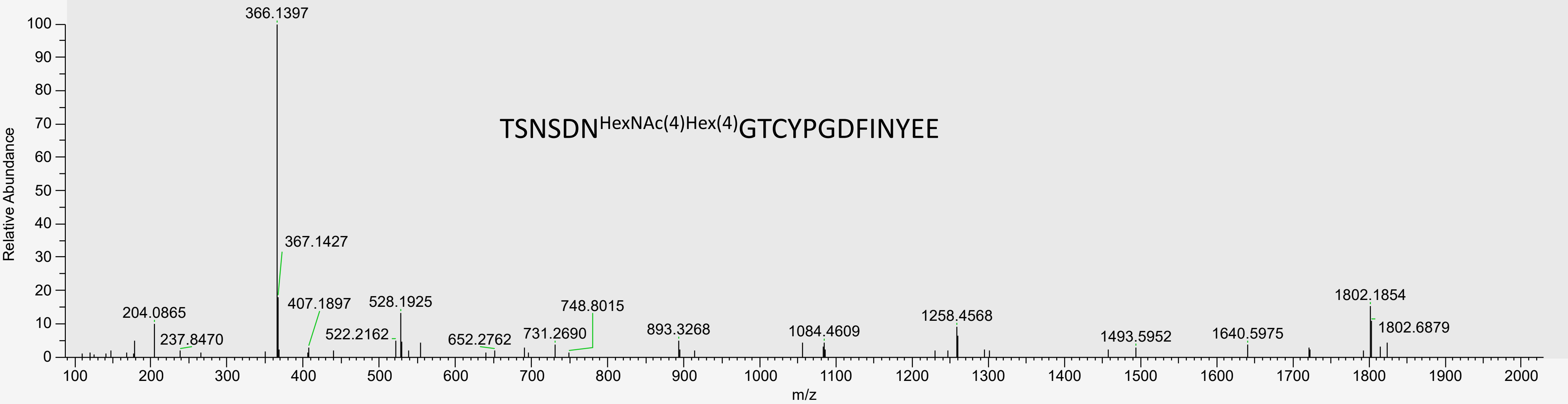

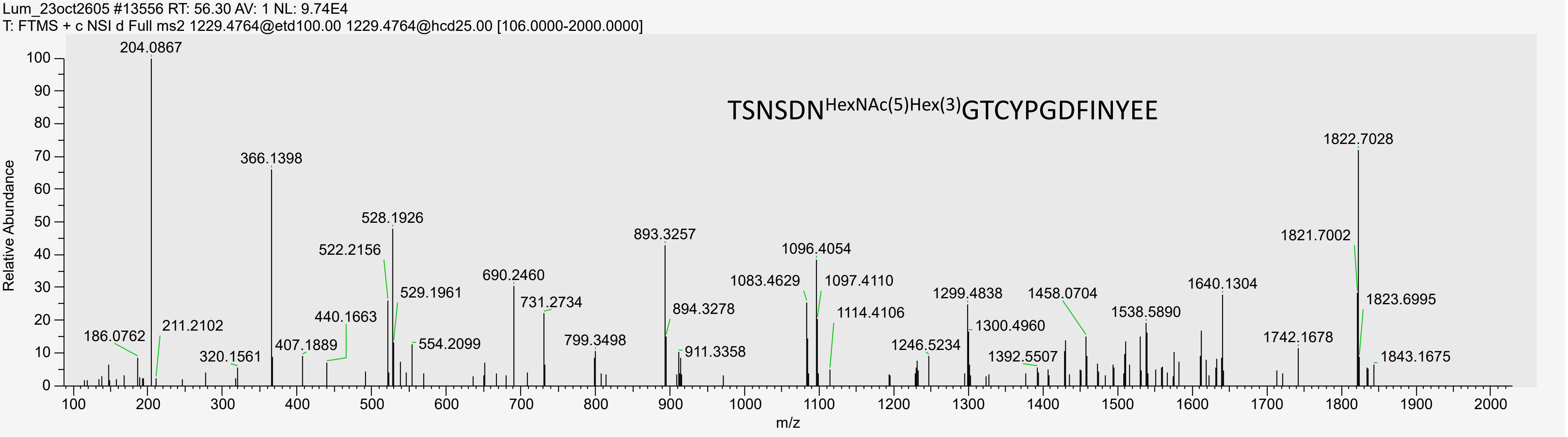
Lum_23oct2605 #13556 RT: 56.30 AV: 1 NL: 9.74E4
T: FTMS + c NSI d Full ms2 1229.4764@etd100.00 1229.4764@hcd25.00 [106.0000-2000.0000]
TSNSDN HexNAc(5)Hex(3) GTCYPGDFINYEE
204.0867
366.1398
528.1926
522.2156
529.1961
186.0762
211.2102
320.1561
407.1889
440.1663
554.2099
690.2460
731.2734
799.3498
893.3257
894.3278
911.3358
1083.4629
1096.4054
1097.4110
1114.4106
1246.5234
1299.4838
1300.4960
1392.5507
1458.0704
1538.5890
1640.1304
1742.1678
1821.7002
1822.7028
1823.6995
1843.1675
Relative Abundance
m/z

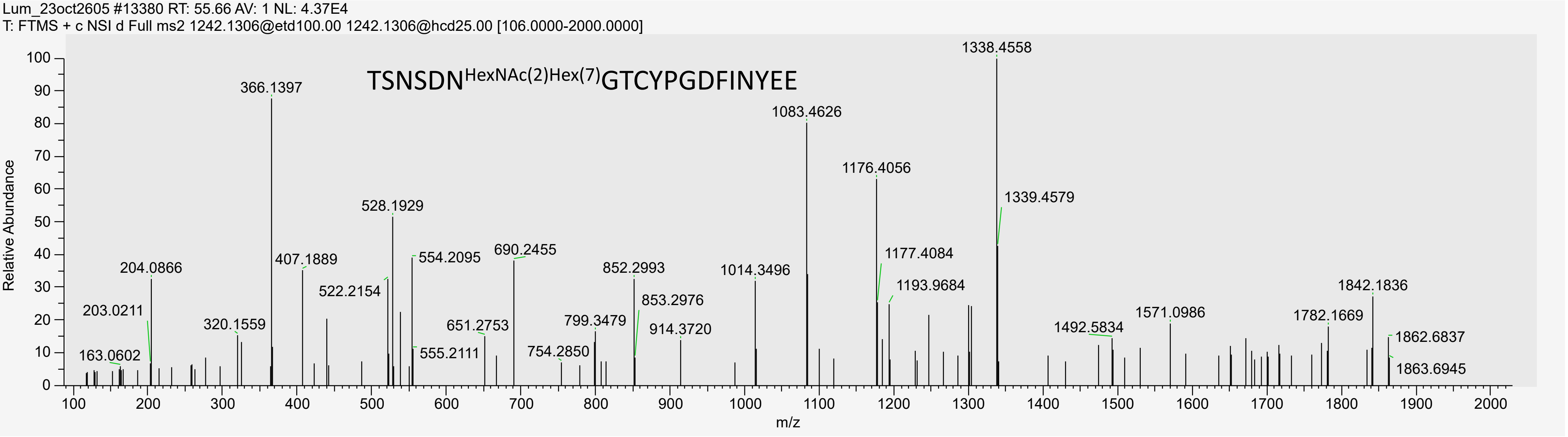
Lum_23oct2605 #13380 RT: 55.66 AV: 1 NL: 4.37E4
T: FTMS + c NSI d Full ms2 1242.1306@etd100.00 1242.1306@hcd25.00 [106.0000-2000.0000]
TSNSDN HexNAc(2)Hex(7) GTCYPGDFINYEE
1338.4558
366.1397
1083.4626
1176.4056
1339.4579
528.1929
204.0866
407.1889
522.2154
554.2095
690.2455
852.2993
1014.3496
1177.4084
1193.9684
1842.1836
203.0211
320.1559
651.2753
853.2976
799.3479
914.3720
1571.0986
1782.1669
1492.5834
163.0602
555.2111
754.2850
1862.6837
1863.6945
Relative Abundance
m/z

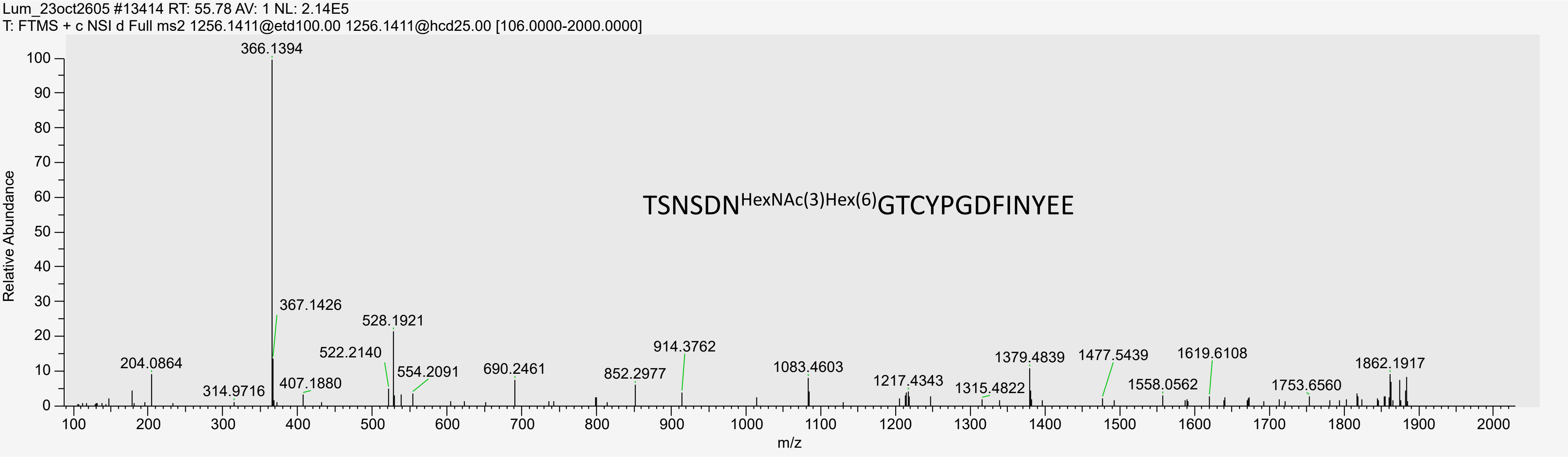
Lum_23oct2605 #13414 RT: 55.78 AV: 1 NL: 2.14E5
T: FTMS + c NSI d Full ms2 1256.1411@etd100.00 1256.1411@hcd25.00 [106.0000-2000.0000]
TSNSDN HexNAc(3)Hex(6) GTCYPGDFINYEE
Relative Abundance
m/z
366.1394
367.1426
528.1921
522.2140
554.2091
204.0864
314.9716
407.1880
690.2461
852.2977
914.3762
1083.4603
1217.4343
1315.4822
1379.4839
1477.5439
1558.0562
1619.6108
1753.6560
1862.1917

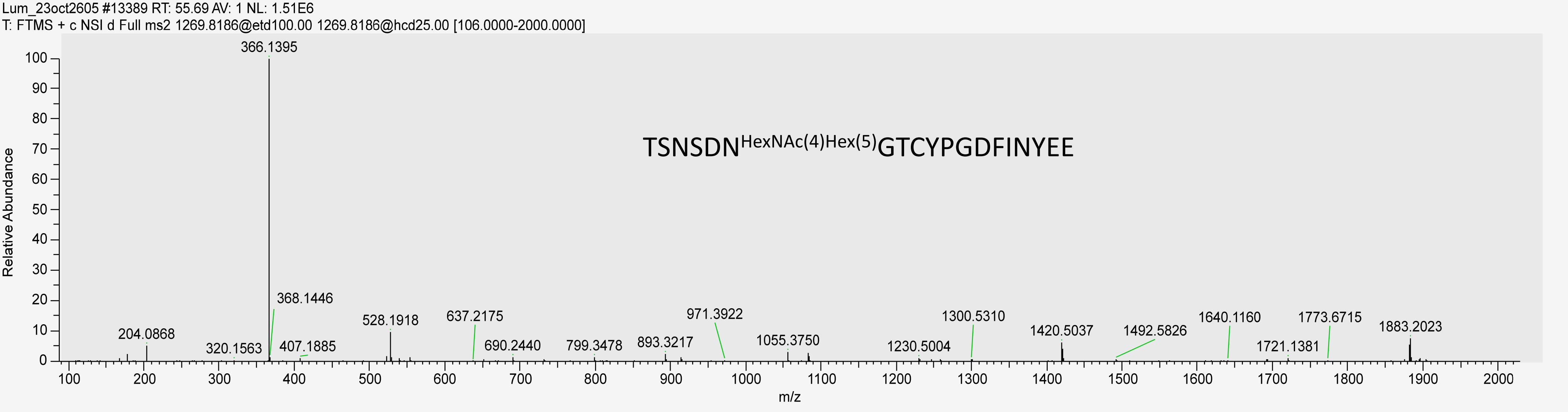
Lum_23oct2605 #13389 RT: 55.69 AV: 1 NL: 1.51E6
T: FTMS + c NSI d Full ms2 1269.8186@etd100.00 1269.8186@hcd25.00 [106.0000-2000.0000]
TSNSDN HexNAc(4)Hex(5) GTCYPGDFINYEE
Relative Abundance
m/z
366.1395
368.1446
204.0868
320.1563
407.1885
528.1918
637.2175
690.2440
799.3478
893.3217
971.3922
1055.3750
1230.5004
1300.5310
1420.5037
1492.5826
1640.1160
1721.1381
1773.6715
1883.2023

Lum_23oct2605 #13317 RT: 55.43 AV: 1 NL: 2.60E4
T: FTMS + c NSI d Full ms2 1296.1506@etd100.00 1296.1506@hcd25.00 [106.0000-2000.0000]

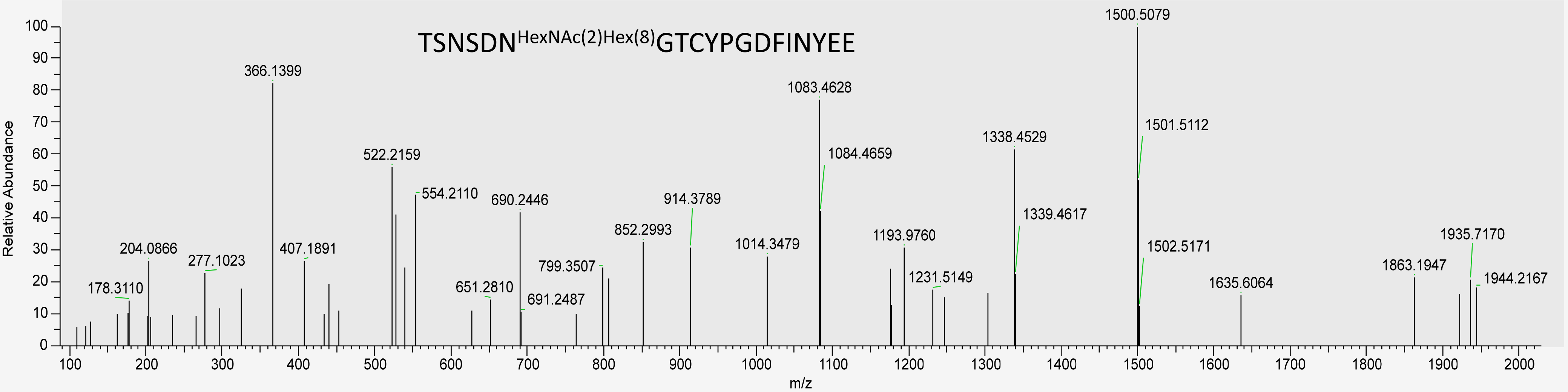

Lum_23oct2605 #13867 RT: 57.43 AV: 1 NL: 1.59E4
T: FTMS + c NSI d Full ms2 1026.3964@etd100.00 1026.3964@hcd25.00 [106.0000-2000.0000]

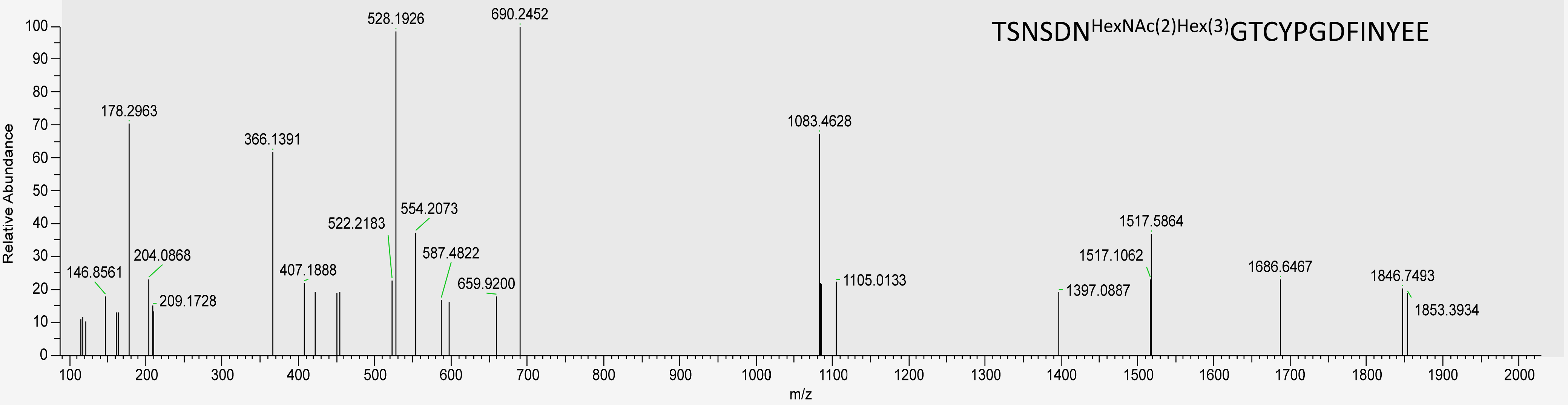

Lum_23oct2614 #14192 RT: 57.74 AV: 1 NL: 4.84E4
T: FTMS + c NSI d Full ms2 1078.1287@etd100.00 1078.1287@hcd25.00 [106.0000-2000.0000]

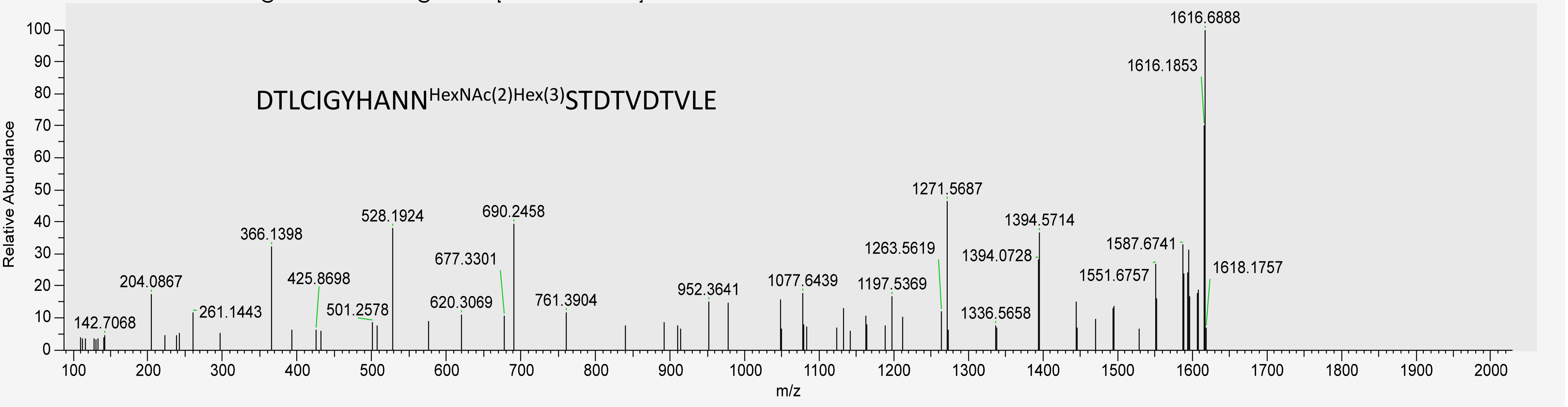

Lum_23oct2614 #14045 RT: 57.21 AV: 1 NL: 3.07E4
T: FTMS + c NSI d Full ms2 1248.1881@etd100.00 1248.1881@hcd25.00 [106.0000-2000.0000]

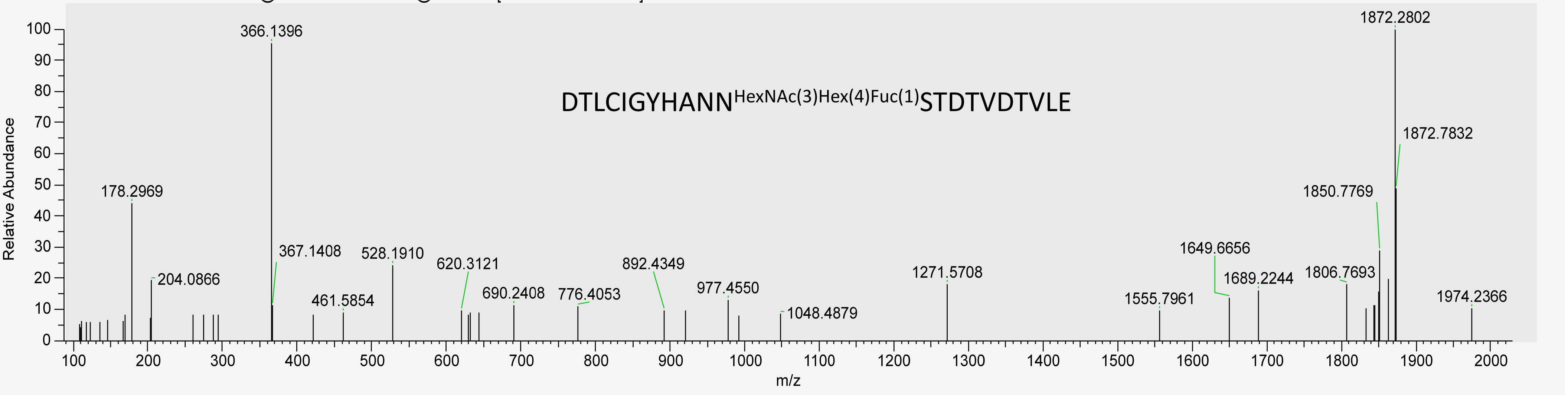

Lum_23oct2614 #13921 RT: 56.76 AV: 1 NL: 3.15E5
T: FTMS + c NSI d Full ms2 1316.5514@etd100.00 1316.5514@hcd25.00 [106.0000-2000.0000]

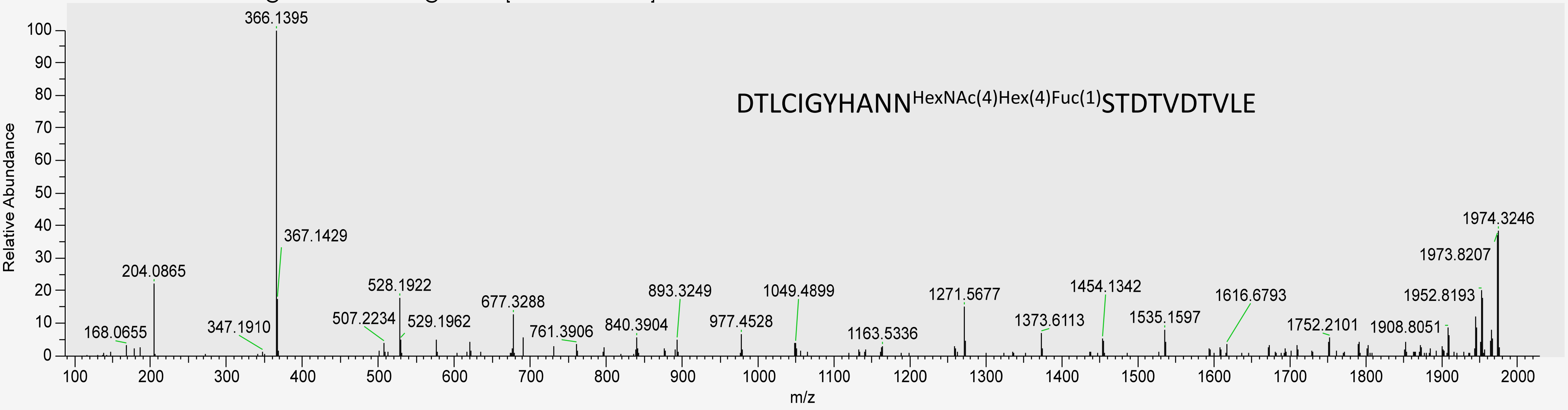

Lum_23oct2614 #15918 RT: 63.99 AV: 1 NL: 2.92E4
T: FTMS + c NSI d Full ms2 1412.9148@etd100.00 1412.9148@hcd25.00 [106.0000-2000.0000]

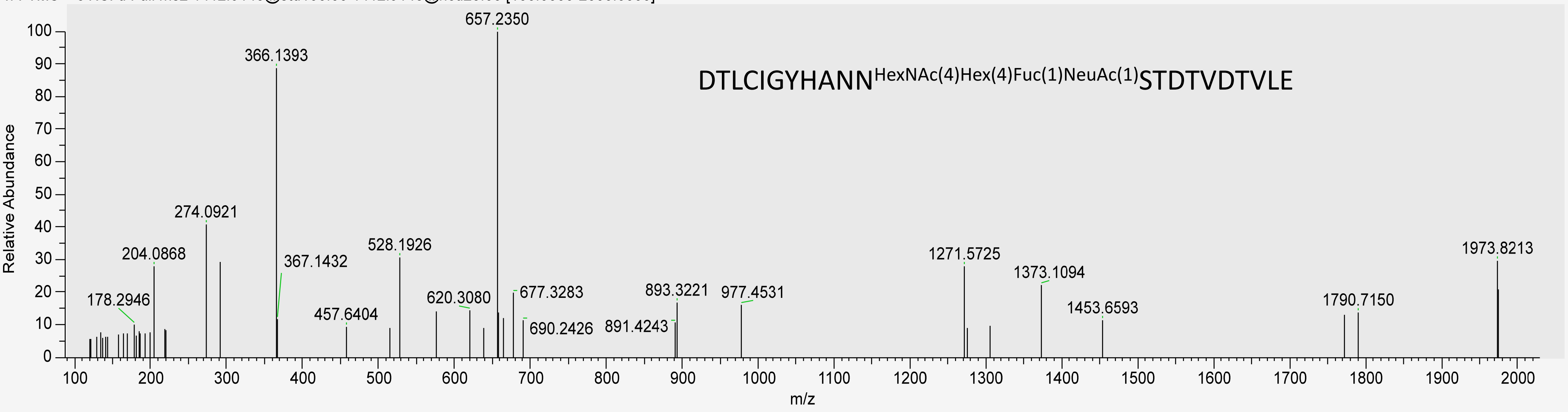

Lum_23oct2614 #13800 RT: 56.32 AV: 1 NL: 7.96E5
T: FTMS + c NSI d Full ms2 1369.9009@etd100.00 1369.9009@hcd25.00 [106.0000-2000.0000]

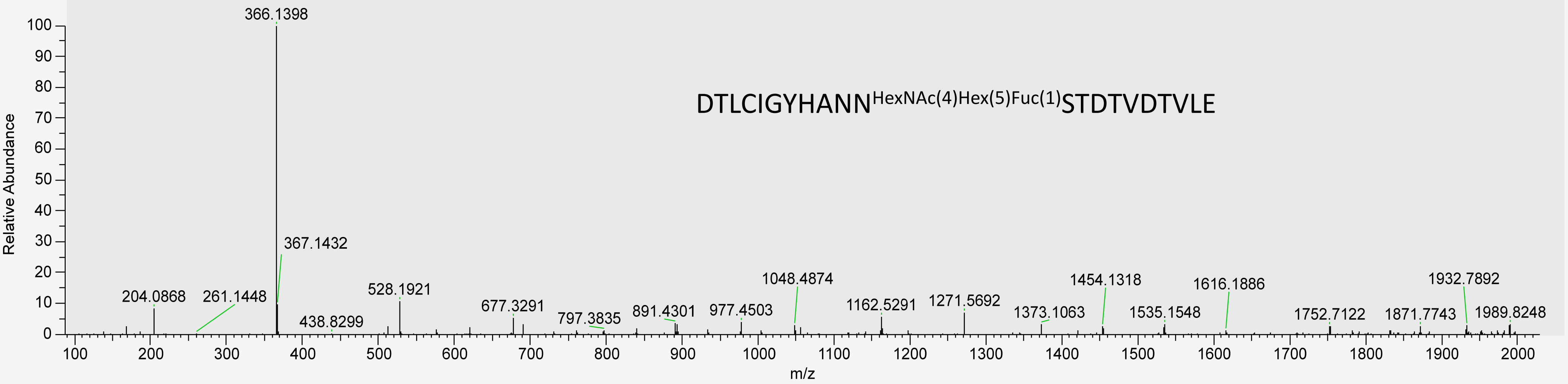

Lum_23oct2614 #15713 RT: 63.25 AV: 1 NL: 9.61E4
T: FTMS + c NSI d Full ms2 1467.2682@etd100.00 1467.2682@hcd25.00 [106.0000-2000.0000]

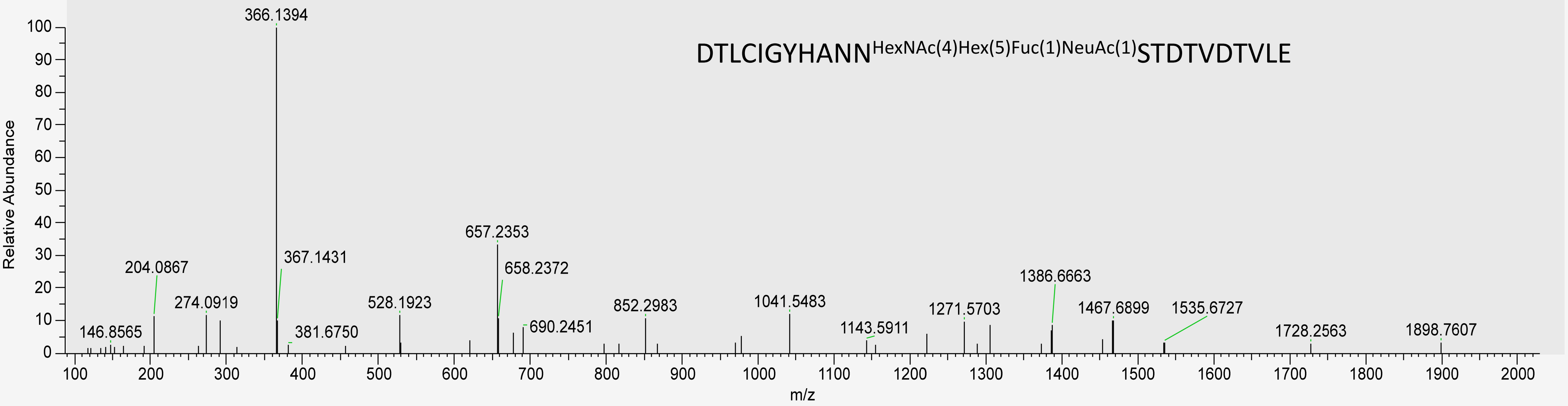

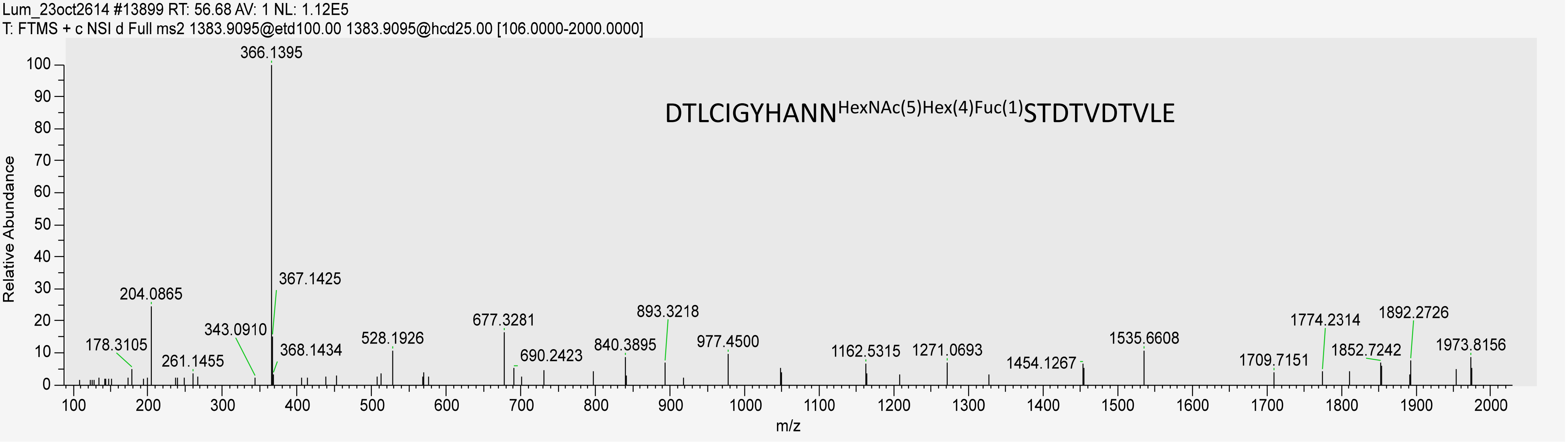
Lum_23oct2614 #13899 RT: 56.68 AV: 1 NL: 1.12E5
T: FTMS + c NSI d Full ms2 1383.9095@etd100.00 1383.9095@hcd25.00 [106.0000-2000.0000]
DTLCIGYHANN HexNAc(5)Hex(4)Fuc(1) STDTVDTVLE
366.1395
367.1425
204.0865
343.0910
178.3105
368.1434
261.1455
528.1926
677.3281
690.2423
893.3218
840.3895
977.4500
1162.5315
1271.0693
1454.1267
1535.6608
1709.7151
1774.2314
1852.7242
1892.2726
1973.8156
Relative Abundance
m/z

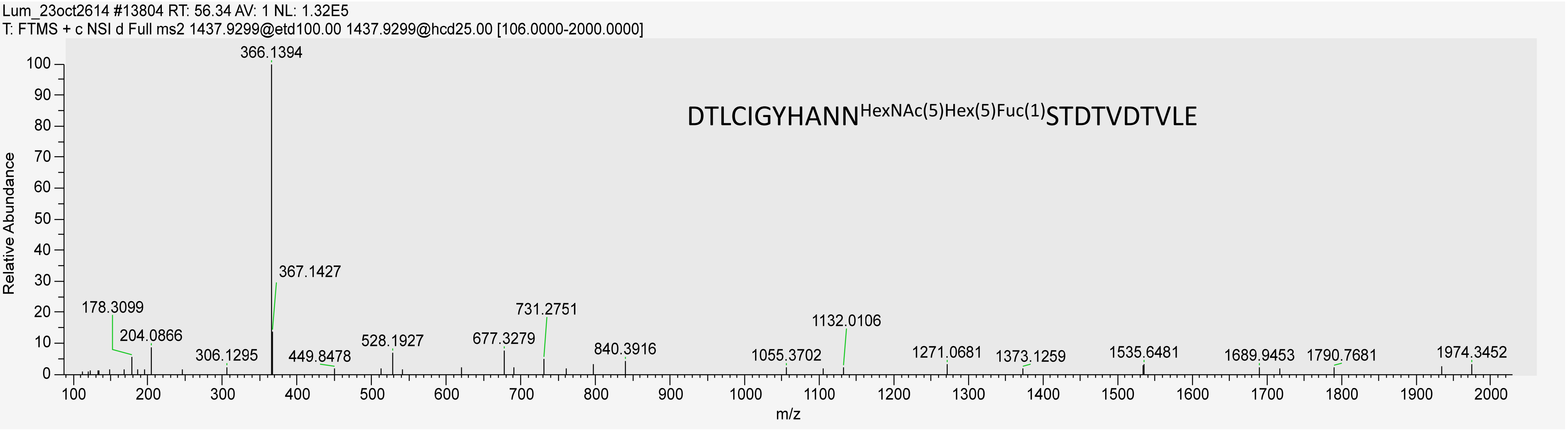
Lum_23oct2614 #13804 RT: 56.34 AV: 1 NL: 1.32E5
T: FTMS + c NSI d Full ms2 1437.9299@etd100.00 1437.9299@hcd25.00 [106.0000-2000.0000]
DTLCIGYHANN HexNAc(5)Hex(5)Fuc(1) STDTVDTVLE
366.1394
367.1427
178.3099
204.0866
306.1295
449.8478
528.1927
677.3279
731.2751
840.3916
1055.3702
1132.0106
1271.0681
1373.1259
1535.6481
1689.9453
1790.7681
1974.3452
Relative Abundance
m/z

Lum_23oct2614 #13665 RT: 55.83 AV: 1 NL: 3.89E5
T: FTMS + c NSI d Full ms2 1491.6141@etd100.00 1491.6141@hcd25.00 [106.0000-2000.0000]

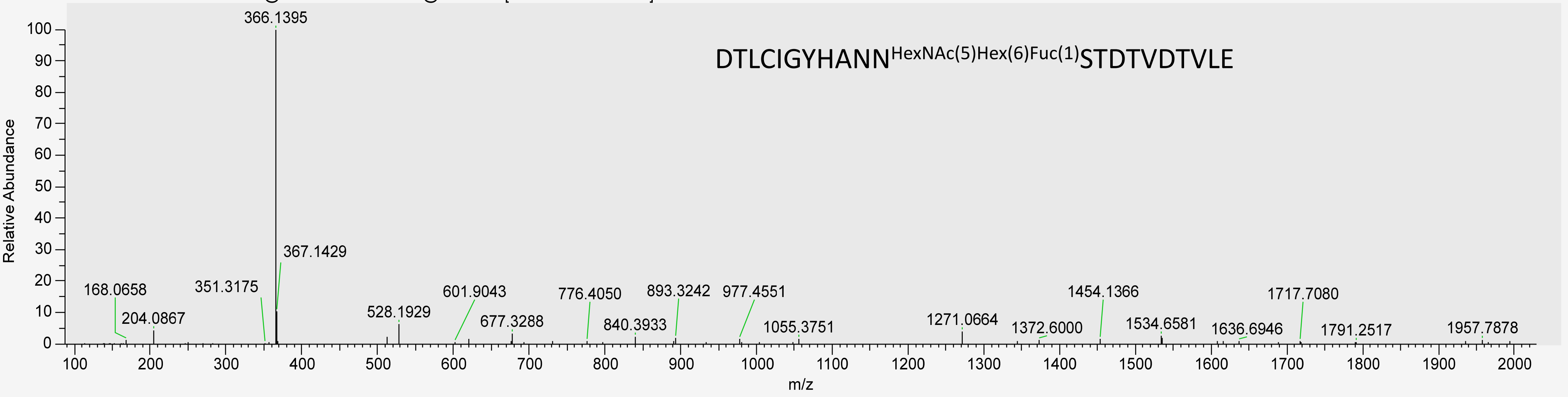

Lum_23oct2614 #13901 RT: 56.69 AV: 1 NL: 2.70E4
T: FTMS + c NSI d Full ms2 1397.5852@etd100.00 1397.5852@hcd25.00 [106.0000-2000.0000]

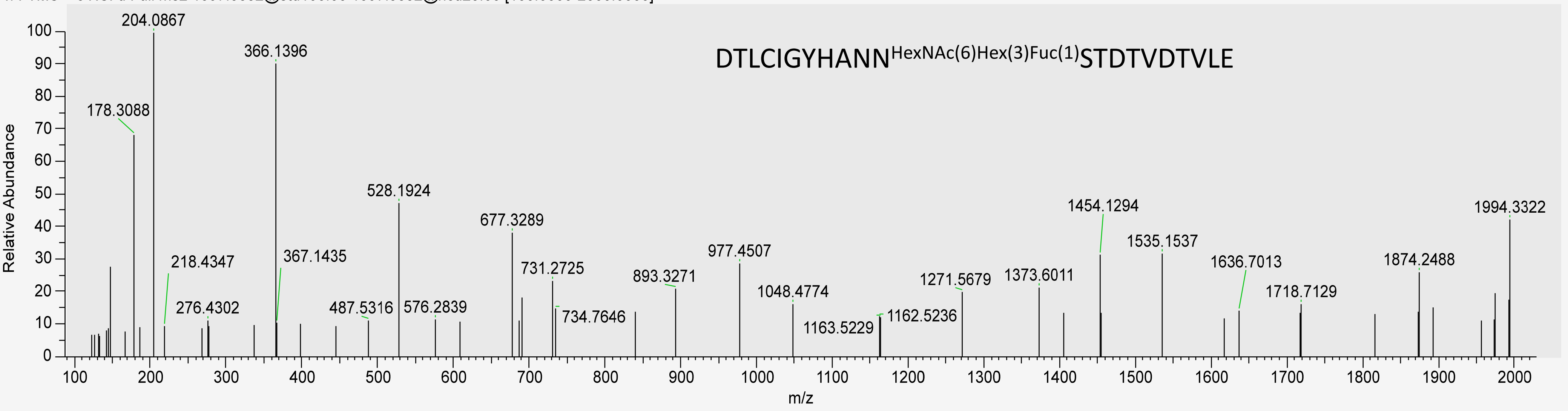

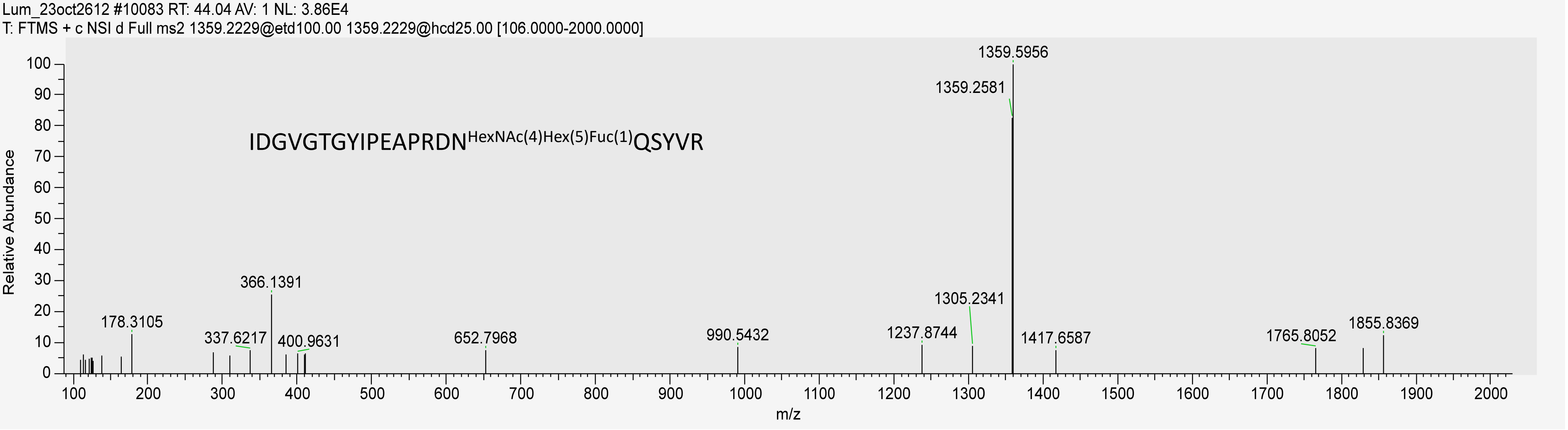
Lum_23oct2612 #10083 RT: 44.04 AV: 1 NL: 3.86E4
T: FTMS + c NSI d Full ms2 1359.2229@etd100.00 1359.2229@hcd25.00 [106.0000-2000.0000]
IDGVGTGYIPEAPRDN HexNAc(4)Hex(5)Fuc(1) QSYVR
Relative Abundance
m/z
1359.5956
1359.2581
366.1391
1305.2341
178.3105
337.6217
400.9631
652.7968
990.5432
1237.8744
1417.6587
1765.8052
1855.8369

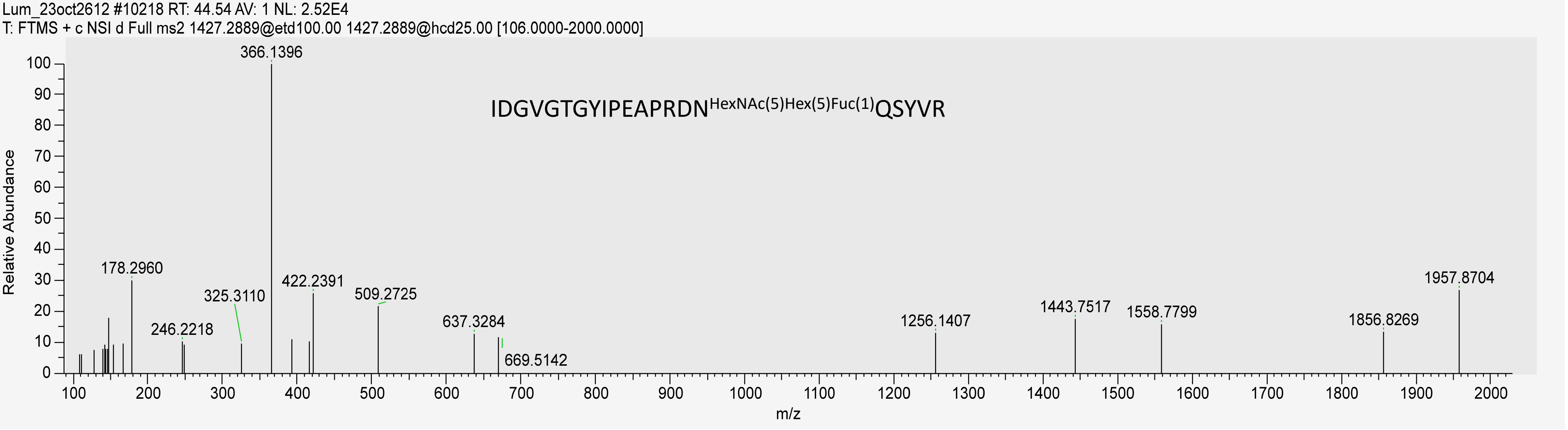
Lum_23oct2612 #10218 RT: 44.54 AV: 1 NL: 2.52E4
T: FTMS + c NSI d Full ms2 1427.2889@etd100.00 1427.2889@hcd25.00 [106.0000-2000.0000]
IDGVGTGYIPEAPRDN HexNAc(5)Hex(5)Fuc(1) QSYVR
Relative Abundance
m/z
366.1396
178.2960
422.2391
325.3110
509.2725
246.2218
637.3284
669.5142
1256.1407
1443.7517
1558.7799
1856.8269
1957.8704

Lum_23oct2612 #10053 RT: 43.93 AV: 1 NL: 1.74E5
T: FTMS + c NSI d Full ms2 1603.3539@etd100.00 1603.3539@hcd25.00 [106.0000-2000.0000]

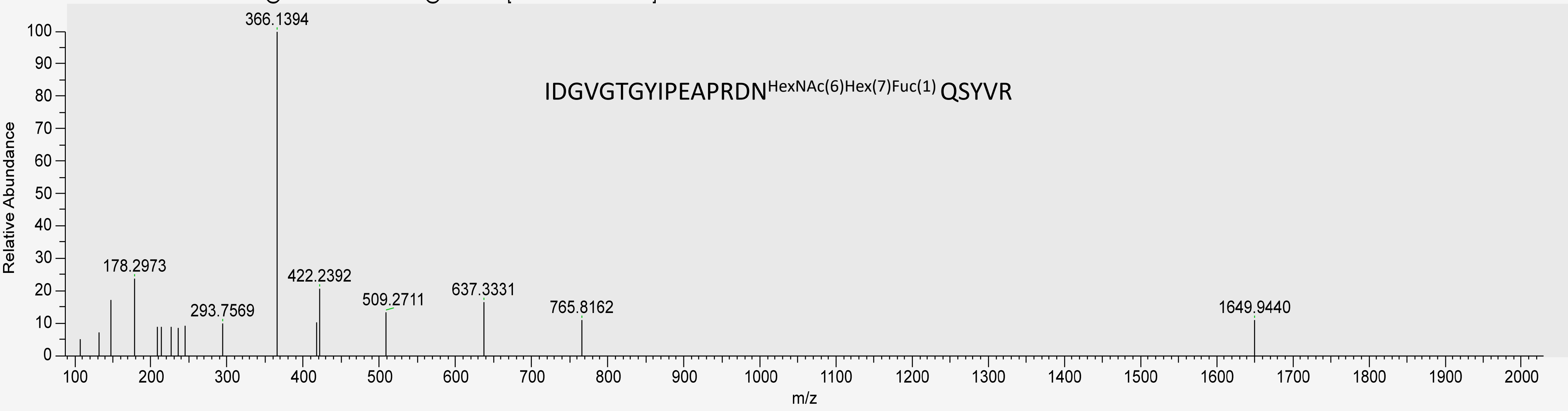

Lum_23oct2612 #11364 RT: 48.71 AV: 1 NL: 5.30E4
T: FTMS + c NSI d Full ms2 1412.5984@etd100.00 1412.5984@hcd25.00 [106.0000-2000.0000]

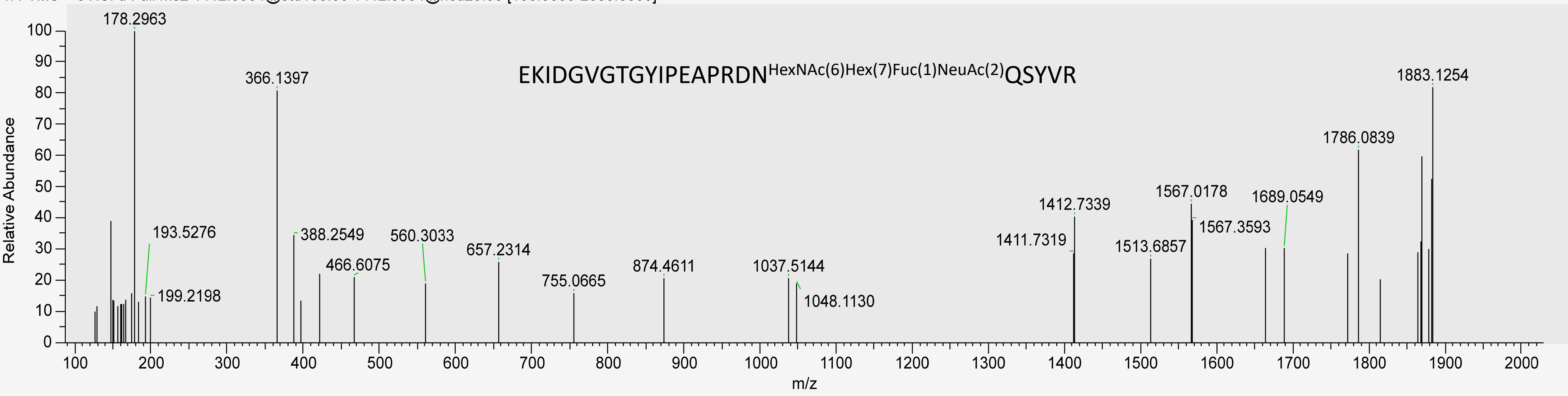

Lum_23oct2612 #9114 RT: 40.55 AV: 1 NL: 5.26E4
T: FTMS + c NSI d Full ms2 1358.3346@etd100.00 1358.3346@hcd25.00 [106.0000-2000.0000]

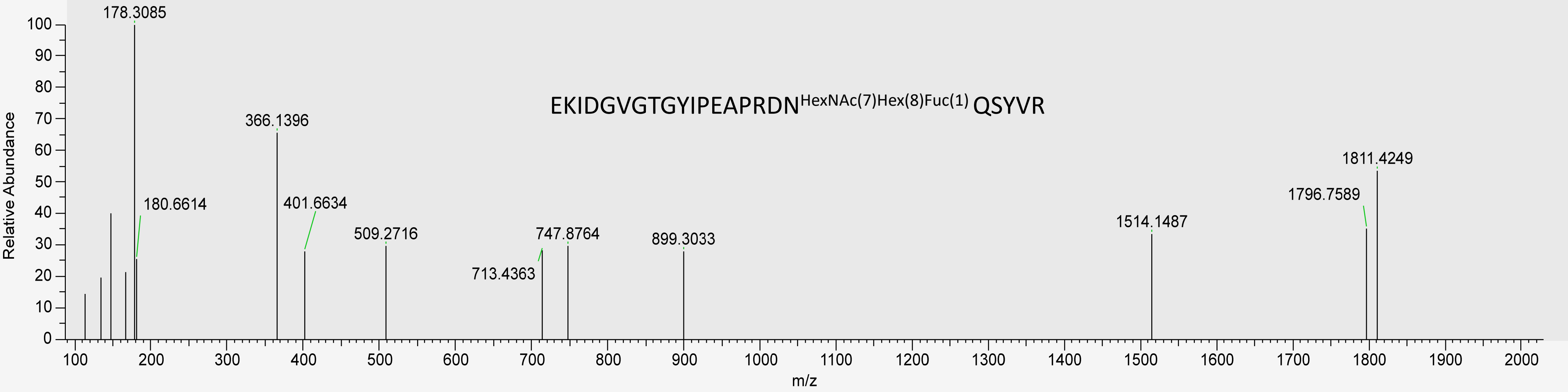

Lum_23oct2614 #7368 RT: 33.33 AV: 1 NL: 9.51E5
T: FTMS + c NSI d Full ms2 1007.4605@etd100.00 1007.4605@hcd25.00 [106.0000-2000.0000]

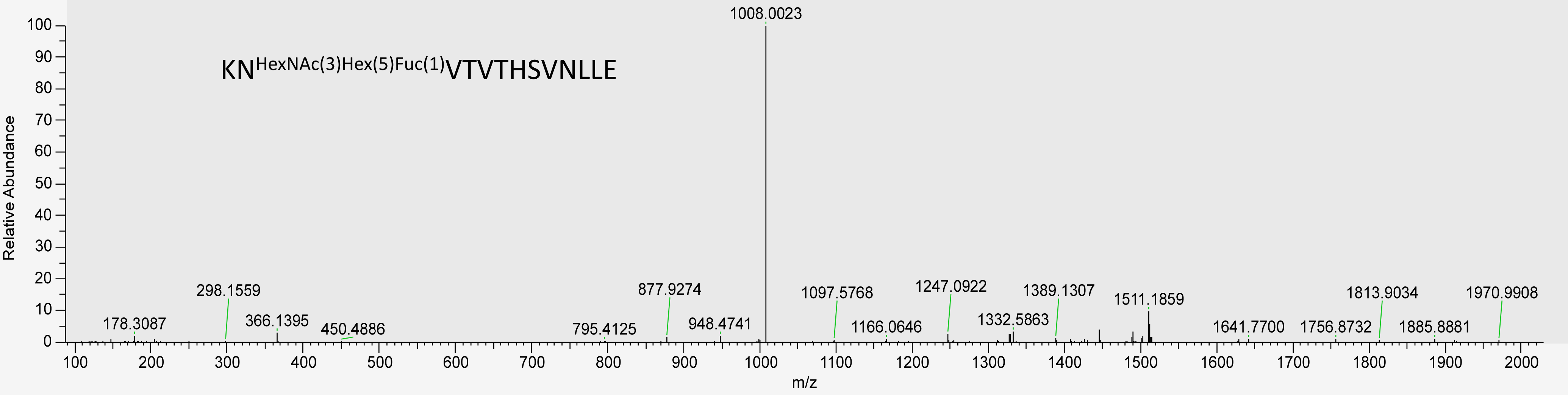

Lum_23oct2614 #8818 RT: 38.54 AV: 1 NL: 5.30E4
T: FTMS + c NSI d Full ms2 1158.8420@etd100.00 1158.8420@hcd25.00 [106.0000-2000.0000]

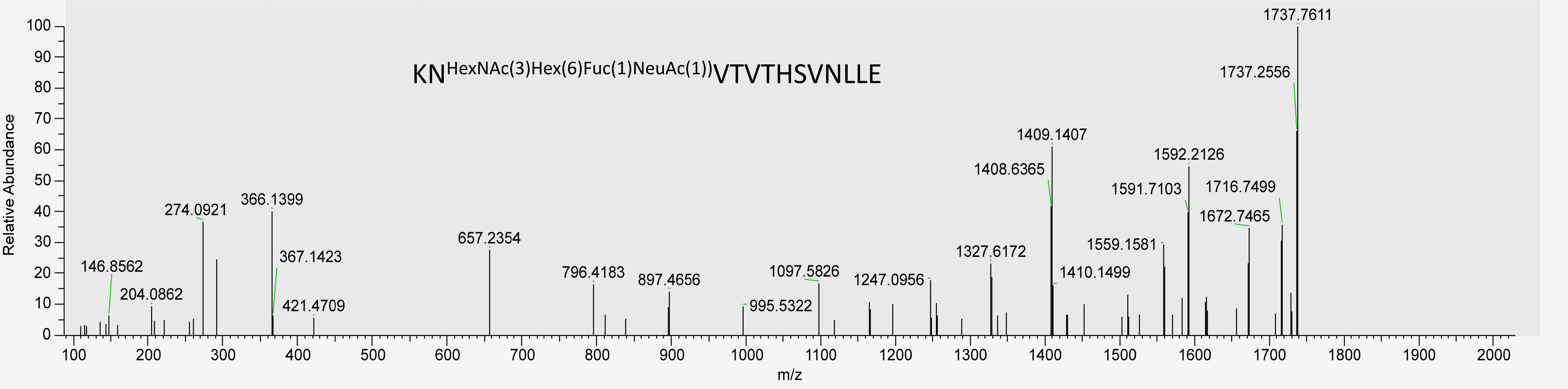

Lum_23oct2614 #7124 RT: 32.46 AV: 1 NL: 1.09E5
T: FTMS + c NSI d Full ms2 1129.1703@etd100.00 1129.1703@hcd25.00 [106.0000-2000.0000]

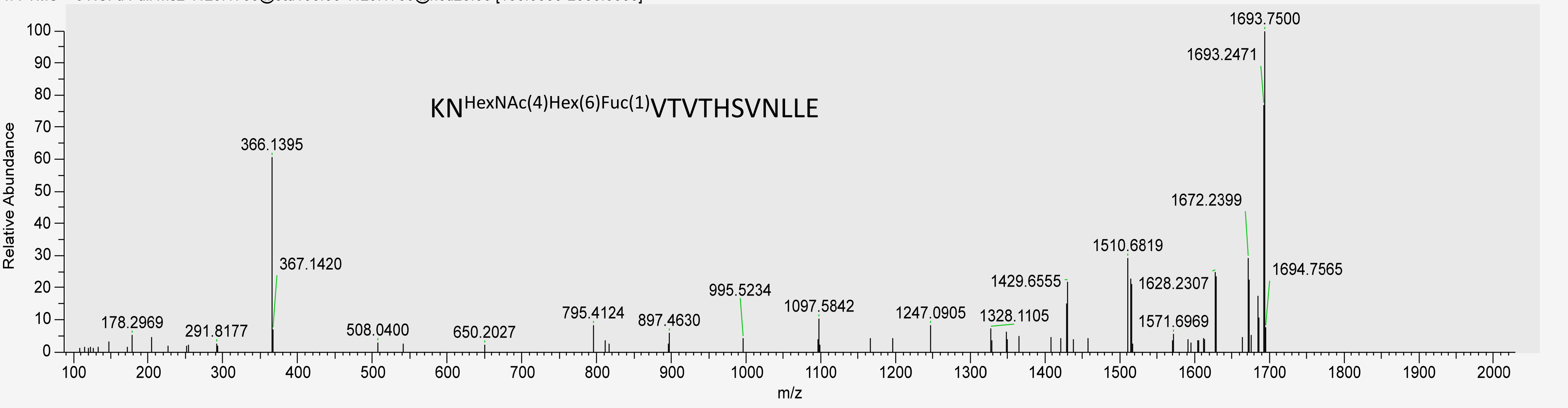

Lum_23oct2614 #7162 RT: 32.60 AV: 1 NL: 1.02E5
T: FTMS + c NSI d Full ms2 1040.1423@etd100.00 1040.1423@hcd25.00 [106.0000-2000.0000]

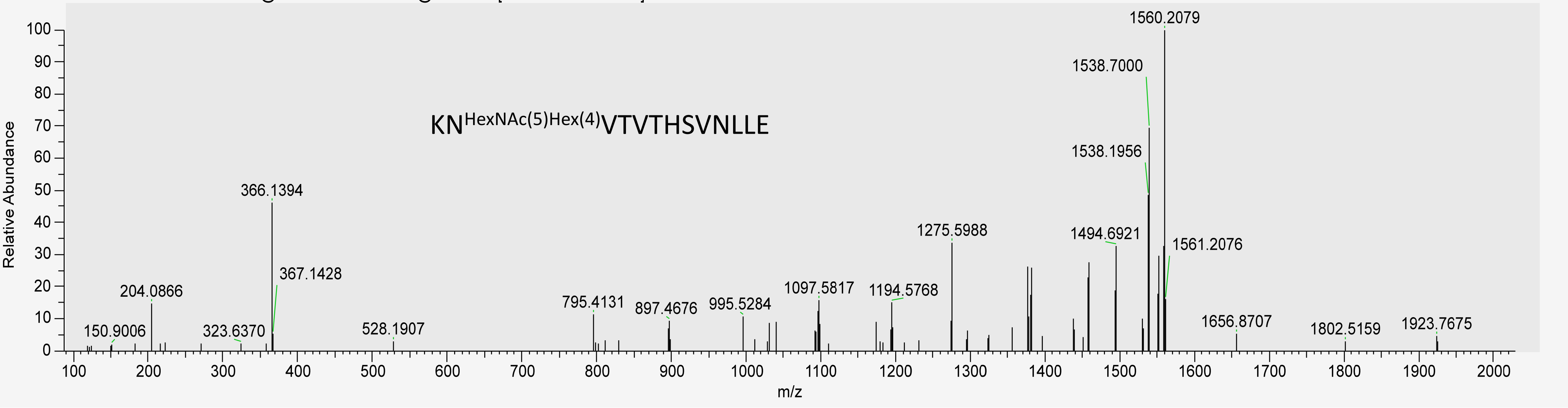

Lum_23oct2614 #10186 RT: 43.50 AV: 1 NL: 9.12E4
T: FTMS + c NSI d Full ms2 1391.5956@etd100.00 1391.5956@hcd25.00 [106.0000-2000.0000]

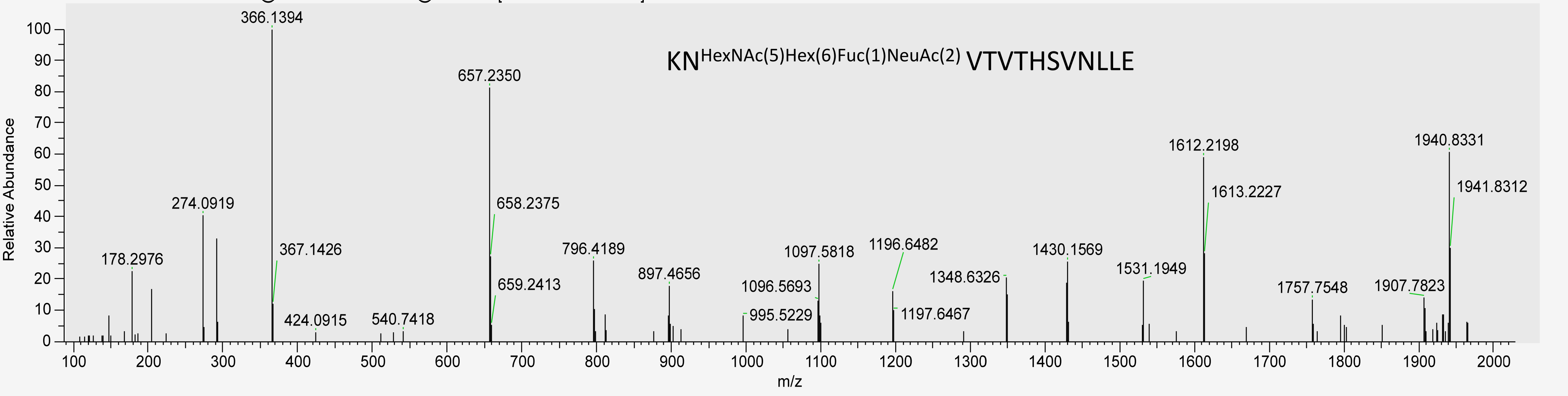

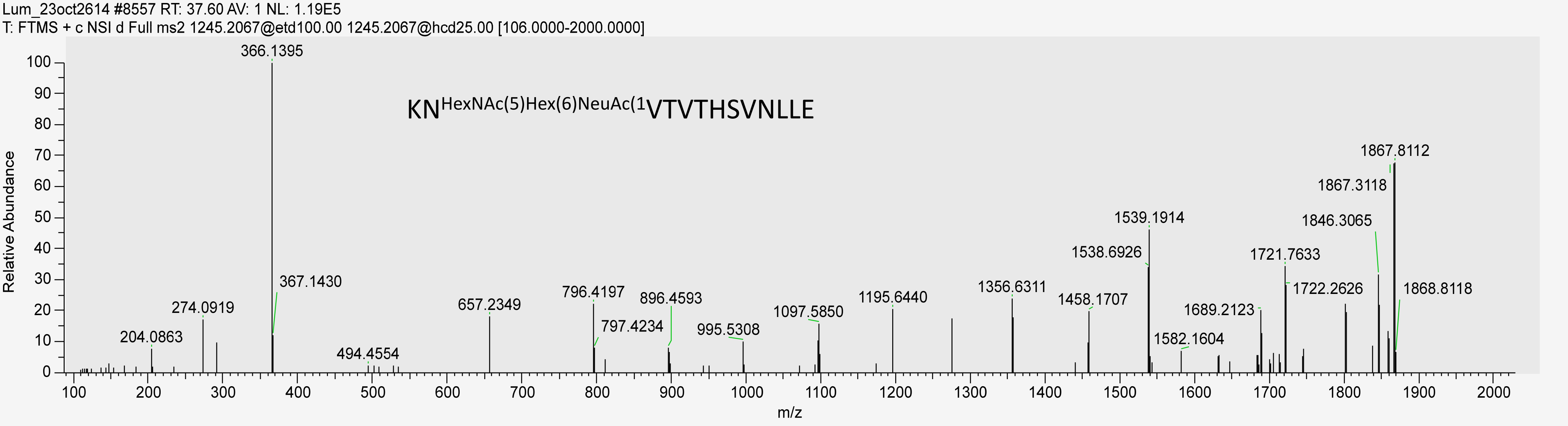
Lum_23oct2614 #8557 RT: 37.60 AV: 1 NL: 1.19E5
T: FTMS + c NSI d Full ms2 1245.2067@etd100.00 1245.2067@hcd25.00 [106.0000-2000.0000]
KN HexNAc(5)Hex(6)NeuAc(1 VTVTHSVNLLE
Relative Abundance
m/z
366.1395
367.1430
274.0919
204.0863
494.4554
657.2349
796.4197
797.4234
896.4593
995.5308
1097.5850
1195.6440
1356.6311
1458.1707
1538.6926
1539.1914
1582.1604
1689.2123
1721.7633
1722.2626
1846.3065
1867.3118
1867.8112
1868.8118

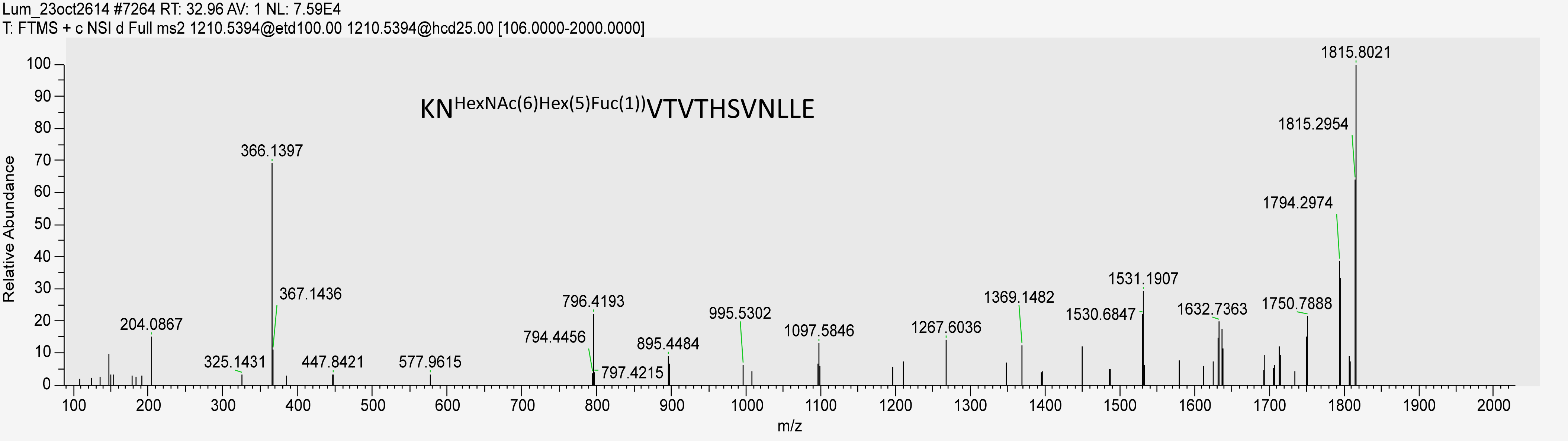
Lum_23oct2614 #7264 RT: 32.96 AV: 1 NL: 7.59E4
T: FTMS + c NSI d Full ms2 1210.5394@etd100.00 1210.5394@hcd25.00 [106.0000-2000.0000]
KN HexNAc(6)Hex(5)Fuc(1)) VTVTHSVNLLE
Relative Abundance
m/z
366.1397
367.1436
204.0867
325.1431
447.8421
577.9615
794.4456
796.4193
797.4215
895.4484
995.5302
1097.5846
1267.6036
1369.1482
1530.6847
1531.1907
1632.7363
1750.7888
1794.2974
1815.2954
1815.8021

Lum_23oct2614 #8605 RT: 37.77 AV: 1 NL: 1.89E5
T: FTMS + c NSI d Full ms2 1361.9208@etd100.00 1361.9208@hcd25.00 [106.0000-2000.0000]

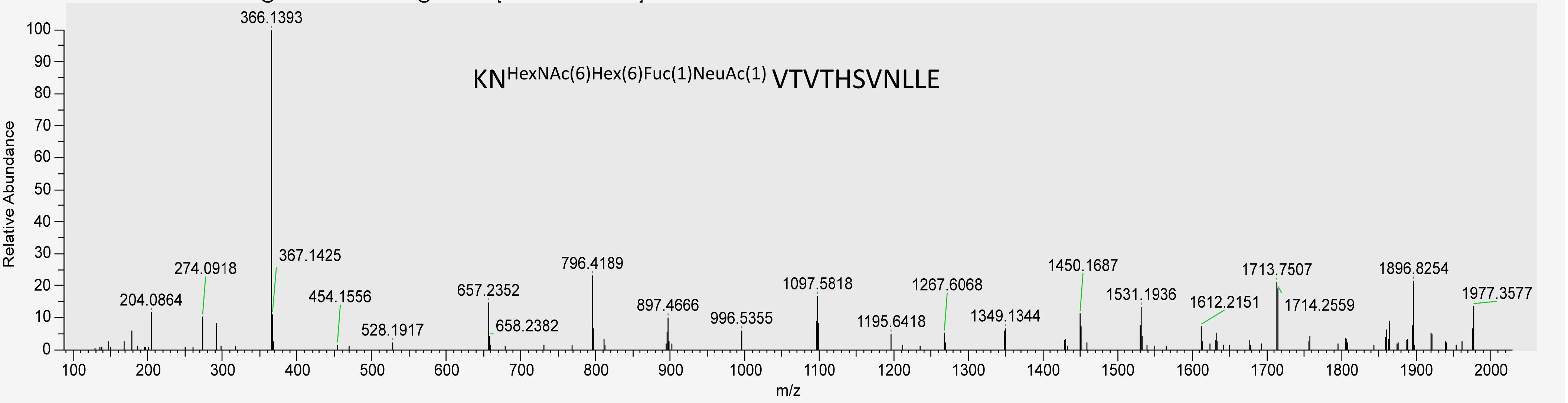

Lum_23oct2614 #7005 RT: 32.04 AV: 1 NL: 4.34E5
T: FTMS + c NSI d Full ms2 1270.2223@etd100.00 1270.2223@hcd25.00 [106.0000-2000.0000]

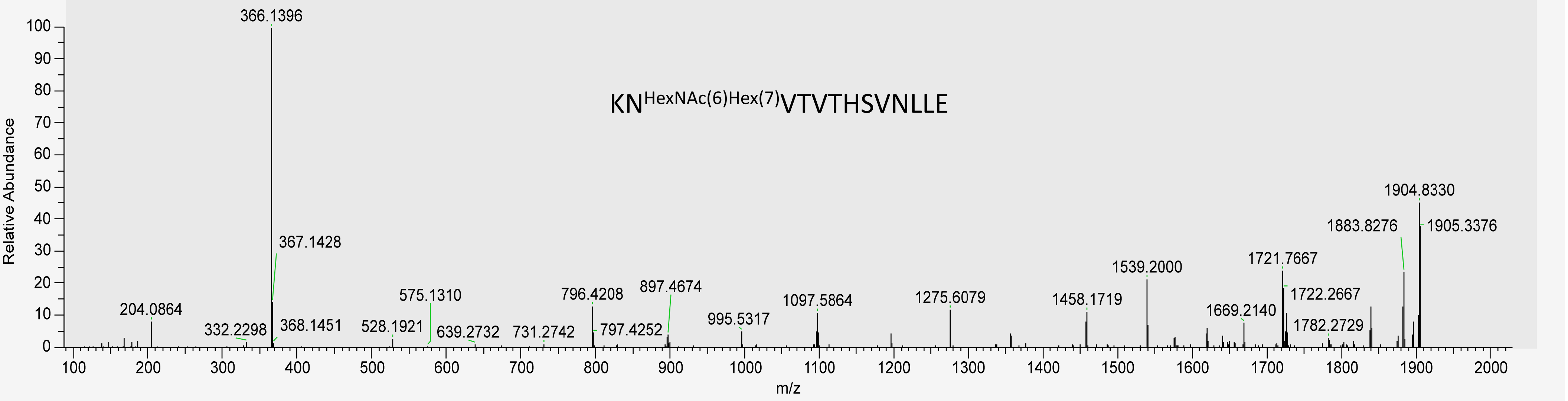

Lum_23oct2614 #8696 RT: 38.10 AV: 1 NL: 5.81E4
T: FTMS + c NSI d Full ms2 1348.5775@etd100.00 1348.5775@hcd25.00 [106.0000-2000.0000]

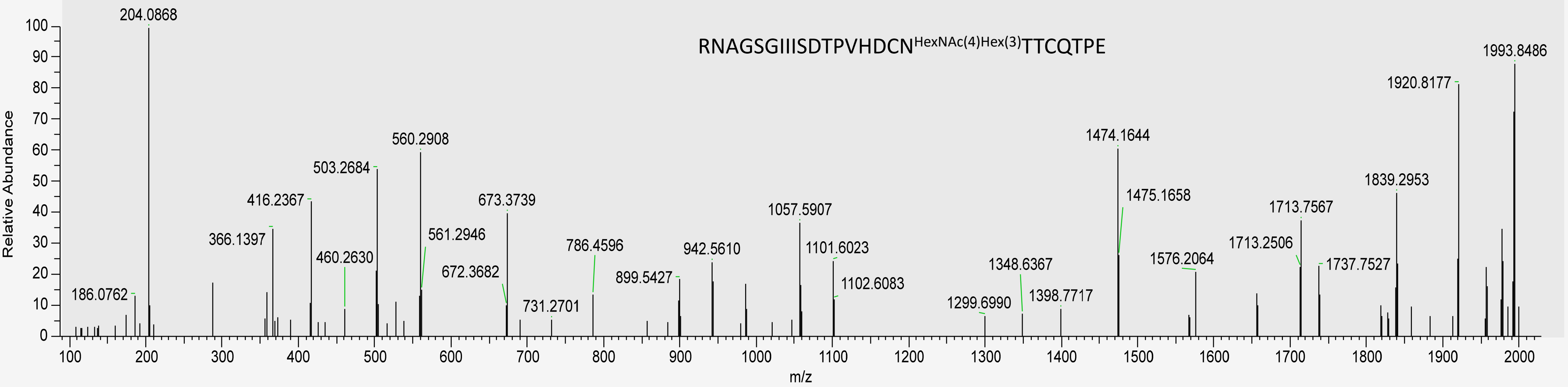

Lum_23oct2614 #8628 RT: 37.85 AV: 1 NL: 6.78E4
T: FTMS + c NSI d Full ms2 1402.5955@etd100.00 1402.5955@hcd25.00 [106.0000-2000.0000]

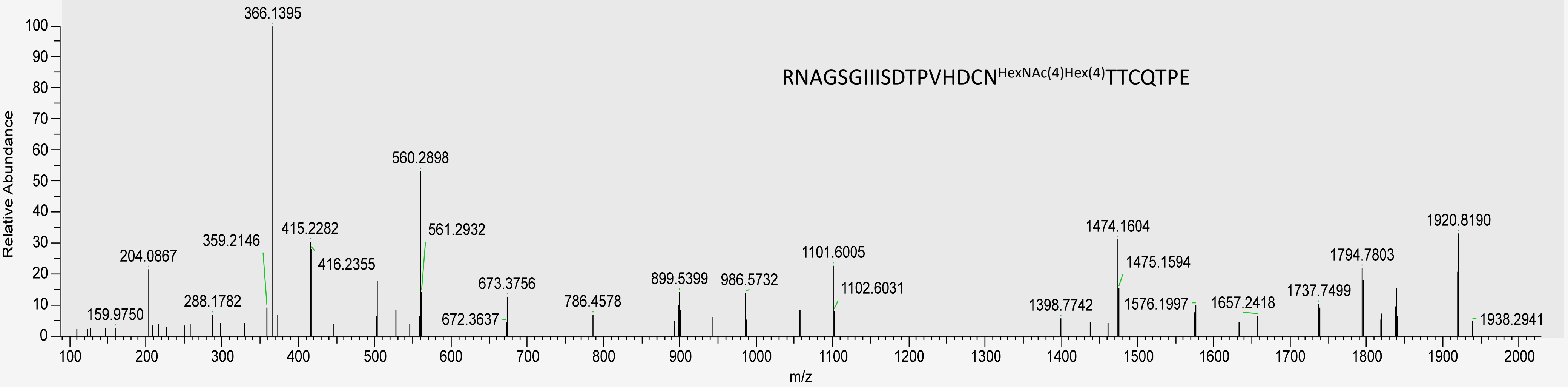

Lum_23oct2614 #9866 RT: 42.34 AV: 1 NL: 1.62E4
T: FTMS + c NSI d Full ms2 1499.6260@etd100.00 1499.6260@hcd25.00 [106.0000-2000.0000]

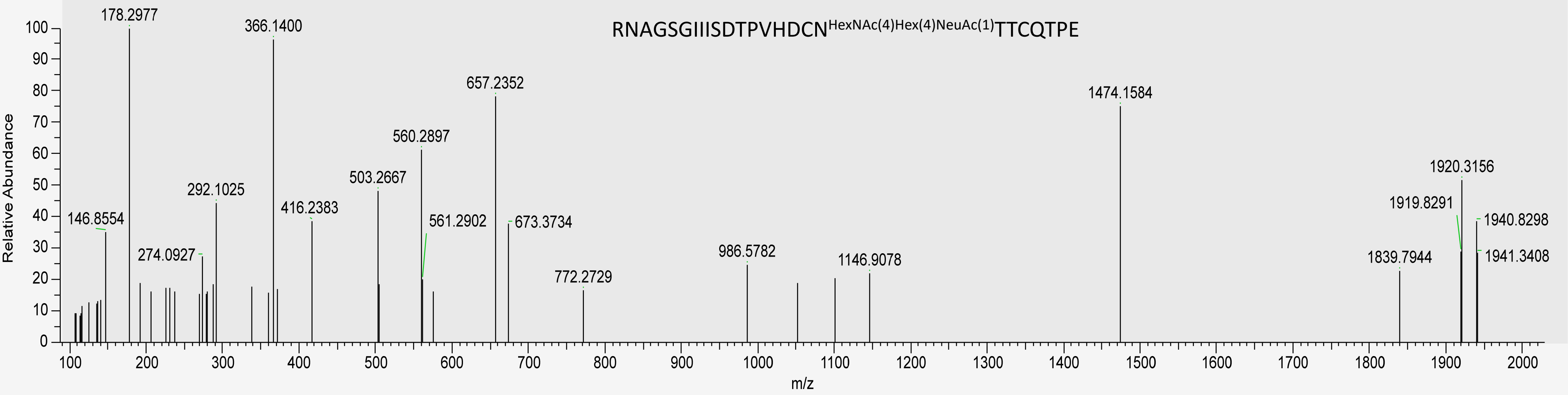

Lum_23oct2614 #9610 RT: 41.41 AV: 1 NL: 3.44E5
T: FTMS + c NSI d Full ms2 1293.5353@etd100.00 1293.5353@hcd25.00 [106.0000-2000.0000]

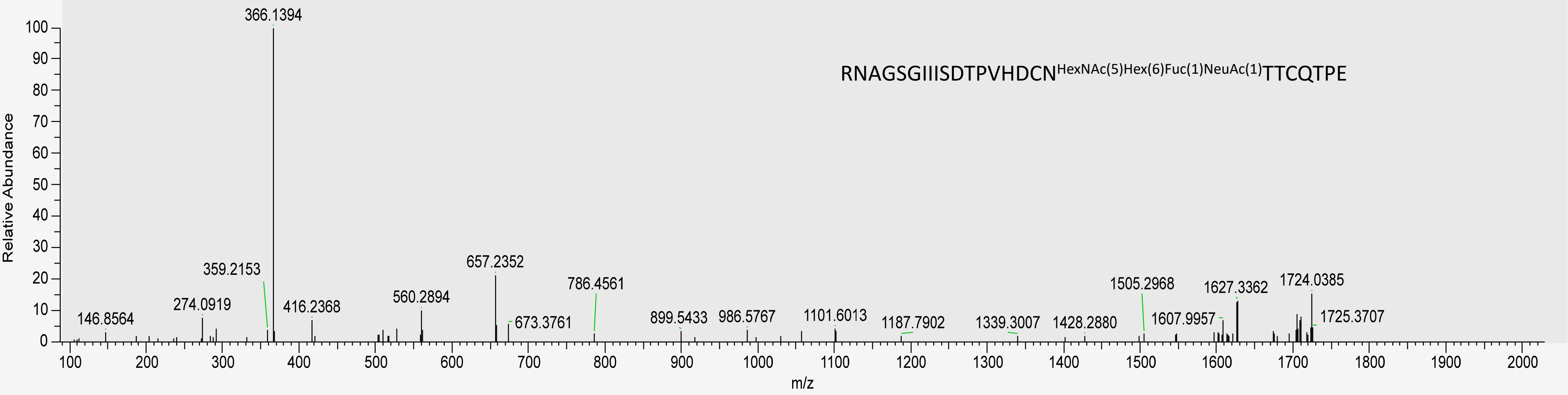

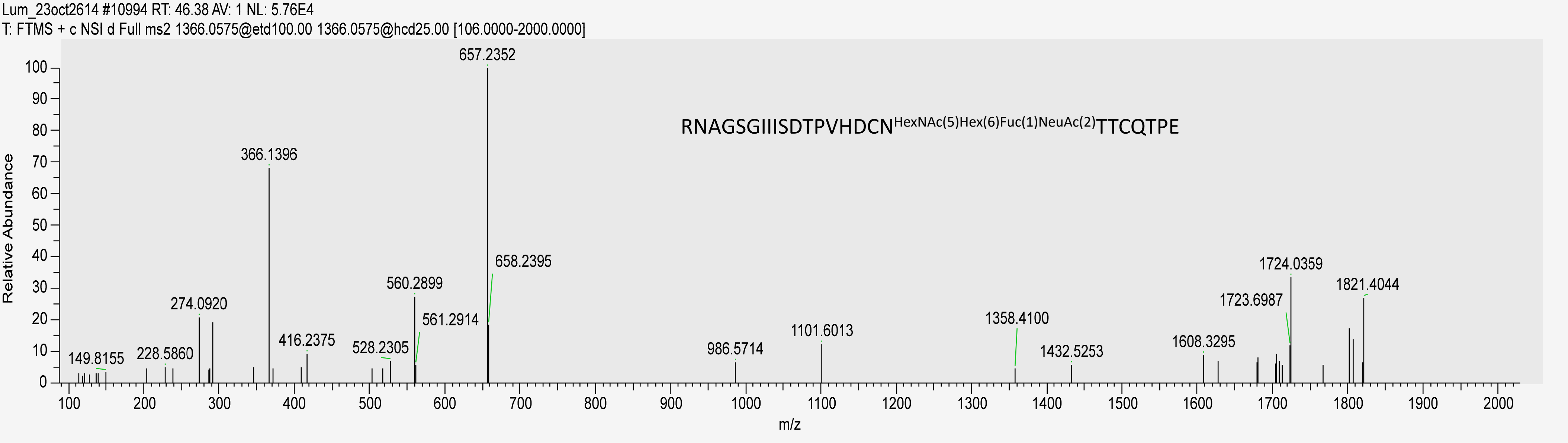
Lum_23oct2614 #10994 RT: 46.38 AV: 1 NL: 5.76E4
T: FTMS + c NSI d Full ms2 1366.0575@etd100.00 1366.0575@hcd25.00 [106.0000-2000.0000]
RNAGSGIIISDTPVHDCN HexNAc(5)Hex(6)Fuc(1)NeuAc(2) TTCQTPE
Relative Abundance
m/z
657.2352
366.1396
658.2395
560.2899
561.2914
274.0920
416.2375
528.2305
228.5860
149.8155
986.5714
1101.6013
1358.4100
1432.5253
1608.3295
1723.6987
1724.0359
1821.4044

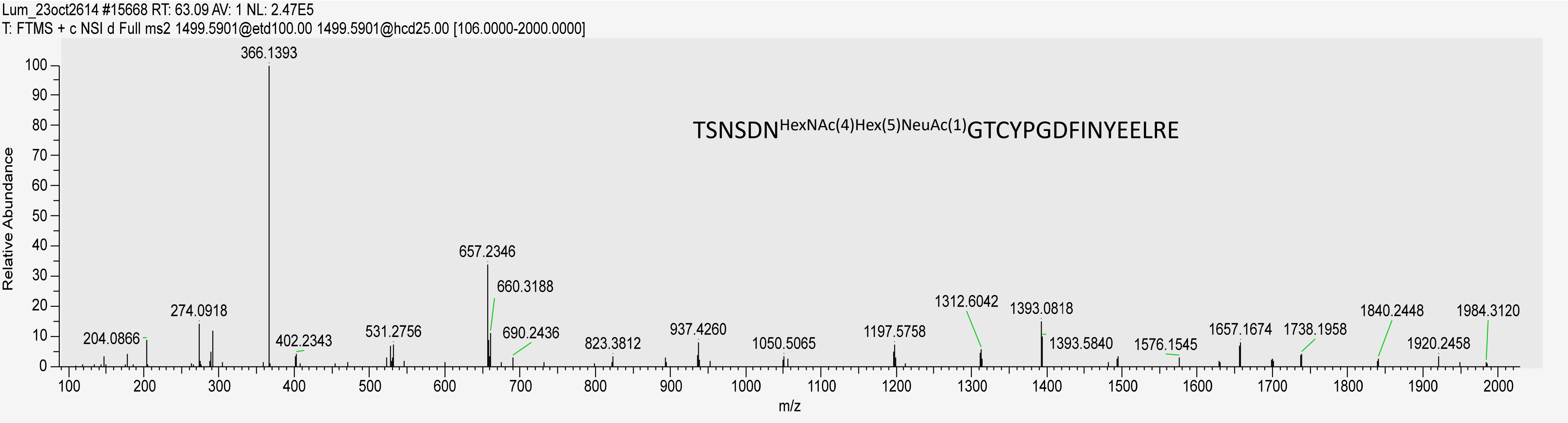
Lum_23oct2614 #15668 RT: 63.09 AV: 1 NL: 2.47E5
T: FTMS + c NSI d Full ms2 1499.5901@etd100.00 1499.5901@hcd25.00 [106.0000-2000.0000]
TSNSDN HexNAc(4)Hex(5)NeuAc(1) GTCYPGDFINYEELRE
Relative Abundance
m/z
366.1393
657.2346
660.3188
274.0918
204.0866
402.2343
531.2756
690.2436
823.3812
937.4260
1050.5065
1197.5758
1312.6042
1393.0818
1393.5840
1576.1545
1657.1674
1738.1958
1840.2448
1920.2458
1984.3120

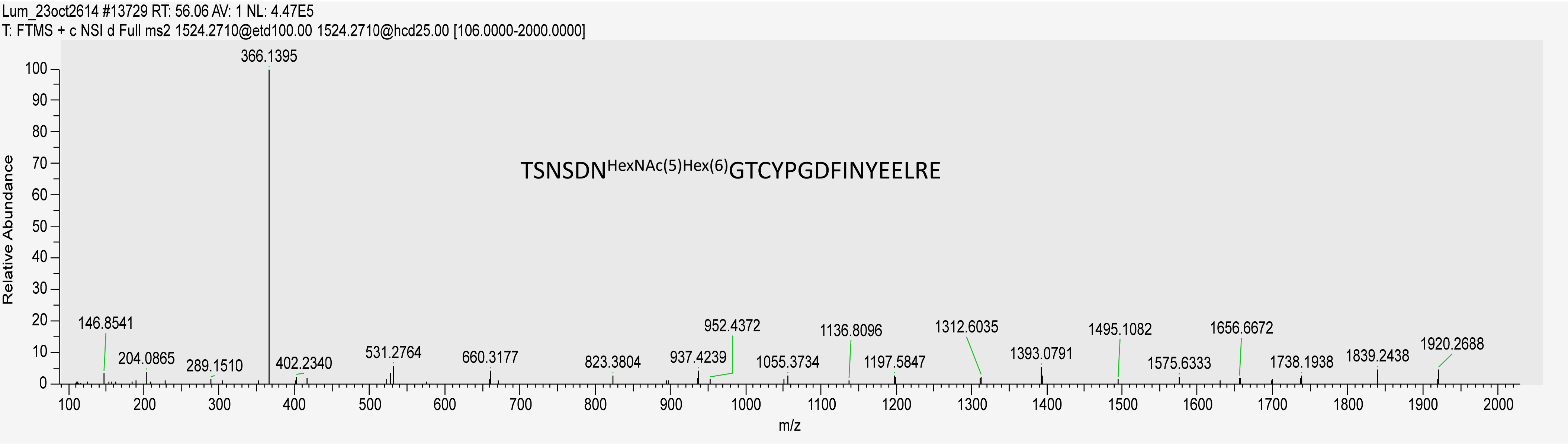
Lum_23oct2614 #13729 RT: 56.06 AV: 1 NL: 4.47E5
T: FTMS + c NSI d Full ms2 1524.2710@etd100.00 1524.2710@hcd25.00 [106.0000-2000.0000]
TSNSDN HexNAc(5)Hex(6) GTCYPGDFINYEELRE
Relative Abundance
m/z
366.1395
146.8541
204.0865
289.1510
402.2340
531.2764
660.3177
823.3804
937.4239
952.4372
1055.3734
1136.8096
1197.5847
1312.6035
1393.0791
1495.1082
1575.6333
1656.6672
1738.1938
1839.2438
1920.2688

Y2-CHO-WT trypsin

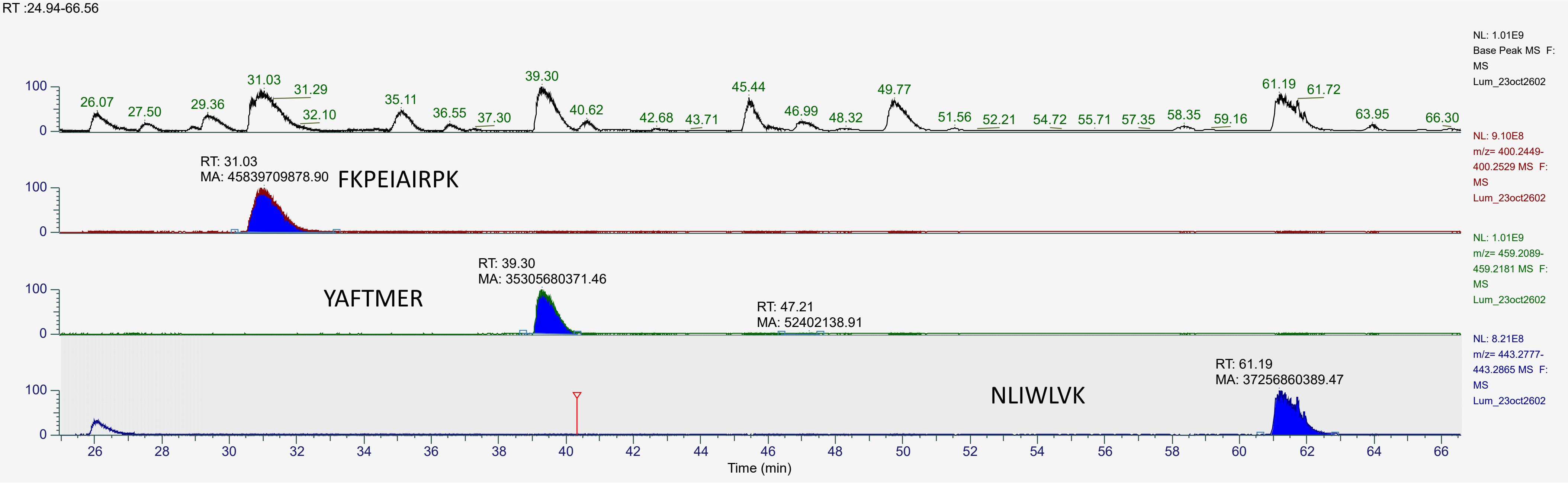

RT (Min)	Base Peak	Peak Area	Peak Height	Baseline Width	SignalToNoise
31.03317	400.2489	4.58E+10	898319579.7	3.018301	INF
39.29645	459.2137	3.53E+10	985123605	1.635085	INF
47.21206	459.2131	52402139	969768.1361	1.157015	INF
61.19271	443.2822	3.73E+10	820931328	2.213653	INF

S1 Y2-CHO-S1 trypsin

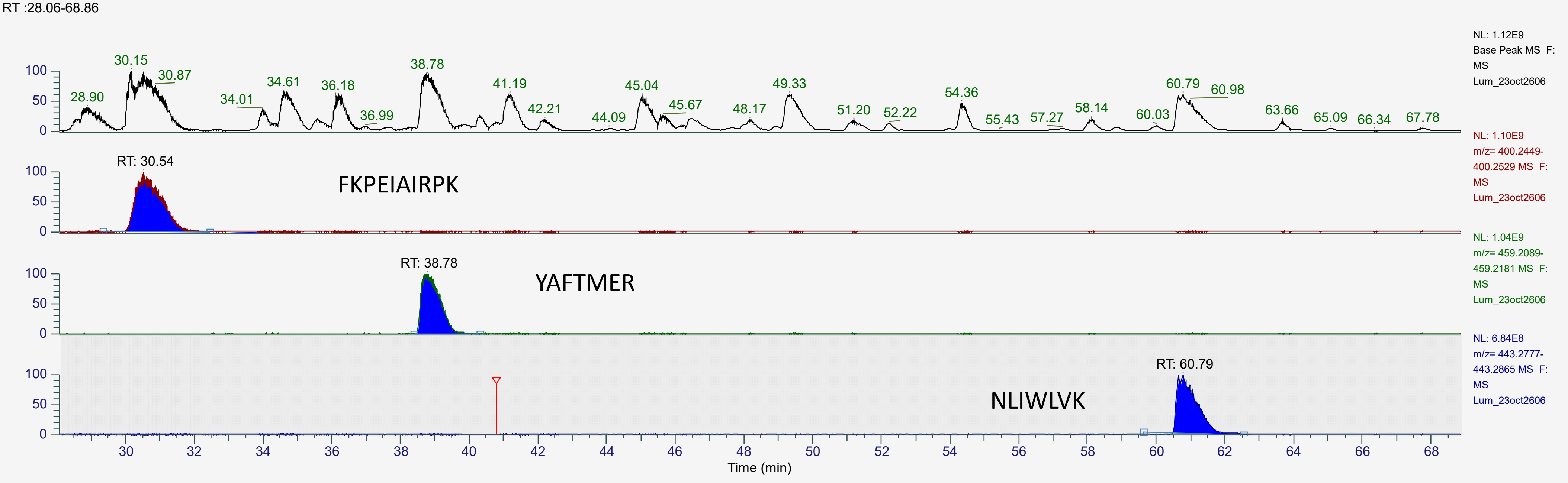

RT (Min)	Base Peak	Peak Area	Peak Height	Baseline Width	SignalToNoise
30.53836	400.2489	5.3E+10	1.09E+09	3.117292	INF
38.77926	459.2137	3.92E+10	1.04E+09	1.927778	INF
60.79105	443.282	2.57E+10	6.62E+08	2.934827	INF

Y2-CHO-S2 trypsin

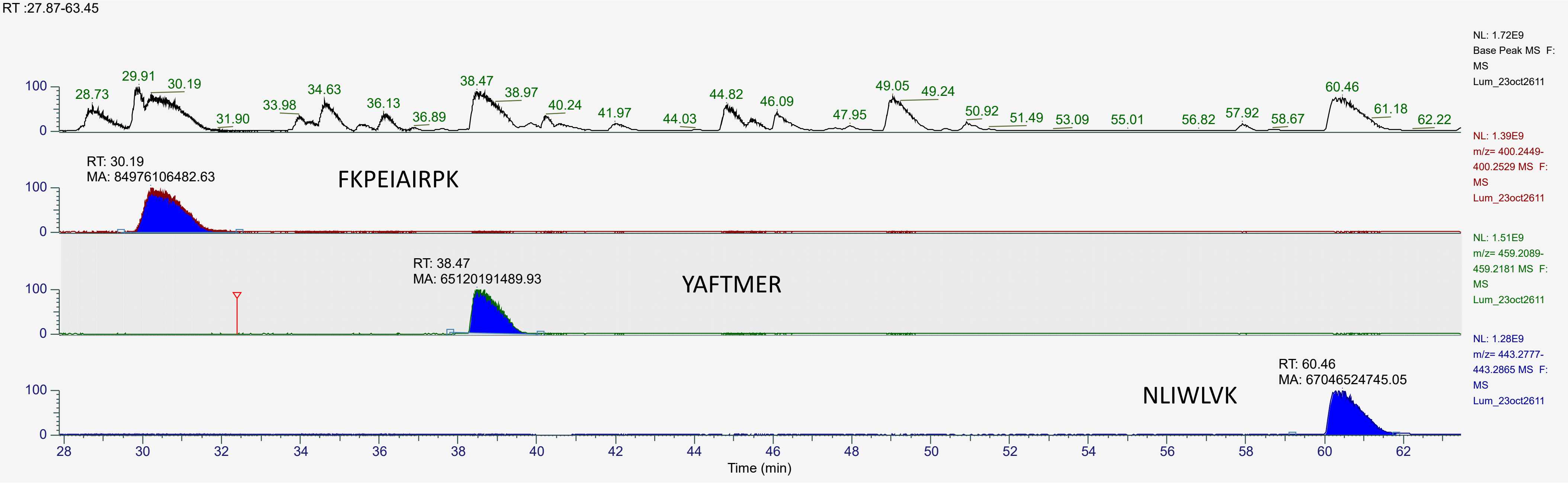

RT (Min)	Base Peak	Peak Area	Peak Height	Baseline Width	SignalToNoise
30.19423	400.2491	8.5E+10	1.39E+09	3.011001	INF
38.47419	459.2133	6.51E+10	1.45E+09	2.283343	INF
60.45907	443.282	6.7E+10	1.28E+09	2.634282	INF

Y2-CHO-S3 trypsin

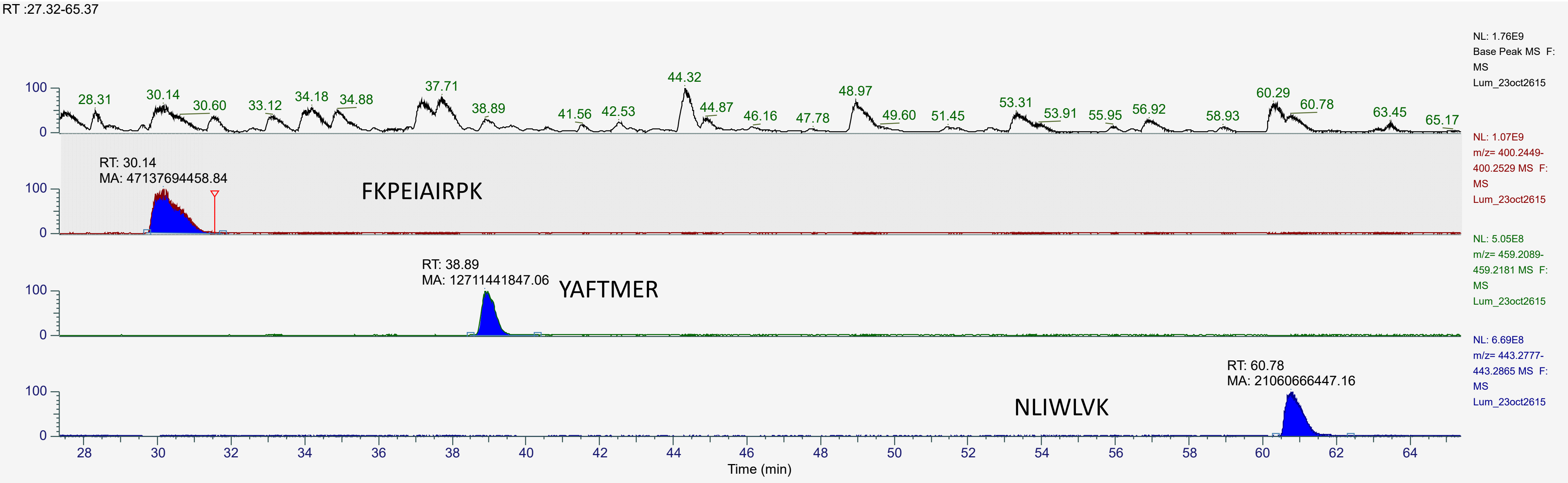

RT (Min)	Base Peak	Peak Area	Peak Height	Baseline Width	SignalToNoise
30.14409	400.249	47137694459	1049484174	2.066185	INF
38.89395	459.2133	12711441847	505220288	1.824723	INF
60.78156	443.2821	21060666447	669484352	2.012562	INF

Y2-CHO-WT trypsin

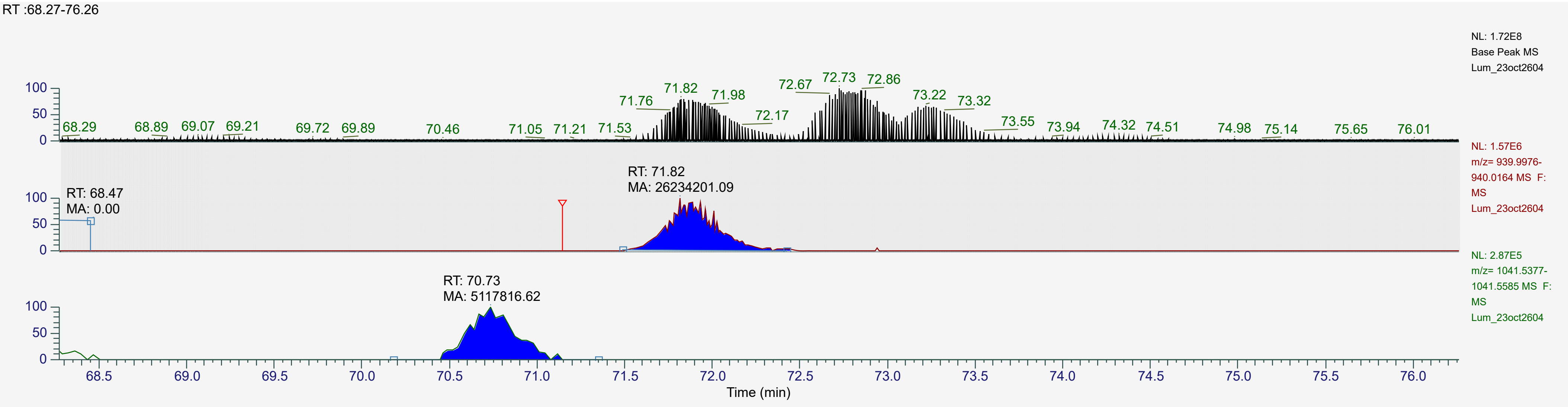

RT (Min)	Base Peak	Peak Area	Peak Height	Baseline Width	SignalToNoise
71.81591	940.0066	26234201	1544070	0.937612	INF
70.73479	1041.547	5117817	287203.5	1.168254	INF

Y2-CHO-S1 trypsin

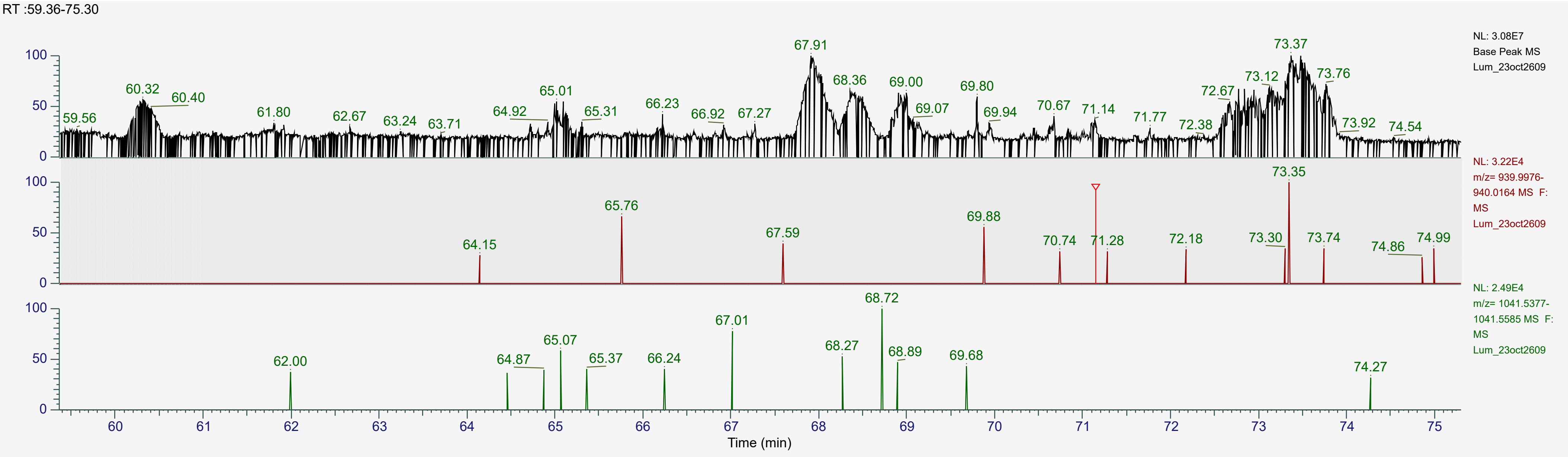

Y2-CHO-S2 trypsin

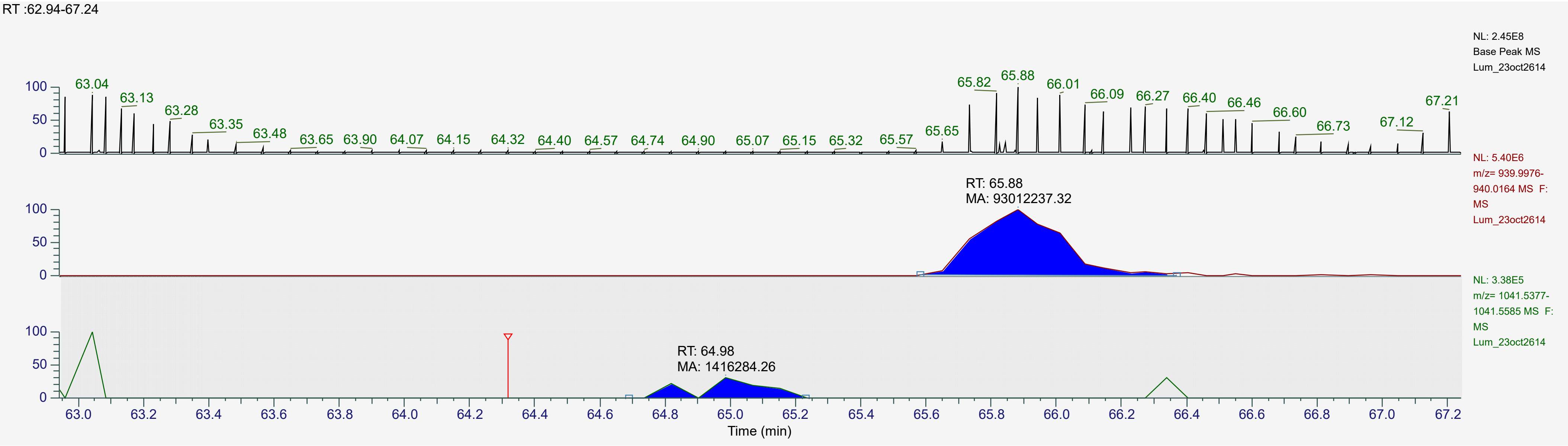

RT (Min)	Base Peak	Peak Area	Peak Height	Baseline Width	SignalToNoise
65.88246	940.0063	93012237.32	5326632	0.78728	INF
64.98499	1041.547	1416284.258	103681.3	0.543187	INF

Y2-CHO-S3 trypsin

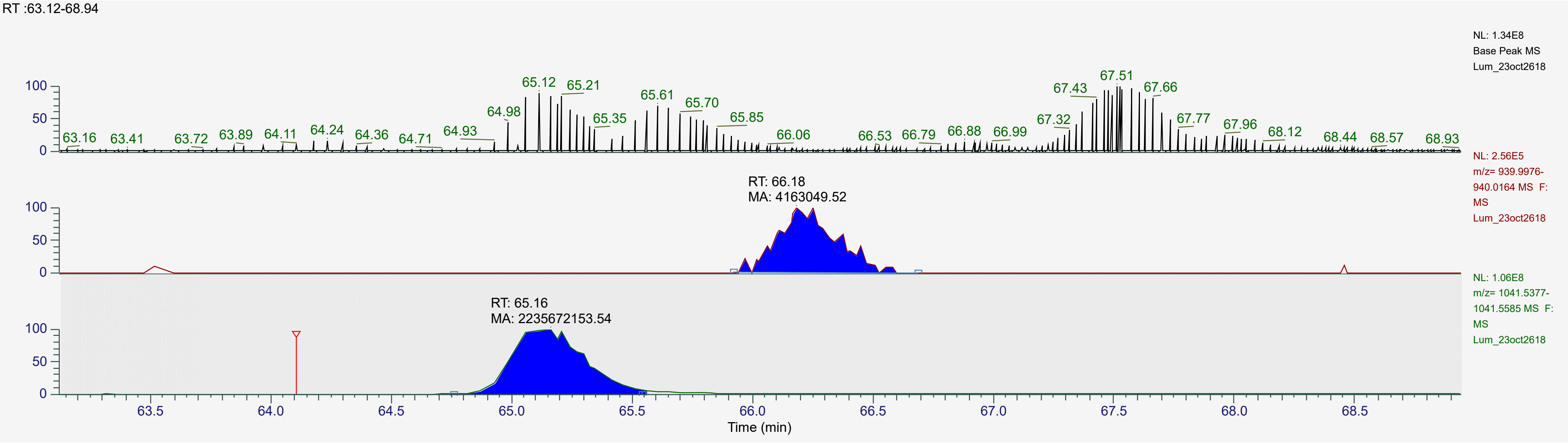

Y2-CHO-WT trypsin

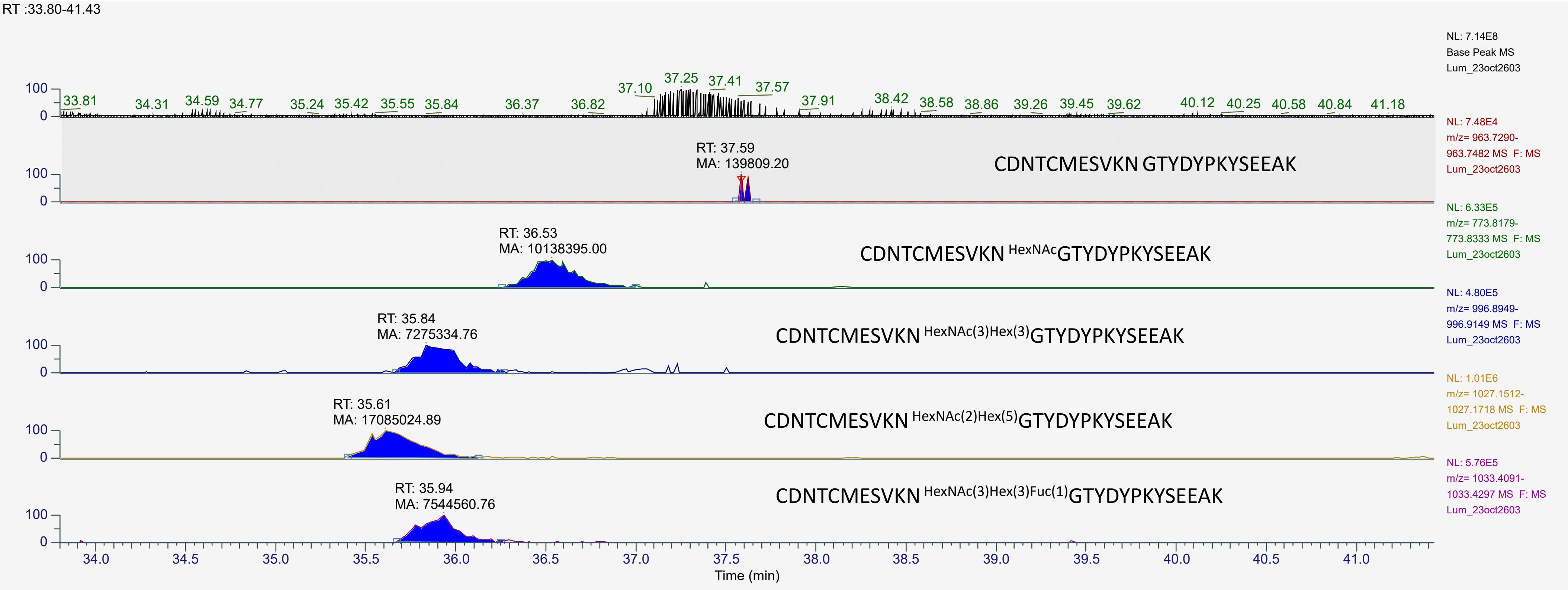

Index	RT (Min)	Base Peak	Peak Area	Peak Height	Baseline Width	SignalToNoise
1	37.58668	963.7386	139809.2	72489.78	0.116436	INF
1	36.5348	773.8262	10138395	633112.3	0.740198	INF
1	35.8379	996.9058	7275335	473849.9	0.603379	INF
1	35.61127	1027.162	17085025	974942.1	0.724054	INF
1	35.93622	1033.419	7544561	562365.5	0.57802	INF

Y2-CHO-S1 trypsin

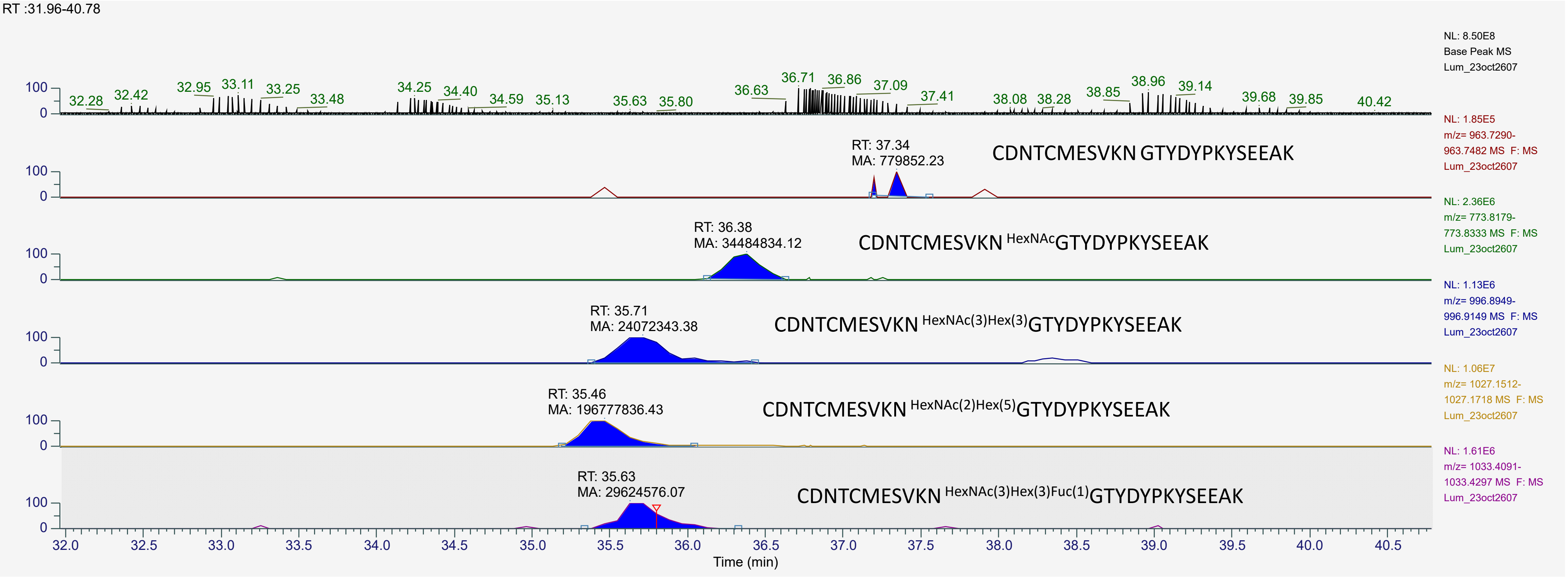

RT (Min)	Base Peak	Peak Area	Peak Height	Baseline Width	SignalToNoise
37.34258	963.7368	779852.2	175178.4	0.361782	INF
36.38083	773.8254	34484834	2306658	0.505248	INF
35.71389	996.905	24072343	1117683	1.054262	INF
35.46364	1027.161	1.97E+08	10564066	0.848558	INF
35.63084	1033.42	29624576	1608273	0.985545	INF

Y2-CHO-S2 trypsin

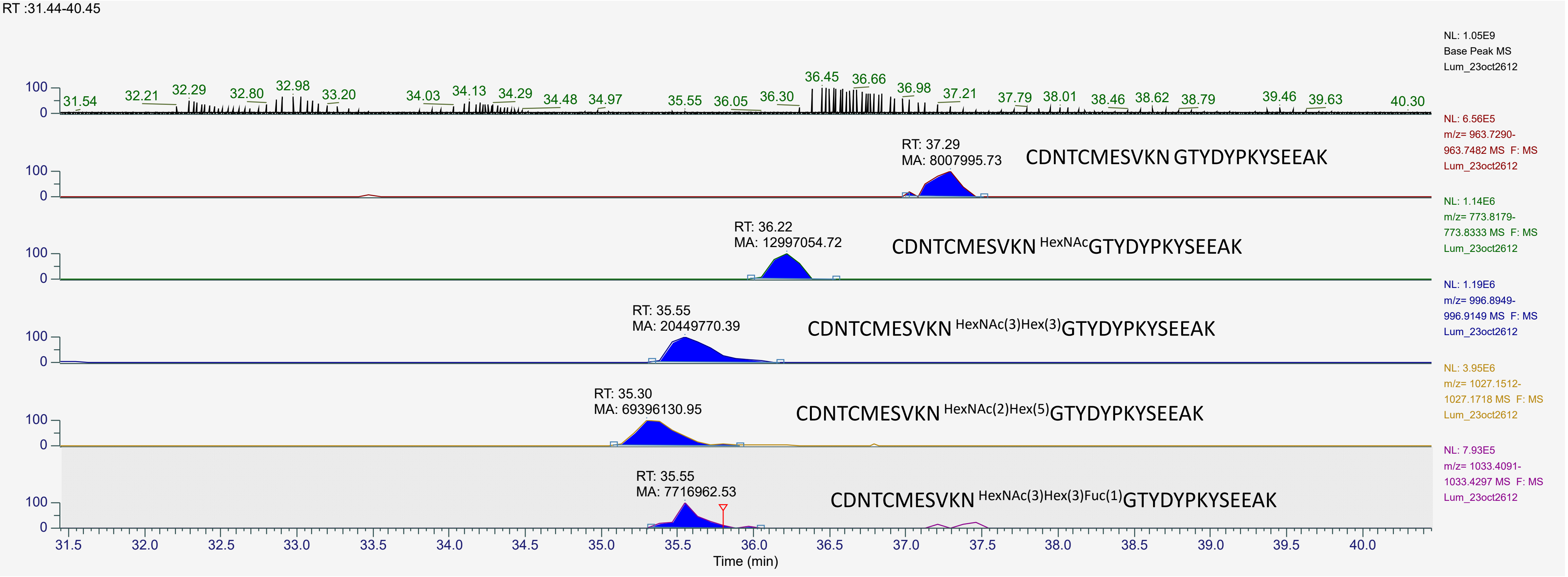

RT (Min)	Base Peak	Peak Area	Peak Height	Baseline Width	SignalToNoise
37.29244	963.7379	8007996	637611.5	0.516132	INF
36.21877	773.8254	12997055	1111261	0.56072	INF
35.55177	996.9049	20449770	1145116	0.847451	INF
35.30073	1027.161	69396131	3825112	0.828359	INF
35.55177	1033.419	7716963	756539.6	0.720035	INF

Y2-CHO-S3 trypsin

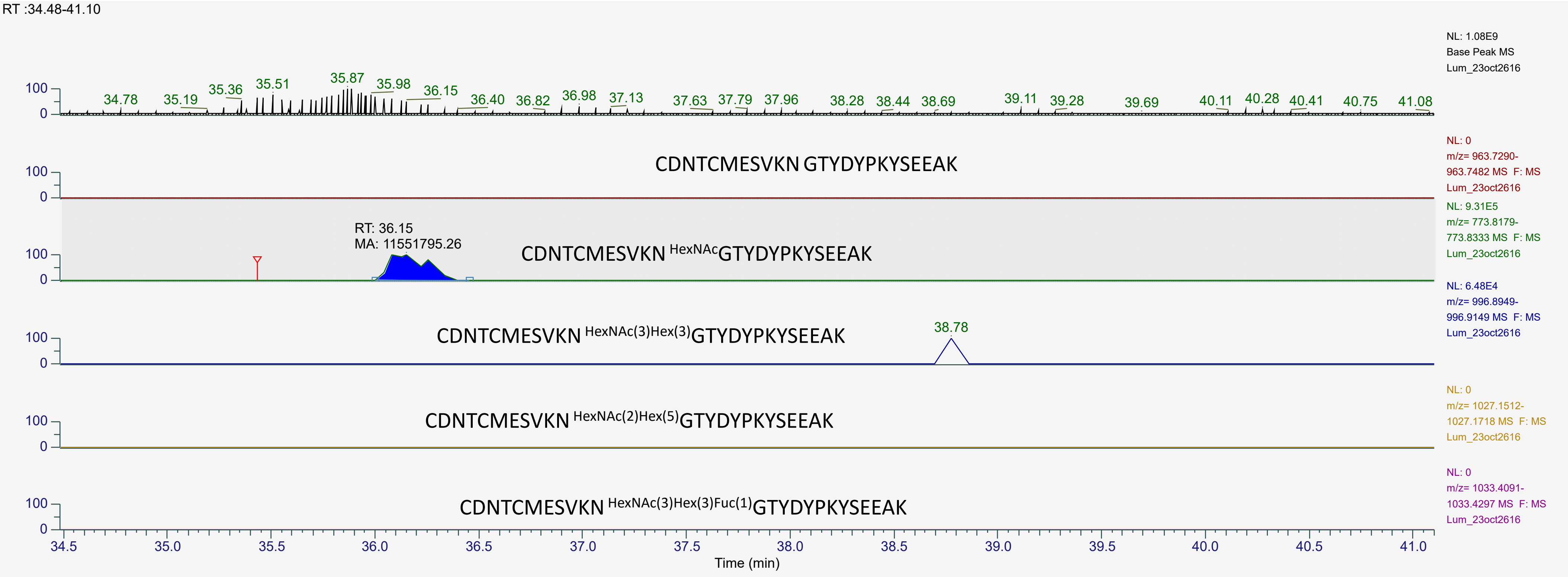

RT (Min)	Base Peak	Peak Area	Peak Height	Baseline Width	SignalToNoise
36.15056	773.8259	11551795	910520.6	0.449412	INF

Y2-CHO-WT trypsin

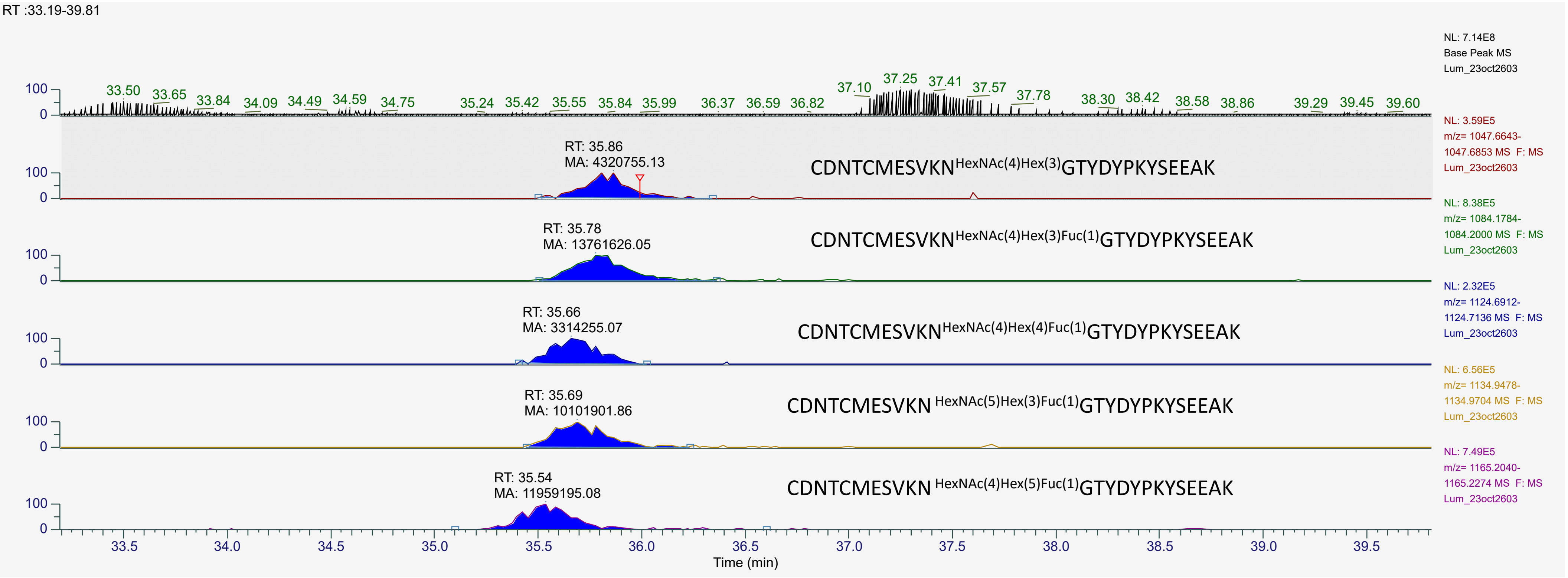

RT (Min)	Base Peak	Peak Area	Peak Height	Baseline Width	SignalToNoise
35.86234	1047.676	4320755	345885.7	0.842716	INF
35.77796	1084.19	13761626	825674.5	0.856761	INF
35.66126	1124.702	3314255	222924.6	0.622673	INF
35.68957	1134.96	10101902	641482.1	0.791154	INF
35.53767	1165.216	11959195	749004.4	1.502725	INF

Y2-CHO-S1 trypsin

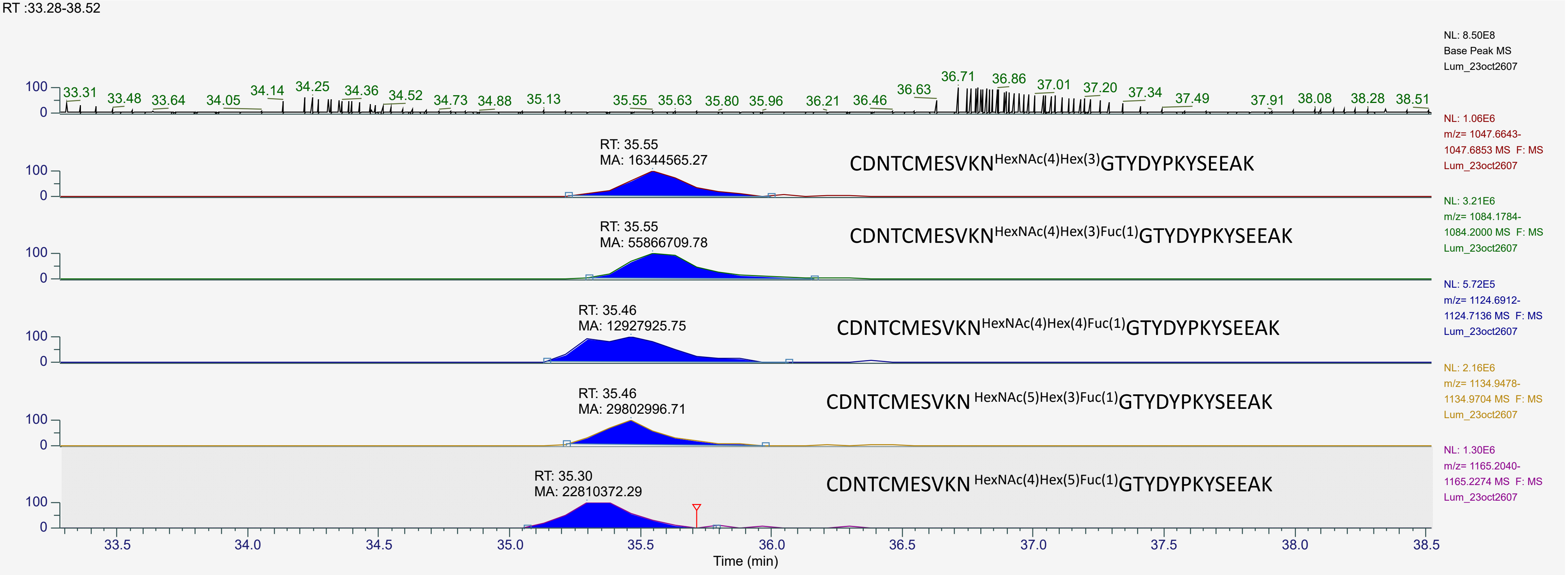

RT (Min)	Base Peak	Peak Area	Peak Height	Baseline Width	SignalToNoise
35.54717	1047.675	16344565	1033801	0.778267	INF
35.54717	1084.188	55866710	3106124	0.859799	INF
35.46364	1124.701	12927926	555484.3	0.92645	INF
35.46364	1134.959	29802997	2033326	0.759689	INF
35.29684	1165.216	22810372	1276721	0.722631	INF

Y2-CHO-S2 trypsin

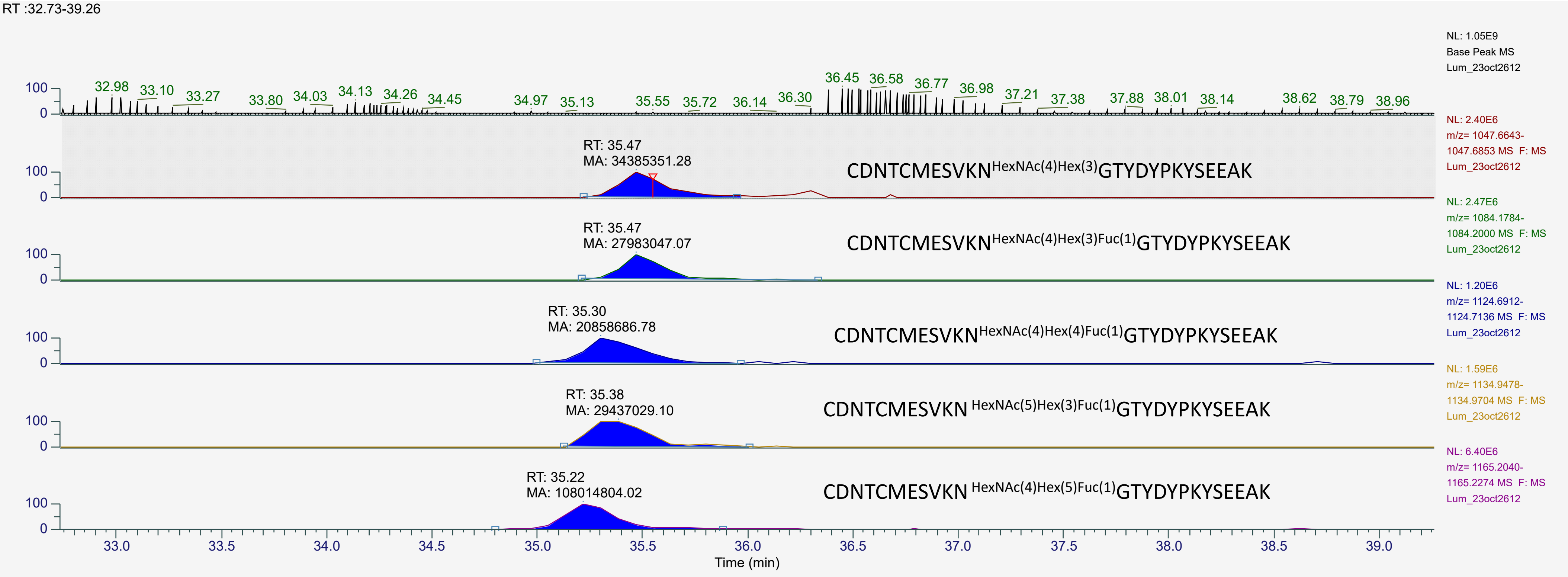

RT (Min)	Base Peak	Peak Area	Peak Height	Baseline Width	SignalToNoise
35.46839	1047.675	34385351	2329375	0.725042	INF
35.46839	1084.188	27983047	2301306	1.122199	INF
35.30073	1124.702	20858687	1167134	0.969426	INF
35.38459	1134.958	29437029	1537653	0.881716	INF
35.21721	1165.215	1.08E+08	6400889	1.084834	INF

Y2-CHO-S3 trypsin

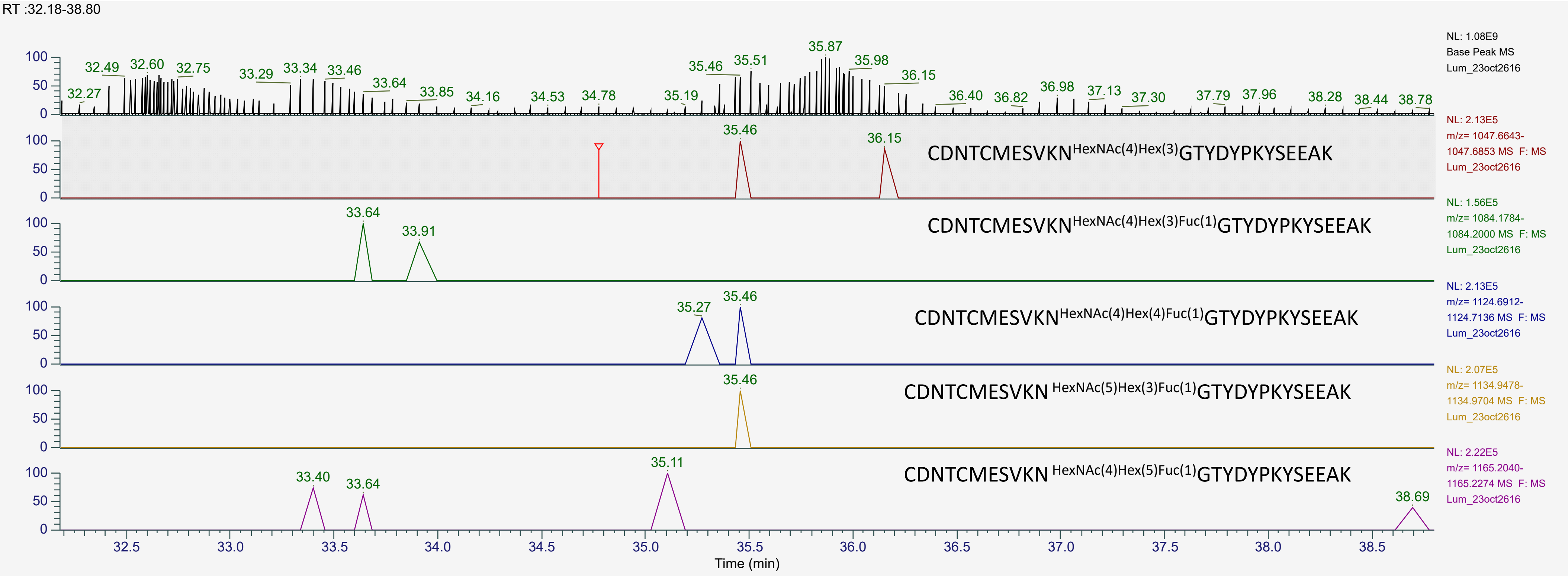

Y2-CHO-WT trypsin

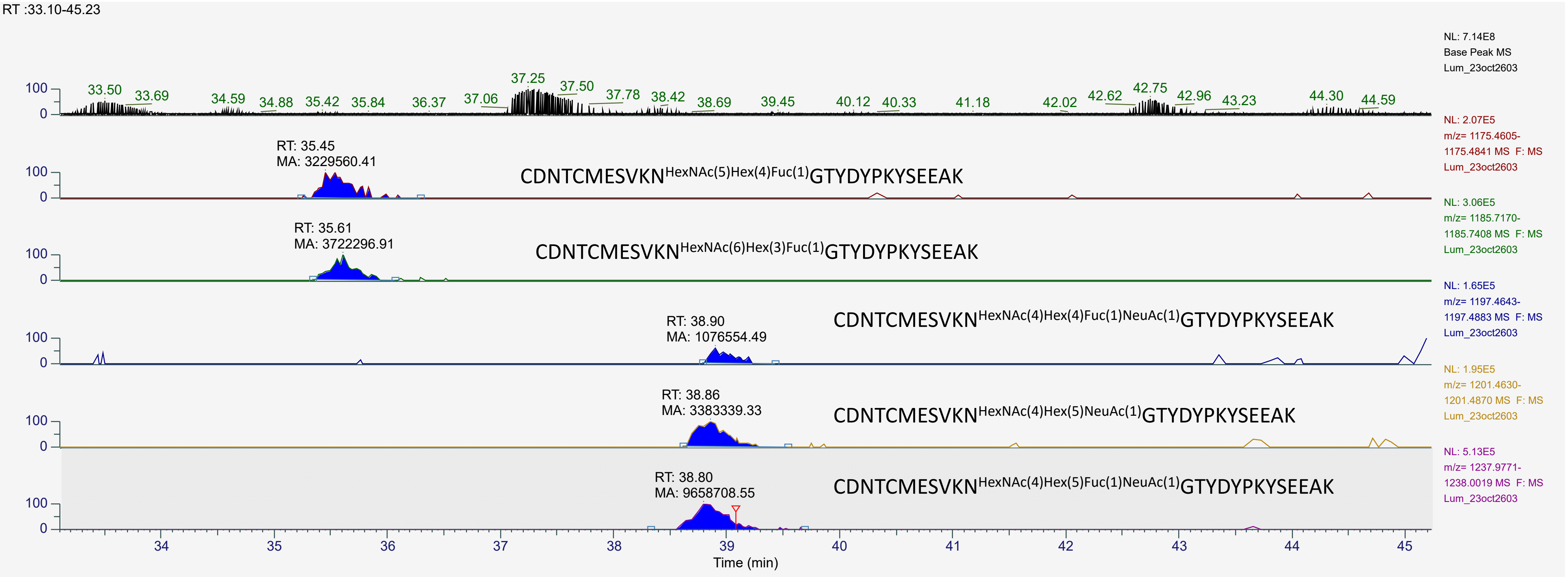

RT (Min)	Base Peak	Peak Area	Peak Height	Baseline Width	SignalToNoise
35.4522	1175.473	3229560	201555.3	1.055653	INF
35.61127	1185.729	3722297	297059.3	0.729516	INF
38.89956	1197.478	1076554	90553.59	0.643388	INF
38.85666	1201.476	3383339	189035.2	0.926478	INF
38.7991	1237.991	9658709	512506.7	1.355403	INF

Y2-CHO-S1 trypsin

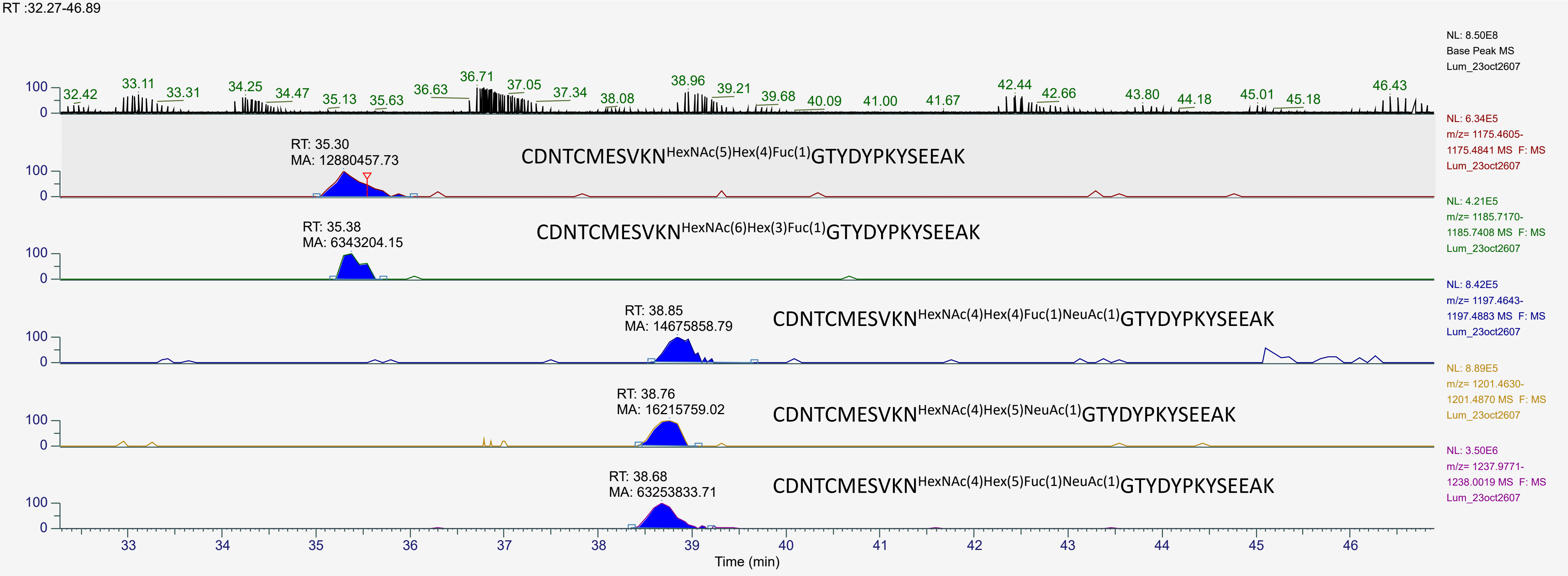

RT (Min)	Base Peak	Peak Area	Peak Height	Baseline Width	SignalToNoise
35.29684	1175.473	12880458	624137.8	1.033946	INF
35.37992	1185.727	6343204	415191.9	0.537652	INF
38.84514	1197.478	14675859	814197.8	1.095983	INF
38.76185	1201.476	16215759	870524.9	0.641047	INF
38.67807	1237.99	63253834	3391869	0.847836	INF

Y2-CHO-S2 trypsin

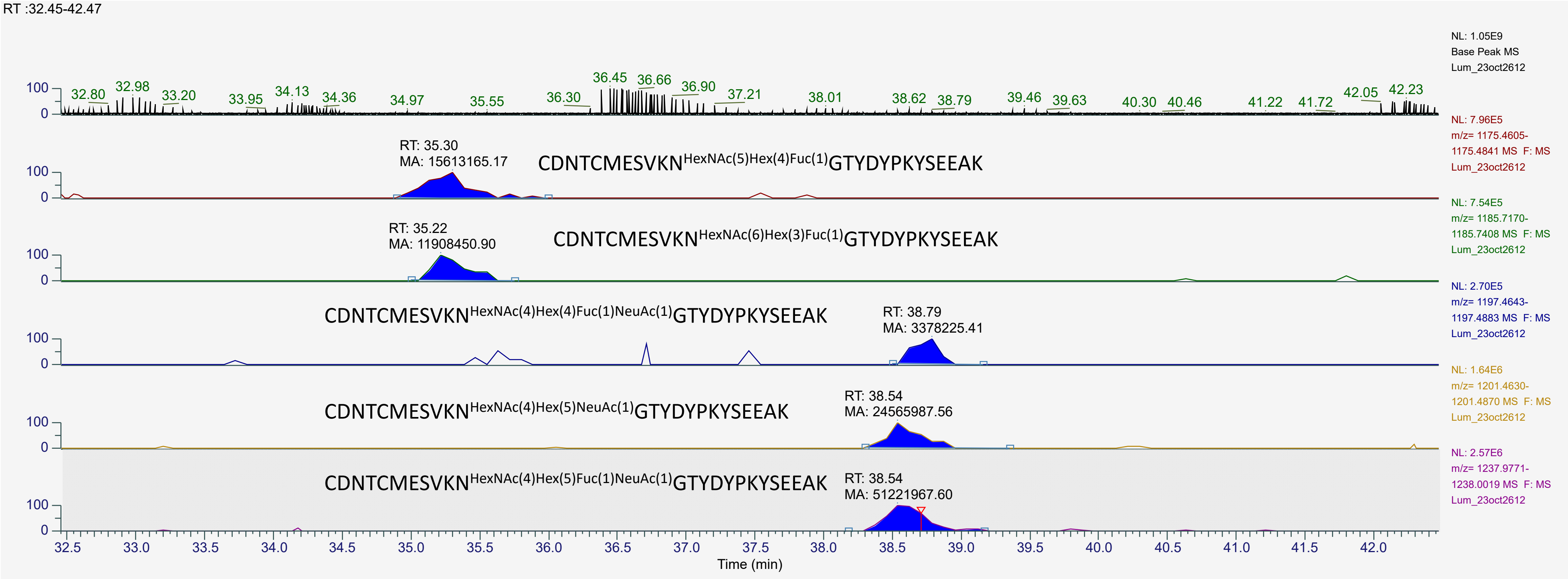

RT (Min)	Base Peak	Peak Area	Peak Height	Baseline Width	SignalToNoise
35.30073	1175.473	15613165	779662.6	1.105575	INF
35.21721	1185.727	11908451	730323.9	0.751224	INF
38.79079	1197.477	3378225	259551.8	0.659024	INF
38.53912	1201.475	24565988	1583323	1.048769	INF
38.53912	1237.989	51221968	2566893	0.984993	INF

Y2-CHO-S3 trypsin

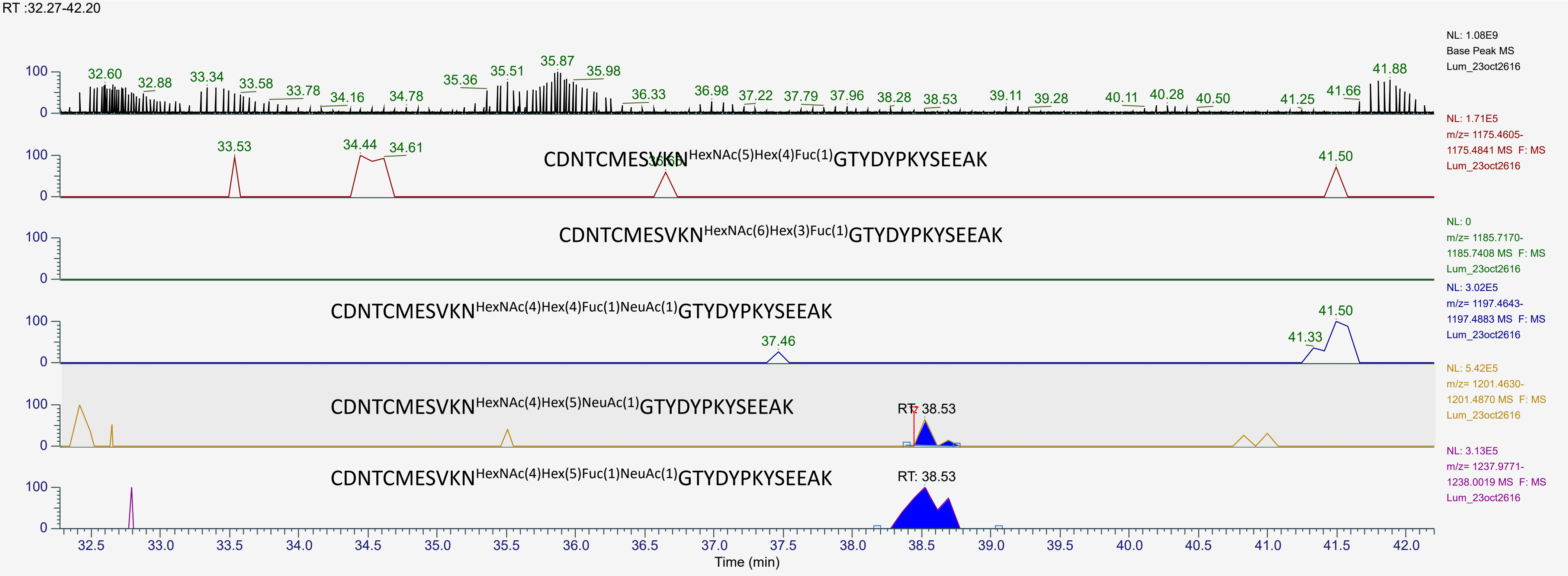

RT (Min)	Base Peak	Peak Area	Peak Height	Baseline Width	SignalToNoise
38.52578	1201.476	2010672	325190.6	0.365147	INF
38.52578	1237.99	5214459	313283.2	0.877757	INF

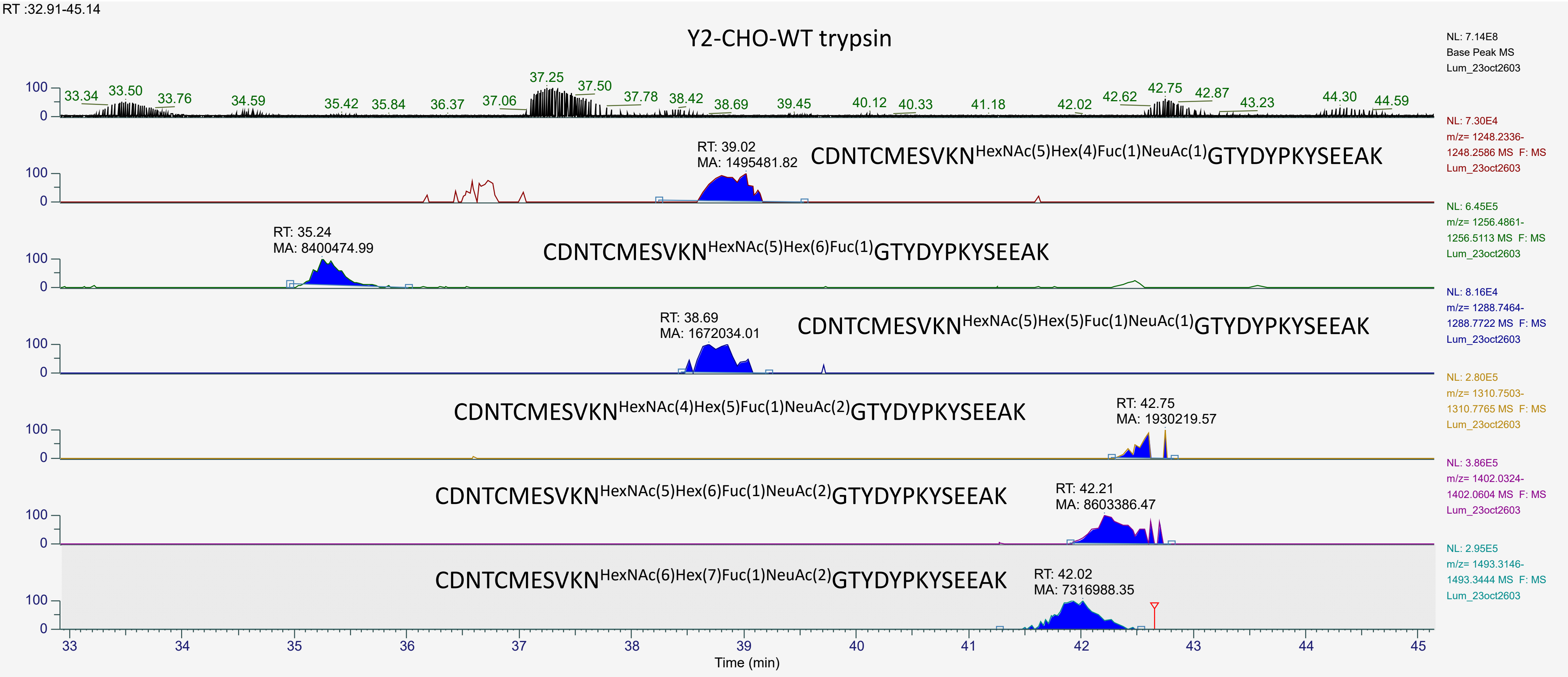

RT (Min)	Base Peak	Peak Area	Peak Height	Baseline Width	SignalToNoise
39.02081	1248.244	1495482	70282.64	1.287121	INF
35.24262	1256.499	8400475	576498.8	1.059944	INF
38.69143	1288.76	1672034	79318.54	0.769633	INF
42.7467	1310.763	1930220	278332.5	0.562199	INF
42.20773	1402.048	8603386	374894.9	0.899519	INF
42.01754	1493.33	7316988	295443.8	1.262786	INF

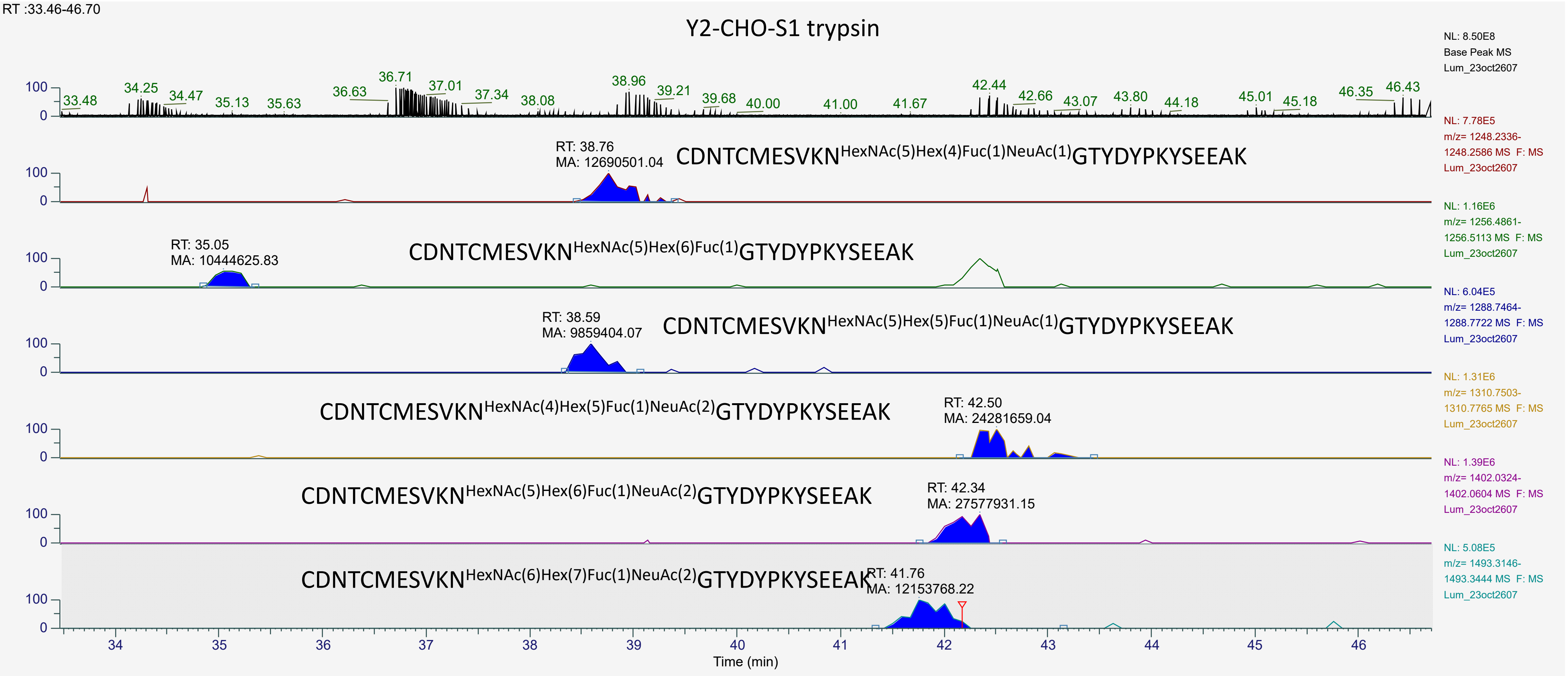

RT (Min)	Base Peak	Peak Area	Peak Height	Baseline Width	SignalToNoise
38.76185	1248.245	12690501	761579.6	0.945623	INF
35.04719	1256.499	10444626	584199.8	0.505581	INF
38.59448	1288.757	9859404	588067.3	0.72099	INF
42.50325	1310.763	24281659	1314809	1.292164	INF
42.34184	1402.044	27577931	1384340	0.805262	INF
41.75632	1493.33	12153768	507716.1	1.816521	INF

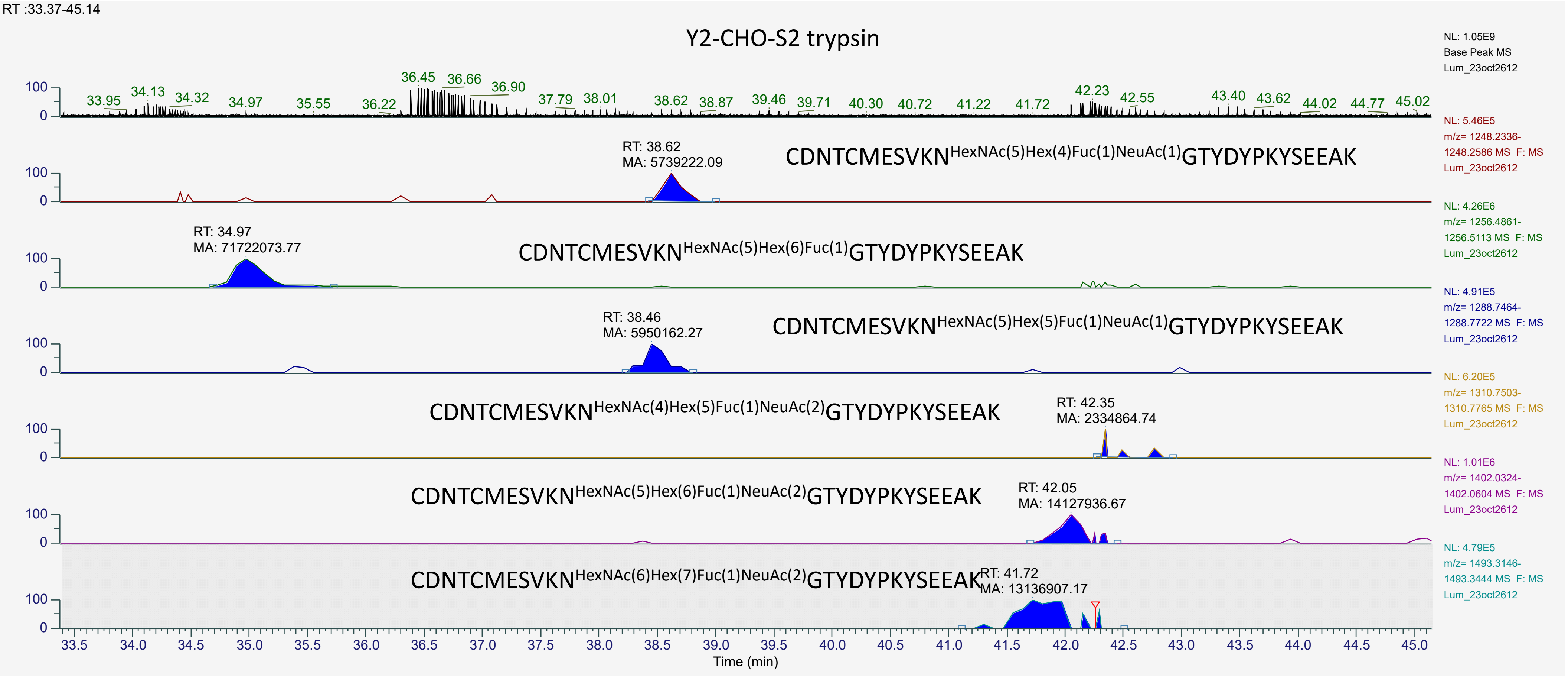

RT (Min)	Base Peak	Peak Area	Peak Height	Baseline Width	SignalToNoise
38.62178	1248.243	5739222	529171.6	0.574244	INF
34.97336	1256.498	71722074	4194209	1.031974	INF
38.45572	1288.761	5950162	484691.1	0.582673	INF
42.34785	1310.766	2334865	597149.6	0.657588	INF
42.05414	1402.037	14127937	1002128	0.749151	INF
41.71901	1493.329	13136907	478961.9	1.390092	INF

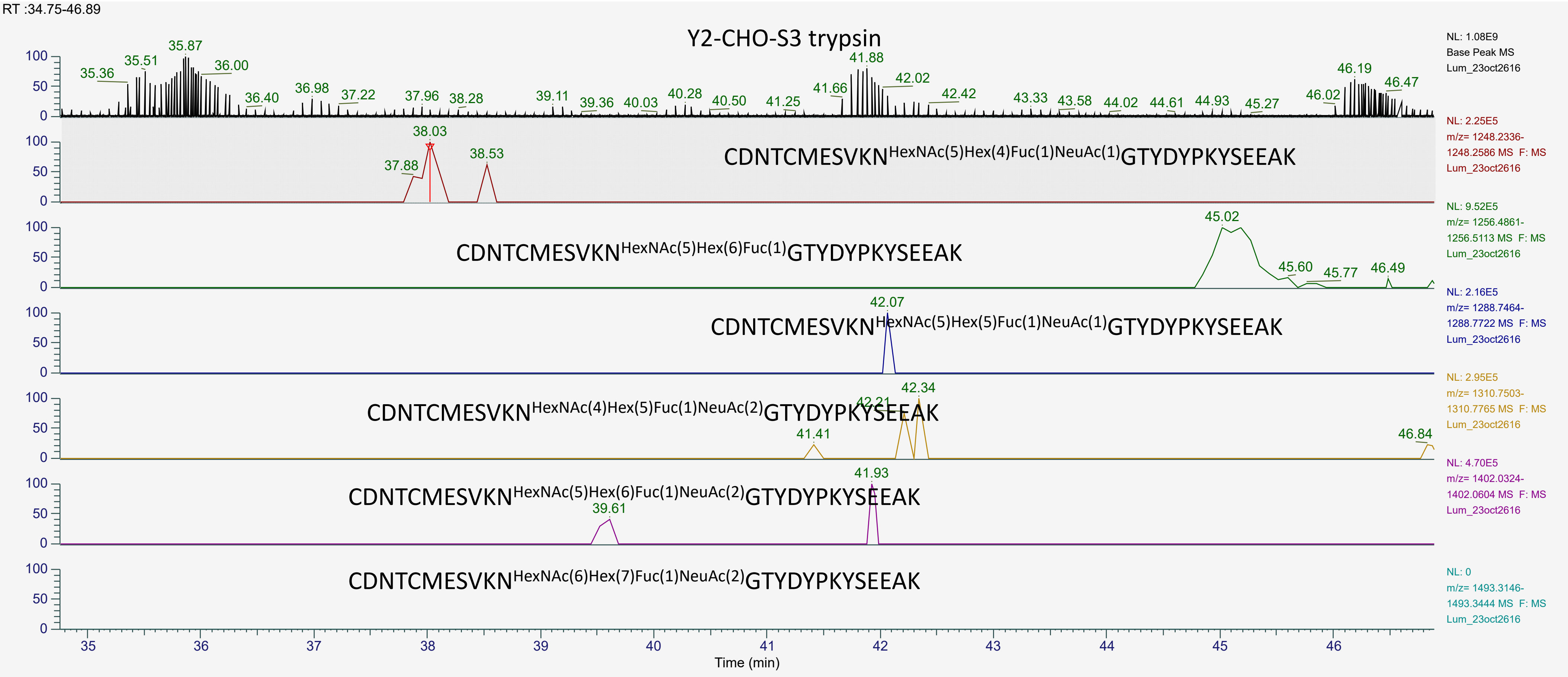
RT :34.75-46.89
Y2-CHO-S3 trypsin
NL: 1.08E9
Base Peak MS
Lum_23oct2616
CDNTCMESVKN HexNAc(5)Hex(4)Fuc(1)NeuAc(1) GTYDYPKYSEEAK
NL: 2.25E5
m/z= 1248.2336-1248.2586 MS F: MS
Lum_23oct2616
CDNTCMESVKN HexNAc(5)Hex(6)Fuc(1) GTYDYPKYSEEAK
NL: 9.52E5
m/z= 1256.4861-1256.5113 MS F: MS
Lum_23oct2616
CDNTCMESVKN HexNAc(5)Hex(5)Fuc(1)NeuAc(1) GTYDYPKYSEEAK
NL: 2.16E5
m/z= 1288.7464-1288.7722 MS F: MS
Lum_23oct2616
CDNTCMESVKN HexNAc(4)Hex(5)Fuc(1)NeuAc(2) GTYDYPKYSEEAK
NL: 2.95E5
m/z= 1310.7503-1310.7765 MS F: MS
Lum_23oct2616
CDNTCMESVKN HexNAc(5)Hex(6)Fuc(1)NeuAc(2) GTYDYPKYSEEAK
NL: 4.70E5
m/z= 1402.0324-1402.0604 MS F: MS
Lum_23oct2616
CDNTCMESVKN HexNAc(6)Hex(7)Fuc(1)NeuAc(2) GTYDYPKYSEEAK
NL: 0
m/z= 1493.3146-1493.3444 MS F: MS
Lum_23oct2616
Time (min)

Y2-CHO-WT trypsin

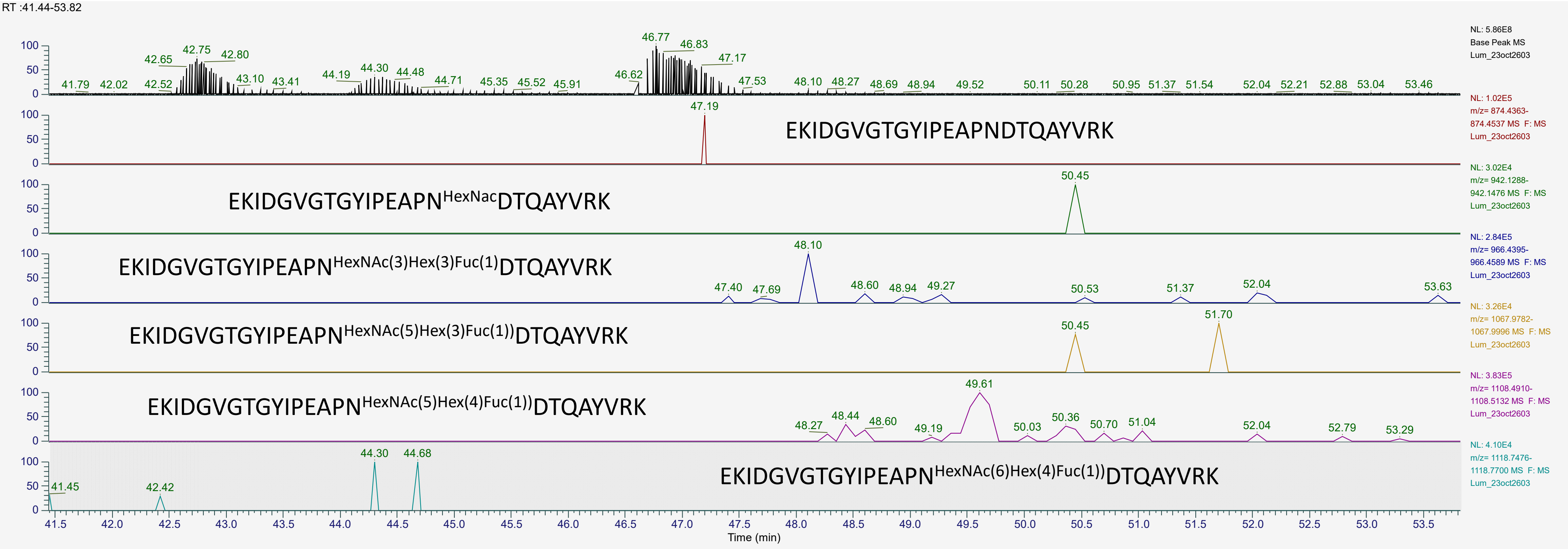

Y2-CHO-S1 trypsin

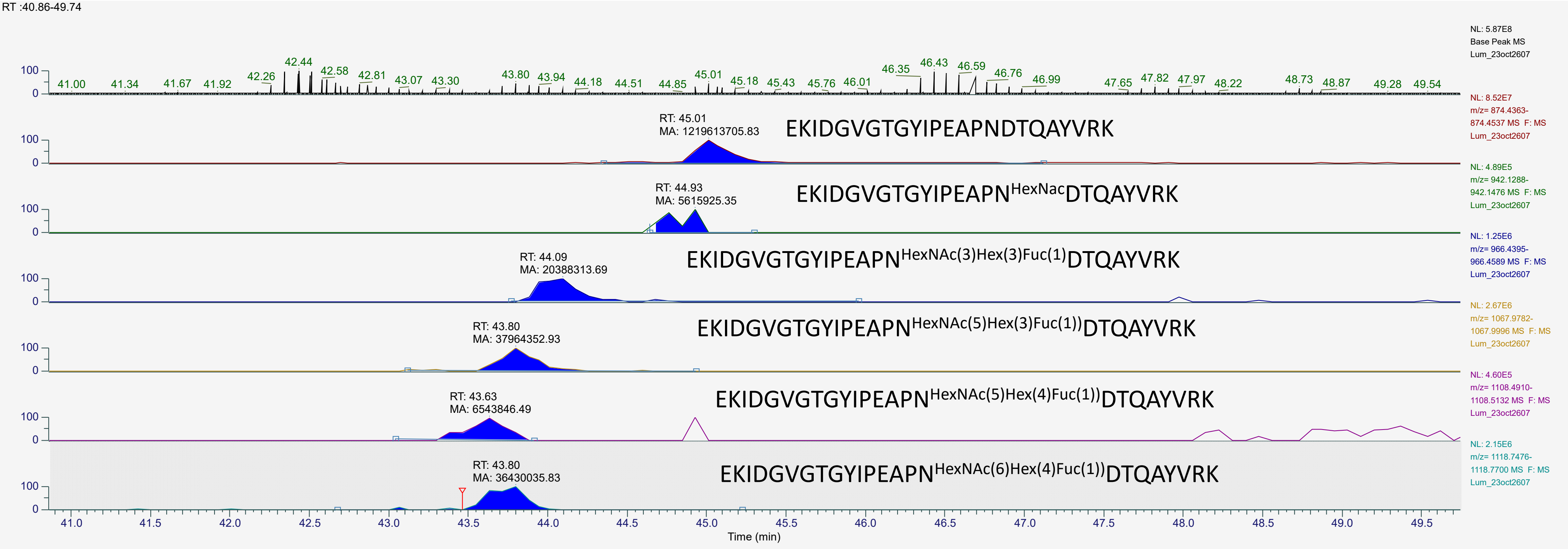

RT (Min)	Base Peak	Peak Area	Peak Height	Baseline Width	SignalToNoise
45.01185	874.4441	1.22E+09	84231886	2.767441	INF
44.92956	942.1388	5615925	488542.3	0.655447	INF
44.09295	966.4498	20388314	1200775	2.184822	INF
43.79769	1067.989	37964353	2606774	1.820685	INF
43.63107	1108.503	6543846	412510.9	0.873929	INF
43.79769	1118.759	36430036	2146168	2.548959	INF

Y2-CHO-S2 trypsin

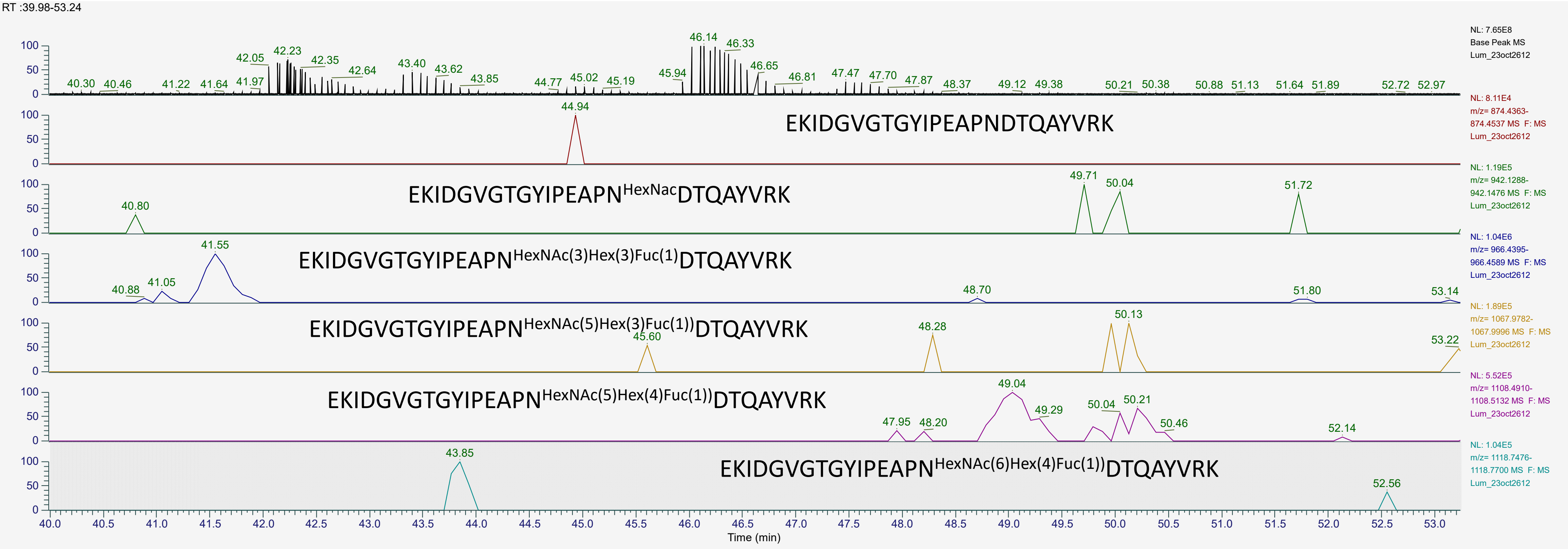

Y2-CHO-S3 trypsin

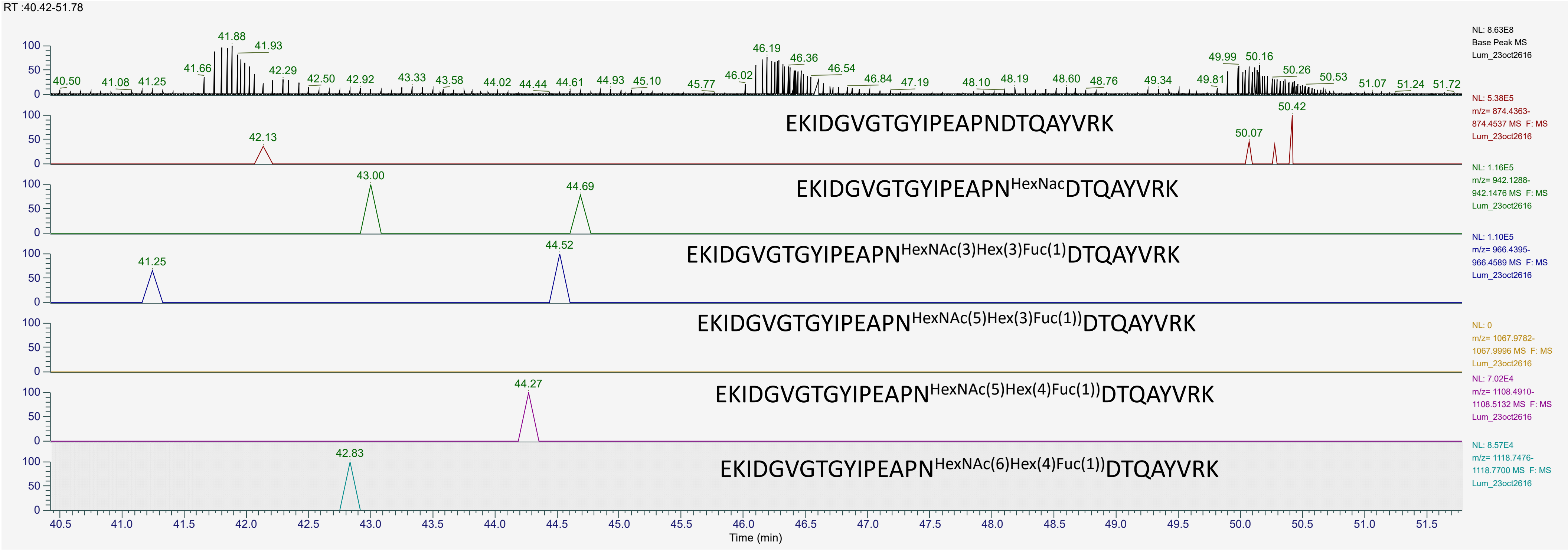

Y2-CHO-WT trypsin

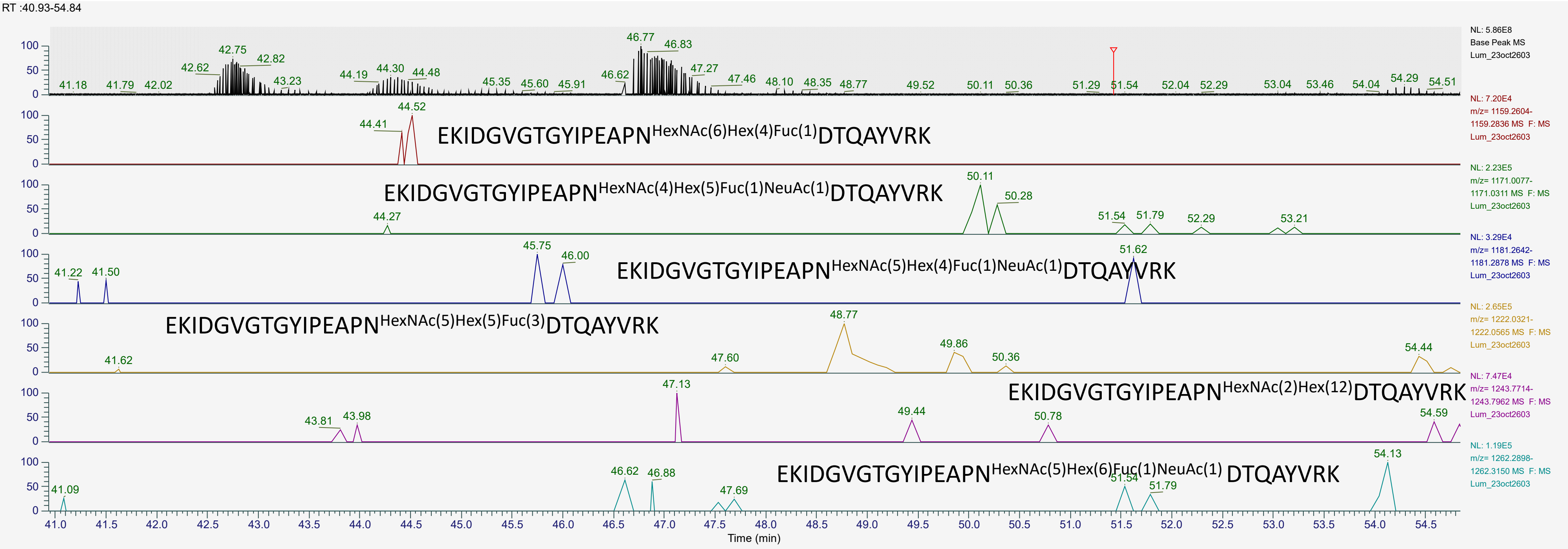

Y2-CHO-S1 trypsin

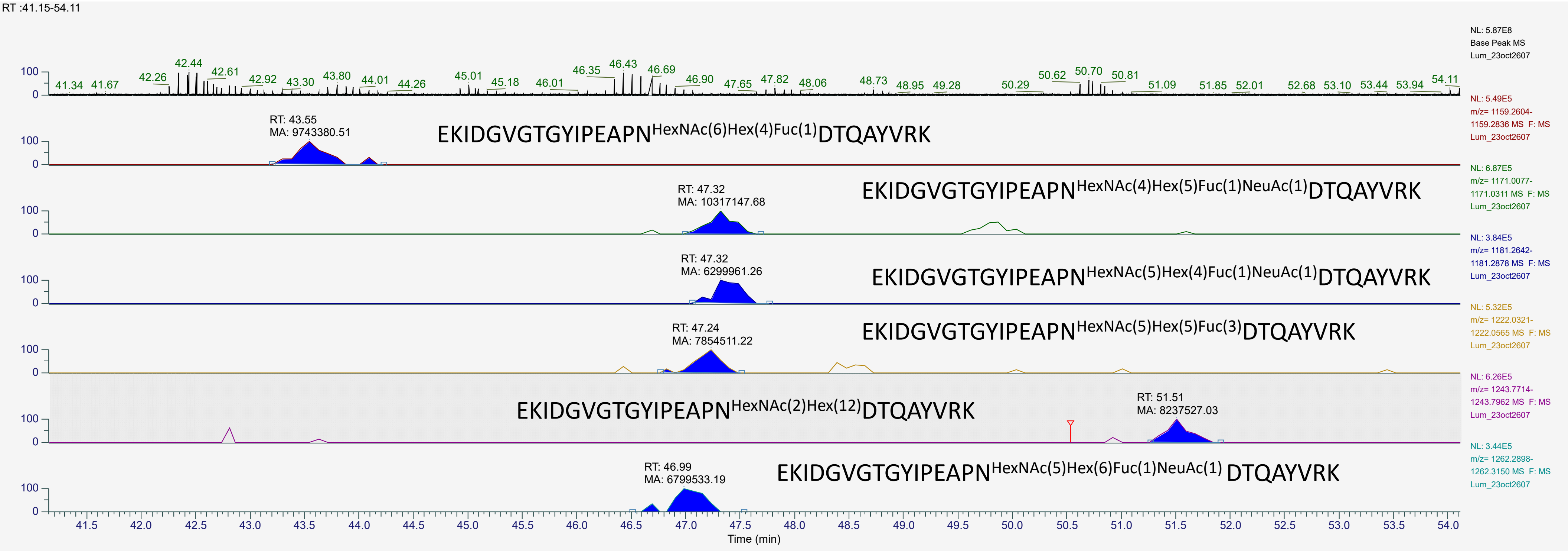

RT (Min)	Base Peak	Peak Area	Peak Height	Baseline Width	SignalToNoise
43.54728	1159.272	9743381	531769.2	1.019832	INF
47.31871	1171.019	10317148	679164.5	0.701445	INF
47.31871	1181.276	6299961	369266.2	0.70642	INF
47.23542	1222.04	7854511	520411.8	0.746218	INF
51.51081	1243.793	8237527	614402.5	0.64235	INF
46.98507	1262.3	6799533	344340.2	1.024807	INF

Y2-CHO-S2 trypsin

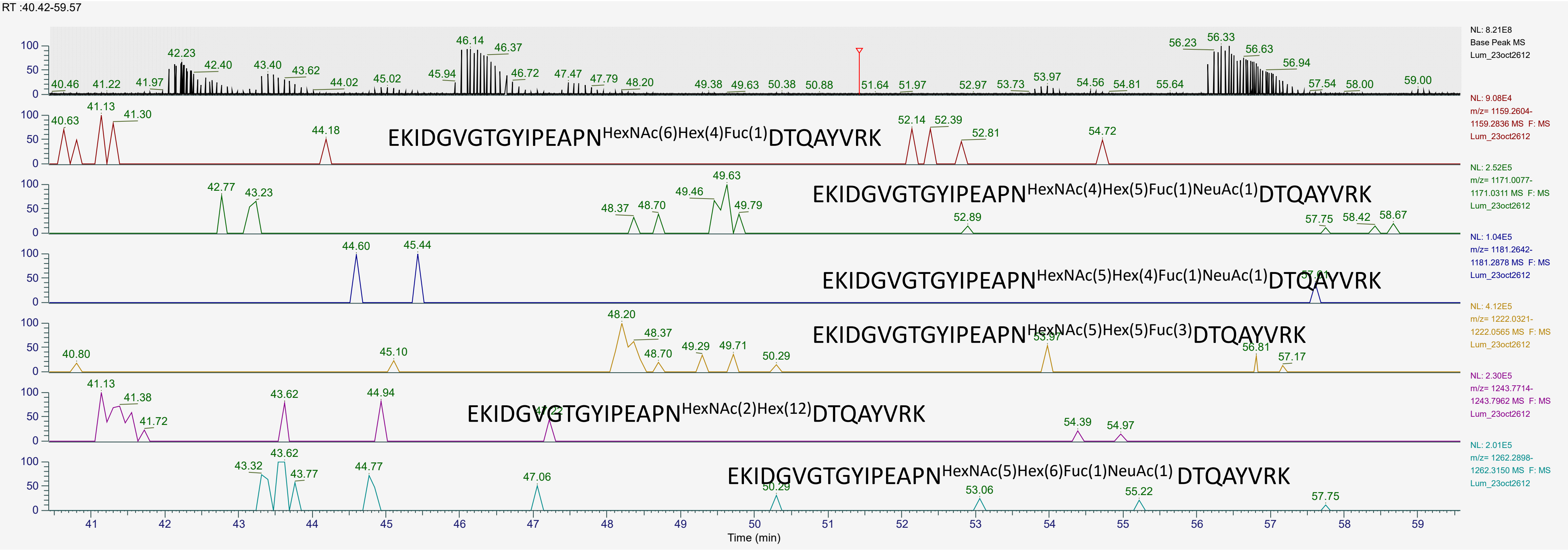

Y2-CHO-S3 trypsin

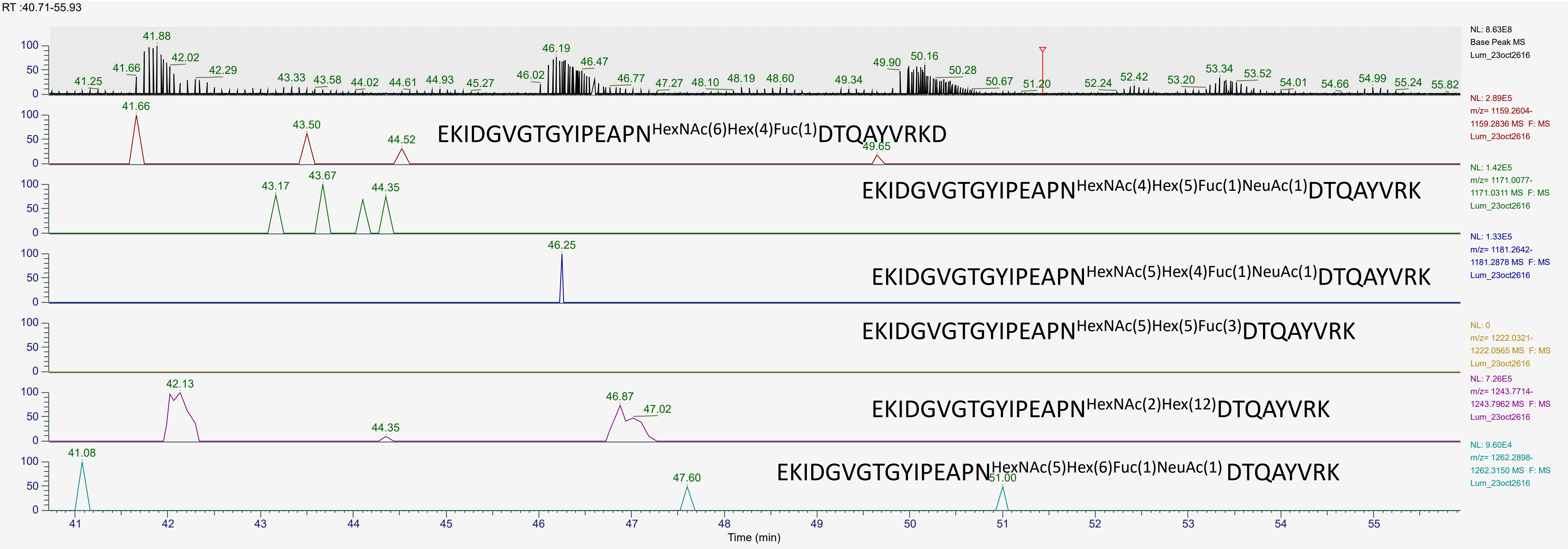

Y2-CHO-WT trypsin

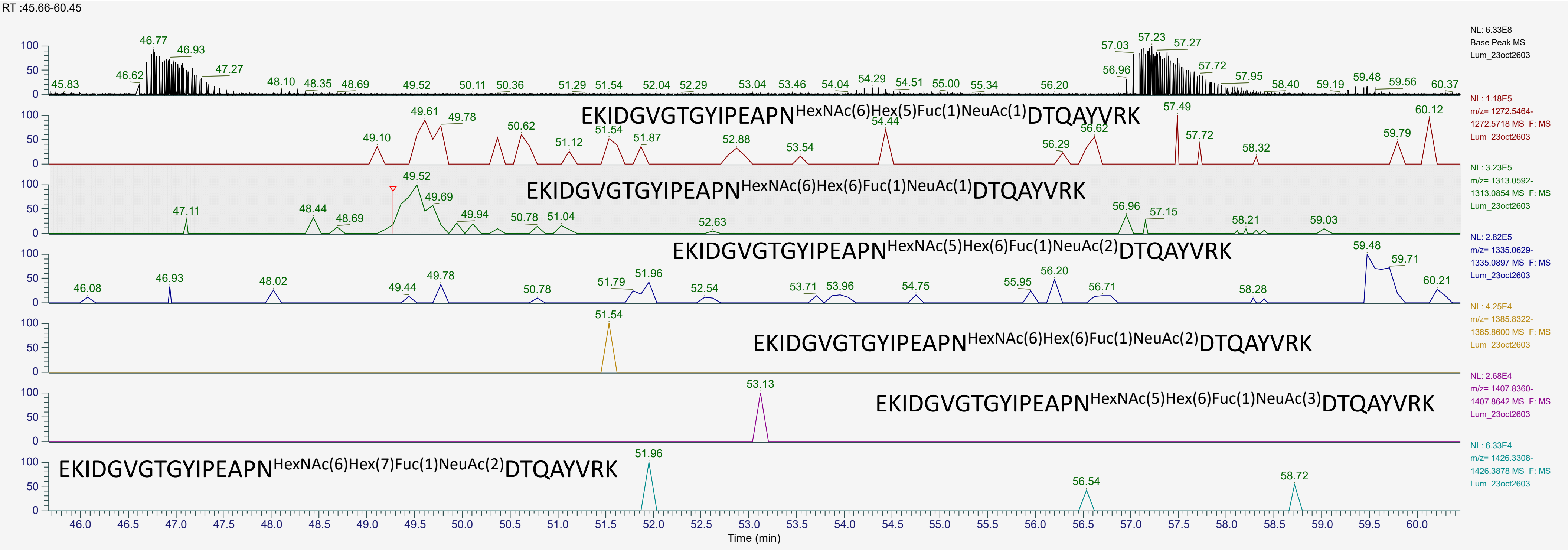

Y2-CHO-S1 trypsin

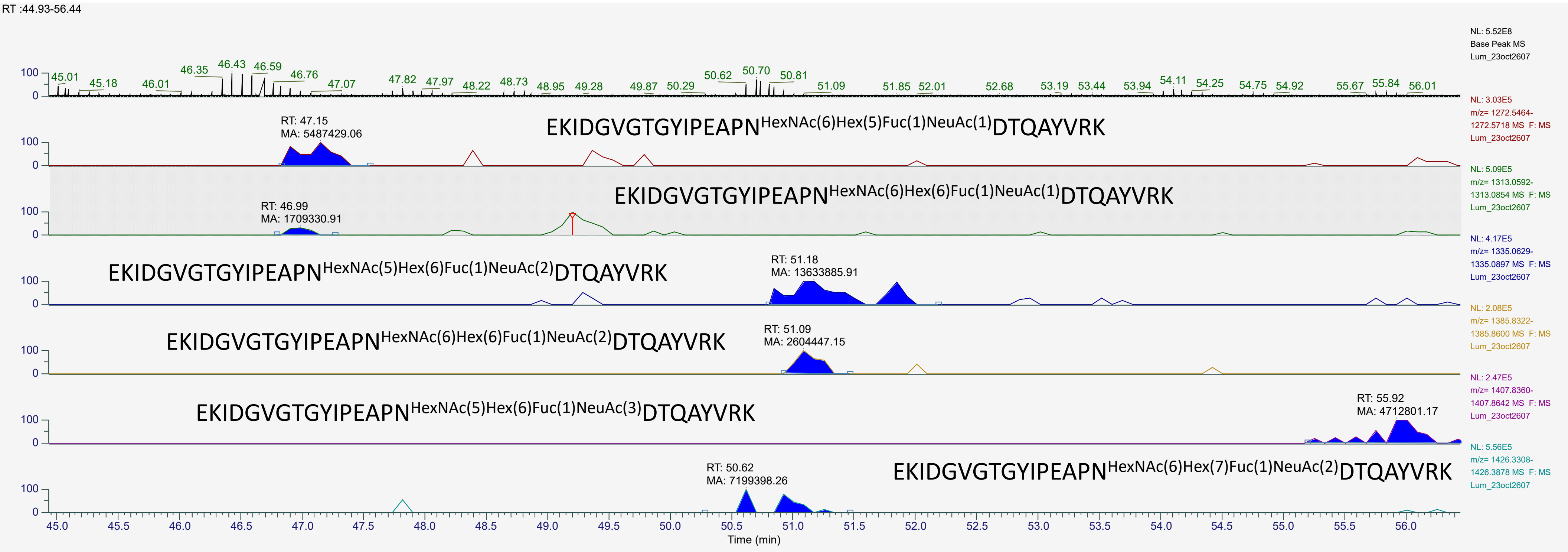

RT (Min)	Base Peak	Peak Area	Peak Height	Baseline Width	SignalToNoise
47.15173	1272.557	5487429	299468.3	0.722644	INF
46.98507	1313.072	1709331	136762.9	0.47703	INF
51.1751	1335.076	13633886	410841.4	1.383641	INF
51.0911	1385.846	2604447	199184.1	0.543014	INF
55.92403	1407.853	4712801	238695.2	1.597714	INF
50.62034	1426.36	7199398	555639.3	1.185232	INF

Y2-CHO-S2 trypsin

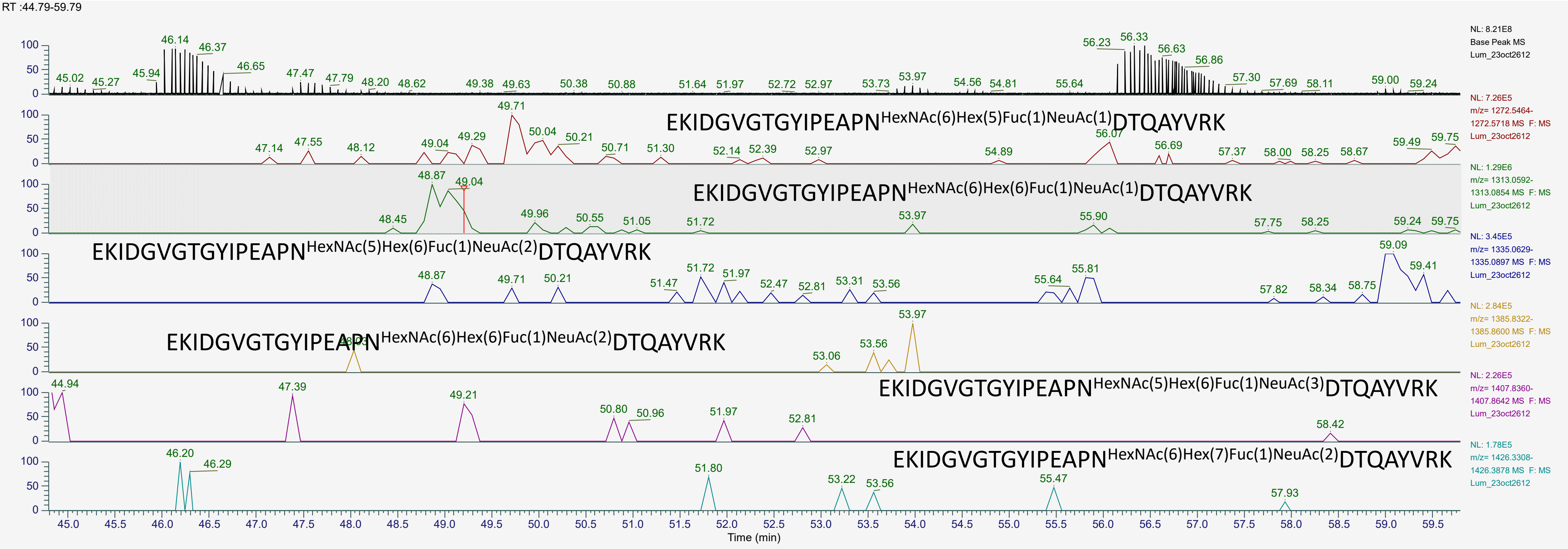

Y2-CHO-S1 trypsin

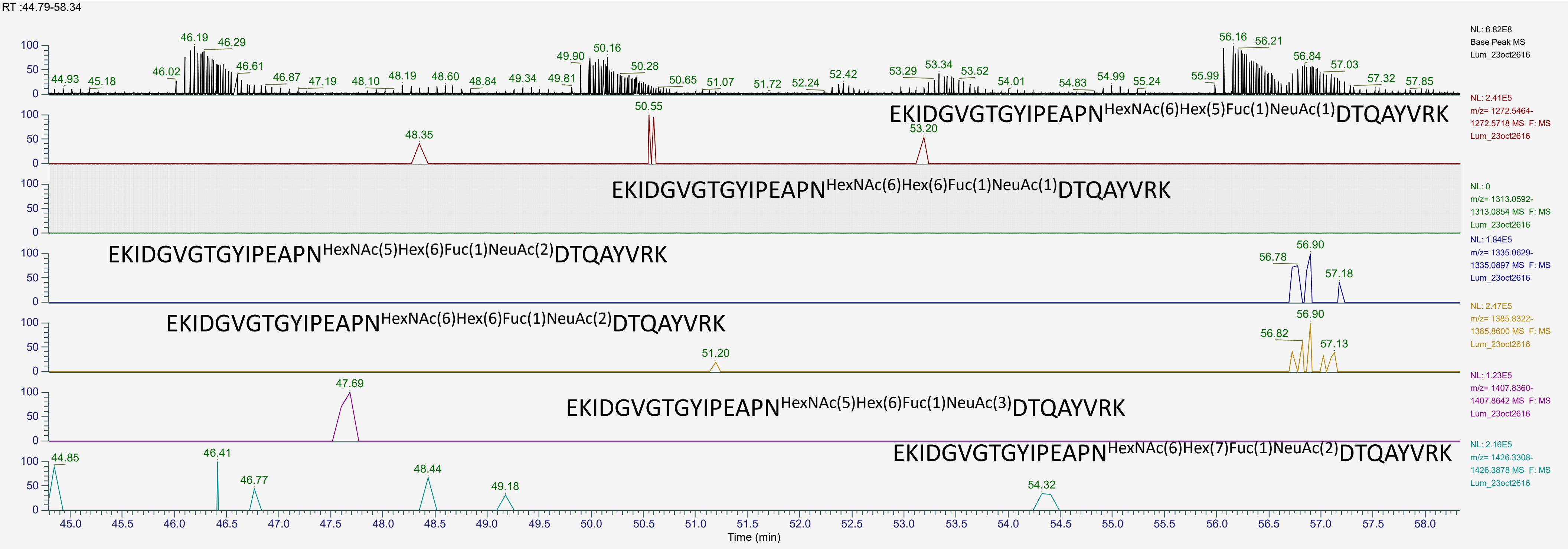

Y2-CHO-WT trypsin

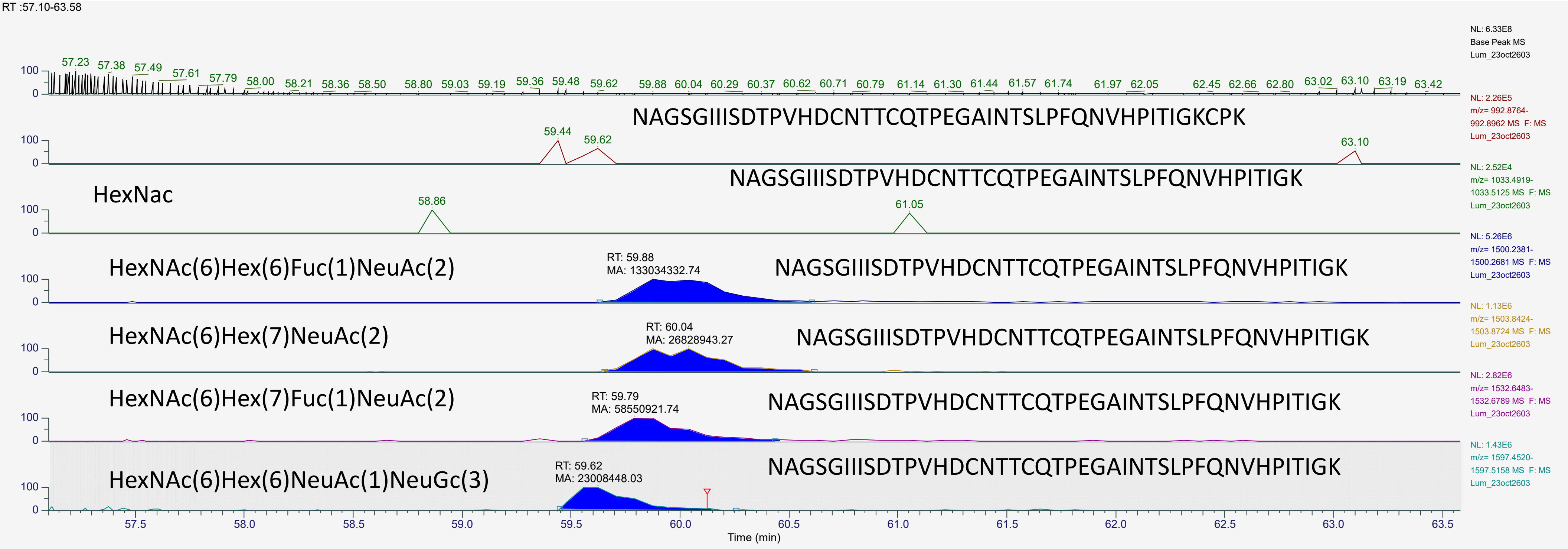

RT (Min)	Base Peak	Peak Area	Peak Height	Baseline Width	SignalToNoise
59.87528	1500.252	1.33E+08	5134288	0.973171	INF
60.03994	1503.858	26828943	1118442	0.962277	INF
59.79005	1532.663	58550922	2779640	0.874891	INF
59.62333	1597.488	23008448	1328821	0.805916	INF

Y2-CHO-S1 trypsin

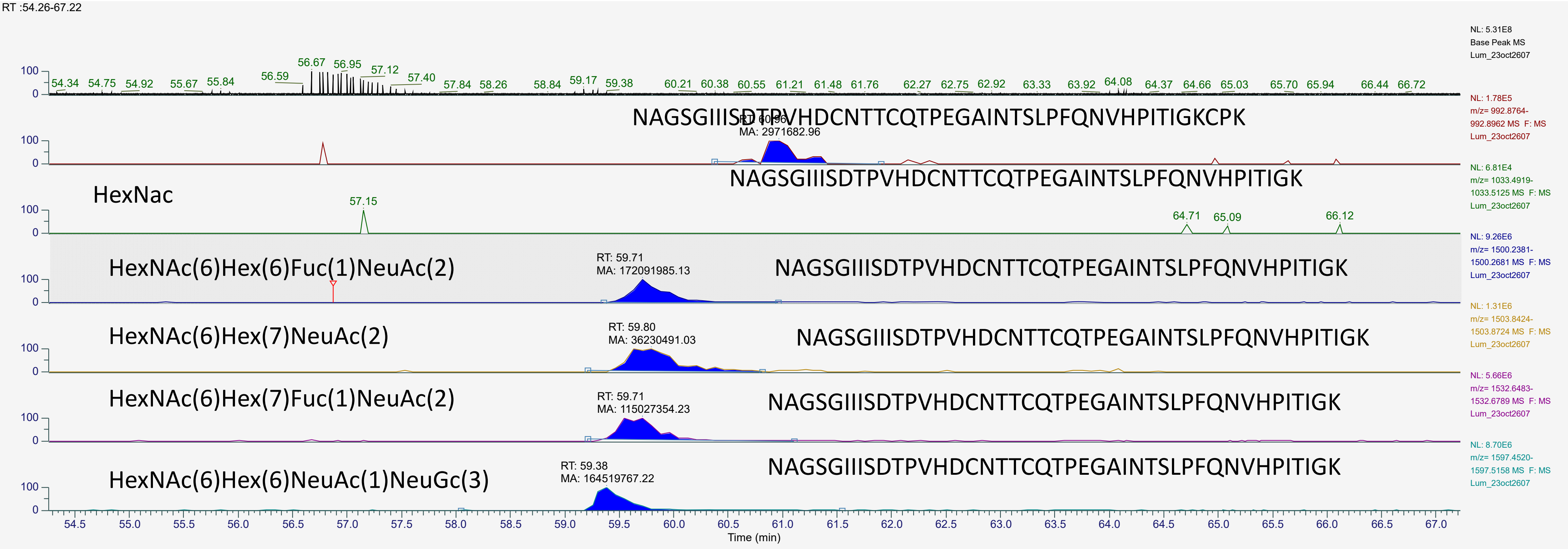

RT (Min)	Base Peak	Peak Area	Peak Height	Baseline Width	SignalToNoise
60.96403	992.8828	2971683	165943	1.529375	INF
59.71382	1500.253	1.72E+08	9050099	1.602203	INF
59.79644	1503.858	36230491	1228840	1.602203	INF
59.71382	1532.661	1.15E+08	5172210	1.893512	INF
59.38325	1597.482	1.65E+08	8703807	3.495798	INF

Y2-CHO-S2 trypsin

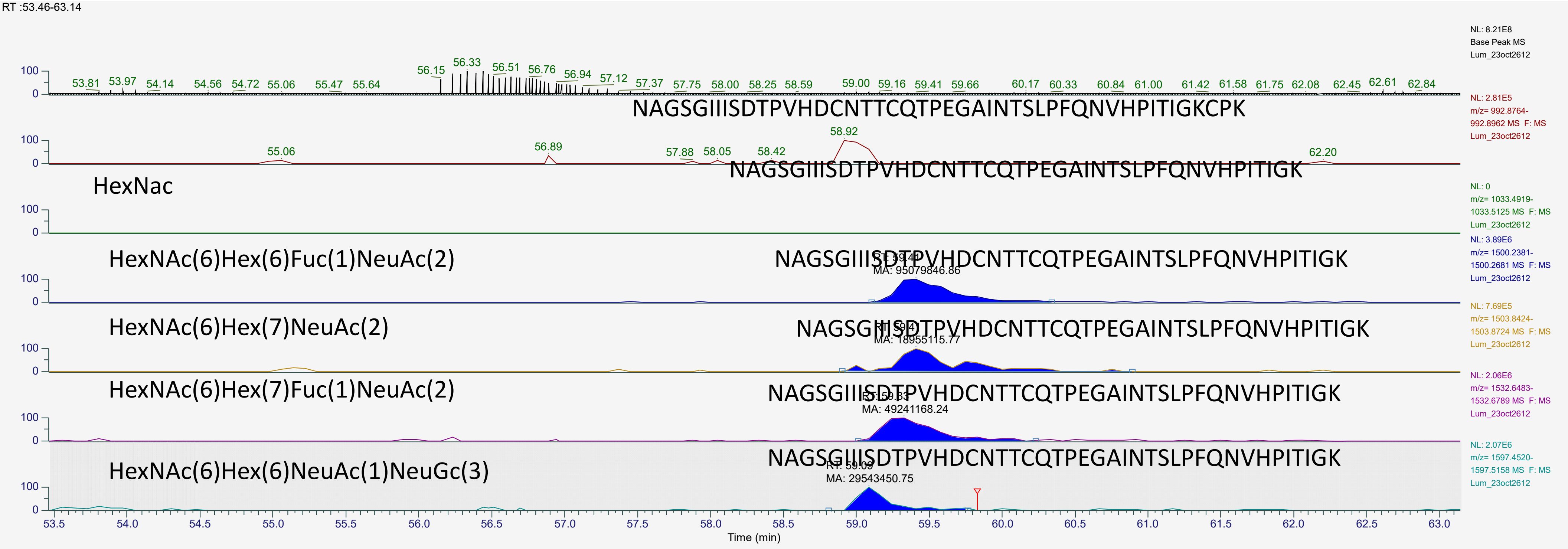

RT (Min)	Base Peak	Peak Area	Peak Height	Baseline Width	SignalToNoise
59.40956	1500.25	95079847	3841580	1.231791	INF
59.40956	1503.857	18955116	744787.3	1.986059	INF
59.32511	1532.662	49241168	2028659	1.214745	INF
59.08507	1597.483	29543451	2072086	0.959866	INF

Y2-CHO-S3 trypsin

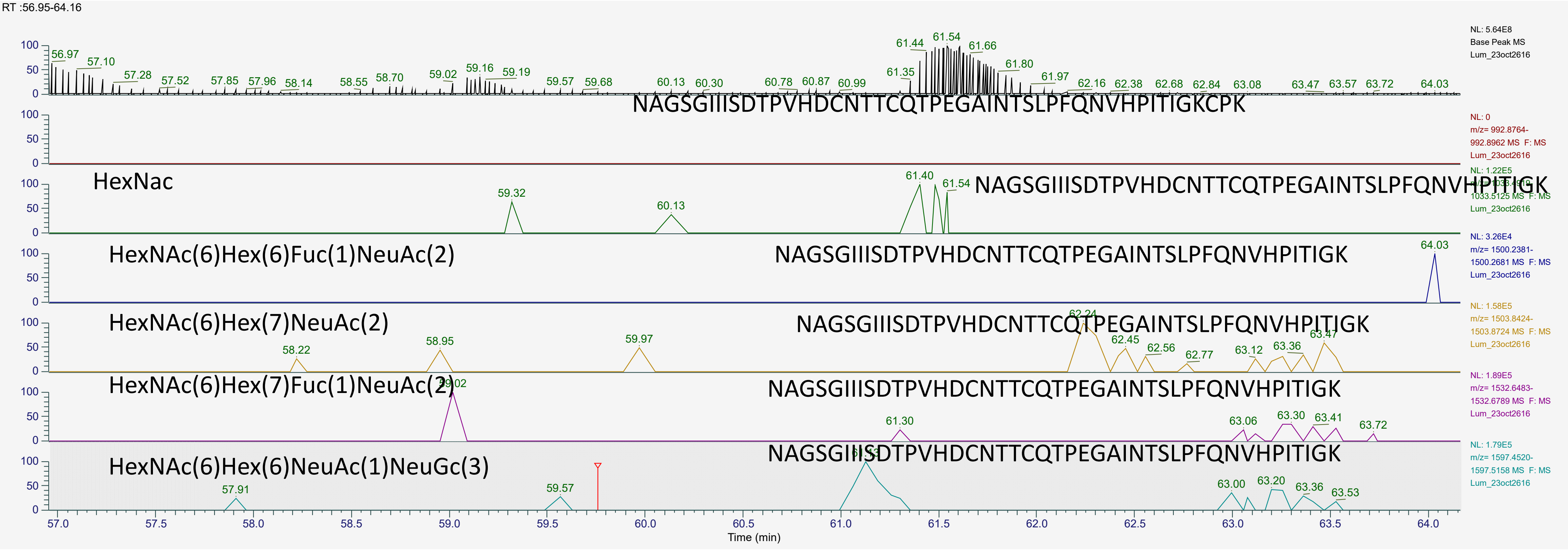

Y2-CHO-WT trypsin

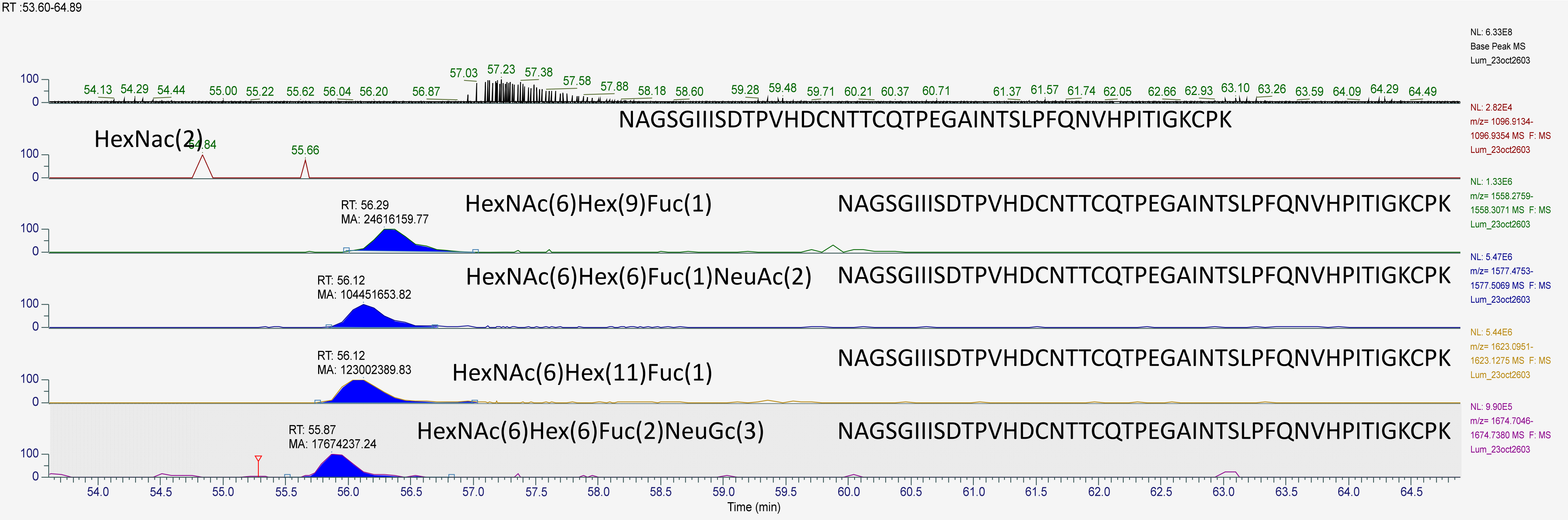

RT (Min)	Base Peak	Peak Area	Peak Height	Baseline Width	SignalToNoise
56.28874	1558.29	24616160	1251302	1.031556	INF
56.12087	1577.489	1.04E+08	5384899	0.850443	INF
56.12087	1623.111	1.23E+08	5436749	1.251513	INF
55.86929	1674.722	17674237	990210.1	1.314482	INF

Y2-CHO-S1 trypsin

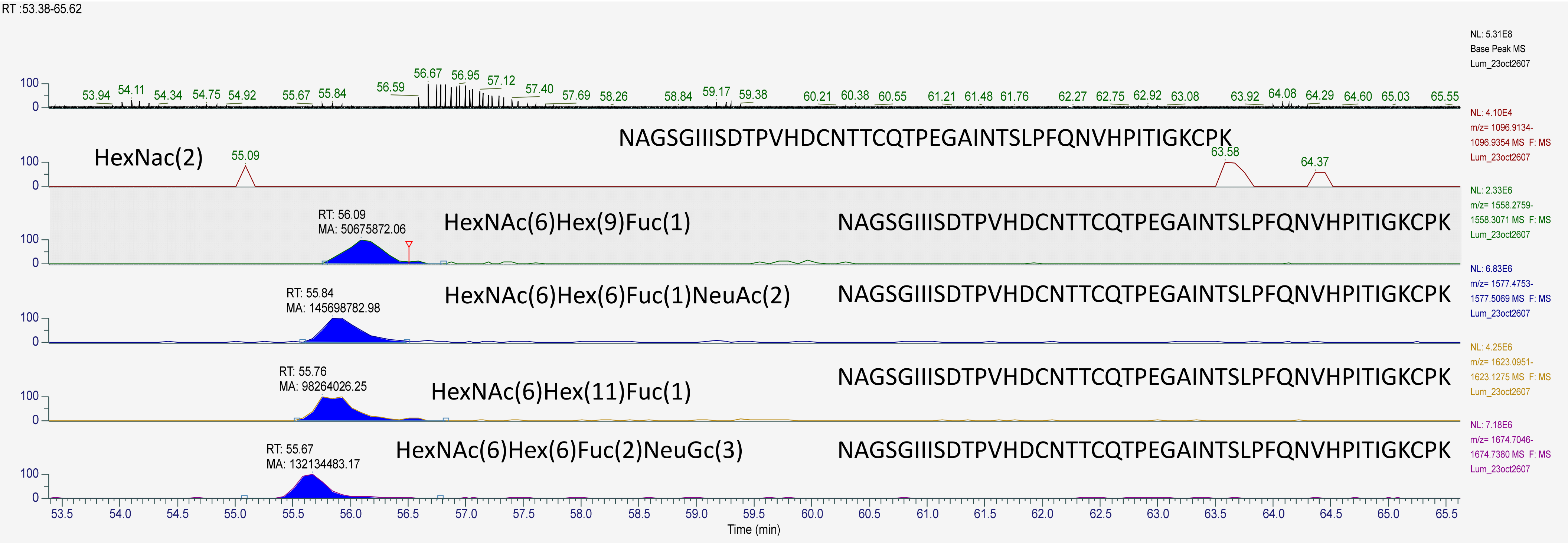

RT (Min)	Base Peak	Peak Area	Peak Height	Baseline Width	SignalToNoise
56.08928	1558.29	50675872	2295221	1.028571	INF
55.84055	1577.49	1.46E+08	6720851	0.905143	INF
55.75731	1623.111	98264026	4172257	1.289143	INF
55.67335	1674.721	1.32E+08	7177069	1.700571	INF

Y2-CHO-S2 trypsin

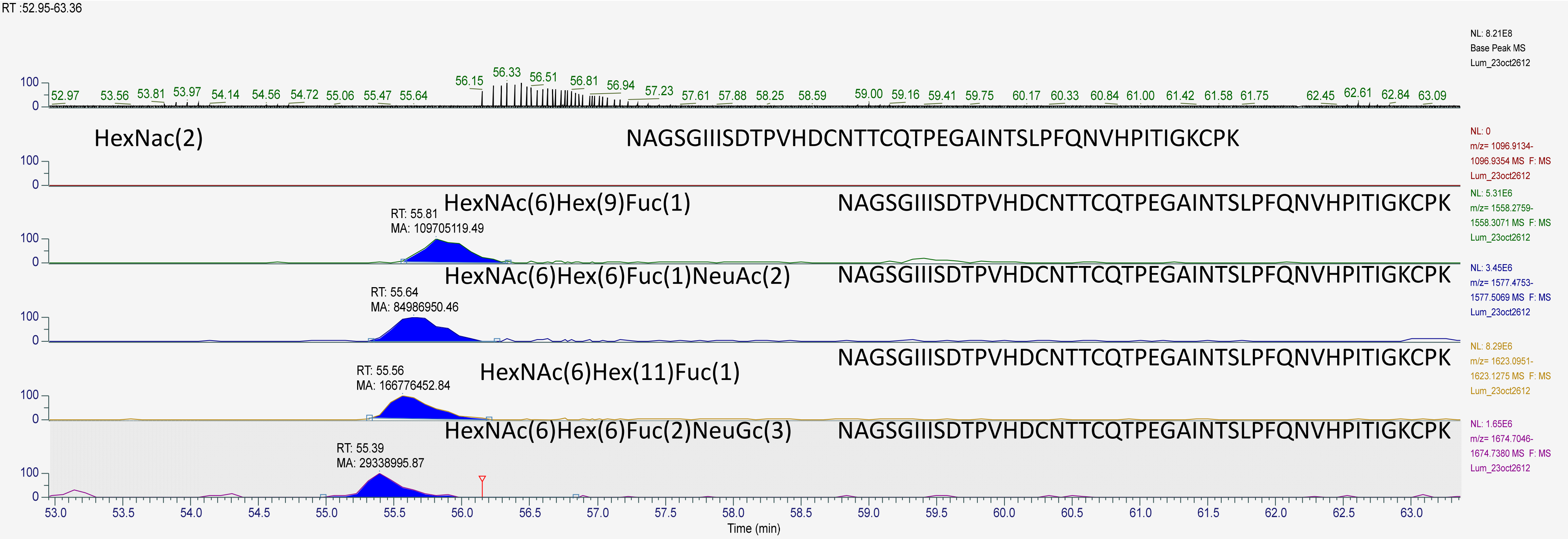

RT (Min)	Base Peak	Peak Area	Peak Height	Baseline Width	SignalToNoise
55.81161	1558.288	1.1E+08	5068700	0.77014	INF
55.6443	1577.485	84986950	3452981	0.927668	INF
55.56062	1623.108	1.67E+08	7689248	0.880622	INF
55.39123	1674.72	29338996	1653248	1.862572	INF

Y2-CHO-S3 trypsin

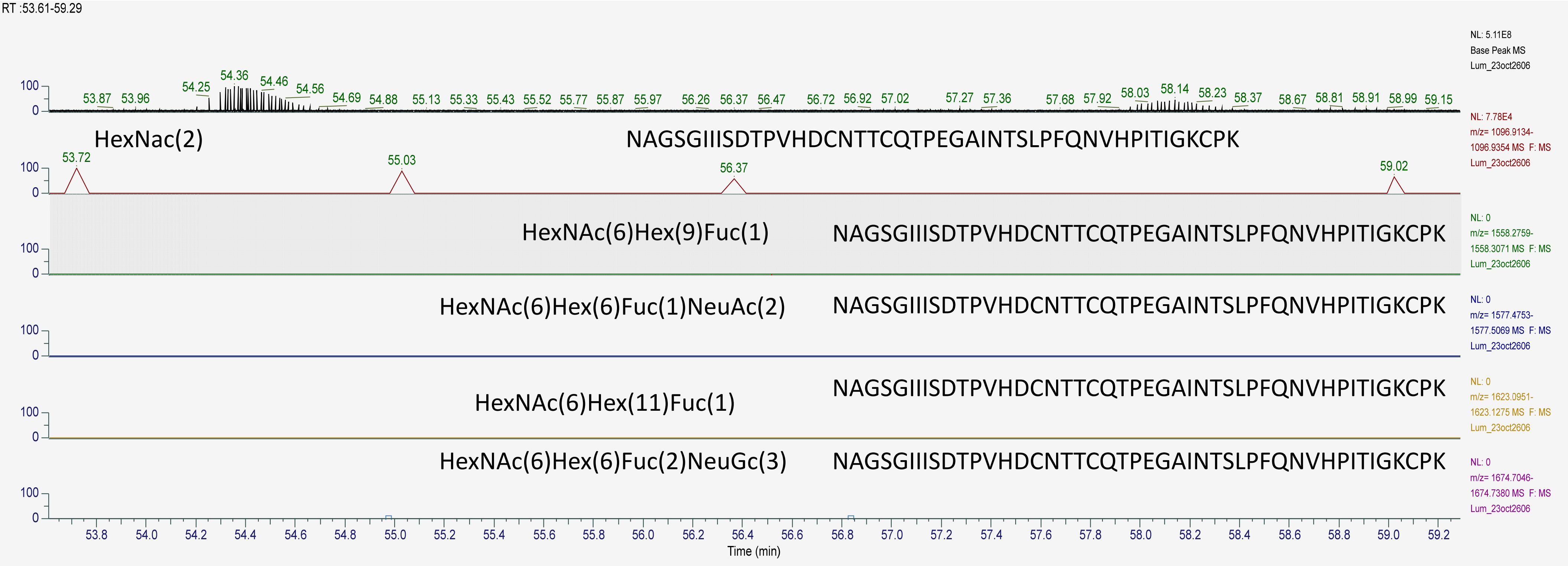

Y2-CHO-WT trypsin

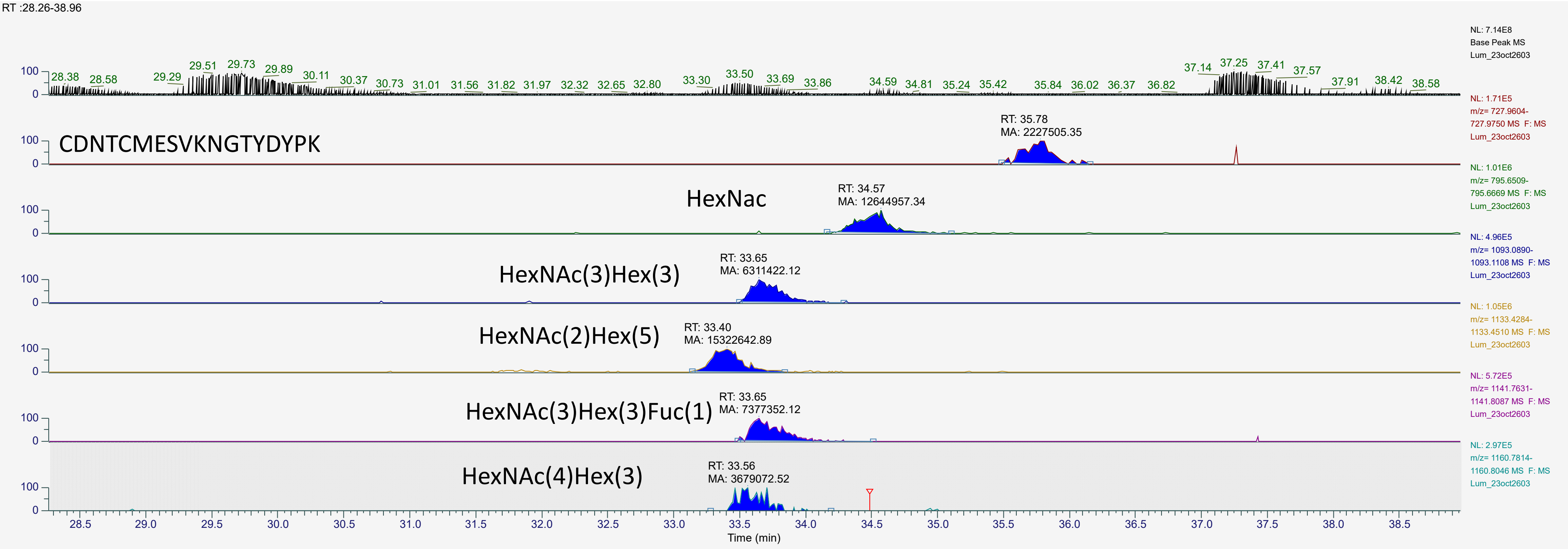

RT (Min)	Base Peak	Peak Area	Peak Height	Baseline Width	SignalToNoise
35.77796	727.968	2227505	163272.9	0.671738	INF
34.57358	795.6611	12644957	967494.1	0.941632	INF
33.64852	1093.099	6311422	471322.9	0.791261	INF
33.40177	1133.442	15322643	1023861	0.701345	INF
33.64852	1141.786	7377352	541717.6	1.031036	INF
33.56269	1160.794	3679073	297208.5	0.911148	INF

Y2-CHO-S1 trypsin

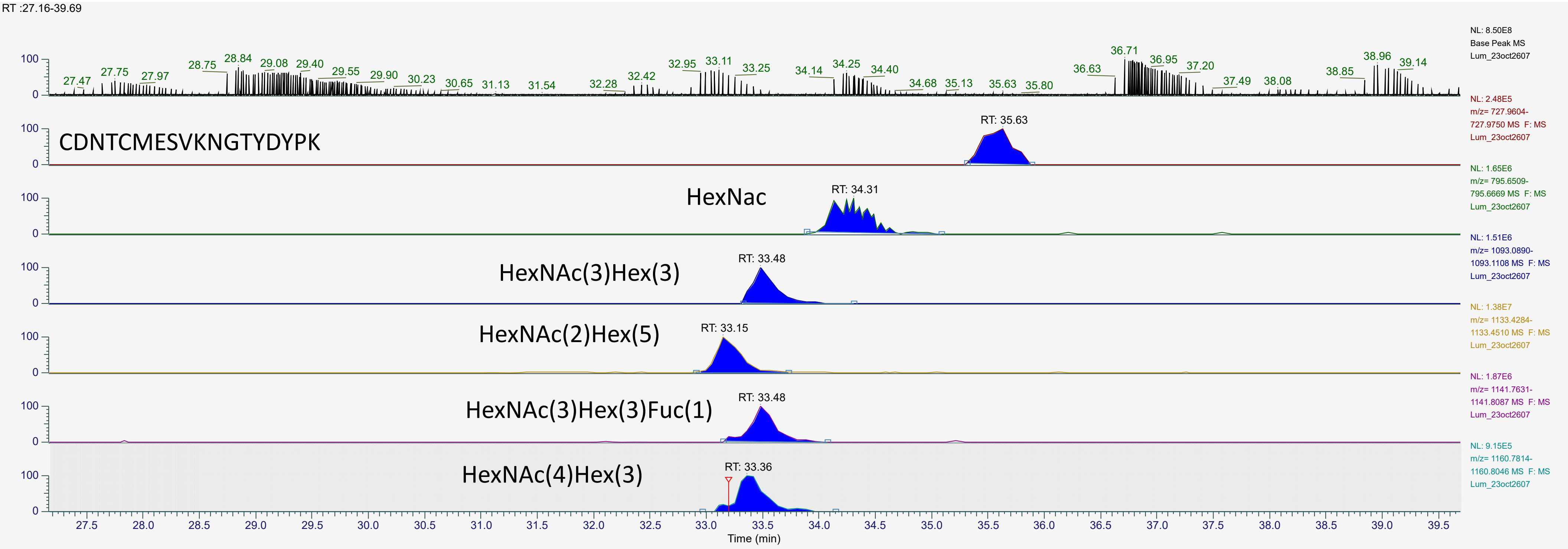

RT (Min)	Base Peak	Peak Area	Peak Height	Baseline Width	SignalToNoise
35.63084	727.9684	4388712	242484.5	0.575438	INF
34.30898	795.6617	29807384	1558929	1.192982	INF
33.48493	1093.1	20134860	1486105	0.982745	INF
33.14976	1133.443	2.02E+08	13842155	0.821294	INF
33.48493	1141.786	26103186	1820438	0.926588	INF
33.36022	1160.793	15903187	915061	1.179294	INF

Y2-CHO-S2 trypsin

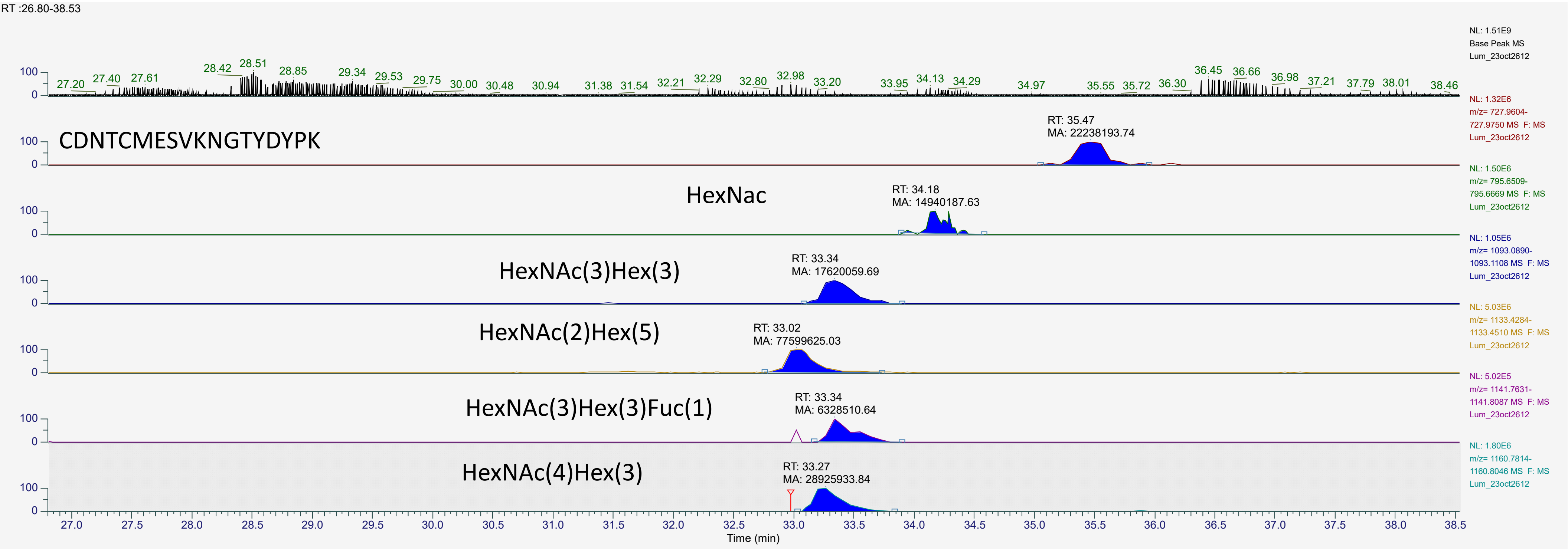

RT (Min)	Base Peak	Peak Area	Peak Height	Baseline Width	SignalToNoise
35.46839	727.9677	22238194	1305158	0.900286	INF
34.17721	795.6602	14940188	1426941	0.69	INF
33.34265	1093.1	17620060	1039382	0.814857	INF
33.02372	1133.442	77599625	4876686	0.972571	INF
33.34265	1141.786	6328511	478030.2	0.729429	INF
33.26686	1160.794	28925934	1795232	0.808286	INF

Y2-CHO-S3 trypsin

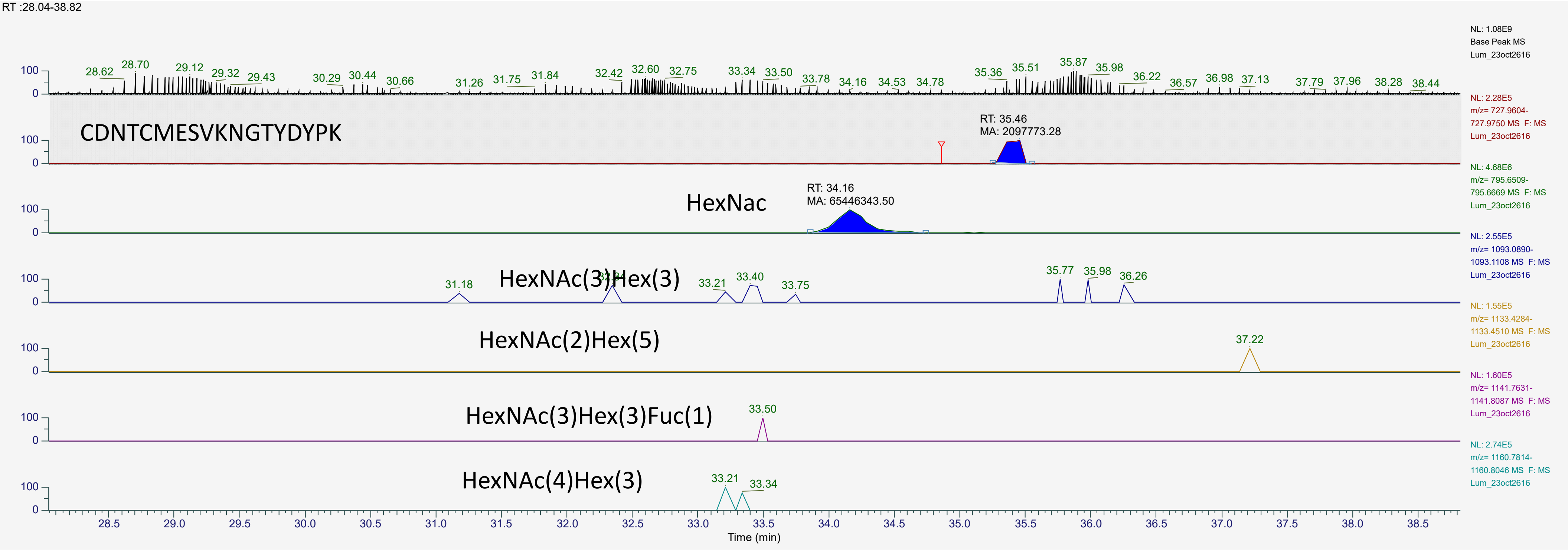

RT (Min)	Base Peak	Peak Area	Peak Height	Baseline Width	SignalToNoise
35.45881	727.9682	2097773	223203.2	0.301922	INF
34.16231	795.661	65446344	4529848	0.887651	INF

Y2-CHO-WT trypsin

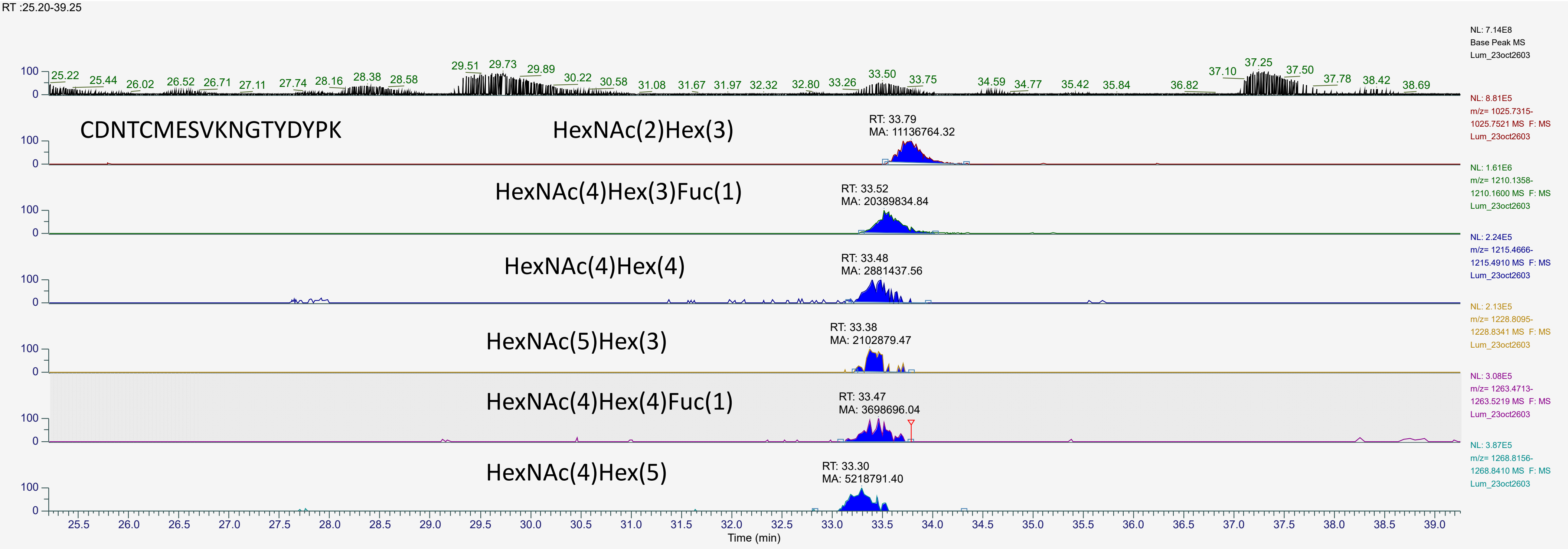

RT (Min)	Base Peak	Peak Area	Peak Height	Baseline Width	SignalToNoise
33.78875	1025.741	11136764	808755.3	0.810728	INF
33.51736	1210.148	20389835	1539862	0.732017	INF
33.4843	1215.479	2881438	216477.6	0.794986	INF
33.37746	1228.823	2102879	203434	0.566723	INF
33.46546	1263.499	3698696	305387.3	0.700532	INF
33.29669	1268.828	5218791	387153.3	1.480401	INF

Y2-CHO-S1 trypsin

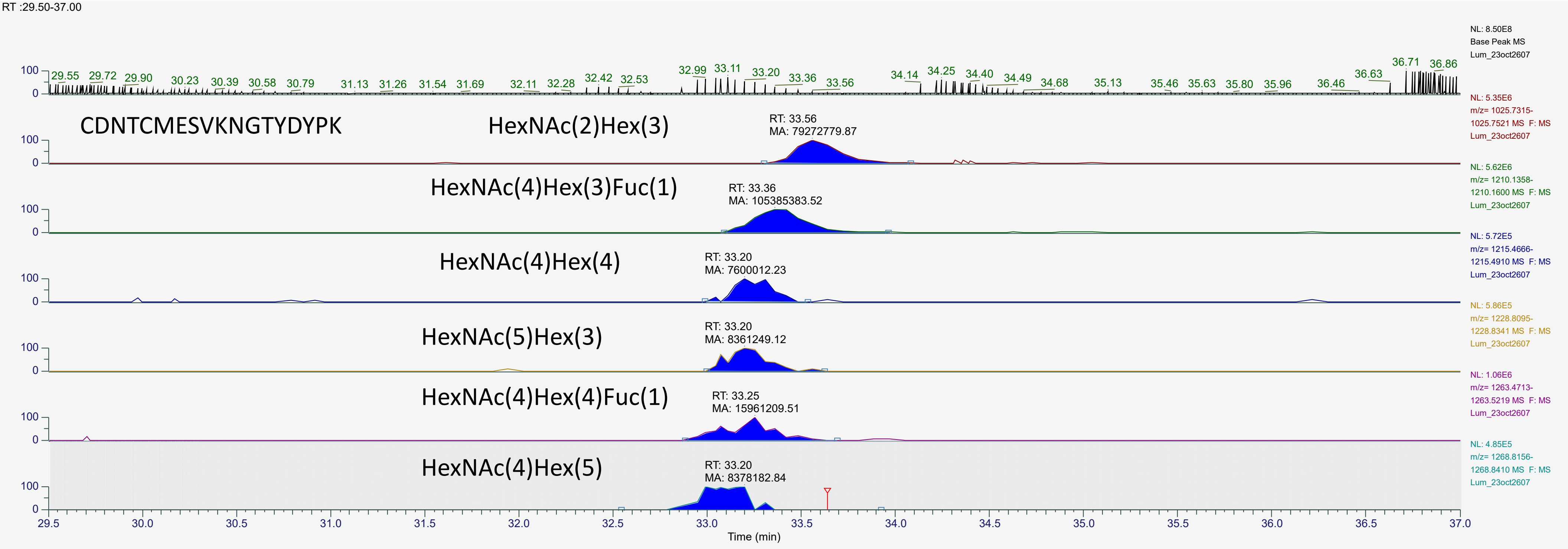

RT (Min)	Base Peak	Peak Area	Peak Height	Baseline Width	SignalToNoise
33.56012	1025.742	79272780	5234182	0.777438	INF
33.36022	1210.147	1.05E+08	5622303	0.874092	INF
33.19906	1215.479	7600012	557205.6	0.546218	INF
33.19906	1228.818	8361249	578196.9	0.62605	INF
33.25265	1263.495	15961210	1060097	0.806723	INF
33.19906	1268.831	8378183	485105.2	1.382353	INF

Y2-CHO-S2 trypsin

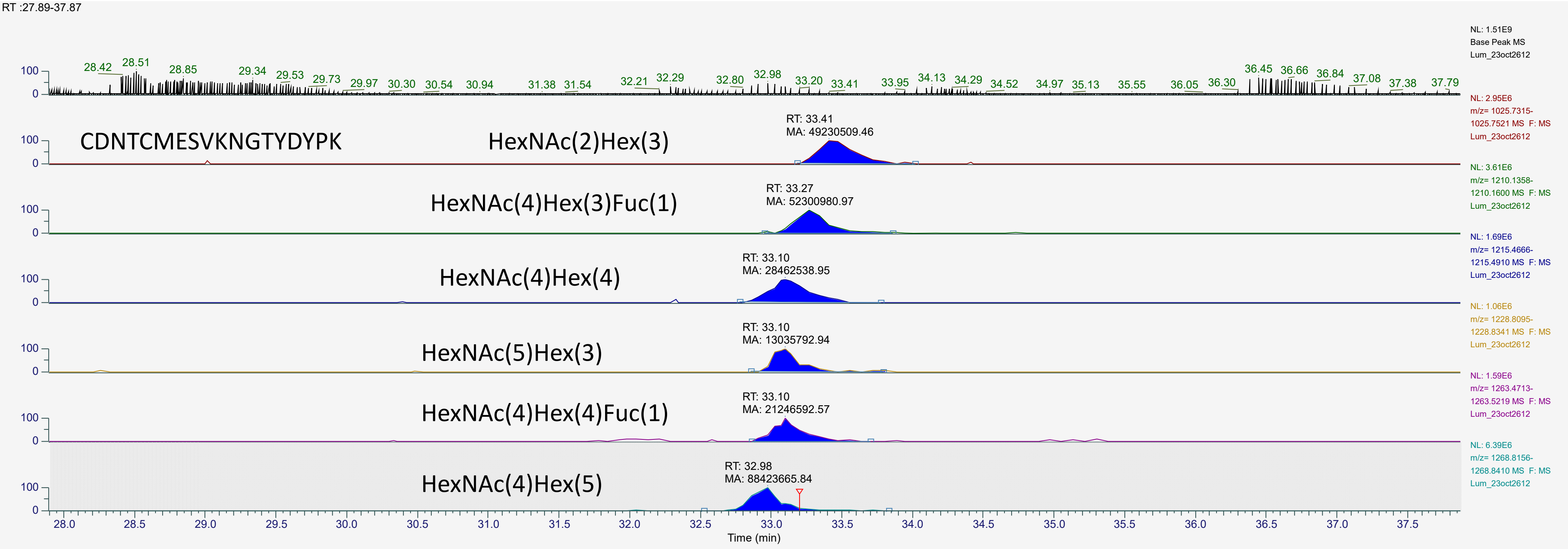

RT (Min)	Base Peak	Peak Area	Peak Height	Baseline Width	SignalToNoise
33.40744	1025.741	49230509	2851540	0.832849	INF
33.26686	1210.148	52300981	3563772	0.911103	INF
33.10176	1215.478	28462539	1615575	1.000796	INF
33.10176	1228.819	13035793	1024753	0.939294	INF
33.10176	1263.496	21246593	1593294	0.844246	INF
32.97776	1268.828	88423666	6394489	1.308303	INF

Y2-CHO-S3 trypsin

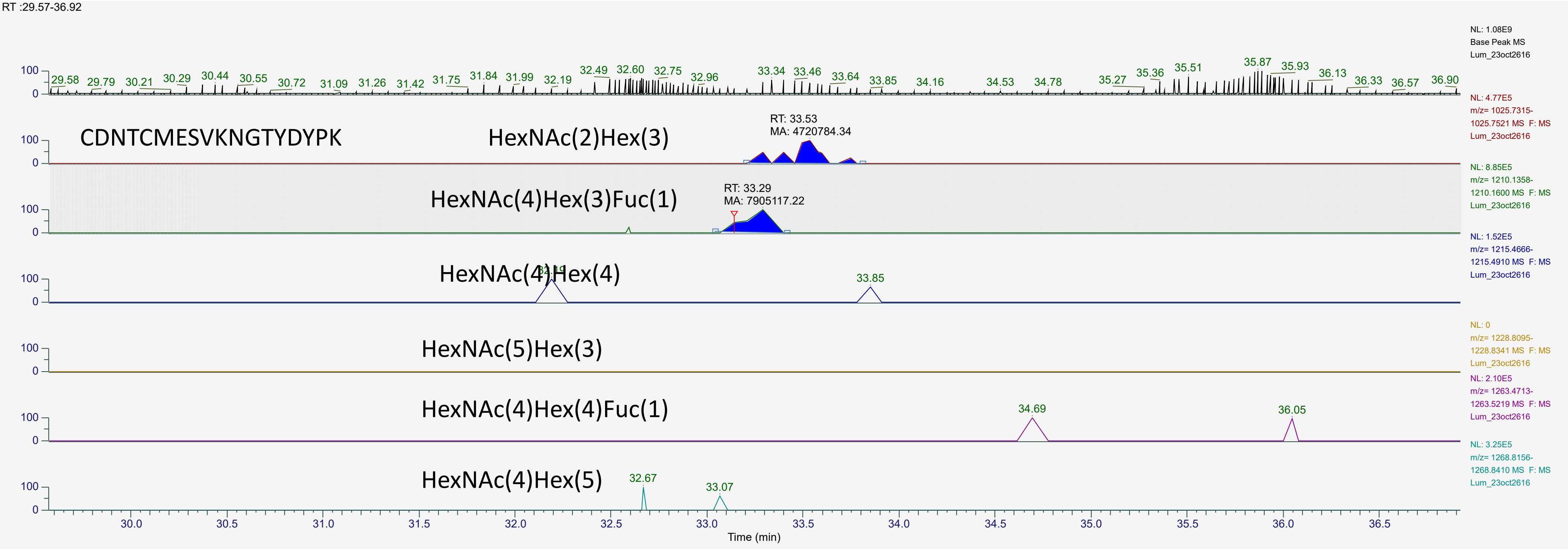

RT (Min)	Base Peak	Peak Area	Peak Height	Baseline Width	SignalToNoise
33.53393	1025.741	4720784	466419.6	0.605761	INF
33.29185	1210.144	7905117	860680.1	0.370874	INF

Y2-CHO-WT trypsin

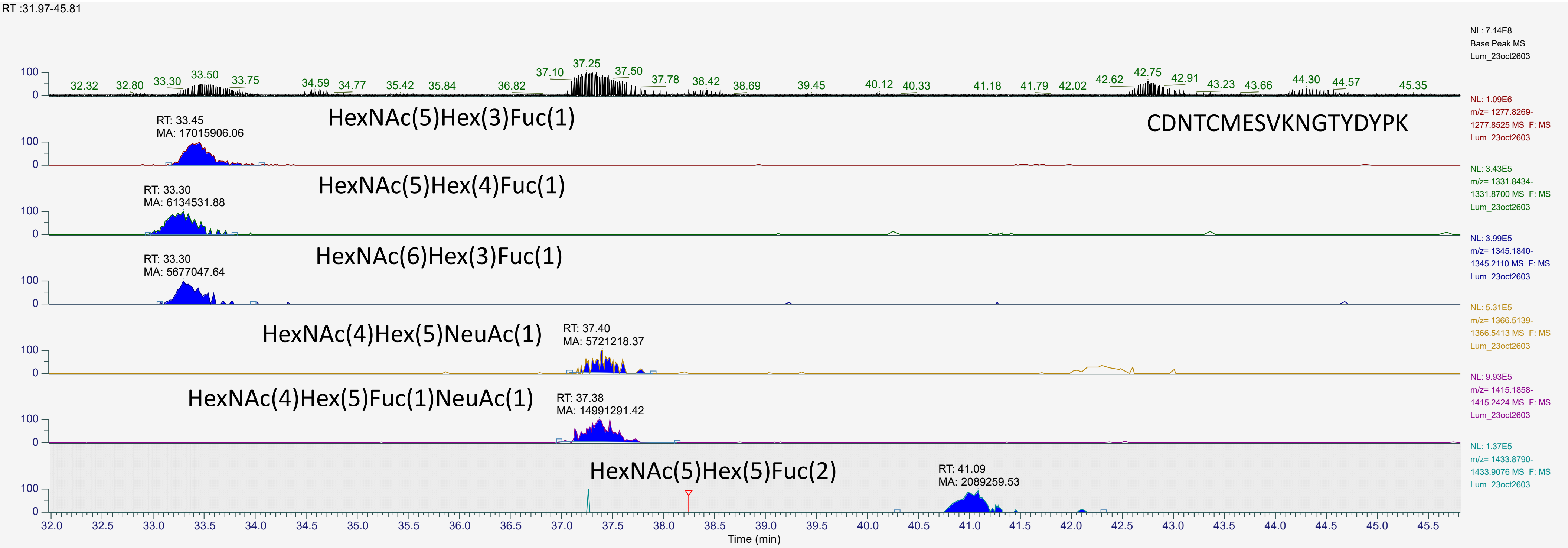

RT (Min)	Base Peak	Peak Area	Peak Height	Baseline Width	SignalToNoise
33.44987	1277.841	17015906	1078041	0.914745	INF
33.29669	1331.857	6134532	342716.2	0.852728	INF
33.29669	1345.2	5677048	399397.3	0.914913	INF
37.40259	1366.53	5721218	511247.5	0.821871	INF
37.3764	1415.217	14991291	938669.8	1.155272	INF
41.08598	1433.884	2089260	124260.5	2.023664	INF

Y2-CHO-S1 trypsin

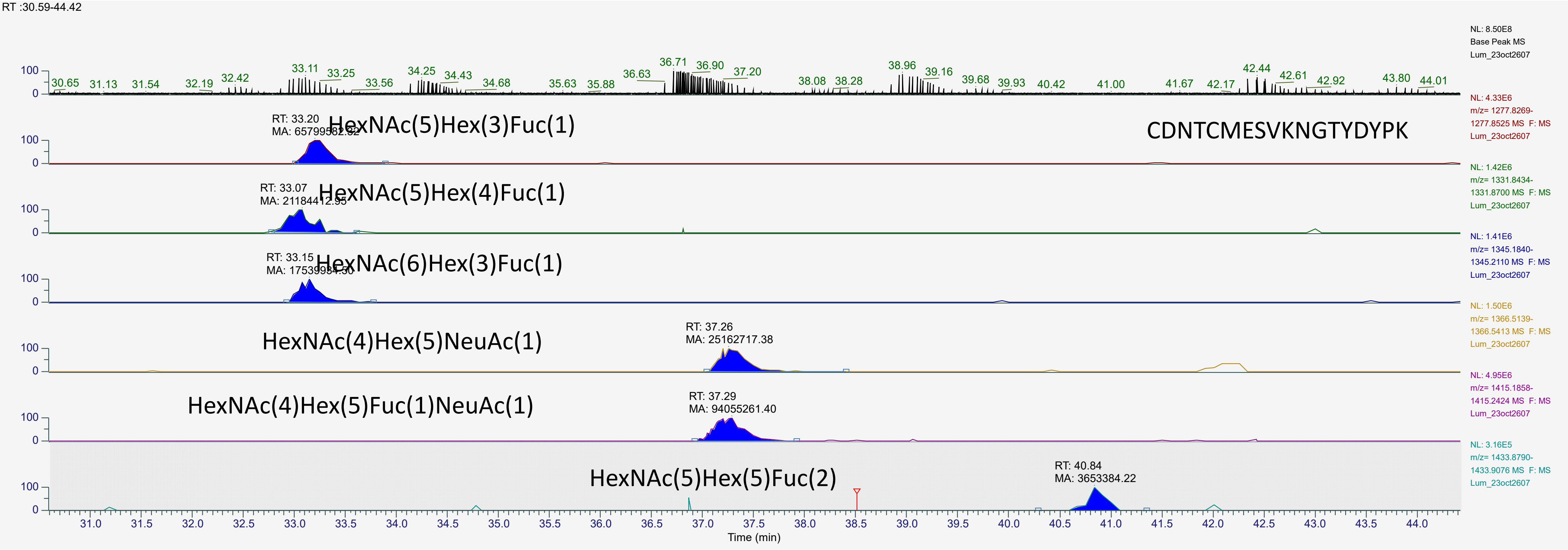

RT (Min)	Base Peak	Peak Area	Peak Height	Baseline Width	SignalToNoise
33.19906	1277.841	65799582	4274922	0.883721	INF
33.07407	1331.856	21184413	1367188	0.837209	INF
33.14976	1345.199	17539934	1392311	0.852269	INF
37.25698	1366.53	25162717	1475947	1.36363	INF
37.28883	1415.215	94055261	4948017	0.999479	INF
40.83749	1433.884	3653384	315901	1.061462	INF

Y2-CHO-S2 trypsin

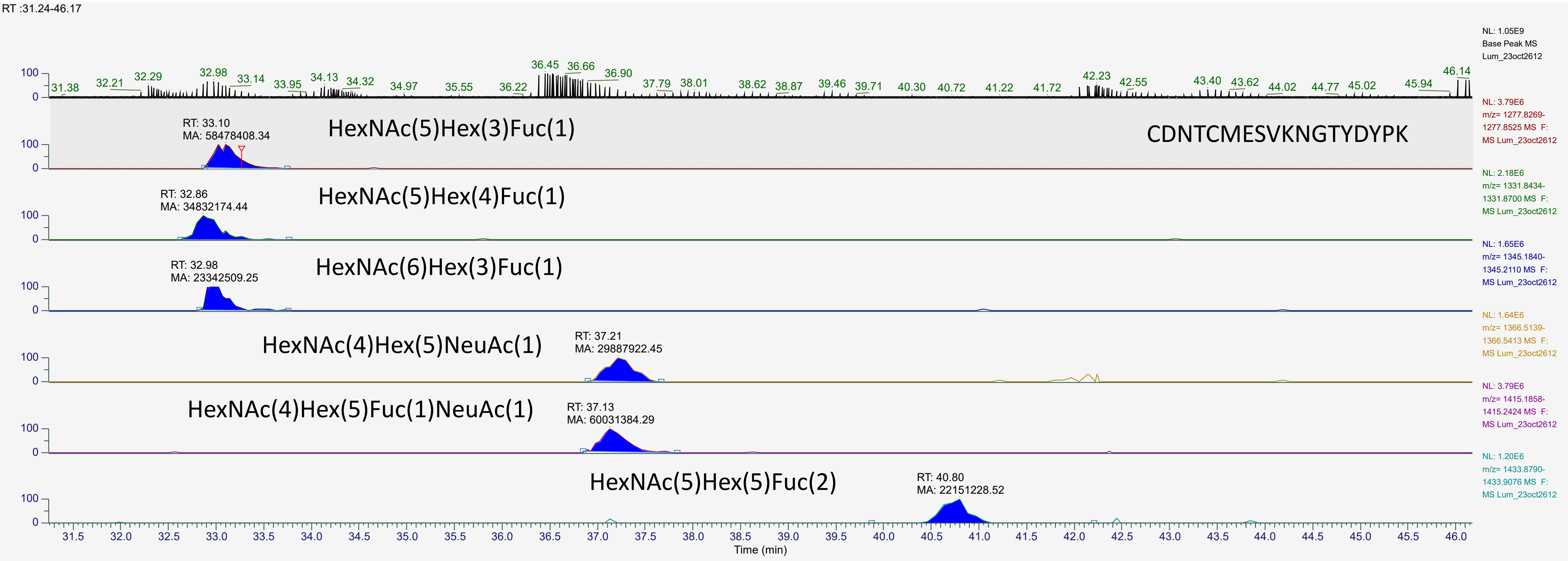

RT (Min)	Base Peak	Peak Area	Peak Height	Baseline Width	SignalToNoise
33.10176	1277.84	58478408	3613834	0.869871	INF
32.86299	1331.858	34832174	2139614	1.137524	INF
32.97776	1345.2	23342509	1592289	0.92842	INF
37.2092	1366.528	29887922	1574685	0.769487	INF
37.1258	1415.214	60031384	3564090	0.98695	INF
40.79904	1433.883	22151229	1204927	2.327294	INF

Y2-CHO-S3 trypsin

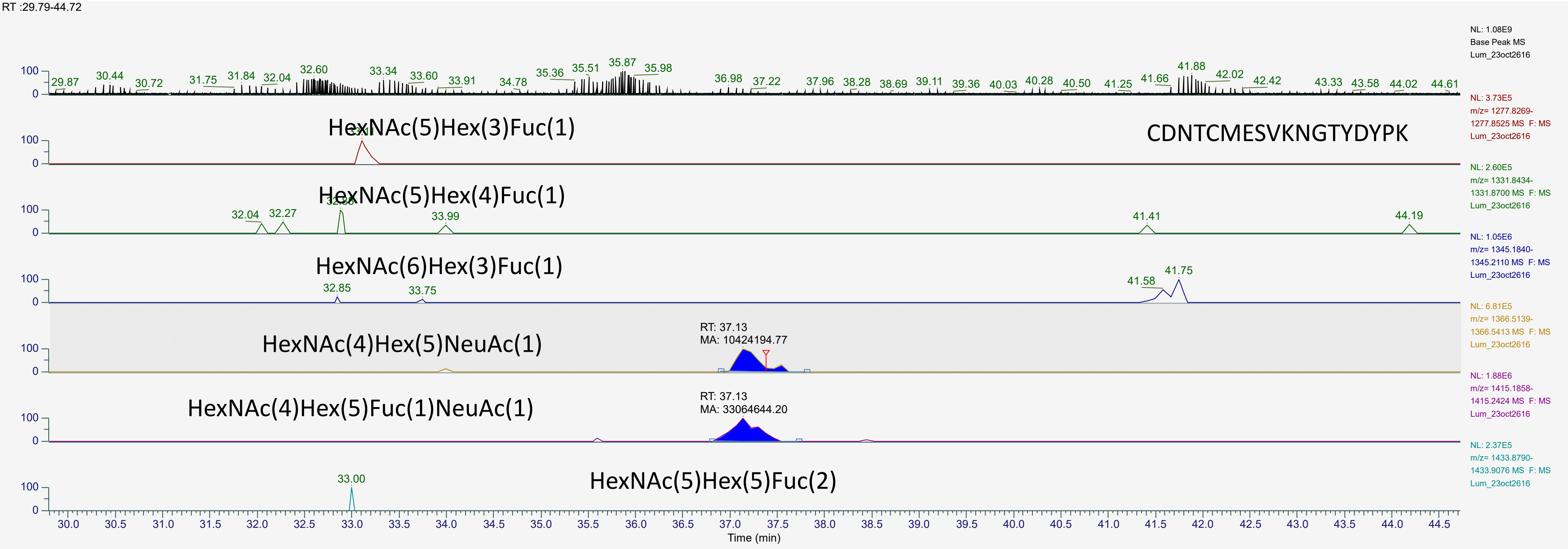

RT (Min)	Base Peak	Peak Area	Peak Height	Baseline Width	SignalToNoise
37.13432	1366.528	10424195	649685.8	0.903318	INF
37.13432	1415.215	33064644	1840980	0.920046	INF

Y2-CHO-WT trypsin

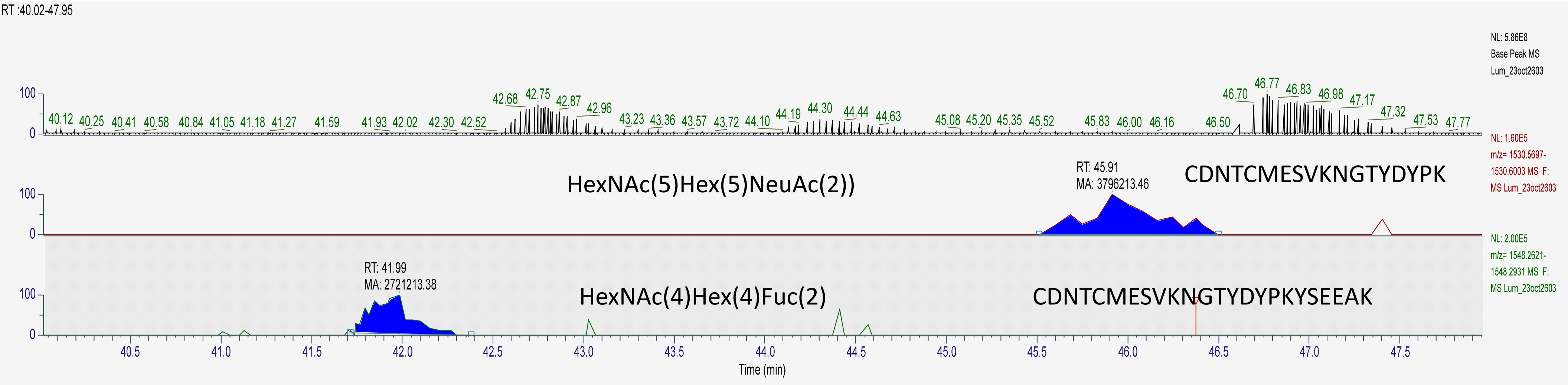

RT (Min)	Base Peak	Peak Area	Peak Height	Baseline Width	SignalToNoise
45.9137	1530.587	3796213	158198.5	0.985349	INF
41.98551	1548.277	2721213	189806.5	0.66863	INF

Y2-CHO-S1 trypsin

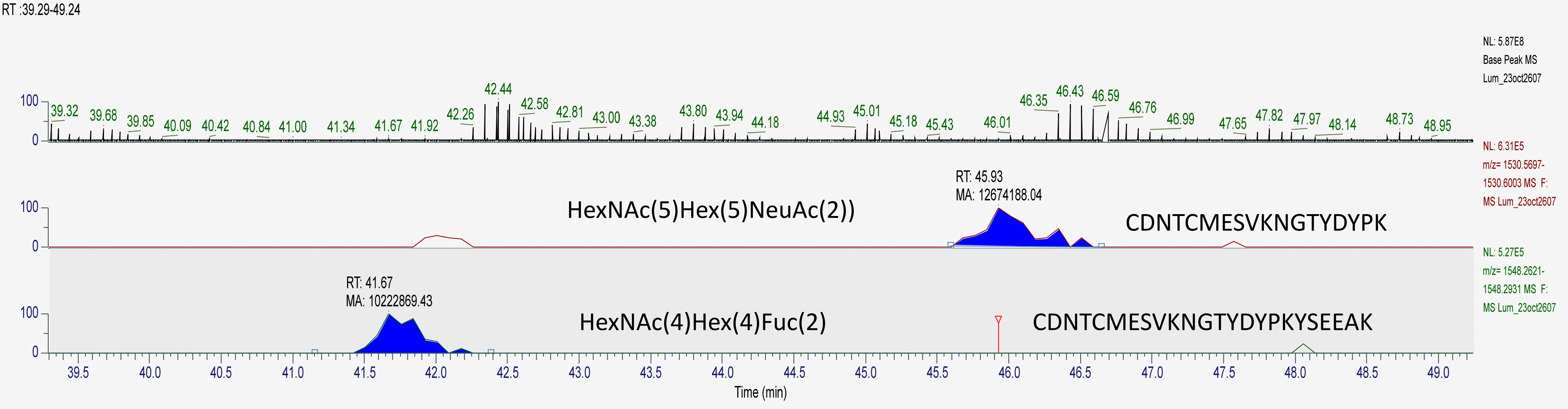

RT (Min)	Base Peak	Peak Area	Peak Height	Baseline Width	SignalToNoise
45.93005	1530.59	12674188	605831.3	1.057445	INF
41.67269	1548.278	10222869	526517.7	1.227294	INF

Y2-CHO-S2 trypsin

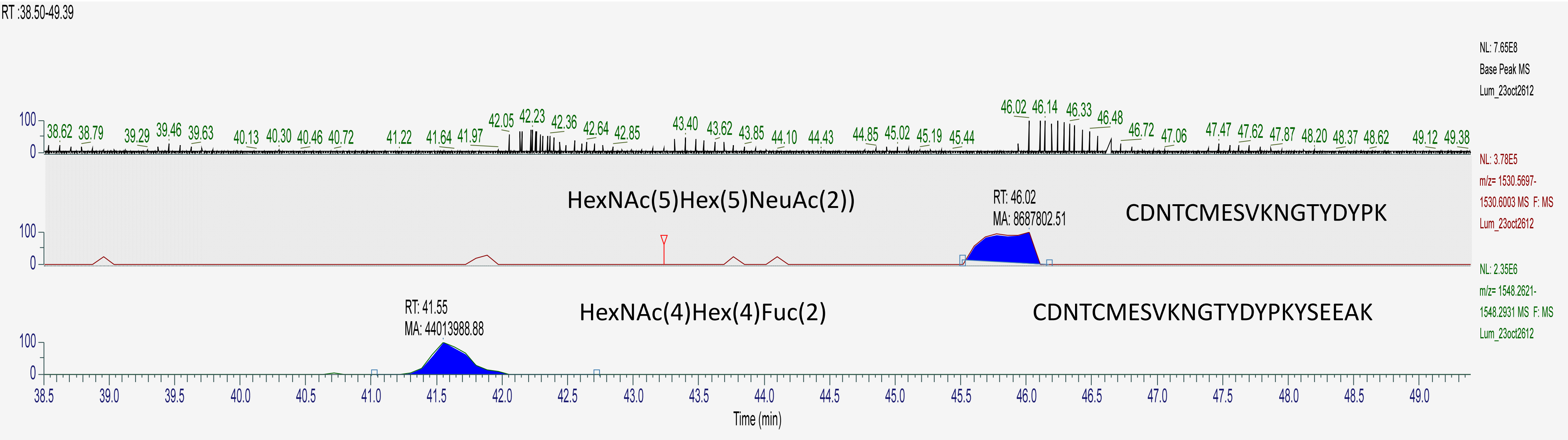

RT (Min)	Base Peak	Peak Area	Peak Height	Baseline Width	SignalToNoise
46.02319	1530.578	8687803	364898.6	0.664393	INF
41.55206	1548.278	44013989	2353818	1.697221	INF

Y2-CHO-S3 trypsin

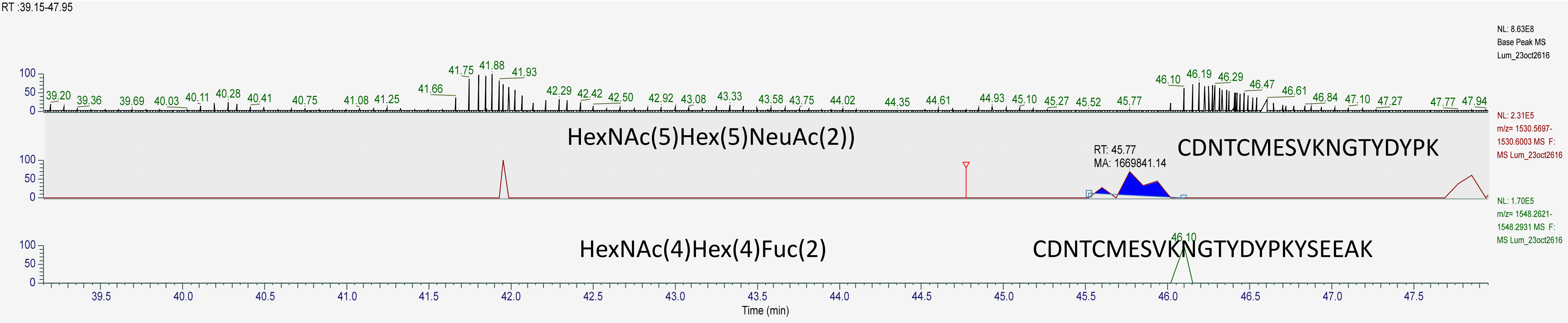

RT (Min)	Base Peak	Peak Area	Peak Height	Baseline Width	SignalToNoise
45.76764	1530.588	1669841	143987.8	0.575691	INF

Y2-CHO-WT trypsin

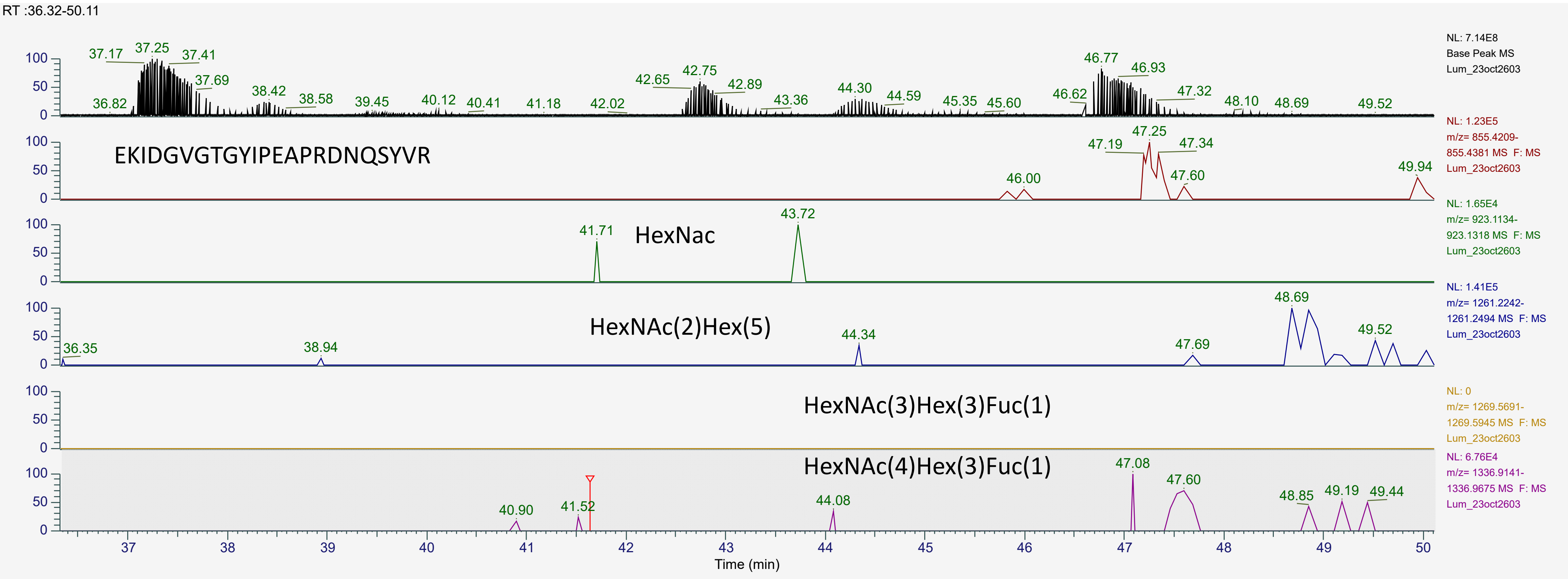

Y2-CHO-S1 trypsin

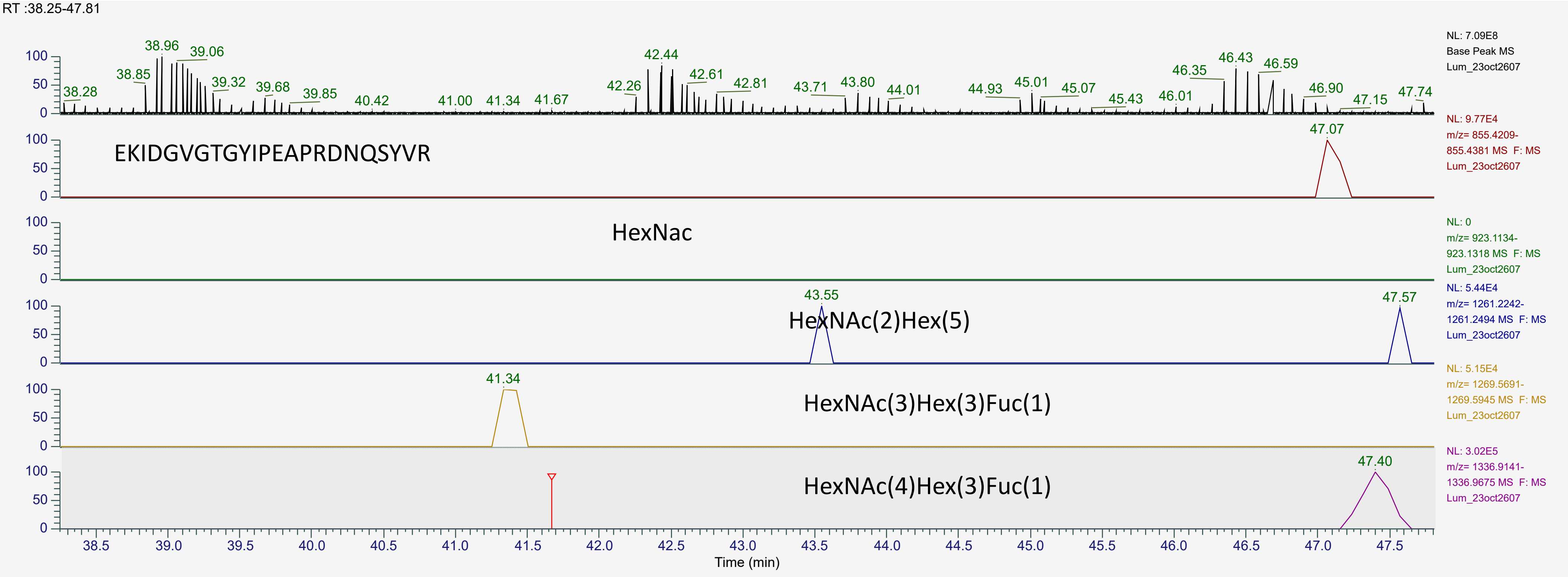

Y2-CHO-S2 trypsin

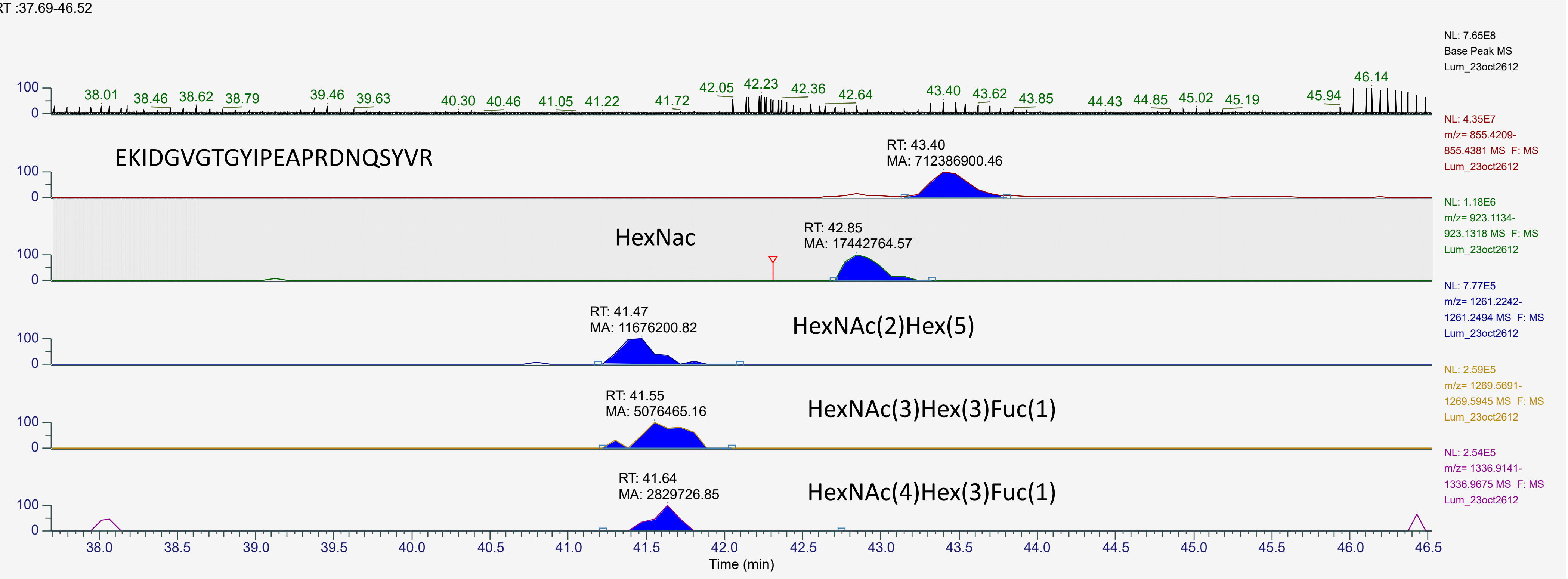

RT (Min)	Base Peak	Peak Area	Peak Height	Baseline Width	SignalToNoise
43.40019	855.4301	7.12E+08	42590473	0.655387	INF
42.84838	923.123	17442765	1181348	0.630714	INF
41.46845	1261.239	11676201	759401.2	0.905481	INF
41.55206	1269.582	5076465	259226.8	0.8243	INF
41.63527	1336.939	2829727	254251.7	1.52995	INF

Y2-CHO-S3 trypsin

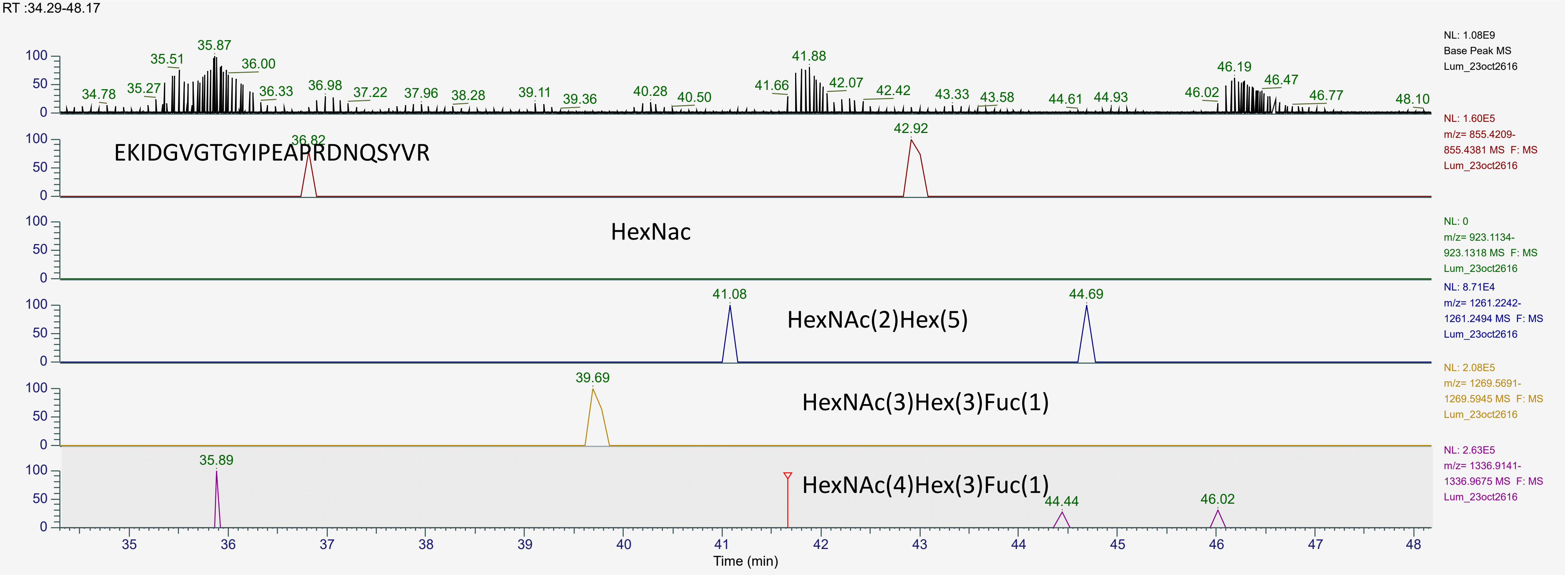

Y2-CHO-WT trypsin

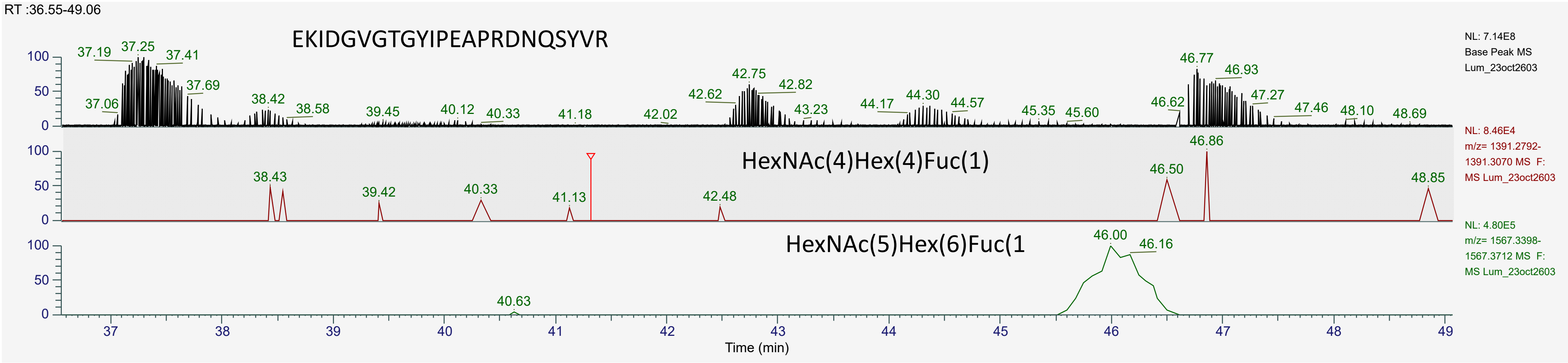

Y2-CHO-S1 trypsin

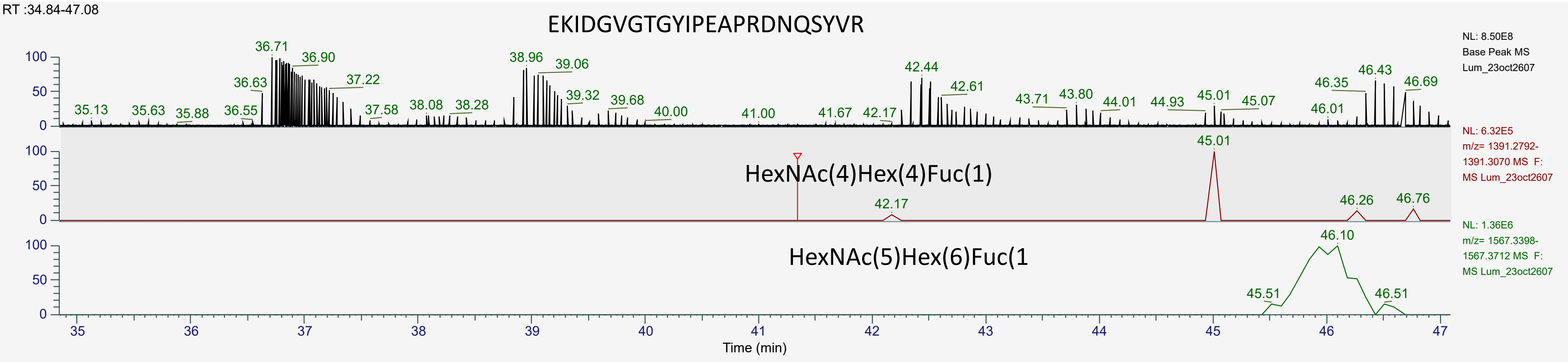

Y2-CHO-S2 trypsin

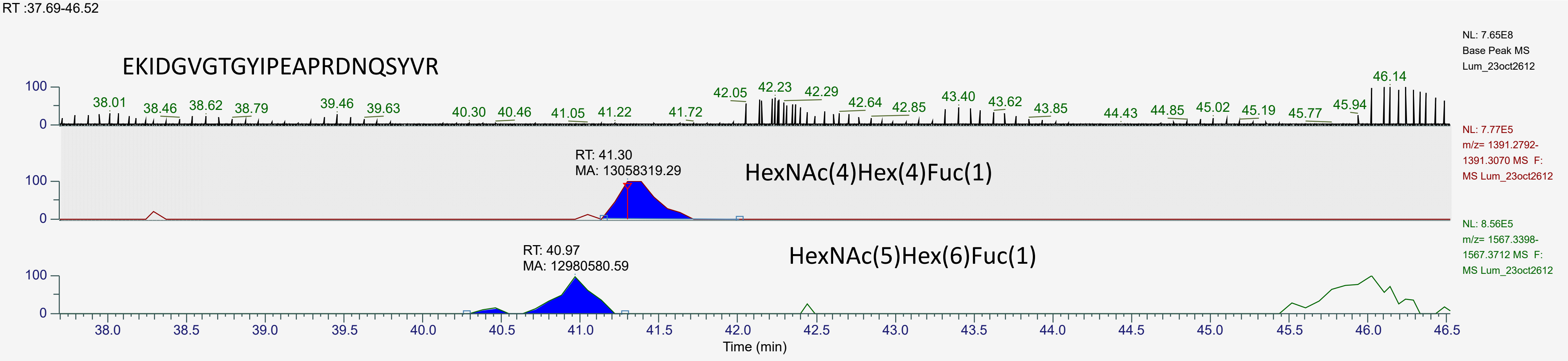

RT (Min)	Base Peak	Peak Area	Peak Height	Baseline Width	SignalToNoise
41.30058	1391.293	13058319	760025.2	0.863268	INF
40.96667	1567.355	12980581	814537.6	1.005091	INF

Y2-CHO-WT trypsin

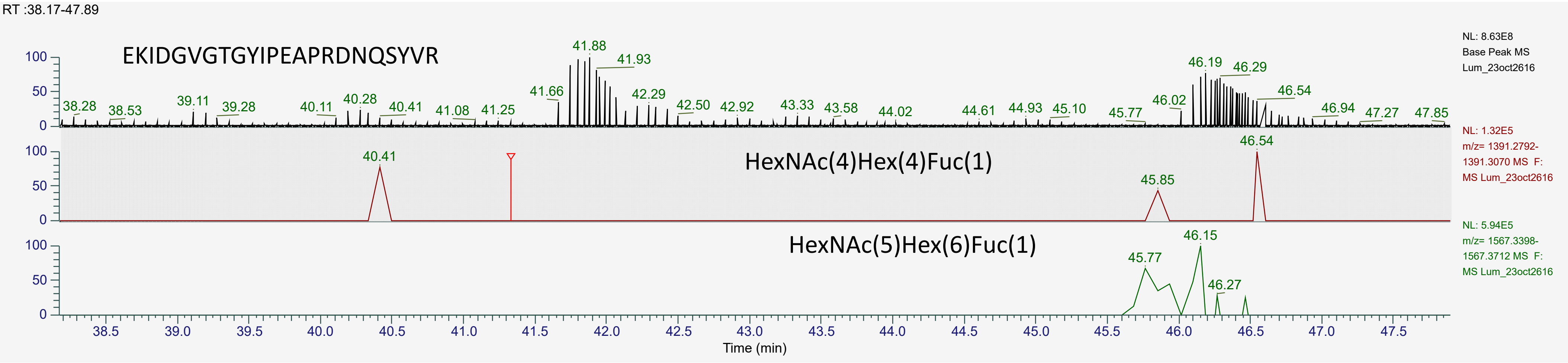

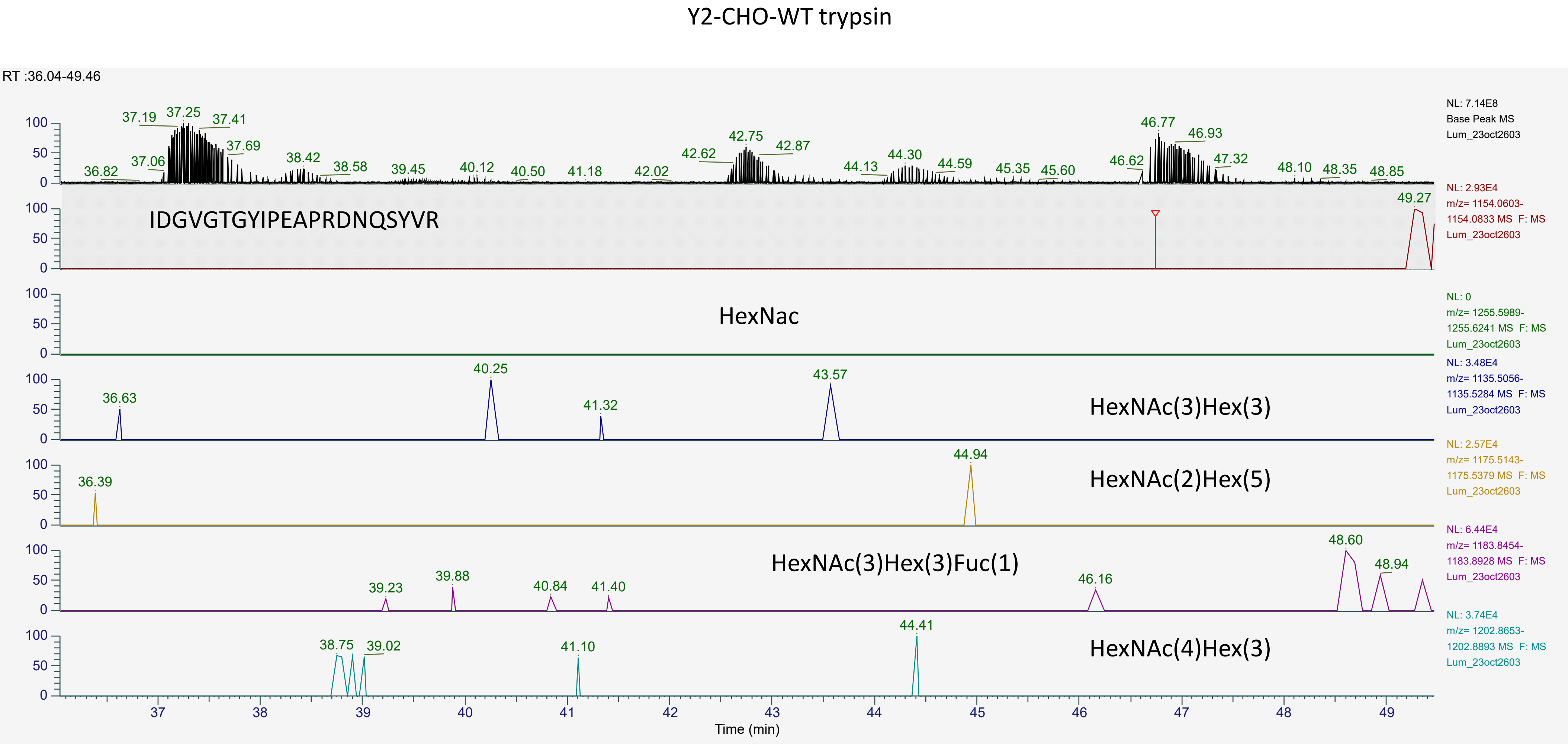
Y2-CHO-WT trypsin
RT :36.04-49.46
NL: 7.14E8
Base Peak MS
Lum_23oct2603
IDGVGTGYIPEAPRDNQSYVR
NL: 2.93E4
m/z= 1154.0603-1154.0833 MS F: MS
Lum_23oct2603
HexNac
NL: 0
m/z= 1255.5989-1255.6241 MS F: MS
Lum_23oct2603
HexNAc(3)Hex(3)
NL: 3.48E4
m/z= 1135.5056-1135.5284 MS F: MS
Lum_23oct2603
HexNAc(2)Hex(5)
NL: 2.57E4
m/z= 1175.5143-1175.5379 MS F: MS
Lum_23oct2603
HexNAc(3)Hex(3)Fuc(1)
NL: 6.44E4
m/z= 1183.8454-1183.8928 MS F: MS
Lum_23oct2603
HexNAc(4)Hex(3)
NL: 3.74E4
m/z= 1202.8653-1202.8893 MS F: MS
Lum_23oct2603
Time (min)

Y2-CHO-S1 trypsin

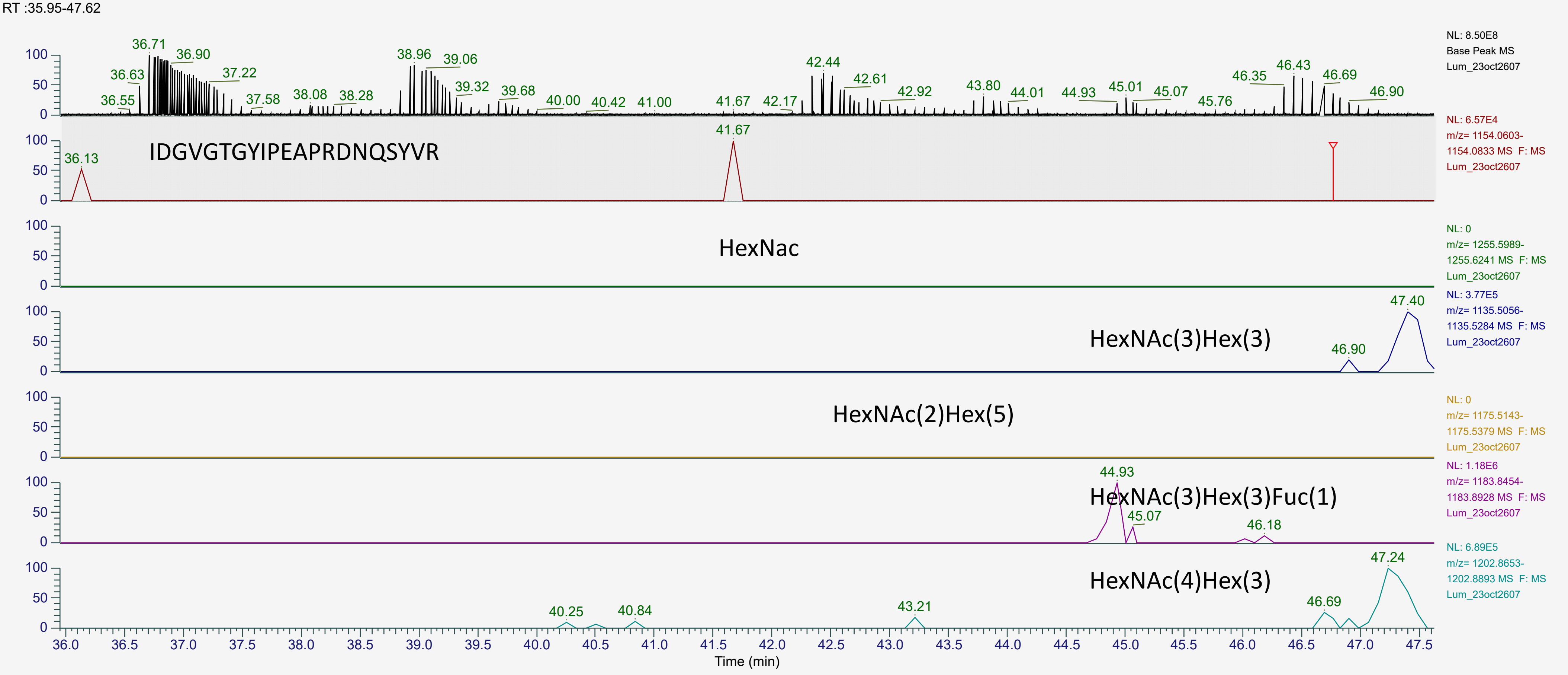

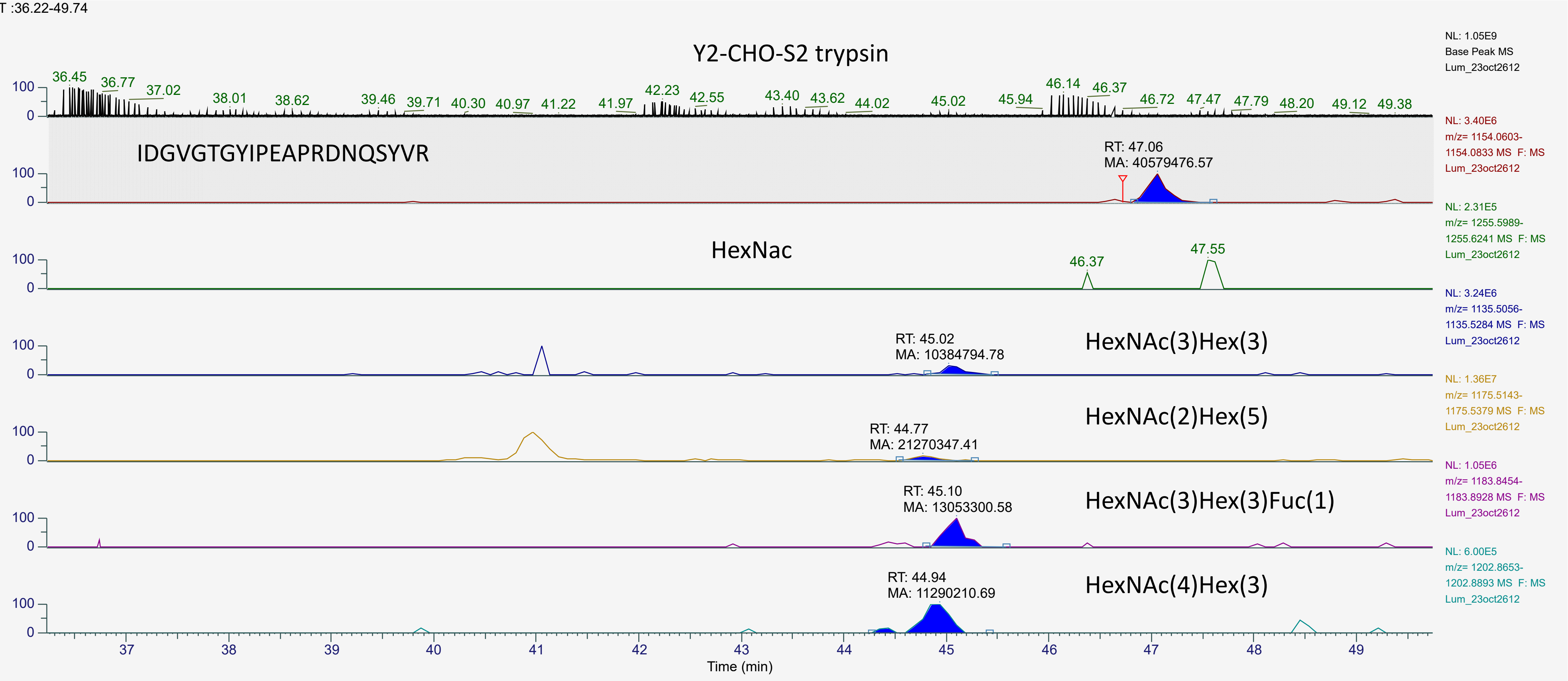

RT (Min)	Base Peak	Peak Area	Peak Height	Baseline Width	SignalToNoise
47.05603	1154.071	40579477	3326421	0.774484	INF
45.01869	1135.517	10384795	801593.9	0.659483	INF
44.76847	1175.525	21270347	1570783	0.736238	INF
45.10248	1183.871	13053301	1027491	0.784045	INF
44.93577	1202.877	11290211	594847.6	1.146927	INF

Y2-CHO-S3 trypsin

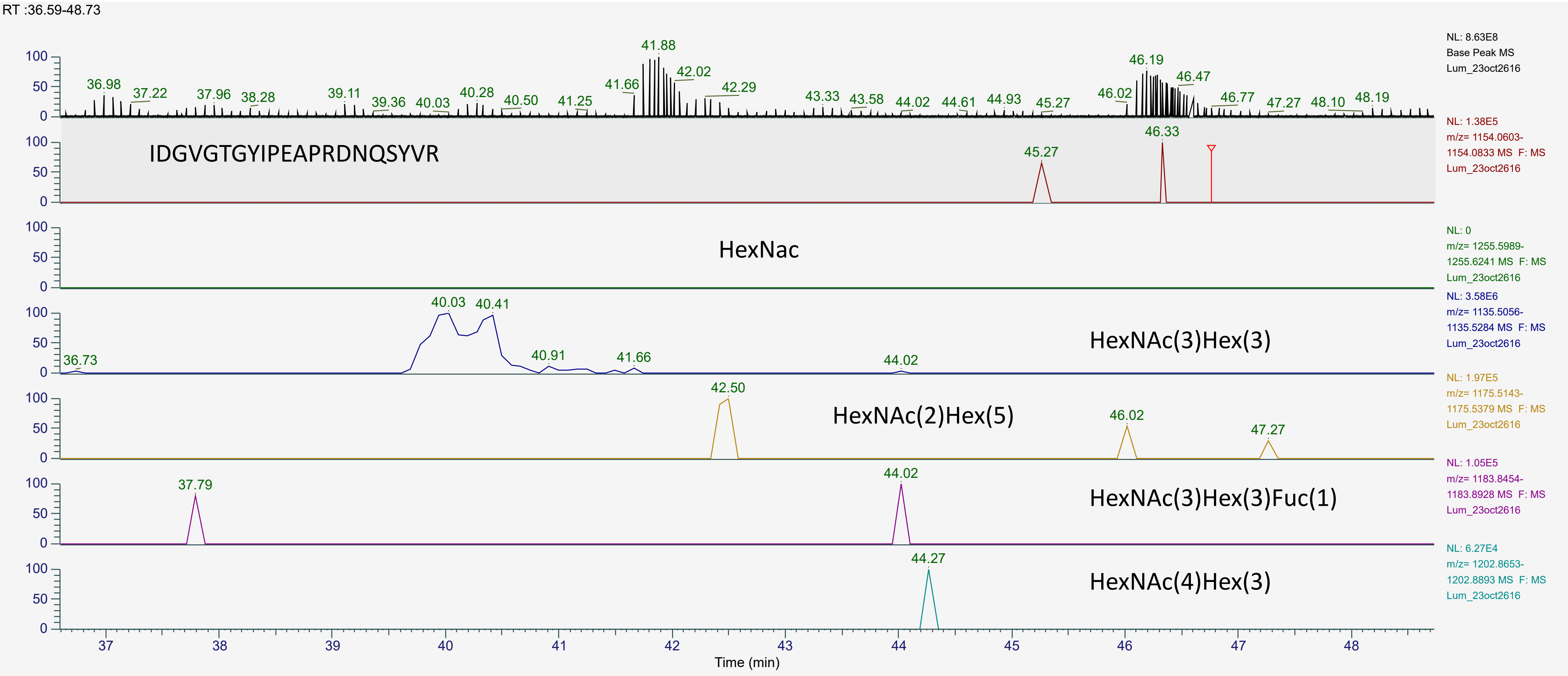

Y2-CHO-WT trypsin

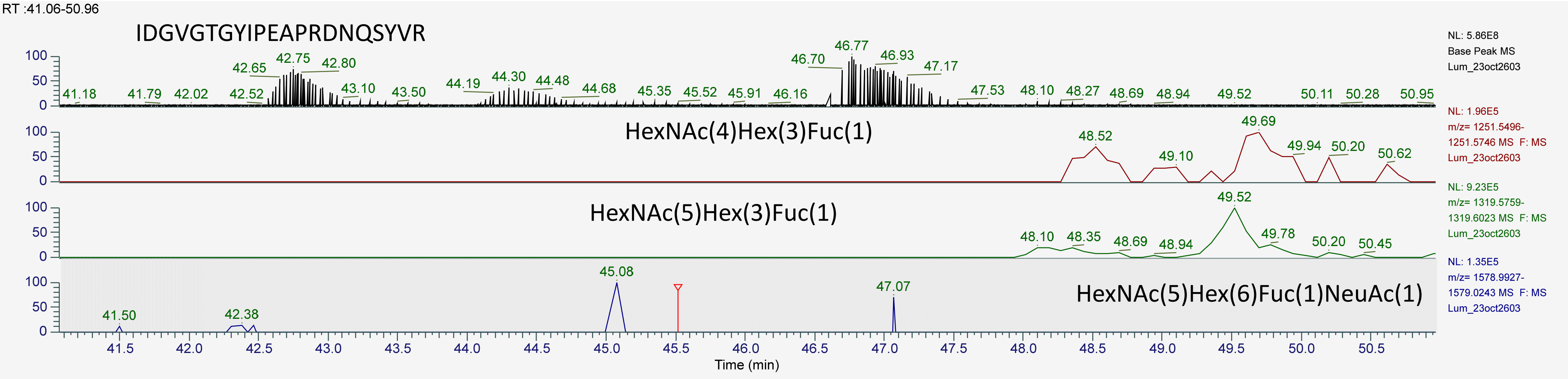

Y2-CHO-S1 trypsin

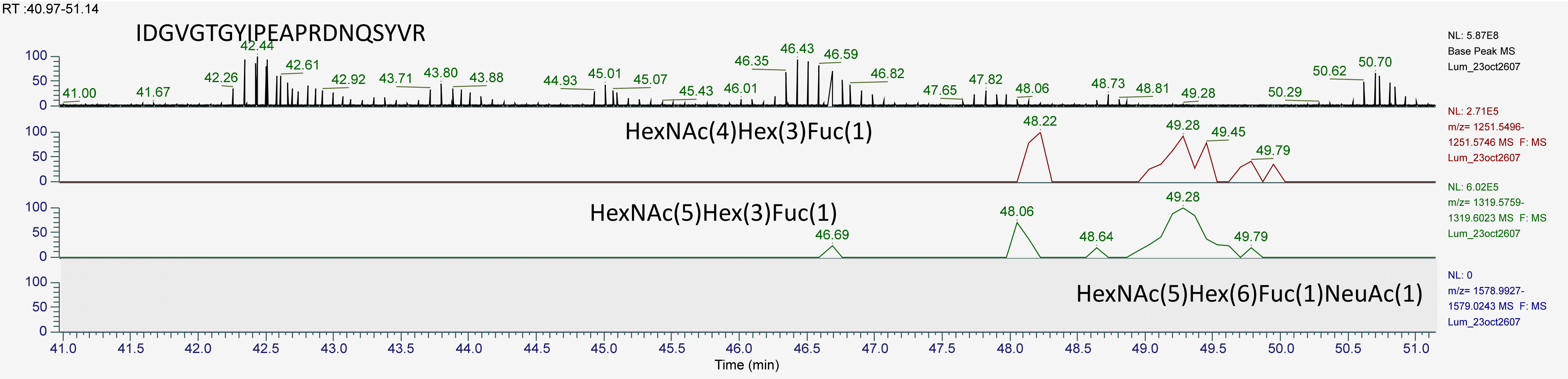

Y2-CHO-S2 trypsin

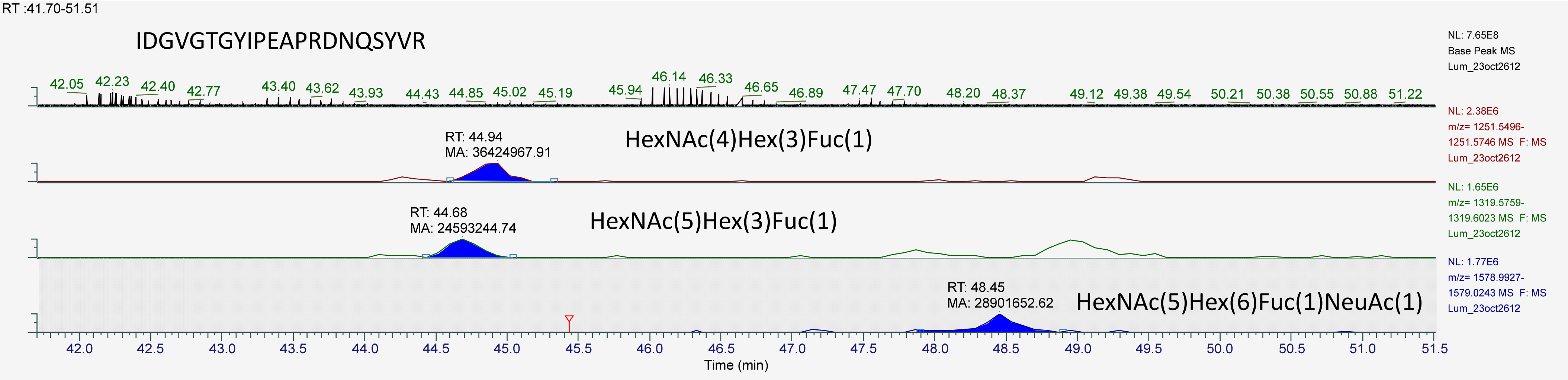

RT (Min)	Base Peak	Peak Area	Peak Height	Baseline Width	SignalToNoise
44.93577	1251.562	36424968	2315527	0.726667	INF
44.68449	1319.589	24593245	1628238	0.613968	INF
48.45121	1579.009	28901653	1769676	1.000881	INF

Y2-CHO-S3 trypsin

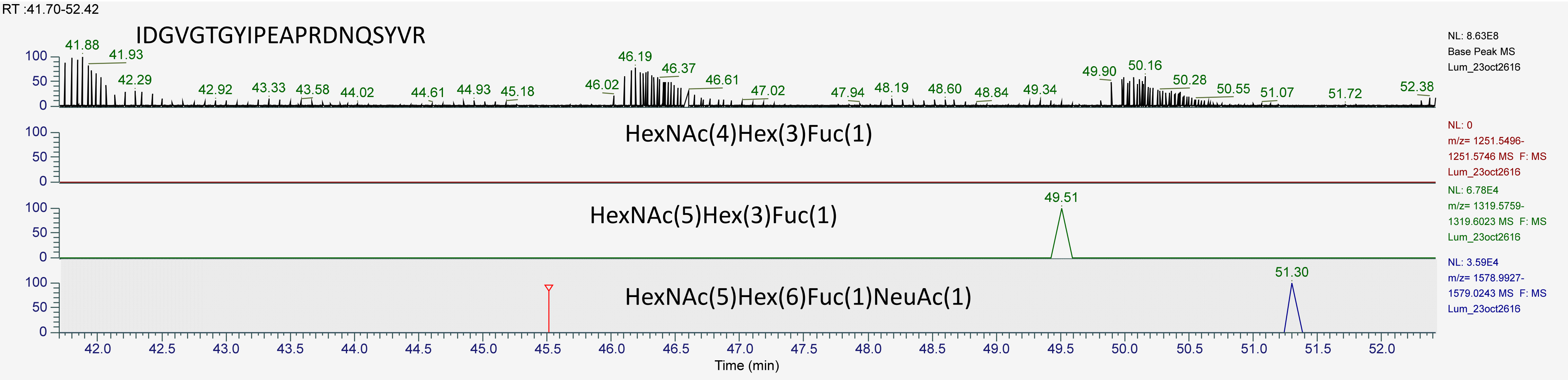

Y2-CHO-S1 trypsin

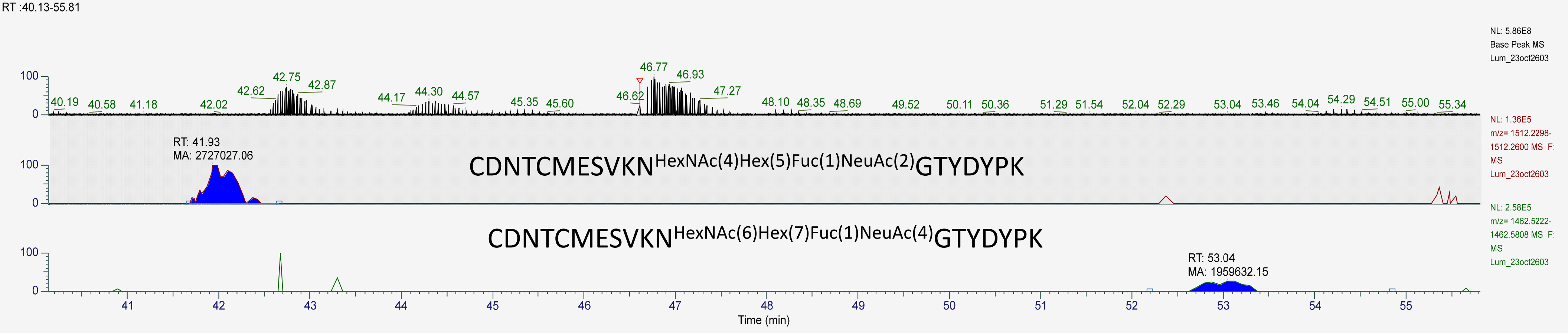

RT (Min)	Base Peak	Peak Area	Peak Height	Baseline Width	SignalToNoise
41.93164	1512.25	2727027	134464.7	0.9843	INF
53.04268	1462.553	1959632	68037.84	2.659338	INF

Y2-CHO-S1 trypsin

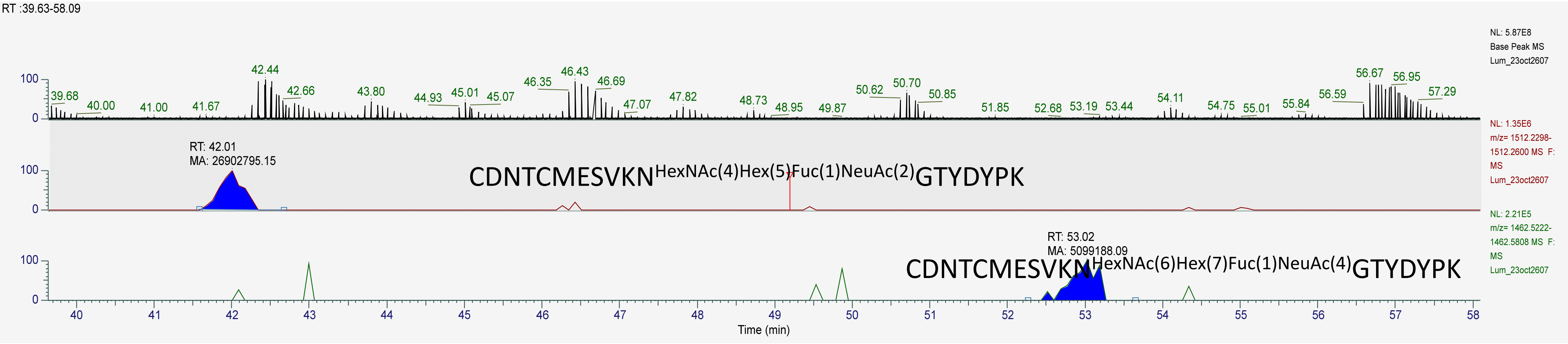

RT (Min)	Base Peak	Peak Area	Peak Height	Baseline Width	SignalToNoise
42.00793	1512.245	26902795	1323440	1.085221	INF
53.0197	1462.551	5099188	220961	1.382121	INF

Y2-CHO-S2 trypsin

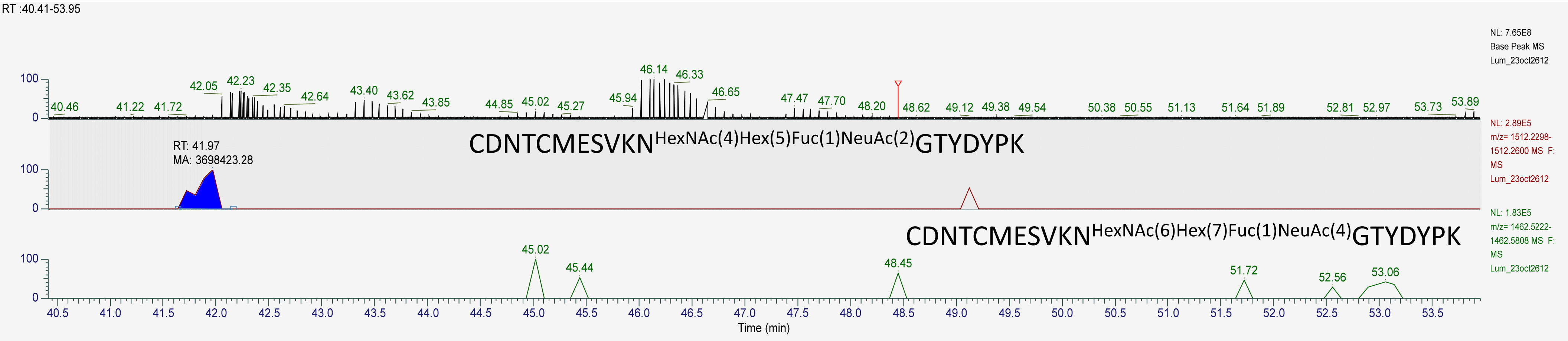

RT (Min)	Base Peak	Peak Area	Peak Height	Baseline Width	SignalToNoise
41.97042	1512.244	3698423	287796	0.521972	INF

Y2-CHO-S3 trypsin

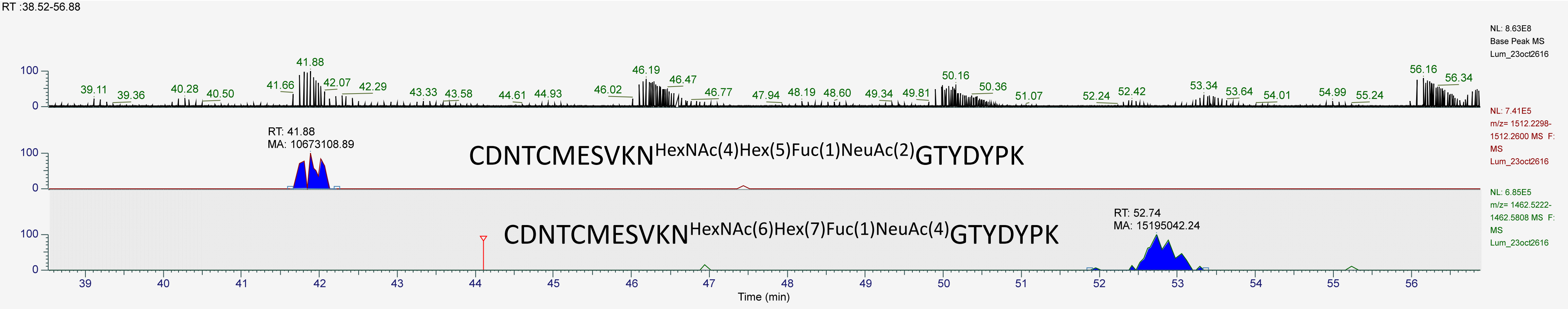

RT (Min)	Base Peak	Peak Area	Peak Height	Baseline Width	SignalToNoise
41.88319	1512.244	10673109	732780.4	0.593231	INF
52.73756	1462.552	15195042	685242.6	1.492439	INF

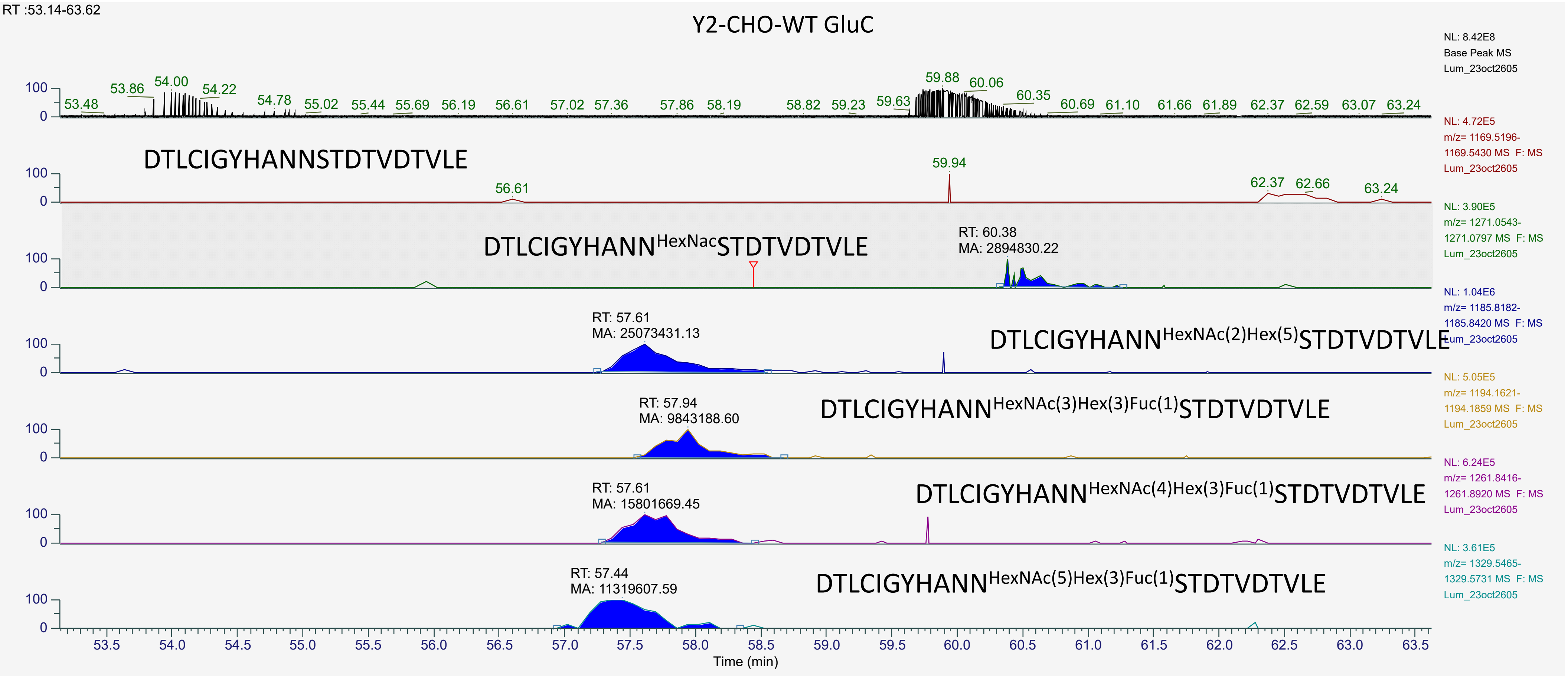

RT (Min)	Base Peak	Peak Area	Peak Height	Baseline Width	SignalToNoise
60.38498	1271.067	2894830	375121.5	0.941273	INF
57.6094	1185.83	25073431	988402.3	1.304441	INF
57.94292	1194.174	9843189	498586.5	1.126563	INF
57.6094	1261.867	15801669	605043.7	1.171033	INF
57.44215	1329.561	11319608	360578	1.39338	INF

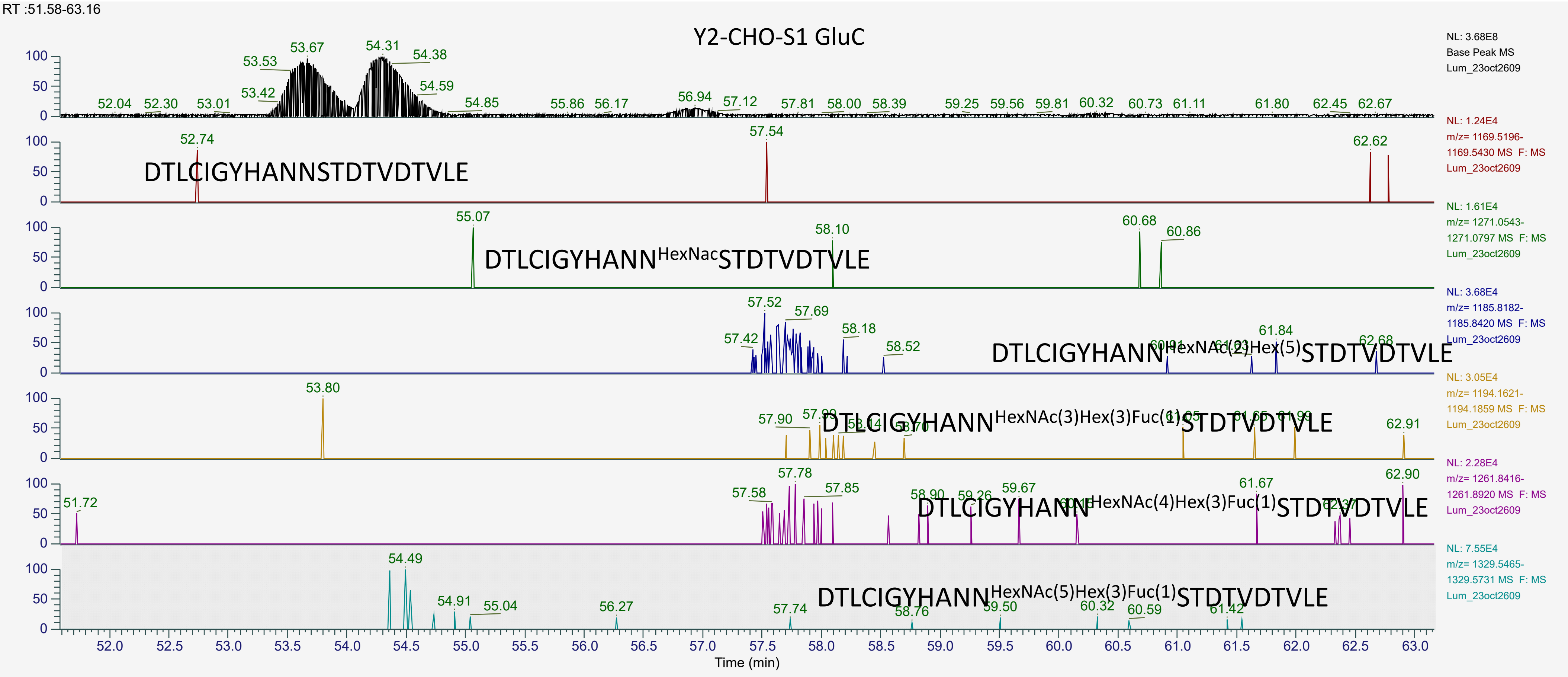
RT :51.58-63.16
Y2-CHO-S1 GluC
NL: 3.68E8
Base Peak MS
Lum_23oct2609
DTLCIGYHANNSTDTVDTVLE
NL: 1.24E4
m/z= 1169.5196-1169.5430 MS F: MS
Lum_23oct2609
DTLCIGYHANN HexNac STDTVDTVLE
NL: 1.61E4
m/z= 1271.0543-1271.0797 MS F: MS
Lum_23oct2609
DTLCIGYHANN HexNAc(2)Hex(5) STDTVDTVLE
NL: 3.68E4
m/z= 1185.8182-1185.8420 MS F: MS
Lum_23oct2609
DTLCIGYHANN HexNAc(3)Hex(3)Fuc(1) STDTVDTVLE
NL: 3.05E4
m/z= 1194.1621-1194.1859 MS F: MS
Lum_23oct2609
DTLCIGYHANN HexNAc(4)Hex(3)Fuc(1) STDTVDTVLE
NL: 2.28E4
m/z= 1261.8416-1261.8920 MS F: MS
Lum_23oct2609
DTLCIGYHANN HexNAc(5)Hex(3)Fuc(1) STDTVDTVLE
NL: 7.55E4
m/z= 1329.5465-1329.5731 MS F: MS
Lum_23oct2609
Time (min)

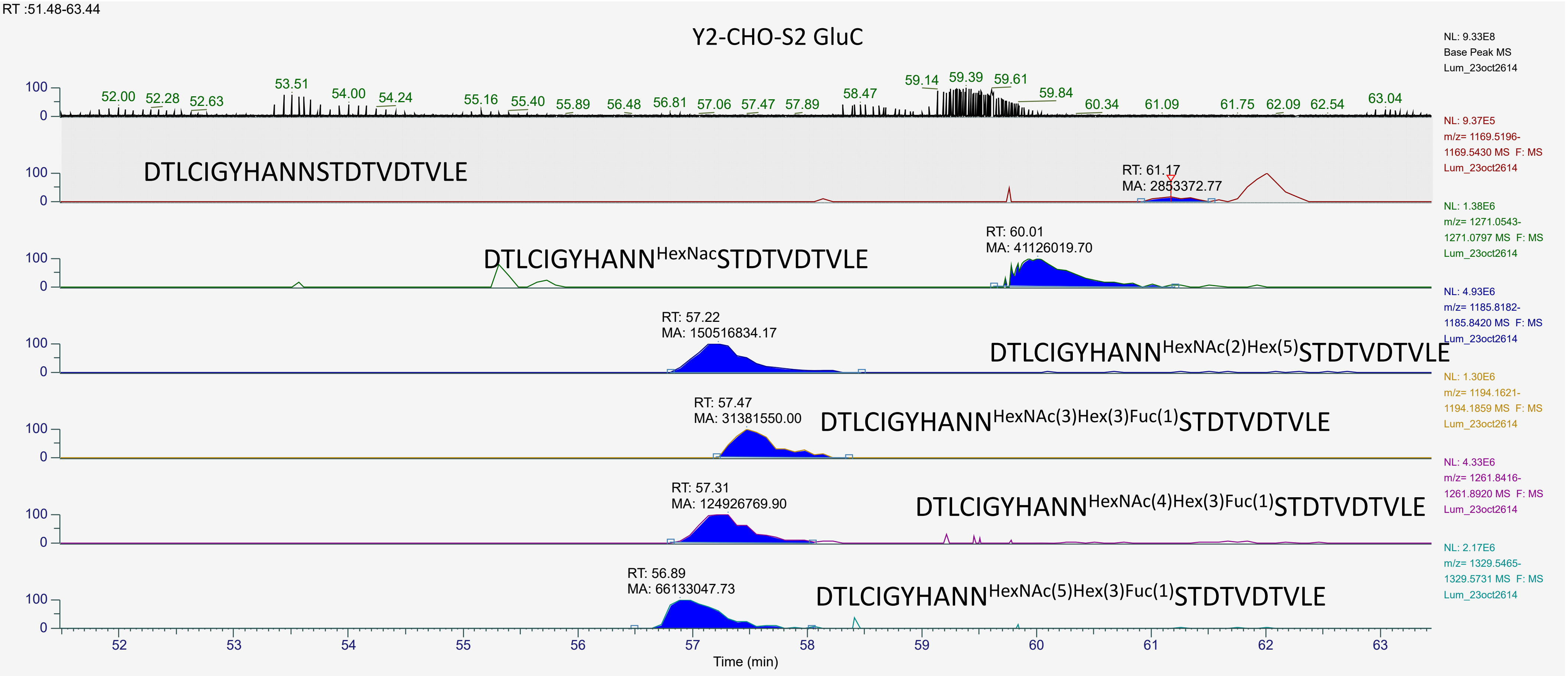

RT (Min)	Base Peak	Peak Area	Peak Height	Baseline Width	SignalToNoise
61.17431	1169.53	2853373	132760	0.608996	INF
60.013	1271.067	41126020	1314802	1.581697	INF
57.22452	1185.829	1.51E+08	4869146	1.665138	INF
57.47317	1194.173	31381550	1261533	1.15799	INF
57.30672	1261.866	1.25E+08	4220458	1.234908	INF
56.89144	1329.558	66133048	2166624	1.547864	INF

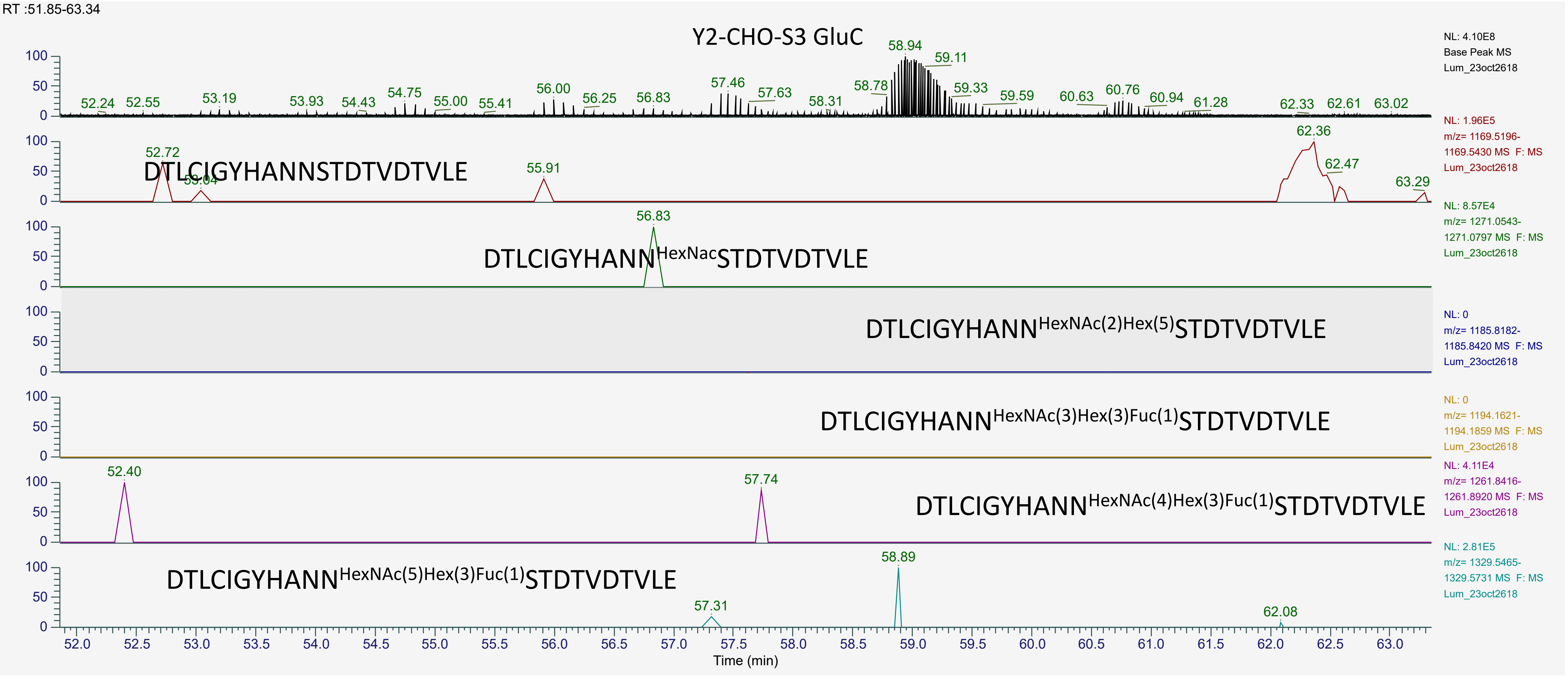

RT :51.85-63.34
Y2-CHO-S3 GluC
NL: 4.10E8
Base Peak MS
Lum_23oct2618
DTLCIGYHANNSTDTVDTVLE
NL: 1.96E5
m/z= 1169.5196-1169.5430 MS F: MS
Lum_23oct2618
DTLCIGYHANN^HexNac STDTVDTVLE
NL: 8.57E4
m/z= 1271.0543-1271.0797 MS F: MS
Lum_23oct2618
DTLCIGYHANN^HexNAc(2)Hex(5) STDTVDTVLE
NL: 0
m/z= 1185.8182-1185.8420 MS F: MS
Lum_23oct2618
DTLCIGYHANN^HexNAc(3)Hex(3)Fuc(1) STDTVDTVLE
NL: 0
m/z= 1194.1621-1194.1859 MS F: MS
Lum_23oct2618
DTLCIGYHANN^HexNAc(4)Hex(3)Fuc(1) STDTVDTVLE
NL: 4.11E4
m/z= 1261.8416-1261.8920 MS F: MS
Lum_23oct2618
DTLCIGYHANN^HexNAc(5)Hex(3)Fuc(1) STDTVDTVLE
NL: 2.81E5
m/z= 1329.5465-1329.5731 MS F: MS
Lum_23oct2618
Time (min)

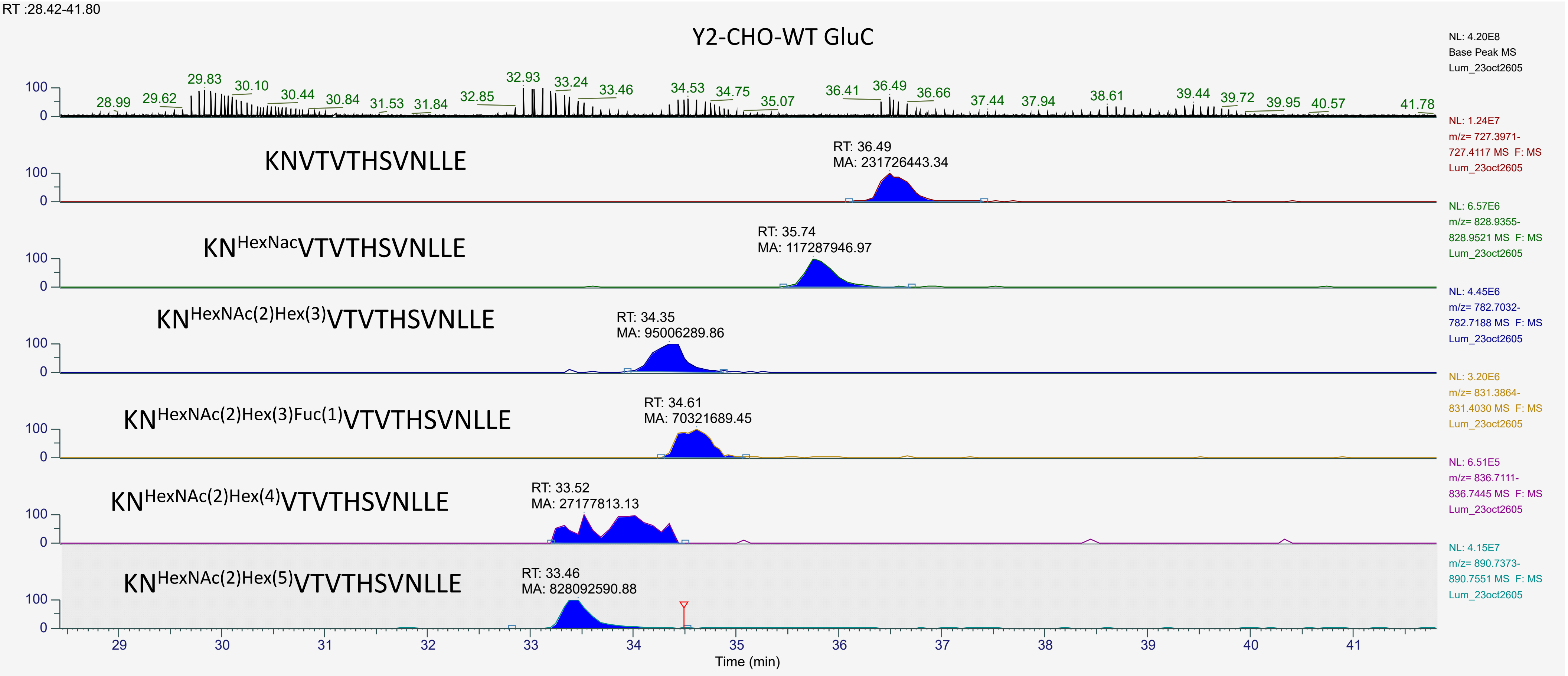

RT (Min)	Base Peak	Peak Area	Peak Height	Baseline Width	SignalToNoise
36.49257	727.4037	2.32E+08	12246240	1.312053	INF
35.74392	828.9437	1.17E+08	6466406	1.245978	INF
34.34969	782.7105	95006290	4350277	0.934147	INF
34.61448	831.3962	70321689	3166210	0.830353	INF
33.51813	836.7274	27177813	641155	1.302144	INF
33.45806	890.7466	8.28E+08	41507732	1.698449	INF

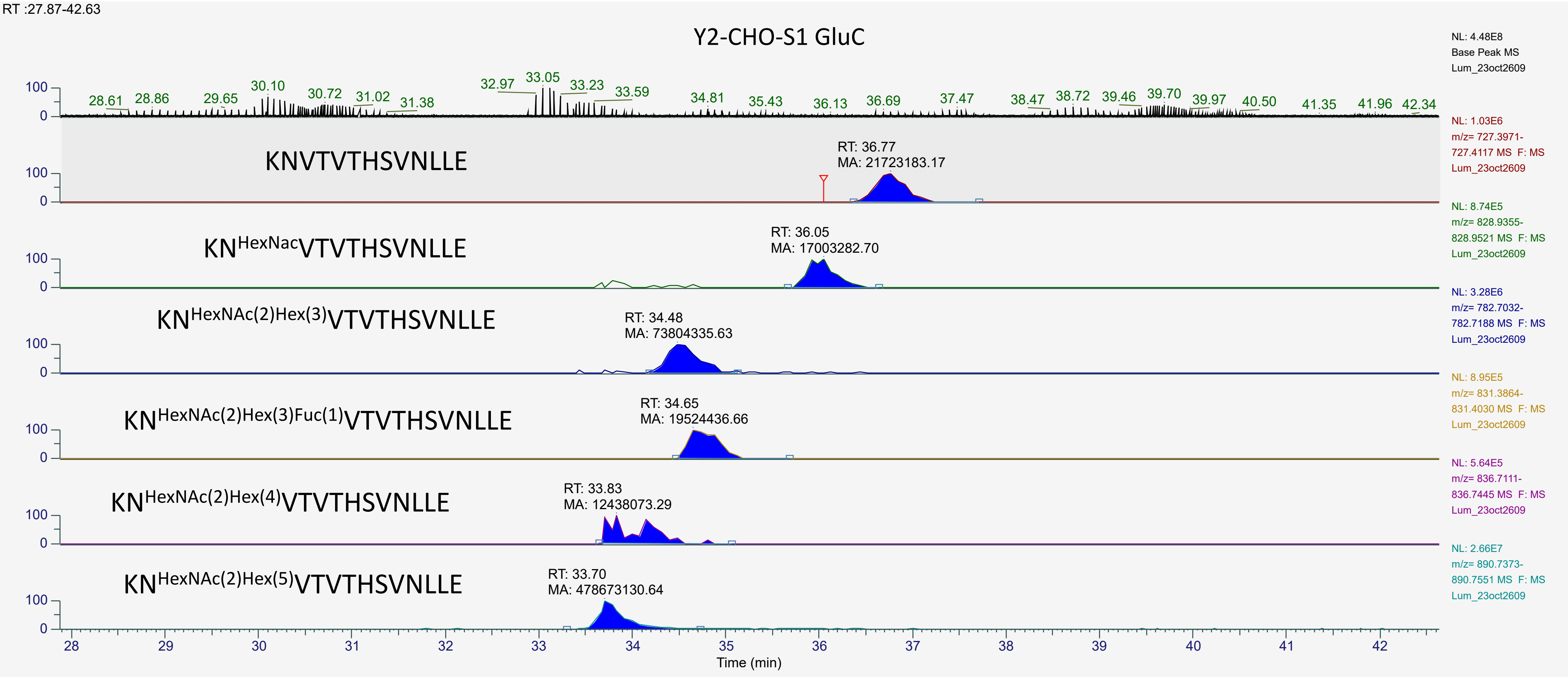

RT (Min)	Base Peak	Peak Area	Peak Height	Baseline Width	SignalToNoise
36.76751	727.4043	21723183	1012925	1.342764	INF
36.05143	828.944	17003283	863382.7	0.978449	INF
34.48122	782.7108	73804336	3229147	0.947221	INF
34.64779	831.397	19524437	879144.2	1.217856	INF
33.83106	836.7283	12438073	543725.2	1.41565	INF
33.70175	890.746	4.79E+08	26637998	1.436468	INF

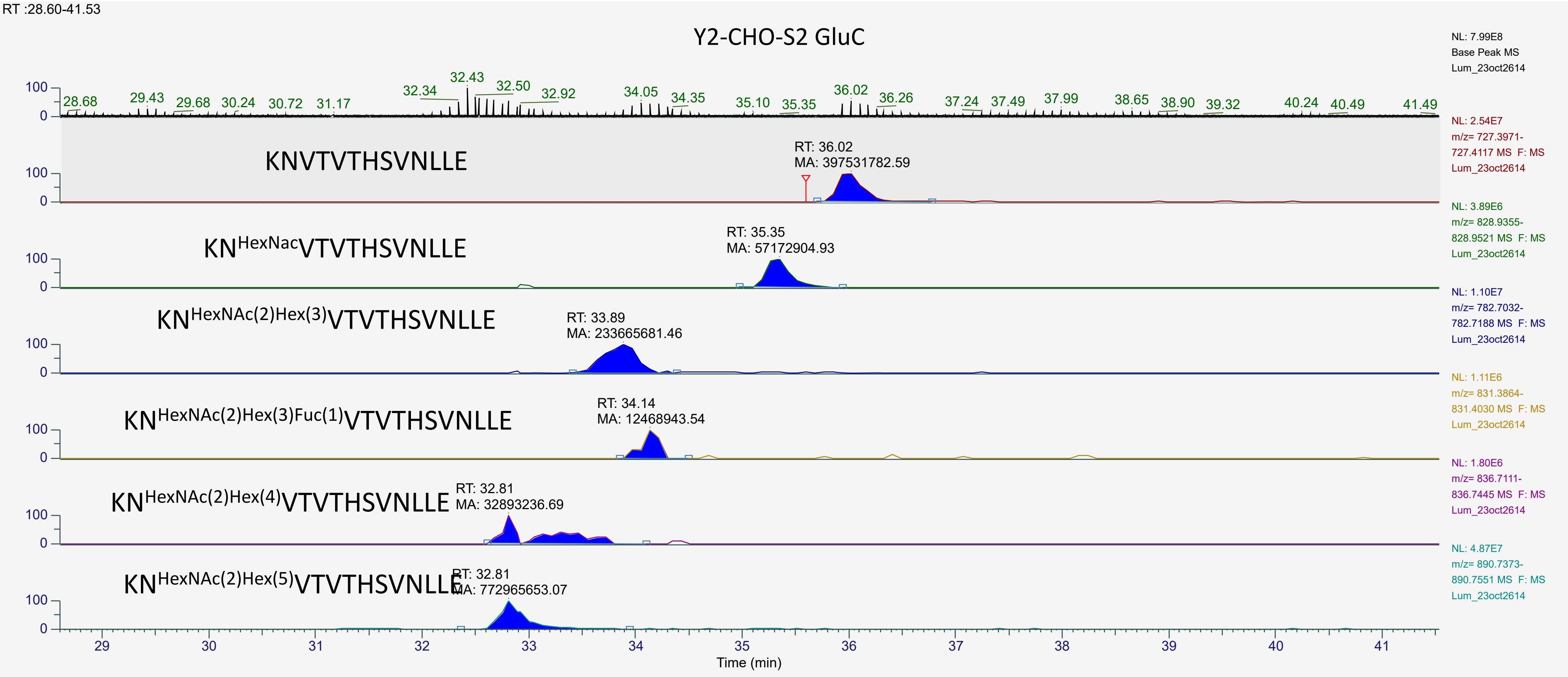

RT (Min)	Base Peak	Peak Area	Peak Height	Baseline Width	SignalToNoise
36.02136	727.4033	3.98E+08	24674107	1.07598	INF
35.35131	828.9438	57172905	3737667	0.966559	INF
33.88558	782.7104	2.34E+08	10863429	0.975677	INF
34.13585	831.3963	12468944	1100942	0.638293	INF
32.806	836.7288	32893237	1732711	1.49503	INF
32.806	890.745	7.73E+08	48707732	1.586191	INF

RT :28.42-38.78

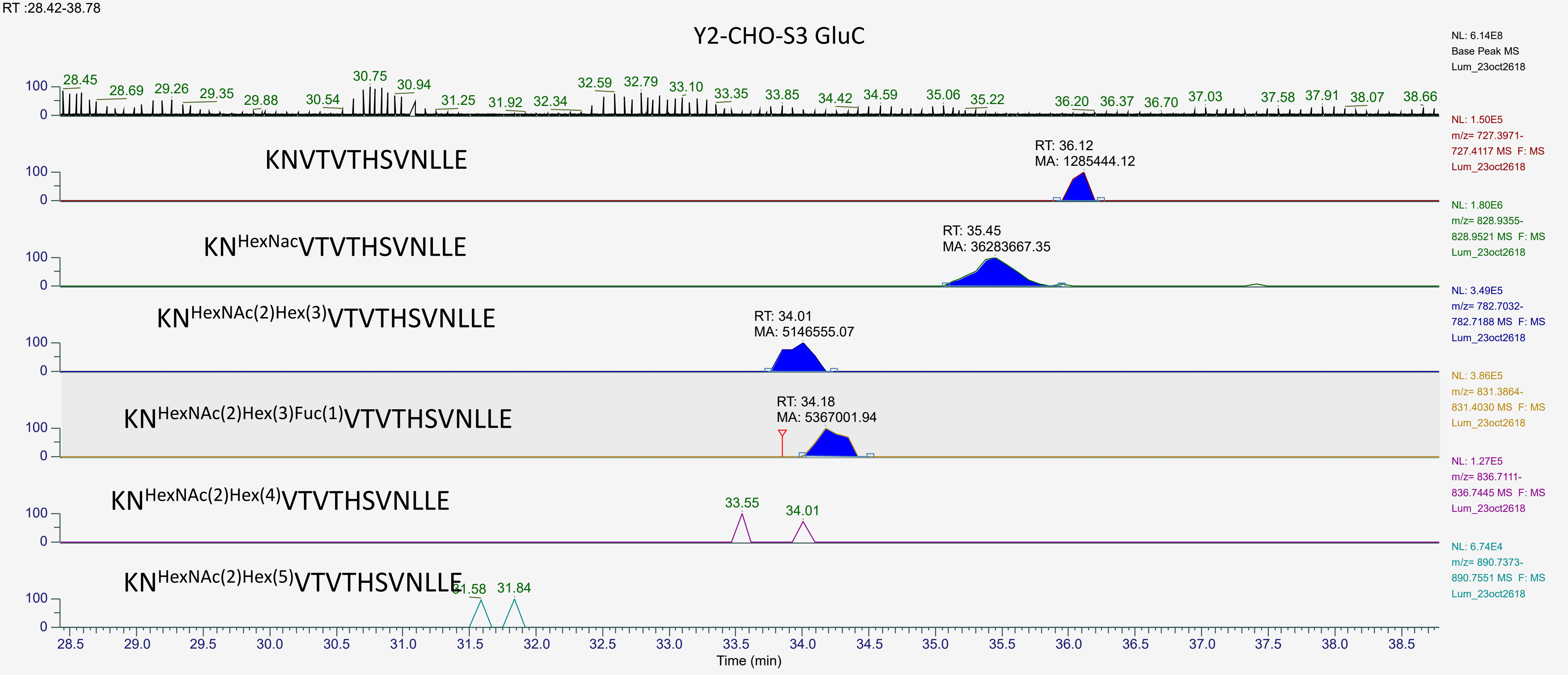

RT (Min)	Base Peak	Peak Area	Peak Height	Baseline Width	SignalToNoise
36.11508	727.4037	1285444	148928.9	0.328761	INF
35.45144	828.9435	36283667	1776264	0.869389	INF
34.00894	782.7115	5146555	346061.9	0.496812	INF
34.1772	831.3961	5367002	375938.5	0.511425	INF

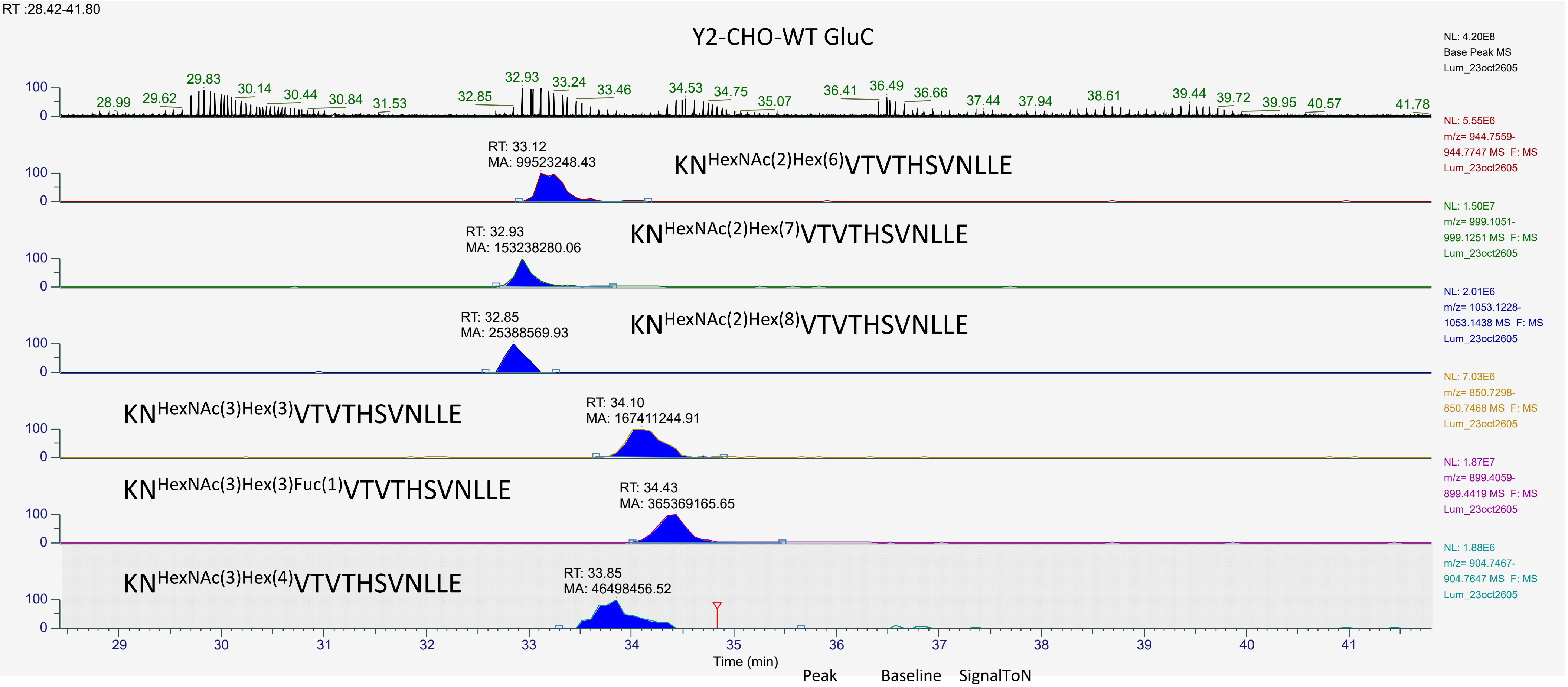

RT (Min)	Base Peak	Peak Area	Peak Height	Baseline Width	SignalToNoise
33.11586	944.7632	99523248	5475696	1.267977	INF
32.93202	999.1151	1.53E+08	14498860	1.135502	INF
32.84889	1053.133	25388570	1985013	0.690764	INF
34.09943	850.7383	1.67E+08	6838252	1.23959	INF
34.43441	899.4233	3.65E+08	18464956	1.46669	INF
33.85176	904.756	46498457	1878307	2.356167	INF

RT :27.86-42.02

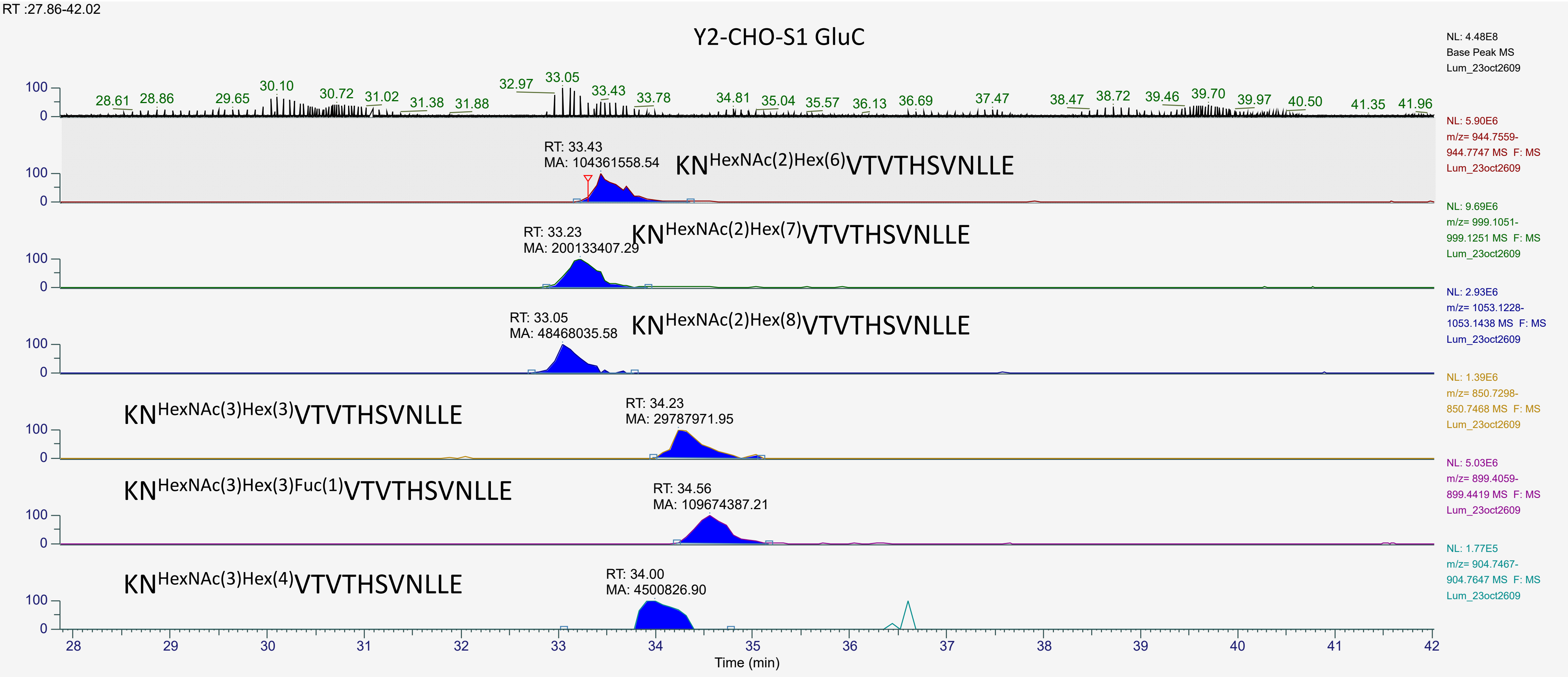

RT (Min)	Base Peak	Peak Area	Peak Height	Baseline Width	SignalToNoise
33.43497	944.764	1.04E+08	5800807	1.171655	INF
33.22793	999.1163	2E+08	9552942	1.051485	INF
33.04829	1053.133	48468036	2888939	1.061499	INF
34.23249	850.7384	29787972	1343719	1.11157	INF
34.56478	899.4244	1.1E+08	4894508	0.951238	INF
33.9958	904.7562	4500827	177282.6	1.722241	INF

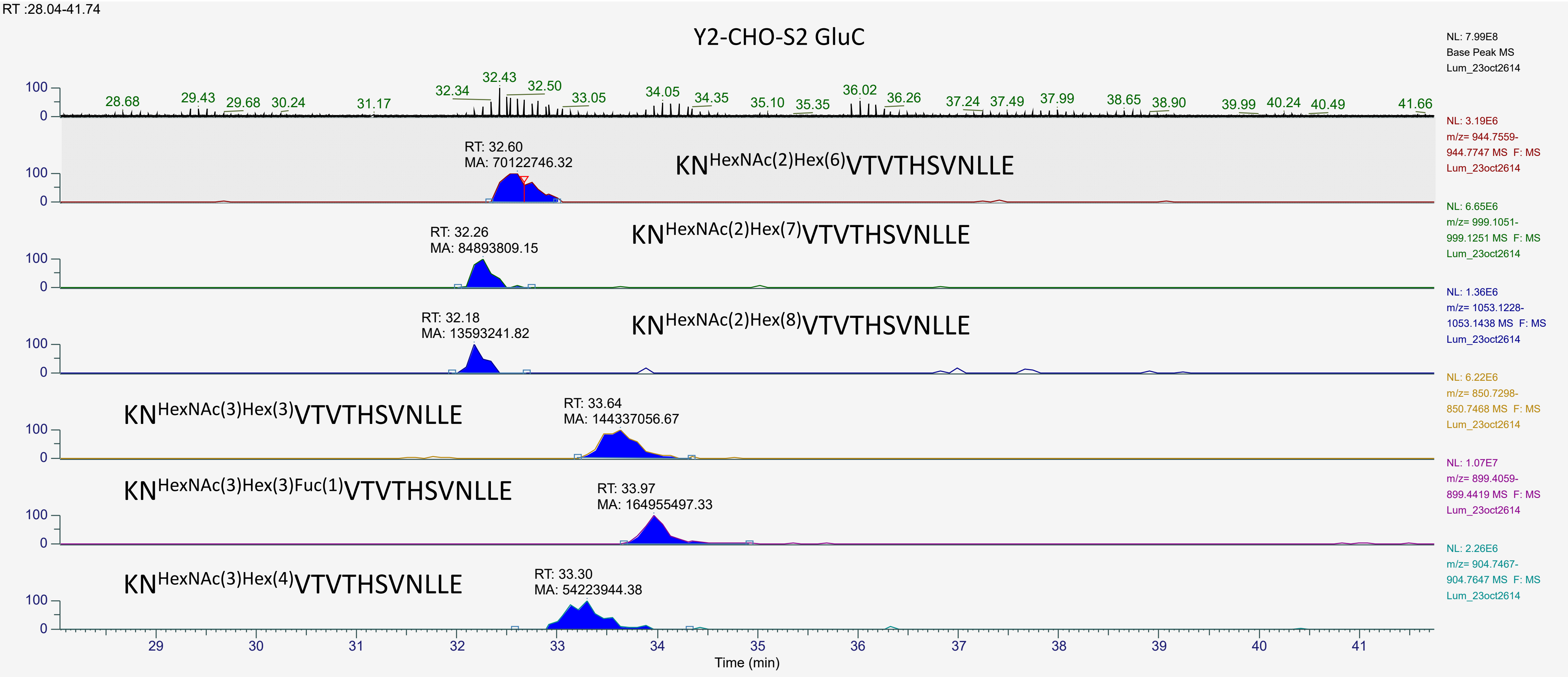

RT (Min)	Base Peak	Peak Area	Peak Height	Baseline Width	SignalToNoise
32.60499	944.7642	70122746	3155429	0.678218	INF
32.25963	999.1158	84893809	6556537	0.726662	INF
32.17715	1053.133	13593242	1342186	0.74604	INF
33.63521	850.7381	1.44E+08	6063390	1.133593	INF
33.96848	899.4235	1.65E+08	10508536	1.259419	INF
33.29912	904.7552	54223944	2256132	1.743811	INF

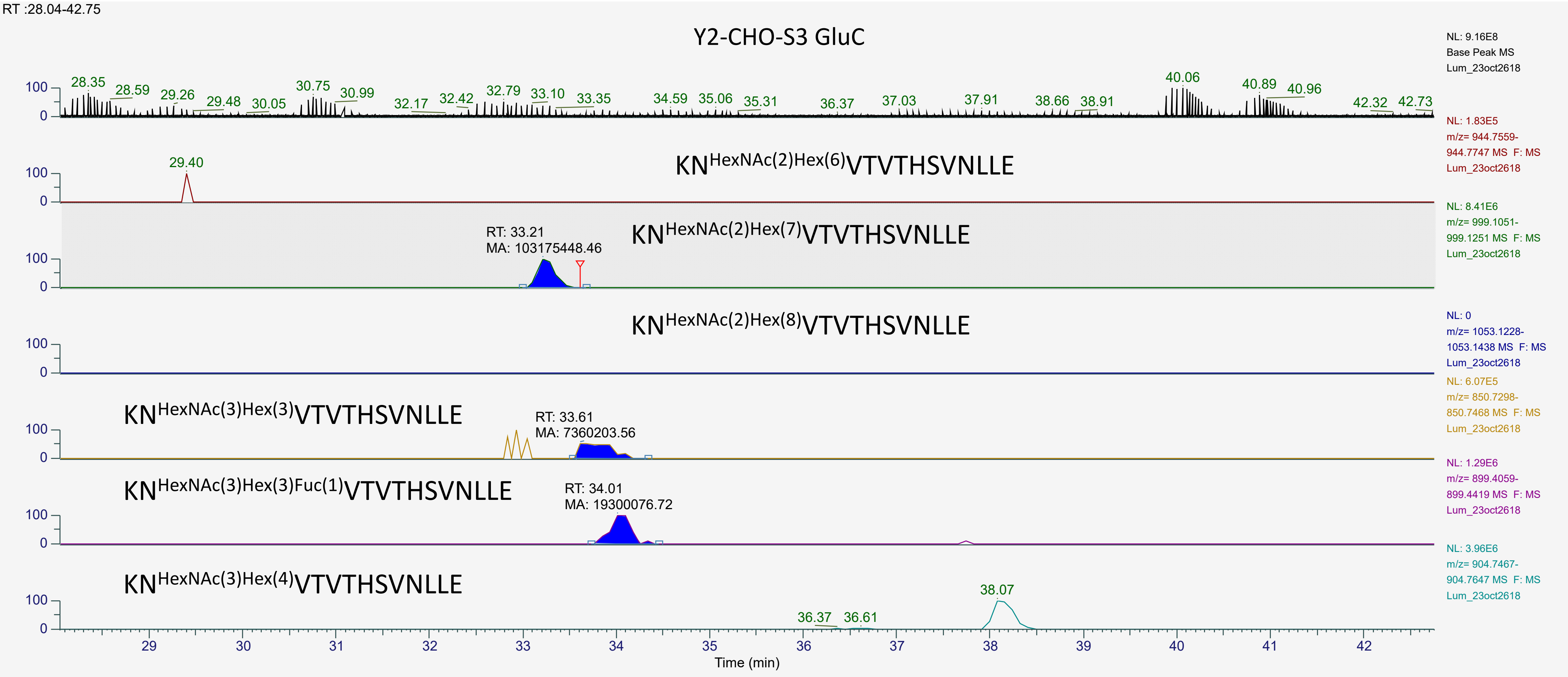

RT (Min)	Base Peak	Peak Area	Peak Height	Baseline Width	SignalToNoise
33.2125	999.1158	1.03E+08	8287148	0.686605	INF
33.61317	850.7369	7360204	295313.5	0.801038	INF
34.00894	899.4241	19300077	1266664	0.728216	INF

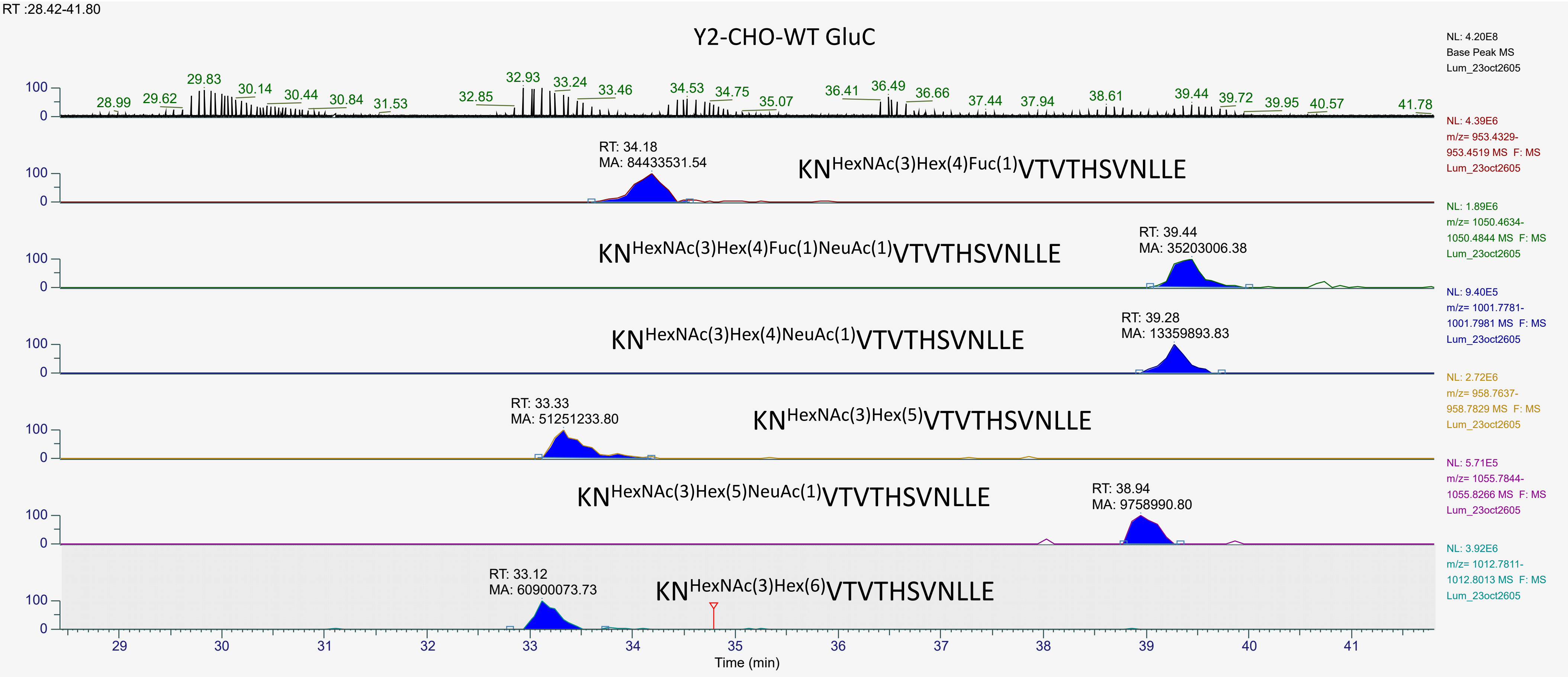

RT (Min)	Base Peak	Peak Area	Peak Height	Baseline Width	SignalToNoise
34.1841	953.4424	84433532	4364570	0.955714	INF
39.44191	1050.474	35203006	1845113	0.965177	INF
39.27577	1001.788	13359894	928564.3	0.804314	INF
33.32972	958.7736	51251234	2636936	1.107115	INF
38.94285	1055.805	9758991	562622.7	0.558289	INF
33.11586	1012.79	60900074	3916990	0.927327	INF

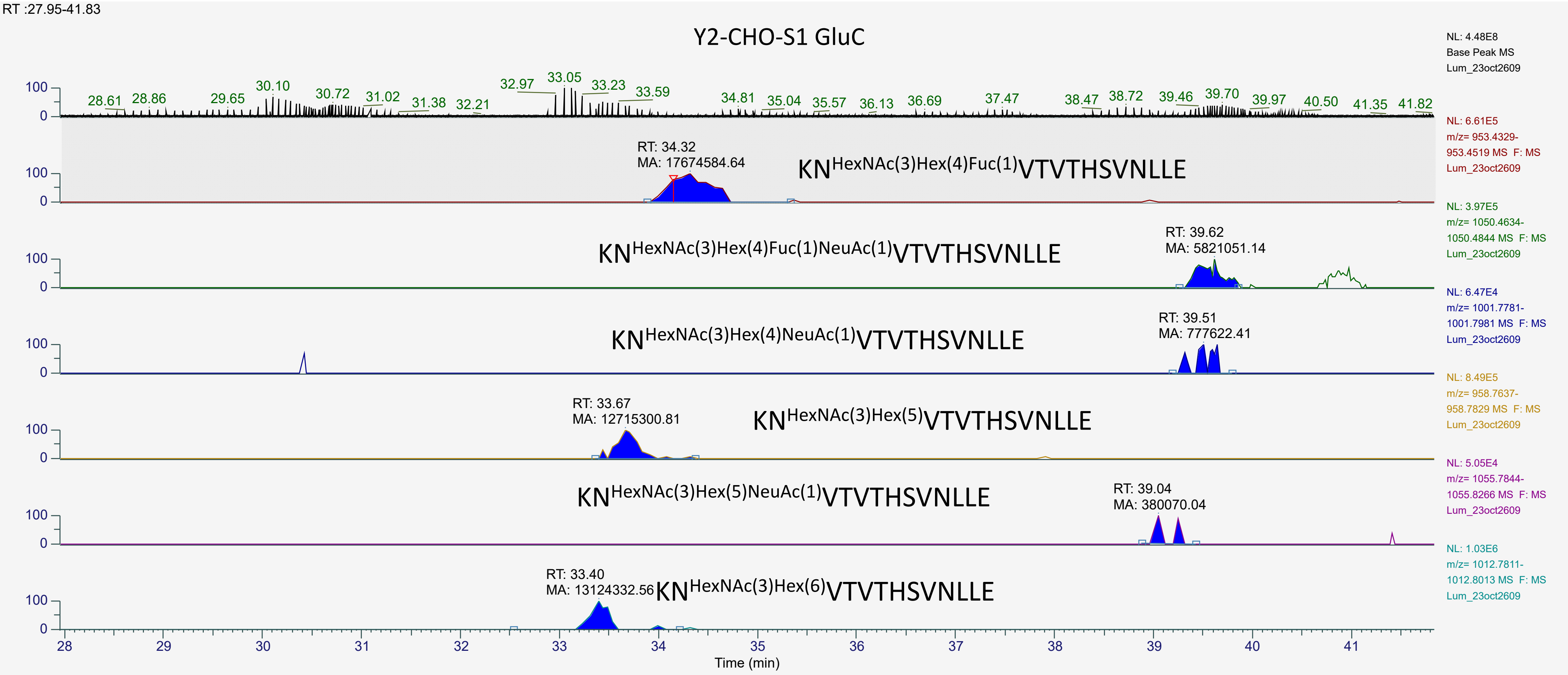

RT (Min)	Base Peak	Peak Area	Peak Height	Baseline Width	SignalToNoise
34.31536	953.4427	17674585	651771.2	1.452786	INF
39.61822	1050.474	5821051	394014.5	0.598784	INF
39.507	1001.79	777622.4	64115.36	0.598784	INF
33.66982	958.7733	12715301	849004.8	1.011061	INF
39.04455	1055.807	380070	49010.39	0.549806	INF
33.39599	1012.792	13124333	1034537	1.678873	INF

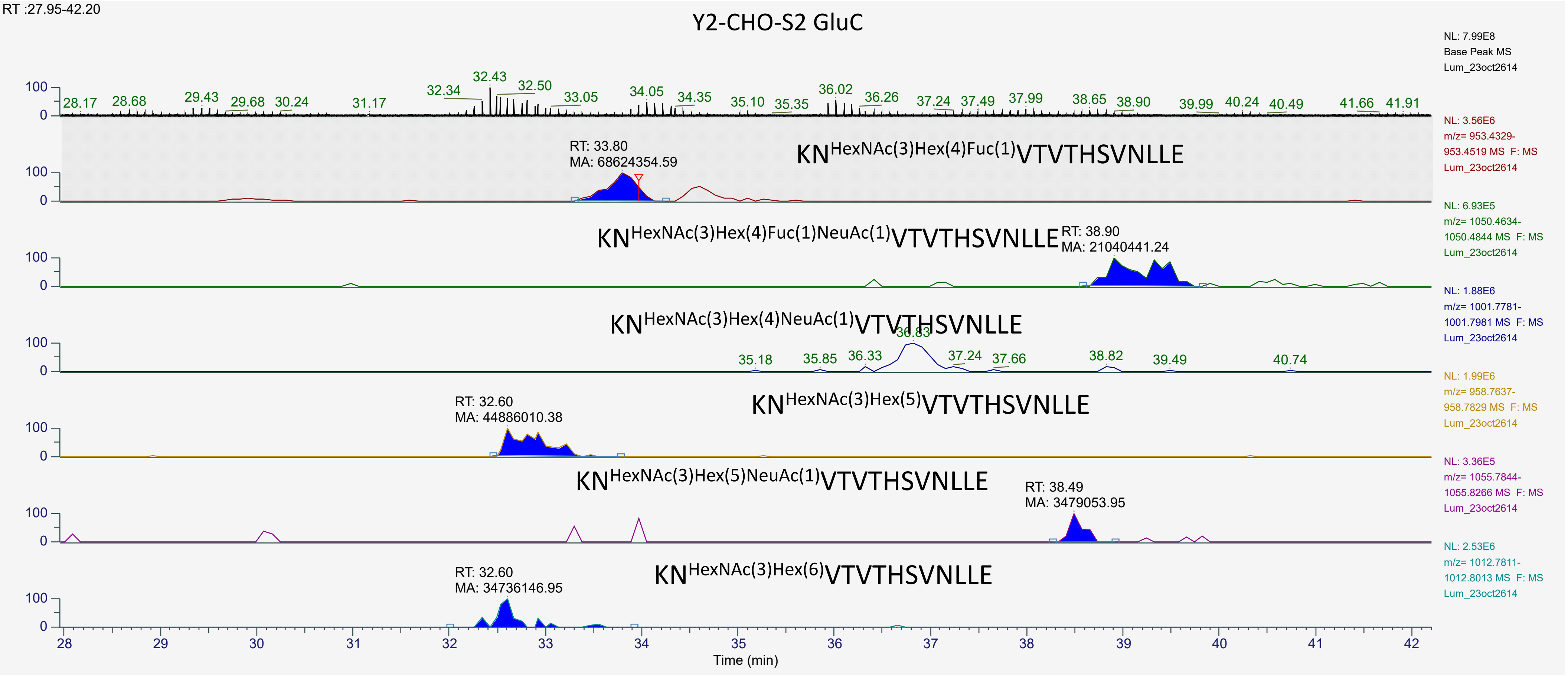

RT (Min)	Base Peak	Peak Area	Peak Height	Baseline Width	SignalToNoise
33.80111	953.4421	68624355	3487243	0.947313	INF
38.9043	1050.474	21040441	672110.4	1.239569	INF
32.60499	958.774	44886010	1876726	1.320191	INF
38.4882	1055.806	3479054	335697.3	0.644989	INF
32.60499	1012.792	34736147	2526427	1.904733	INF

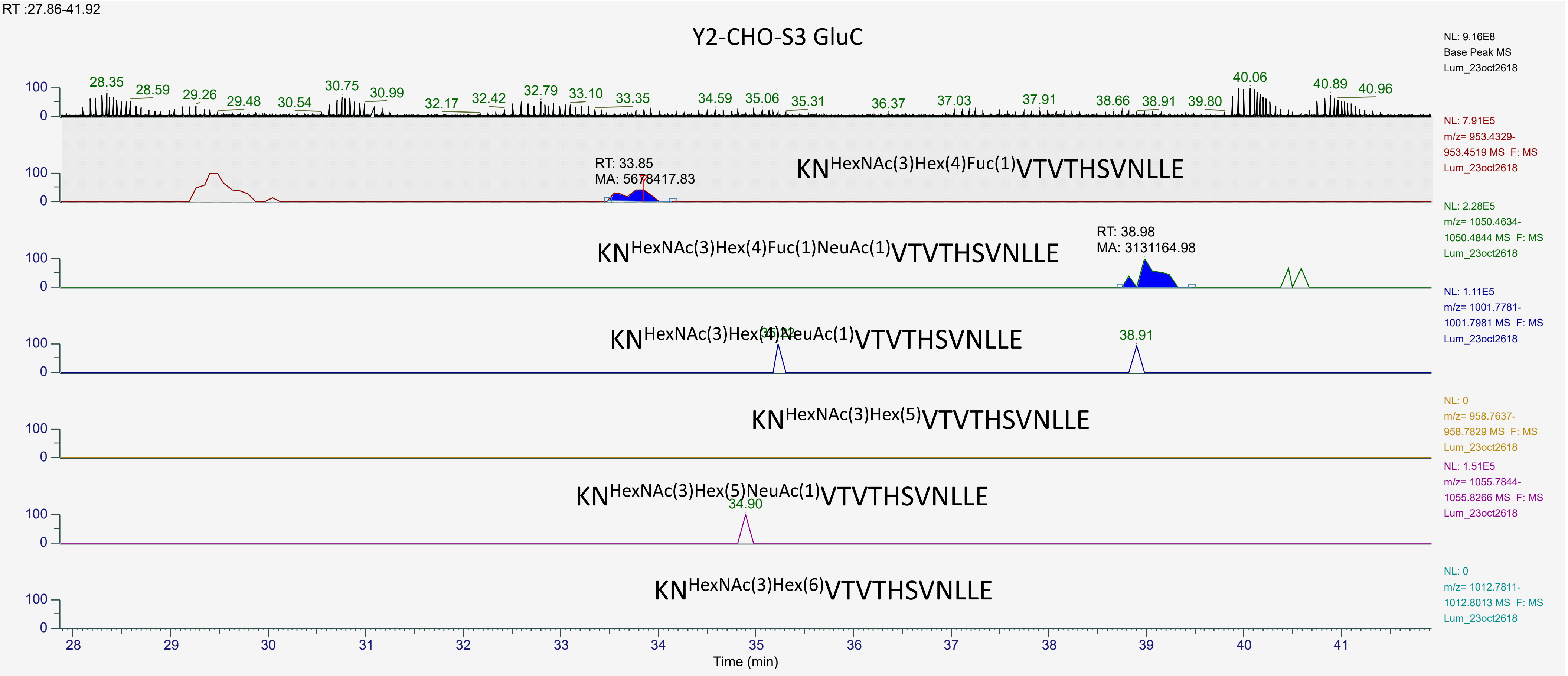

RT (Min)	Base Peak	Peak Area	Peak Height	Baseline Width	SignalToNoise
33.85045	953.4435	5678418	309385	0.666513	INF
38.98383	1050.472	3131165	225846.5	0.736149	INF

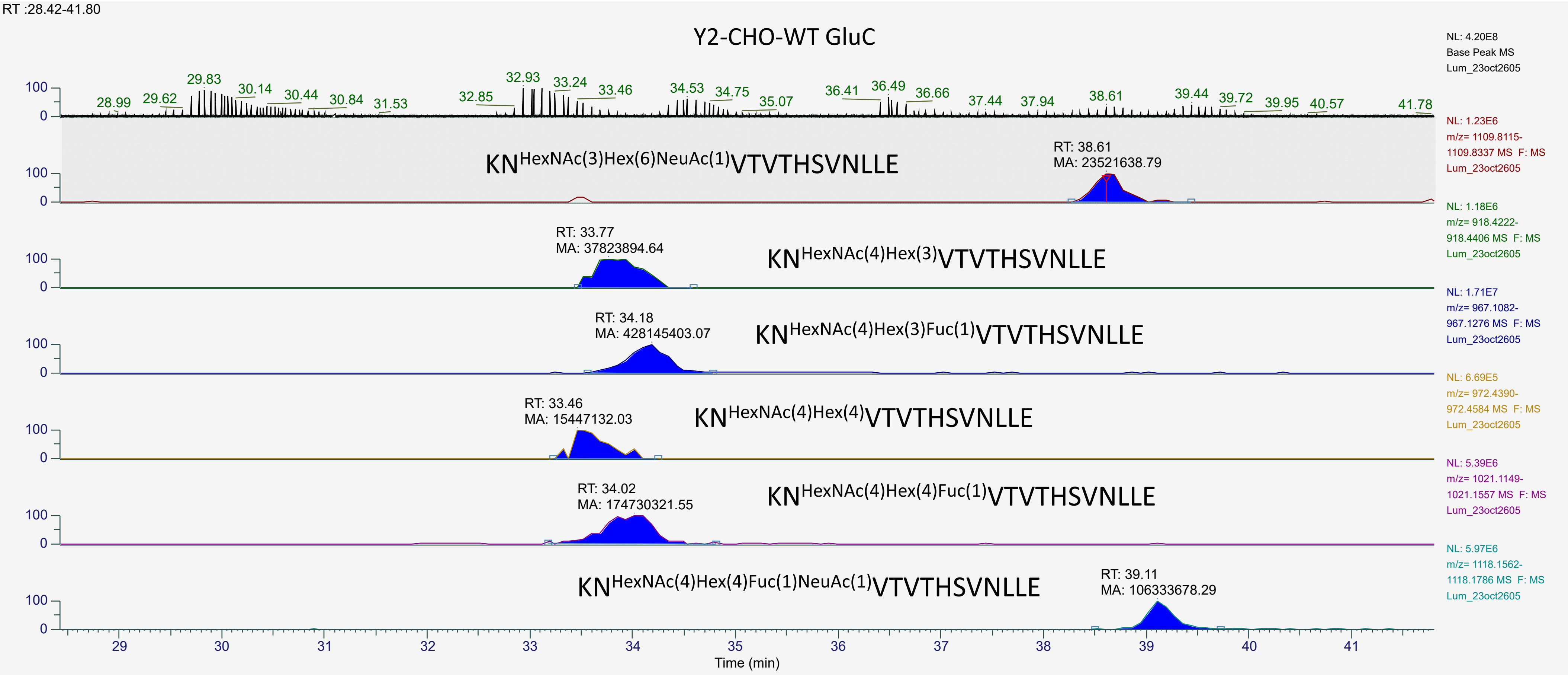

RT (Min)	Base Peak	Peak Area	Peak Height	Baseline Width	SignalToNoise
38.60799	1109.823	23521639	1211869	1.16389	INF
33.76871	918.4315	37823895	1165183	1.12604	INF
34.1841	967.1179	4.28E+08	16892190	1.220665	INF
33.45806	972.4499	15447132	661127.4	1.021952	INF
34.01676	1021.135	1.75E+08	5284947	1.637016	INF
39.10995	1118.167	1.06E+08	5968554	1.220665	INF

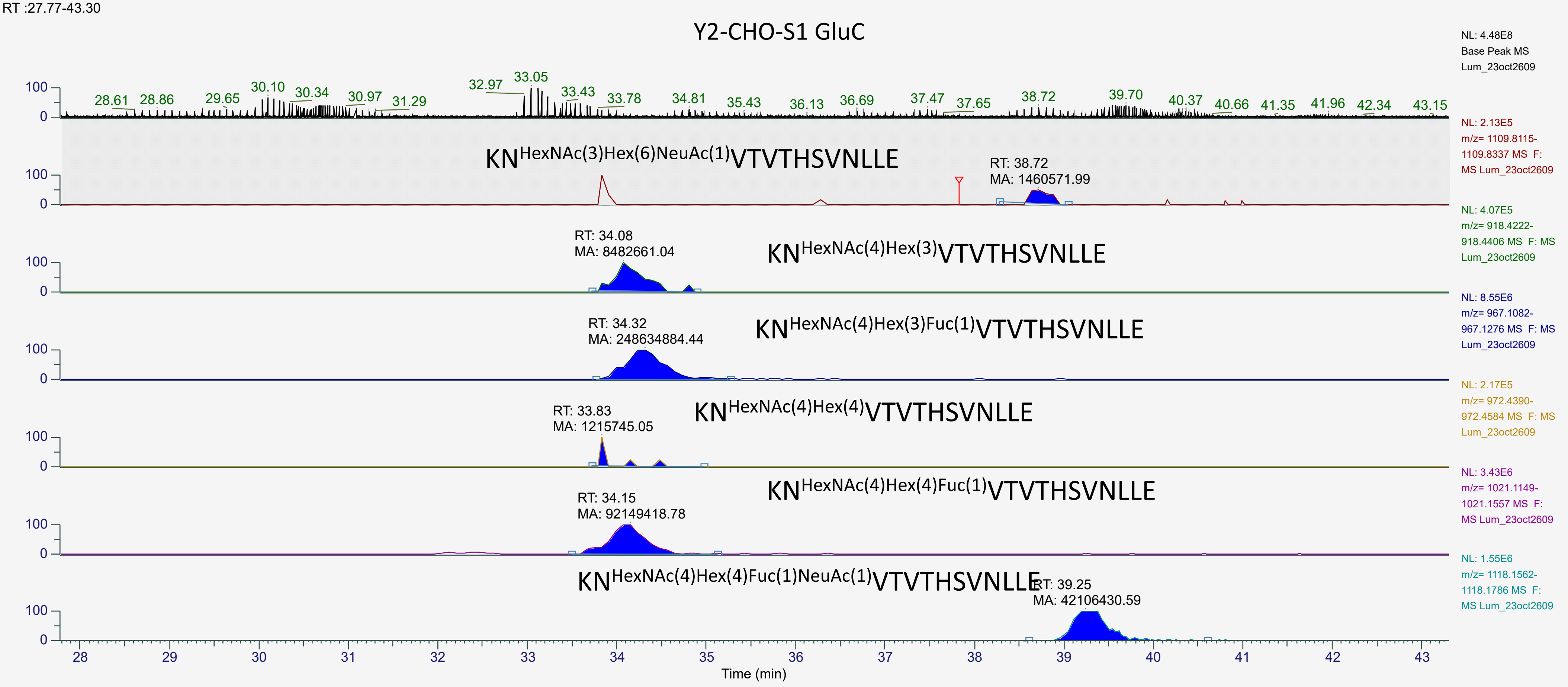

RT (Min)	Base Peak	Peak Area	Peak Height	Baseline Width	SignalToNoise
38.71968	1109.823	1460572	89599.01	0.768812	INF
34.07858	918.4318	8482661	388981.6	1.175184	INF
34.31536	967.1184	2.49E+08	8547758	1.504675	INF
33.83106	972.4501	1215745	208604.3	1.252065	INF
34.14937	1021.137	92149419	3384807	1.63726	INF
39.24591	1118.168	42106431	1549618	1.999875	INF

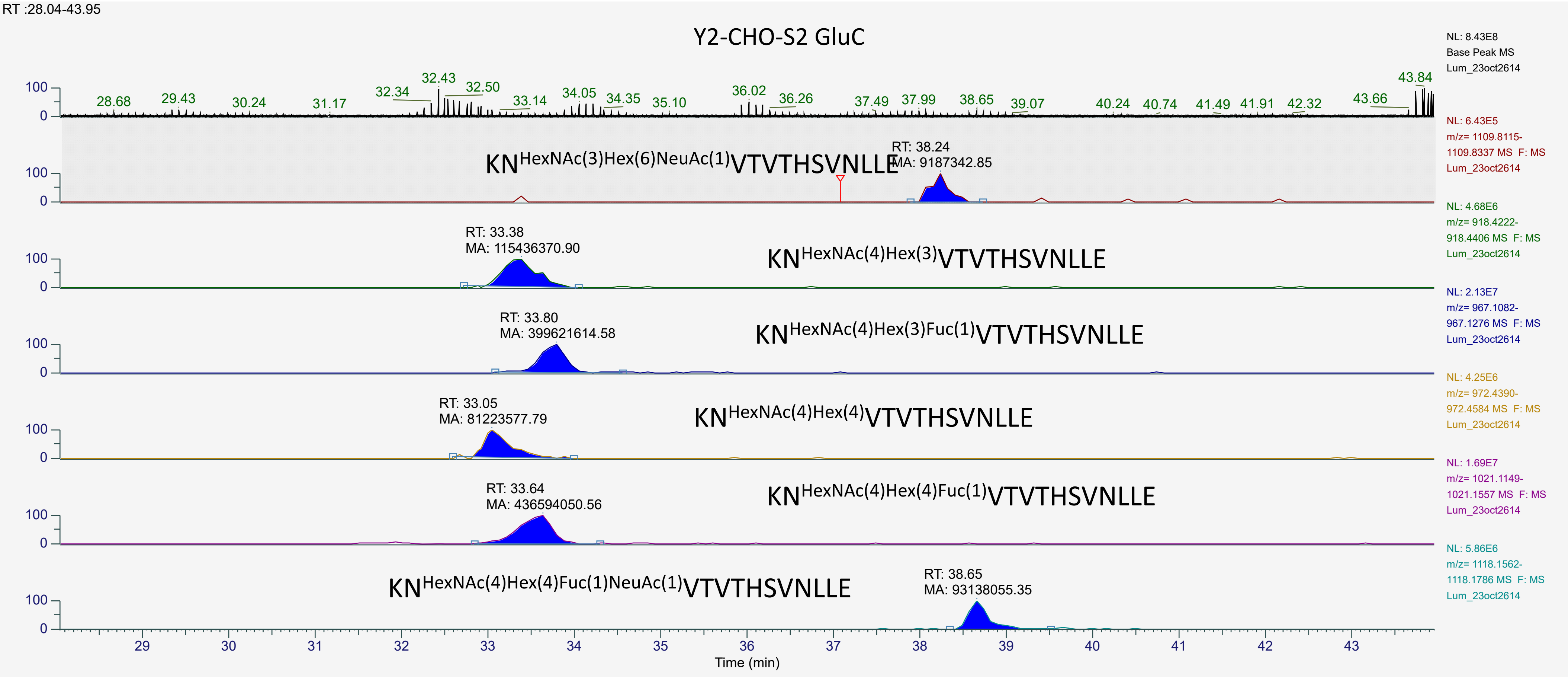

RT (Min)	Base Peak	Peak Area	Peak Height	Baseline Width	SignalToNoise
38.23988	1109.821	9187343	642972.1	0.843883	INF
33.38141	918.431	1.15E+08	4486562	1.327709	INF
33.80111	967.118	4E+08	20626362	1.473982	INF
33.05139	972.4495	81223578	4014244	1.406471	INF
33.63521	1021.136	4.37E+08	16736063	1.45103	INF
38.65495	1118.167	93138055	5862243	1.169823	INF

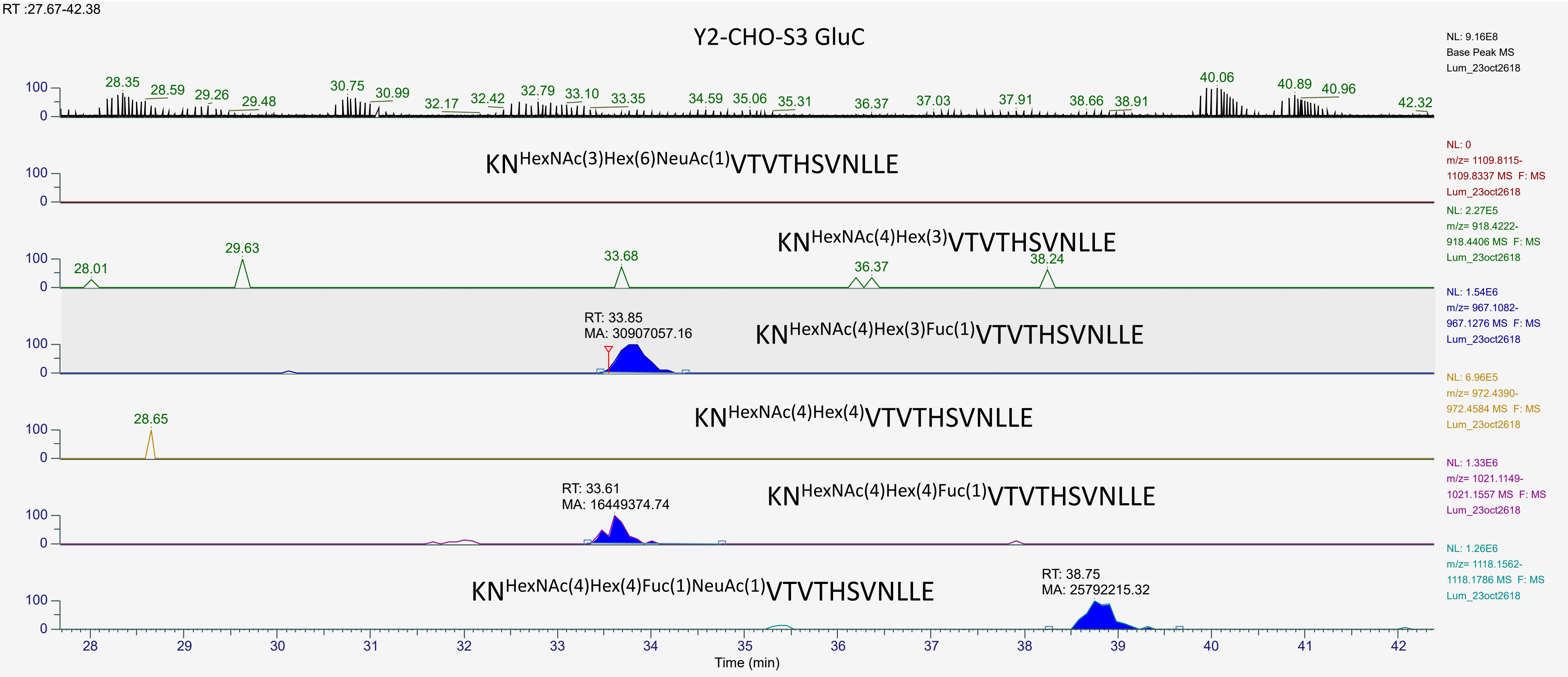

RT (Min)	Base Peak	Peak Area	Peak Height	Baseline Width	SignalToNoise
33.85045	967.118	30907057	1503039	0.915474	INF
33.61317	1021.134	16449375	1289217	1.435627	INF
38.74859	1118.167	25792215	1261835	1.404417	INF

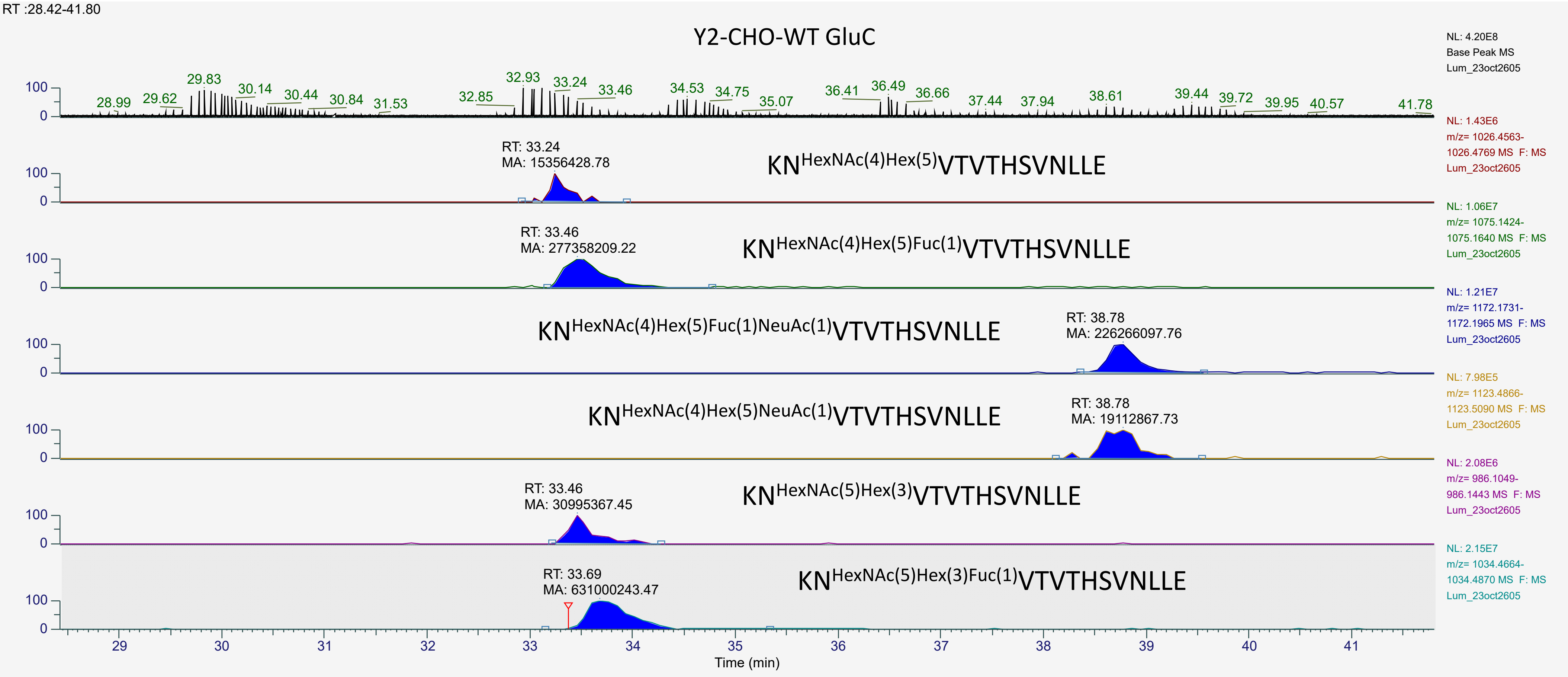

RT (Min)	Base Peak	Peak Area	Peak Height	Baseline Width	SignalToNoise
33.24426	1026.468	15356429	1370585	1.021952	INF
33.45806	1075.154	2.77E+08	10562098	1.608628	INF
38.77565	1172.185	2.26E+08	11804884	1.20174	INF
38.77565	1123.498	19112868	789408.1	1.419378	INF
33.45806	986.1248	30995367	2013007	1.059802	INF
33.68533	1034.477	6.31E+08	21539170	2.195304	INF

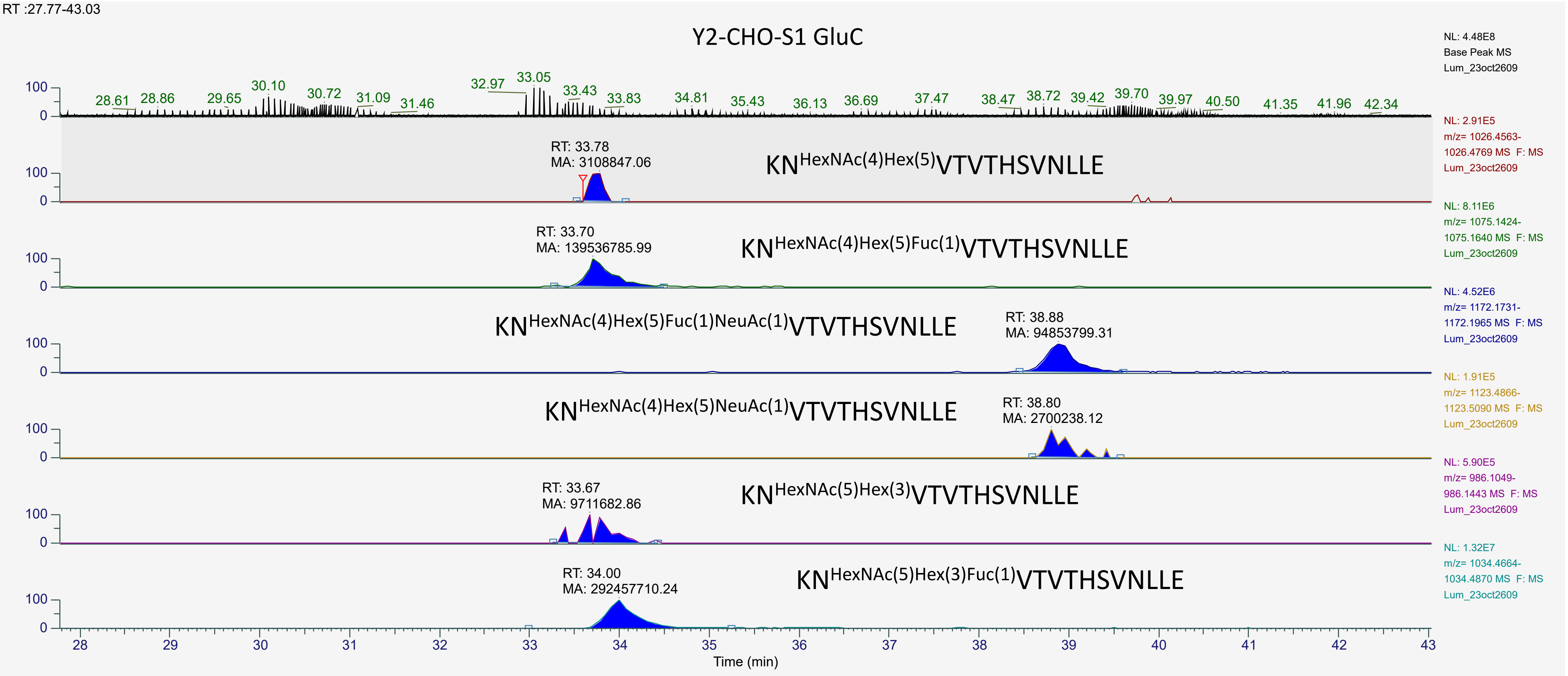

RT (Min)	Base Peak	Peak Area	Peak Height	Baseline Width	SignalToNoise
33.78462	1026.467	3108847	282133.2	0.539604	INF
33.70175	1075.153	1.4E+08	7778701	1.219505	INF
38.87903	1172.185	94853799	4402243	1.154752	INF
38.803	1123.501	2700238	181750.7	0.982079	INF
33.66982	986.126	9711683	574504.4	1.165672	INF
33.9958	1034.477	2.92E+08	13160127	2.255792	INF

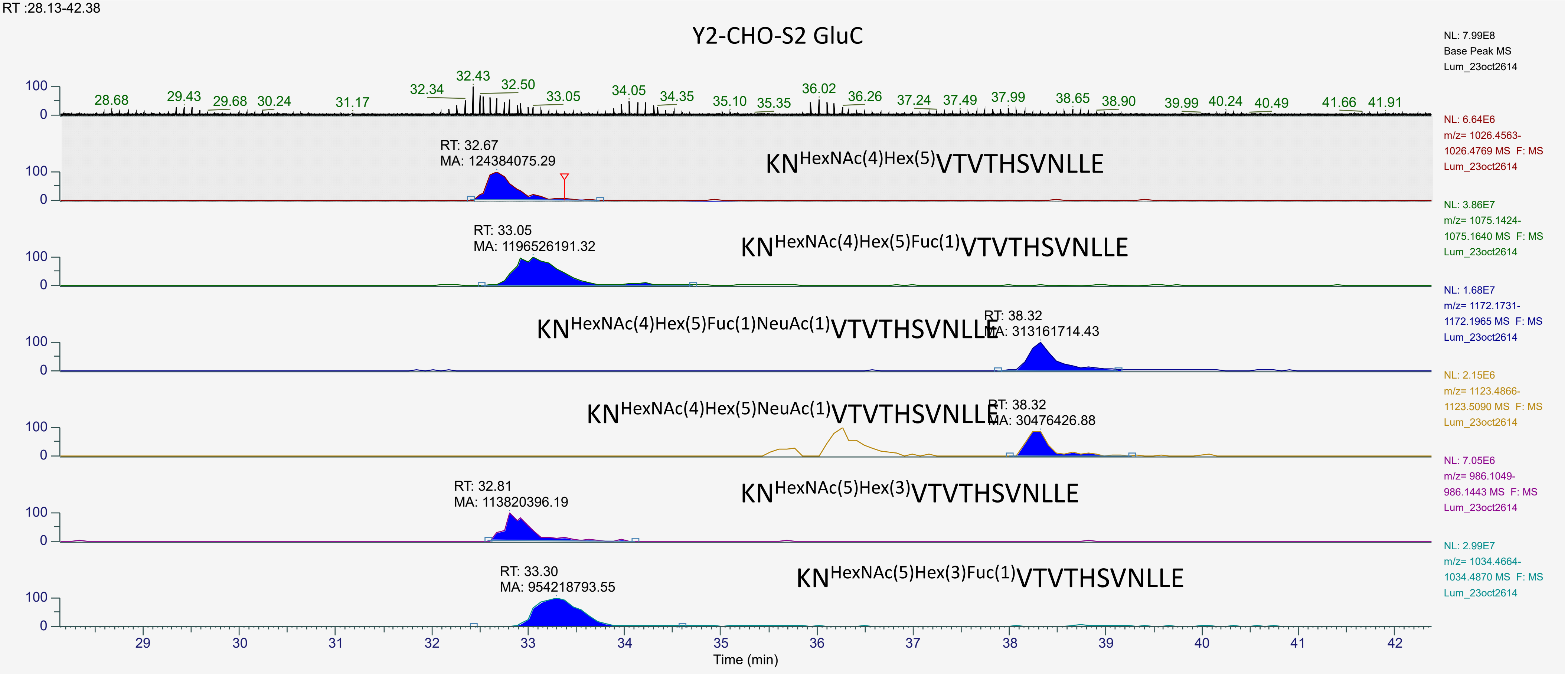

RT (Min)	Base Peak	Peak Area	Peak Height	Baseline Width	SignalToNoise
32.67158	1026.467	1.24E+08	6414171	1.340347	INF
33.05139	1075.153	1.2E+09	37968641	2.196959	INF
38.32296	1172.185	3.13E+08	16557046	1.249646	INF
38.32296	1123.498	30476427	1801778	1.269802	INF
32.806	986.1232	1.14E+08	6673674	1.521771	INF
33.29912	1034.476	9.54E+08	29864280	2.176838	INF

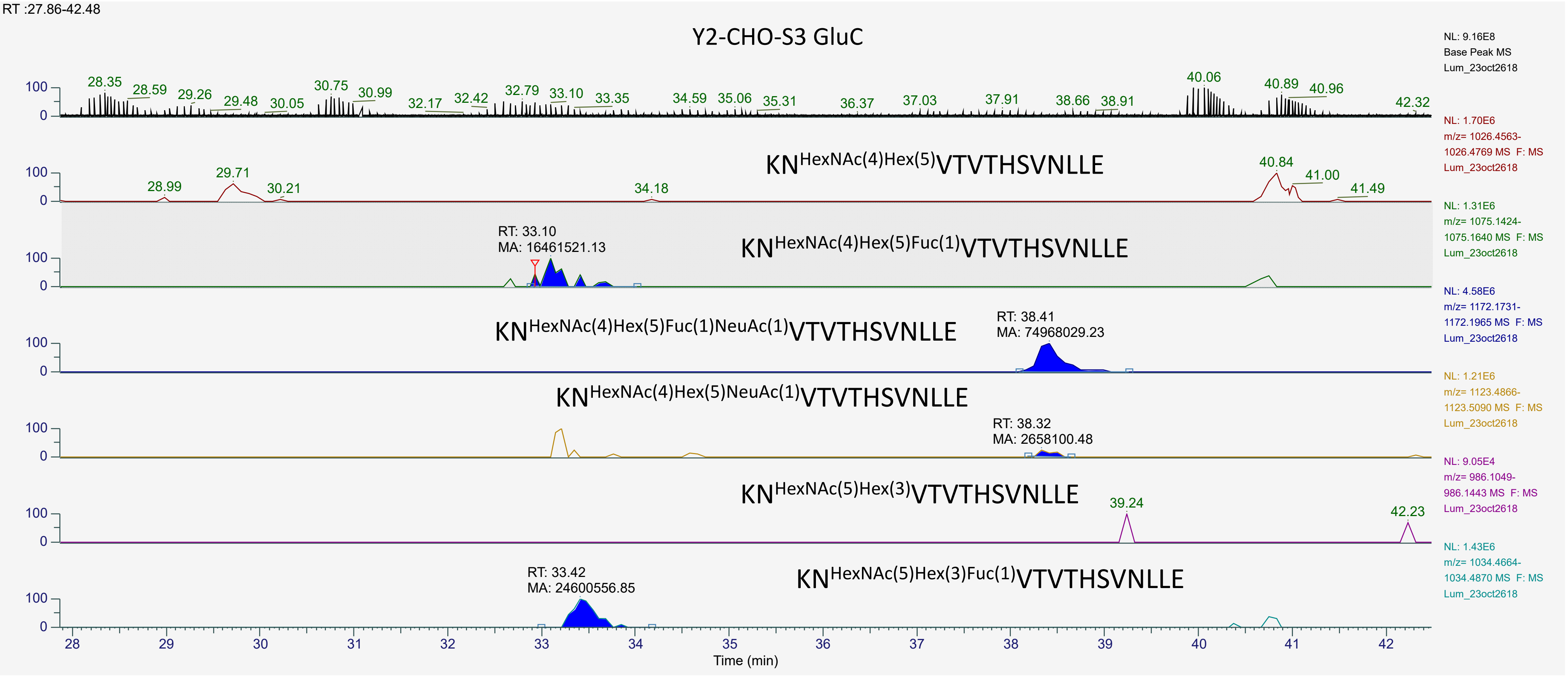

RT (Min)	Base Peak	Peak Area	Peak Height	Baseline Width	SignalToNoise
33.09652	1075.153	16461521	1287301	1.137341	INF
38.40955	1172.185	74968029	4506943	1.168359	INF
38.32457	1123.499	2658100	221130.9	0.454875	INF
33.41547	1034.477	24600557	1434539	1.17854	INF

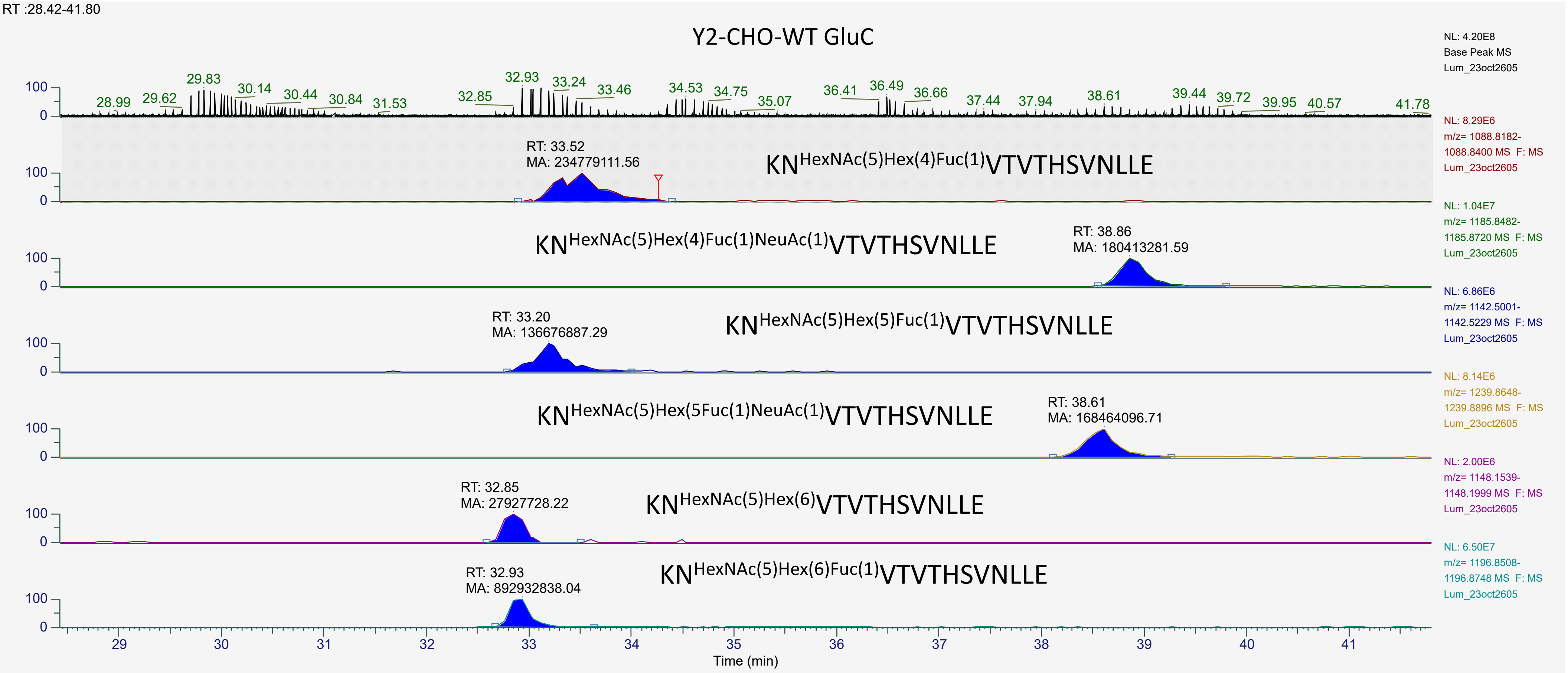

RT (Min)	Base Peak	Peak Area	Peak Height	Baseline Width	SignalToNoise
33.51813	1088.829	2.35E+08	8192614	1.50454	INF
38.85911	1185.859	1.8E+08	10049849	1.249052	INF
33.19854	1142.513	1.37E+08	6787019	1.211202	INF
38.60799	1239.877	1.68E+08	8141642	1.154427	INF
32.84889	1148.177	27927728	1965619	0.917864	INF
32.93202	1196.863	8.93E+08	62995946	0.965177	INF

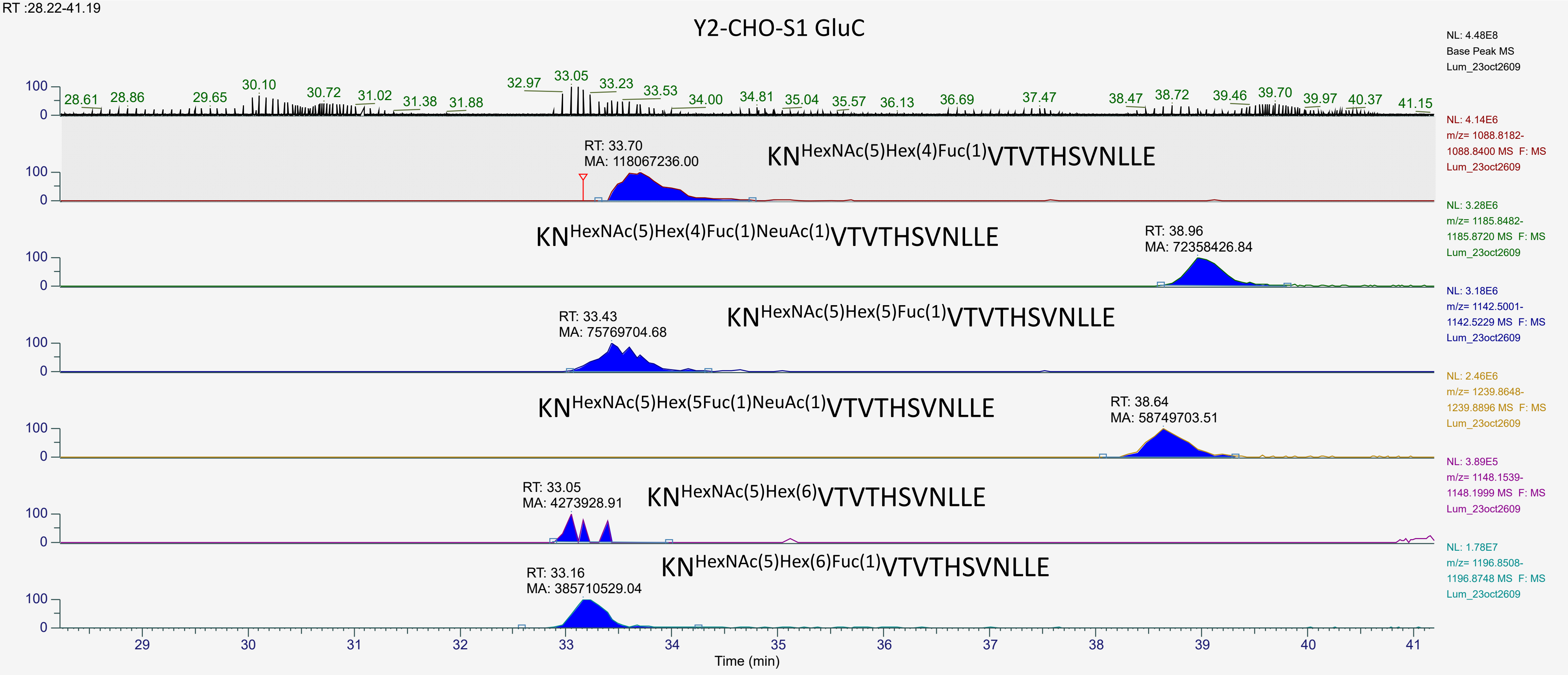

RT (Min)	Base Peak	Peak Area	Peak Height	Baseline Width	SignalToNoise
33.70175	1088.828	1.18E+08	4076477	1.458437	INF
38.96211	1185.86	72358427	3185091	1.192433	INF
33.43497	1142.512	75769705	3133895	1.302504	INF
38.63636	1239.878	58749704	2432606	1.247468	INF
33.04829	1148.179	4273929	374927.8	1.090965	INF
33.16316	1196.864	3.86E+08	17772710	1.668534	INF

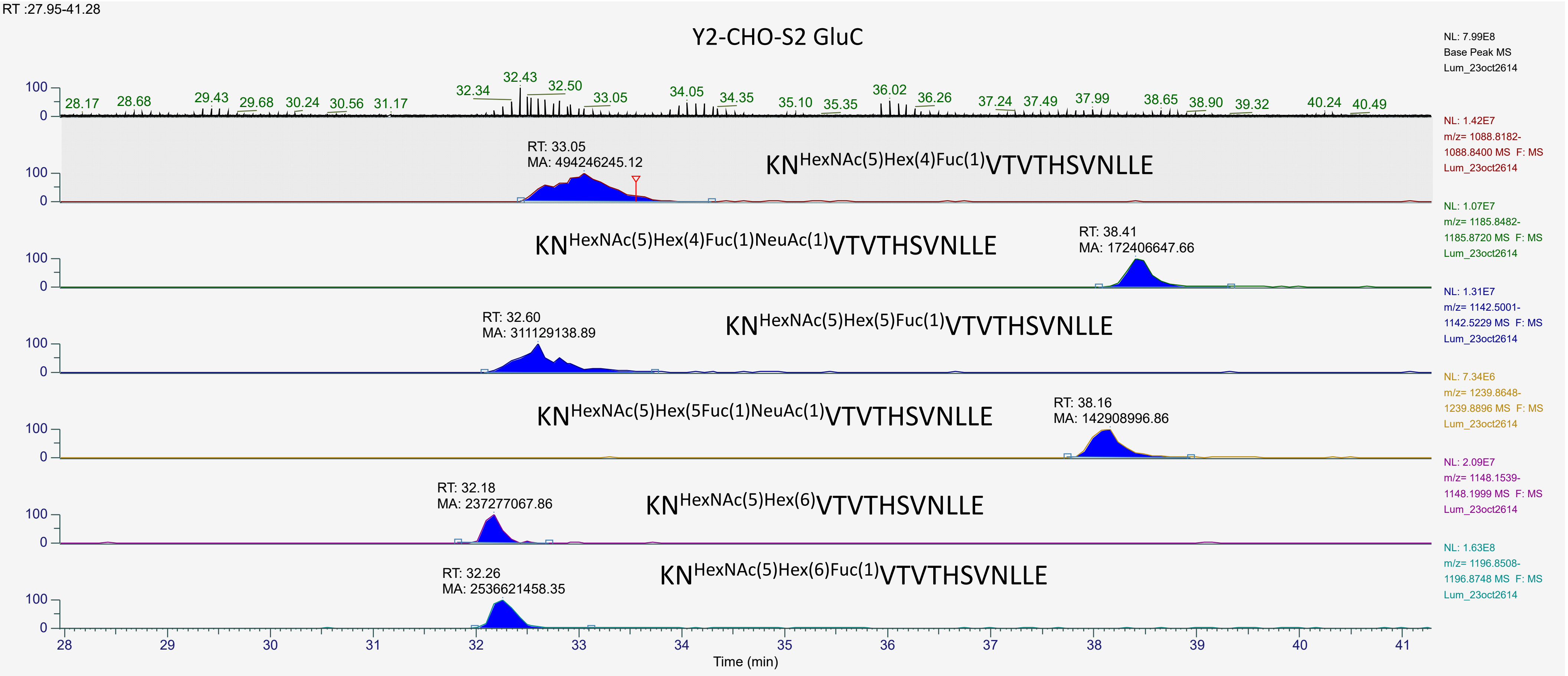

RT (Min)	Base Peak	Peak Area	Peak Height	Baseline Width	SignalToNoise
33.05139	1088.828	4.94E+08	13775170	1.85715	INF
38.40533	1185.859	1.72E+08	10511326	1.282093	INF
32.60499	1142.512	3.11E+08	12943381	1.65918	INF
38.15748	1239.878	1.43E+08	7142477	1.197249	INF
32.17715	1148.177	2.37E+08	20331361	0.88621	INF
32.25963	1196.865	2.54E+09	1.6E+08	1.131332	INF

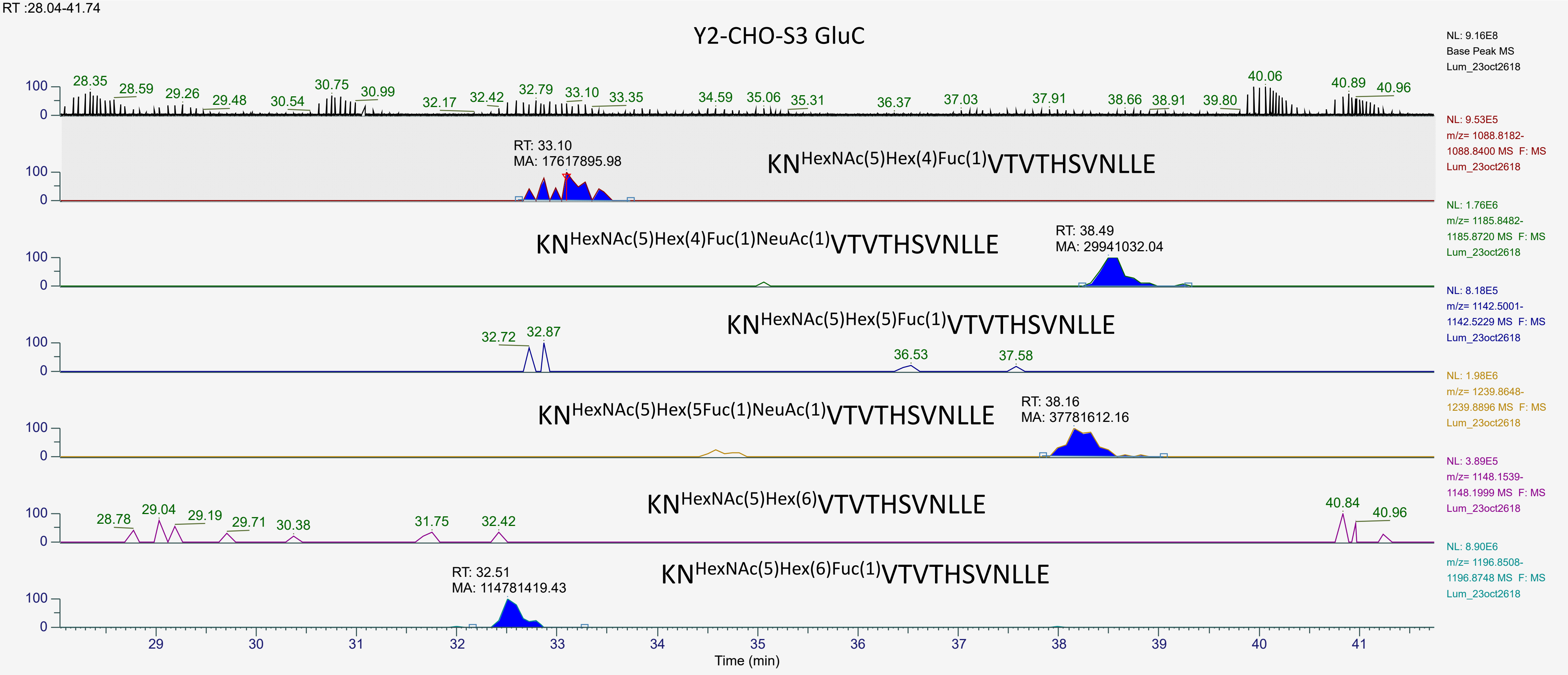

RT (Min)	Base Peak	Peak Area	Peak Height	Baseline Width	SignalToNoise
33.09652	1088.829	17617896	930976.2	1.114215	INF
38.49487	1185.86	29941032	1759320	1.056082	INF
38.15793	1239.878	37781612	1916602	1.201297	INF
32.506	1196.865	1.15E+08	8900314	1.114106	INF

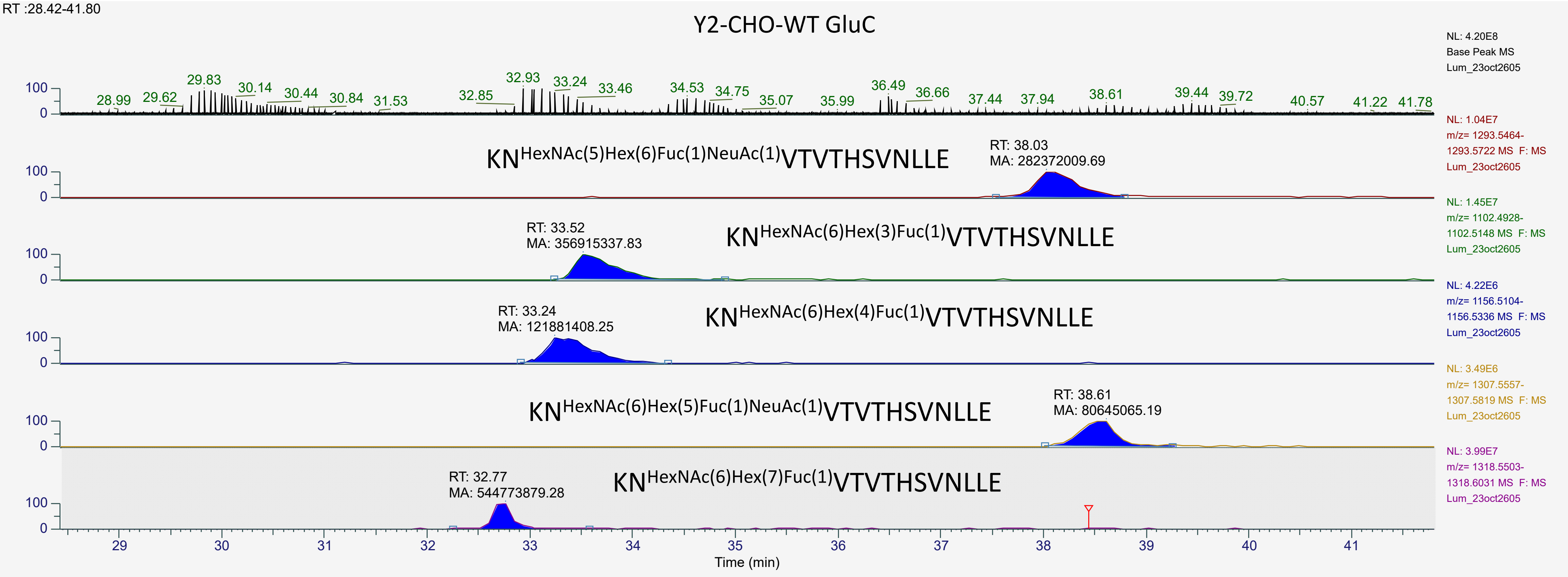

RT (Min)	Base Peak	Peak Area	Peak Height	Baseline Width	SignalToNoise
38.0277	1293.561	2.82E+08	10219677	1.249052	INF
33.51813	1102.504	3.57E+08	14008918	1.655941	INF
33.24426	1156.521	1.22E+08	4072216	1.438303	INF
38.60799	1307.57	80645065	3366440	1.23959	INF
32.76532	1318.575	5.45E+08	39871444	1.334215	INF

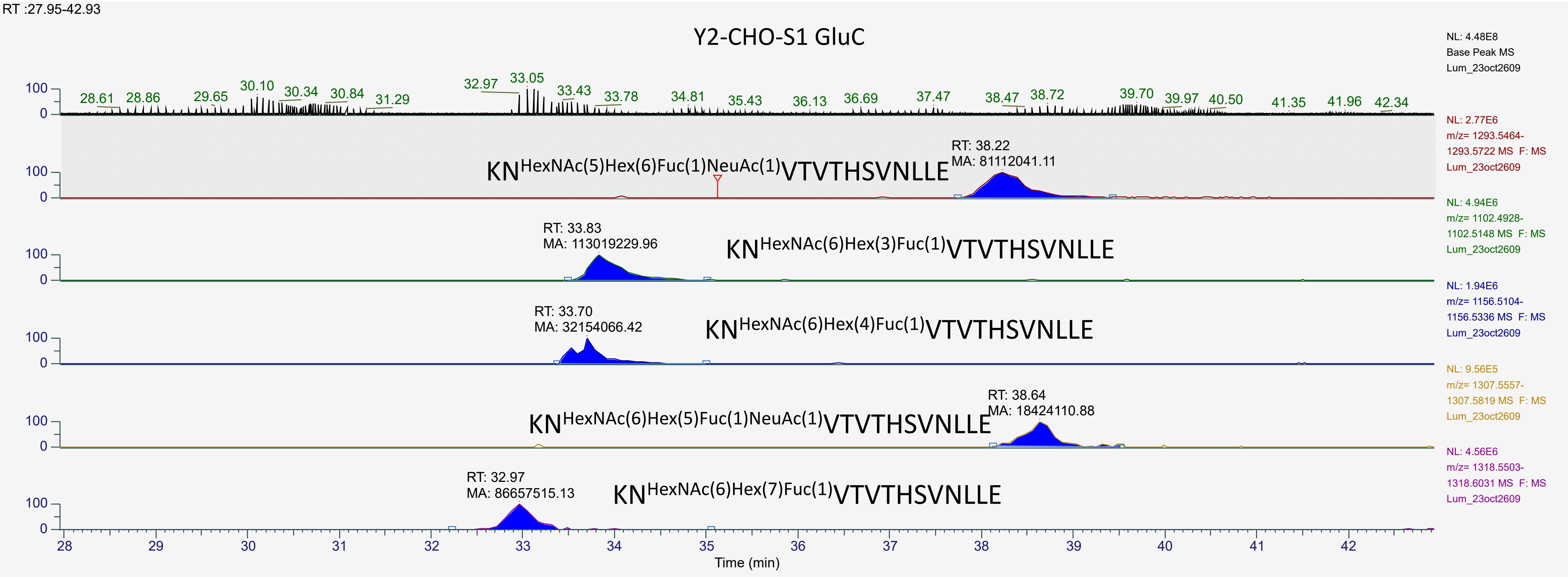

RT (Min)	Base Peak	Peak Area	Peak Height	Baseline Width	SignalToNoise
38.2225	1293.561	81112041	2728746	1.684455	INF
33.83106	1102.504	1.13E+08	4937934	1.51495	INF
33.70175	1156.522	32154066	1906592	1.620891	INF
38.63636	1307.572	18424111	908076.3	1.388365	INF
32.96527	1318.574	86657515	4559016	2.82972	INF

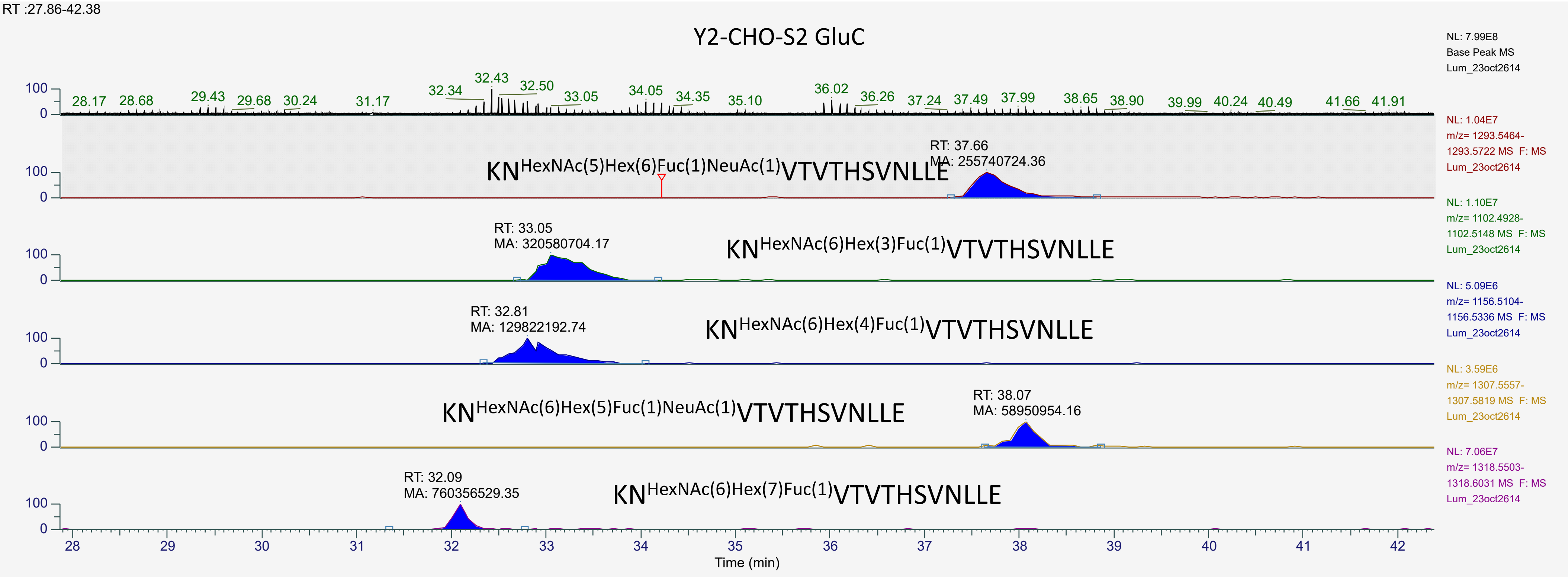

RT (Min)	Base Peak	Peak Area	Peak Height	Baseline Width	SignalToNoise
37.65968	1293.56	2.56E+08	10189800	1.540311	INF
33.05139	1102.504	3.21E+08	10840310	1.499236	INF
32.806	1156.519	1.3E+08	4922022	1.704611	INF
38.07399	1307.569	58950954	3542224	1.222488	INF
32.09419	1318.574	7.6E+08	70584472	1.427949	INF

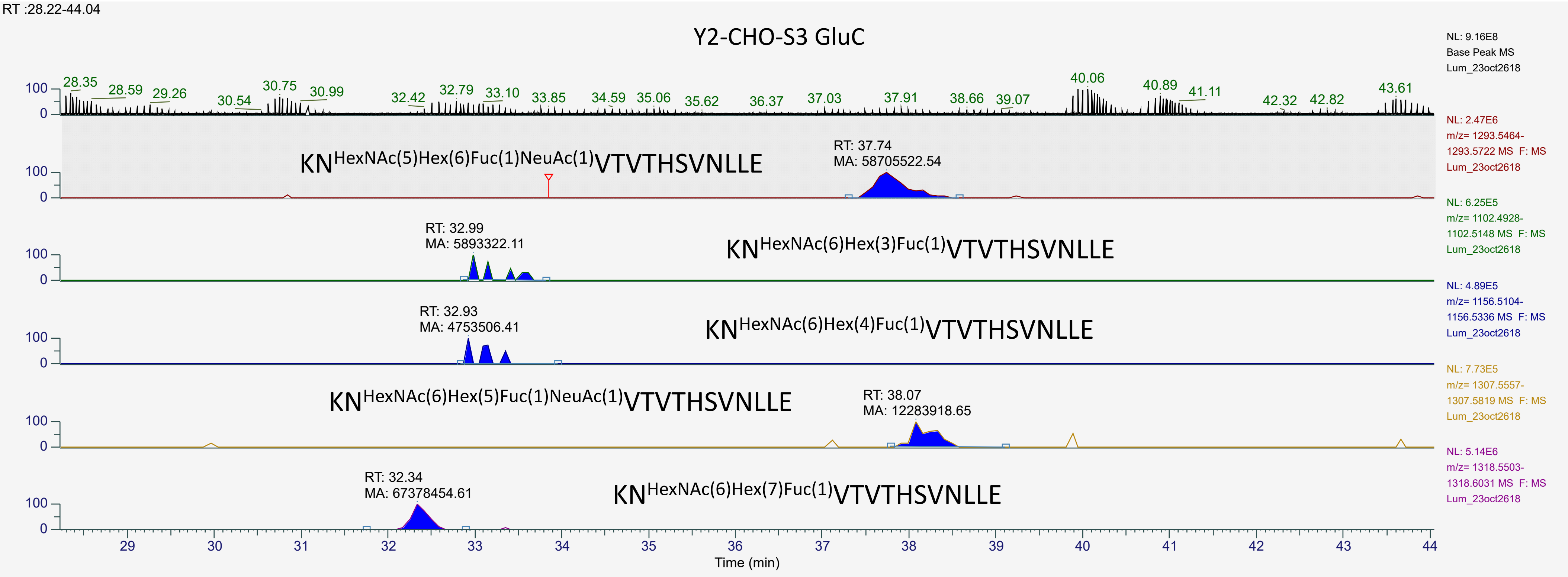

RT (Min)	Base Peak	Peak Area	Peak Height	Baseline Width	SignalToNoise
37.74304	1293.561	58705523	2432905	1.275446	INF
32.98706	1102.502	5893322	588745.8	0.939802	INF
32.92601	1156.516	4753506	478503.1	1.118812	INF
38.07456	1307.57	12283919	745963.3	1.319632	INF
32.33844	1318.575	67378455	5141757	1.140699	INF

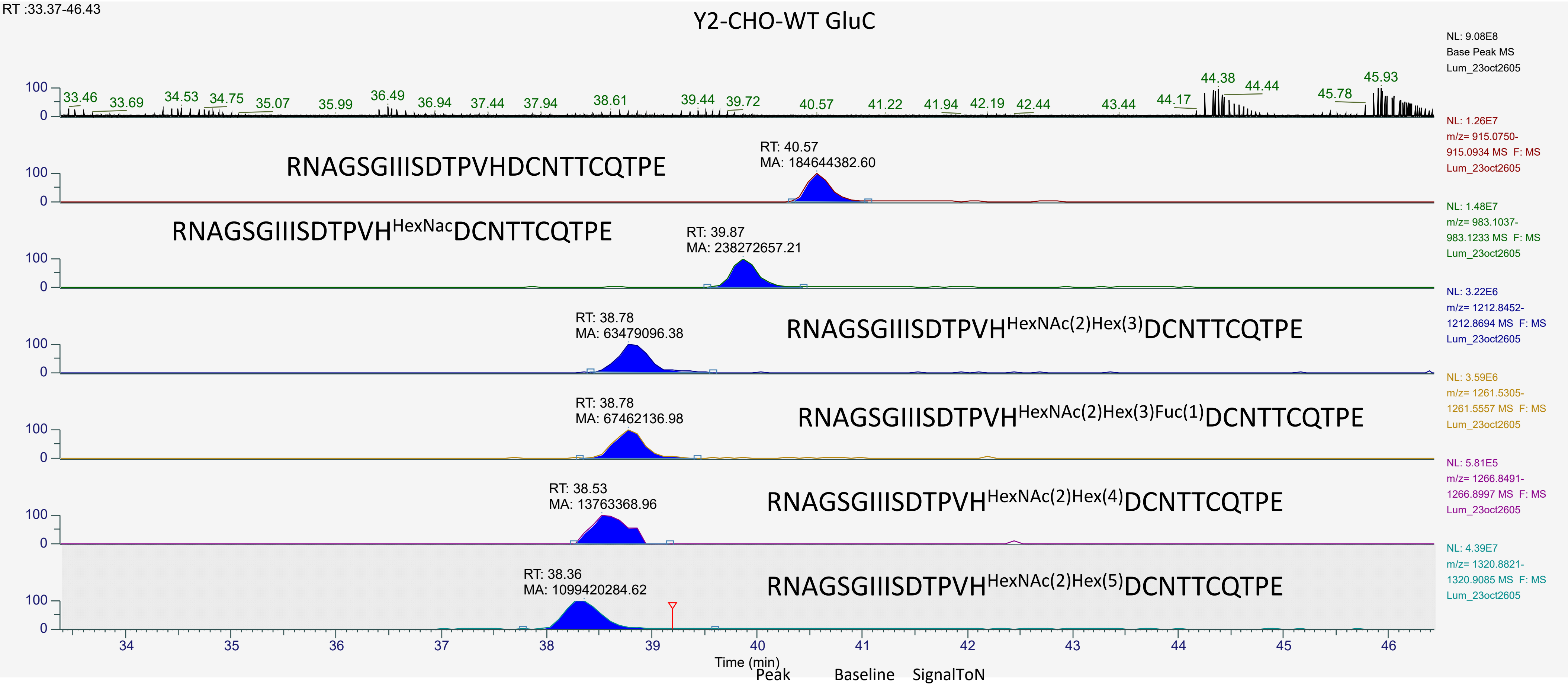

RT (Min)	Base Peak	Peak Area	Peak Height	Baseline Width	SignalToNoise
40.57047	915.0862	1.85E+08	12313698	0.729661	INF
39.86622	983.1135	2.38E+08	14615847	0.914385	INF
38.77565	1212.858	63479096	3123751	1.163762	INF
38.77565	1261.543	67462137	3543972	1.117581	INF
38.52526	1266.876	13763369	572452.7	0.914385	INF
38.35877	1320.895	1.1E+09	43878424	1.828769	INF

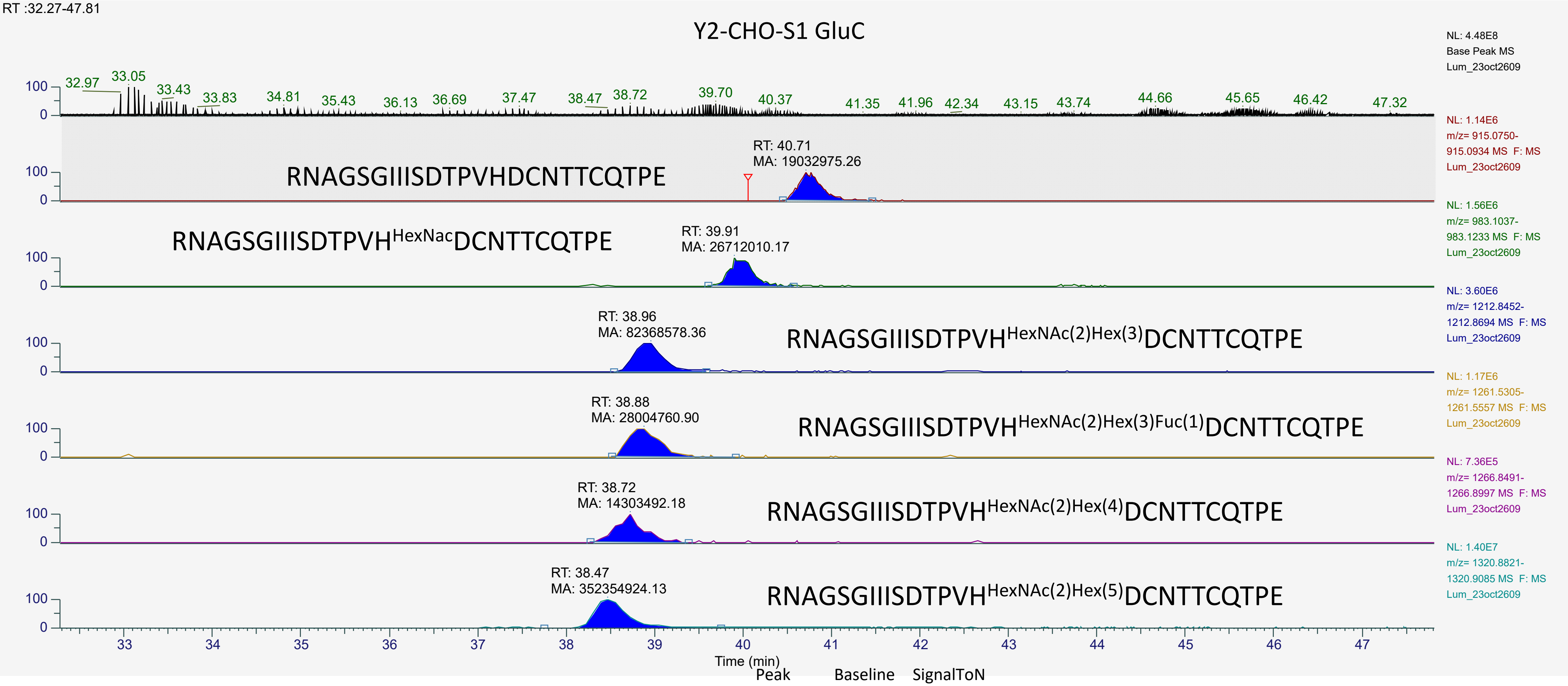

RT (Min)	Base Peak	Peak Area	Peak Height	Baseline Width	SignalToNoise
40.71111	915.0861	19032975	1109172	1.011089	INF
39.90657	983.1135	26712010	1518976	0.967129	INF
38.96211	1212.859	82368578	3600269	1.044059	INF
38.87903	1261.544	28004761	1135711	1.395743	INF
38.71968	1266.875	14303492	717576.9	1.109821	INF
38.47277	1320.895	3.52E+08	13967729	1.999875	INF

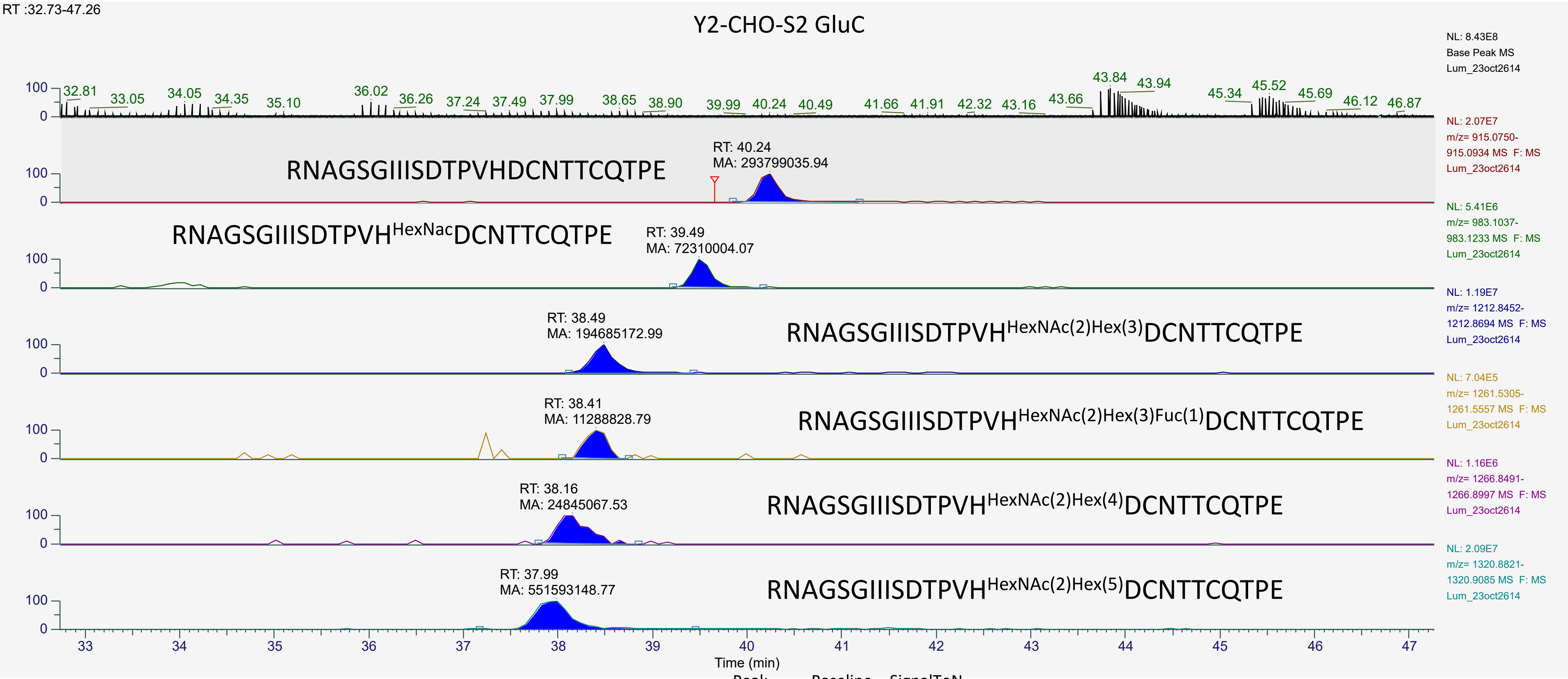

RT (Min)	Base Peak	Peak Area	Peak Height	Baseline Width	SignalToNoise
40.24317	915.0854	2.94E+08	20104330	1.346132	INF
39.4902	983.1125	72310004	5252213	0.955651	INF
38.4882	1212.858	1.95E+08	11939350	1.315304	INF
38.40533	1261.544	11288829	682572.3	0.698755	INF
38.15748	1266.875	24845068	1113735	1.05812	INF
37.99063	1320.892	5.52E+08	20900664	2.280609	INF

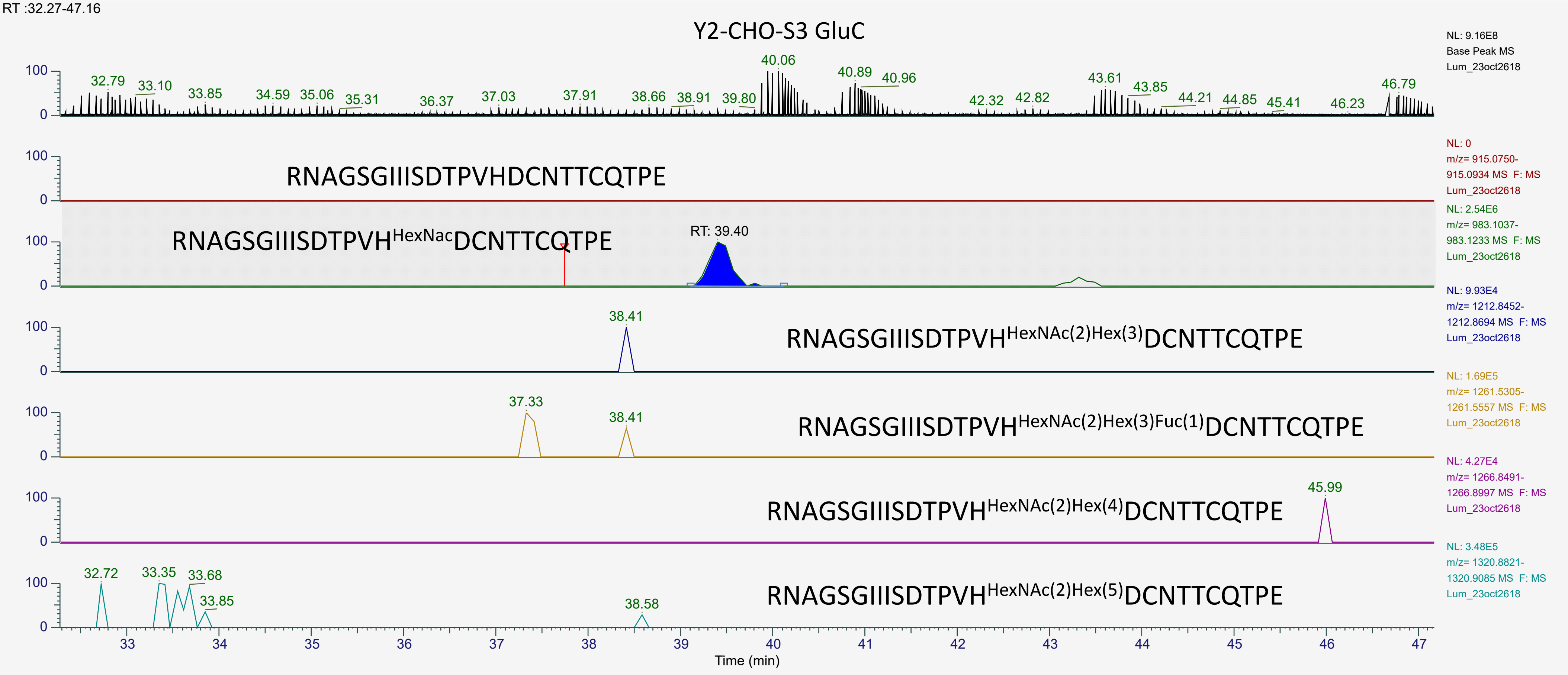

RT (Min)	Base Peak	Peak Area	Peak Height	Baseline Width	SignalToNoise
39.40315	983.1122	40547458	2507206	1.01118	INF

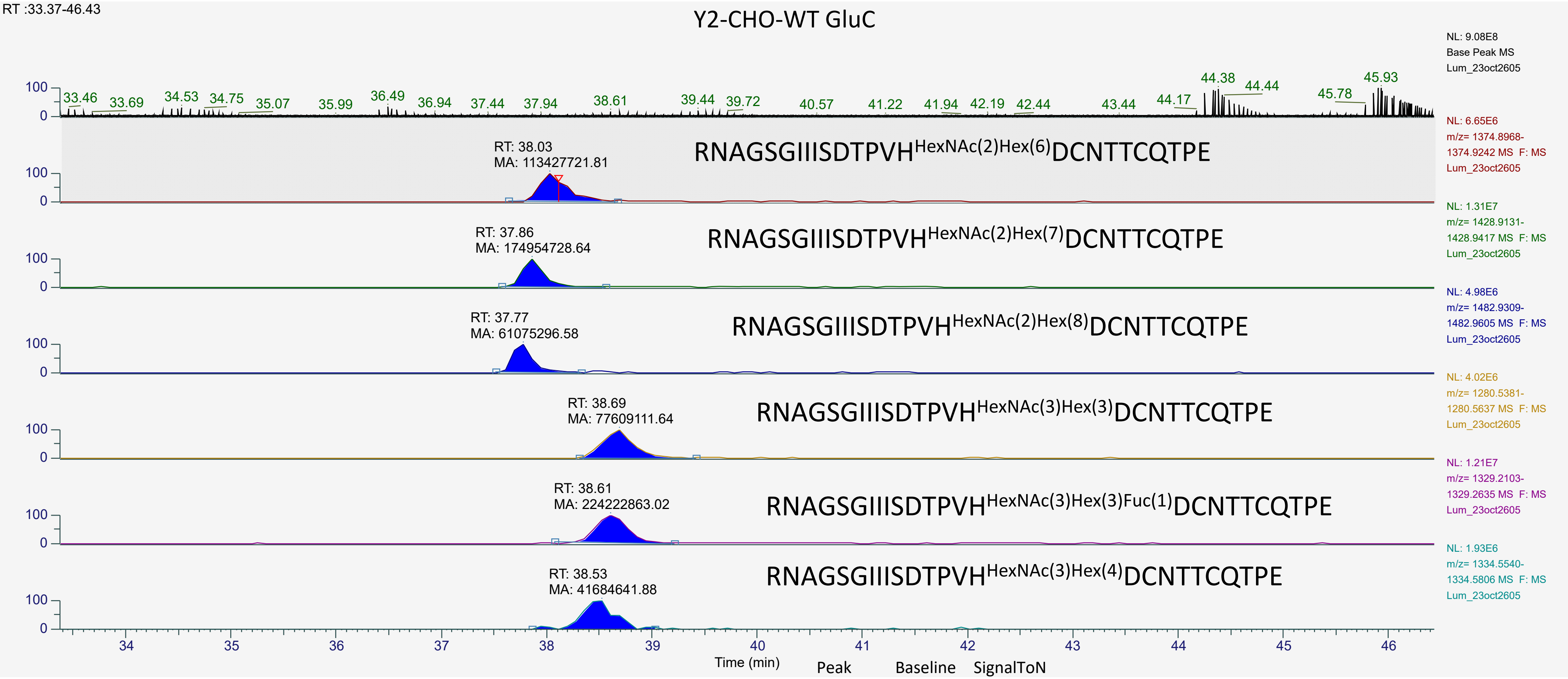

RT (Min)	Base Peak	Peak Area	Peak Height	Baseline Width	SignalToNoise
38.0277	1374.911	1.13E+08	6389091	1.034455	INF
37.85862	1428.927	1.75E+08	12692108	0.988274	INF
37.77485	1482.946	61075297	4833786	0.812786	INF
38.69196	1280.55	77609112	3967299	1.108345	INF
38.60799	1329.237	2.24E+08	11402006	1.136054	INF
38.52526	1334.569	41684642	1930525	1.172999	INF

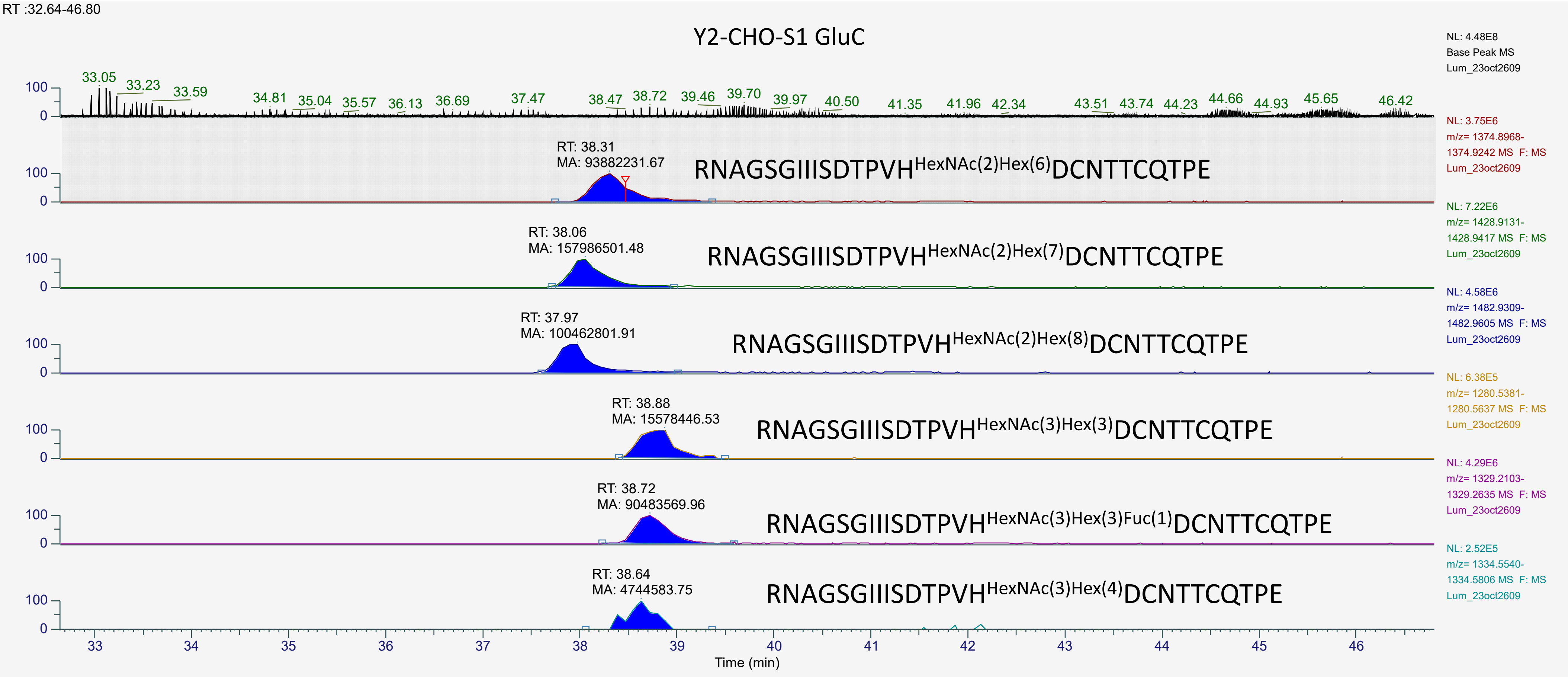

RT (Min)	Base Peak	Peak Area	Peak Height	Baseline Width	SignalToNoise
38.30617	1374.911	93882232	3700021	1.612277	INF
38.05539	1428.931	1.58E+08	7004272	1.251768	INF
37.97189	1482.946	1E+08	4512724	1.411994	INF
38.87903	1280.552	15578447	623118.5	1.091542	INF
38.71968	1329.238	90483570	4118690	1.361772	INF
38.63636	1334.57	4744584	251935.8	1.301694	INF

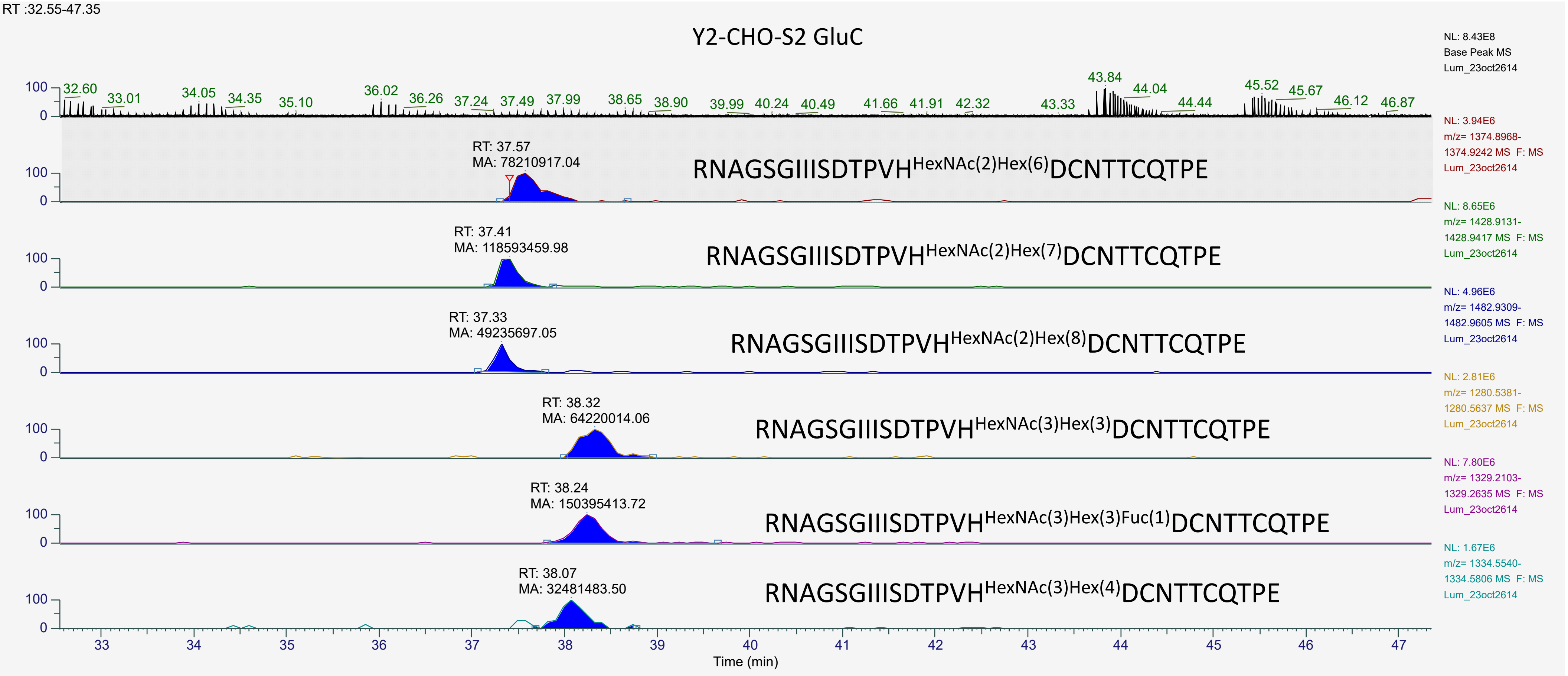

RT (Min)	Base Peak	Peak Area	Peak Height	Baseline Width	SignalToNoise
37.57455	1374.91	78210917	3874395	1.371146	INF
37.40823	1428.927	1.19E+08	8526455	0.71174	INF
37.32583	1482.945	49235697	4763893	0.722207	INF
38.32296	1280.551	64220014	2808587	0.962942	INF
38.23988	1329.237	1.5E+08	7675500	1.84238	INF
38.07399	1334.568	32481484	1670612	1.088679	INF

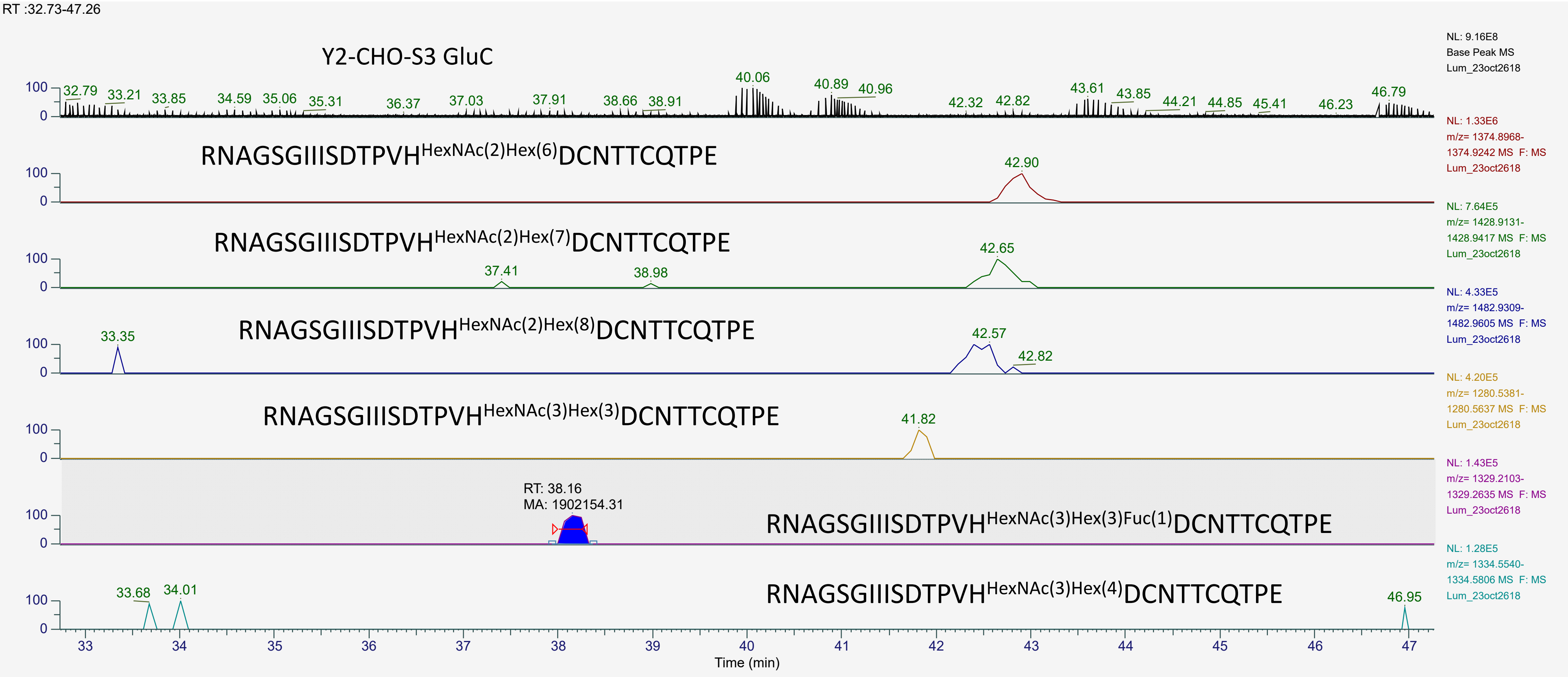

RT (Min)	Base Peak	Peak Area	Peak Height	Baseline Width	SignalToNoise
38.15793	1329.242	1902154	140492.7	0.431468	INF

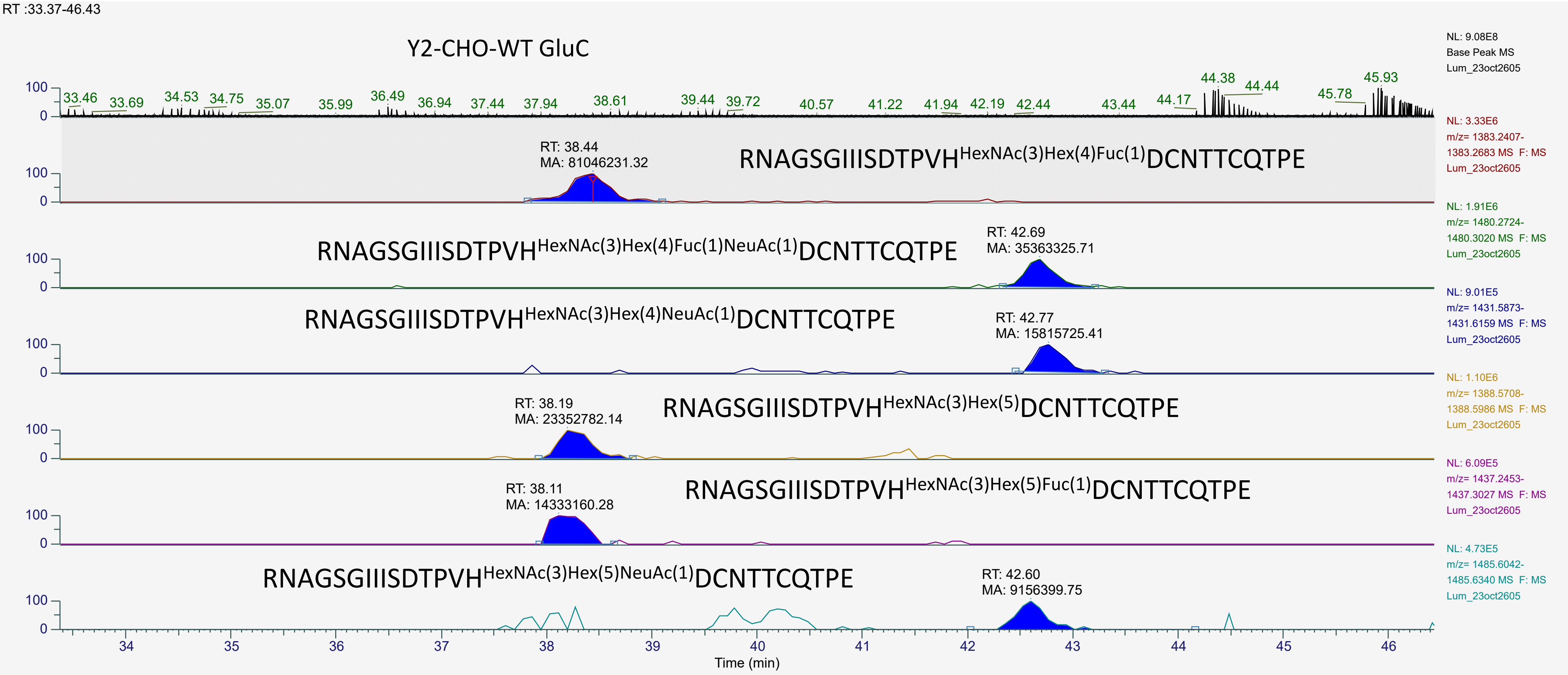

RT (Min)	Base Peak	Peak Area	Peak Height	Baseline Width	SignalToNoise
38.44246	1383.253	81046231	3258227	1.283833	INF
42.68719	1480.288	35363326	1861566	0.87744	INF
42.77079	1431.602	15815725	854307.9	0.849731	INF
38.19299	1388.585	23352782	1085054	0.895912	INF
38.11027	1437.274	14333160	600057.8	0.711188	INF
42.60447	1485.62	9156400	473416.3	2.133564	INF

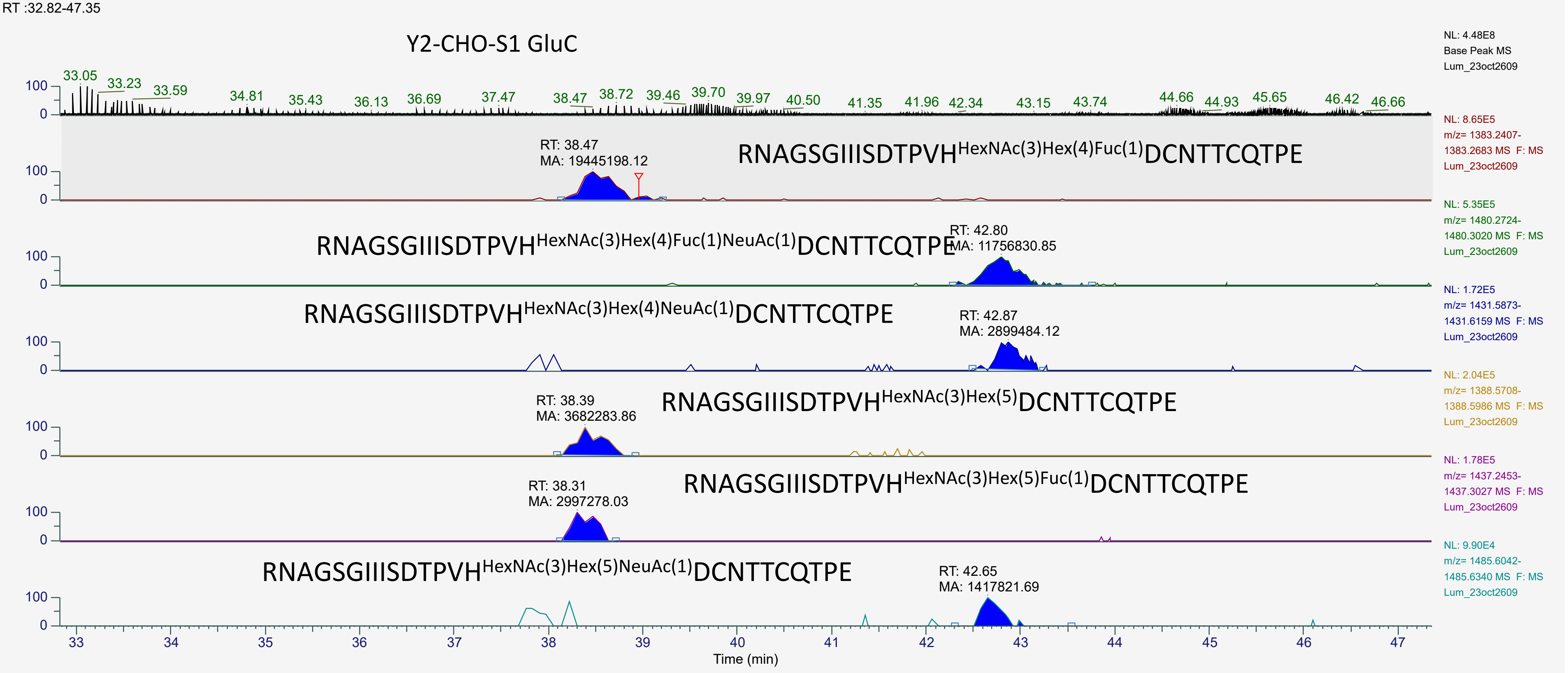

RT (Min)	Base Peak	Peak Area	Peak Height	Baseline Width	SignalToNoise
38.47277	1383.255	19445198	852700.3	1.07896	INF
42.7953	1480.287	11756831	528200.7	1.469441	INF
42.87006	1431.601	2899484	165166.3	0.750134	INF
38.38945	1388.59	3682284	198910.6	0.822065	INF
38.30617	1437.272	2997278	175180.9	0.585567	INF
42.65456	1485.617	1417822	99030.55	1.232773	INF

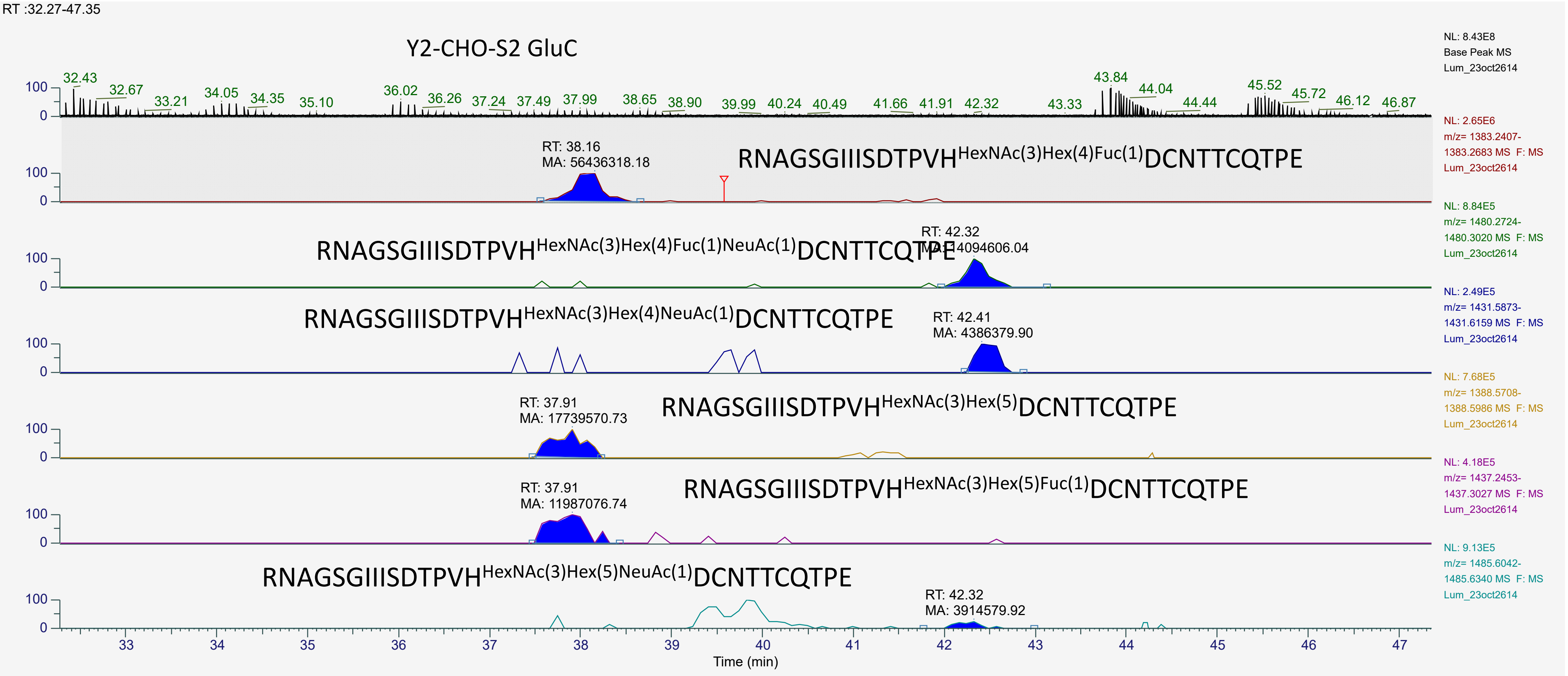

RT (Min)	Base Peak	Peak Area	Peak Height	Baseline Width	SignalToNoise
38.15748	1383.255	56436318	2599813	1.098472	INF
42.32436	1480.287	14094606	871642.6	1.173126	INF
42.40792	1431.601	4386380	241608.8	0.639887	INF
37.9078	1388.584	17739571	748334.6	0.757199	INF
37.9078	1437.271	11987077	413800.1	0.959681	INF
42.32436	1485.621	3914580	203015.5	1.22626	INF

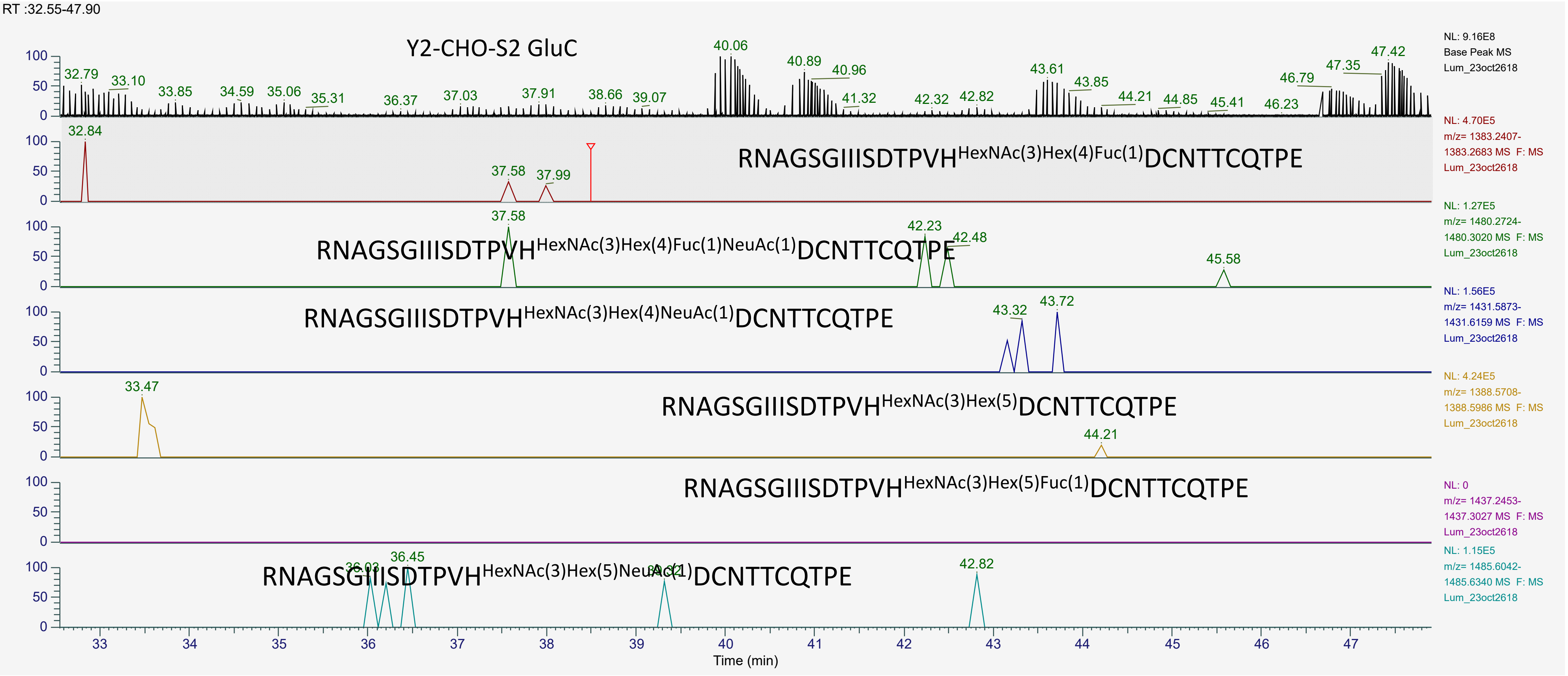
RT :32.55-47.90
Y2-CHO-S2 GluC
NL: 9.16E8
Base Peak MS
Lum_23oct2618
RNAGSGIIISDTPVH HexNAc(3)Hex(4)Fuc(1) DCNTTCQTPE
NL: 4.70E5
m/z= 1383.2407-1383.2683 MS F: MS
Lum_23oct2618
RNAGSGIIISDTPVH HexNAc(3)Hex(4)Fuc(1)NeuAc(1) DCNTTCQTPE
NL: 1.27E5
m/z= 1480.2724-1480.3020 MS F: MS
Lum_23oct2618
RNAGSGIIISDTPVH HexNAc(3)Hex(4)NeuAc(1) DCNTTCQTPE
NL: 1.56E5
m/z= 1431.5873-1431.6159 MS F: MS
Lum_23oct2618
RNAGSGIIISDTPVH HexNAc(3)Hex(5) DCNTTCQTPE
NL: 4.24E5
m/z= 1388.5708-1388.5986 MS F: MS
Lum_23oct2618
RNAGSGIIISDTPVH HexNAc(3)Hex(5)Fuc(1) DCNTTCQTPE
NL: 0
m/z= 1437.2453-1437.3027 MS F: MS
Lum_23oct2618
RNAGSGIIISDTPVH HexNAc(3)Hex(5)NeuAc(1) DCNTTCQTPE
NL: 1.15E5
m/z= 1485.6042-1485.6340 MS F: MS
Lum_23oct2618
Time (min)

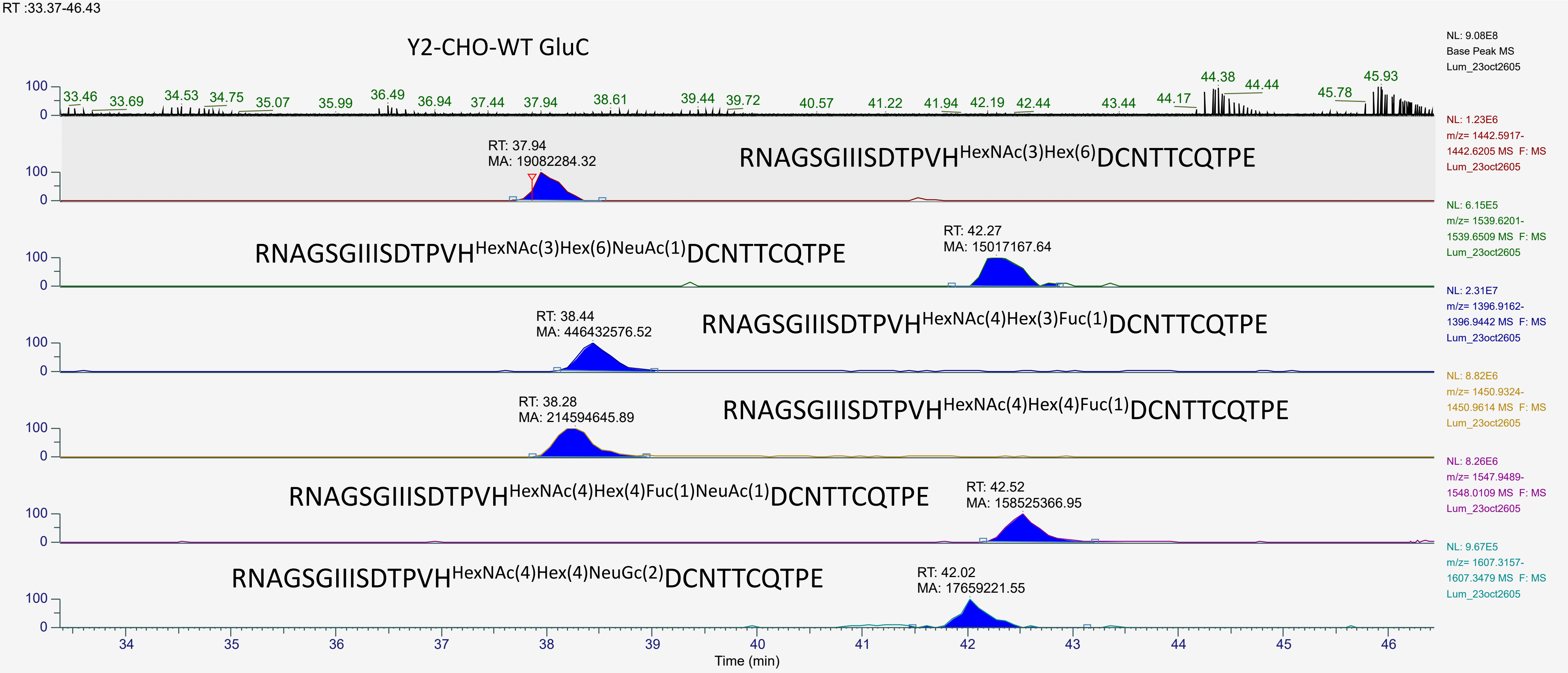

RT (Min)	Base Peak	Peak Area	Peak Height	Baseline Width	SignalToNoise
37.94213	1442.603	19082284	1190601	0.849731	INF
42.27306	1539.635	15017168	604162.2	1.025219	INF
38.44246	1396.93	4.46E+08	22251213	0.923621	INF
38.27637	1450.947	2.15E+08	8705370	1.080636	INF
42.52202	1547.98	1.59E+08	8033534	1.062164	INF
42.02099	1607.332	17659222	967356.4	1.662518	INF

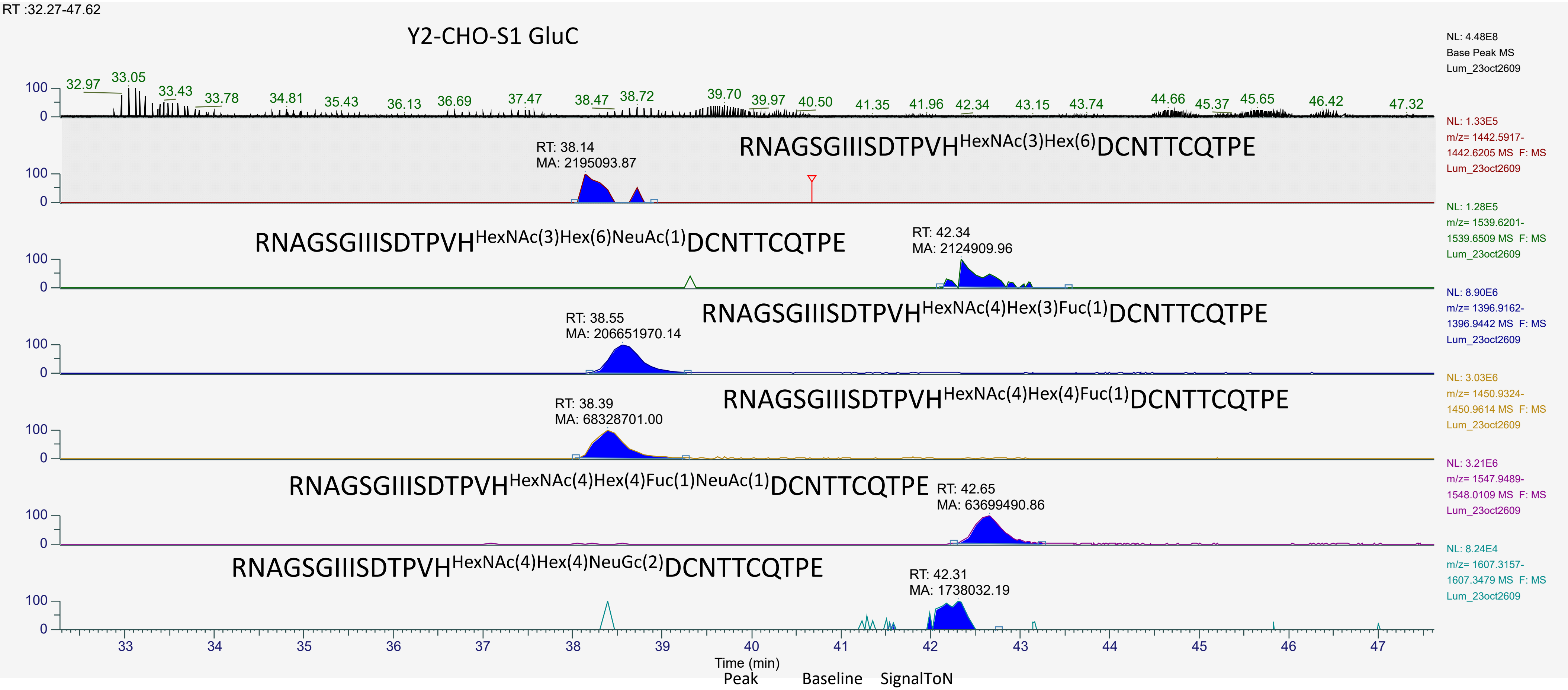

RT (Min)	Base Peak	Peak Area	Peak Height	Baseline Width	SignalToNoise
38.13905	1442.601	2195094	130958.1	0.89017	INF
42.34436	1539.637	2124910	123209.5	1.443812	INF
38.55266	1396.931	2.07E+08	8773329	1.096429	INF
38.38945	1450.949	68328701	2944555	1.226697	INF
42.65456	1547.98	63699491	3087893	0.988104	INF
42.30866	1607.336	1738032	82405.07	1.205269	INF

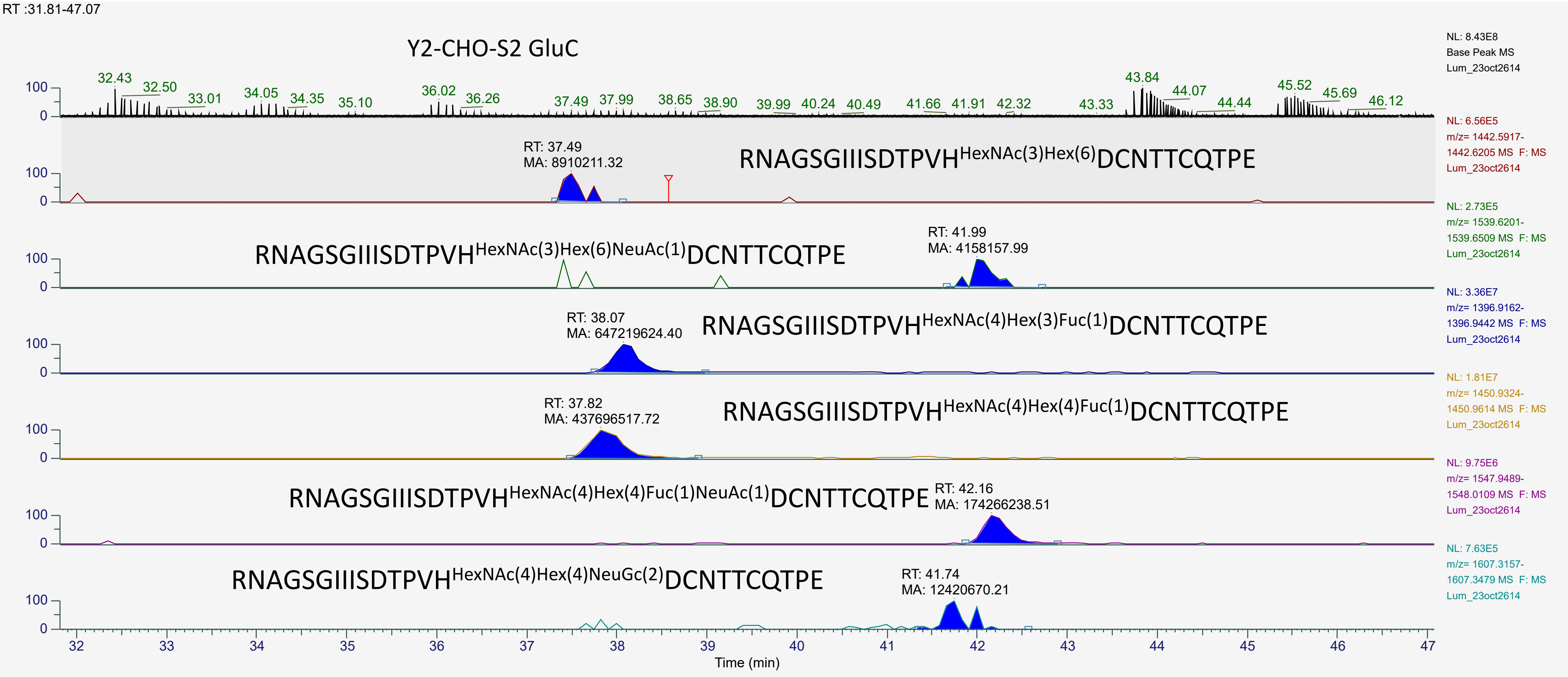

RT (Min)	Base Peak	Peak Area	Peak Height	Baseline Width	SignalToNoise
37.49044	1442.606	8910211	624877.2	0.744653	INF
41.99305	1539.632	4158158	261089	1.046832	INF
38.07399	1396.93	6.47E+08	32542774	1.230297	INF
37.82492	1450.948	4.38E+08	17766426	1.424554	INF
42.15826	1547.979	1.74E+08	9455540	1.025351	INF
41.74432	1607.332	12420670	763487.6	1.219628	INF

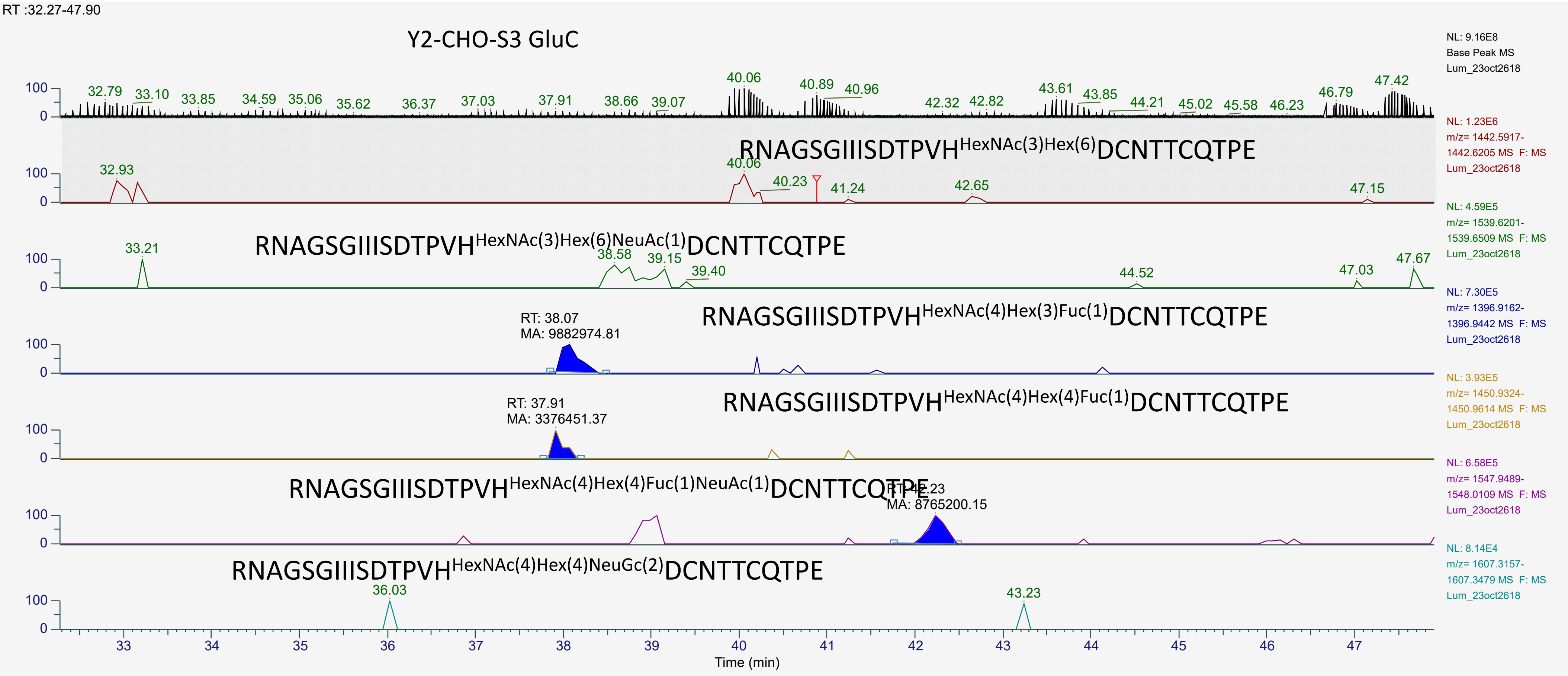

RT (Min)	Base Peak	Peak Area	Peak Height	Baseline Width	SignalToNoise
38.07456	1396.93	9882975	691714.1	0.641117	INF
37.90917	1450.947	3376451	387218.7	0.431078	INF
42.2325	1547.982	8765200	644026.9	0.729517	INF

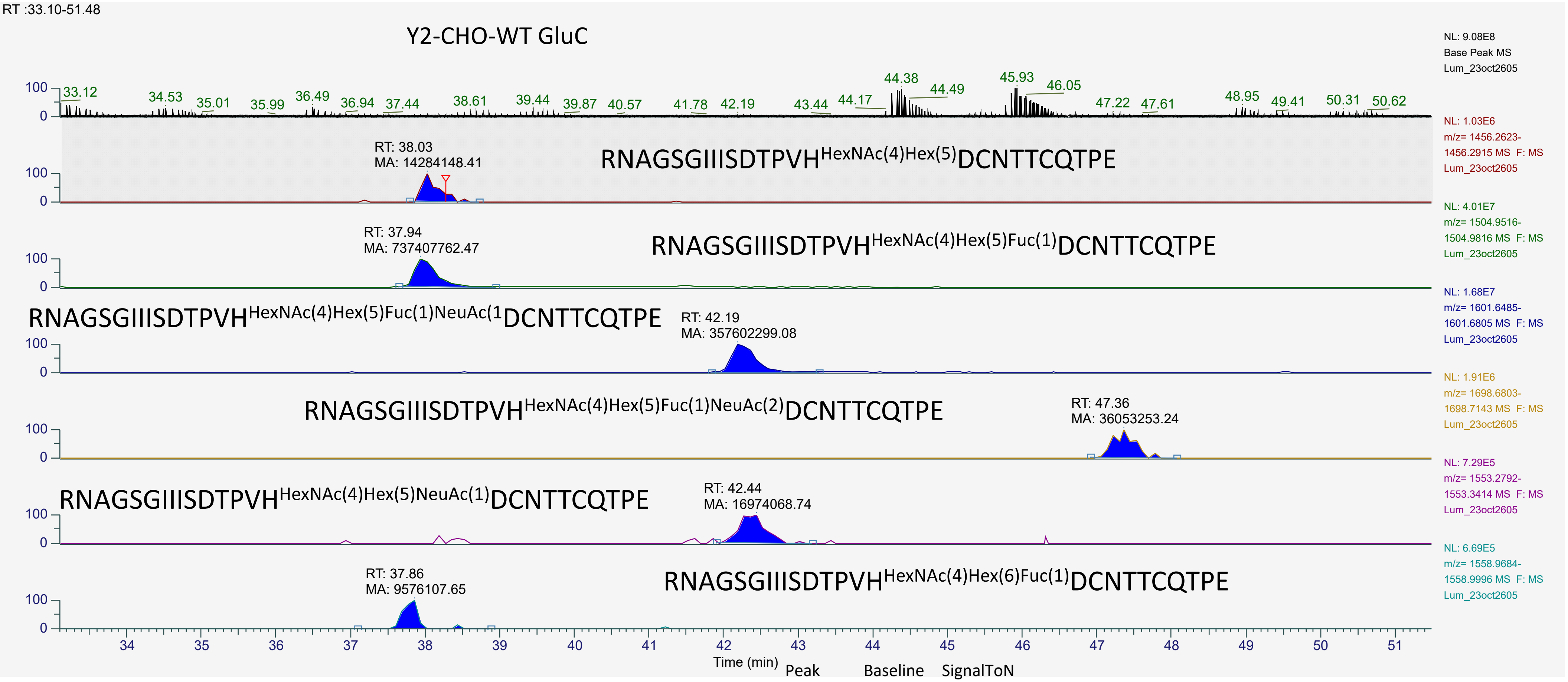

RT (Min)	Base Peak	Peak Area	Peak Height	Baseline Width	SignalToNoise
38.0277	1456.278	14284148	978538.5	0.935898	INF
37.94213	1504.966	7.37E+08	38181228	1.299859	INF
42.18846	1601.662	3.58E+08	16492939	1.442843	INF
47.36298	1698.698	36053253	1865897	1.156874	INF
42.43912	1553.31	16974069	710898.2	1.28686	INF
37.85862	1558.979	9576108	669497.3	1.780806	INF

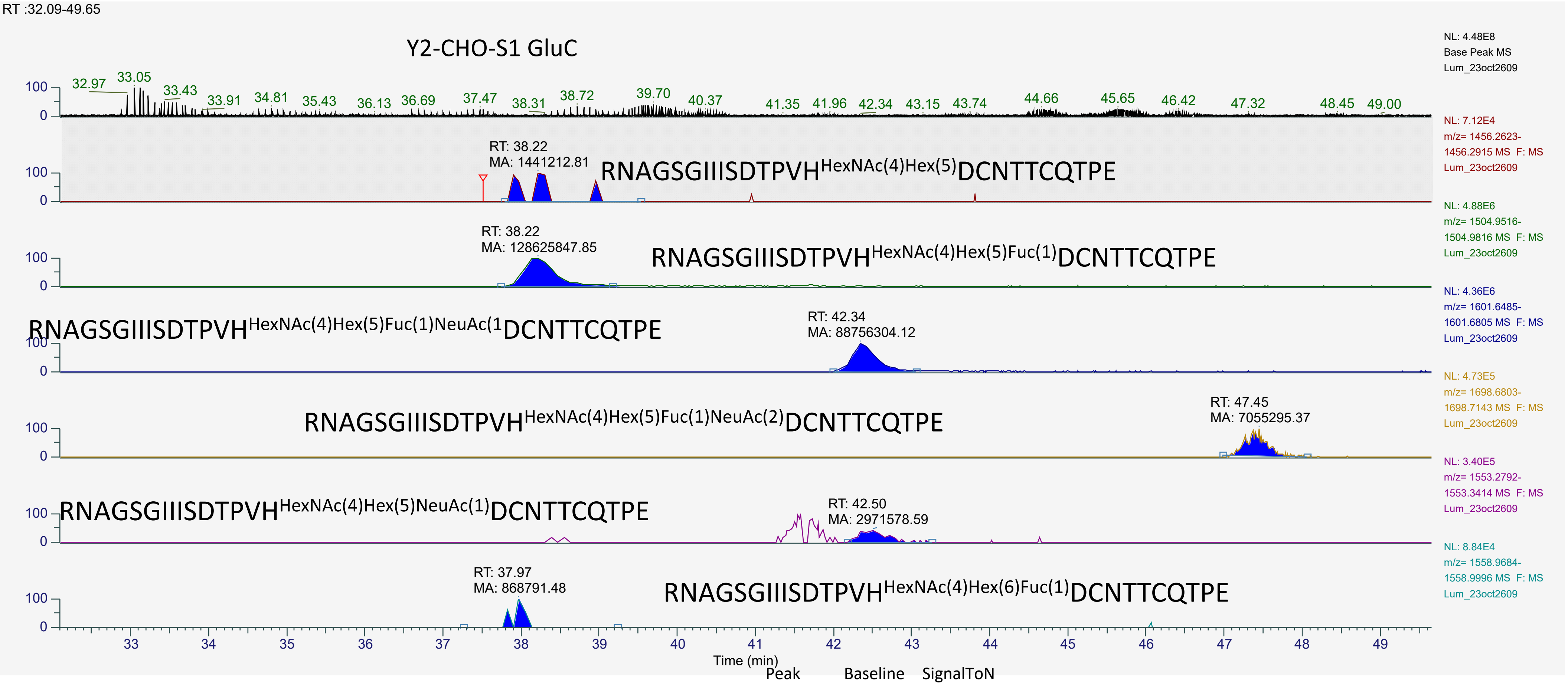

RT (Min)	Base Peak	Peak Area	Peak Height	Baseline Width	SignalToNoise
38.2225	1456.282	1441213	71200.57	1.738614	INF
38.2225	1504.967	1.29E+08	4812726	1.428147	INF
42.34436	1601.663	88756304	4297064	1.068006	INF
47.44557	1698.696	7055295	450662.5	1.080424	INF
42.5037	1553.316	2971579	126553.8	1.080432	INF
37.97189	1558.984	868791.5	88368.86	1.962163	INF

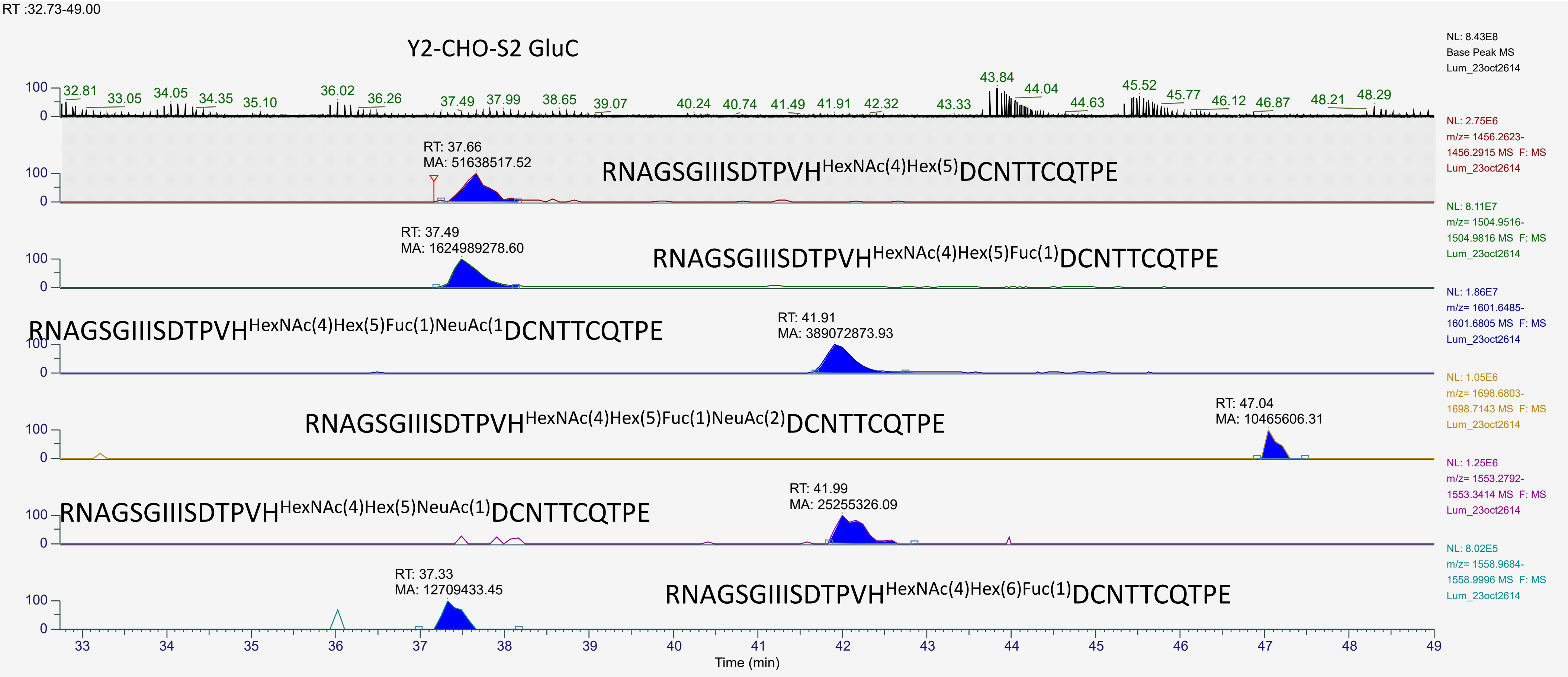

RT (Min)	Base Peak	Peak Area	Peak Height	Baseline Width	SignalToNoise
37.65968	1456.28	51638518	2691722	0.909003	INF
37.49044	1504.966	1.62E+09	80003851	0.943522	INF
41.9107	1601.663	3.89E+08	18322423	1.070092	INF
47.04122	1698.694	10465606	1030361	0.563812	INF
41.99305	1553.311	25255326	1204820	1.012737	INF
37.32583	1558.984	12709433	802459.8	1.185363	INF

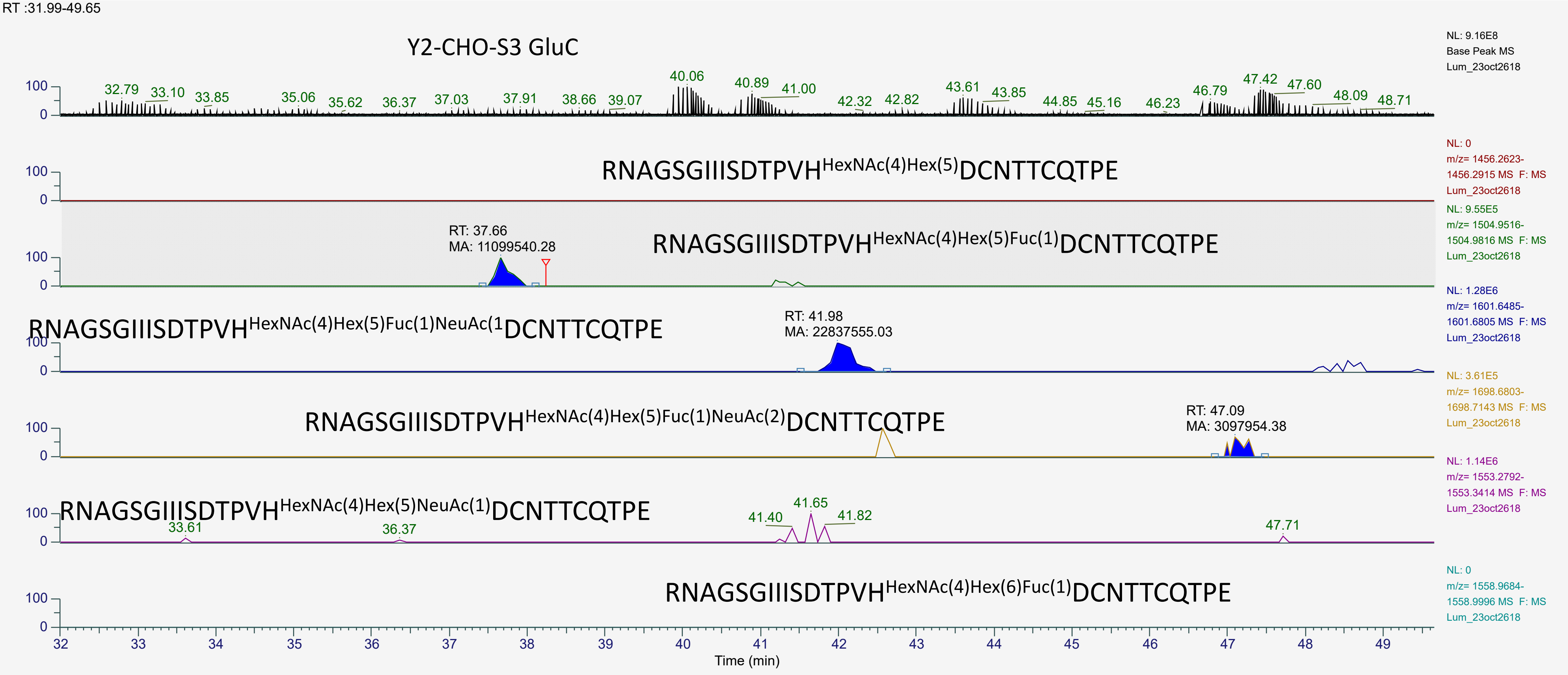

RT (Min)	Base Peak	Peak Area	Peak Height	Baseline Width	SignalToNoise
37.66085	1504.969	11099540	942259.6	0.674427	INF
41.98253	1601.665	22837555	1265412	1.11105	INF
47.09127	1698.702	3097954	231883.1	0.649153	INF

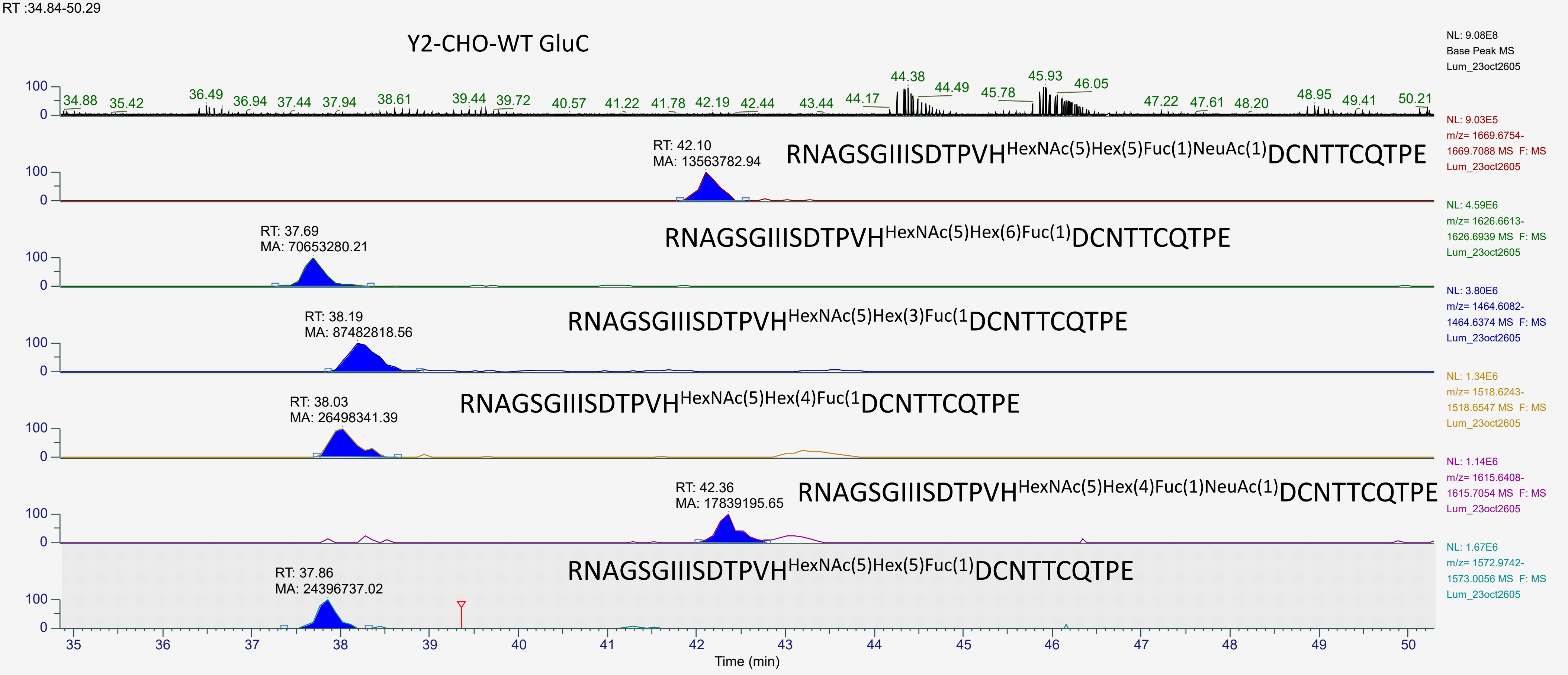

RT (Min)	Base Peak	Peak Area	Peak Height	Baseline Width	SignalToNoise
42.10481	1669.692	13563783	902771.3	0.732072	INF
37.69101	1626.678	70653280	4591922	1.070792	INF
38.19299	1464.623	87482819	3802081	1.027086	INF
38.0277	1518.639	26498341	1302062	0.917822	INF
42.35619	1615.673	17839196	1126170	0.775778	INF
37.85862	1572.991	24396737	1667762	0.950601	INF

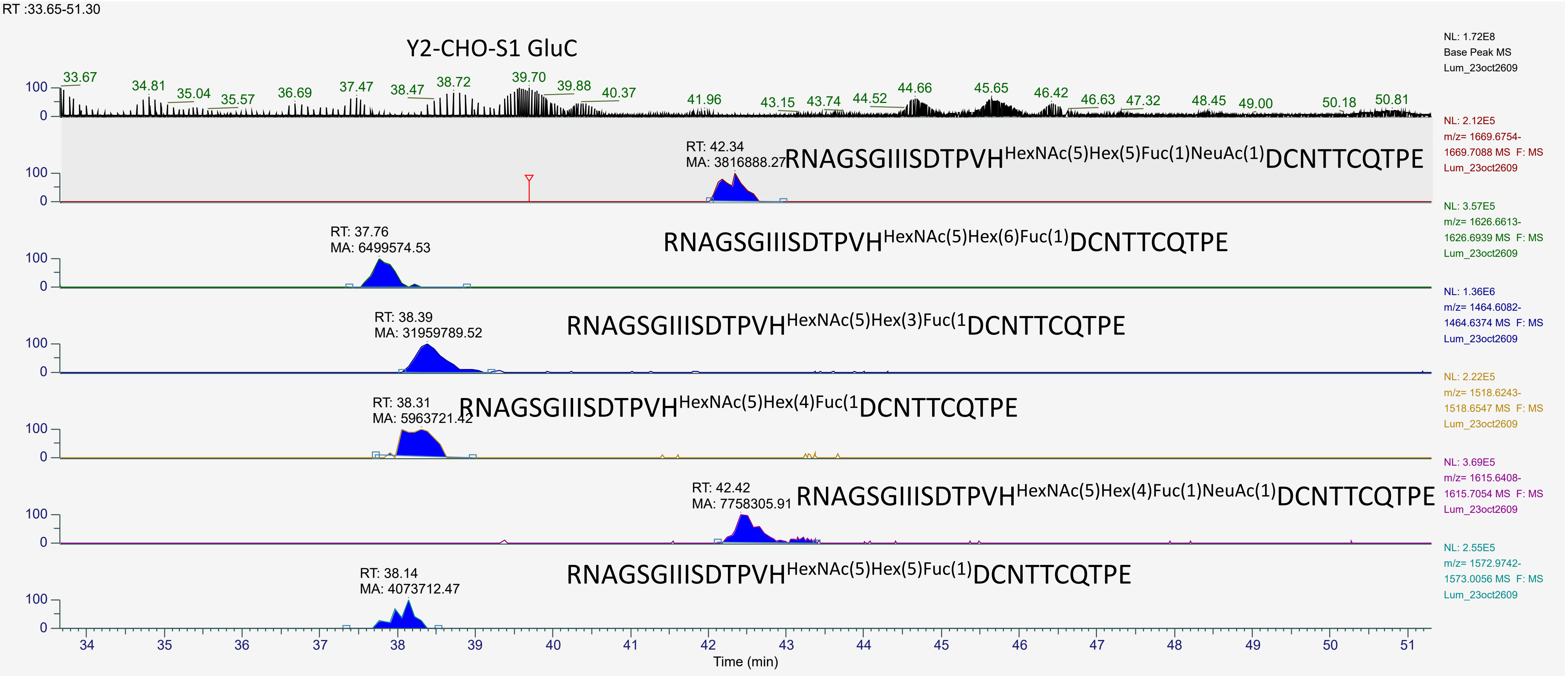

RT (Min)	Base Peak	Peak Area	Peak Height	Baseline Width	SignalToNoise
42.34436	1669.691	3816888	205904.6	0.948656	INF
37.76215	1626.68	6499575	356659.2	1.522843	INF
38.38945	1464.624	31959790	1339943	1.148373	INF
38.30617	1518.636	5963721	207143.6	1.248232	INF
42.42034	1615.671	7758306	357082.1	1.273345	INF
38.13905	1572.994	4073712	254769.9	1.185959	INF

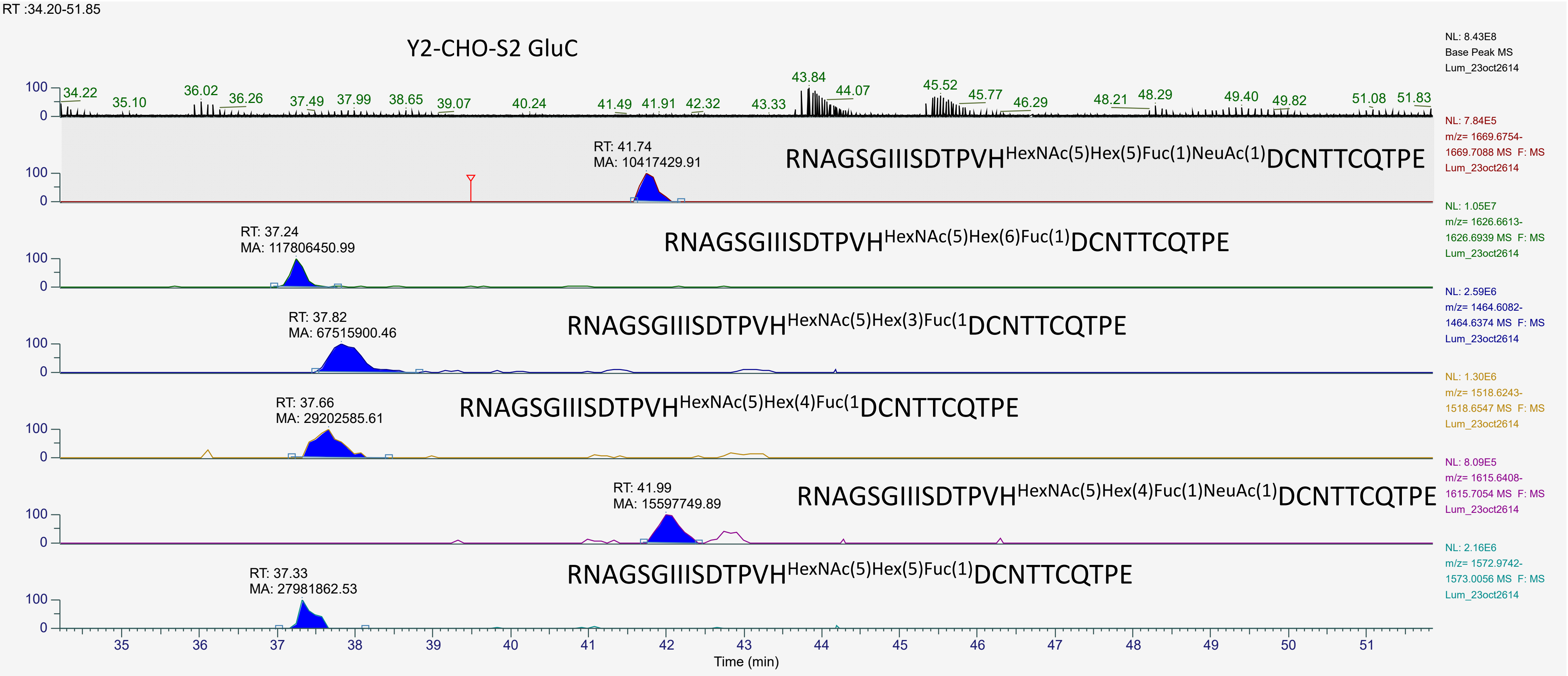

RT (Min)	Base Peak	Peak Area	Peak Height	Baseline Width	SignalToNoise
41.74432	1669.695	10417430	747939.2	0.611634	INF
37.24248	1626.677	1.18E+08	10262481	0.811351	INF
37.82492	1464.623	67515900	2510127	1.335608	INF
37.65968	1518.64	29202586	1251840	1.248232	INF
41.99305	1615.672	15597750	789385.9	0.711569	INF
37.32583	1572.994	27981863	2164546	1.111046	INF

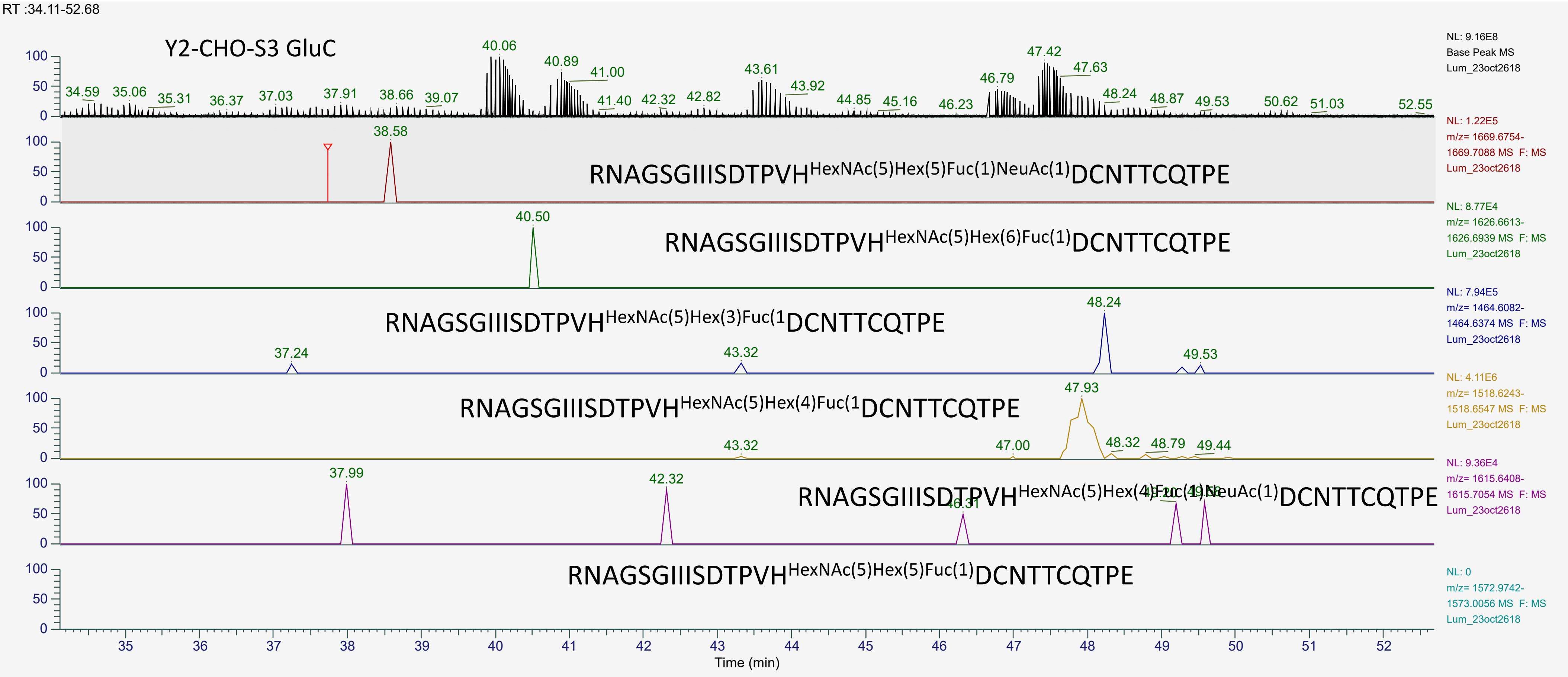
RT :34.11-52.68
Y2-CHO-S3 GluC
NL: 9.16E8
Base Peak MS
Lum_23oct2618
RNAGSGIIISDTPVH HexNAc(5)Hex(5)Fuc(1)NeuAc(1) DCNTTCQTPE
NL: 1.22E5
m/z= 1669.6754-1669.7088 MS F: MS
Lum_23oct2618
RNAGSGIIISDTPVH HexNAc(5)Hex(6)Fuc(1) DCNTTCQTPE
NL: 8.77E4
m/z= 1626.6613-1626.6939 MS F: MS
Lum_23oct2618
RNAGSGIIISDTPVH HexNAc(5)Hex(3)Fuc(1 DCNTTCQTPE
NL: 7.94E5
m/z= 1464.6082-1464.6374 MS F: MS
Lum_23oct2618
RNAGSGIIISDTPVH HexNAc(5)Hex(4)Fuc(1 DCNTTCQTPE
NL: 4.11E6
m/z= 1518.6243-1518.6547 MS F: MS
Lum_23oct2618
RNAGSGIIISDTPVH HexNAc(5)Hex(4)Fuc(1)NeuAc(1) DCNTTCQTPE
NL: 9.36E4
m/z= 1615.6408-1615.7054 MS F: MS
Lum_23oct2618
RNAGSGIIISDTPVH HexNAc(5)Hex(5)Fuc(1) DCNTTCQTPE
NL: 0
m/z= 1572.9742-1573.0056 MS F: MS
Lum_23oct2618
Time (min)

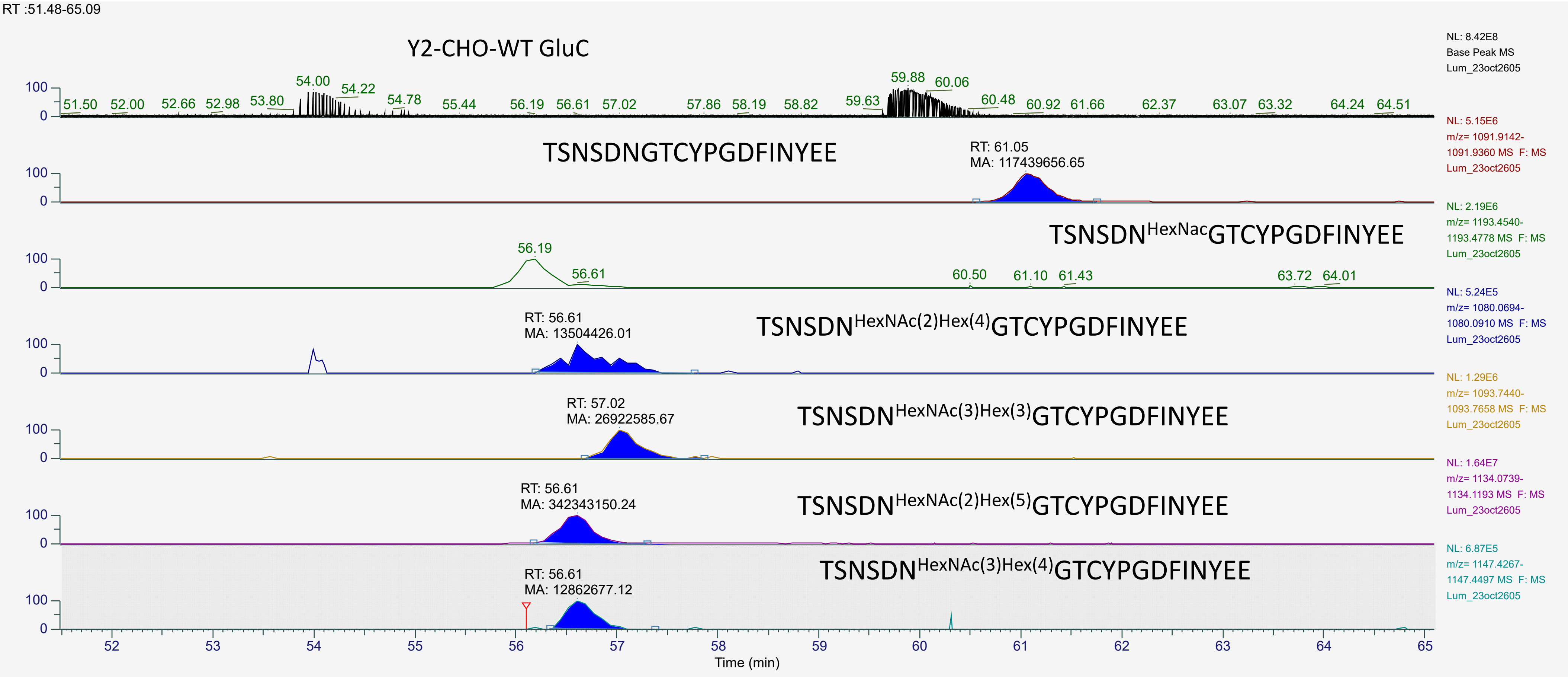

RT (Min)	Base Peak	Peak Area	Peak Height	Baseline Width	SignalToNoise
61.05395	1091.93	1.17E+08	5149503	1.193221	INF
56.60587	1080.08	13504426	508114.5	1.57813	INF
57.02326	1093.754	26922586	1292701	1.183598	INF
56.60587	1134.099	3.42E+08	15811393	1.126146	INF
56.60587	1147.441	12862677	655667.6	1.049144	INF

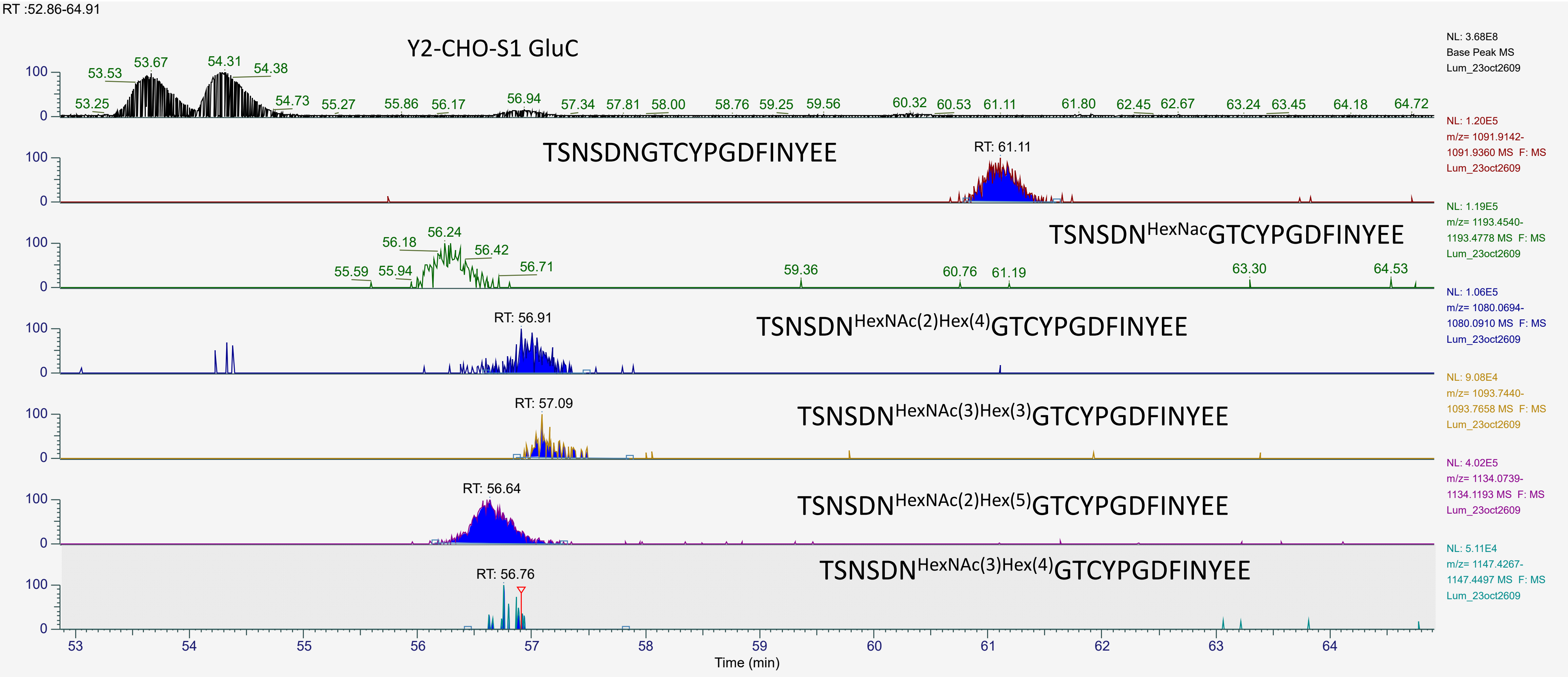

RT (Min)	Base Peak	Peak Area	Peak Height	Baseline Width	SignalToNoise
61.10602	1091.929	1852711	117632.5	0.792134	INF
56.90773	1080.081	1234605	105528.8	0.87731	INF
57.08939	1093.755	510410.7	87895.1	0.988039	INF
56.6373	1134.098	7688627	392655.9	1.133416	INF
56.75892	1147.44	115208.7	51142.2	1.380552	INF

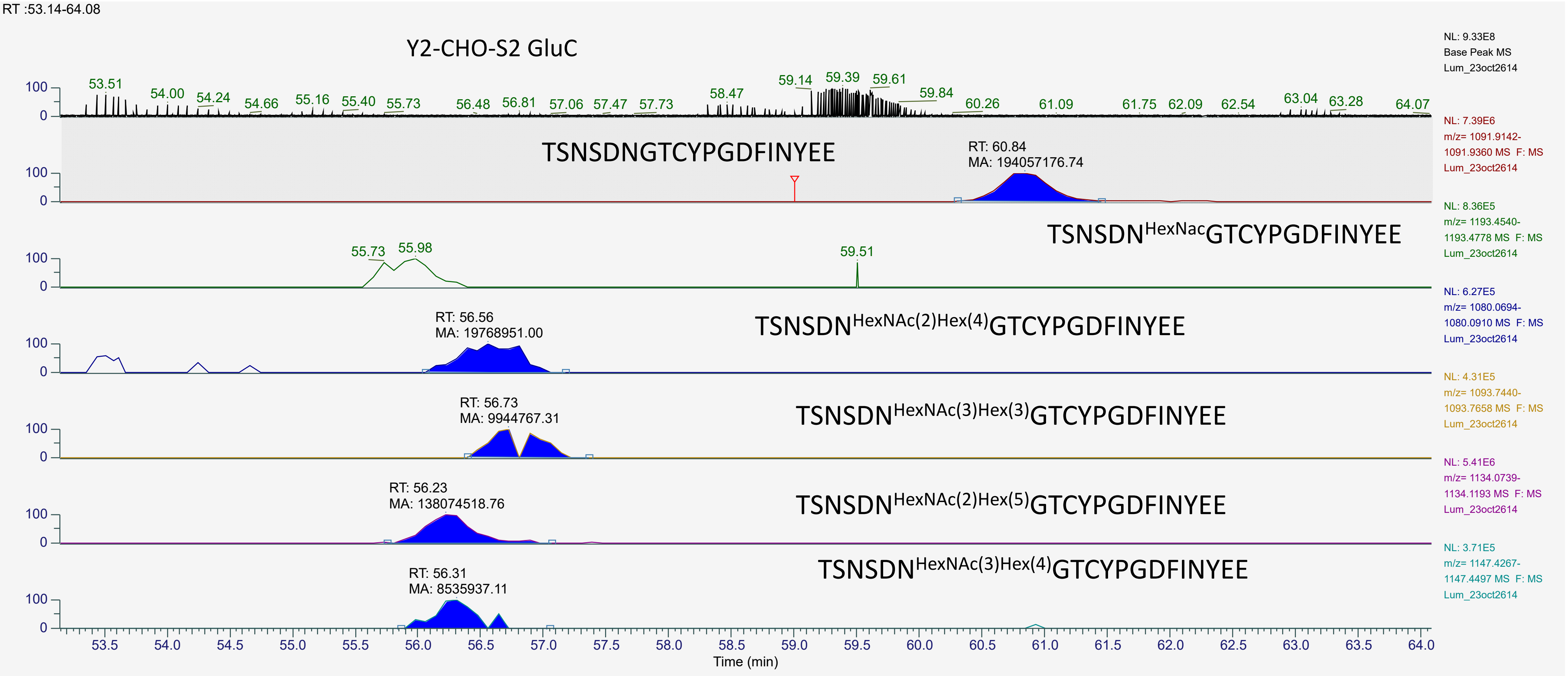

RT (Min)	Base Peak	Peak Area	Peak Height	Baseline Width	SignalToNoise
60.84118	1091.93	1.94E+08	7206618	1.145064	INF
56.55982	1080.079	19768951	616487.5	1.121904	INF
56.72622	1093.755	9944767	418926.6	0.967159	INF
56.22646	1134.097	1.38E+08	5409322	1.315276	INF
56.31022	1147.438	8535937	370818.4	1.191485	INF

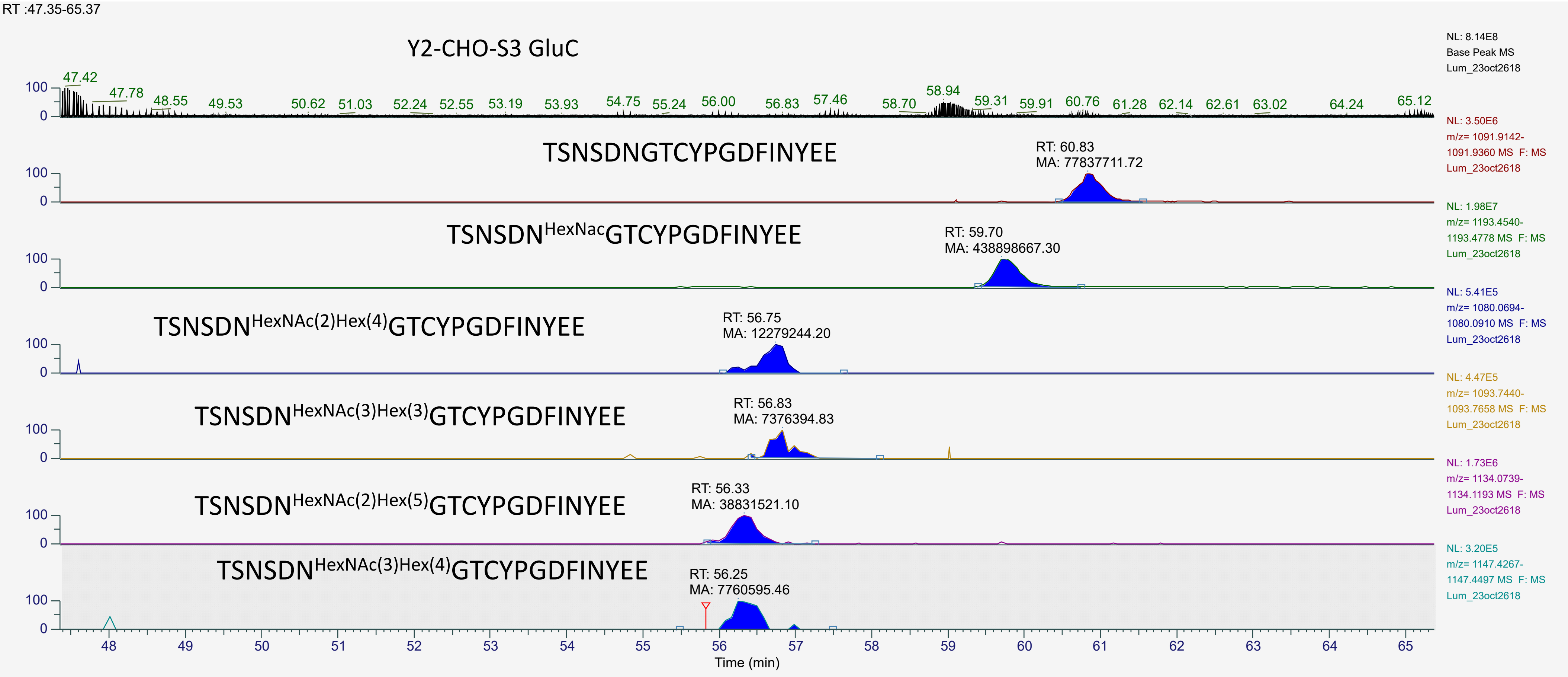

RT (Min)	Base Peak	Peak Area	Peak Height	Baseline Width	SignalToNoise
60.83379	1091.929	77837712	3501432	1.108727	INF
59.70403	1193.469	4.39E+08	19208629	1.350863	INF
56.74518	1080.08	12279244	541005.4	1.580255	INF
56.82855	1093.755	7376395	432887.9	1.682207	INF
56.32889	1134.097	38831521	1687780	1.414583	INF
56.2454	1147.437	7760595	319565.7	2.000806	INF

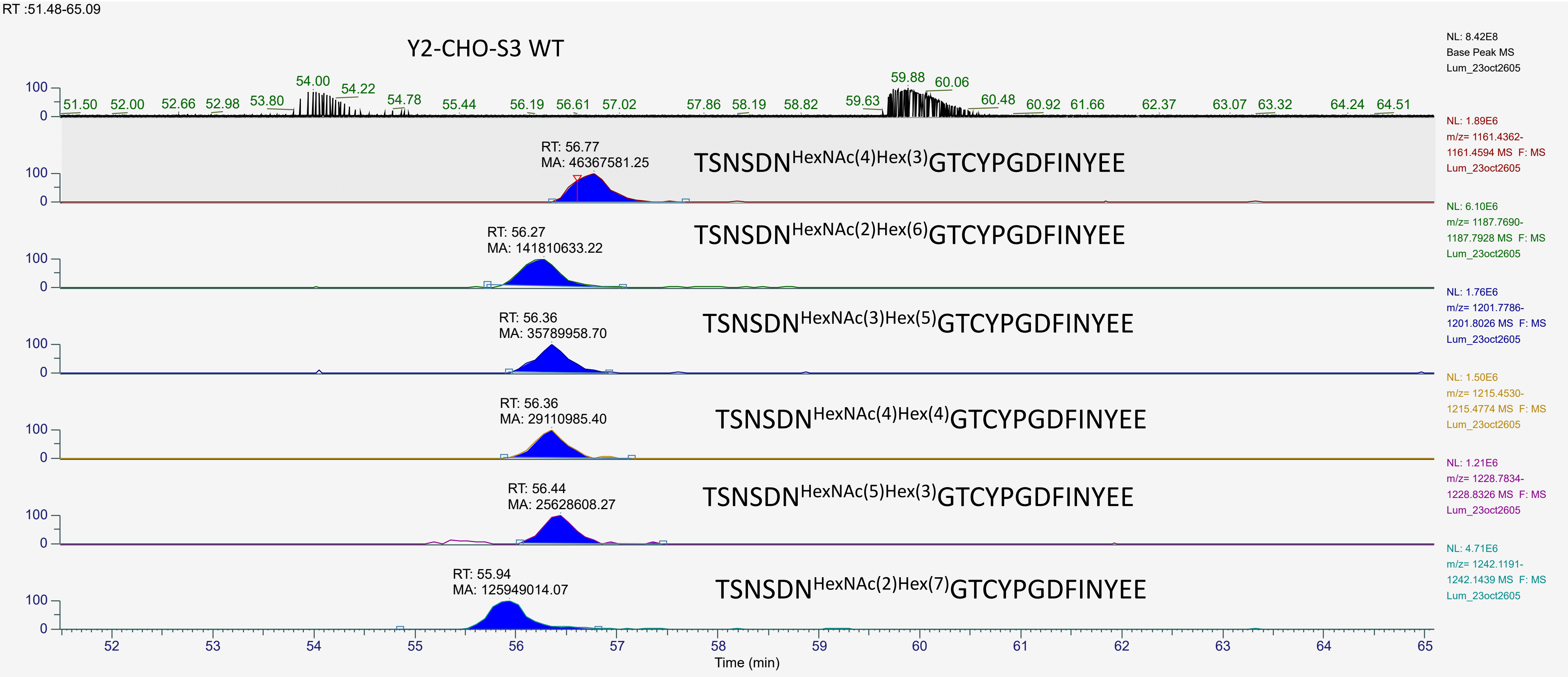

RT (Min)	Base Peak	Peak Area	Peak Height	Baseline Width	SignalToN oise
56.77257	1161.448	46367581	1891275	1.318649	INF
56.27322	1187.781	1.42E+08	5731949	1.3379	INF
56.35598	1201.791	35789959	1696196	0.991393	INF
56.35598	1215.465	29110985	1439644	1.260898	INF
56.43899	1228.807	25628608	1170795	1.424526	INF
55.93988	1242.132	1.26E+08	4709883	1.963536	INF

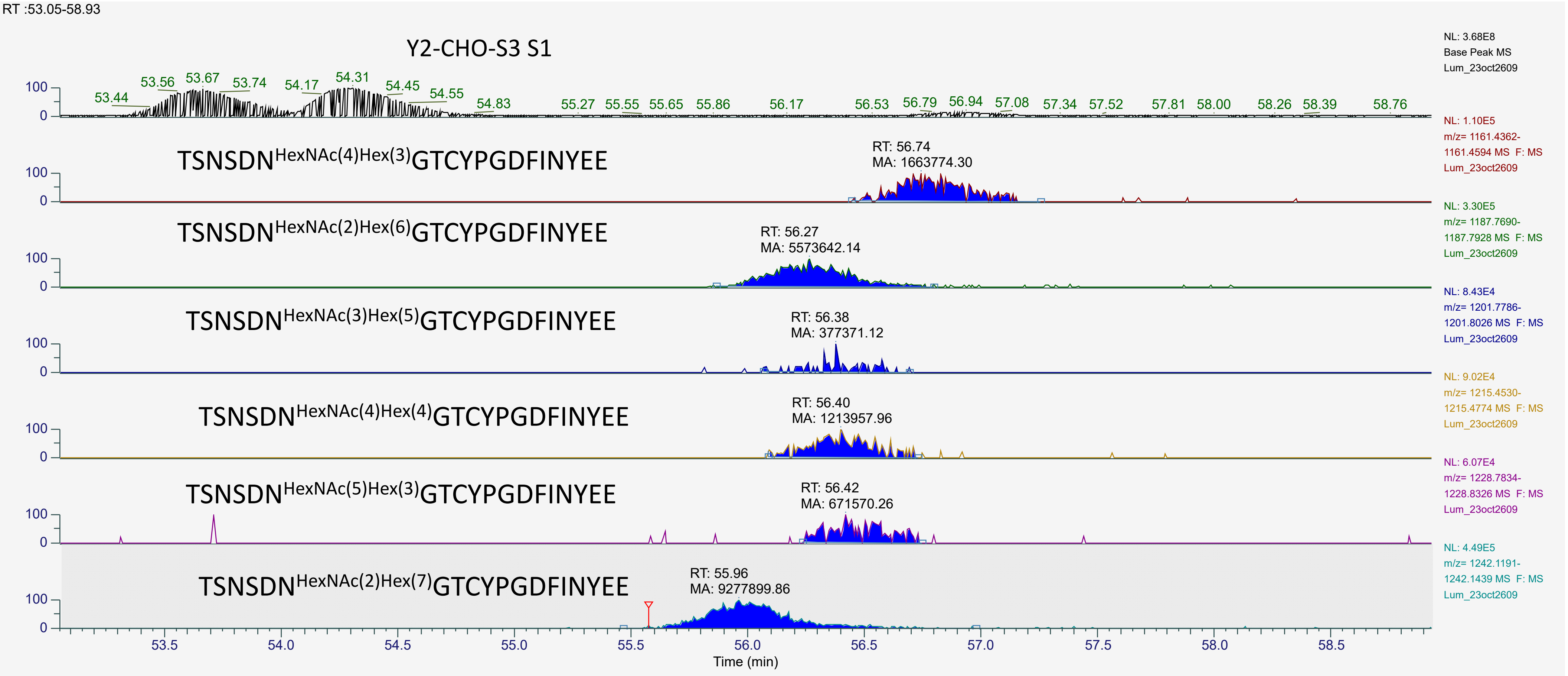

RT (Min)	Base Peak	Peak Area	Peak Height	Baseline Width	SignalToNoise
56.7431	1161.45	1663774	105544.3	0.811055	INF
56.26598	1187.781	5573642	322003.6	0.933357	INF
56.37884	1201.791	377371.1	82554.27	0.623654	INF
56.40157	1215.465	1213958	88315.57	0.64131	INF
56.41863	1228.804	671570.3	58209.86	0.509194	INF
55.96115	1242.132	9277900	448516.1	1.514698	INF

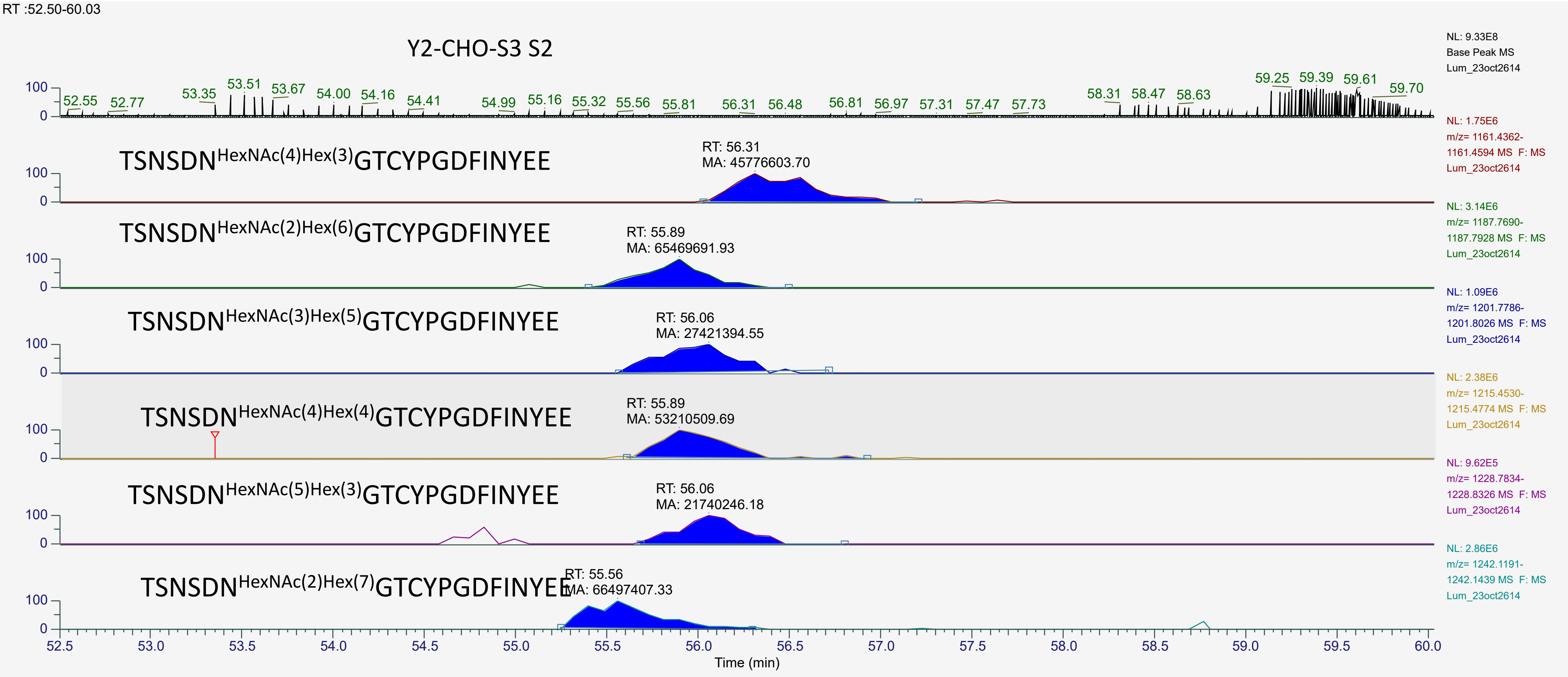

RT (Min)	Base Peak	Peak Area	Peak Height	Baseline Width	SignalToNoise
56.31022	1161.447	45776604	1709048	1.178275	INF
55.89419	1187.779	65469692	3104807	1.098302	INF
56.06028	1201.79	27421395	1027430	1.151618	INF
55.89419	1215.465	53210510	2262121	1.320679	INF
56.06028	1228.806	21740246	948820.6	1.118317	INF
55.55905	1242.131	66497407	2696449	1.049088	INF

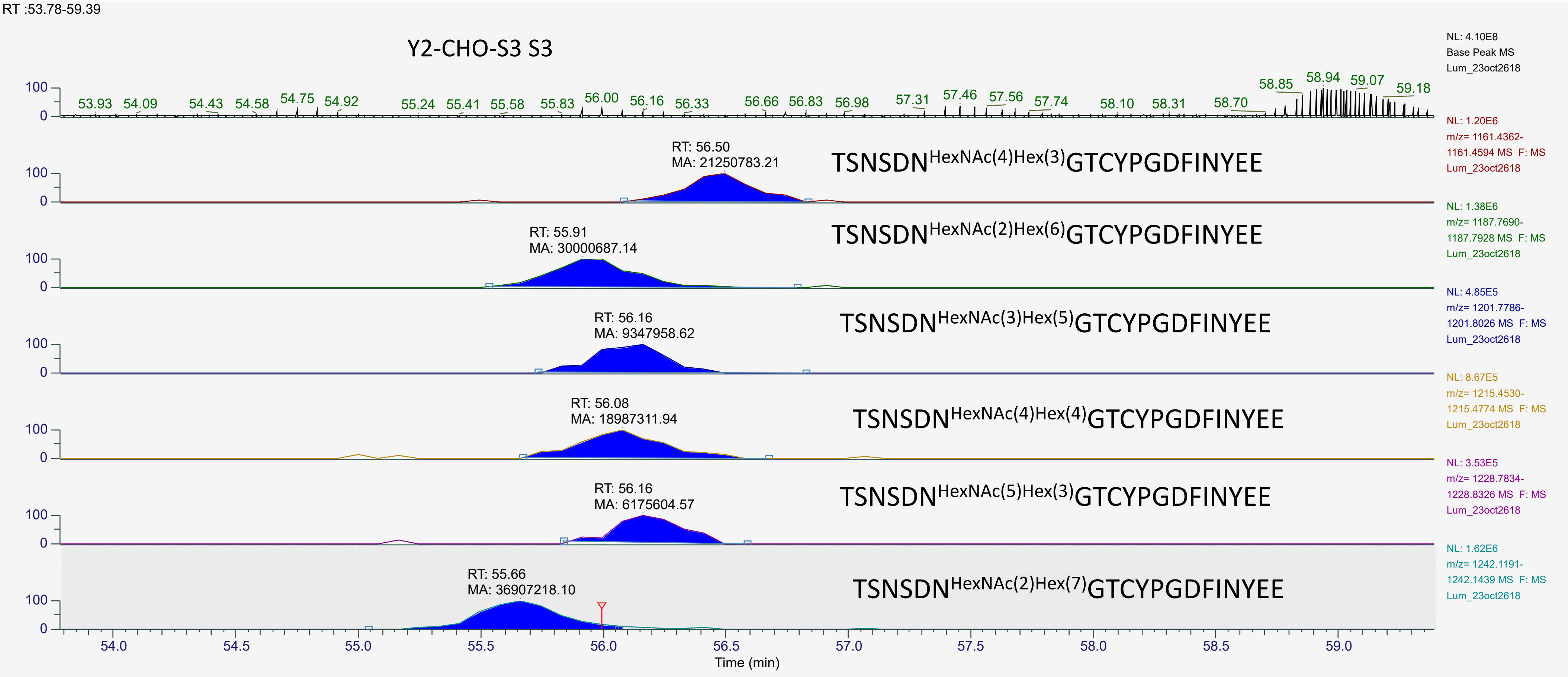

RT (Min)	Base Peak	Peak Area	Peak Height	Baseline Width	SignalToNoise
56.49562	1161.448	21250783	1167924	0.753574	INF
55.91242	1187.78	30000687	1339949	1.257279	INF
56.16213	1201.791	9347959	472536.5	1.095021	INF
56.07905	1215.467	18987312	845661.1	1.007737	INF
56.16213	1228.807	6175605	332311	0.745884	INF
55.66181	1242.131	36907218	1615497	1.019639	INF

Y2-CHO-WT GluC

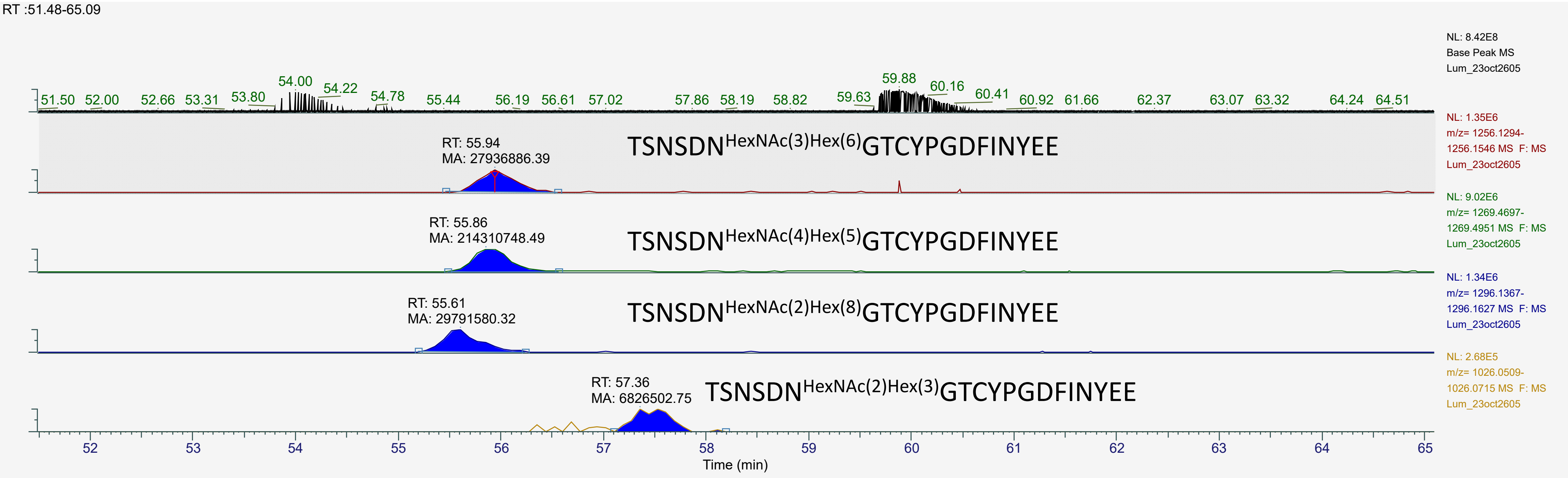

RT (Min)	Base Peak	Peak Area	Peak Height	Baseline Width	SignalToNoise
55.93988	1256.142	27936886	1296545	1.087645	INF
55.85683	1269.482	2.14E+08	8879265	1.07802	INF
55.60605	1296.15	29791580	1296937	1.039519	INF
57.35825	1026.061	6826503	267802	1.087645	INF

Y2-CHO-S1 GluC

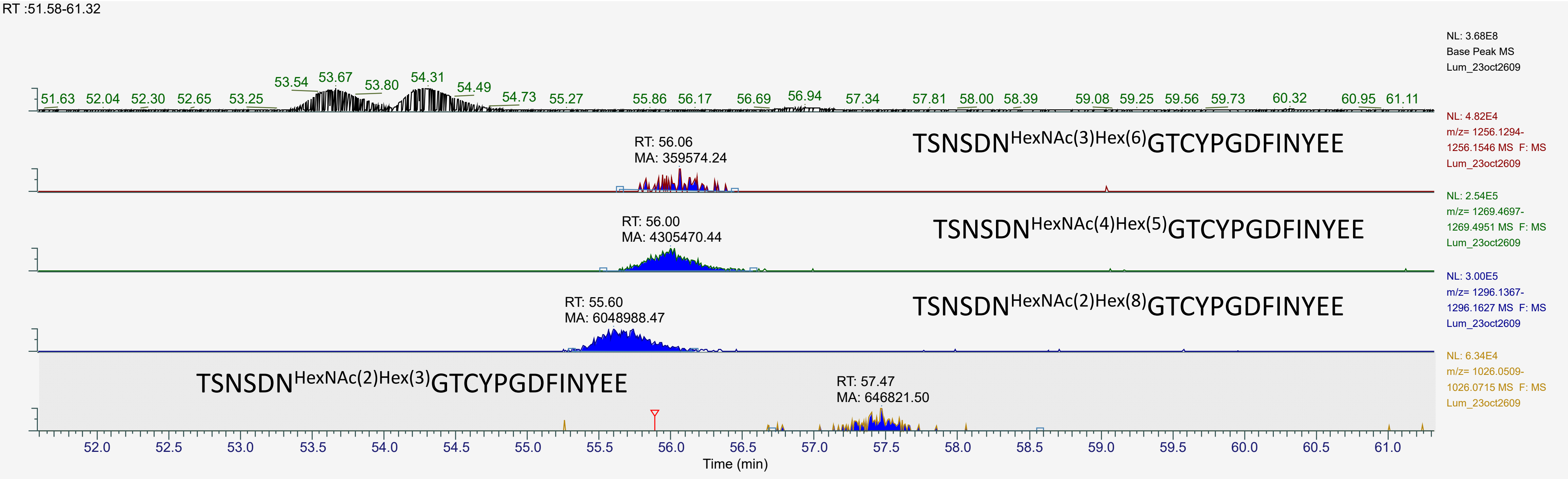

RT (Min)	Base Peak	Peak Area	Peak Height	Baseline Width	SignalToNoise
56.0601	1256.142	359574.2	46134.23	0.799482	INF
55.99591	1269.484	4305470	254489.8	1.047597	INF
55.6031	1296.15	6048988	295315.3	0.854144	INF
57.47154	1026.061	646821.5	63391.26	1.866719	INF

Y2-CHO-S2 GluC

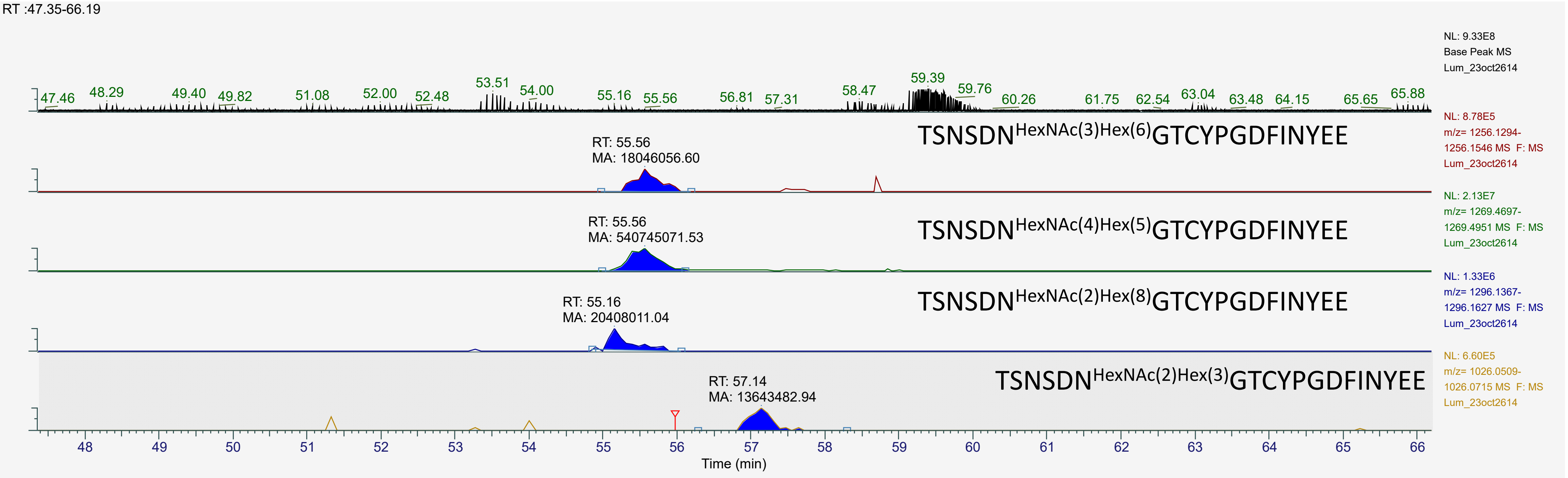

RT (Min)	Base Peak	Peak Area	Peak Height	Baseline Width	SignalToNoise
55.55905	1256.141	18046057	862208.6	1.212931	INF
55.55905	1269.483	5.41E+08	21060315	1.119628	INF
55.16022	1296.15	20408011	1211164	1.199151	INF
57.14113	1026.062	13643483	659583.8	2.011909	INF

Y2-CHO-S3 GluC

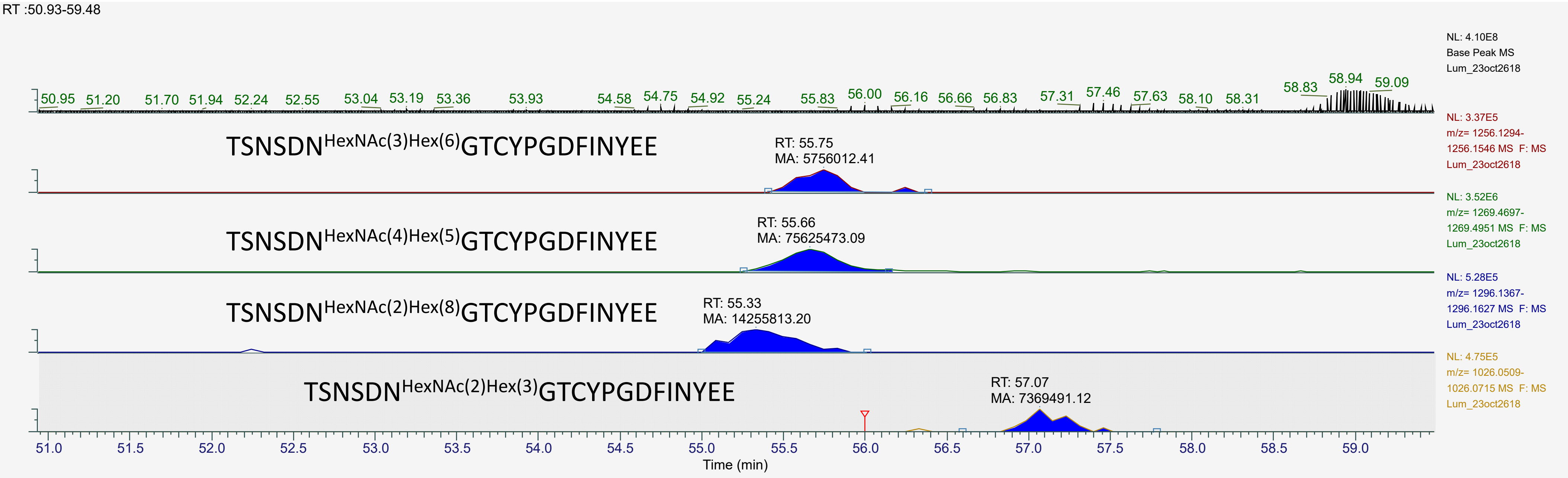

RT (Min)	Base Peak	Peak Area	Peak Height	Baseline Width	SignalToNoise
55.74521	1256.14	5756012	325106.7	0.979581	INF
55.66181	1269.483	75625473	3428158	0.888879	INF
55.32832	1296.15	14255813	519186	1.015842	INF
57.06648	1026.062	7369491	474861.2	1.185149	INF

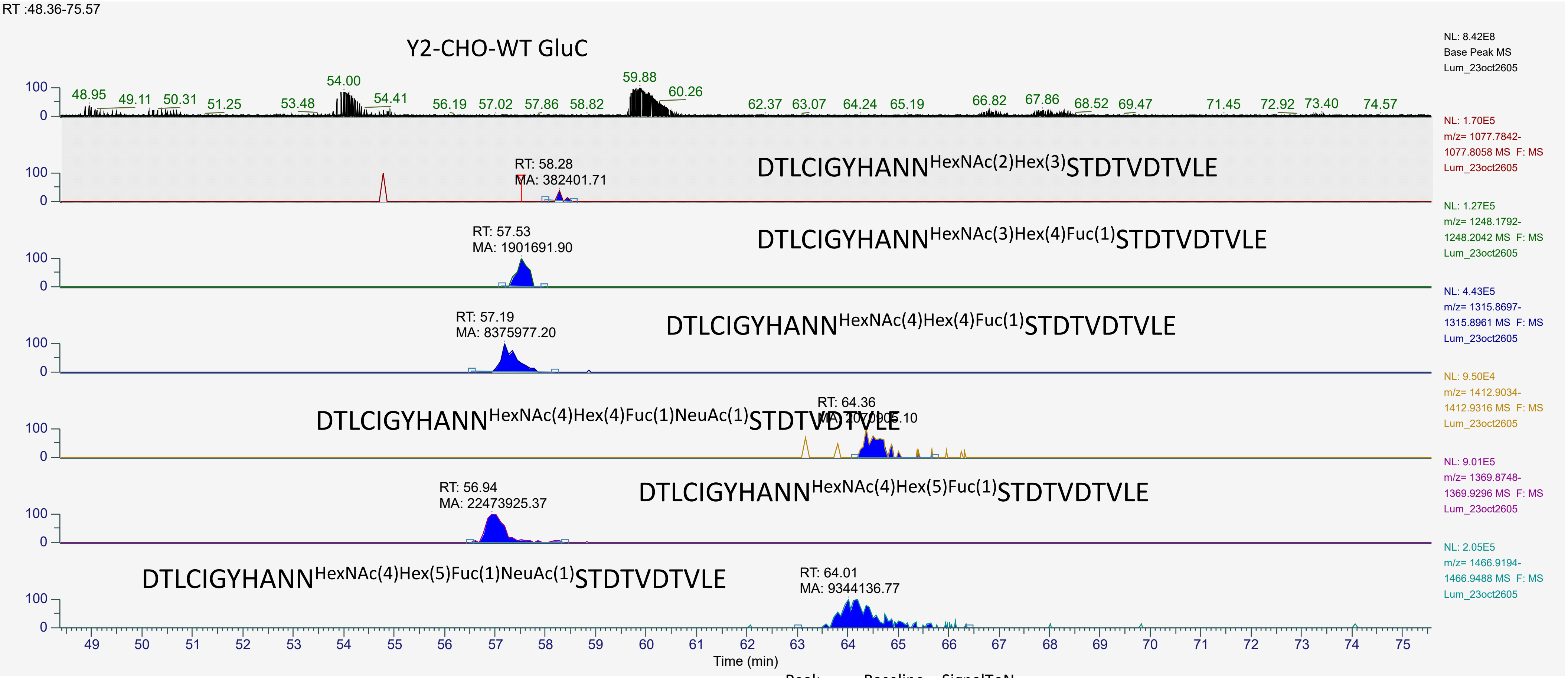

RT (Min)	Base Peak	Peak Area	Peak Height	Baseline Width	SignalToNoise
58.27577	1077.794	382401.7	57192.86	0.577298	INF
57.52578	1248.192	1901692	122923.2	0.827461	INF
57.19094	1315.885	8375977	432273.2	1.654922	INF
64.36195	1412.917	2070905	95038.45	1.577949	INF
56.93977	1369.904	22473925	886972.8	1.886058	INF
64.00639	1466.933	9344137	205281	3.406452	INF

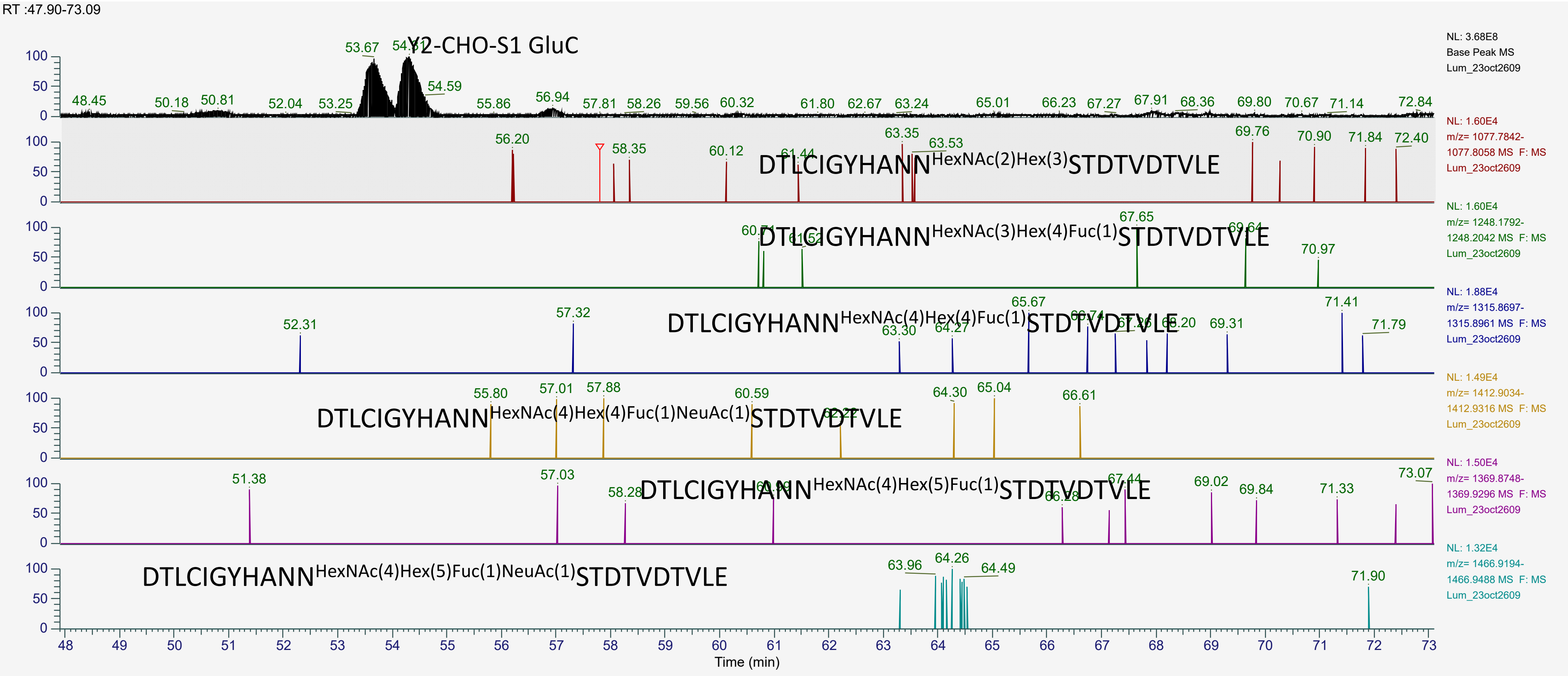

RT :47.90-73.09
Y2-CHO-S1 GluC
NL: 3.68E8
Base Peak MS
Lum_23oct2609
DTLCIGYHANN HexNAc(2)Hex(3) STDTVDTVLE
NL: 1.60E4
m/z= 1077.7842-1077.8058 MS F: MS
Lum_23oct2609
DTLCIGYHANN HexNAc(3)Hex(4)Fuc(1) STDTVDTVLE
NL: 1.60E4
m/z= 1248.1792-1248.2042 MS F: MS
Lum_23oct2609
DTLCIGYHANN HexNAc(4)Hex(4)Fuc(1) STDTVDTVLE
NL: 1.88E4
m/z= 1315.8697-1315.8961 MS F: MS
Lum_23oct2609
DTLCIGYHANN HexNAc(4)Hex(4)Fuc(1)NeuAc(1) STDTVDTVLE
NL: 1.49E4
m/z= 1412.9034-1412.9316 MS F: MS
Lum_23oct2609
DTLCIGYHANN HexNAc(4)Hex(5)Fuc(1) STDTVDTVLE
NL: 1.50E4
m/z= 1369.8748-1369.9296 MS F: MS
Lum_23oct2609
DTLCIGYHANN HexNAc(4)Hex(5)Fuc(1)NeuAc(1) STDTVDTVLE
NL: 1.32E4
m/z= 1466.9194-1466.9488 MS F: MS
Lum_23oct2609
Time (min)

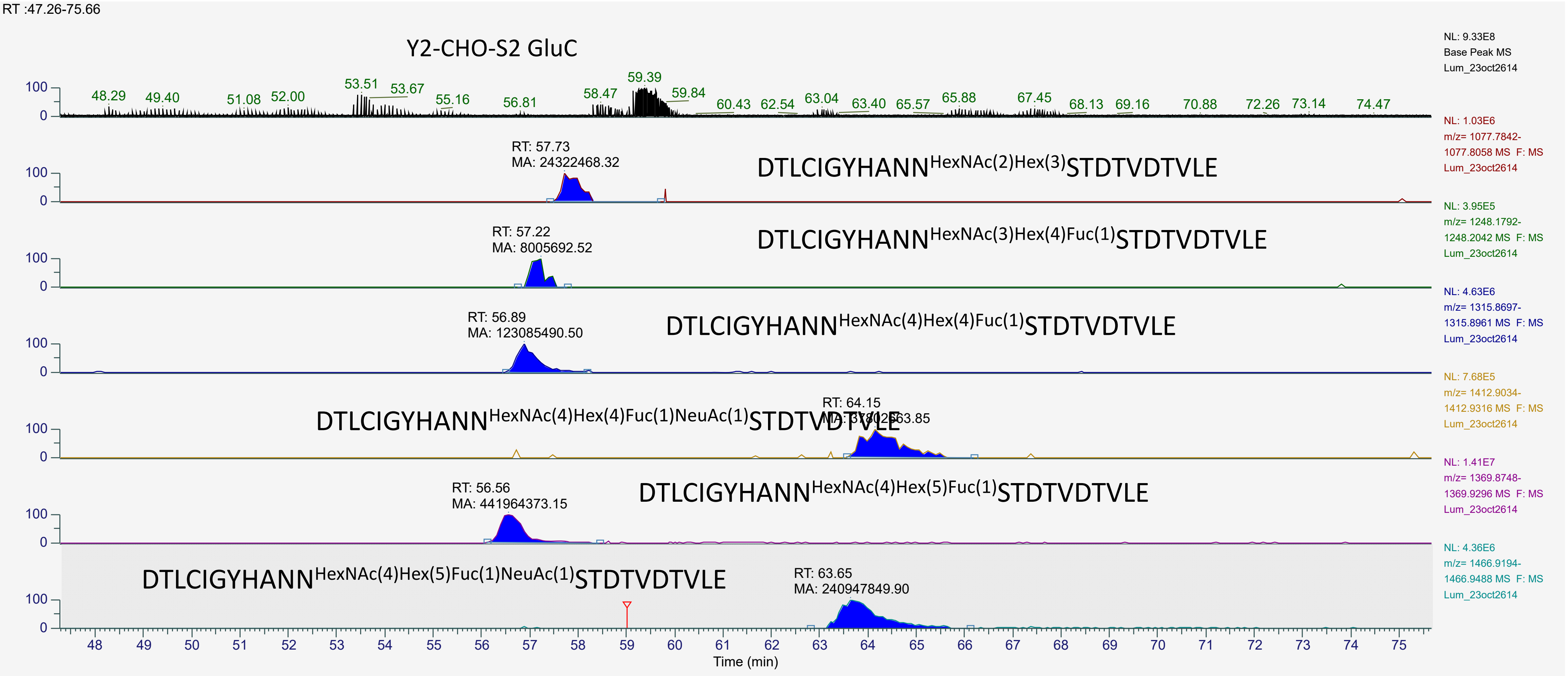

RT (Min)	Base Peak	Peak Area	Peak Height	Baseline Width	SignalToNoise
57.72503	1077.795	24322468	999640.8	2.289675	INF
57.22452	1248.19	8005693	390999.7	1.044413	INF
56.89144	1315.883	1.23E+08	4552210	1.687129	INF
64.15145	1412.916	37802664	743542.7	2.631117	INF
56.55982	1369.902	4.42E+08	13643484	2.329844	INF
63.64991	1466.934	2.41E+08	4362410	3.314003	INF

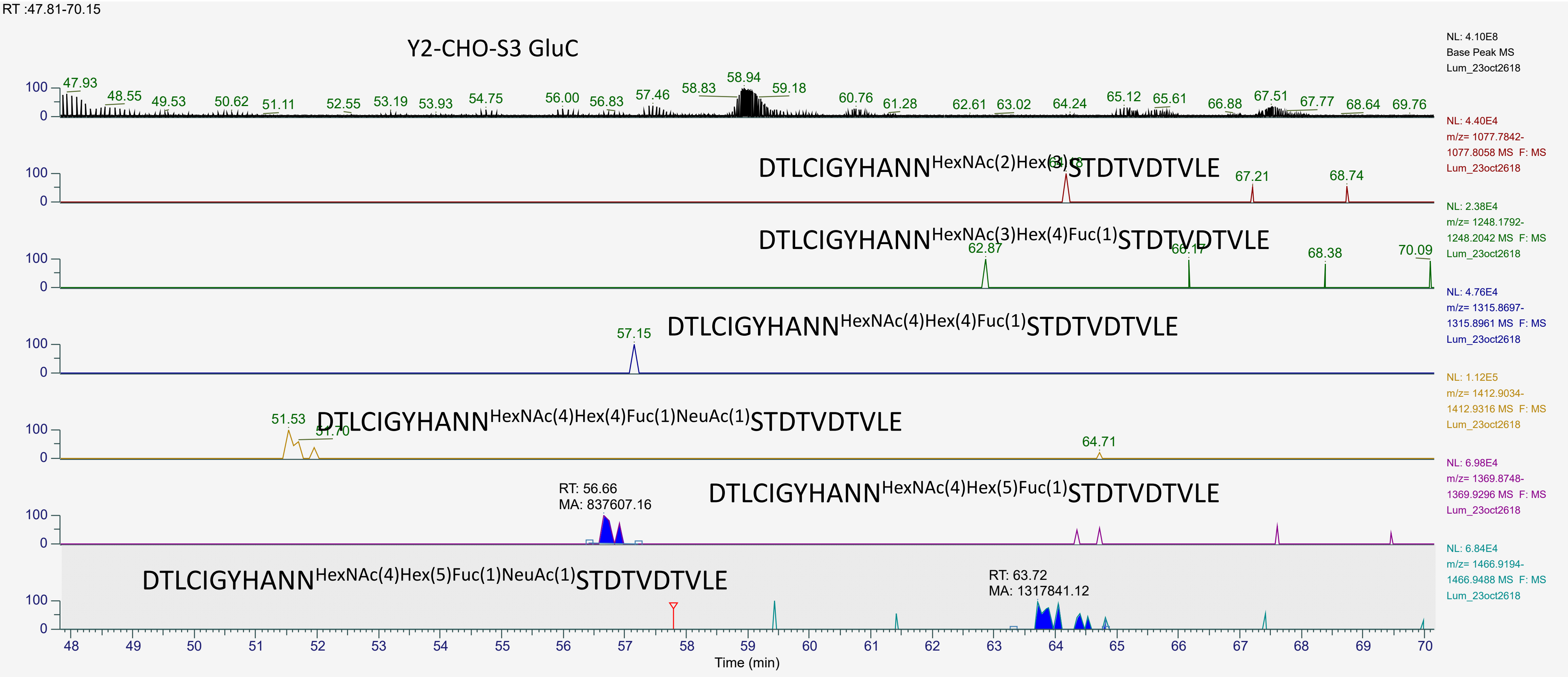

RT (Min)	Base Peak	Peak Area	Peak Height	Baseline Width	SignalToNoise
56.66221	1369.899	837607.2	67527.57	0.805784	INF
63.71845	1466.935	1317841	63996.25	1.500971	INF

Y2-CHO-S2 GluC

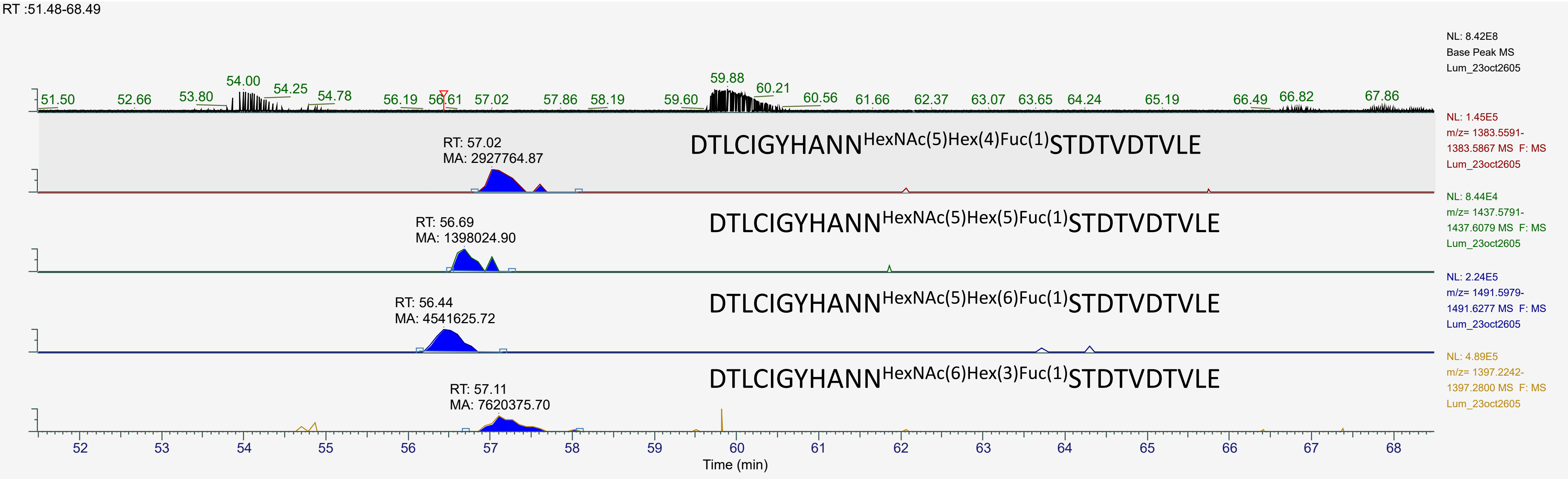

RT (Min)	Base Peak	Peak Area	Peak Height	Baseline Width	SignalToNoise
57.02326	1383.577	2927765	142267.6	1.263119	INF
56.68894	1437.597	1398025	81368.43	0.745842	INF
56.43899	1491.614	4541626	216865.7	1.010388	INF
57.1071	1397.254	7620376	338613.8	1.395298	INF

Y2-CHO-S2 GluC

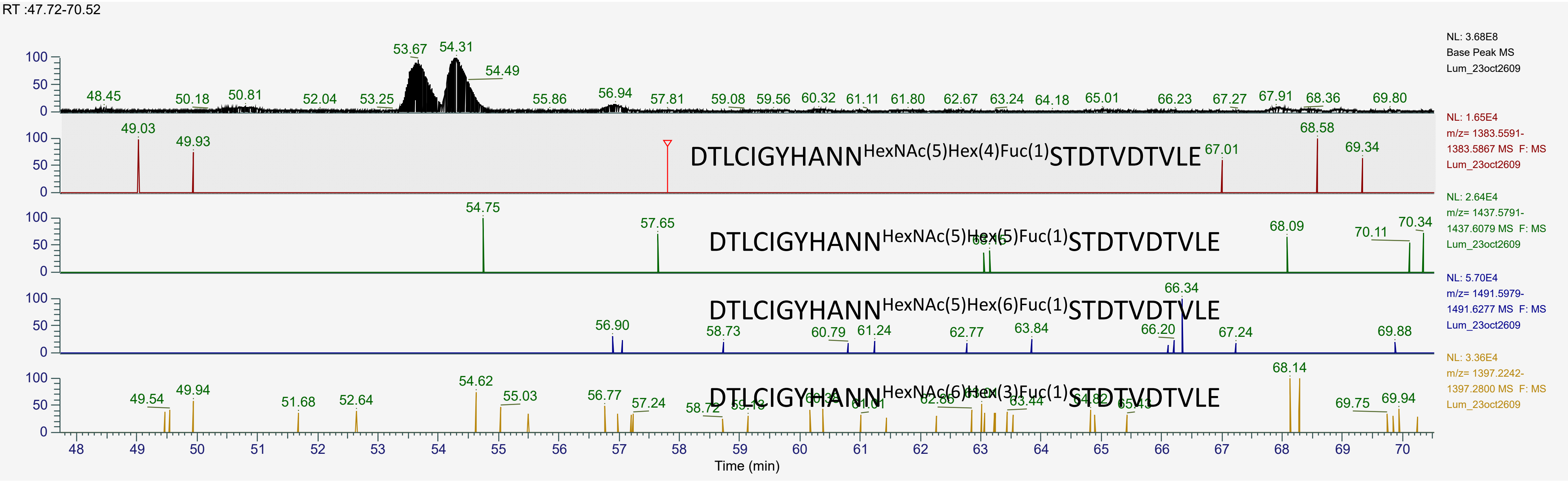

Y2-CHO-S2 GluC

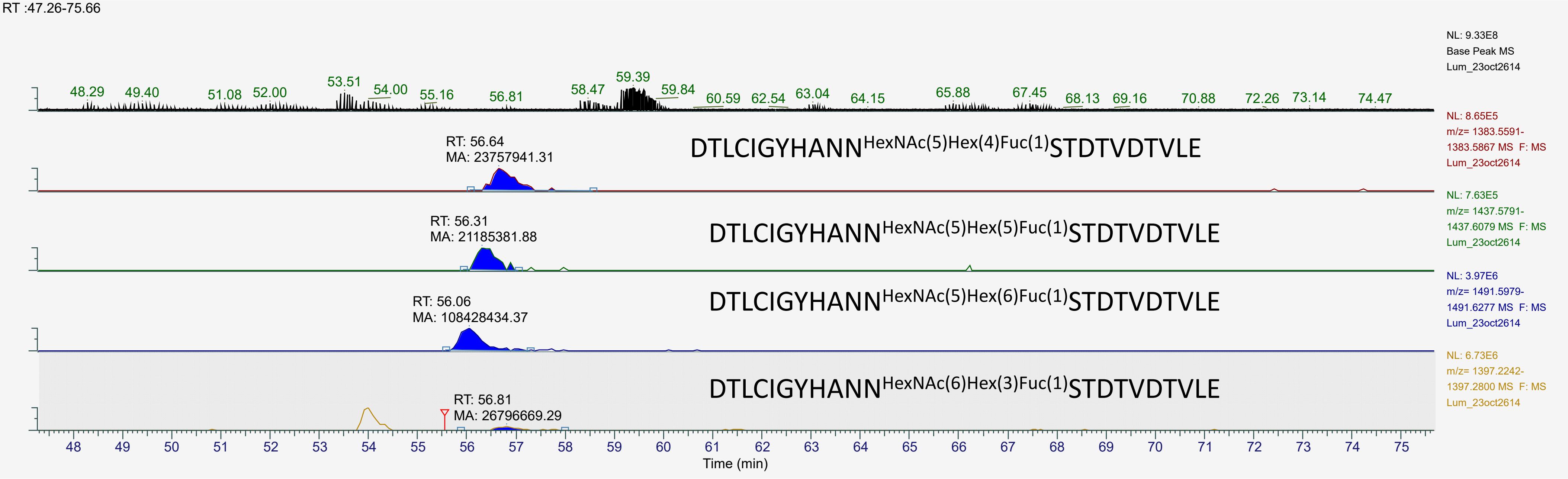

RT (Min)	Base Peak	Peak Area	Peak Height	Baseline Width	SignalToNoise
56.64281	1383.576	23757941	820223.8	2.490523	INF
56.31022	1437.594	21185382	740204.5	1.104668	INF
56.06028	1491.613	1.08E+08	3766346	1.707214	INF
56.80873	1397.252	26796669	893067.1	2.108911	INF

Y2-CHO-S3 GluC

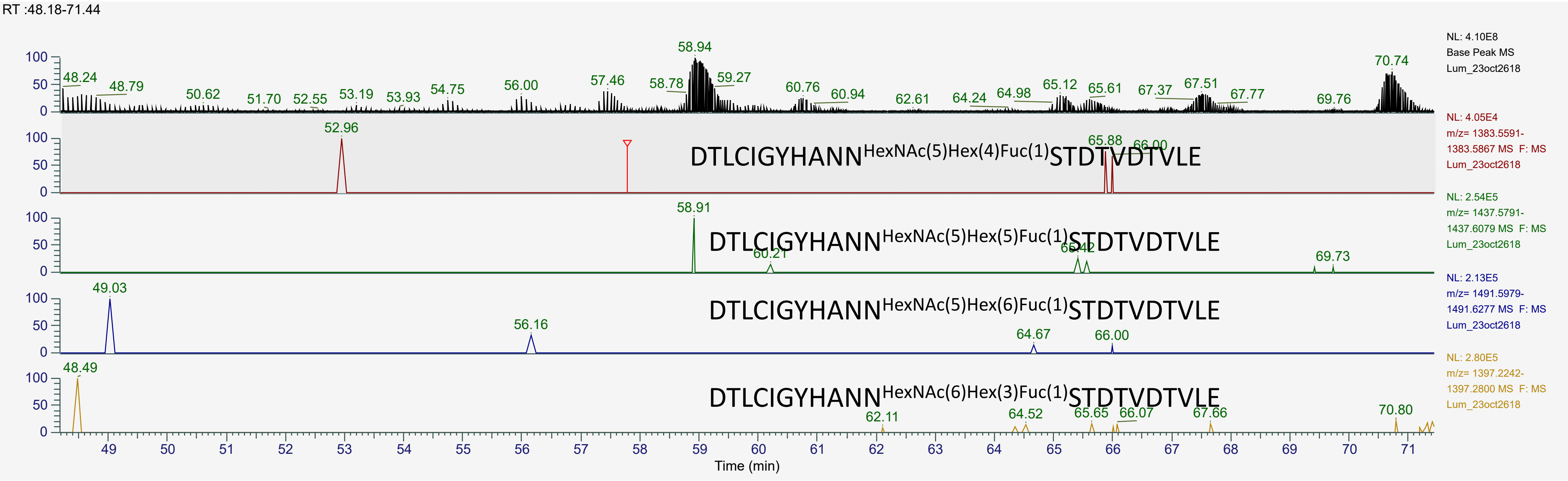

Y2-CHO-WT Trypsin

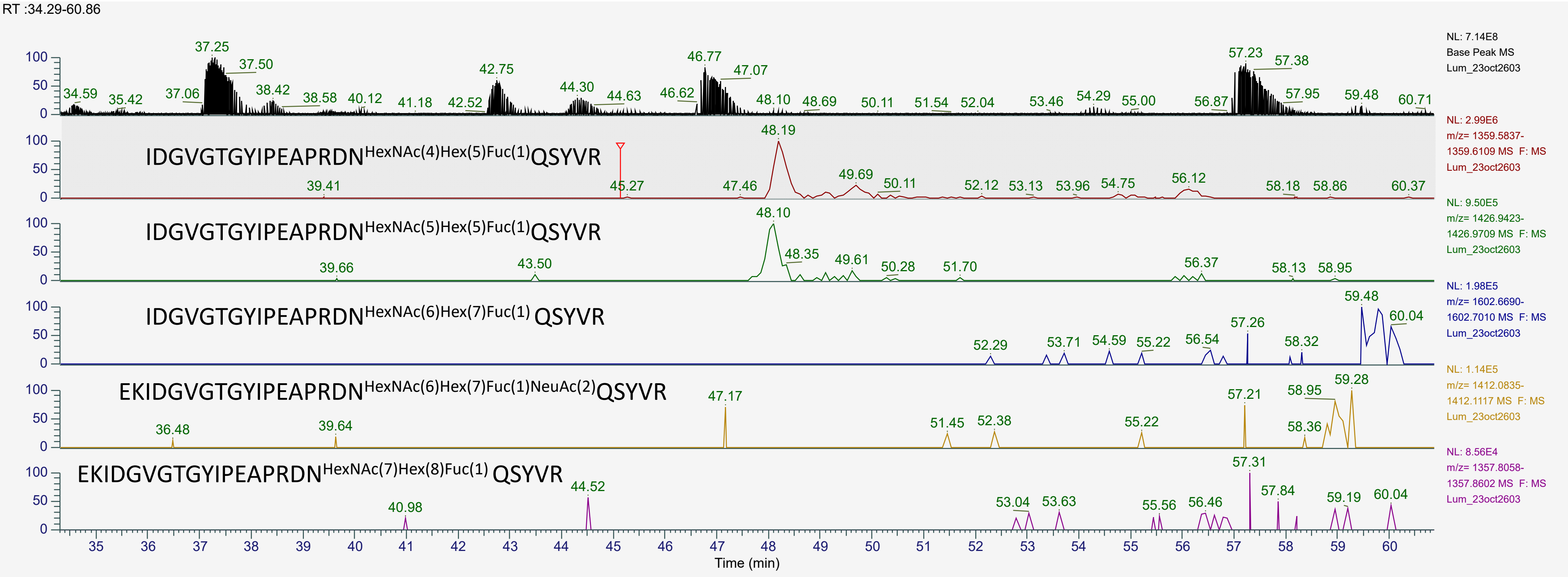

Y2-CHO-WT Trypsin

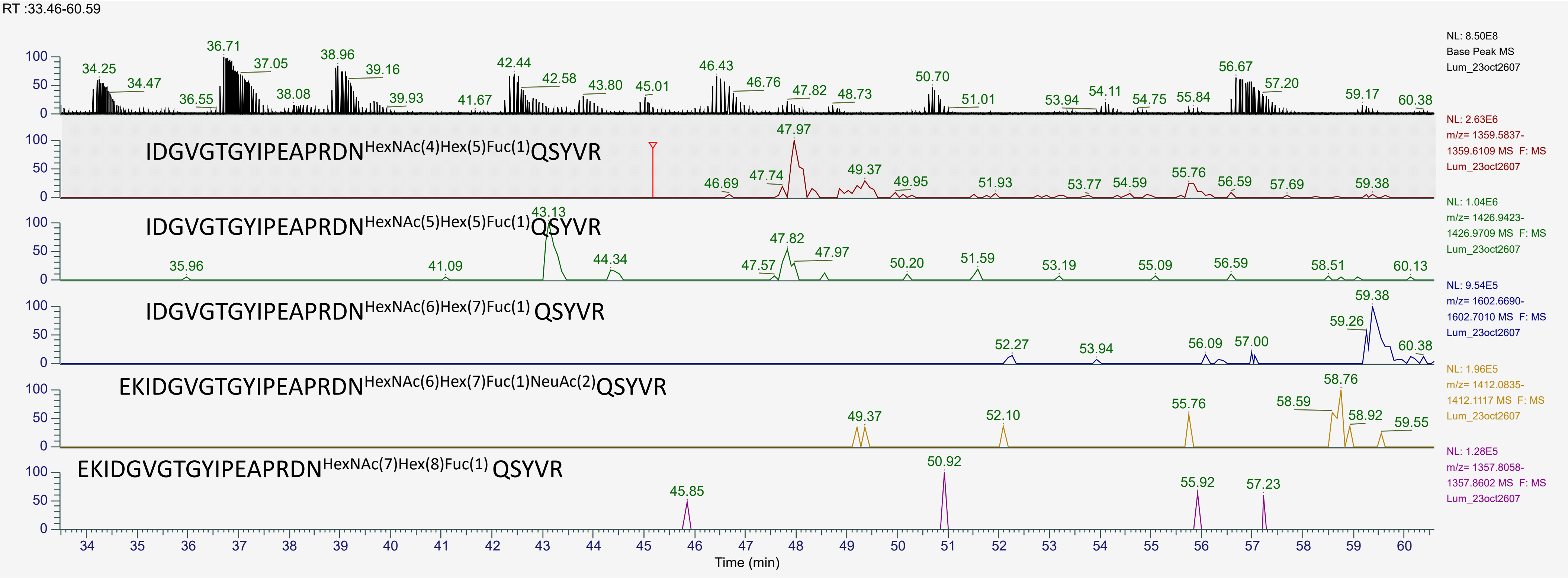

Y2-CHO-S2 Trypsin

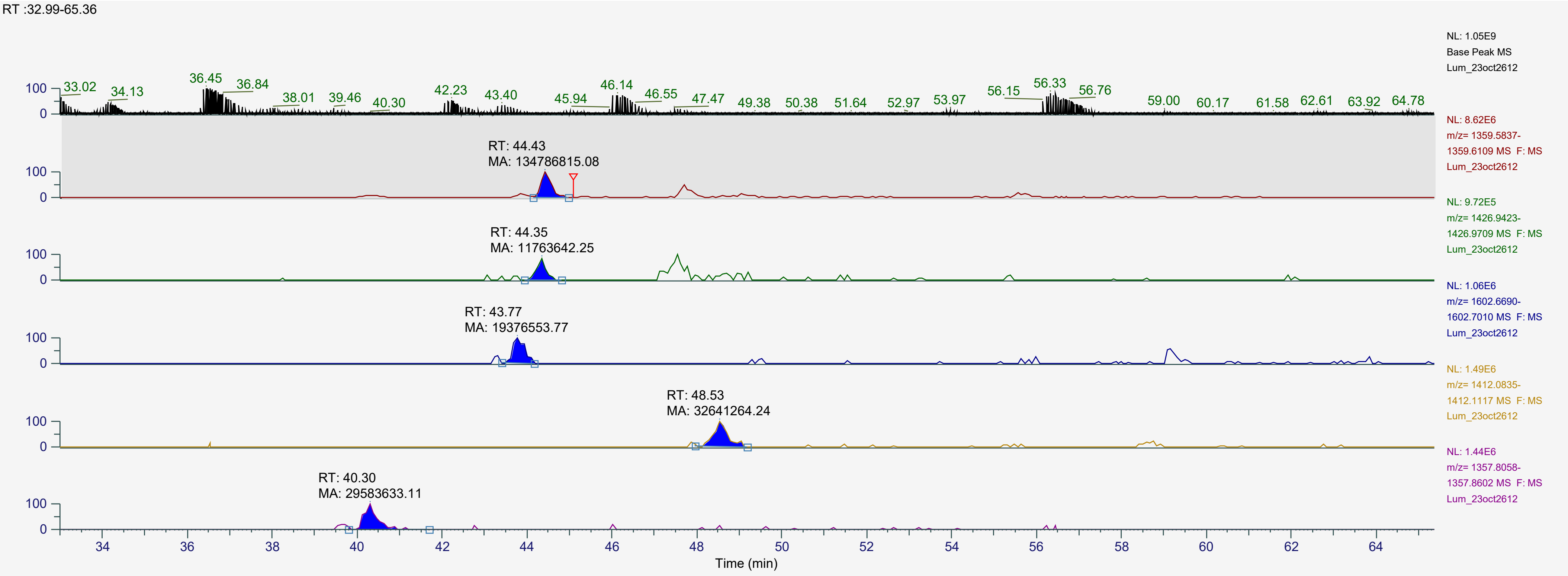

RT (Min)	Base Peak	Peak Area	Peak Height	Baseline Width	SignalToNoise
44.43452	1359.597	1.35E+08	8495077	0.847023	INF
44.35067	1426.957	11763642	798368.5	0.892808	INF
43.76663	1602.686	19376554	1033543	0.755453	INF
48.53431	1412.096	32641264	1453538	1.236195	INF
40.29662	1357.832	29583633	1438141	1.900078	INF

Y2-CHO-S3 Trypsin

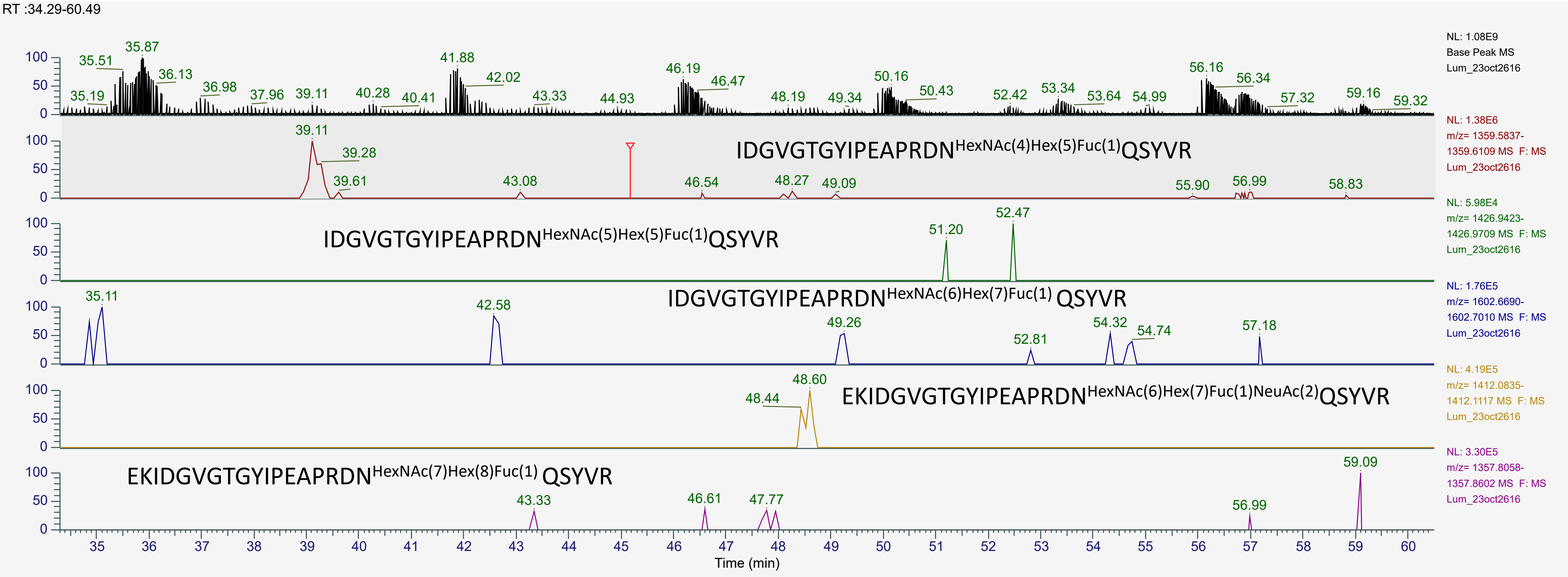

Y2-CHO-WT Trypsin

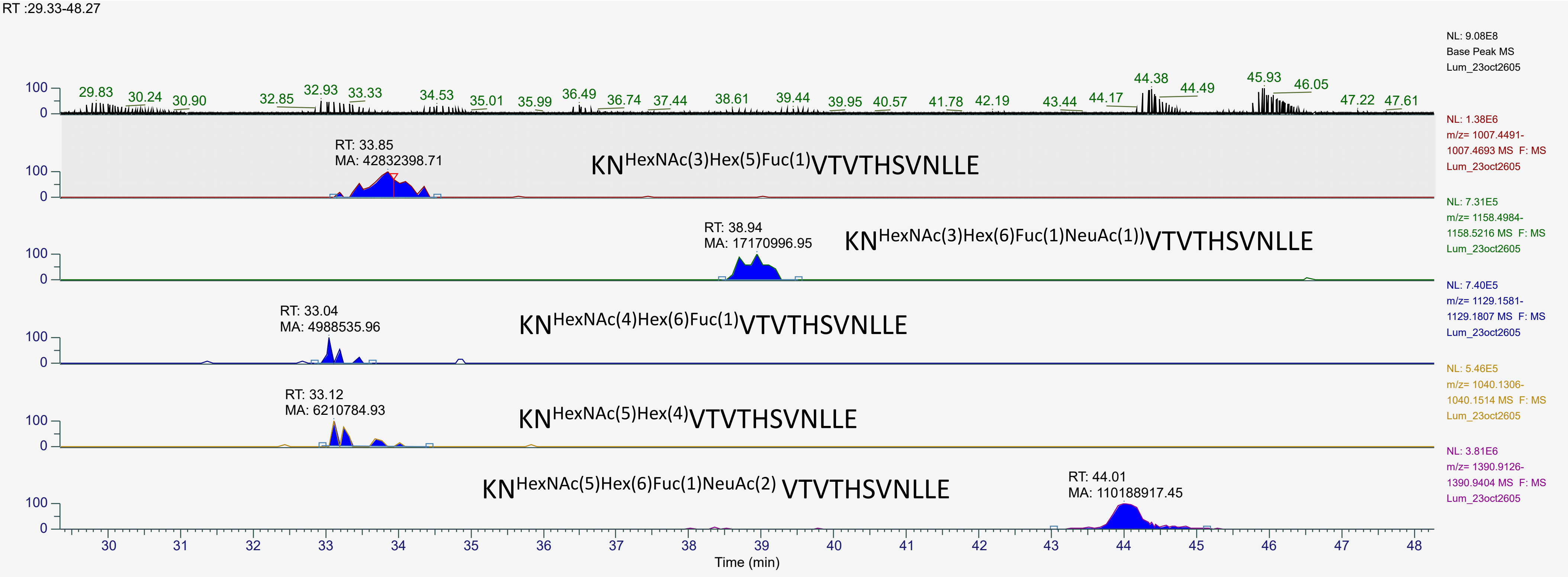

RT (Min)	Base Peak	Peak Area	Peak Height	Baseline Width	SignalToNoise
33.85176	1007.459	42832399	1362520	1.433225	INF
38.94285	1158.509	17170997	731255.4	1.058175	INF
33.03863	1129.17	4988536	727225.7	0.803678	INF
33.11586	1040.146	6210785	524426.4	1.473321	INF
44.00768	1390.927	1.1E+08	3805037	2.10283	INF

Y2-CHO-S2 Trypsin

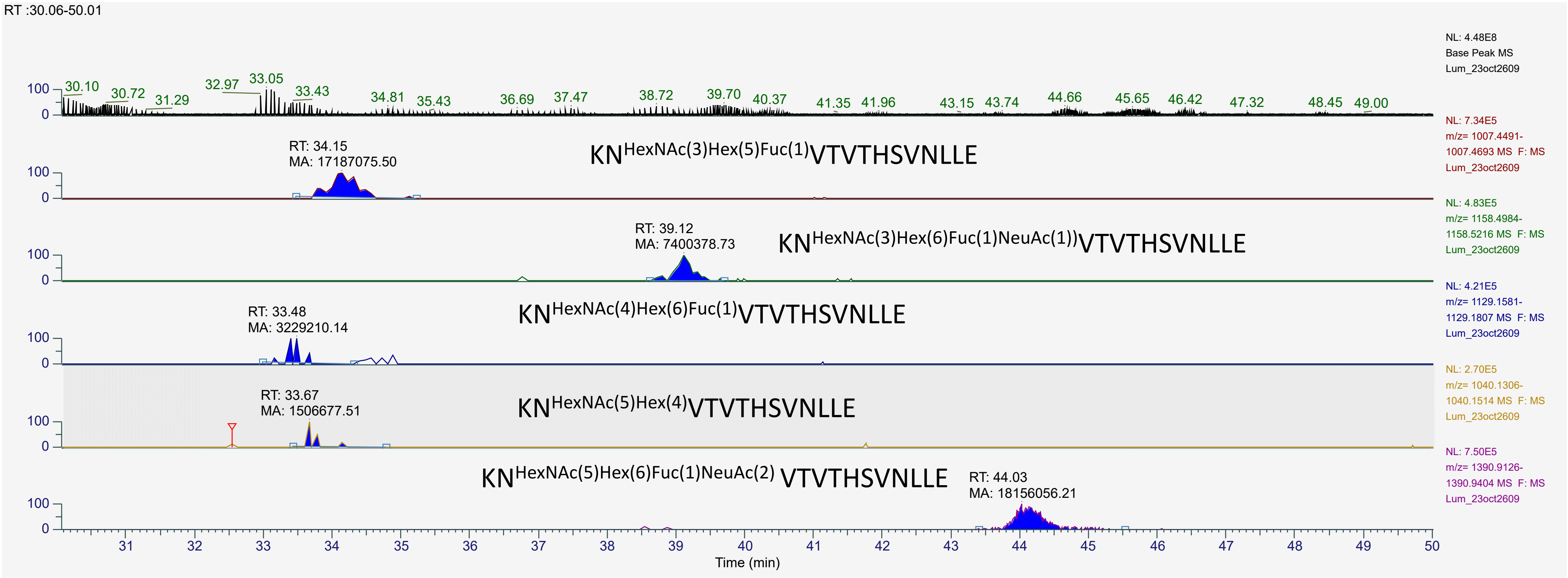

RT (Min)	Base Peak	Peak Area	Peak Height	Baseline Width	SignalToNoise
34.14937	1007.46	17187075	694154.6	1.749549	INF
39.11678	1158.51	7400379	476972.1	1.072277	INF
33.48325	1129.17	3229210	391839	1.312129	INF
33.66982	1040.145	1506678	260537.2	1.354455	INF
44.02887	1390.928	18156056	749775.3	2.11639	INF

Y2-CHO-S2 Trypsin

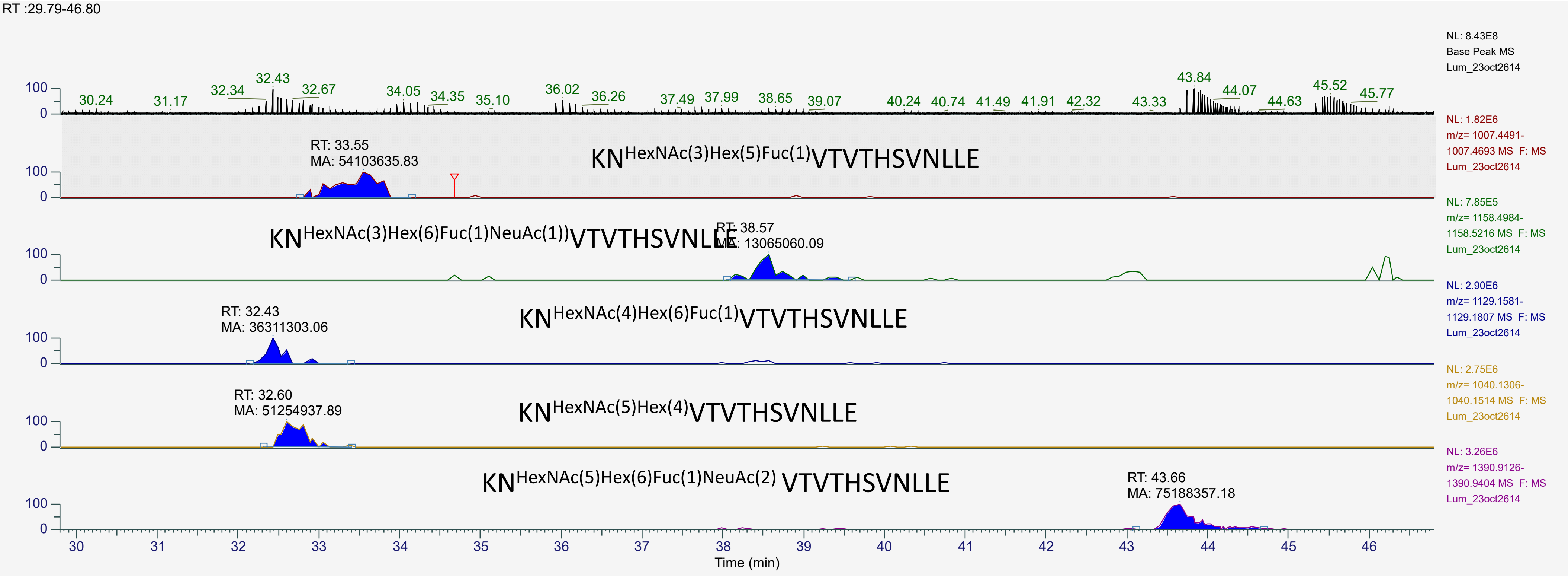

RT (Min)	Base Peak	Peak Area	Peak Height	Baseline Width	SignalToNoise
33.54975	1007.459	54103636	1803800	1.383416	INF
38.57147	1158.508	13065060	761983.9	1.551832	INF
32.42757	1129.17	36311303	2846083	1.251089	INF
32.60499	1040.142	51254938	2657964	1.082673	INF
43.65826	1390.926	75188357	3256389	1.587921	INF

Y2-CHO-S3 Trypsin

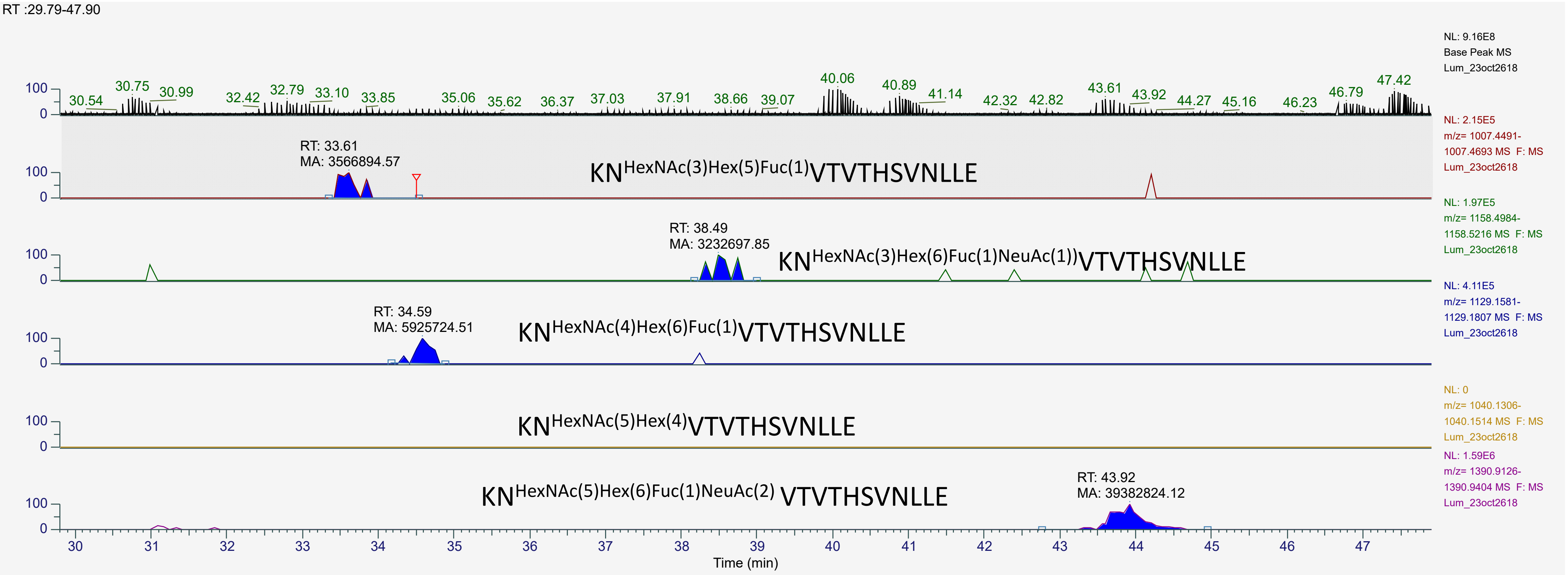

RT (Min)	Base Peak	Peak Area	Peak Height	Baseline Width	SignalToNoise
33.61317	1007.46	3566895	211801.9	1.19111	INF
38.49487	1158.51	3232698	194331.3	0.832496	INF
34.58513	1129.161	5925725	403068.2	0.717293	INF
43.92003	1390.926	39382824	1593980	2.177497	INF

Y2-CHO-WT Trypsin

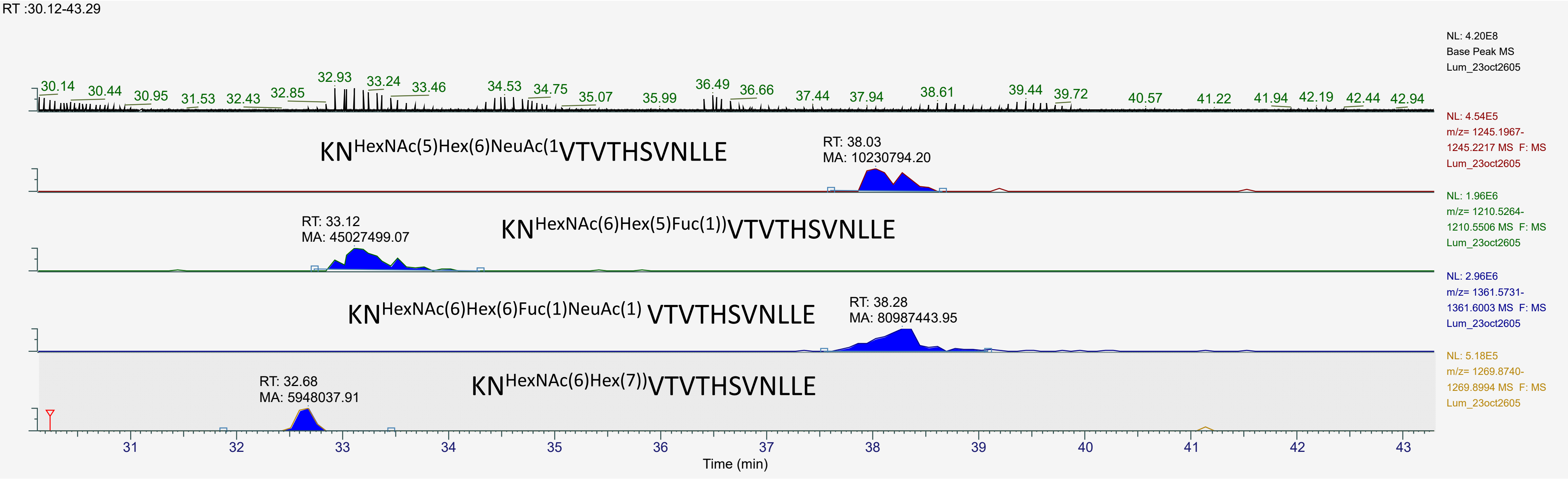

RT (Min)	Base Peak	Peak Area	Peak Height	Baseline Width	SignalToNoise
38.0277	1245.206	10230794	440642.8	1.057659	INF
33.11586	1210.538	45027499	1822267	1.556096	INF
38.27637	1361.587	80987444	2927291	1.544126	INF
32.68148	1269.886	5948038	518366.3	1.580601	INF

Y2-CHO-S1 Trypsin

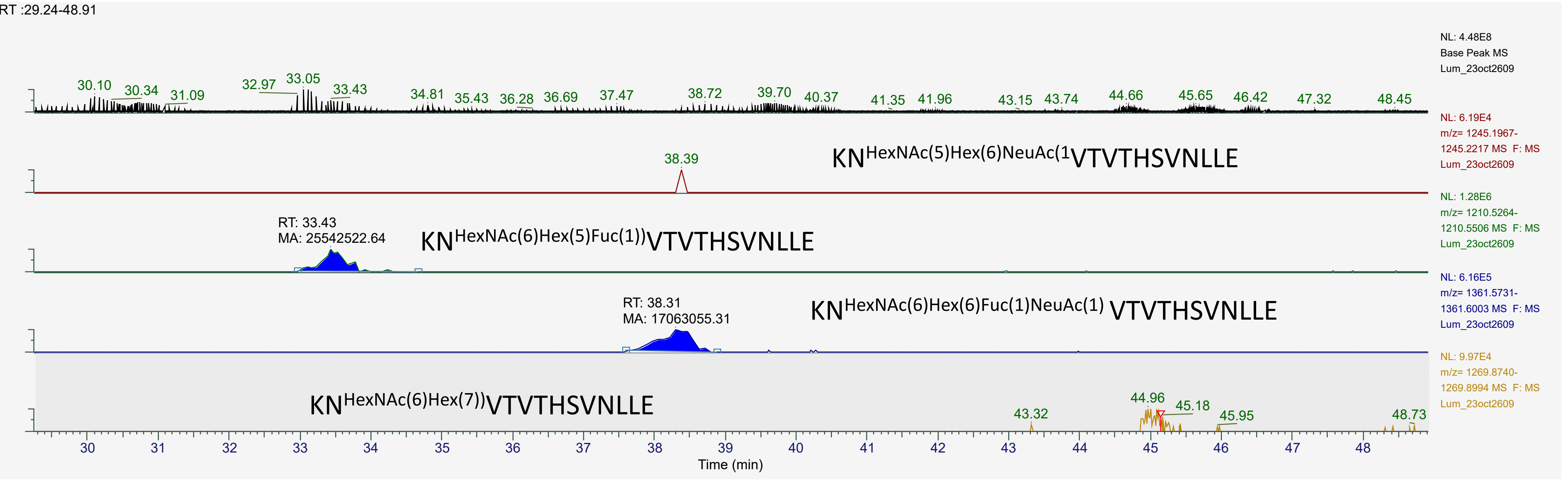

RT (Min)	Base Peak	Peak Area	Peak Height	Baseline Width	SignalToNoise
33.43497	1210.54	25542523	1234648	1.697533	INF
38.30617	1361.588	17063055	589432.6	1.280107	INF

Y2-CHO-S2 Trypsin

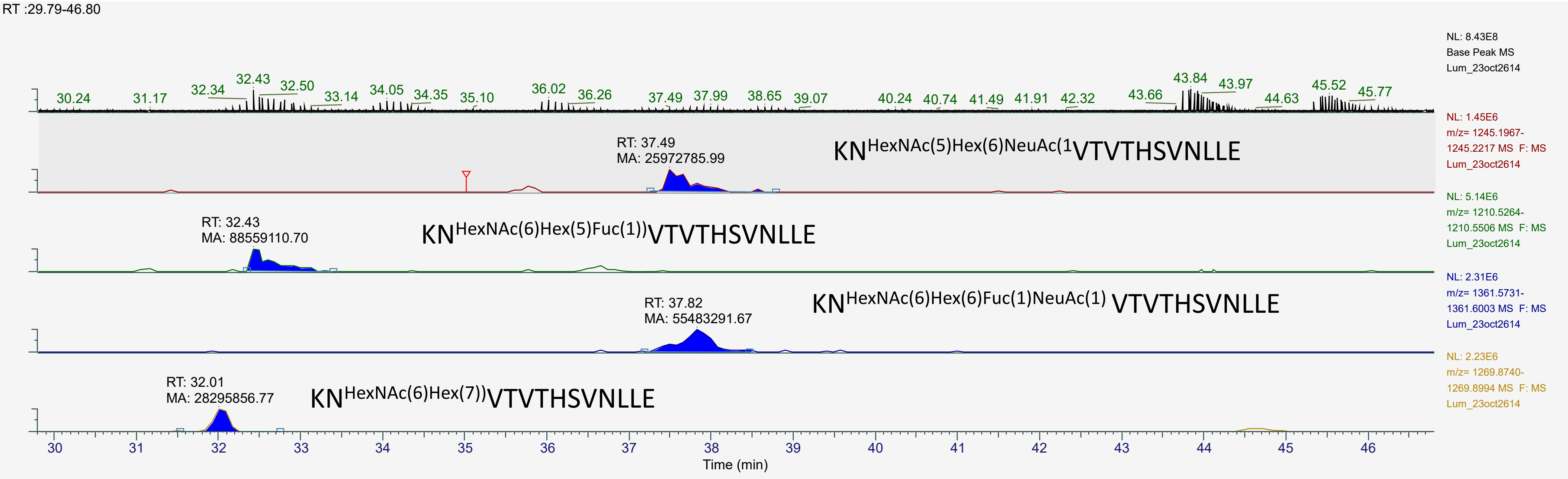

RT (Min)	Base Peak	Peak Area	Peak Height	Baseline Width	SignalToNoise
37.49044	1245.207	25972786	1388393	1.527772	INF
32.42757	1210.538	88559111	4919367	1.058614	INF
37.82492	1361.587	55483292	2270041	1.275149	INF
32.011	1269.887	28295857	2230515	1.215	INF

Y2-CHO-S3 Trypsin

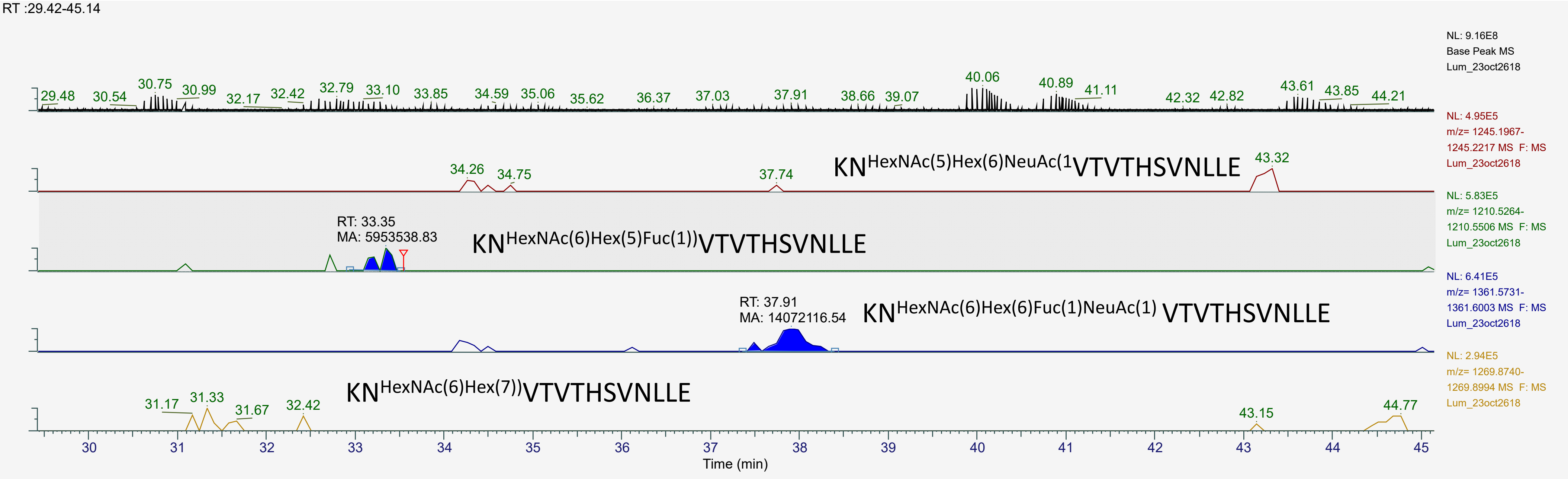

RT (Min)	Base Peak	Peak Area	Peak Height	Baseline Width	SignalToNoise
33.35236	1210.527	5953539	571050.3	0.578152	INF
37.90917	1361.588	14072117	630149.6	1.034002	INF

Y2-CHO-WT Trypsin

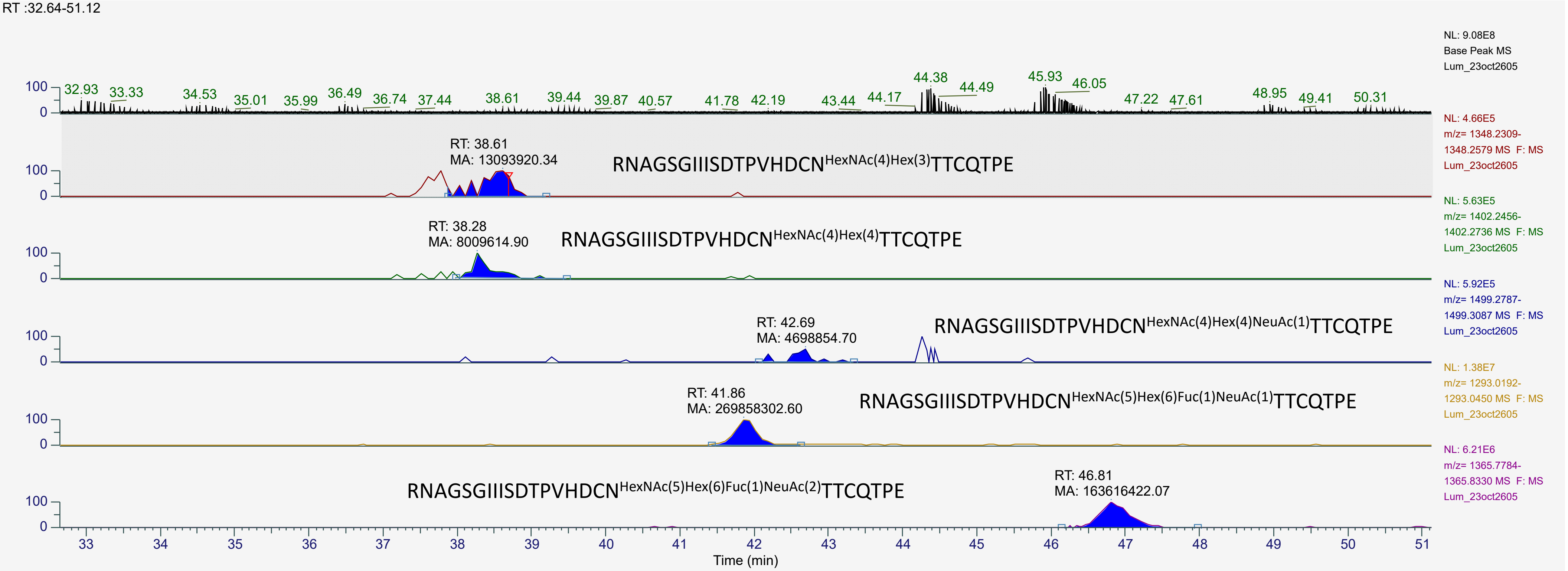

RT (Min)	Base Peak	Peak Area	Peak Height	Baseline Width	SignalToNoise
38.60799	1348.244	13093920	454340.7	1.32	INF
38.27637	1402.263	8009615	533017.7	1.489901	INF
42.68719	1499.296	4698855	281776.9	1.280792	INF
41.85817	1293.034	2.7E+08	13587674	1.202323	INF
46.80643	1365.807	1.64E+08	6213793	1.829622	INF

Y2-CHO-S2 Trypsin

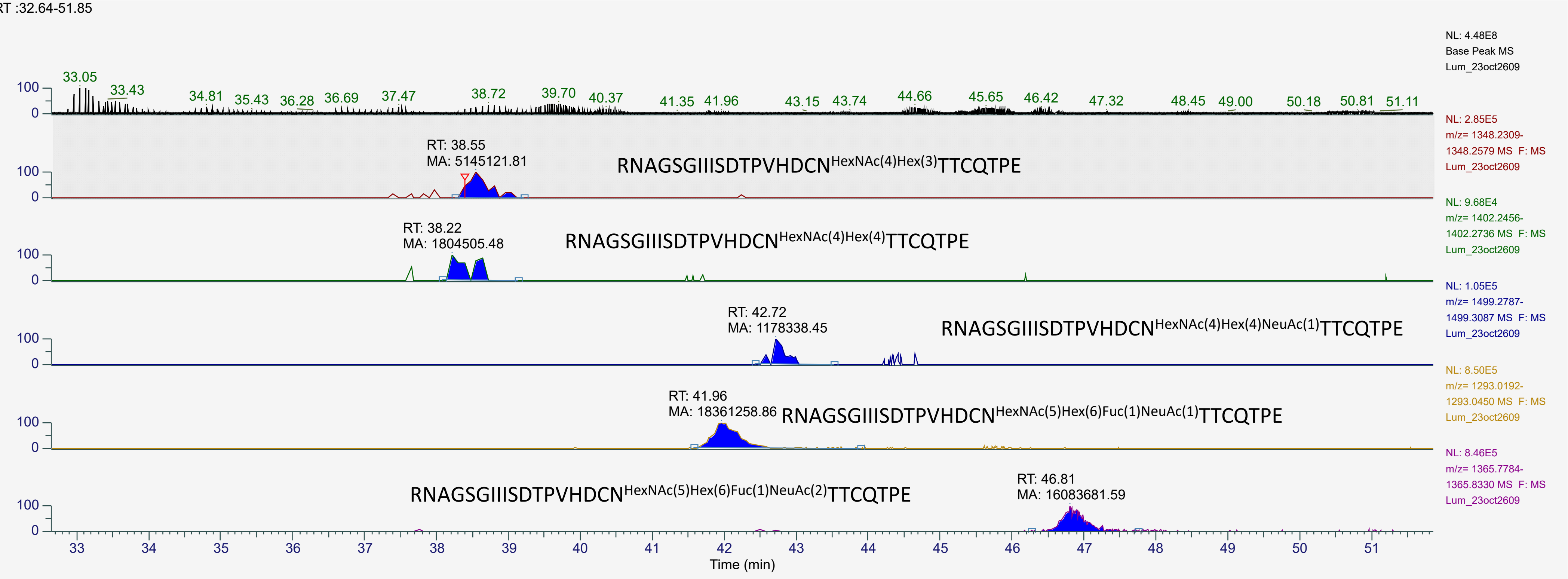

RT (Min)	Base Peak	Peak Area	Peak Height	Baseline Width	SignalToNoise
38.55266	1348.242	5145122	280765.4	0.95099	INF
38.2225	1402.257	1804505	91239.9	1.059675	INF
42.72319	1499.295	1178338	101795.6	1.086846	INF
41.95729	1293.035	18361259	802948.7	2.323737	INF
46.80611	1365.808	16083682	845593.9	1.481213	INF

Y2-CHO-S2 Trypsin

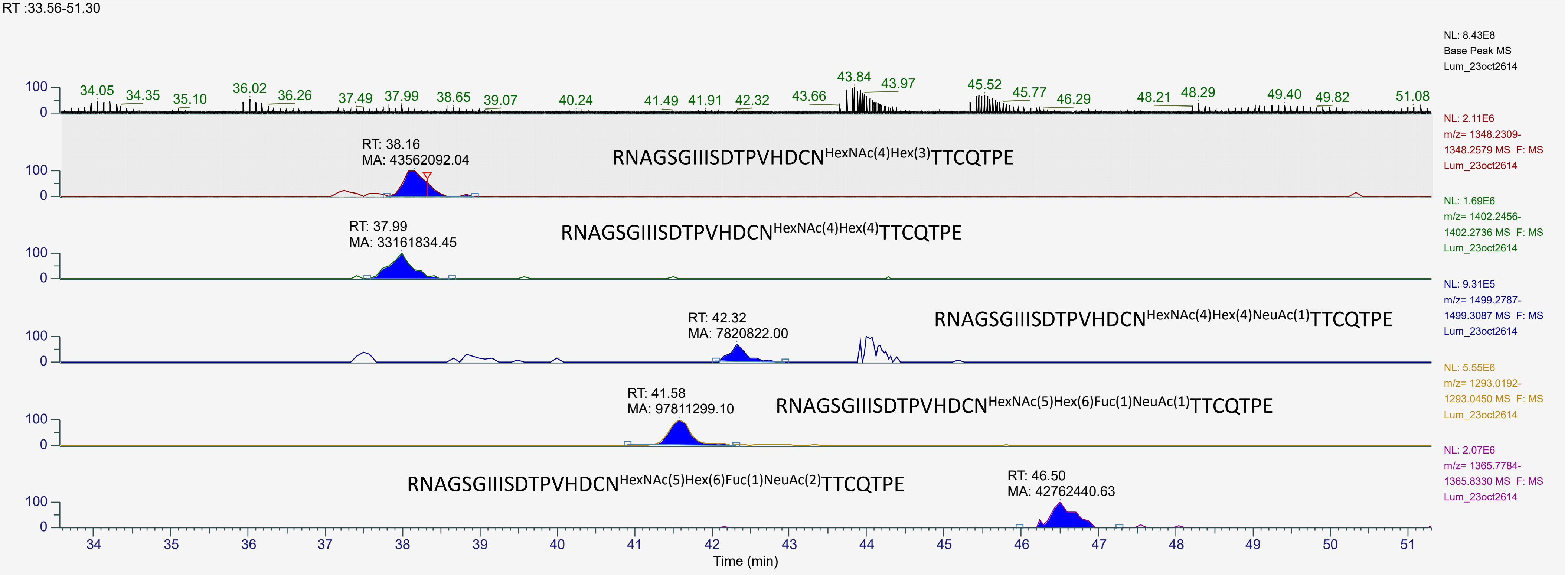

RT (Min)	Base Peak	Peak Area	Peak Height	Baseline Width	SignalToNoise
38.15748	1348.244	43562092	2107698	1.141683	INF
37.99063	1402.26	33161834	1690708	1.104045	INF
42.32436	1499.294	7820822	591032.4	0.90331	INF
41.57756	1293.034	97811299	5363014	1.405452	INF
46.50477	1365.806	42762441	2072241	1.292514	INF

Y2-CHO-S3 Trypsin

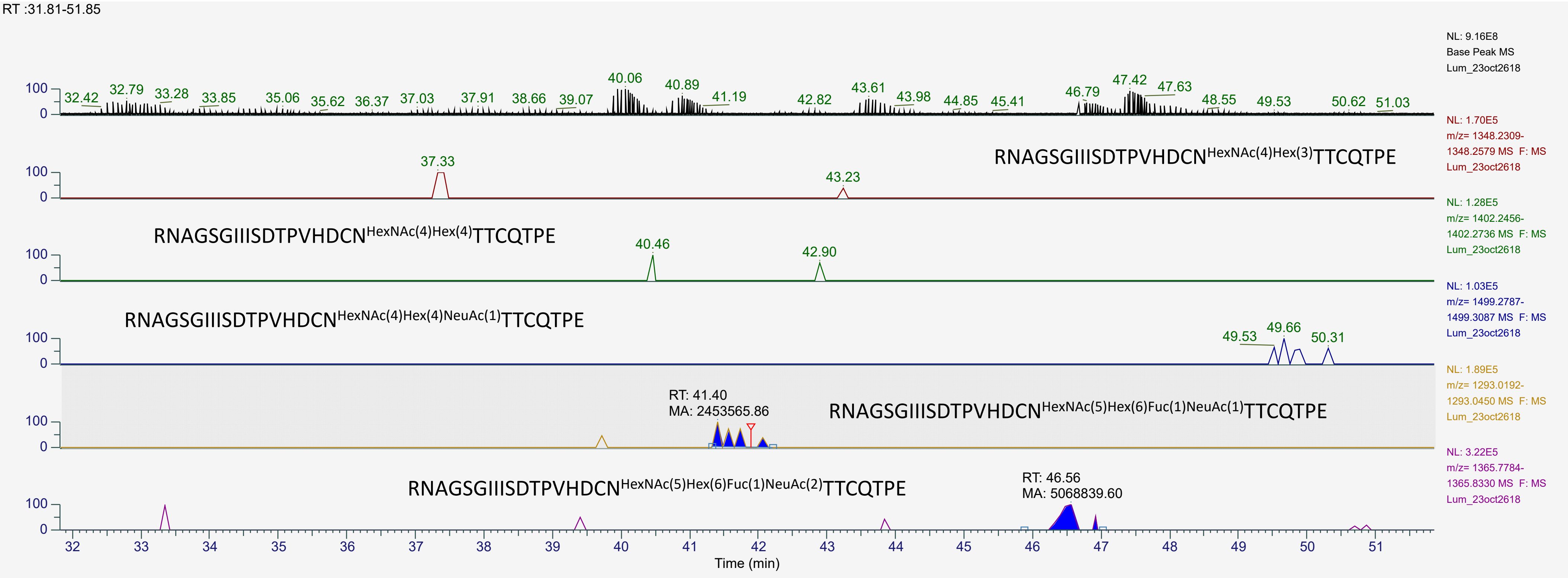

RT (Min)	Base Peak	Peak Area	Peak Height	Baseline Width	SignalToNoise
41.4027	1293.031	2453566	177199.5	0.882502	INF
46.56363	1365.805	5068840	321794.4	1.132962	INF

Y2-CHO-WT Trypsin

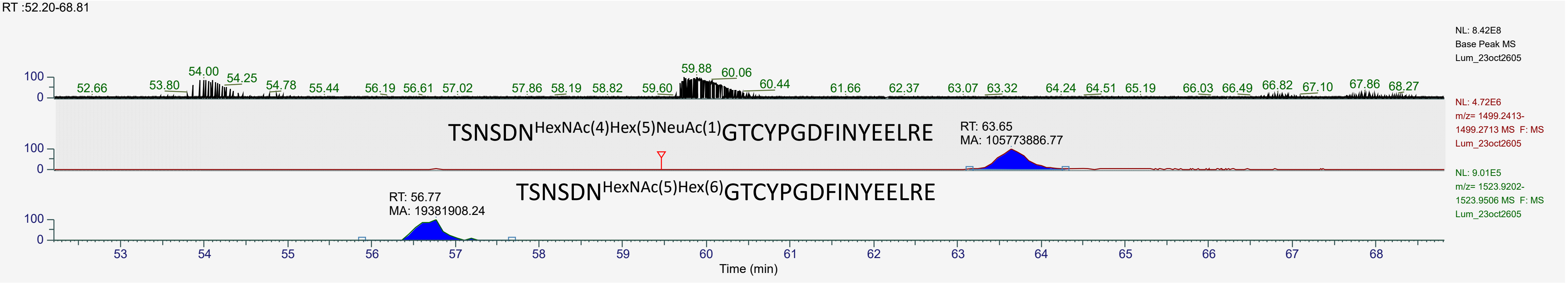

RT (Min)	Base Peak	Peak Area	Peak Height	Baseline Width	SignalToNoise
63.64536	1499.257	1.06E+08	4638812	1.148514	INF
56.77257	1523.936	19381908	900698.8	1.798179	INF

Y2-CHO-S1 Trypsin

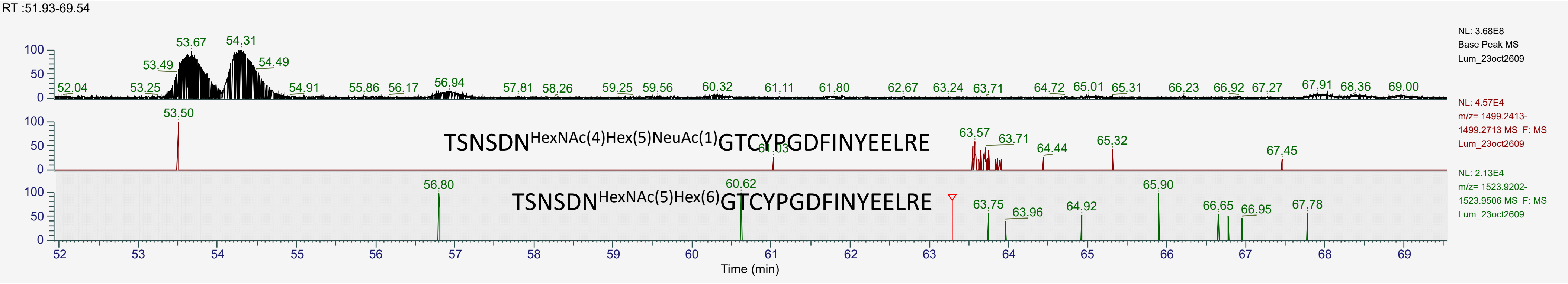

Y2-CHO-S2 Trypsin

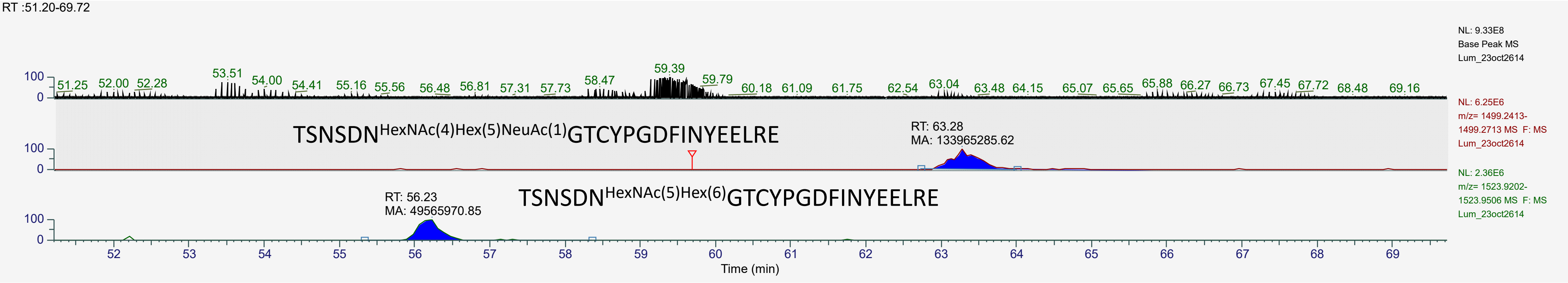

RT (Min)	Base Peak	Peak Area	Peak Height	Baseline Width	SignalToNoise
63.28058	1499.256	1.34E+08	6038223	1.26743	INF
56.22646	1523.937	49565971	2360558	3.039246	INF

Y2-CHO-S3 Trypsin

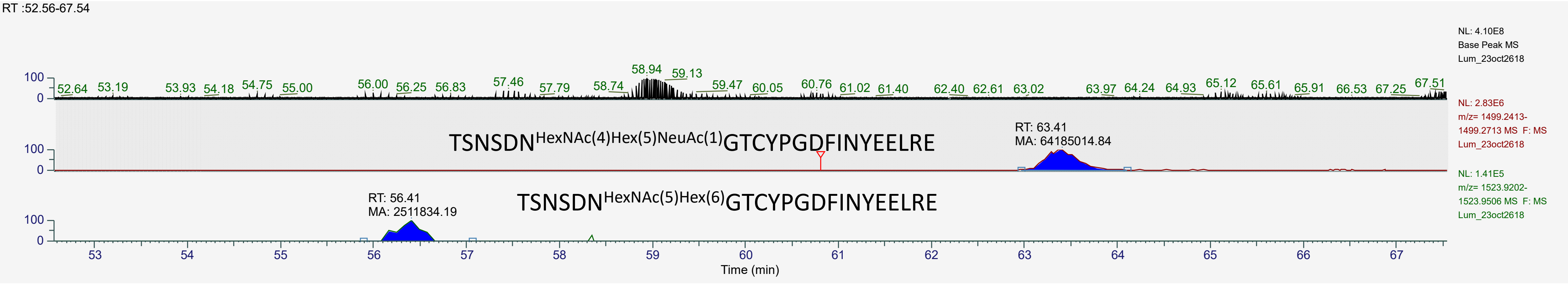

RT (Min)	Base Peak	Peak Area	Peak Height	Baseline Width	SignalToNoise
63.40513	1499.258	64185015	2783202	1.14016	INF
56.41211	1523.932	2511834	140596.1	1.17154	INF

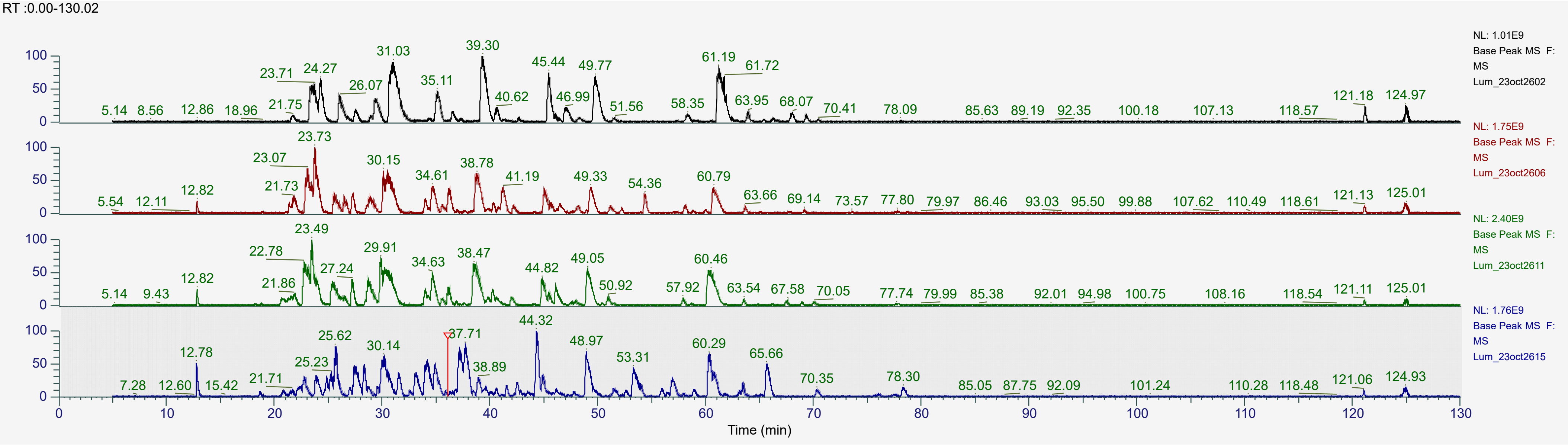
RT :0.00-130.02
NL: 1.01E9
Base Peak MS F: MS
Lum_23oct2602
5.14 8.56 12.86 18.96 21.75 23.71 24.27 26.07 31.03 35.11 39.30 40.62 45.44 46.99 49.77 51.56 58.35 61.19 61.72 63.95 68.07 70.41 78.09 85.63 89.19 92.35 100.18 107.13 118.57 121.18 124.97
NL: 1.75E9
Base Peak MS
Lum_23oct2606
5.54 12.11 12.82 21.73 23.07 23.73 30.15 34.61 38.78 41.19 49.33 54.36 60.79 63.66 69.14 73.57 77.80 79.97 86.46 93.03 95.50 99.88 107.62 110.49 118.61 121.13 125.01
NL: 2.40E9
Base Peak MS F: MS
Lum_23oct2611
5.14 9.43 12.82 21.86 22.78 23.49 27.24 29.91 34.63 38.47 44.82 49.05 50.92 57.92 60.46 63.54 67.58 70.05 77.74 79.99 85.38 92.01 94.98 100.75 108.16 118.54 121.11 125.01
NL: 1.76E9
Base Peak MS F: MS
Lum_23oct2615
7.28 12.60 12.78 15.42 21.71 25.23 25.62 30.14 37.71 38.89 44.32 48.97 53.31 60.29 65.66 70.35 78.30 85.05 87.75 92.09 101.24 110.28 118.48 121.06 124.93
100
50
0
0 10 20 30 40 50 60 70 80 90 100 110 120 130
Time (min)

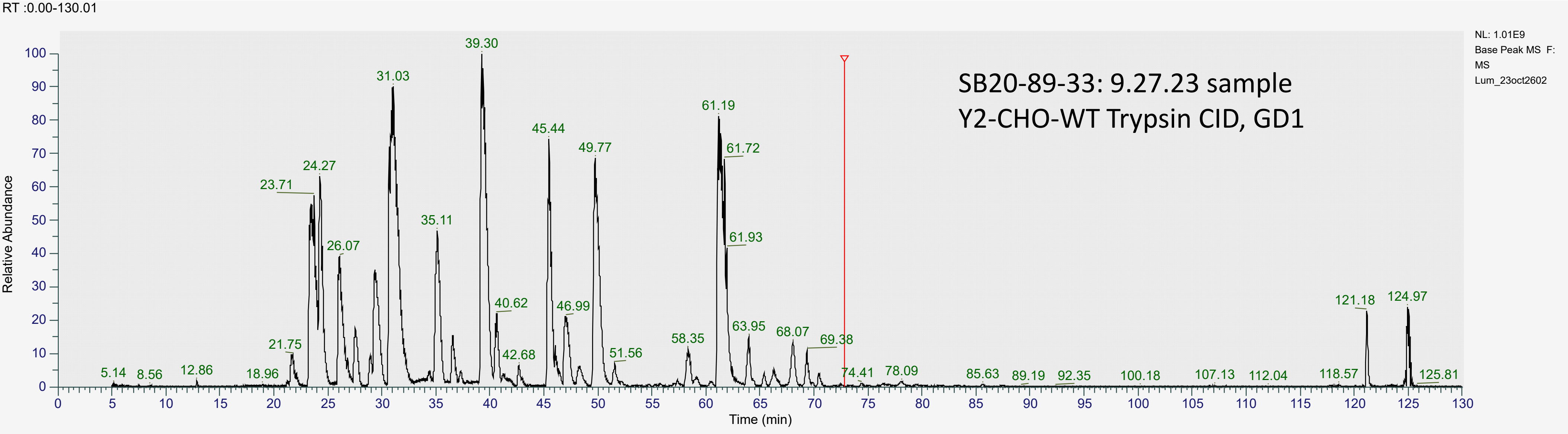
RT :0.00-130.01
SB20-89-33: 9.27.23 sample
Y2-CHO-WT Trypsin CID, GD1
NL: 1.01E9
Base Peak MS F: MS
Lum_23oct2602
Relative Abundance
Time (min)
39.30
31.03
61.19
45.44
49.77
61.72
24.27
23.71
35.11
26.07
61.93
121.18
124.97
40.62
46.99
21.75
63.95
68.07
69.38
58.35
42.68
51.56
5.14
8.56
12.86
18.96
74.41
78.09
85.63
89.19
92.35
100.18
107.13
112.04
118.57
125.81

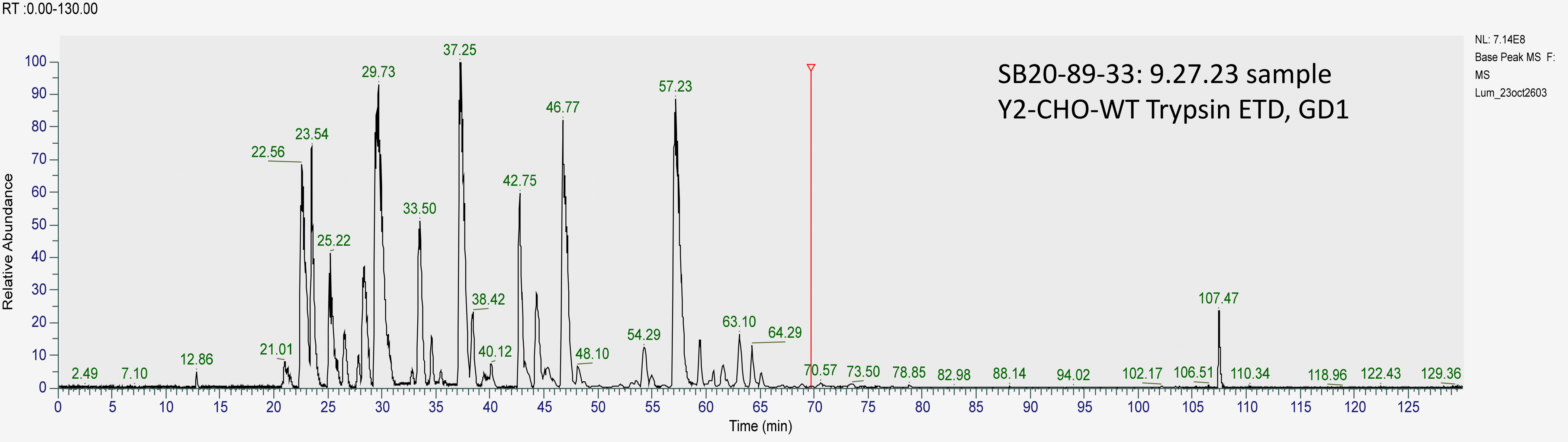
RT :0.00-130.00
SB20-89-33: 9.27.23 sample
Y2-CHO-WT Trypsin ETD, GD1
NL: 7.14E8
Base Peak MS F: MS
Lum_23oct2603
Relative Abundance
Time (min)
37.25
29.73
57.23
46.77
23.54
22.56
42.75
33.50
25.22
38.42
107.47
63.10
64.29
54.29
48.10
40.12
21.01
12.86
2.49
7.10
70.57
73.50
78.85
82.98
88.14
94.02
102.17
106.51
110.34
118.96
122.43
129.36

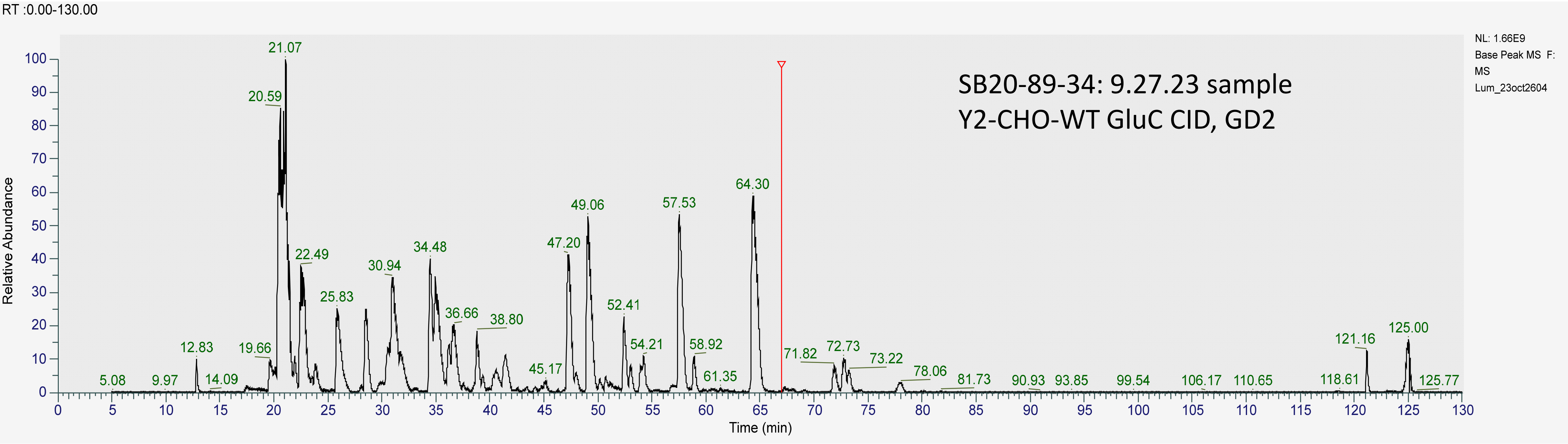
RT :0.00-130.00
SB20-89-34: 9.27.23 sample
Y2-CHO-WT GluC CID, GD2
NL: 1.66E9
Base Peak MS F: MS
Lum_23oct2604
Relative Abundance
Time (min)
21.07
20.59
22.49
25.83
30.94
34.48
36.66
38.80
47.20
49.06
52.41
54.21
57.53
58.92
61.35
64.30
71.82
72.73
73.22
78.06
81.73
90.93
93.85
99.54
106.17
110.65
118.61
121.16
125.00
125.77
5.08
9.97
12.83
14.09
19.66
45.17

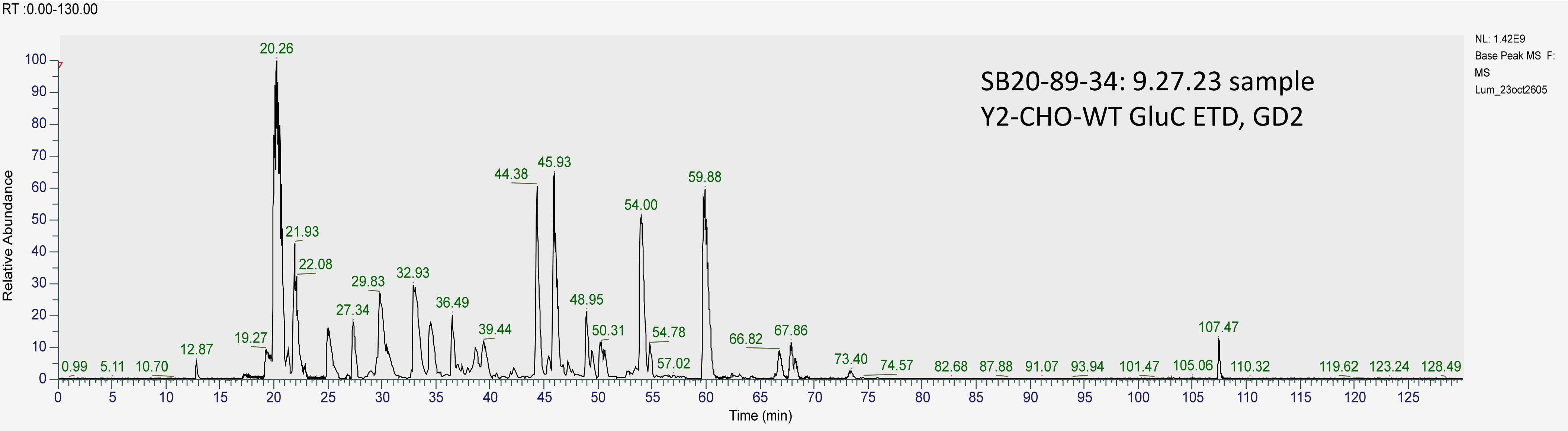
RT :0.00-130.00
SB20-89-34: 9.27.23 sample
Y2-CHO-WT GluC ETD, GD2
NL: 1.42E9
Base Peak MS F: MS
Lum_23oct2605
Relative Abundance
Time (min)
20.26
21.93
22.08
27.34
29.83
32.93
36.49
39.44
44.38
45.93
48.95
50.31
54.00
54.78
57.02
59.88
66.82
67.86
73.40
74.57
82.68
87.88
91.07
93.94
101.47
105.06
107.47
110.32
119.62
123.24
128.49
0.99
5.11
10.70
12.87
19.27

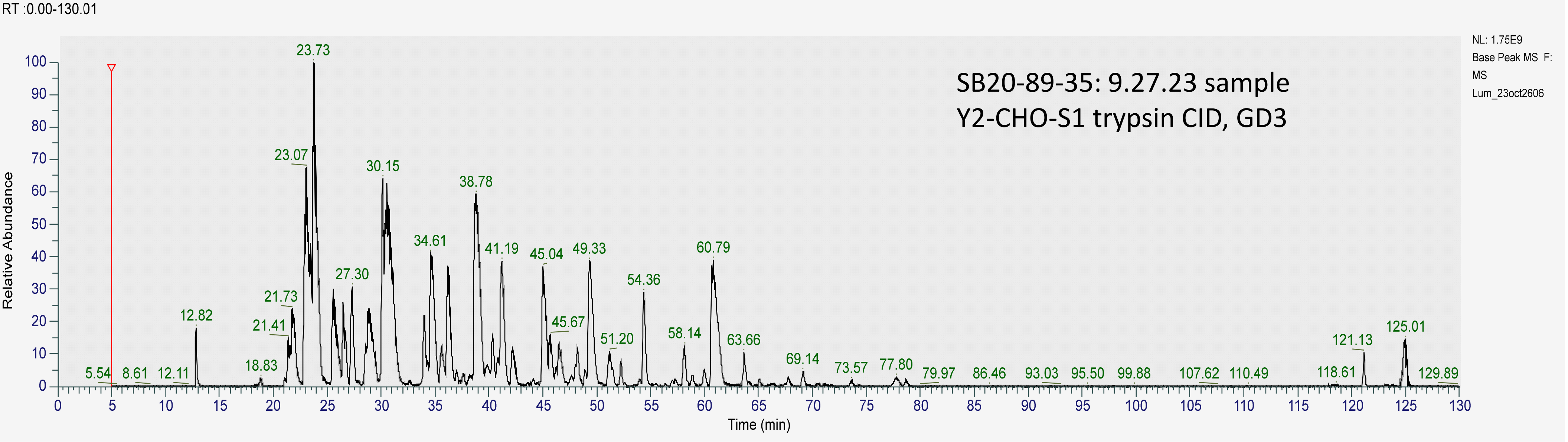
RT :0.00-130.01
NL: 1.75E9
Base Peak MS F: MS
Lum_23oct2606
SB20-89-35: 9.27.23 sample
Y2-CHO-S1 trypsin CID, GD3
Relative Abundance
Time (min)
23.73
23.07
30.15
38.78
34.61
41.19
45.04
49.33
60.79
27.30
54.36
21.73
12.82
21.41
45.67
58.14
51.20
63.66
125.01
121.13
18.83
69.14
73.57
77.80
5.54
8.61
12.11
79.97
86.46
93.03
95.50
99.88
107.62
110.49
118.61
129.89

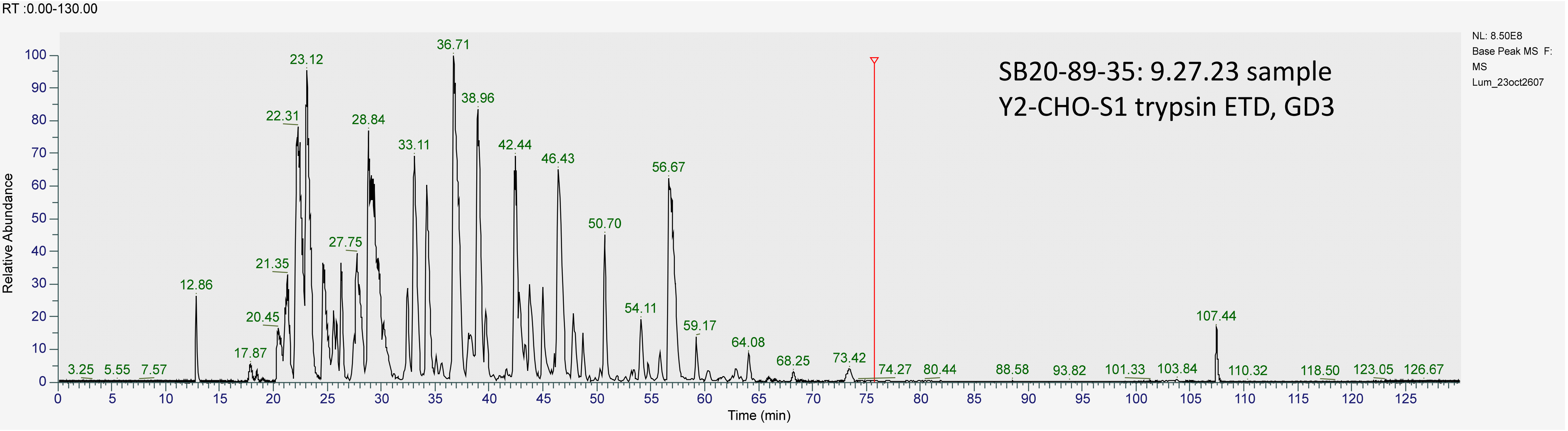
RT :0.00-130.00
NL: 8.50E8
Base Peak MS F: MS
Lum_23oct2607
SB20-89-35: 9.27.23 sample
Y2-CHO-S1 trypsin ETD, GD3
Relative Abundance
Time (min)
36.71
23.12
38.96
22.31
28.84
33.11
42.44
46.43
56.67
50.70
27.75
21.35
12.86
20.45
54.11
59.17
107.44
64.08
17.87
68.25
73.42
3.25
5.55
7.57
74.27
80.44
88.58
93.82
101.33
103.84
110.32
118.50
123.05
126.67

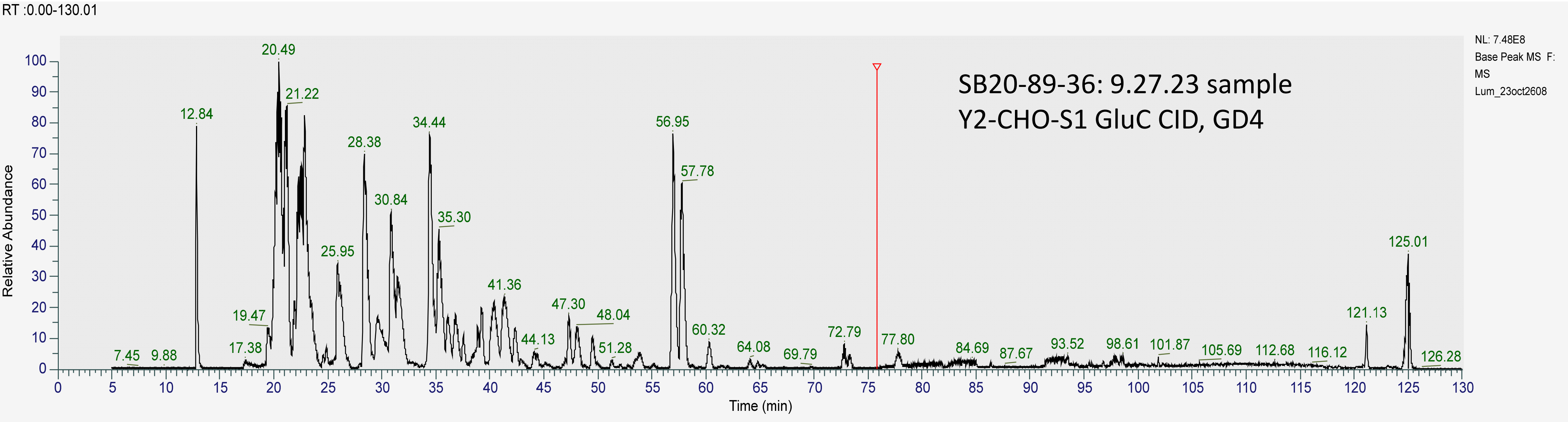
RT :0.00-130.01
NL: 7.48E8
Base Peak MS F: MS
Lum_23oct2608
SB20-89-36: 9.27.23 sample
Y2-CHO-S1 GluC CID, GD4
Relative Abundance
Time (min)

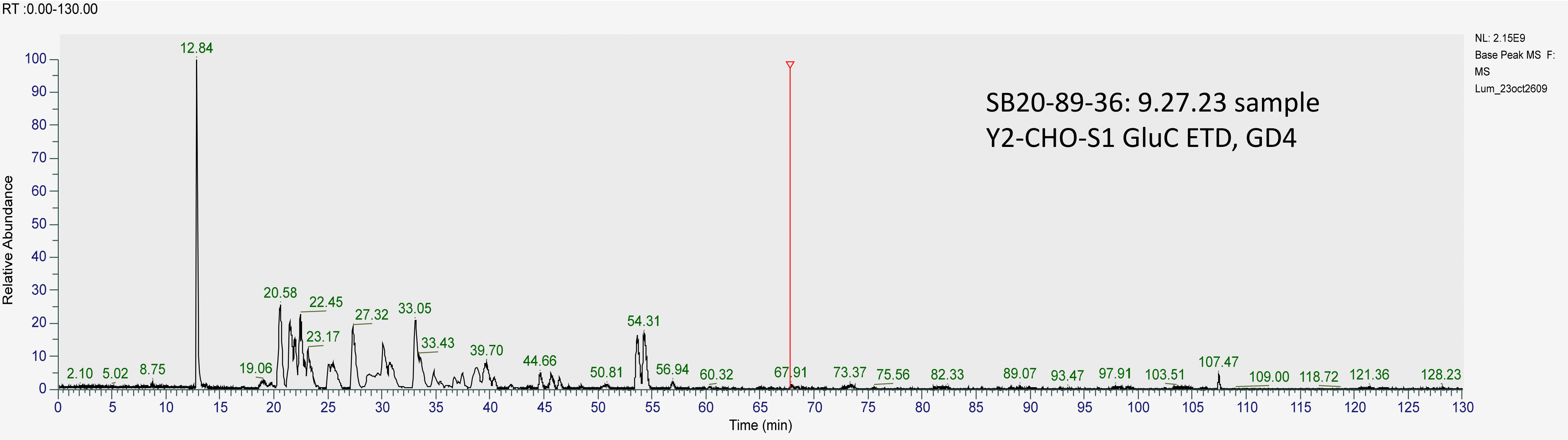
RT :0.00-130.00
NL: 2.15E9
Base Peak MS F: MS
Lum_23oct2609
SB20-89-36: 9.27.23 sample
Y2-CHO-S1 GluC ETD, GD4
Relative Abundance
Time (min)

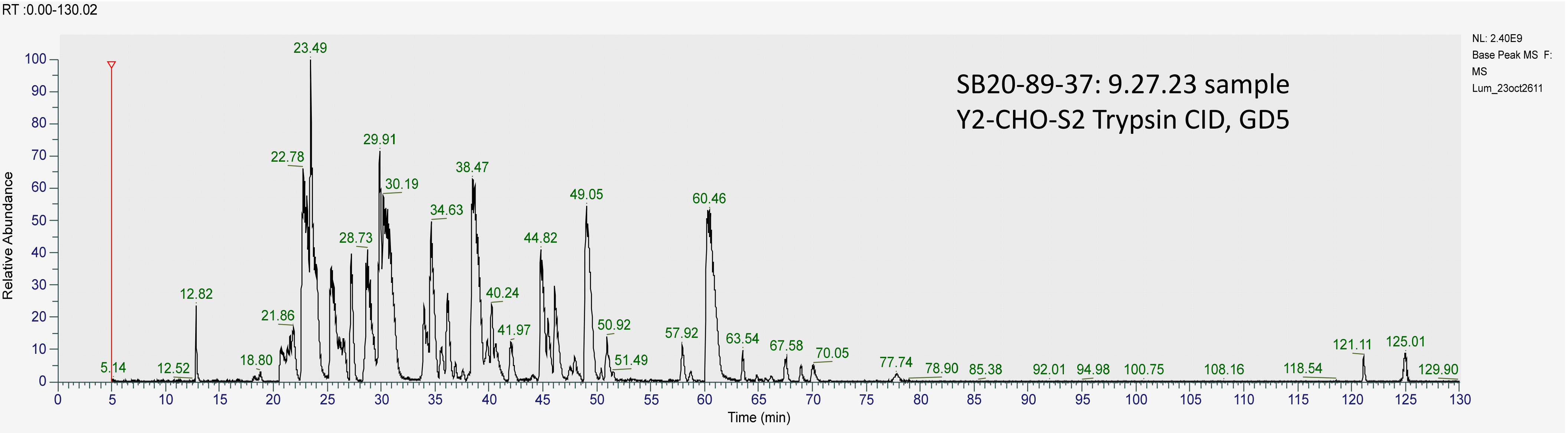
RT :0.00-130.02
NL: 2.40E9
Base Peak MS F: MS
Lum_23oct2611
SB20-89-37: 9.27.23 sample
Y2-CHO-S2 Trypsin CID, GD5
Relative Abundance
Time (min)
23.49
22.78
29.91
30.19
38.47
34.63
49.05
60.46
28.73
44.82
12.82
21.86
40.24
41.97
50.92
57.92
63.54
67.58
70.05
77.74
78.90
85.38
92.01
94.98
100.75
108.16
118.54
121.11
125.01
129.90
5.14
12.52
18.80
51.49

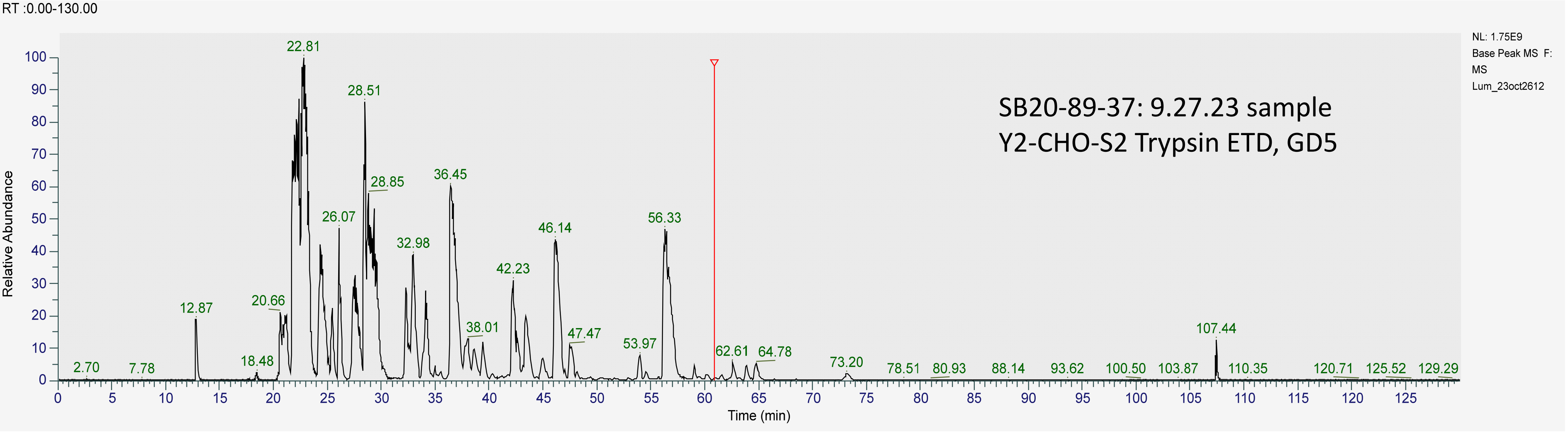
RT :0.00-130.00
NL: 1.75E9
Base Peak MS F: MS
Lum_23oct2612
SB20-89-37: 9.27.23 sample
Y2-CHO-S2 Trypsin ETD, GD5
Relative Abundance
Time (min)
22.81
28.51
28.85
36.45
26.07
32.98
46.14
56.33
42.23
12.87
20.66
38.01
47.47
53.97
62.61
64.78
73.20
107.44
2.70
7.78
18.48
78.51
80.93
88.14
93.62
100.50
103.87
110.35
120.71
125.52
129.29

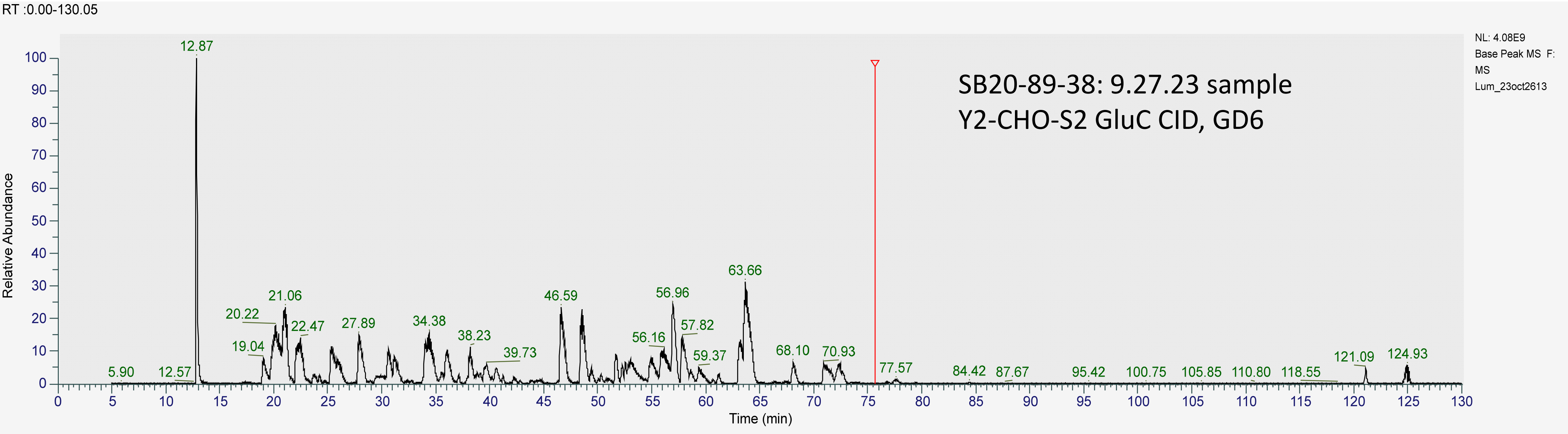
RT :0.00-130.05
NL: 4.08E9
Base Peak MS F: MS
Lum_23oct2613
SB20-89-38: 9.27.23 sample
Y2-CHO-S2 GluC CID, GD6
Relative Abundance
Time (min)
12.87
21.06
20.22
22.47
19.04
27.89
34.38
38.23
39.73
46.59
56.96
56.16
57.82
59.37
63.66
68.10
70.93
77.57
84.42
87.67
95.42
100.75
105.85
110.80
118.55
121.09
124.93
5.90
12.57

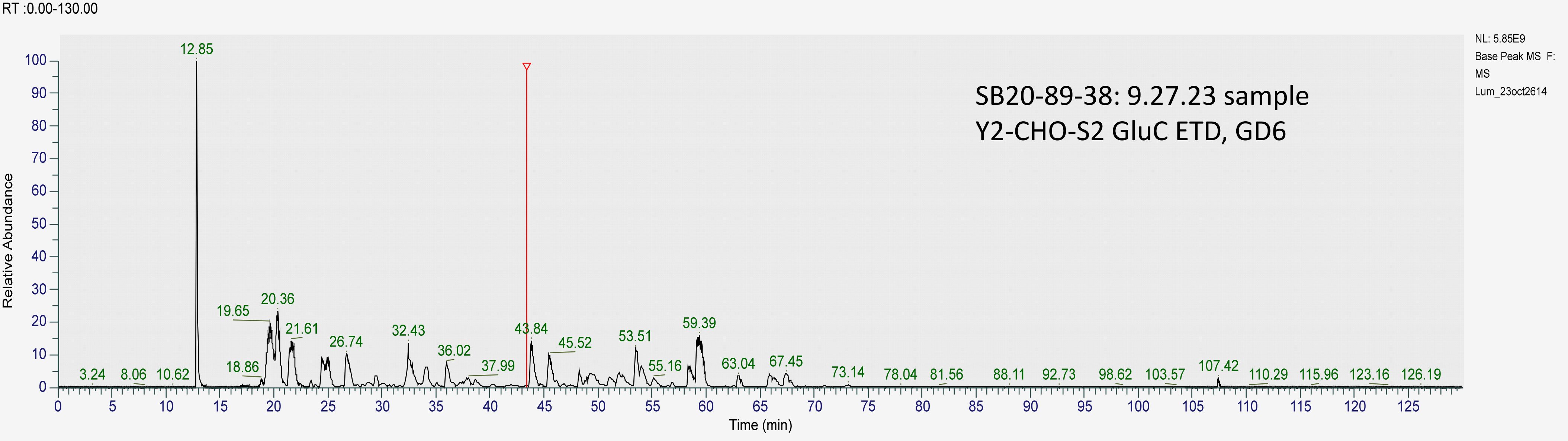
RT :0.00-130.00
NL: 5.85E9
Base Peak MS F: MS
Lum_23oct2614
SB20-89-38: 9.27.23 sample
Y2-CHO-S2 GluC ETD, GD6
Relative Abundance
Time (min)
12.85
20.36
19.65
21.61
18.86
26.74
32.43
36.02
37.99
43.84
45.52
53.51
55.16
59.39
63.04
67.45
73.14
78.04
81.56
88.11
92.73
98.62
103.57
107.42
110.29
115.96
123.16
126.19
3.24
8.06
10.62

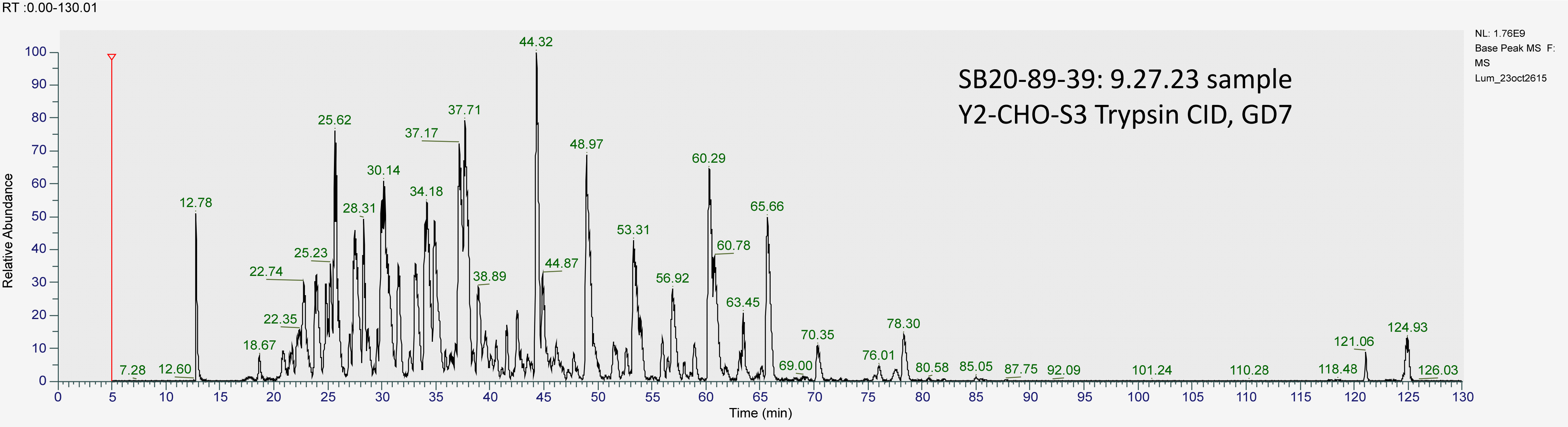
RT :0.00-130.01
NL: 1.76E9
Base Peak MS F: MS
Lum_23oct2615
SB20-89-39: 9.27.23 sample
Y2-CHO-S3 Trypsin CID, GD7
Relative Abundance
Time (min)
44.32
37.71
25.62
37.17
48.97
60.29
30.14
34.18
12.78
28.31
65.66
53.31
25.23
60.78
22.74
44.87
38.89
56.92
63.45
22.35
18.67
70.35
78.30
124.93
121.06
76.01
69.00
80.58
85.05
87.75
92.09
101.24
110.28
118.48
126.03
7.28
12.60

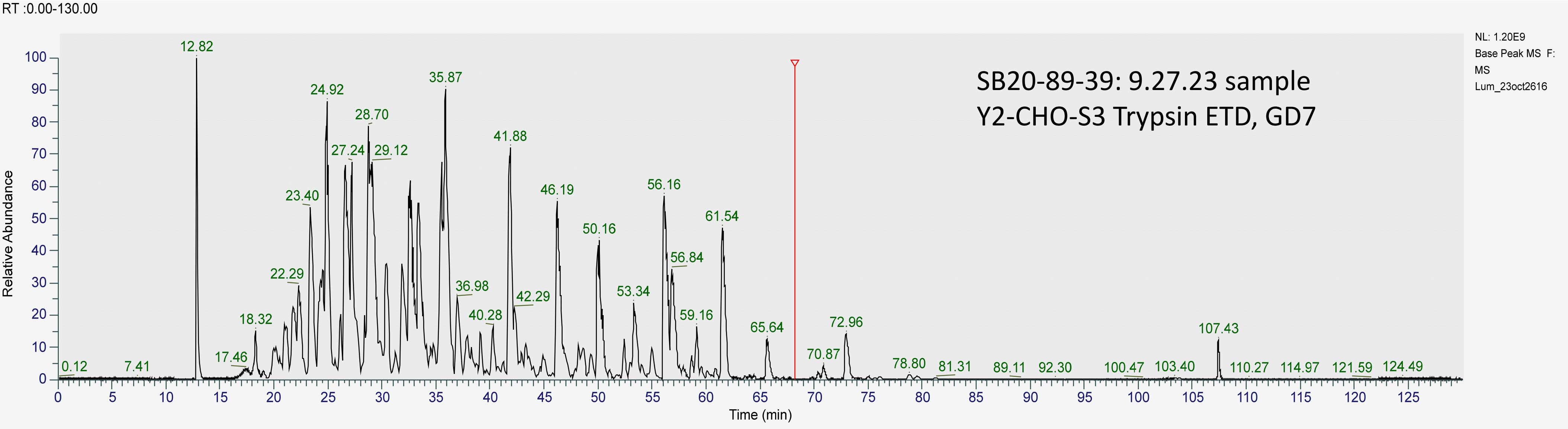
RT :0.00-130.00
NL: 1.20E9
Base Peak MS F: MS
Lum_23oct2616
SB20-89-39: 9.27.23 sample
Y2-CHO-S3 Trypsin ETD, GD7
Relative Abundance
Time (min)
12.82
35.87
24.92
28.70
41.88
27.24
29.12
46.19
56.16
23.40
61.54
50.16
56.84
22.29
36.98
42.29
53.34
18.32
40.28
59.16
65.64
72.96
107.43
17.46
70.87
78.80
81.31
89.11
92.30
100.47
103.40
110.27
114.97
121.59
124.49
0.12
7.41

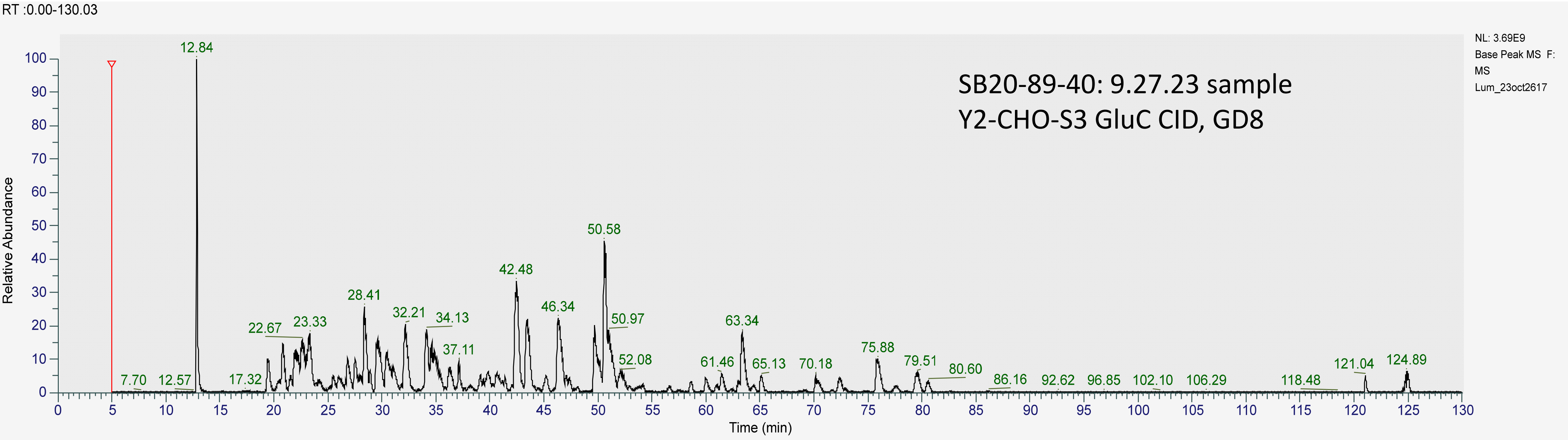

RT :0.00-130.03
NL: 3.69E9
Base Peak MS F: MS
Lum_23oct2617
SB20-89-40: 9.27.23 sample
Y2-CHO-S3 GluC CID, GD8
Relative Abundance
Time (min)
12.84
50.58
42.48
28.41
46.34
32.21
34.13
22.67
23.33
50.97
63.34
37.11
52.08
75.88
61.46
65.13
70.18
79.51
80.60
124.89
121.04
7.70
12.57
17.32
86.16
92.62
96.85
102.10
106.29
118.48

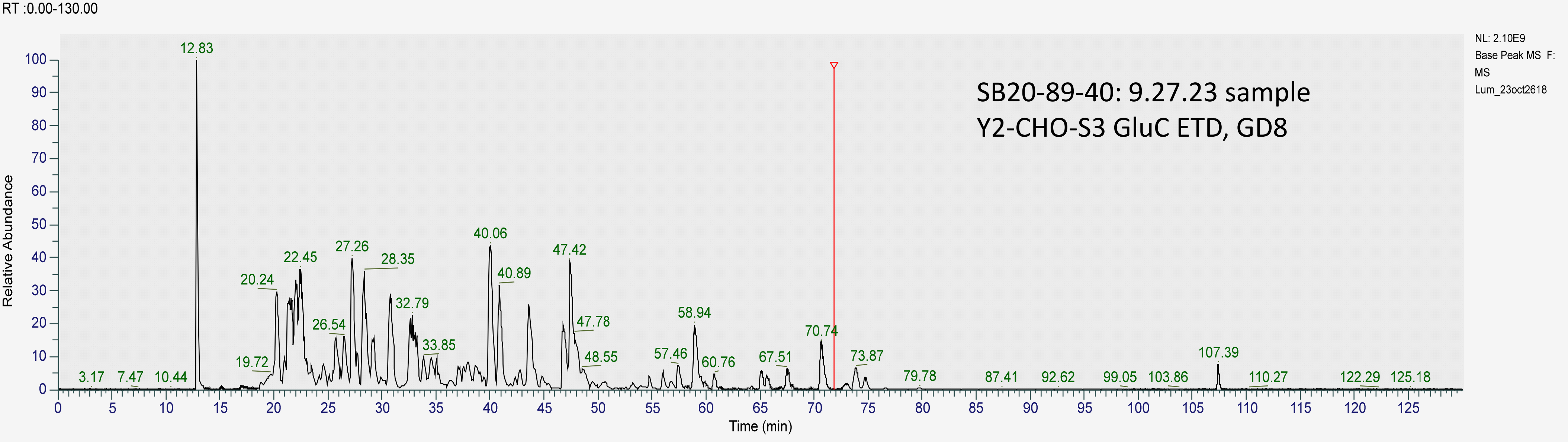

RT :0.00-130.00
NL: 2.10E9
Base Peak MS F: MS
Lum_23oct2618
SB20-89-40: 9.27.23 sample
Y2-CHO-S3 GluC ETD, GD8
Relative Abundance
Time (min)
12.83
40.06
47.42
27.26
22.45
28.35
40.89
20.24
32.79
58.94
26.54
47.78
70.74
33.85
57.46
48.55
67.51
73.87
19.72
60.76
107.39
3.17
7.47
10.44
79.78
87.41
92.62
99.05
103.86
110.27
122.29
125.18
