# Supplementary material for: Immunogenicity of the Foldon trimerization domain and efforts to shield it
Source: PLoS One. 2026 Nov 13;21(11):e0362571. doi: 10.1371/journal.pone.0362571 (PMC13766278; doi:10.1371/journal.pone.0362571)
Supplement: Supplementary file 6 [file pone.0362571.s005.zip › S11_Data/Report number 5232 - Ross Spenser.pdf]

Lerner Research Institute
Mass Spectrometry Laboratory for Protein Sequencing
The Cleveland Clinic Foundation

Belinda Willard, Ph.D., Director
216-444-7170, willarb@ccf.org

Report number: 5232
Title: Sequence of analysis of four protein samples: Hemagglutinin
Date: 7-24-2024

Principal Investigator: Dr. Ted Ross, Global Director for Vaccine development
For: Spencer Pierce

Samples prepared by: Autumn Saddler
Reported by: Belinda Willard
Approved by: Belinda Willard
Charges: 1 Protein PTM @ $ 260 = $260
iLab Service ID: PMC-SP-1874

Brief description of samples and goal of the experiment.

The samples were a four expressed protein samples. Table 1 contains a summary of the samples analyzed in this set. The goal of these studies is to identify sites of glycosylation.

Table 1. Samples received.[a]

	Investigator sample name	Digest sample number	MS file number	Method
			Orbi	
1	Ross-Pierce 5.15.24 in-soln sample Y2-CHO-S3 trypsin	SB20-136-59	Lum_24jun04<br>Lum_24jun04	CID<br>ETD
2	Ross-Pierce 5.15.24 in-soln sample Y2-CHO-S3 GluC	SB20-136-60	Lum_24jun04<br>Lum_24jun04	CID<br>ETD

Methods:

One protein sample was submitted for in-solution protein digestion each contain 10 ug of protein with volumes ranged from 7 ul to 24 ul. The volumes for these samples were adjusted to 24 ul using 0.1M TrisHCl pH 8 buffer. A 30 ul aliquot of 8M urea, 0.1M TrisHCl pH 8 buffer was added to each sample. The samples were reduced by adding 2 ul 0.2M dithiothreitol and incubated at room temperature for 30 minutes. Reduction was followed by alkylation using a 9.6 ul aliquot of 0.2M iodoacetamide and incubating at room temperature in the dark for 30 minutes. The alkylation reaction was quenched by adding 8 ul 0.2M dithiothreitol and incubating at room temperature for 30 minutes. The pH of the samples were adjusted to 8 with 1M triethyl ammonium bicarbonate. The sample volume was adjused to 240 ul using deionized water. The samples were then split into 2 aliquots of 120 ul each. These samples were digested by adding 0.1 ug sequencing grade trypsin or Glu_C and incubating the samples overnight at 37°C. After digestion, the samples were desalted using C18 Ziptips, the eluted samples were dried on a SpeedVac, then reconstituted in 30 ul 0.1% formic acid for LC-MS analysis.

The LC-MS system was a Finnigan LTQ-Obitrap Fusion Lumos hybrid mass spectrometer system. The HPLC column was a Dionex 15 cm x 75 µm id Acclaim Pepmap C18, 2µm, 100 Å reversed-phase capillary chromatography column. Five µL volumes of the extract were injected and the peptides eluted from the column by an acetonitrile/0.1% formic acid gradient at a flow rate of

0.25 µL/min were introduced into the source of the mass spectrometer on-line. The microelectrospray ion source is operated at 2.5 kV. The digest was analyzed using the data dependent multitask capability of the instrument acquiring full scan mass spectra to determine peptide molecular weights and product ion spectra to determine amino acid sequence in successive instrument scans. The data were analyzed by using all CID spectra collected in the experiment to search the human reference sequence database with the search program Mascot. All data was filtered based on a Mascot ion score > 40. In addition, the data was searched specifically against the sequence of mouse and human Akt1 using the program Sequest and considering phosphorylation, methylation, ubiquitination, acetylation, and glycosylation.

Results:

One protein sample was submitted on 5.15.24. These samples were digested with trypsin and GluC. The digests were analyzed by capillary column LC-tandem MS and the CID spectra searched against the sequence of Hemagglutinin using the program Sequest. These samples were analyzed utilizing an LC gradient from 2 to 70% acetonitrile in 110 minutes. The results are summarized in Table 2, with the specific peptides that were sequenced shown in the attached figures.

SB20-136-59: 5.15.24 in-soln sample Y2-CHO-S3 trypsin

The data from this digest was searched specifically against the expressed protein sequences. The S3 form of Hemagglutinin was identified by 85 peptides covering 86% of the protein sequence.

SB20-136-60: 5.15.24 in-soln sample Y2-CHO-S3 GluC

The data from this digest was searched specifically against the expressed protein sequences. The S3 form of Hemagglutinin was identified by 267 peptides covering 96% of the protein sequence.

Chromatographic analysis

The previous analysis identified several semi-tryptic peptides in the S3 sample. Based on this result, the data from this sample was researched considering semi-tryptic protein digestion. The number of tryptic and semi-tryptic peptides identified in these samples is summarized below. The results from the current S3 sample is more in-line with the results from the WT, S1, and S2 samples from the initial set of samples from report 5037.

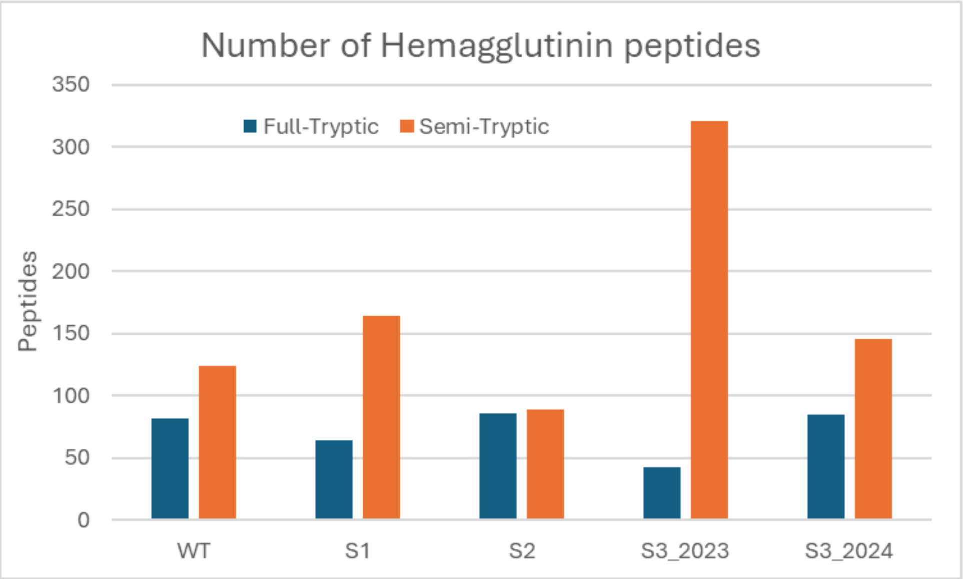

Post Translational modification analysis

The goal of these experiments is to quantify sites of hemagglutinin glycosylation. The CID data was searched specifically against the sequences of Hemagglutinin looking for HexNac modification using the program Sequest. A total of 5 HexNac modified peptides were identified and are given in Table 3. The more complex sugar modifications cannot be identified with CID fragmentation. Due to this, the samples were analyzed using EThcD fragmentation, a method more commonly used for glycosylation analysis. The EThcD data was searched using the program MSFragger specifically against the sequences of Hemagglutinin considering N-linked glycosylation as a variable modification. Thirteen glycosylated peptides were identified and are given in Table 4. These peptides were glycosylated on three different residues including N23, N276, and N481.

The goal of these experiments were to compare the glycosylation between this sample and the previously analyzed samples. The previous analysis identified several glycosylated peptides that were not identified in the analysis of this sample. To directly compare the two sets of samples, chromatograms were plotted for all of the peptides given in Table 4 as well as the glycosylated peptides identified in report 5037. For all positively identified peptides, single ion monitoring (SIM) chromatograms were plotted for each modified peptide, these chromatograms were integrated, and the resulting peak areas were used to estimate the site specific relative abundance of each glycosylation event

N481: SVKNGTYDYPKYSEE

The previous analysis identified 42 unique N481 containing tryptic peptides (see Table 5). Chromatograms were plotted for all 42 previously identified N481 peptides and six of these peptides were identified in this sample. These results were combined with the eight GluC N481 peptides identified in this sample to determine the site-specific relative abundance of glycosylation on this site. This data indicates that the HexNAc(2)Hex(5) and HexNAc(4)Hex(3)Fuc(1) forms of this peptide are the most abundant.

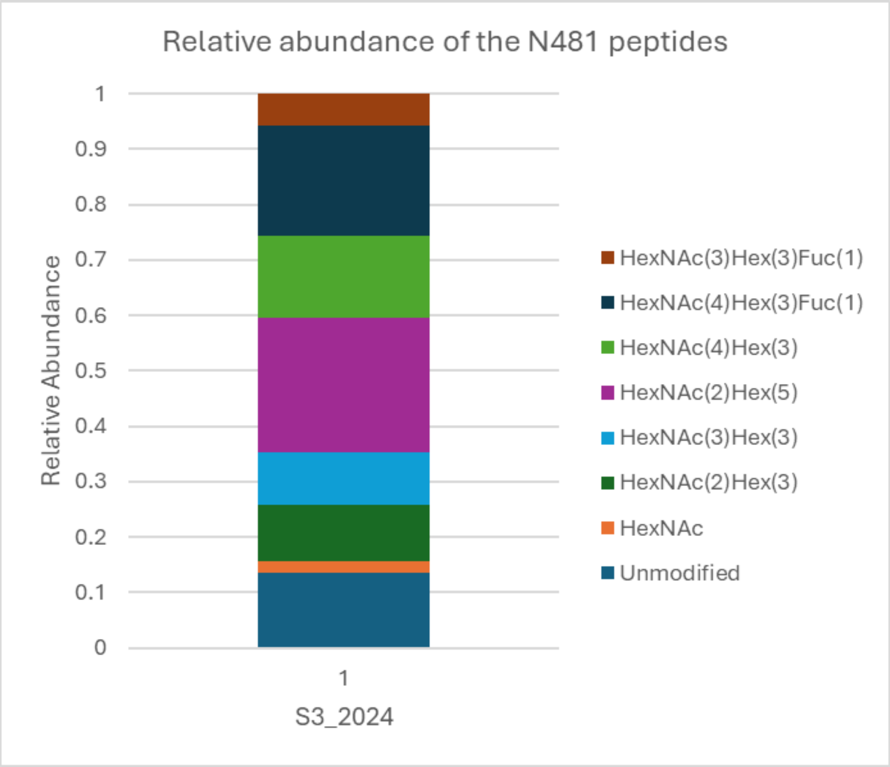

N276-N287: NAGSGIIISDTPVHDCNTTCQTPEGAINTSLPFQNVHPITIGK

The previous analysis identified 10 unique N276/N287 containing peptides (see Table 7). Chromatograms were plotted for all 10 previously identified peptides and only the unmodified form of this peptide was identified.

N23: KNVTVTHSVNLLE

The previous analysis identified 50 unique N23 containing peptides (see Table 8). Chromatograms were plotted for all 50 previously identified peptides and 28 of these peptides were identified in this sample. These results were combined with the single N23 peptide identified in this sample to determine the site specific relative abundance of glycosylation on this site. This data indicates that the unmodified and HexNAc(5)Hex(6)Fuc(1) forms of this peptide are the most abundant.

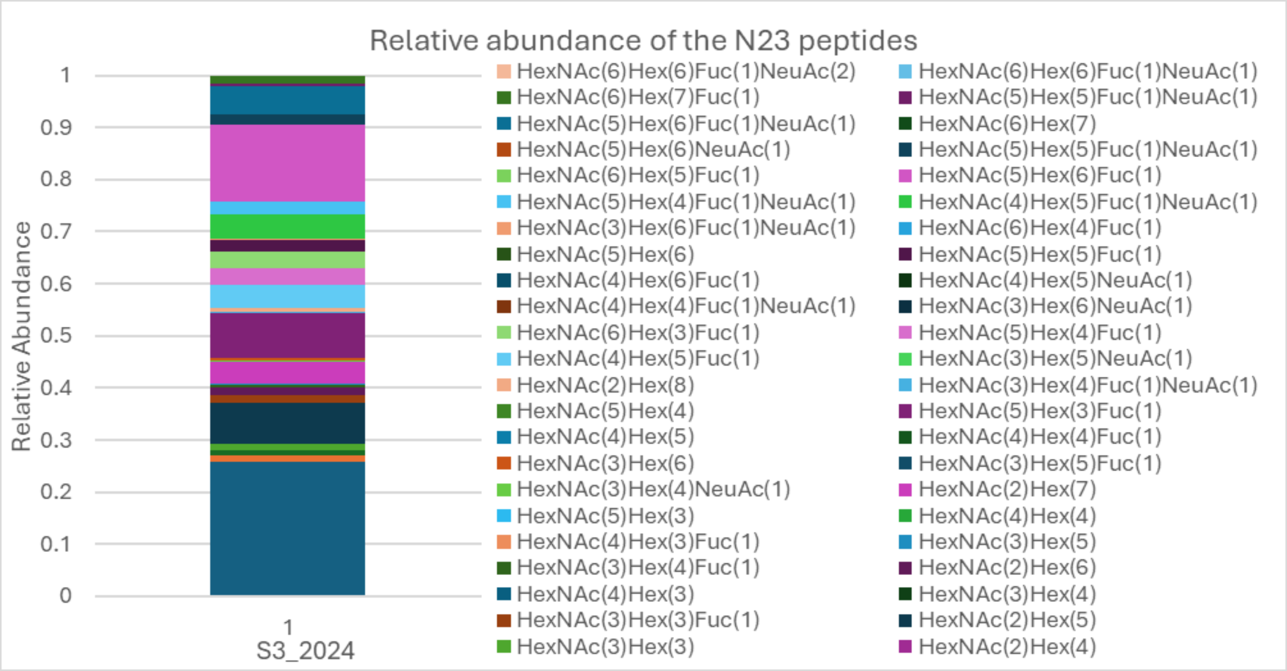

N276: RNAGSGIIISDTPVHDCNTTCQTPE

The previous analysis identified 41 unique N276 containing peptides (see Table 9). Chromatograms were plotted for all 41 previously identified peptides and 26 of these peptides were identified in this sample. These results were combined with the four N276 peptide identified in this sample to determine the site-specific relative abundance of glycosylation on this site. This data indicates that the unmodified and HexNAc(4)Hex(5)Fuc(1) forms of this peptide are the most abundant.

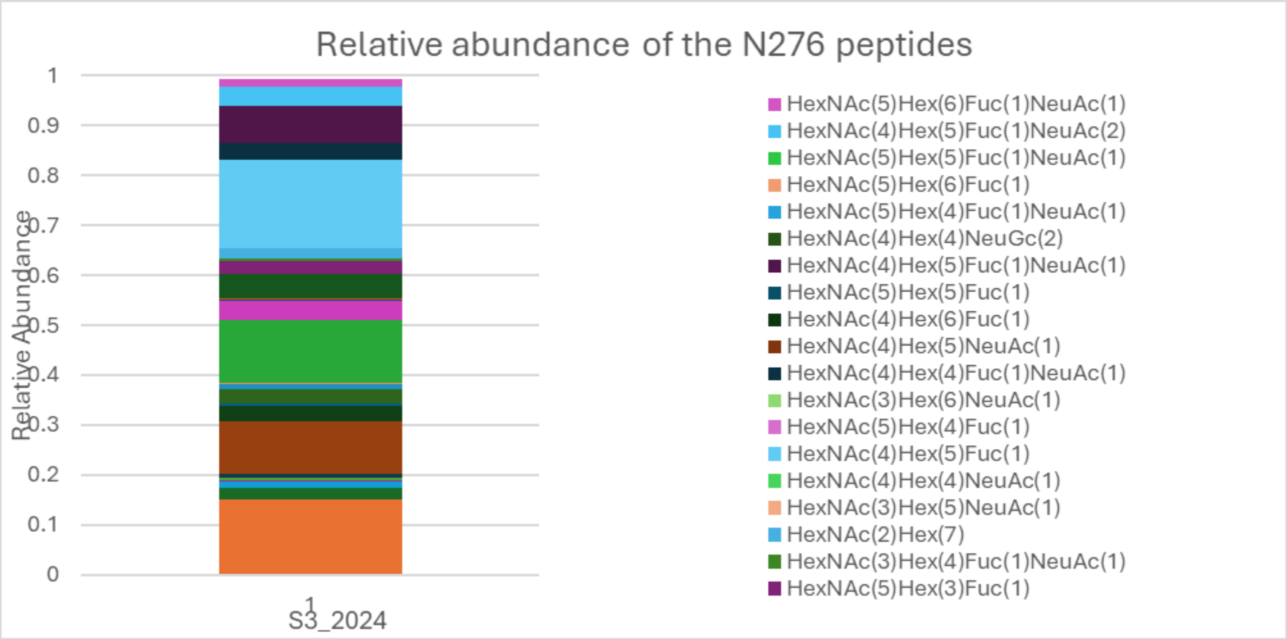

N87: TSNSDNGTCYPGDFINYEE

The previous analysis identified 18 unique N87 containing peptides (see Table 10). Chromatograms were plotted for all 18 previously identified peptides and 11 of these peptides were identified in this sample. Chromatographic analysis was performed to determine the site-specific relative abundance of glycosylation on this site. This data indicates that the unmodified and HexNAc(5)Hex(3) forms of this peptide are the most abundant.

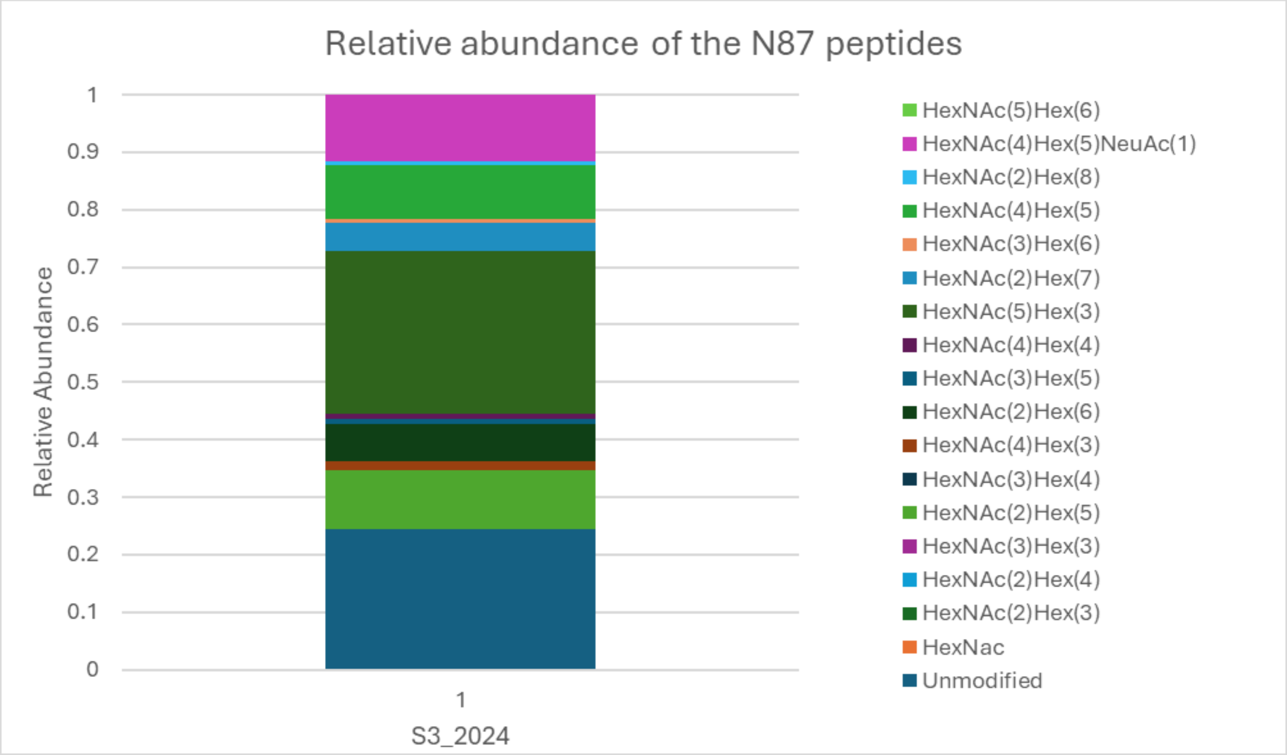

N287: TSNSDNGTCYPGDFINYEE

The previous analysis identified 2 unique N287 containing peptides (see Table 11). Chromatograms were plotted for all previously identified peptides and only the unmodified form of this peptide was identified.

N11: DTLCIGYHANNSTDTVDTVLE

The previous analysis identified 16 unique N11 containing peptides (see Table 12). Chromatograms were plotted for all previously identified peptides and only the HexNac form of this peptide was identified.

N513: EKIDGVGTGYIPEAPNDTQAYVRK

The previous analysis identified 18 unique N513 containing peptides (see Table 13). Chromatograms were plotted for all these peptides and none of the EKIDGVGTGYIPEAPNDTQAYVRK were identified in this sample.

N515: IDGVGTGYIPEAPRDNQSYVR and EKIDGVGTGYIPEAPRDNQSYVR

The previous analysis identified 21 unique N515 containing peptides (see Table 14). Chromatograms were plotted for all these peptides and none of the IDGVGTGYIPEAPRDNQSYVR or EKIDGVGTGYIPEAPRDNQSYVR peptides were identified in this sample.

Conclusion

One protein sample was submitted for in-solution digestion and LC-MS/MS analysis. This sample was digested with both trypsin and GluC and the LC-MS/MS data from these digests was searched against the sequences of Hemagglutinin submitted by the investigator. Hemagglutinin was identified in the tryptic digests by 85 peptides covering 86% of the protein sequences and by 267 GluC peptides covering 96% of the protein sequence.

The goal of these experiments is to compare glycosylation between this sample and the previously analyzed samples (see report 5037). The data from these samples was searched looking for glycosylation and while several glycosylated peptides were identified (see Tables 3 and 4), several of the previously identified glycopeptides were not identified in the analysis. To directly compare the two sets of samples, chromatograms were plotted for all peptides identified in the current and previous experiments. For all positively identified peptides, single ion monitoring (SIM) chromatograms were plotted for each modified peptide, these chromatograms were integrated, and the resulting peak areas were used to estimate the site-specific relative abundance of each glycosylation event. These results are summarized in Tables 5 to 14 in the attached spreadsheet.

A summary of total glycosylation sites identified in the current and previous LC-MS/MS analysis is given below.

	WT	S1	S2	S3_2023	S3_2024
N11	15	0	15	2	1
N23	49	47	48	30	27
N87	16	14	16	17	11
N276	40	40	40	10	26
N287	1	0	1	1	0
N481	28	28	27	8	7
N513	0	17	0	0	0
N515	0	0	15	0	0

Table 2. Summary of results

Sample Name	Sample number	Protein	Accession	Gene	Mass kDa	Pep	Seq. Cov	Sequest Score
5.15.24 Y2-CHO S3 Trypsin	SB20-136-59	Hemagglutinin Y2.CHO.S3	C3W5S1	HA	62.5	85	86	5155.9
		Hemagglutinin Y2.CHO S3 (semitryptic search)				231	95	6343.9
5.15.24 Y2-CHO S3 GluC	SB20-136-60	Hemagglutinin Y2.CHO.wt<br>O	C3W5S1	HA	62.5	267	96	7128.3

SB20-136-59: 5.15.24 in-soln sample Y2-CHO-S3 trypsin

Protein MW: **62520**
Amino Acid Composition: **A29 C12 D28 E37 F19 G45 H22 I31 K42 L41 M5 N44 P21 Q16 R18 S38 T38 V33 W11 Y26**

```
1   DTLCIGYHAN NSTDTVDTVL EKNVTVTHSV NLLEDKHNGK LCKLRGVAPL HLGKCNIAGW ILGNPECESL STASSWSYIV
81  ETSNSDNGTC YPGDFINYEE LREQLSSVSS FERFEIFPKT SSWPNHDSNK GVTAACPHAG AKSFYKNLIW LVKKGNSYPK
161 LSQSYINDKG KEVLVLWGIH HPSTTADQQS LYQNADAYVF VGTSRYSKKF KPEIAIRPKV RDQEGRMNYY WTLVEPGDKI
241 TFEATGNLVV PRYAFTMERN AGSGIIISDT PVHDCNTTCQ TPEGAINTSL PFQNVHPITI GKCPKYVKST KLRLATGLRN
321 VPSIQSRGLF GAIAGFIEGG WTGMVDGWYG YHHQNEQGSG YAADLKSTQN AIDKITNKVN SVIEKMNTQF TAVGKEFNHL
401 EKRIENLNKK VDDGFLDIWT YNAELLVLLE NERTLDYHDS NVKNLYEKVR NQLKNNAKEI GNGCFEFYHK CDNTCMESVK
481 NGTYDYPKYS EEAKLNREKI DGVGTGYIPE APRDGQAYVR KDGNWTLLST FLGSGLNDIF EAQKIEWHEG HHHHHH
```

SB20-89-40: 9.27.23 in-soln sample Y2-CHO-S3 GluC

Protein MW: **62520**
Amino Acid Composition: **A29 C12 D28 E37 F19 G45 H22 I31 K42 L41 M5 N44 P21 Q16 R18 S38 T38 V33 W11 Y26**

```
1   DTLCIGYHAN NSTDTVDTVL EKNVTVTHSV NLLEDKHNGK LCKLRGVAPL HLGKCNIAGW ILGNPECESL STASSWSYIV
81  ETSNSDNGTC YPGDFINYEE LREQLSSVSS FERFEIFPKT SSWPNHDSNK GVTAACPHAG AKSFYKNLIW LVKKGNSYPK
161 LSQSYINDKG KEVLVLWGIH HPSTTADQQS LYQNADAYVF VGTSRYSKKF KPEIAIRPKV RDQEGRMNYY WTLVEPGDKI
241 TFEATGNLVV PRYAFTMERN AGSGIIISDT PVHDCNTTCQ TPEGAINTSL PFQNVHPITI GKCPKYVKST KLRLATGLRN
321 VPSIQSRGLF GAIAGFIEGG WTGMVDGWYG YHHQNEQGSG YAADLKSTQN AIDKITNKVN SVIEKMNTQF TAVGKEFNHL
401 EKRIENLNKK VDDGFLDIWT YNAELLVLLE NERTLDYHDS NVKNLYEKVR NQLKNNAKEI GNGCFEFYHK CDNTCMESVK
481 NGTYDYPKYS EEAKLNREKI DGVGTGYIPE APRDGQAYVR KDGNWTLLST FLGSGLNDIF EAQKIEWHEG HHHHHH
```

SB20-136-59 and 60: 5.15.24 in-soln sample Y2-CHO-S3 trypsin, GluC, trypsin and GluC (95% coverage)

Protein MW: **62520**
Amino Acid Composition: **A29 C12 D28 E37 F19 G45 H22 I31 K42 L41 M5 N44 P21 Q16 R18 S38 T38 V33 W11 Y26**

```
1   DTLCIGYHAN NSTDTVDTVL EKNVTVTHSV NLLEDKHNGK LCKLRGVAPL HLGKCNIAGW ILGNPECESL STASSWSYIV
81  ETSNSDNGTC YPGDFINYEE LREQLSSVSS FERFEIFPKT SSWPNHDSNK GVTAACPHAG AKSFYKNLIW LVKKGNSYPK
161 LSQSYINDKG KEVLVLWGIH HPSTTADQQS LYQNADAYVF VGTSRYSKKF KPEIAIRPKV RDQEGRMNYY WTLVEPGDKI
241 TFEATGNLVV PRYAFTMERN AGSGIIISDT PVHDCNTTCQ TPEGAINTSL PFQNVHPITI GKCPKYVKST KLRLATGLRN
321 VPSIQSRGLF GAIAGFIEGG WTGMVDGWYG YHHQNEQGSG YAADLKSTQN AIDKITNKVN SVIEKMNTQF TAVGKEFNHL
401 EKRIENLNKK VDDGFLDIWT YNAELLVLLE NERTLDYHDS NVKNLYEKVR NQLKNNAKEI GNGCFEFYHK CDNTCMESVK
481 NGTYDYPKYS EEAKLNREKI DGVGTGYIPE APRDGQAYVR KDGNWTLLST FLGSGLNDIF EAQKIEWHEG HHHHHH
```

Y2-CHO-S3 trypsin

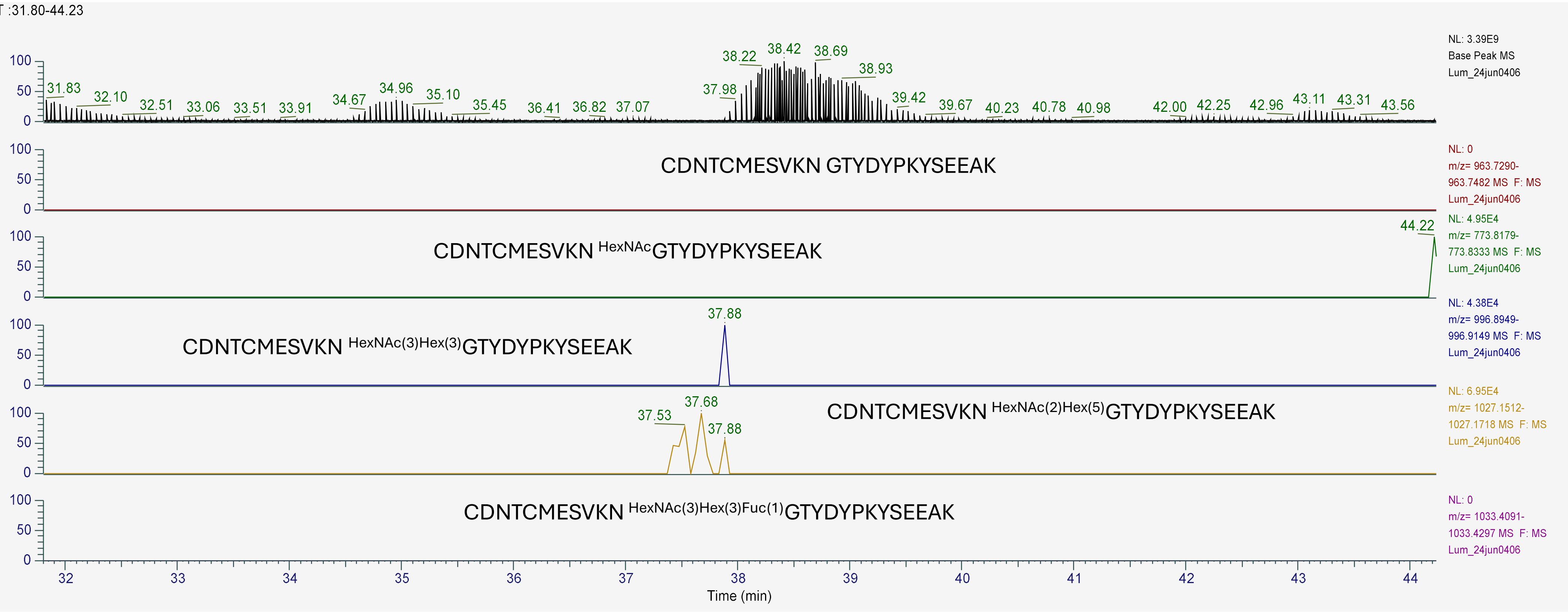

Y2-CHO-S3 trypsin

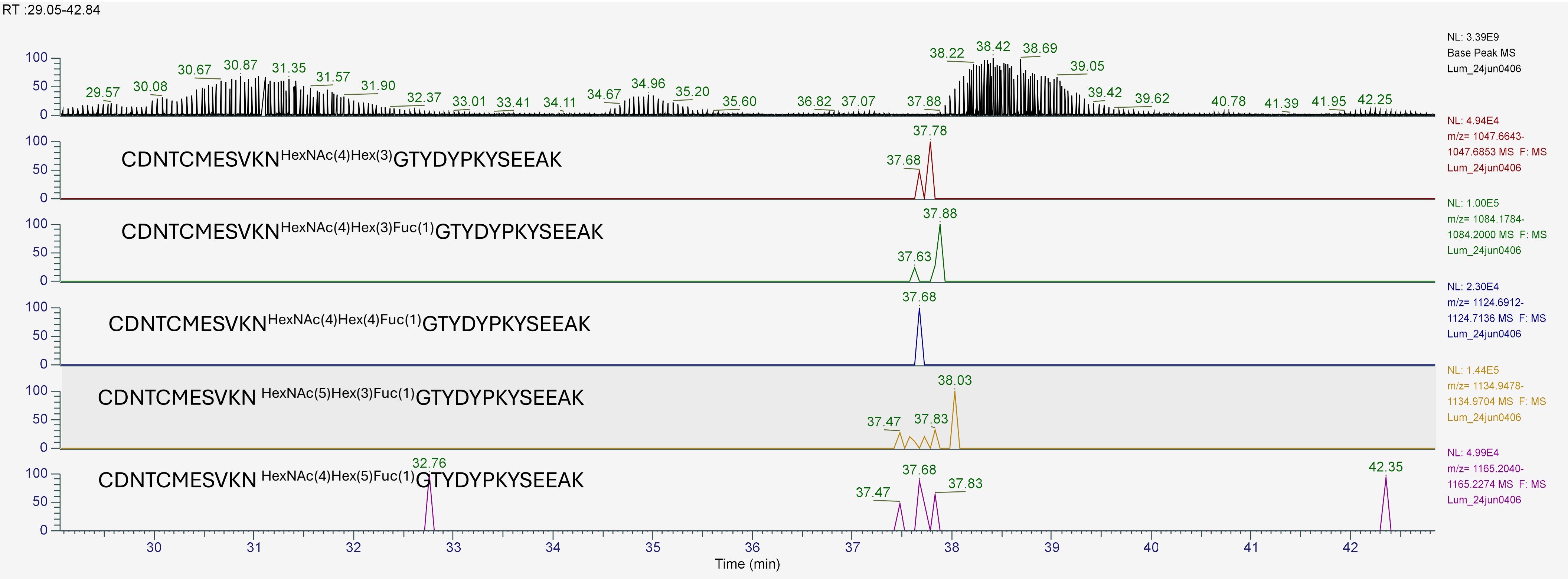

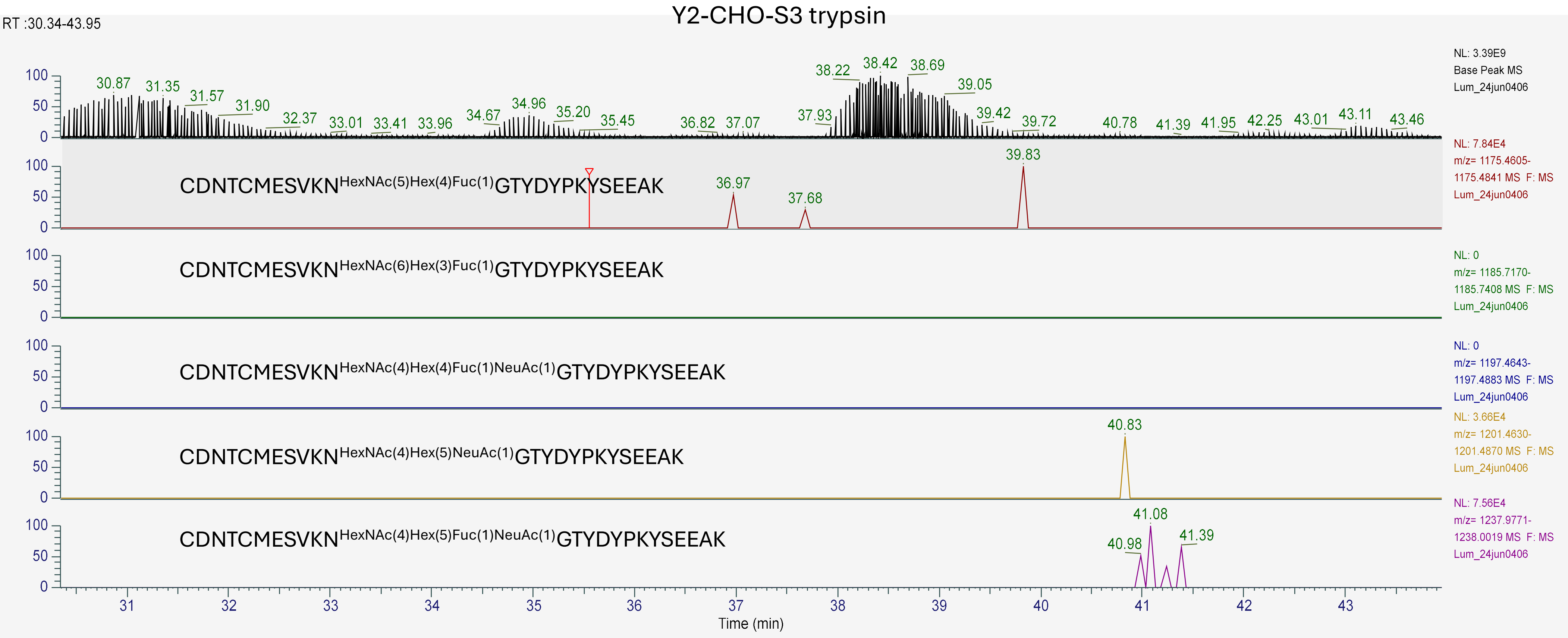

Y2-CHO-S3 trypsin
RT :30.34-43.95
NL: 3.39E9
Base Peak MS
Lum_24jun0406
CDNTCMESVKN HexNAc(5)Hex(4)Fuc(1) GTYDYPKYSEEAK
NL: 7.84E4
m/z= 1175.4605-1175.4841 MS F: MS
Lum_24jun0406
CDNTCMESVKN HexNAc(6)Hex(3)Fuc(1) GTYDYPKYSEEAK
NL: 0
m/z= 1185.7170-1185.7408 MS F: MS
Lum_24jun0406
CDNTCMESVKN HexNAc(4)Hex(4)Fuc(1)NeuAc(1) GTYDYPKYSEEAK
NL: 0
m/z= 1197.4643-1197.4883 MS F: MS
Lum_24jun0406
CDNTCMESVKN HexNAc(4)Hex(5)NeuAc(1) GTYDYPKYSEEAK
NL: 3.66E4
m/z= 1201.4630-1201.4870 MS F: MS
Lum_24jun0406
CDNTCMESVKN HexNAc(4)Hex(5)Fuc(1)NeuAc(1) GTYDYPKYSEEAK
NL: 7.56E4
m/z= 1237.9771-1238.0019 MS F: MS
Lum_24jun0406
Time (min)

Y2-CHO-S3 trypsin

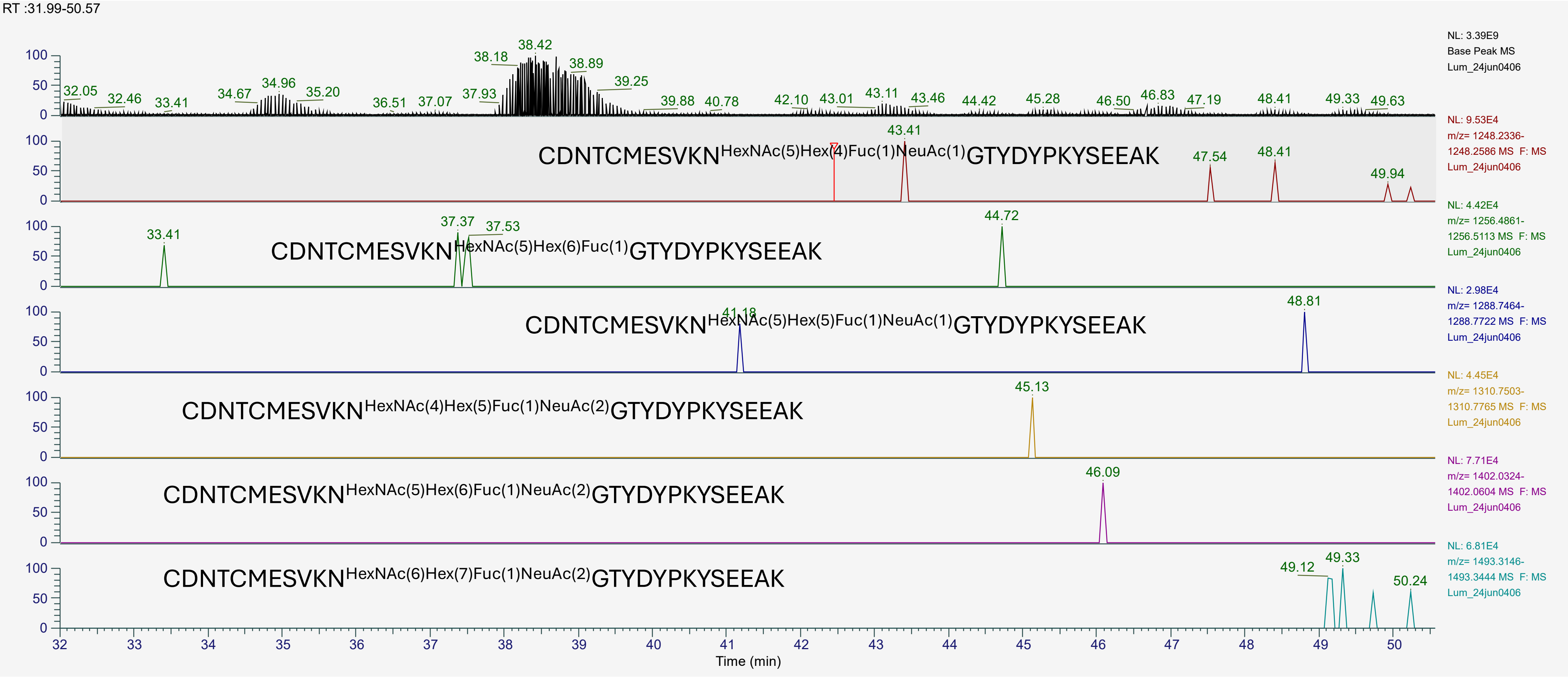

Y2-CHO-S3 trypsin

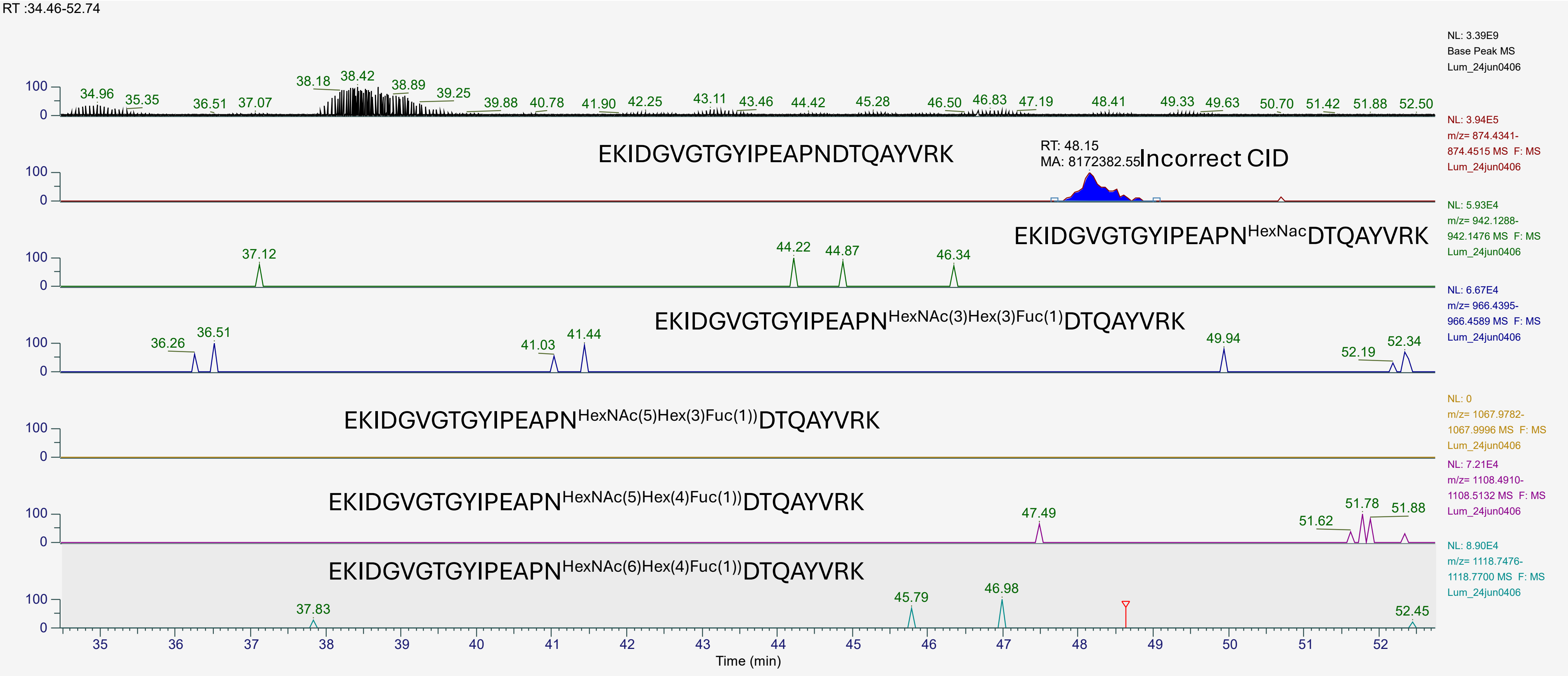

Y2-CHO-S3 trypsin

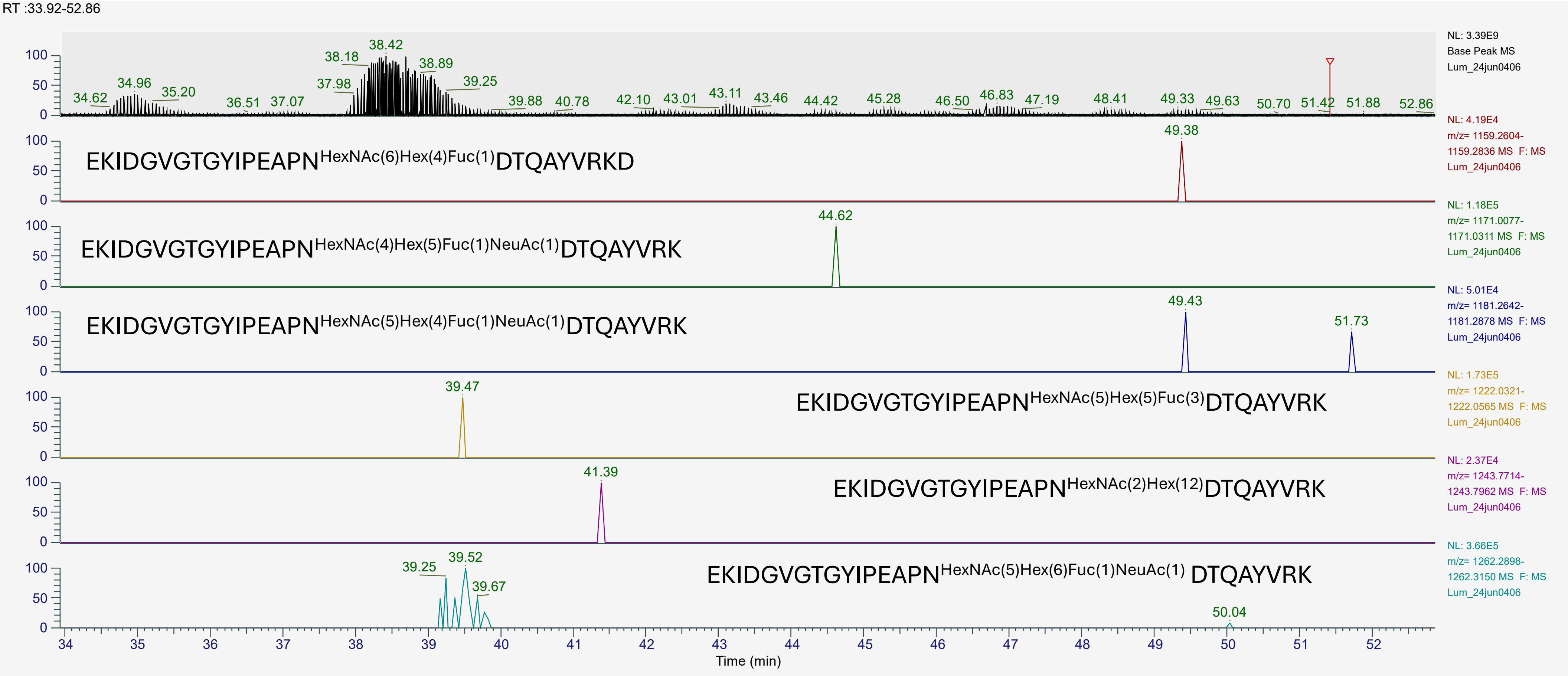

Y2-CHO-S3 trypsin

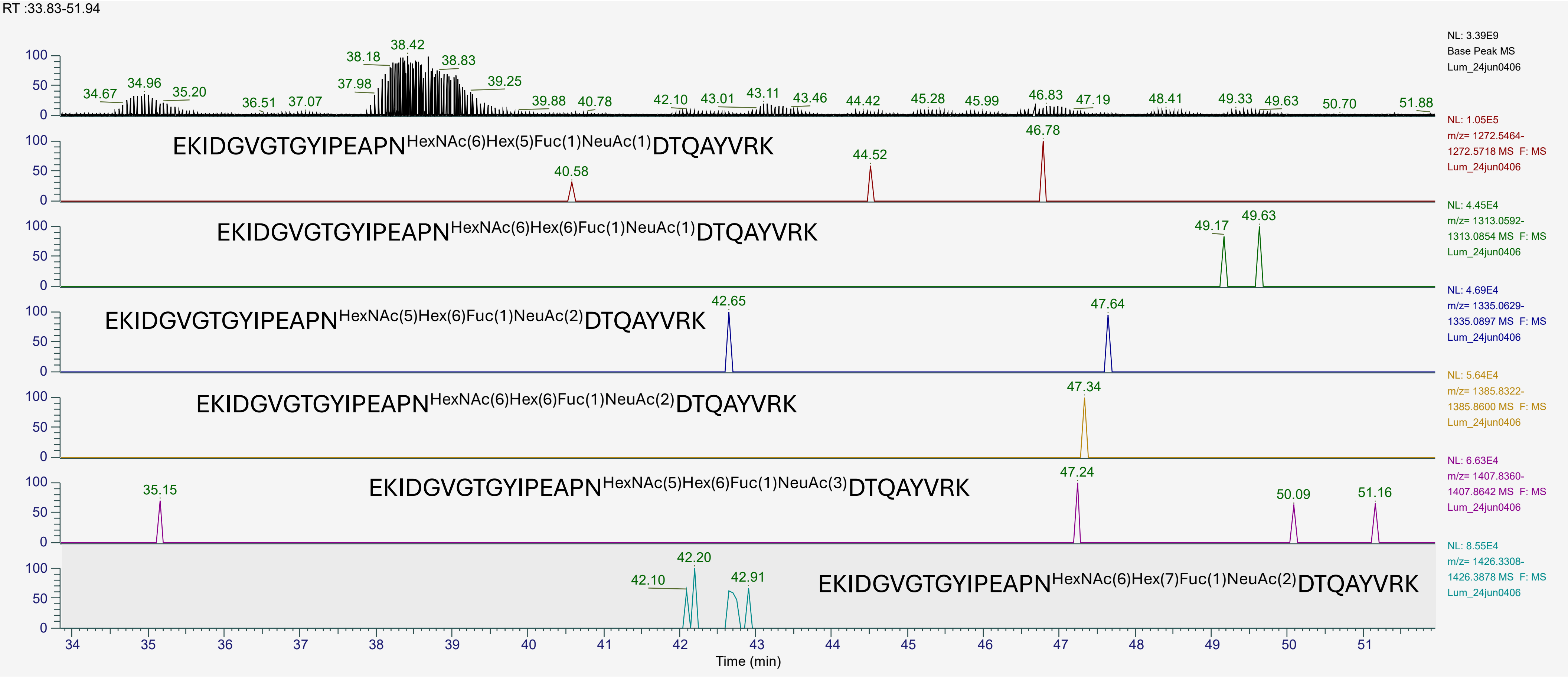

Y2-CHO-S3 trypsin

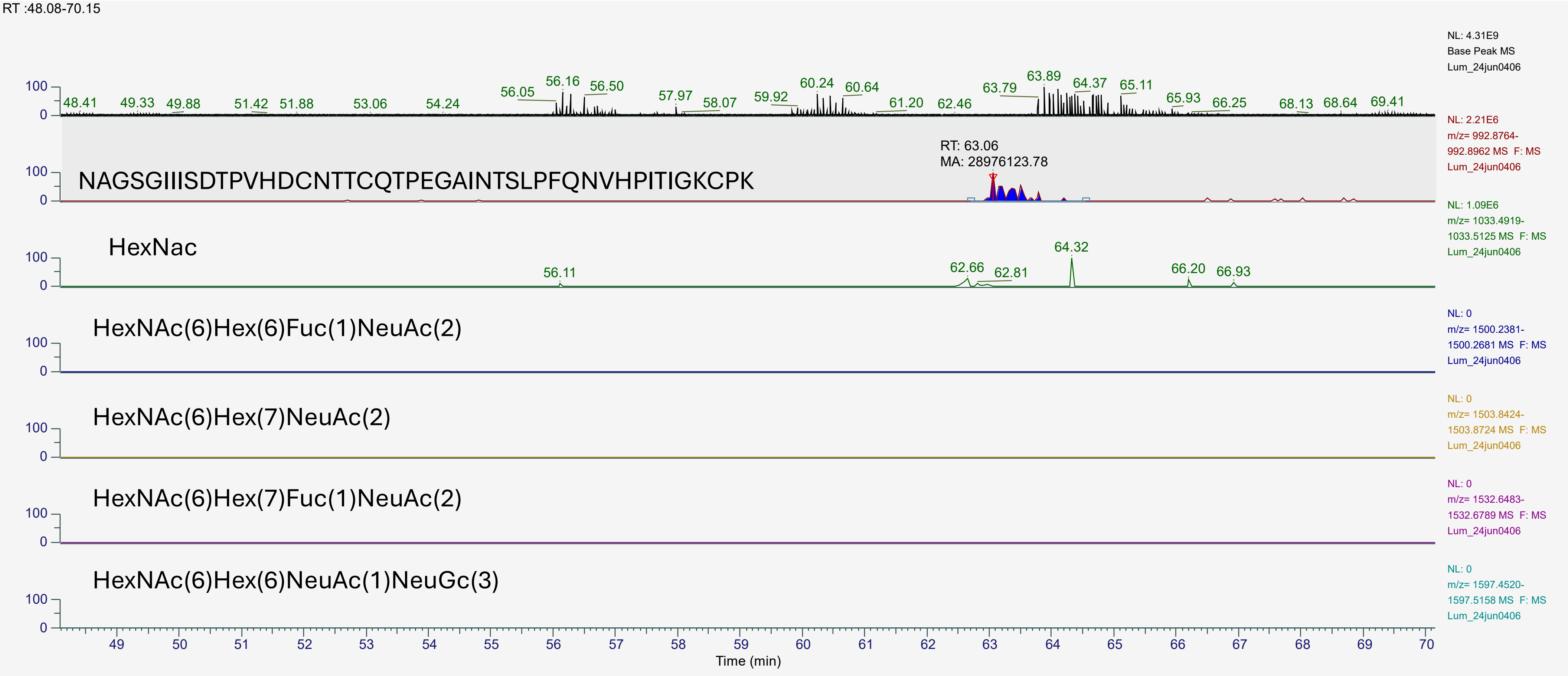

Y2-CHO-S3 trypsin

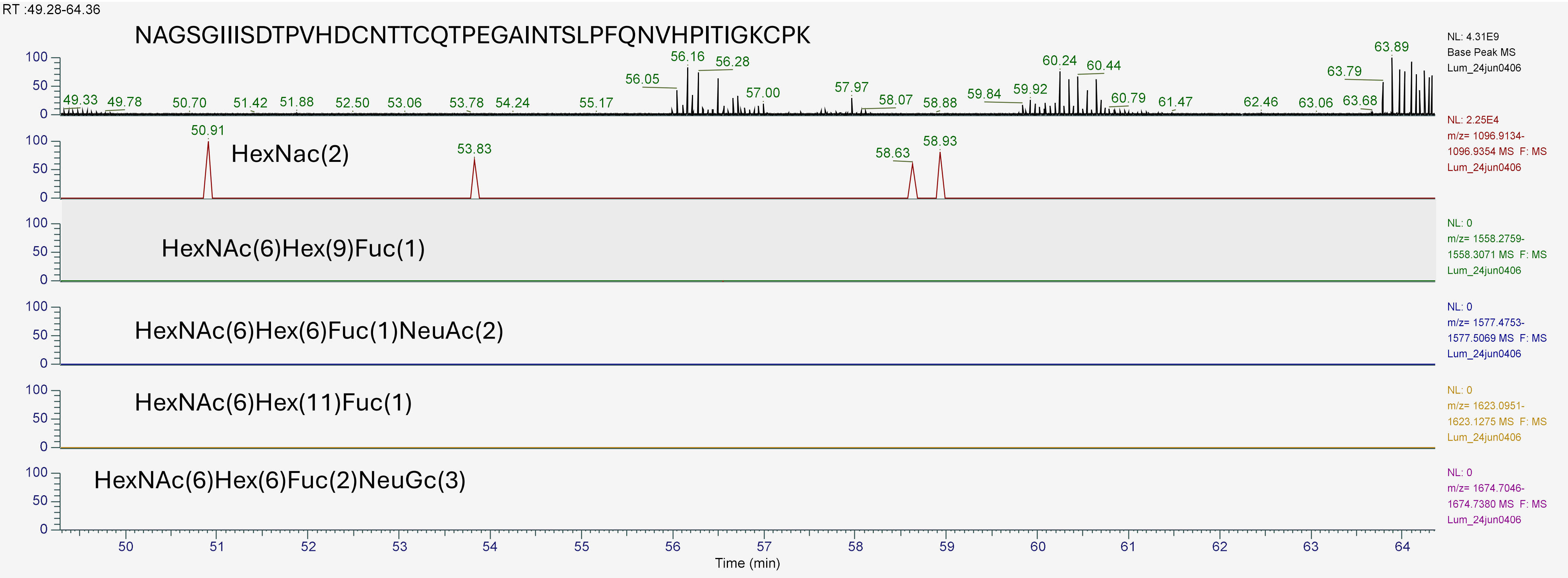

Y2-CHO-S3 trypsin

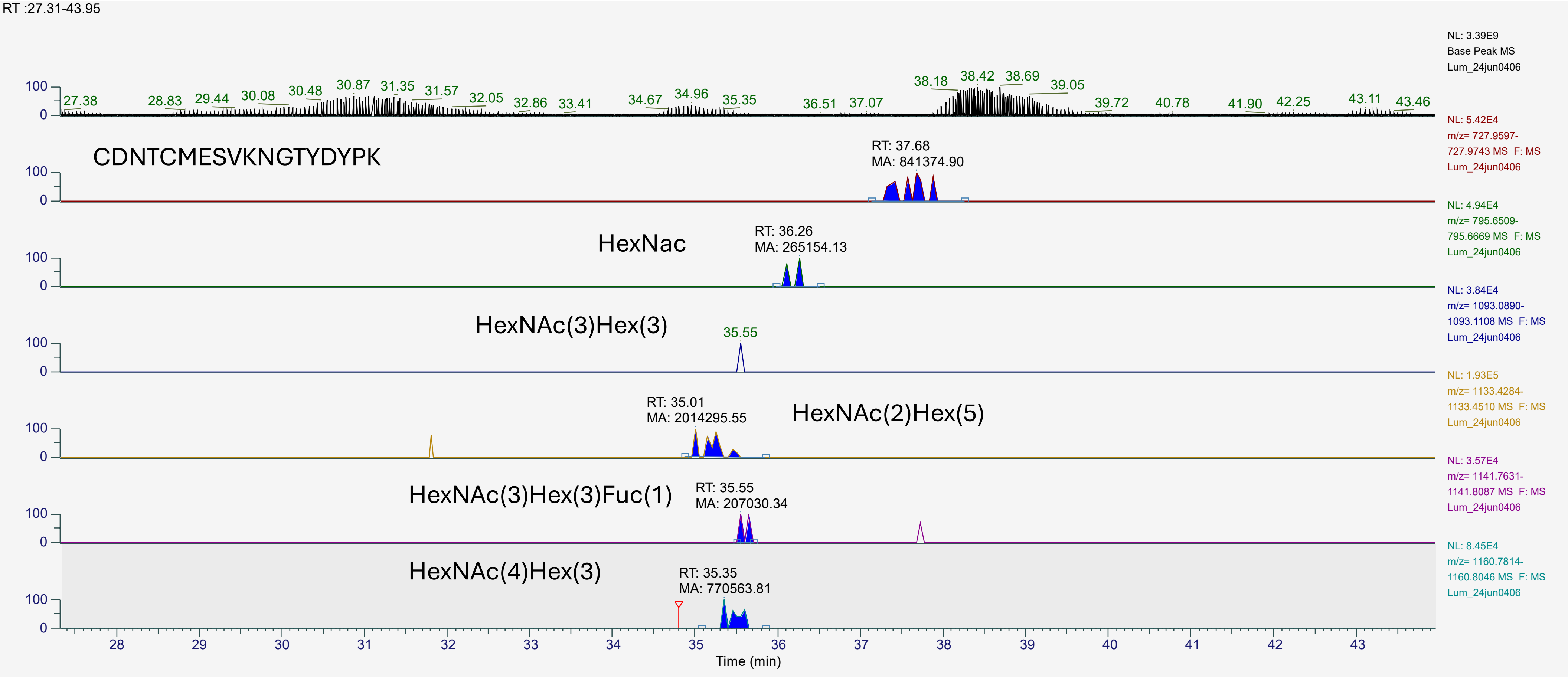

Y2-CHO-S3 trypsin

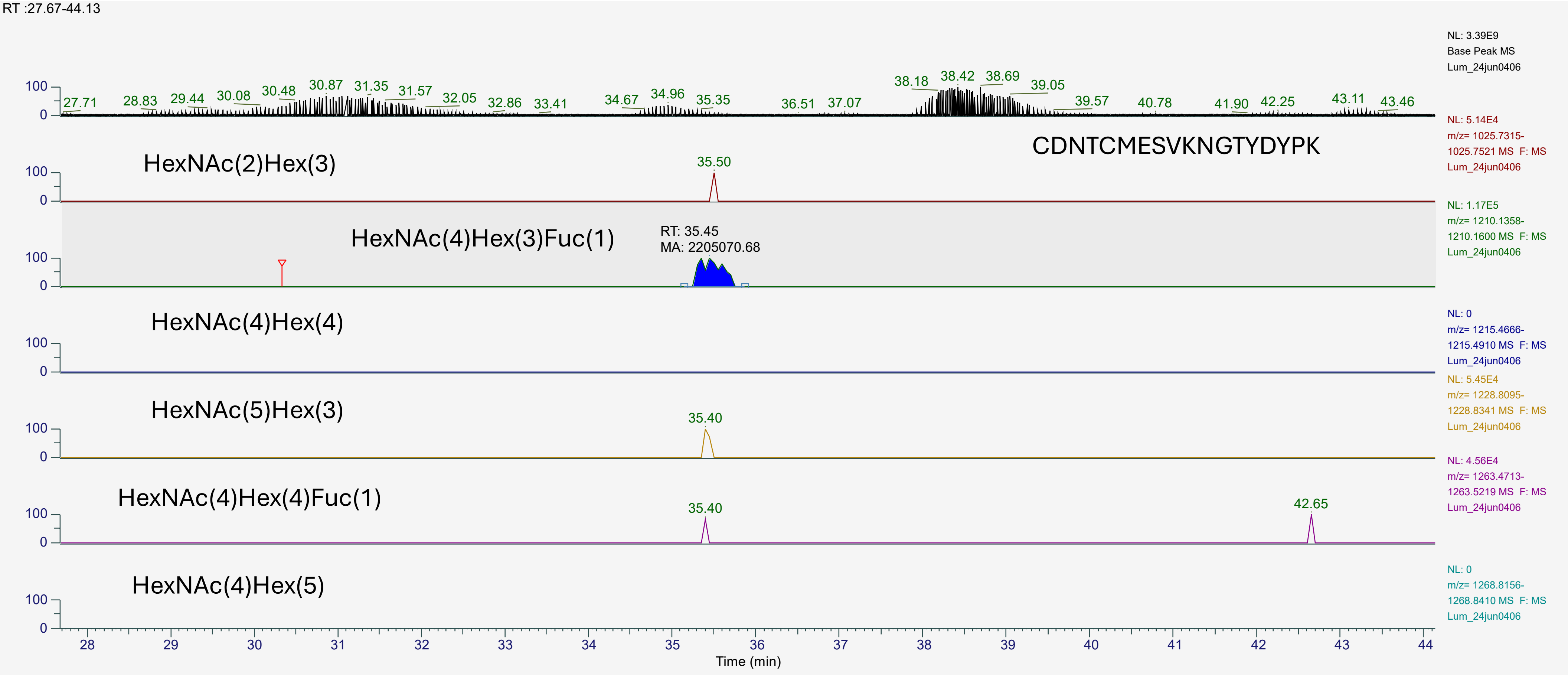

Y2-CHO-S3 trypsin

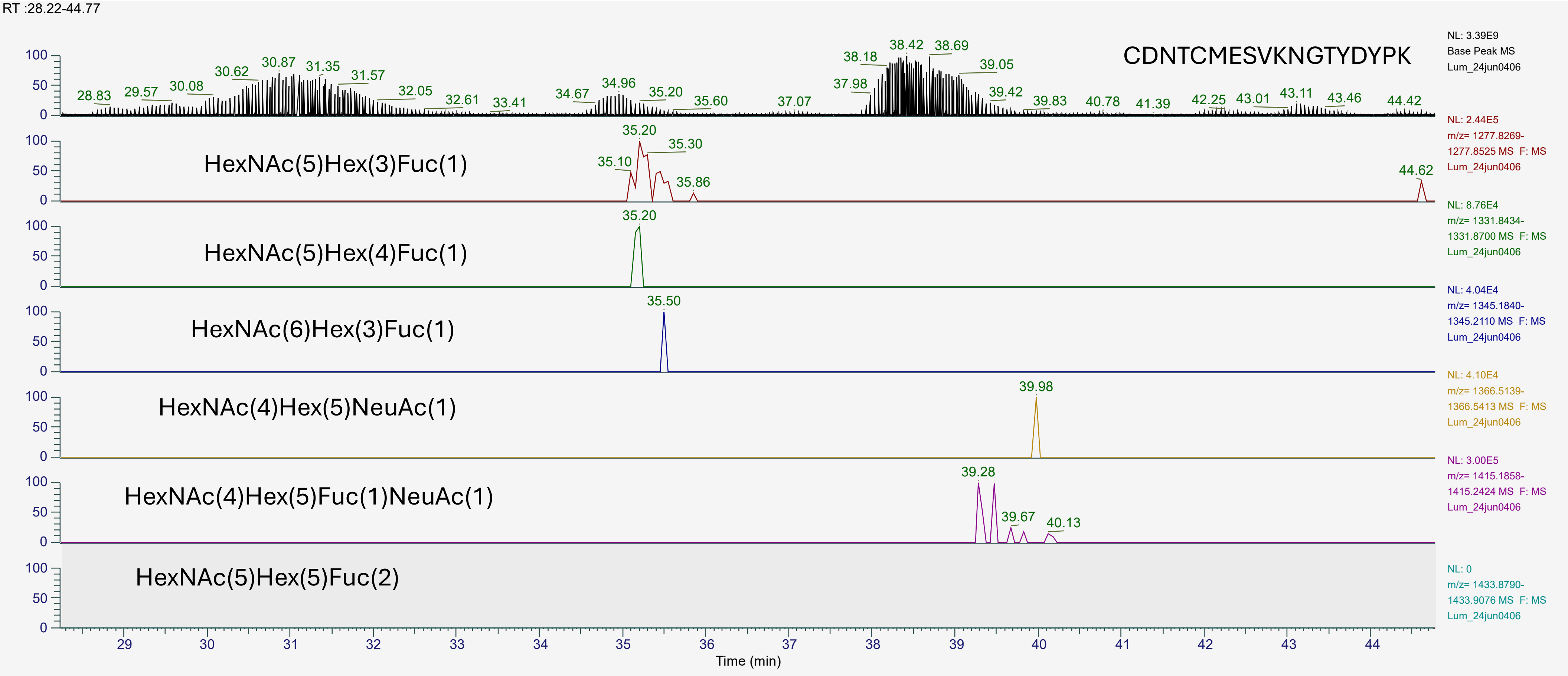

Y2-CHO-S3 trypsin

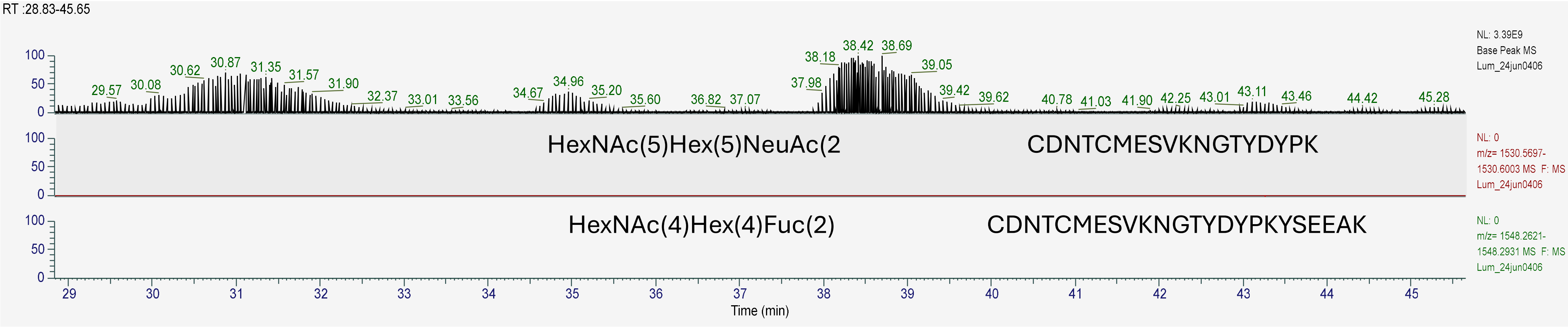

Y2-CHO-S3 trypsin

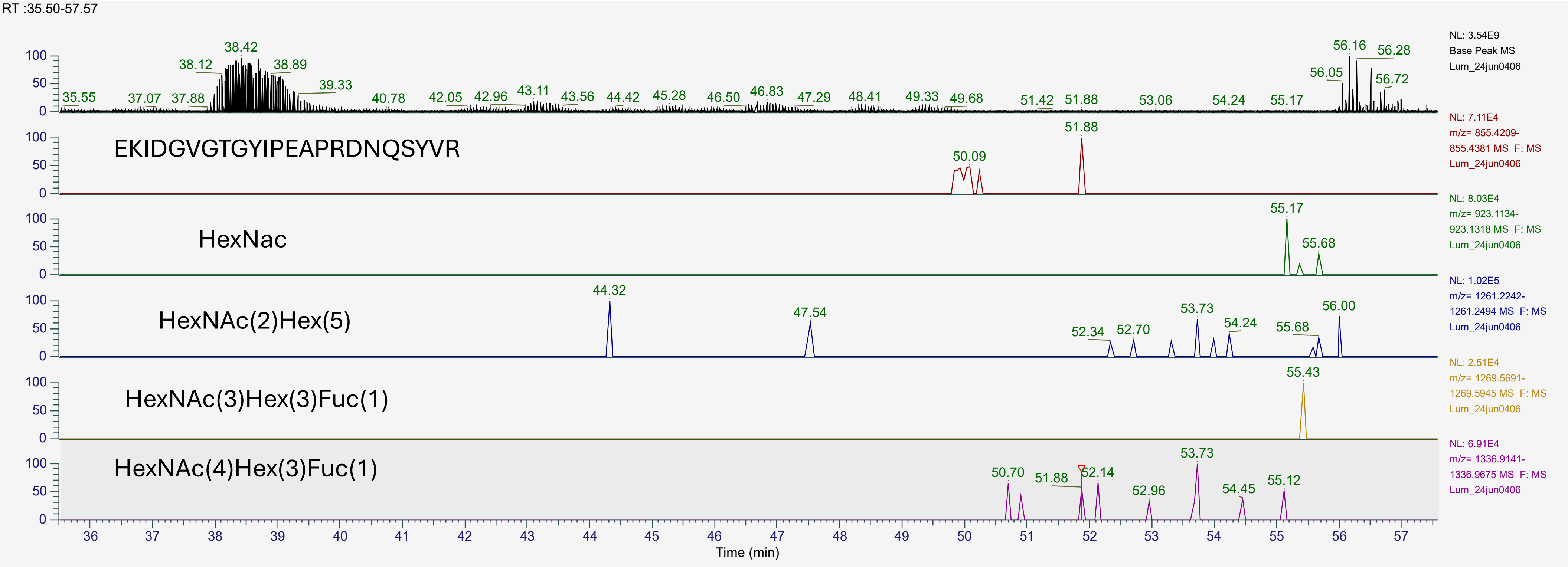

Y2-CHO-WT trypsin

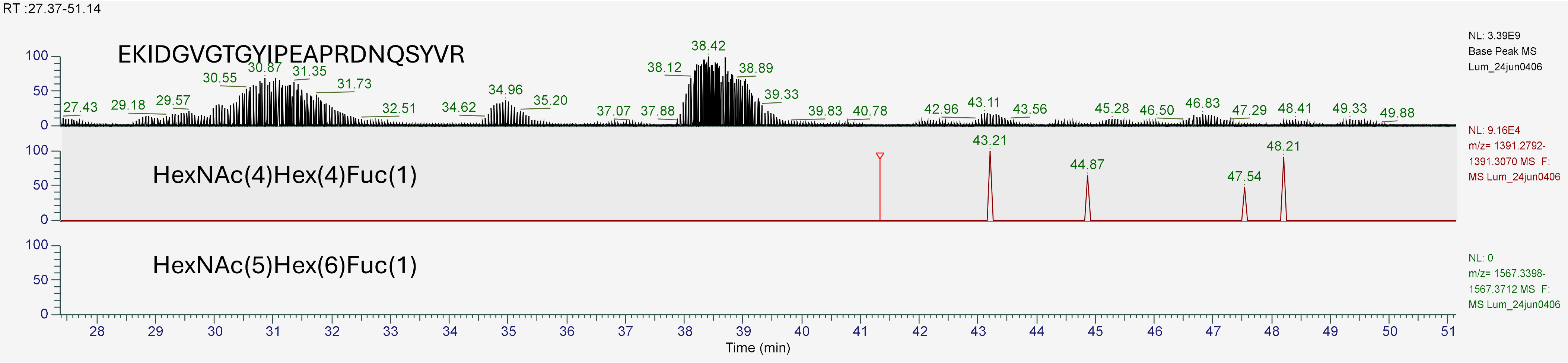

Y2-CHO-S3 trypsin

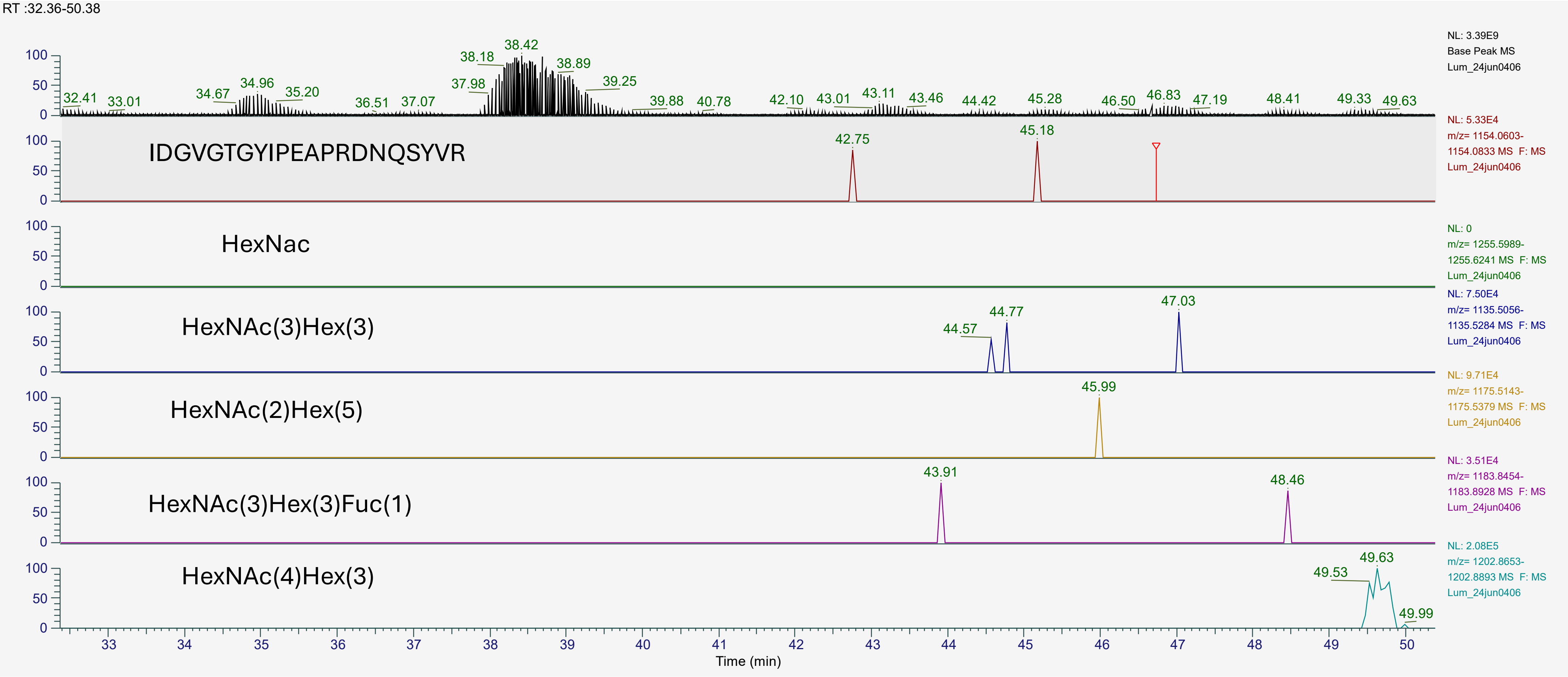

Y2-CHO-S3 trypsin

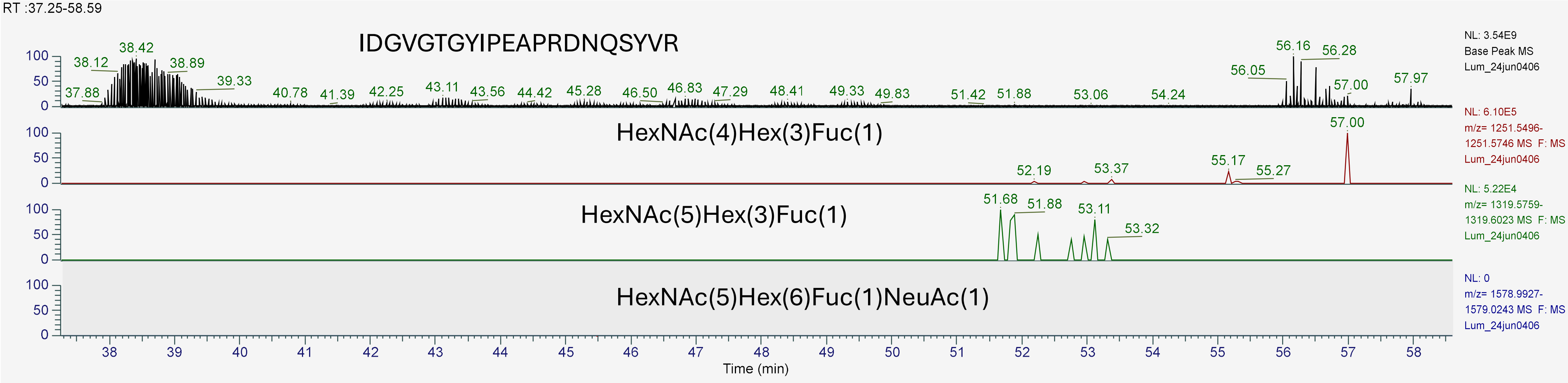

Y2-CHO-S3 trypsin

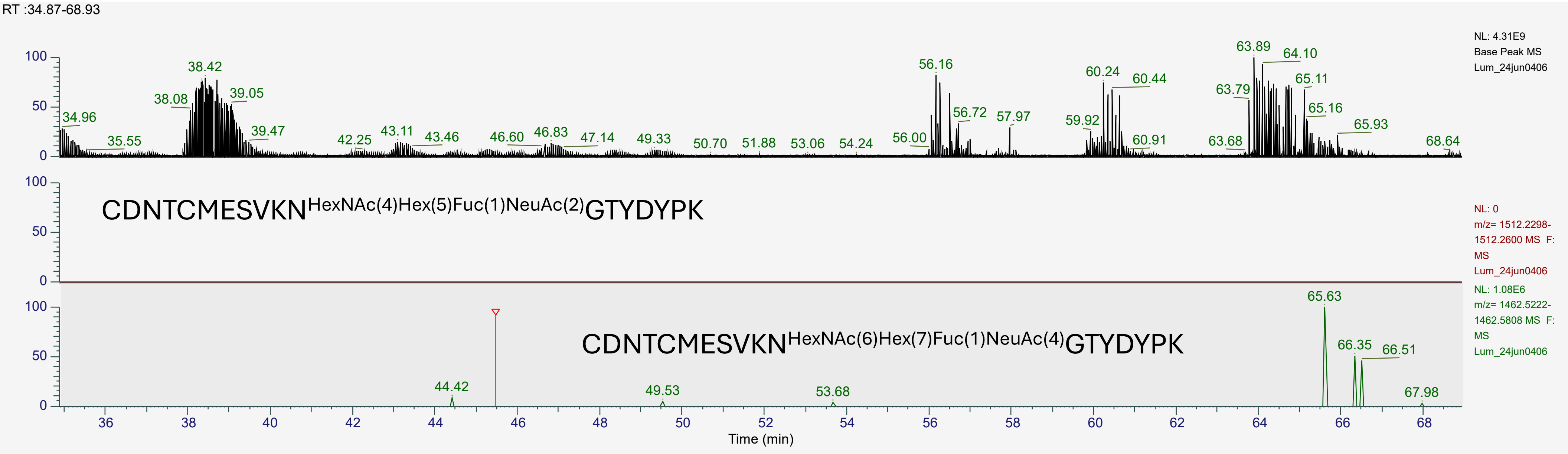

Y2-CHO-S3 GluC

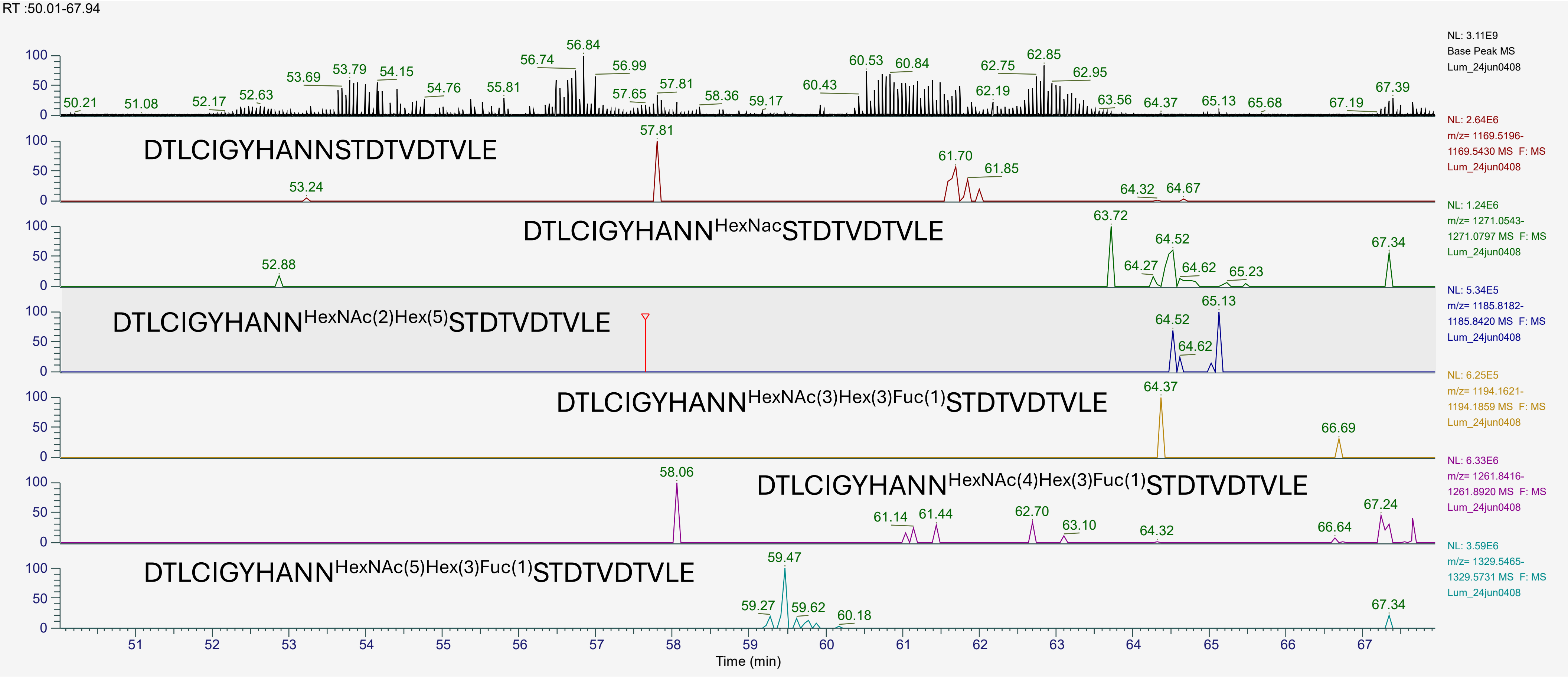

Y2-CHO-S3 GluC

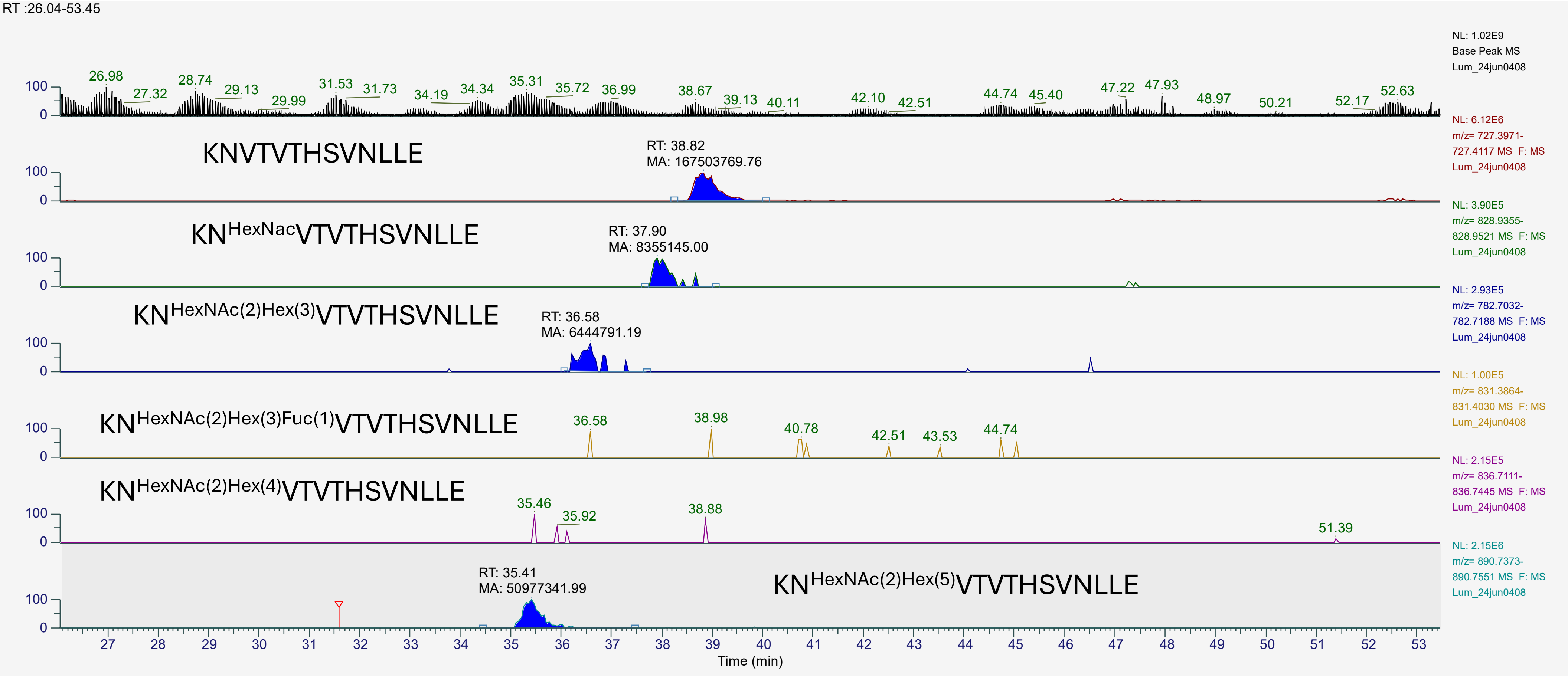

Y2-CHO-S3 GluC

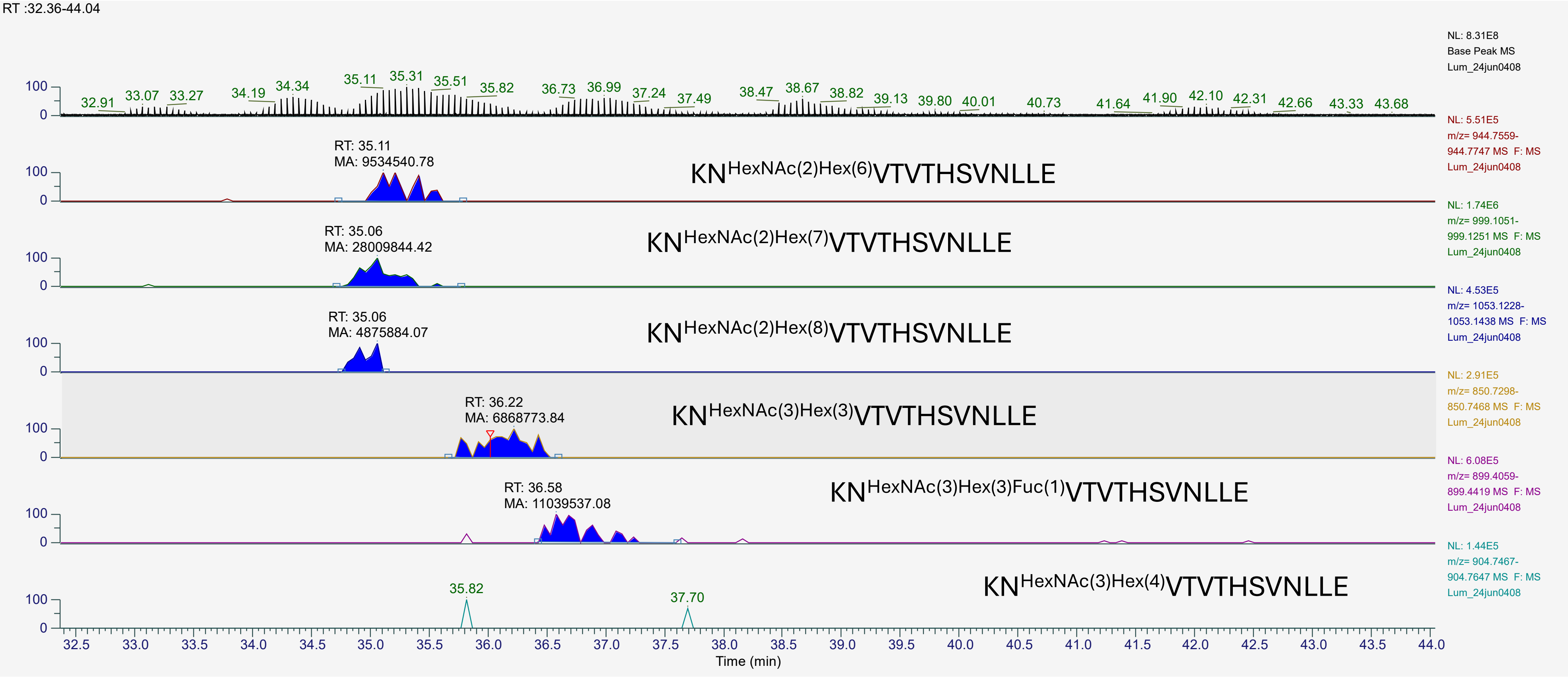

Y2-CHO-S3 GluC

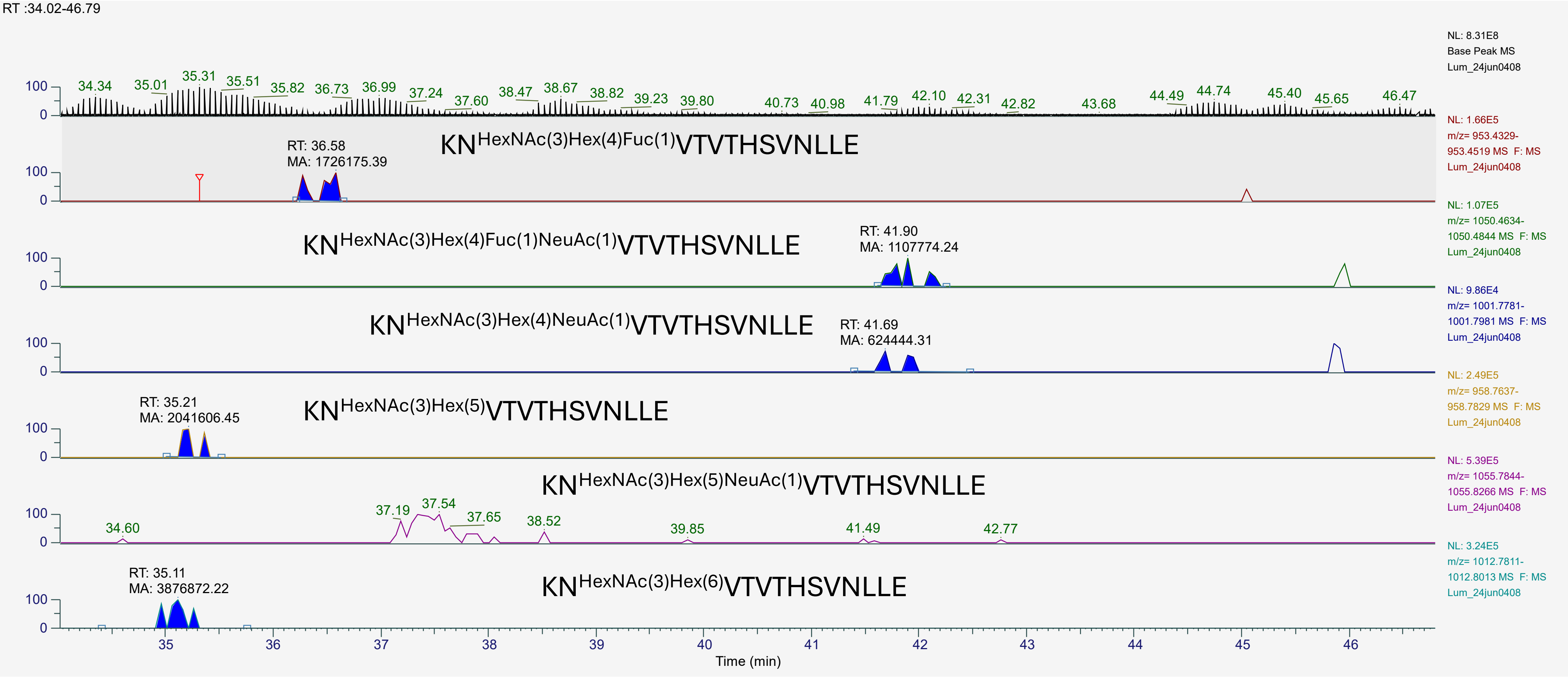

Y2-CHO-S3 GluC

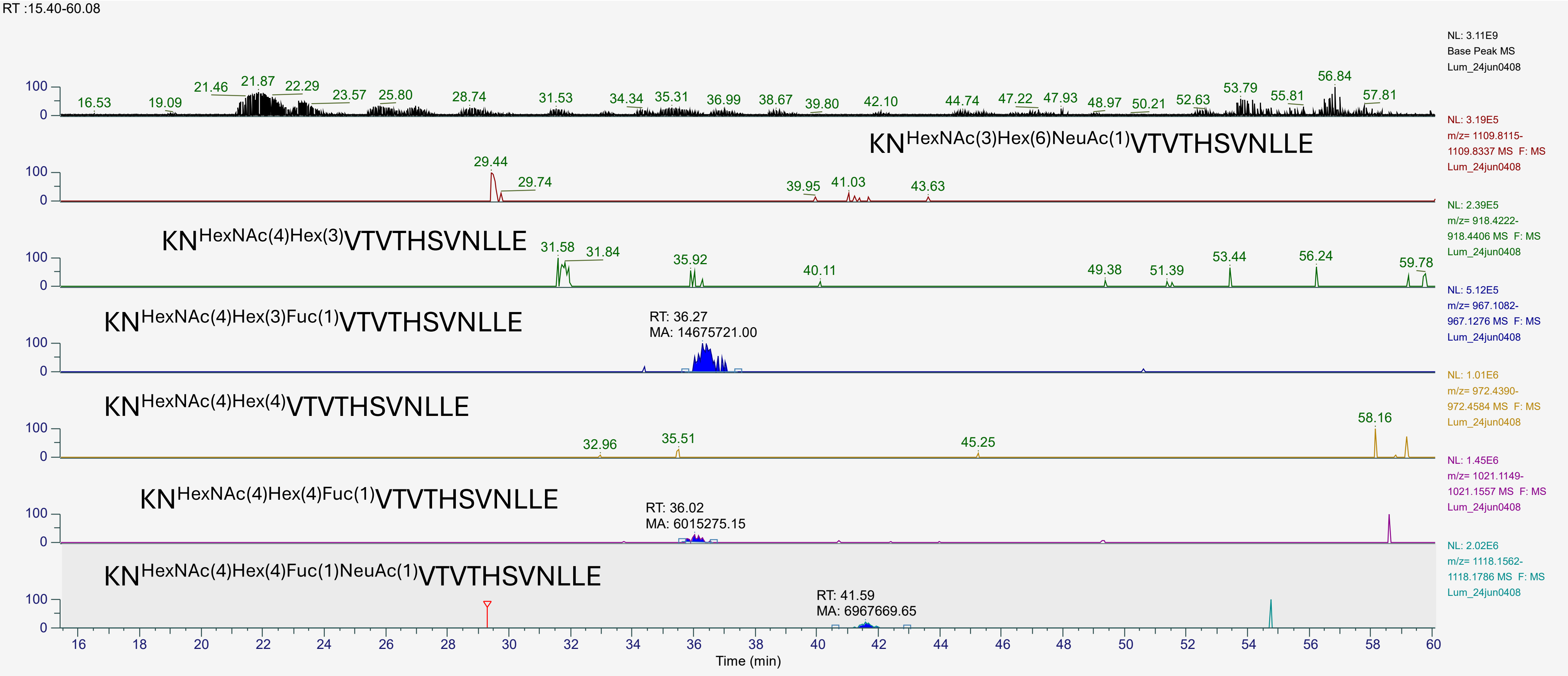

Y2-CHO-S3 GluC

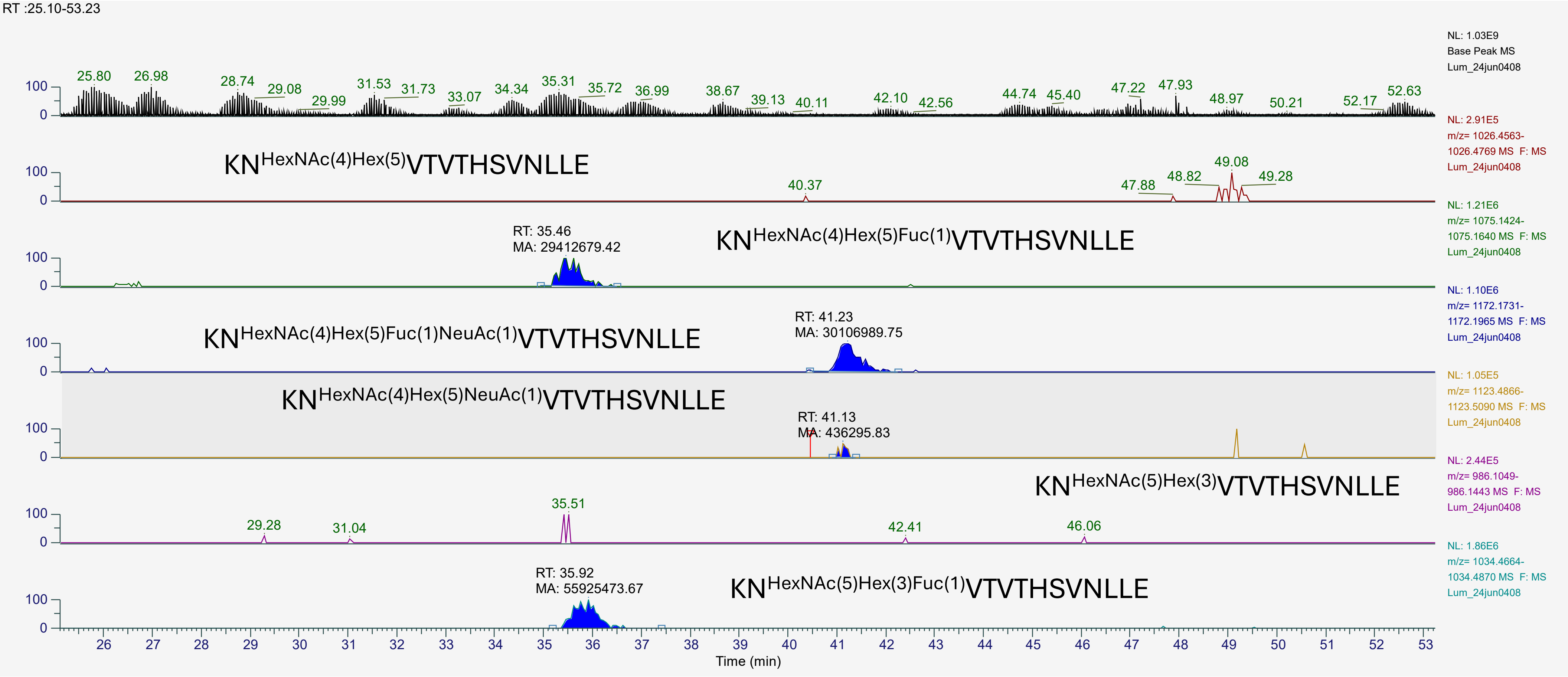

Y2-CHO-S3 GluC

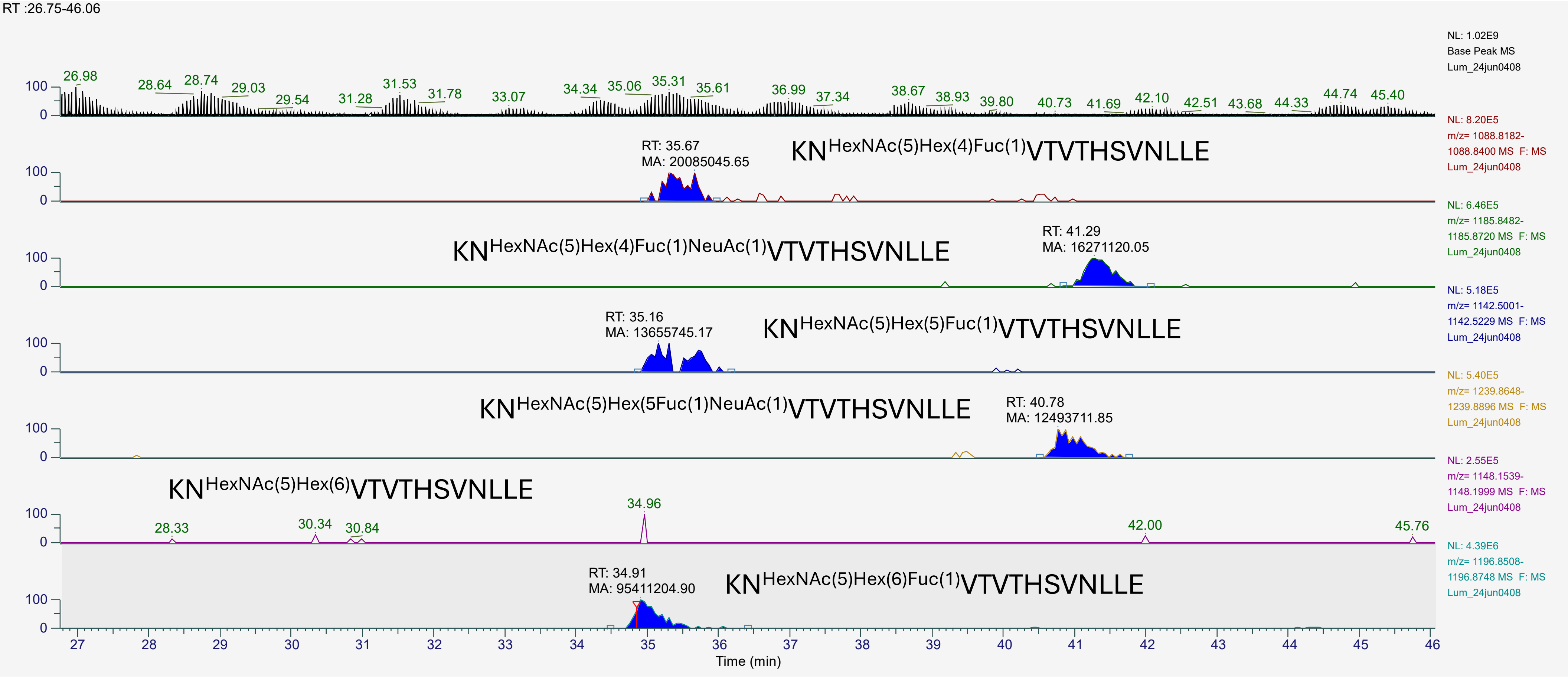

Y2-CHO-S3 GluC

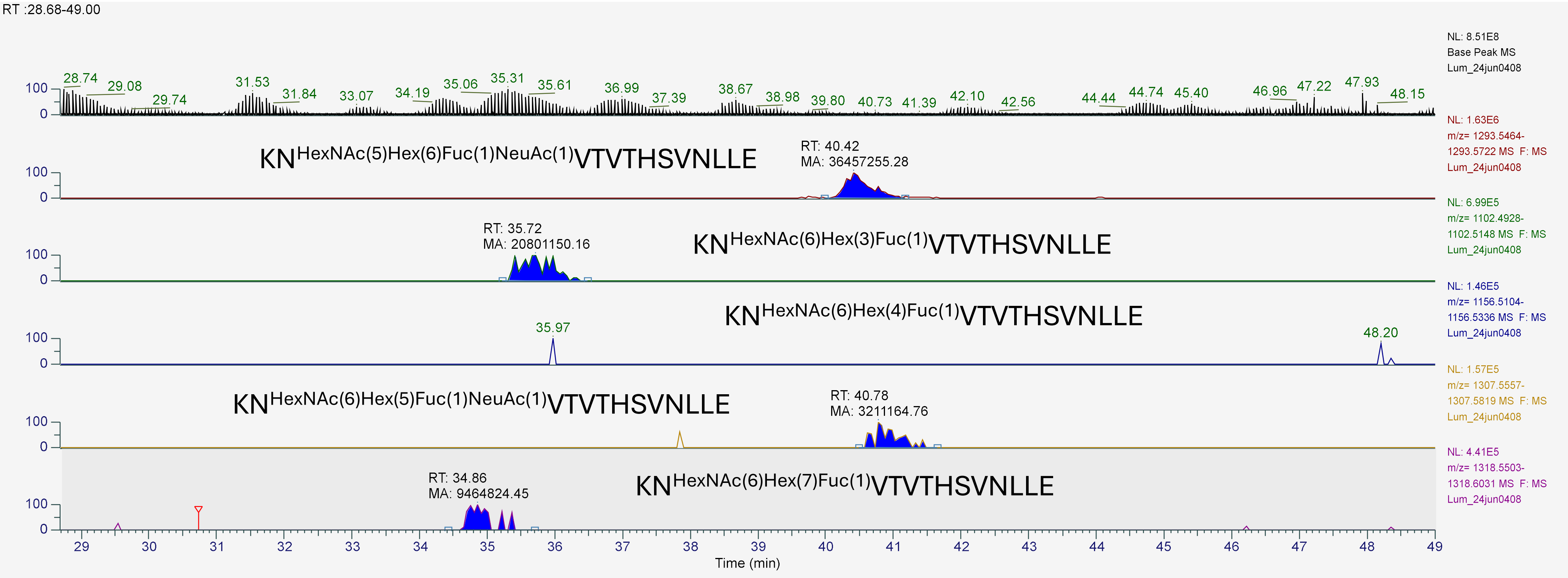

Y2-CHO-S3 GluC

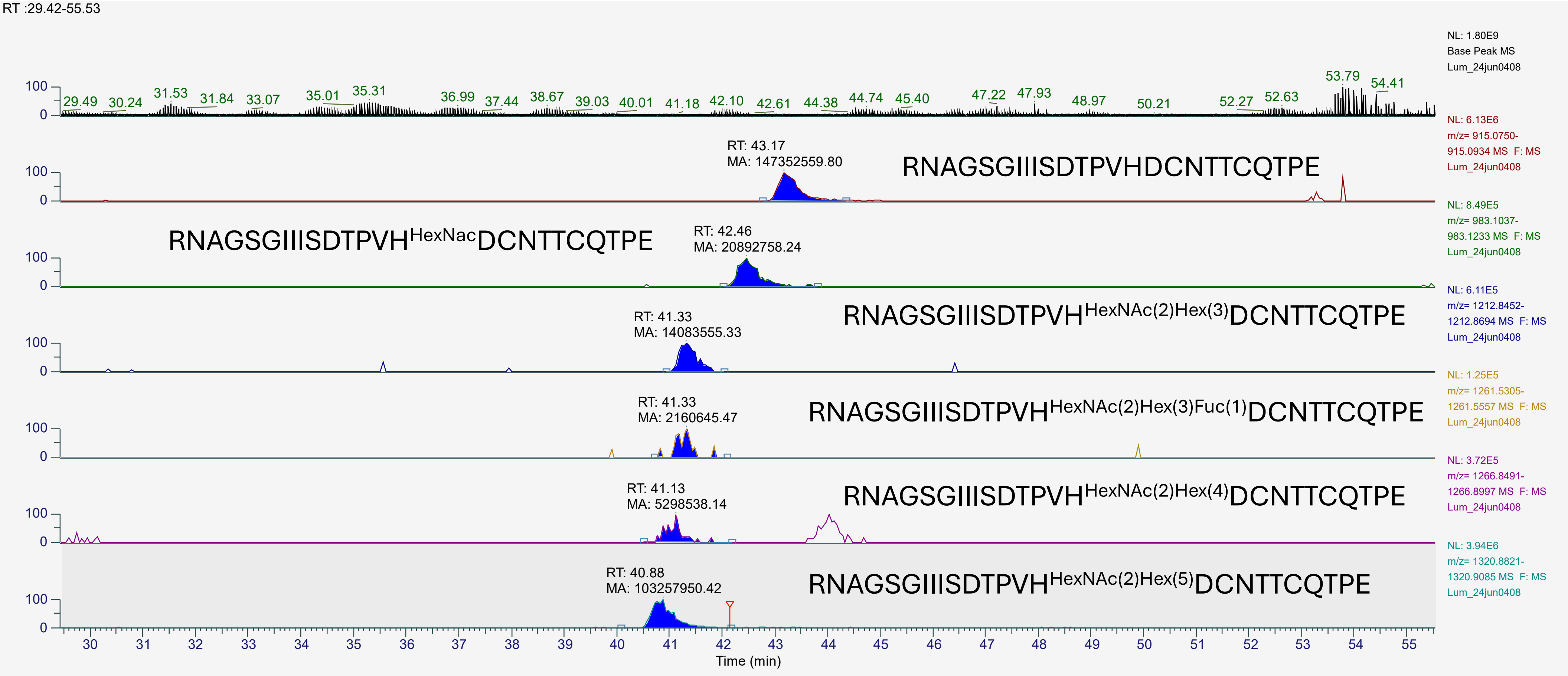

Y2-CHO-S3 GluC

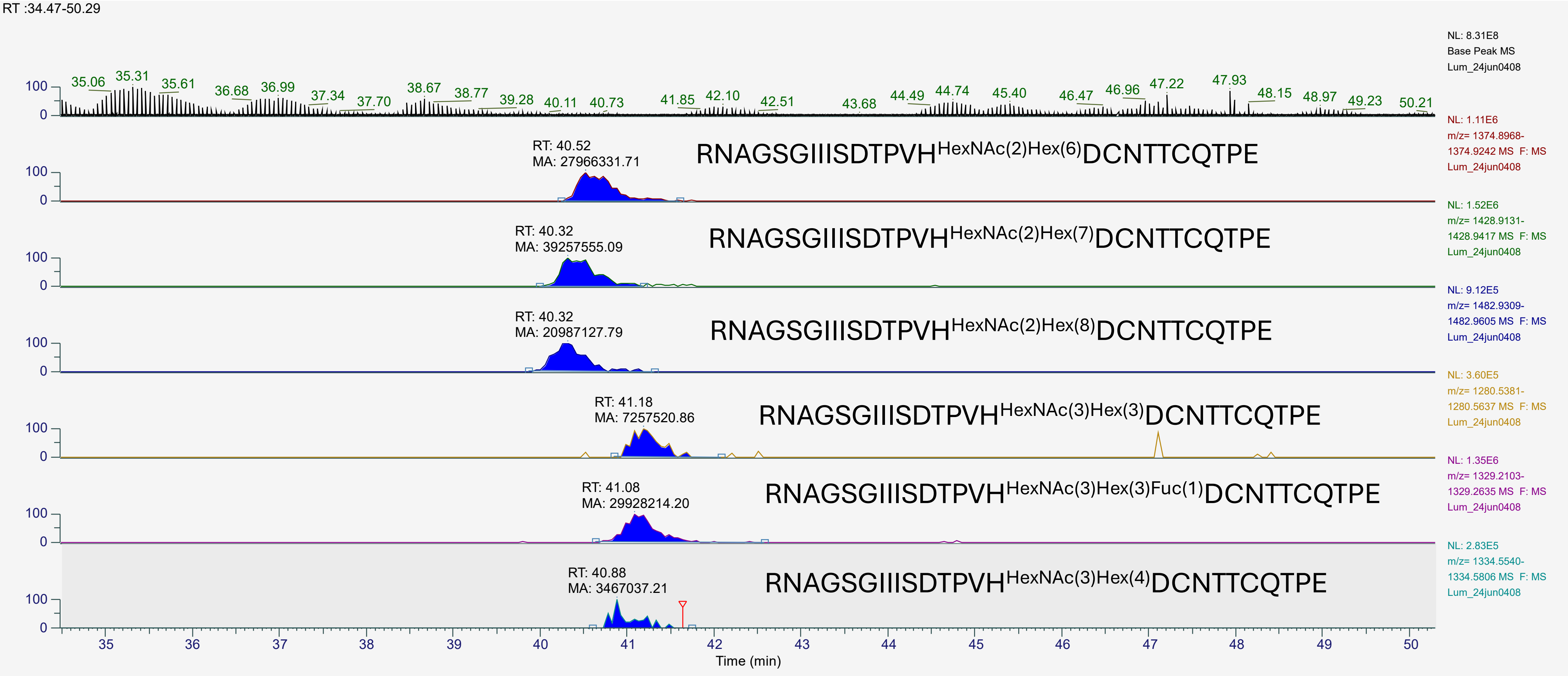

Y2-CHO-S3 GluC

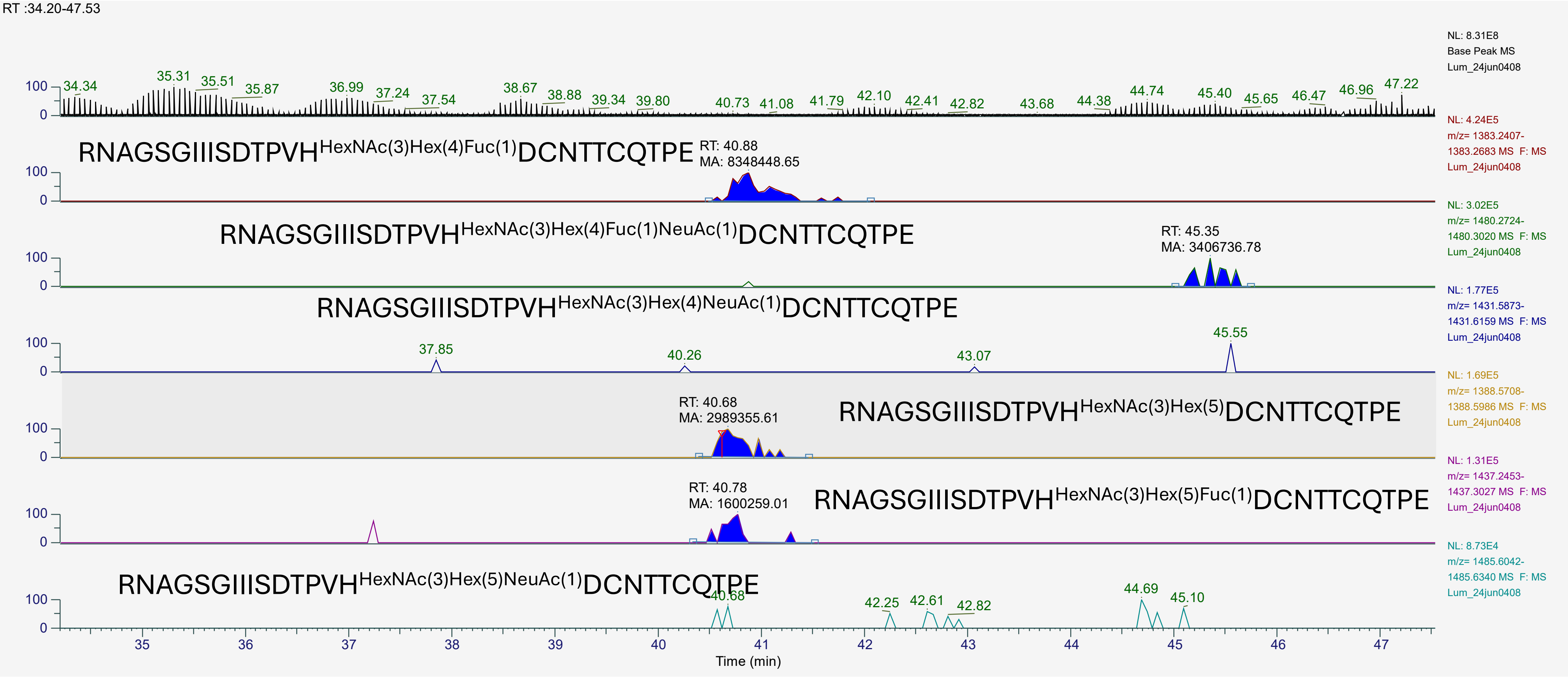

Y2-CHO-S3 GluC

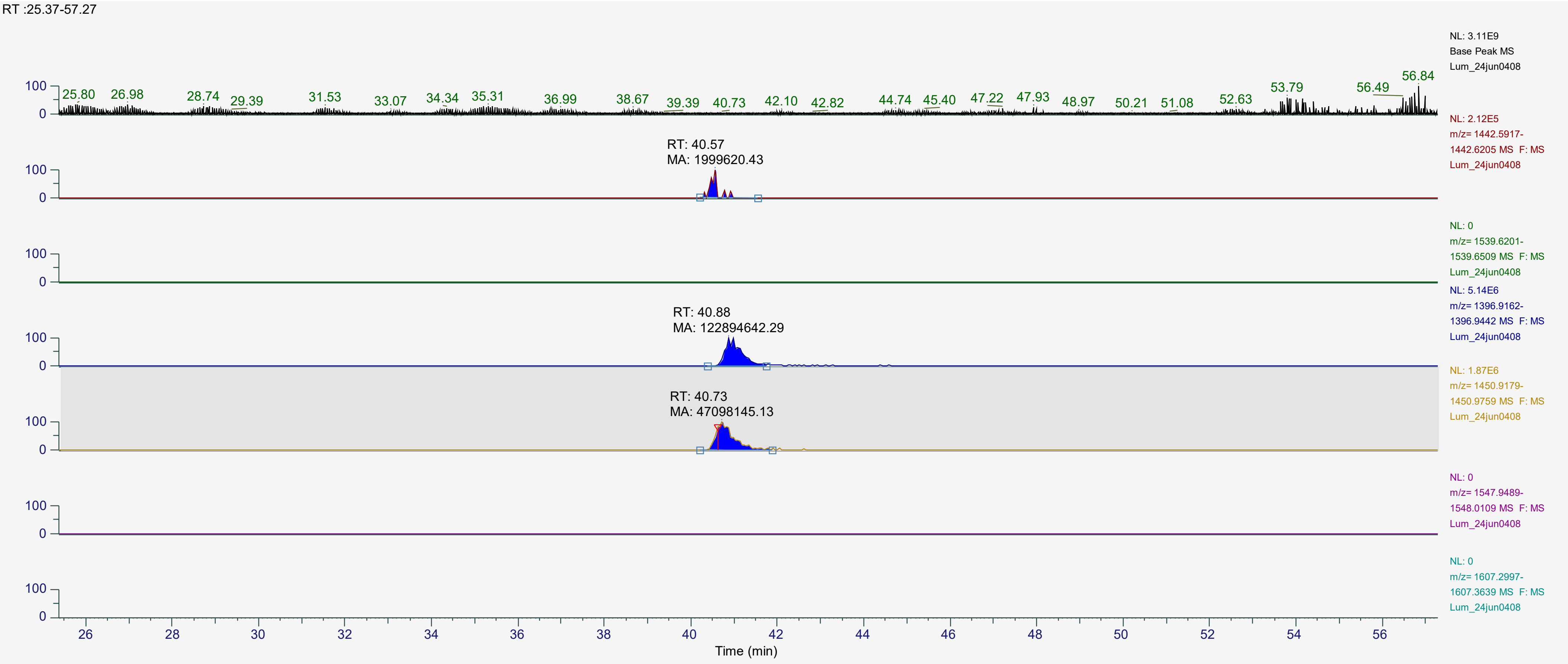

Y2-CHO-S3 GluC

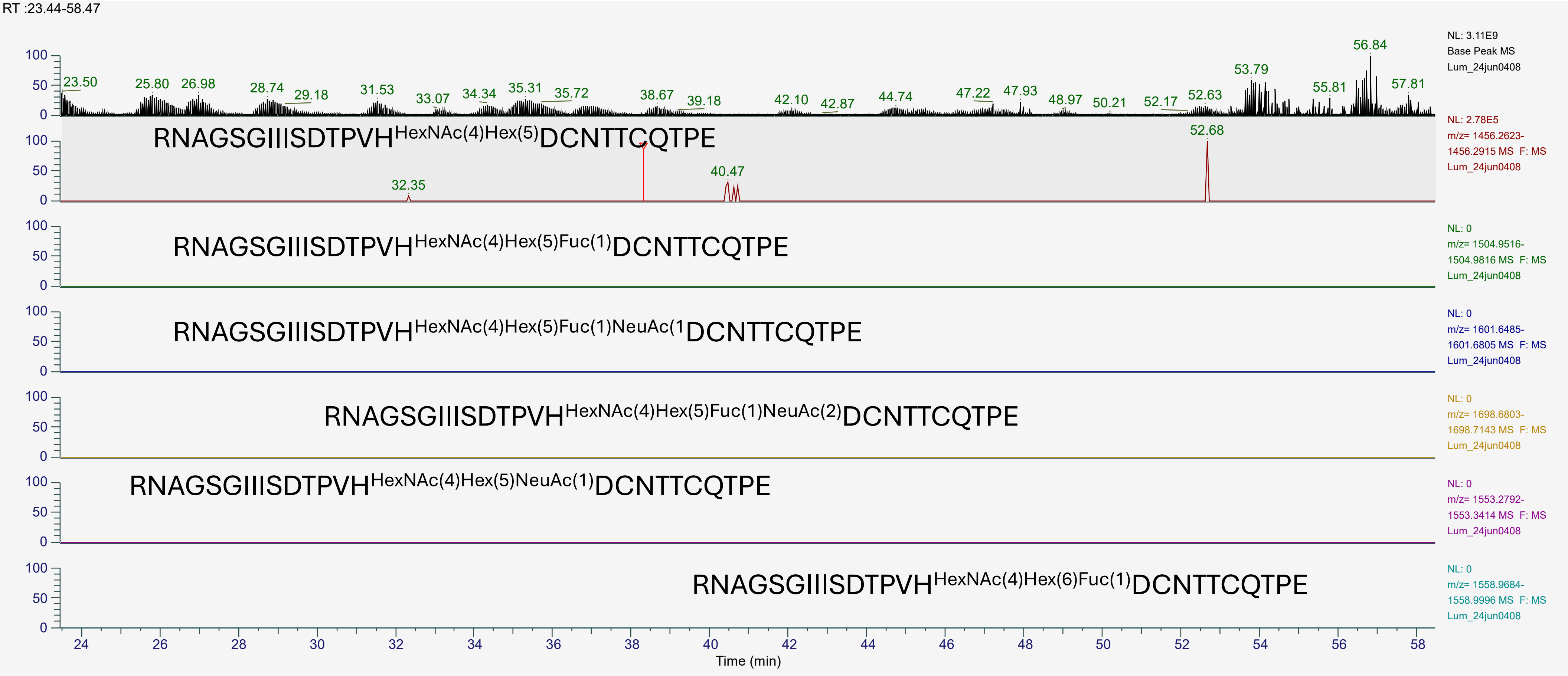

Y2-CHO-S3 GluC

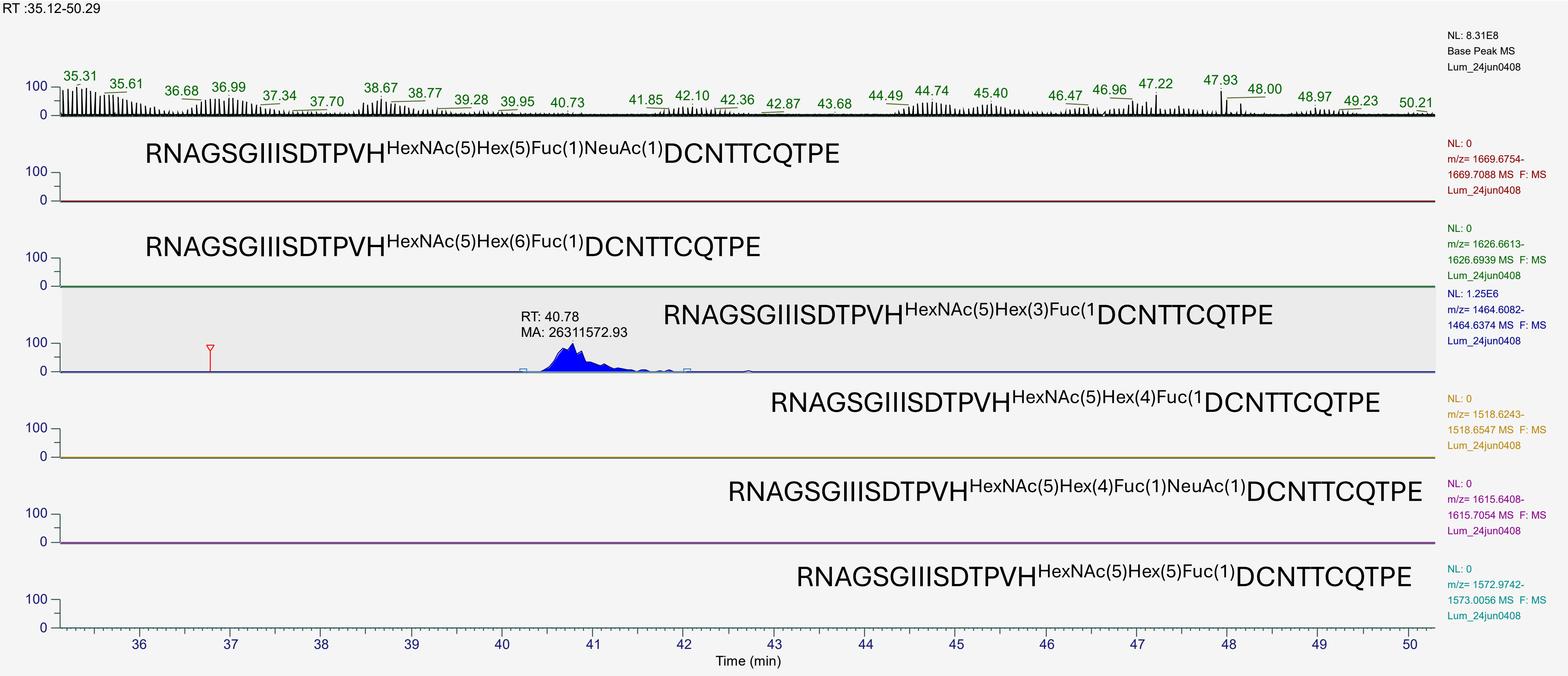

Y2-CHO-S3 GluC

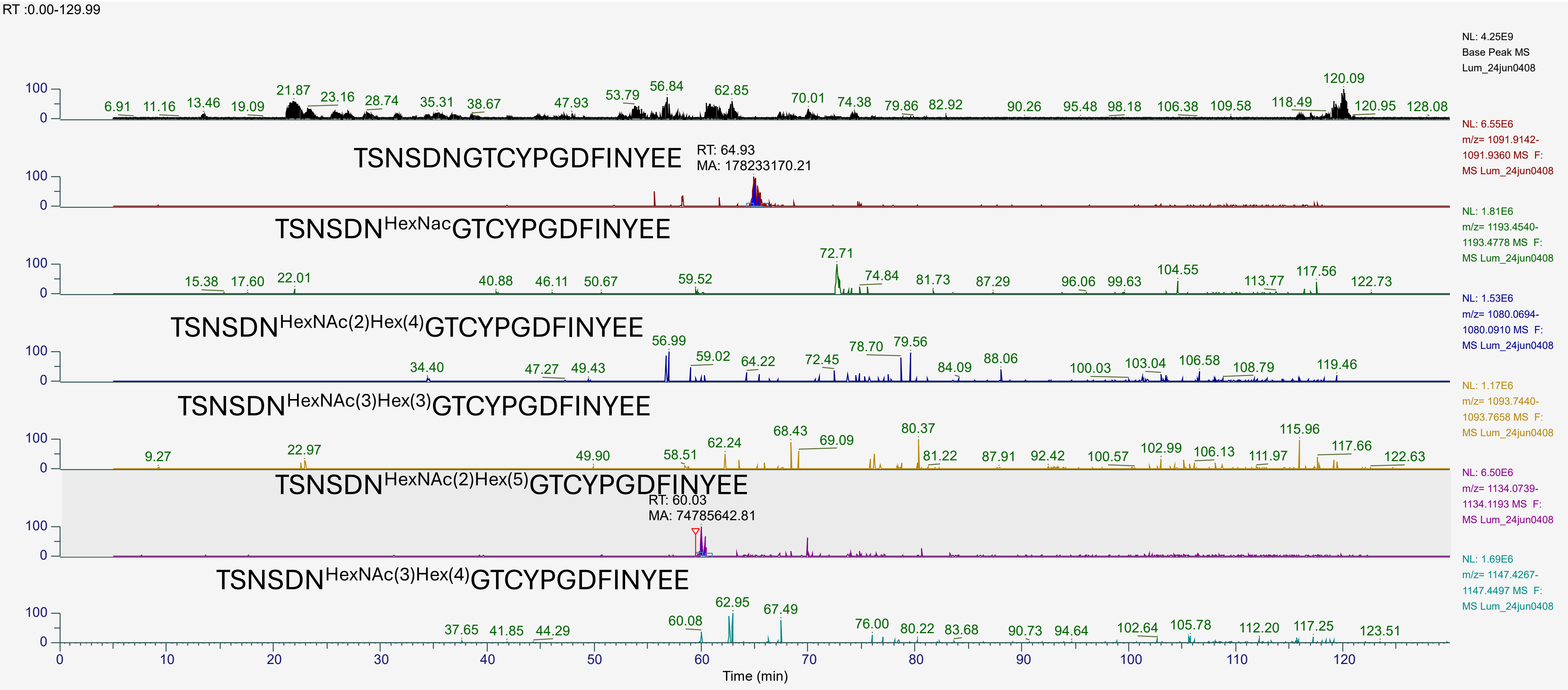

Y2-CHO-S3 S3

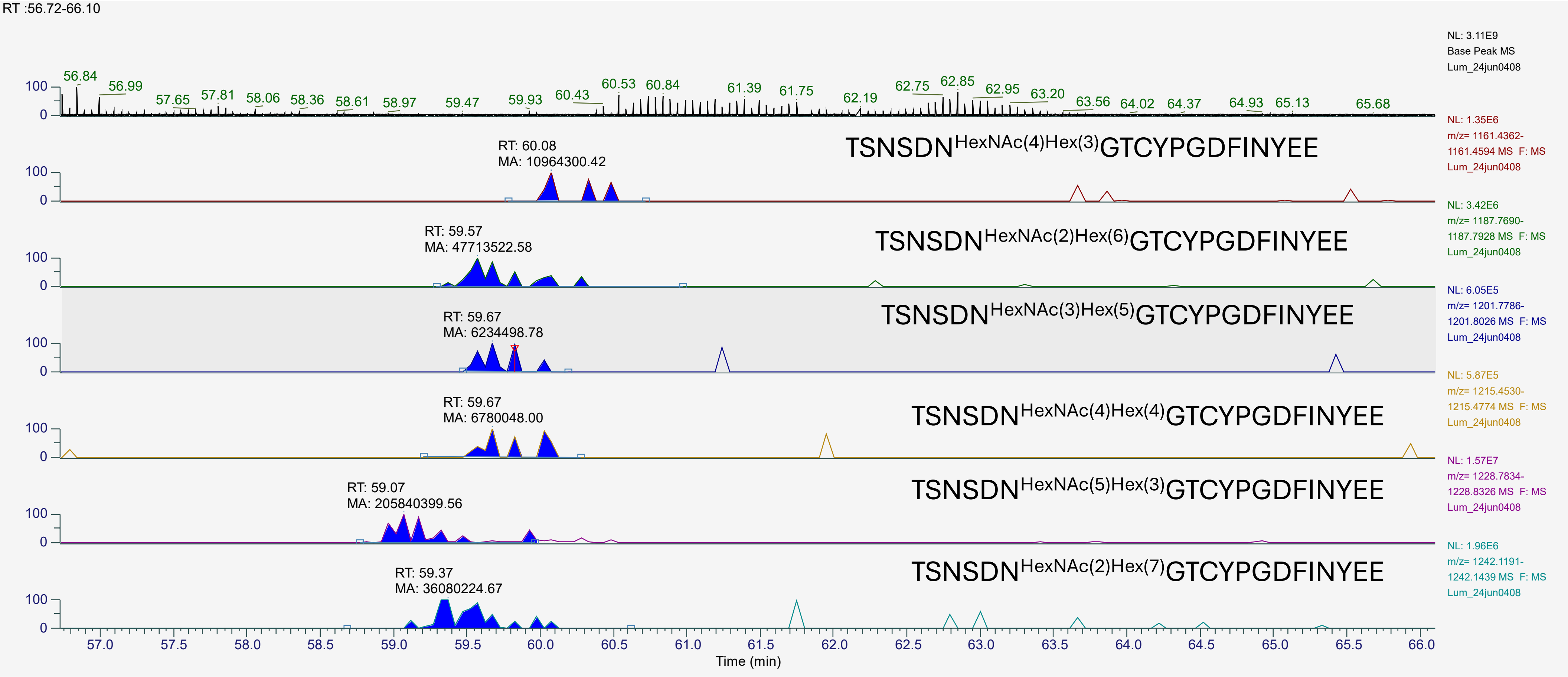
RT :56.72-66.10
NL: 3.11E9
Base Peak MS
Lum_24jun0408
56.84
56.99
57.65
57.81
58.06
58.36
58.61
58.97
59.47
59.93
60.43
60.53
60.84
61.39
61.75
62.19
62.75
62.85
62.95
63.20
63.56
64.02
64.37
64.93
65.13
65.68
RT: 60.08
MA: 10964300.42
TSNSDN HexNAc(4)Hex(3) GTCYPGDFINYEE
NL: 1.35E6
m/z= 1161.4362-1161.4594 MS F: MS Lum_24jun0408
RT: 59.57
MA: 47713522.58
TSNSDN HexNAc(2)Hex(6) GTCYPGDFINYEE
NL: 3.42E6
m/z= 1187.7690-1187.7928 MS F: MS Lum_24jun0408
RT: 59.67
MA: 6234498.78
TSNSDN HexNAc(3)Hex(5) GTCYPGDFINYEE
NL: 6.05E5
m/z= 1201.7786-1201.8026 MS F: MS Lum_24jun0408
RT: 59.67
MA: 6780048.00
TSNSDN HexNAc(4)Hex(4) GTCYPGDFINYEE
NL: 5.87E5
m/z= 1215.4530-1215.4774 MS F: MS Lum_24jun0408
RT: 59.07
MA: 205840399.56
TSNSDN HexNAc(5)Hex(3) GTCYPGDFINYEE
NL: 1.57E7
m/z= 1228.7834-1228.8326 MS F: MS Lum_24jun0408
RT: 59.37
MA: 36080224.67
TSNSDN HexNAc(2)Hex(7) GTCYPGDFINYEE
NL: 1.96E6
m/z= 1242.1191-1242.1439 MS F: MS Lum_24jun0408
100
0
57.0
57.5
58.0
58.5
59.0
59.5
60.0
60.5
61.0
61.5
62.0
62.5
63.0
63.5
64.0
64.5
65.0
65.5
66.0
Time (min)

Y2-CHO-S3 GluC

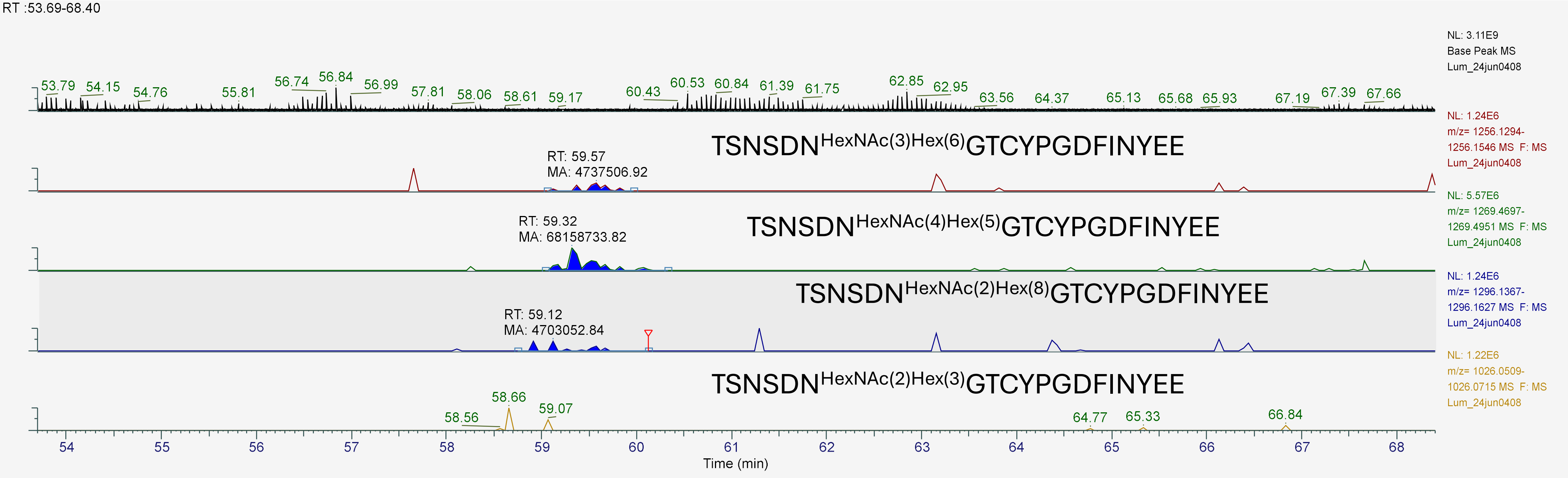

Y2-CHO-S3 GluC

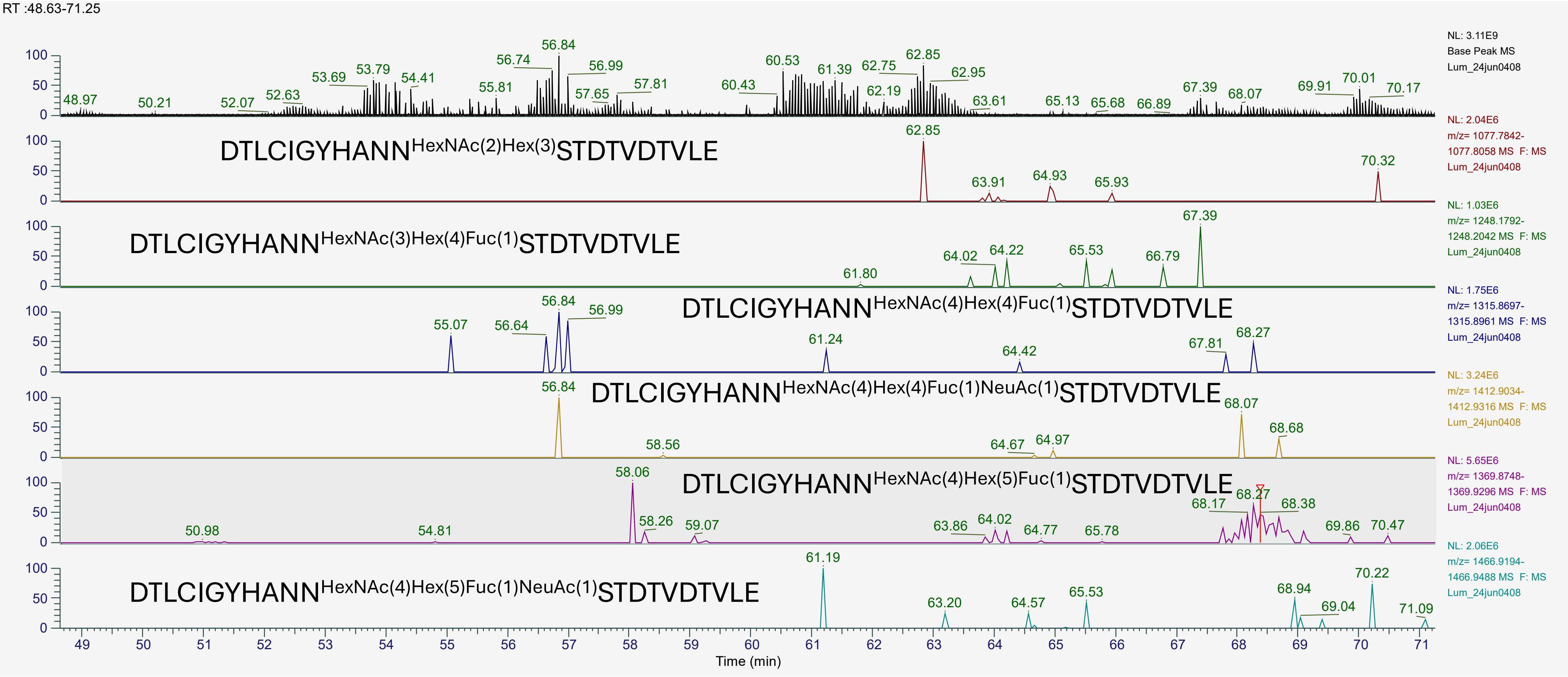

Y2-CHO-S3 GluC

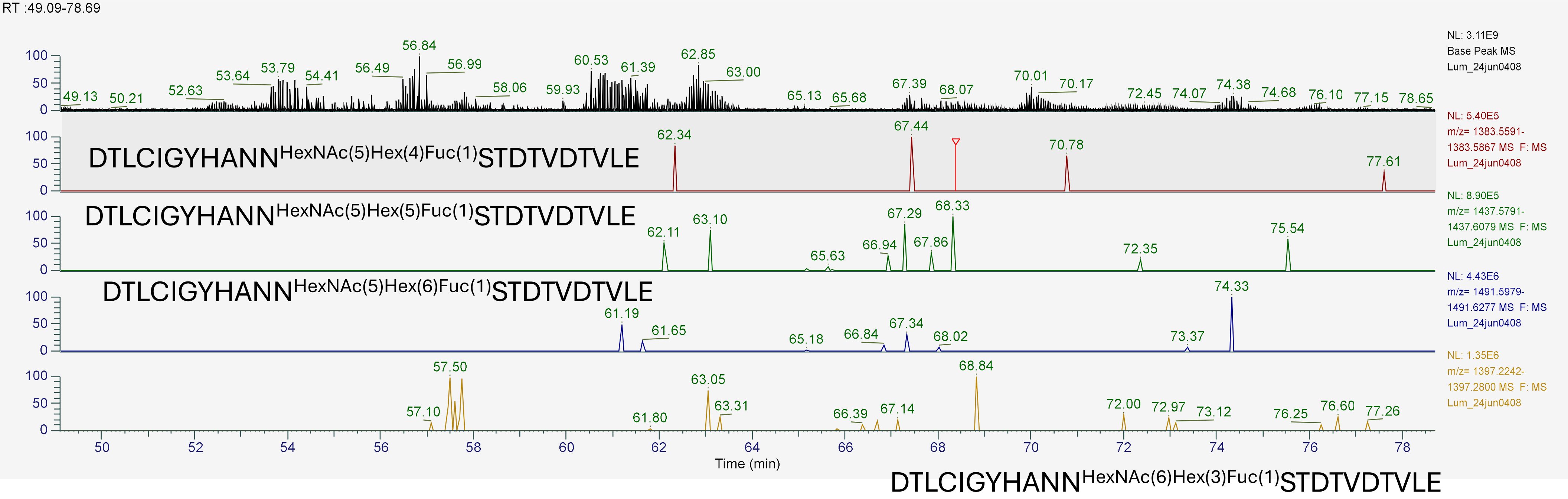

Y2-CHO-S3 Trypsin

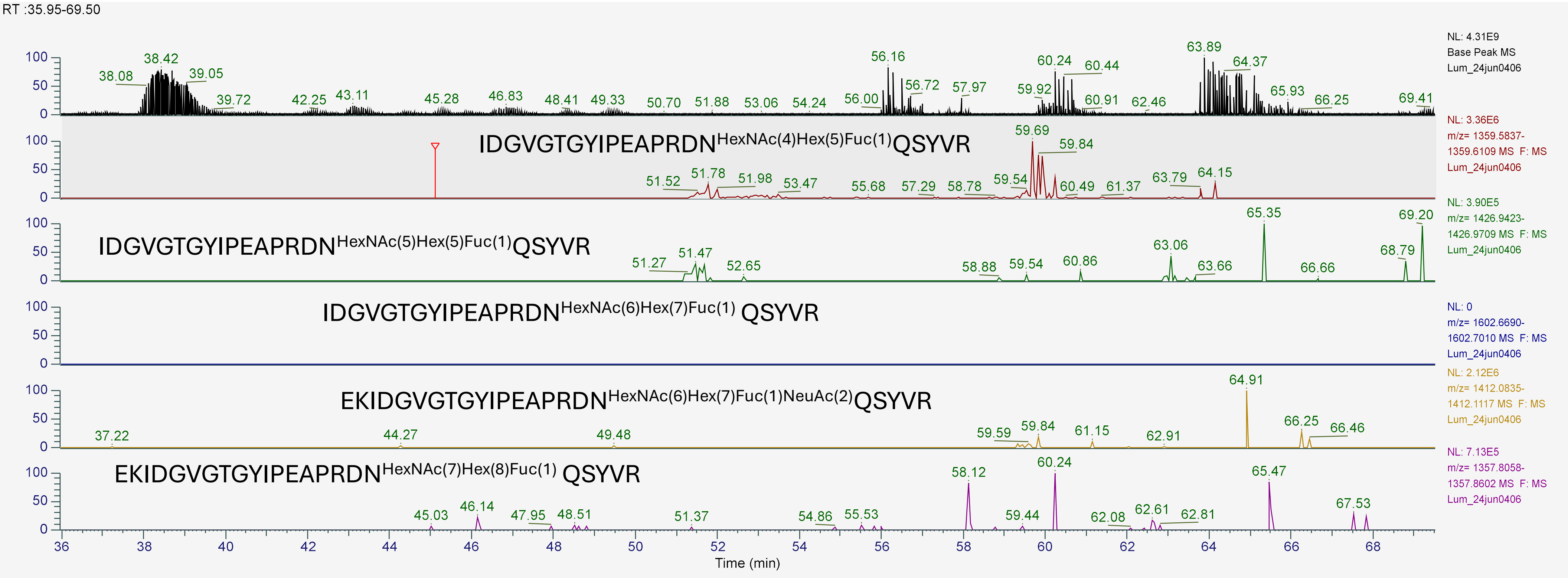

Y2-CHO-S3 GluC

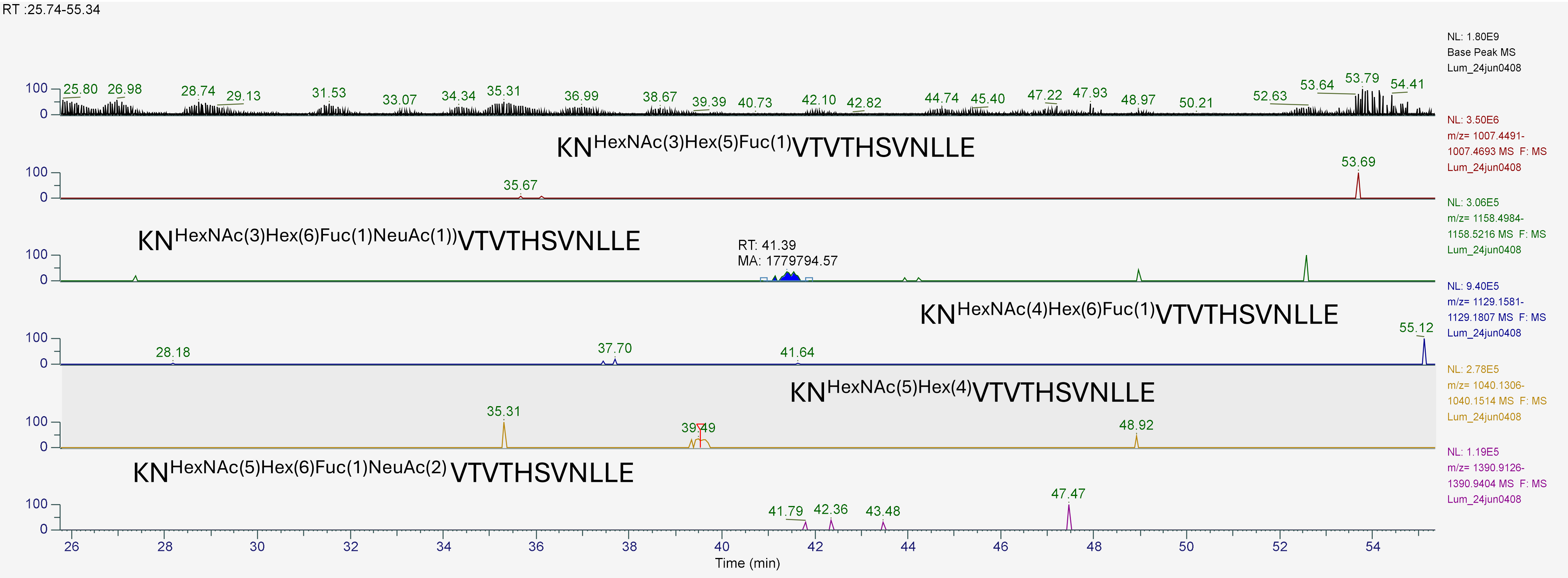

Y2-CHO-S3 GluC

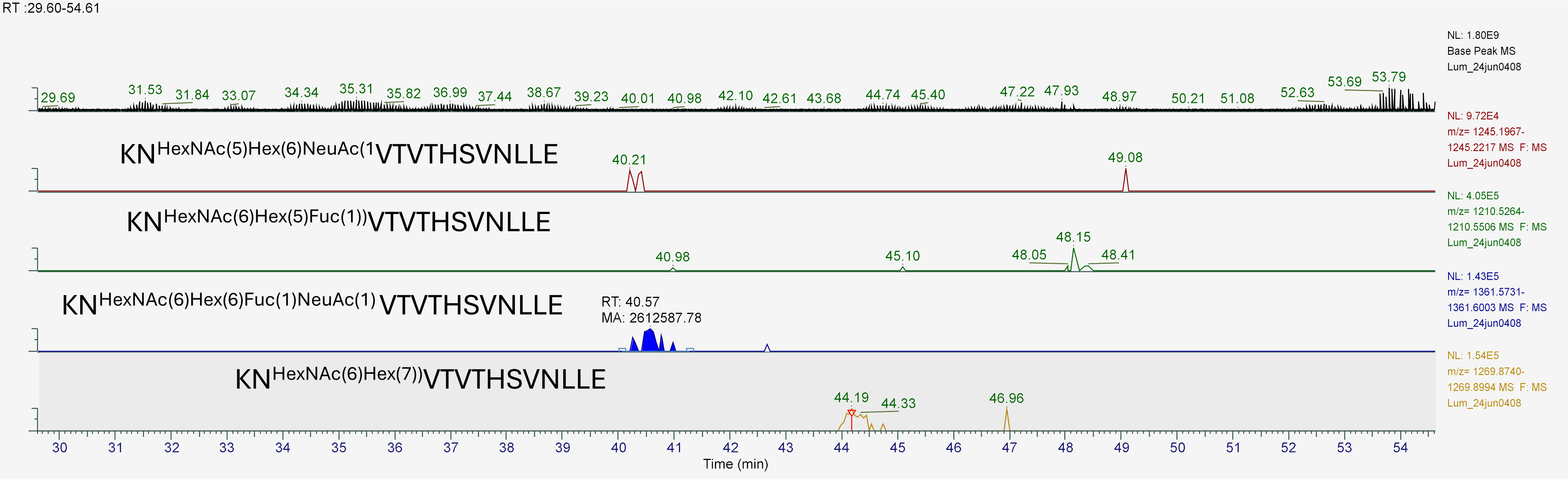

Y2-CHO-S3 GluC

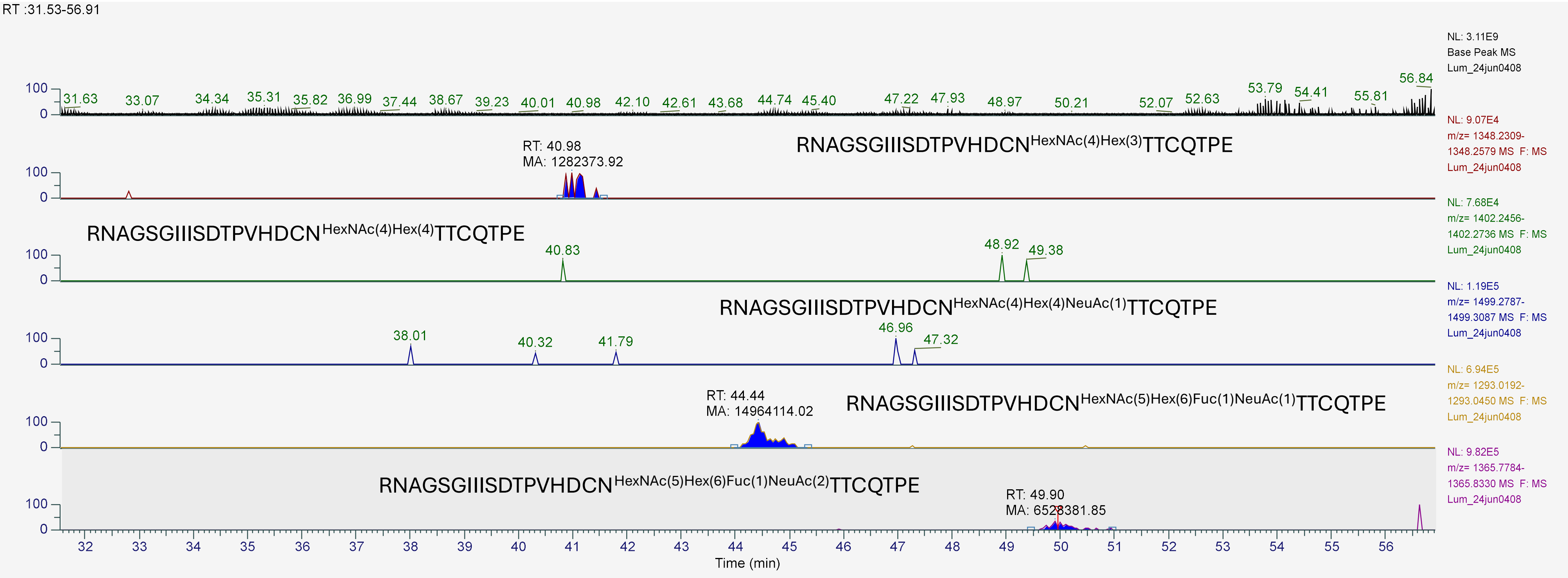

Y2-CHO-S3 GluC

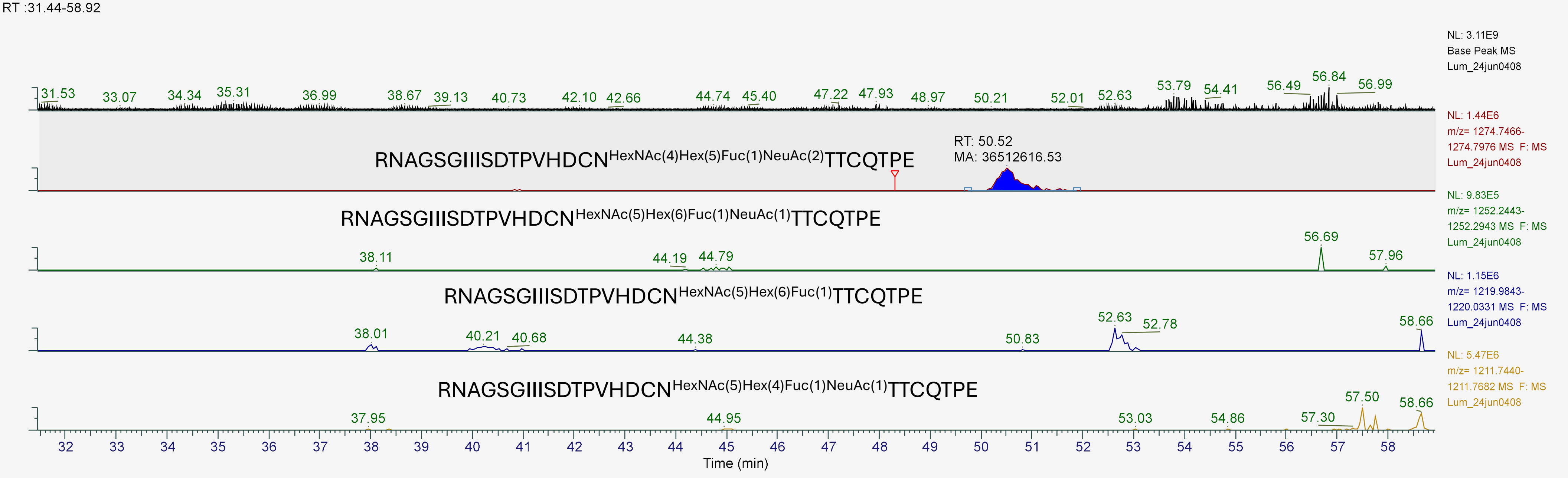

Y2-CHO-S3 GluC

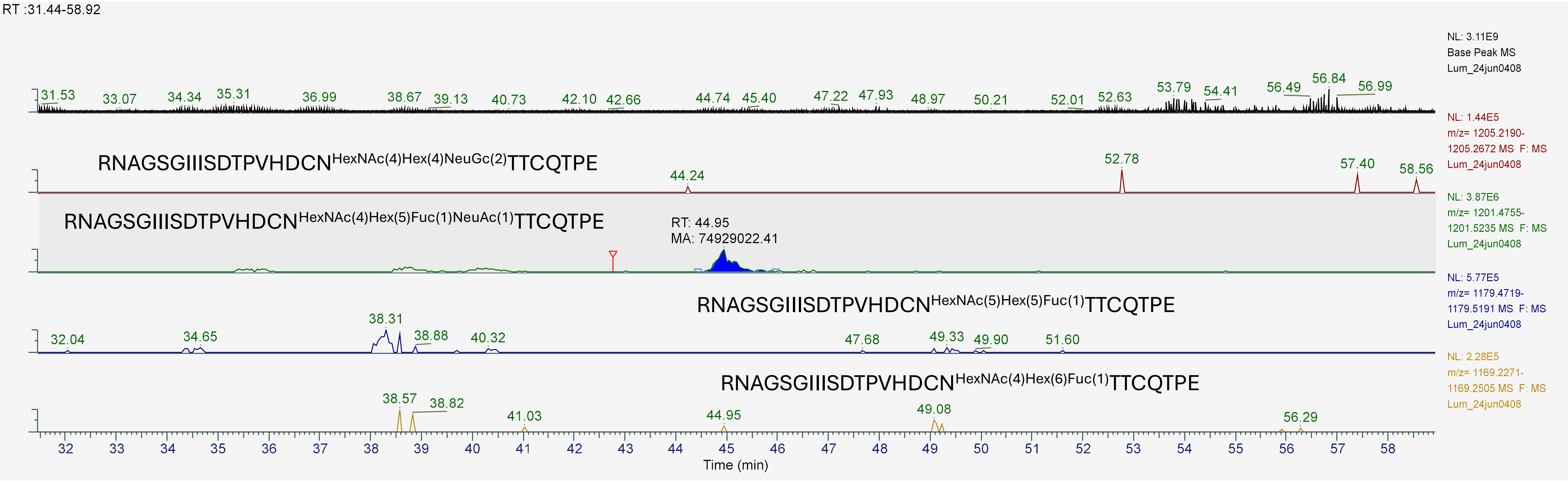

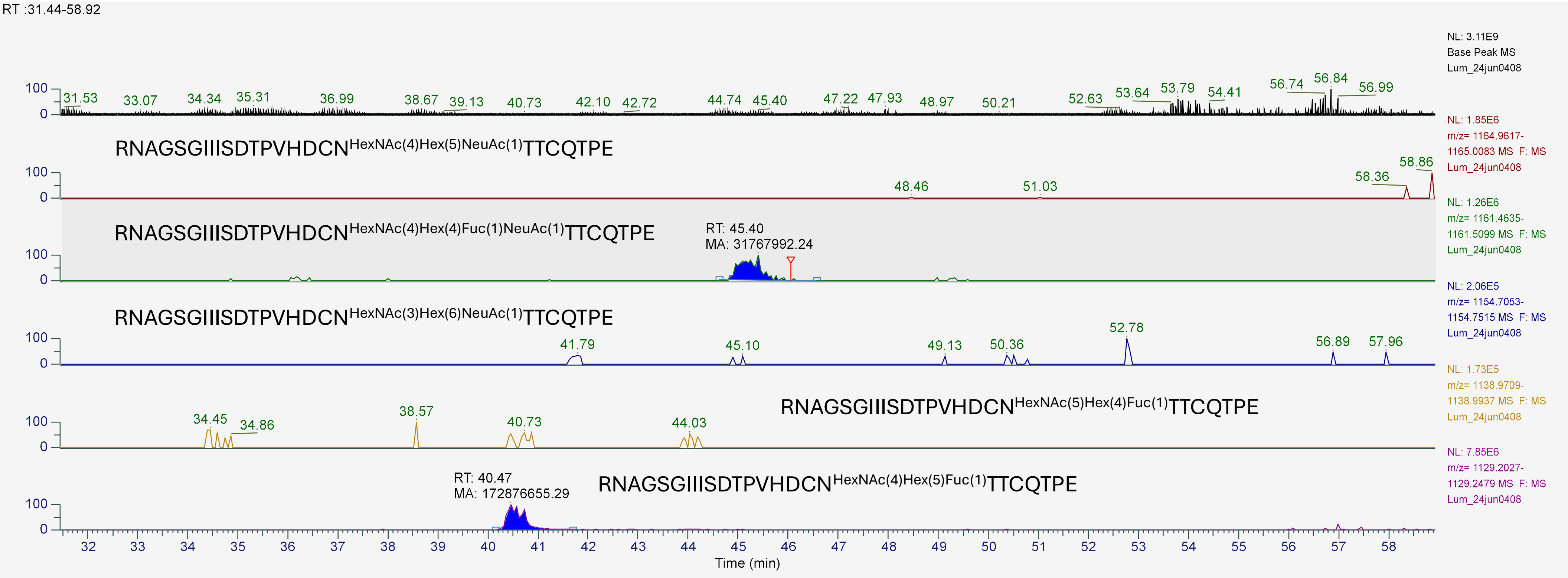
RT :31.44-58.92
NL: 3.11E9
Base Peak MS
Lum_24jun0408
RNAGSGIIISDTPVHDCN^HexNAc(4)Hex(5)NeuAc(1)TTCQTPE
NL: 1.85E6
m/z= 1164.9617-1165.0083 MS F: MS
Lum_24jun0408
RNAGSGIIISDTPVHDCN^HexNAc(4)Hex(4)Fuc(1)NeuAc(1)TTCQTPE
RT: 45.40
MA: 31767992.24
NL: 1.26E6
m/z= 1161.4635-1161.5099 MS F: MS
Lum_24jun0408
RNAGSGIIISDTPVHDCN^HexNAc(3)Hex(6)NeuAc(1)TTCQTPE
NL: 2.06E5
m/z= 1154.7053-1154.7515 MS F: MS
Lum_24jun0408
RNAGSGIIISDTPVHDCN^HexNAc(5)Hex(4)Fuc(1)TTCQTPE
NL: 1.73E5
m/z= 1138.9709-1138.9937 MS F: MS
Lum_24jun0408
RNAGSGIIISDTPVHDCN^HexNAc(4)Hex(5)Fuc(1)TTCQTPE
RT: 40.47
MA: 172876655.29
NL: 7.85E6
m/z= 1129.2027-1129.2479 MS F: MS
Lum_24jun0408
Time (min)

Y2-CHO-S3 GluC

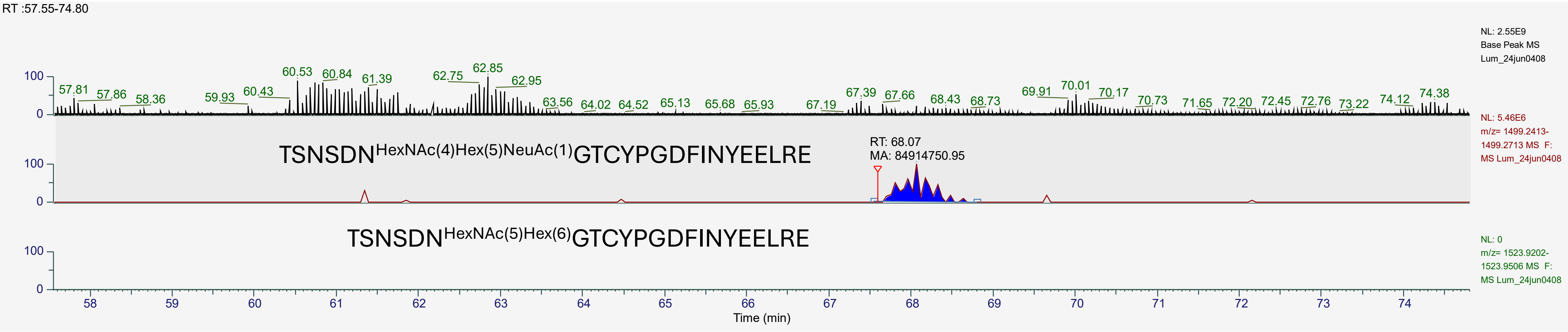

Y2-CHO-S3 GluC

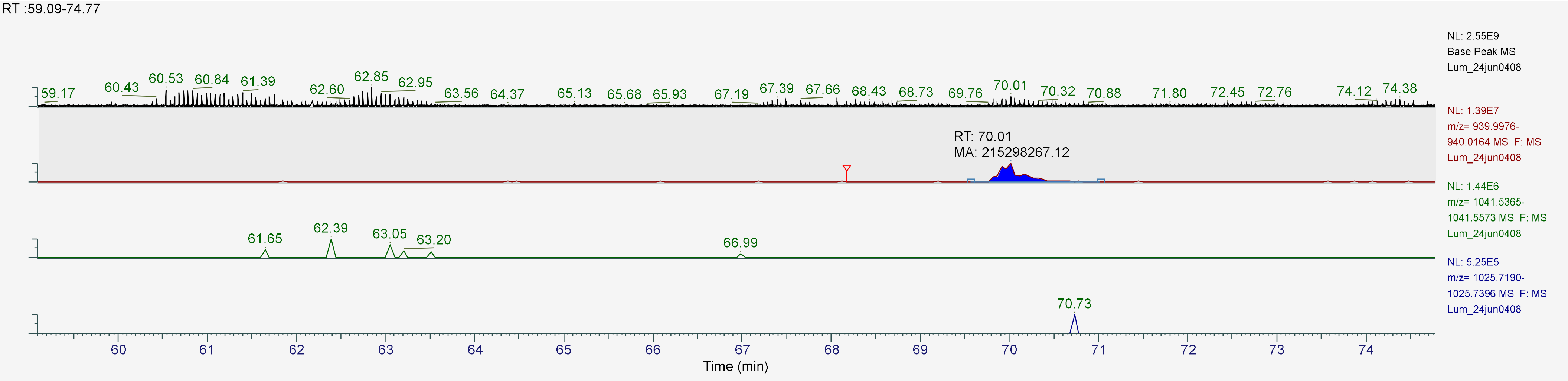

Y2-CHO-S3 GluC

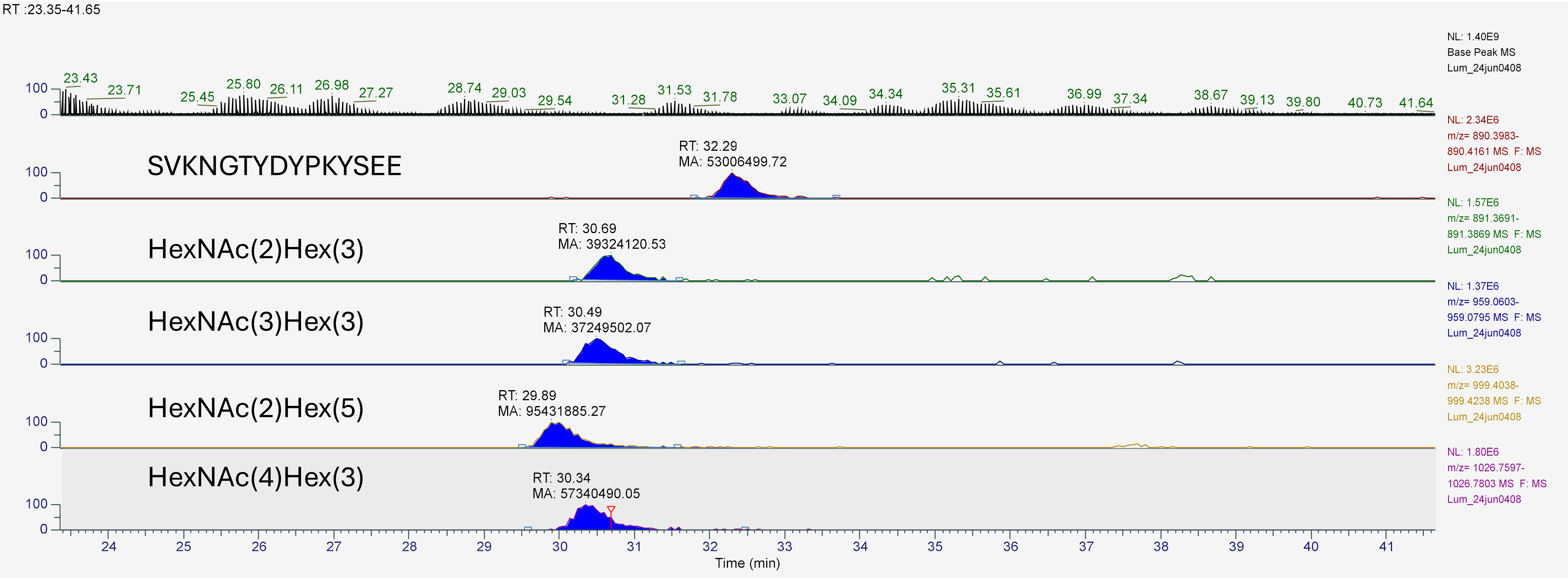

Y2-CHO-S3 GluC

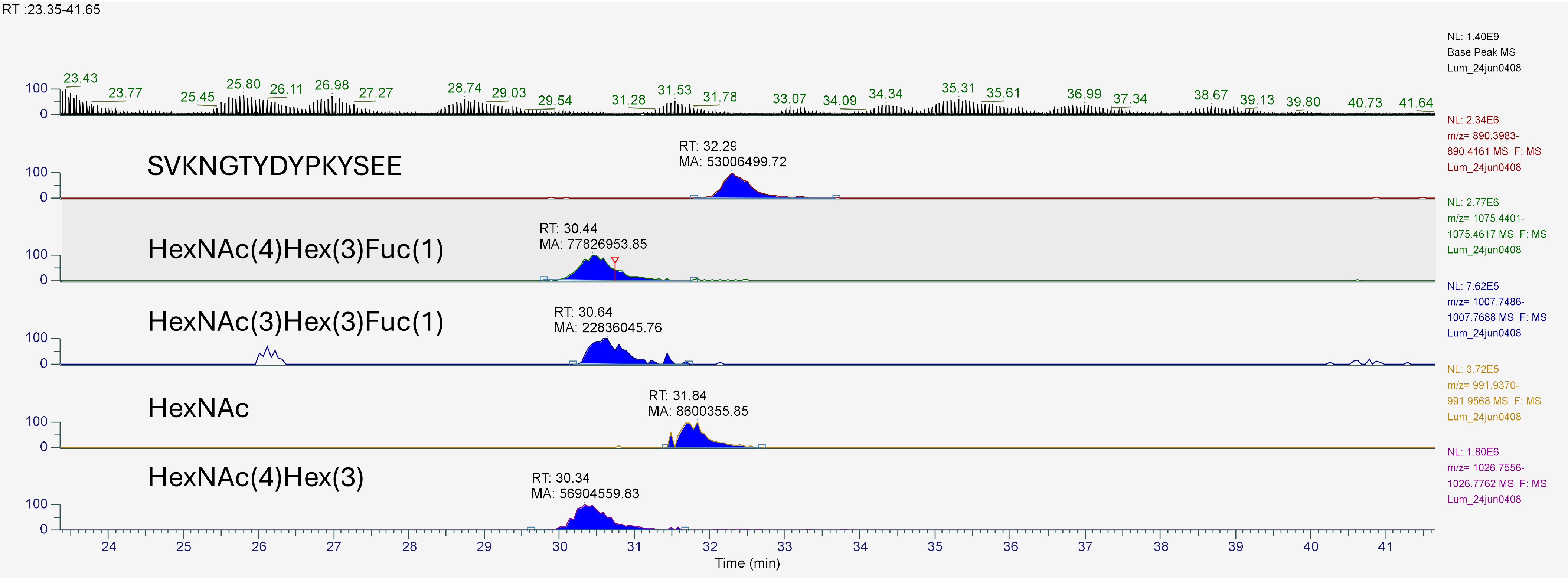

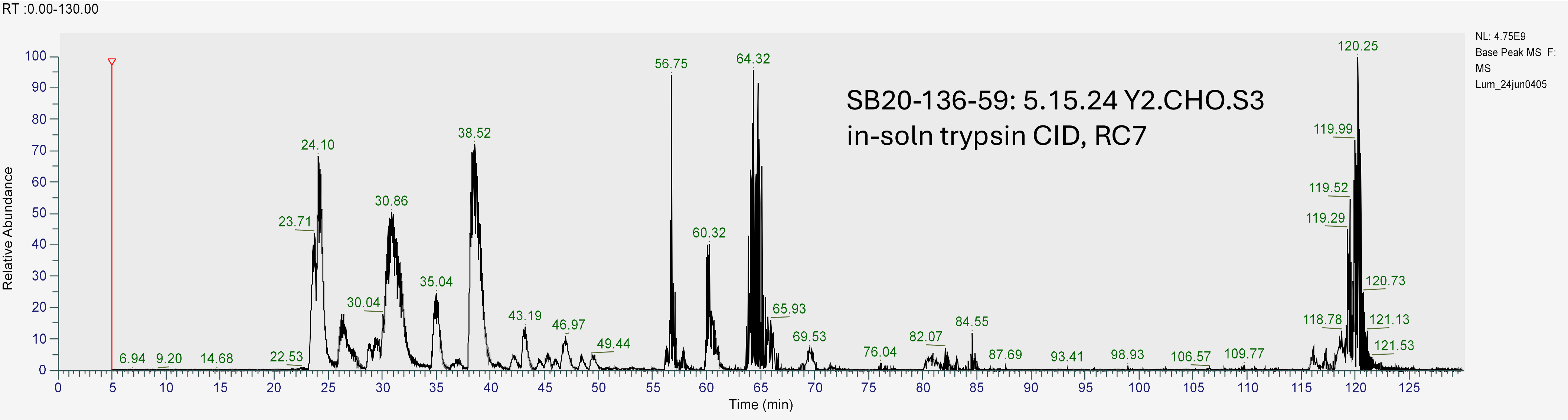
RT :0.00-130.00
NL: 4.75E9
Base Peak MS F: MS
Lum_24jun0405
SB20-136-59: 5.15.24 Y2.CHO.S3
in-soln trypsin CID, RC7
Relative Abundance
Time (min)
120.25
64.32
56.75
119.99
38.52
24.10
119.52
30.86
23.71
119.29
60.32
35.04
120.73
30.04
65.93
43.19
118.78
121.13
46.97
84.55
69.53
82.07
49.44
121.53
76.04
87.69
93.41
98.93
106.57
109.77
6.94
9.20
14.68
22.53

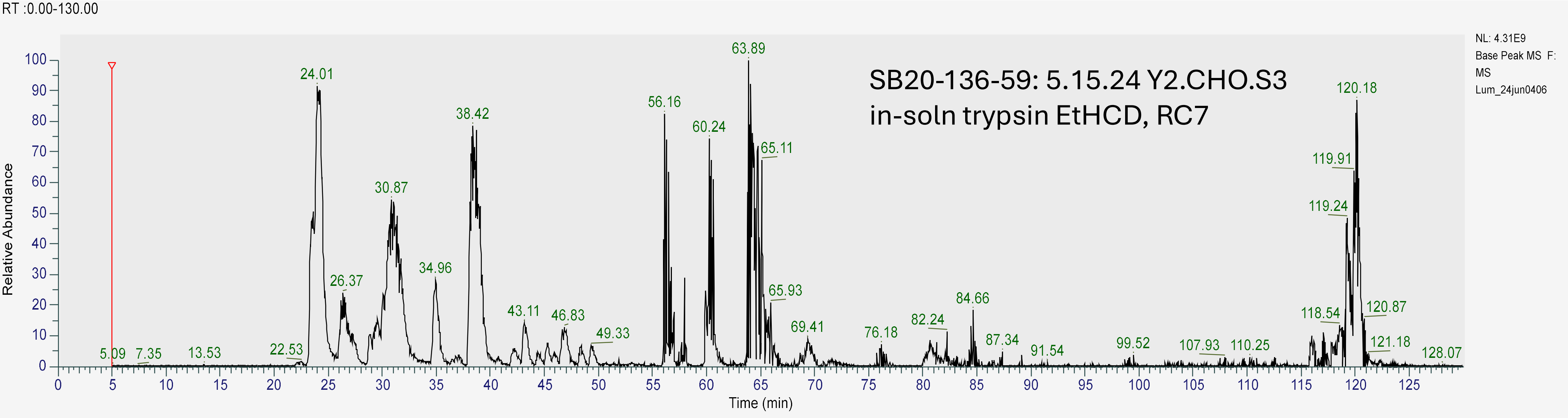
RT :0.00-130.00
NL: 4.31E9
Base Peak MS F: MS
Lum_24jun0406
SB20-136-59: 5.15.24 Y2.CHO.S3
in-soln trypsin EtHCD, RC7
Relative Abundance
Time (min)
63.89
24.01
120.18
56.16
38.42
60.24
65.11
119.91
30.87
119.24
34.96
26.37
65.93
84.66
120.87
118.54
43.11
46.83
82.24
49.33
69.41
87.34
121.18
76.18
99.52
107.93
110.25
91.54
128.07
5.09
7.35
13.53
22.53

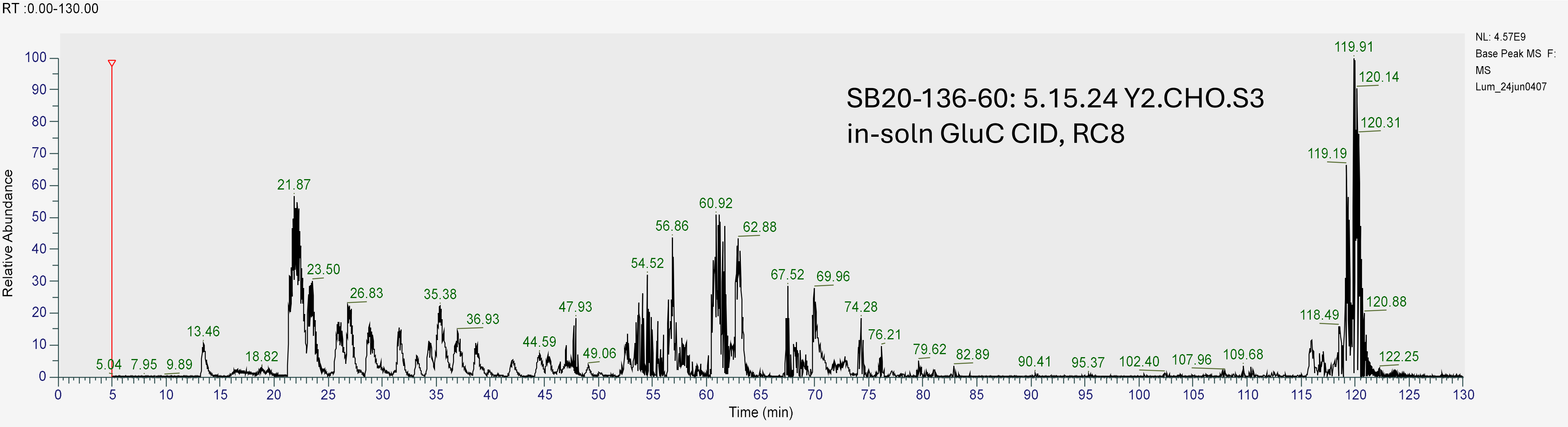
RT :0.00-130.00
SB20-136-60: 5.15.24 Y2.CHO.S3
in-soln GluC CID, RC8
NL: 4.57E9
Base Peak MS F: MS
Lum_24jun0407
Relative Abundance
Time (min)
119.91
120.14
120.31
119.19
21.87
60.92
56.86
62.88
54.52
23.50
67.52
69.96
26.83
35.38
36.93
47.93
74.28
120.88
118.49
13.46
44.59
76.21
79.62
82.89
49.06
18.82
5.04
7.95
9.89
90.41
95.37
102.40
107.96
109.68
122.25

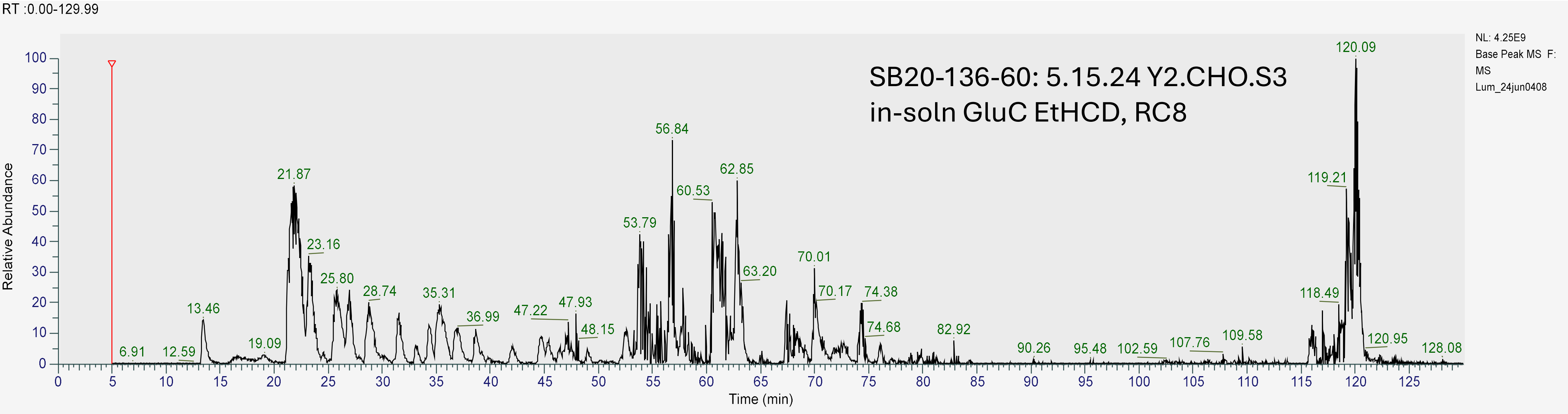
RT :0.00-129.99
SB20-136-60: 5.15.24 Y2.CHO.S3
in-soln GluC EtHCD, RC8
NL: 4.25E9
Base Peak MS F: MS
Lum_24jun0408
Relative Abundance
Time (min)
120.09
56.84
21.87
62.85
60.53
119.21
53.79
23.16
70.01
63.20
25.80
28.74
35.31
70.17
74.38
118.49
13.46
36.99
47.22
47.93
48.15
74.68
82.92
19.09
6.91
12.59
90.26
95.48
102.59
107.76
109.58
120.95
128.08
