# Supplementary material for: Immunogenicity of the Foldon trimerization domain and efforts to shield it
Source: PLoS One. 2026 Nov 13;21(11):e0362571. doi: 10.1371/journal.pone.0362571 (PMC13766278; doi:10.1371/journal.pone.0362571)

DGNWTLLSTFLGSGLNDIFEAQKIEWHEGHHH
HHH

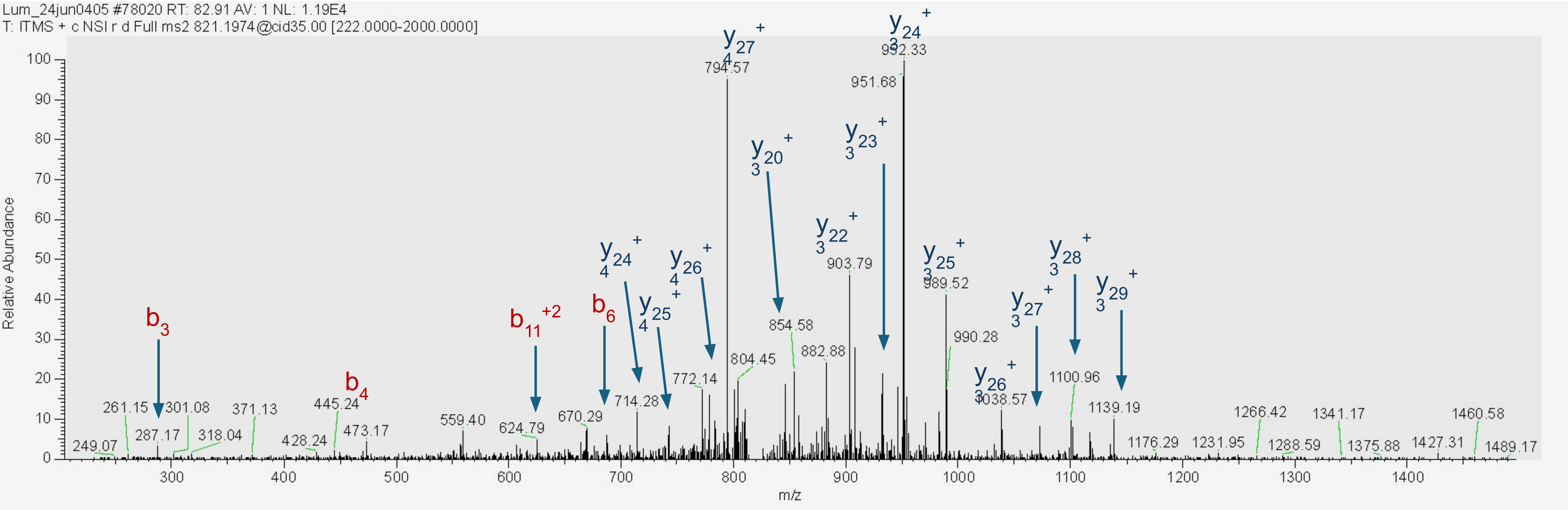

Supplement: Supplementary file 6 [file pone.0362571.s005.zip › S11_Data/Ross_5232_N524.pptx.pdf]
